# IGFBP5 is released by senescent cells and is internalized by healthy cells, promoting their senescence through interaction with retinoic receptors

**DOI:** 10.1186/s12964-024-01469-1

**Published:** 2024-02-13

**Authors:** Nicola Alessio, Domenico Aprile, Gianfranco Peluso, Valeria Mazzone, Deanira Patrone, Giovanni Di Bernardo, Umberto Galderisi

**Affiliations:** 1 Department of Experimental Medicine, Luigi Vanvitelli Campania University, via Luigi De Crecchio 7, Naples, 80138 Italy; 2 Genome and Stem Cell Center (GENKÖK), Erciyes University, Kayseri, Turkey; 3 Center for Biotechnology, Sbarro Institute for Cancer Research and Molecular Medicine Temple University, PA Philadelphia, USA; 4 International Medical University UNICAMILLUS, Rome, Italy

**Keywords:** Senescence, Secretome, SASP, IGFBP, Mesenchymal stromal cells

## Abstract

Cells that are exposed to harmful genetic damage, either from internal or external sources, may undergo senescence if they are unable to repair their DNA. Senescence, characterized by a state of irreversible growth arrest, can spread to neighboring cells through a process known as the senescence-associated secretory phenotype (SASP). This phenomenon contributes to both aging and the development of cancer. The SASP comprises a variety of factors that regulate numerous functions, including the induction of secondary senescence, modulation of immune system activity, remodeling of the extracellular matrix, alteration of tissue structure, and promotion of cancer progression. Identifying key factors within the SASP is crucial for understanding the underlying mechanisms of senescence and developing effective strategies to counteract cellular senescence. Our research has specifically focused on investigating the role of IGFBP5, a component of the SASP observed in various experimental models and conditions.

Through our studies, we have demonstrated that IGFBP5 actively contributes to promoting senescence and can induce senescence in neighboring cells. We have gained valuable insights into the mechanisms through which IGFBP5 exerts its pro-senescence effects. These mechanisms include its release following genotoxic stress, involvement in signaling pathways mediated by reactive oxygen species and prostaglandins, internalization via specialized structures called caveolae, and interaction with a specific protein known as RARα. By uncovering these mechanisms, we have advanced our understanding of the intricate role of IGFBP5 in the senescence process. The significance of IGFBP5 as a pro-aging factor stems from an in vivo study we conducted on patients undergoing Computer Tomography analysis. In these patients, we observed an elevation in circulating IGFBP5 levels in response to radiation-induced organismal stress.

Globally, our findings highlight the potential of IGFBP5 as a promising therapeutic target for age-related diseases and cancer.

**Supplementary Information:**

The online version contains supplementary material available at 10.1186/s12964-024-01469-1.

## Introduction

Cells experiencing genotoxic stress, either from endogenous or exogenous stimuli, may enter senescence if they cannot properly repair damaged DNA. The senescent executive program promotes cell cycle arrest, chromatin remodeling, the onset of senescence-related gene expression patterns, and the release of various factors collectively referred to as the senescence-specific associate phenotype (SASP) [1, 2]. SASP facilitates the spread of senescence from directly affected cells (primary senescence), which were directly hit by genotoxic agents, to neighboring and even distant cells. These cells experience what is known as secondary senescence [3].

Both primary and secondary senescence may have beneficial effects on health, including blocking cancer onset, promoting wound healing, and contributing to tissue development. These effects are thought to be attributed to the early stages of senescence. However, if senescent cells are not eliminated by the immune system, they undergo changes in their functions and SASP composition over time. Consequently, they transition into late/deep senescent cells, which exhibit pro-tumorigenic activity, sustain chronic inflammation, and contribute to organismal aging [2, 4–6]. Analyzing the composition of SASP and how it changes as cells progress from the early to late stage of senescence is of great interest. However, the content and modifications of SASP depend on several factors, including cell type, the quality and intensity of genotoxic stress, and time [6–9], which makes studies on SASP challenging.

Another aspect to consider is the difficulty in identifying among the numerous SASP factors which ones have a causative role in sustaining primary senescence (autocrine factors) or promoting secondary senescence (paracrine factors).

In our previous studies, we observed the presence of certain components of insulin-like growth factor binding proteins (IGFBPs) in the SASP of senescent mesenchymal stromal cells (MSCs) [10–12]. Cynthia Kenyon coined the term 'conserved regulatory system for aging' to describe a biological mechanism that has remained unchanged throughout evolution, which includes insulin growth factors (IGFs) and the related IGFBPs [13]. We demonstrated a causative role for IGFBP4 and IGFBP7 in triggering secondary senescence since their inactivation in SASP abolished its pro-senescent effect, while their supplementation in a culture medium or in vivo promoted senescence [11, 14].

To further investigate the role of IGFBP5 in senescence and its potential involvement in spreading the senescence process, we focused our attention on this factor. The biological function of IGFBP5 remains controversial in the literature. Some studies suggest that IGFBP5 may induce senescence via STAT3 signaling or P53-related pathways, while others indicate an increase in IGFBP5 levels in senescent cells following radiation exposure or treatment with kinase inhibitors. Conversely, there are reports associating downregulation of IGFBP5 with senescence. Moreover, the role of IGFBP5 in cancer progression appears to be context-dependent [15–20].

The multiple and even contradictory biological functions attributed to IGFBP5 may be related to its involvement in various signaling pathways. IGFBP5 can bind and sequester IGFs, thereby preventing their interaction with IGF receptors. Alternatively, by binding IGFs, IGFBP5 can protect them from degradation and gradually allow their release and interaction with cognate receptors [21, 22]. IGFBP5 may also have its own receptor, as studies suggest its binding to the LRP1 cell surface receptor (also known as Type V transforming growth factor β receptor) [22, 23]. Furthermore, it has been observed that secreted IGFBP3 and IGFBP5 can localize within the nucleus [24]. For IGFBP3, nuclear localization may occur via caveolae endocytosis [25]. However, there is currently no data regarding IGFBP5 internalization via caveolae, although studies have shown that a decrease in CAVEOLIN-1 expression contributes to the extracellular deposition of IGFBP5 [26].

Our aim is to demonstrate the causative role of IGFBP5 in senescence by evaluating its release following genotoxic stress and investigating the signaling pathways that promote its paracrine pro-senescent activity.

## Results

We utilized mesenchymal stromal cells (MSCs) as an experimental model because these cells play a crucial role in maintaining body homeostasis. MSCs are a heterogeneous population consisting of stem cells, progenitor cells, fibroblasts, and stromal cells. They are found in the bone marrow and in the stromal component of almost every tissue [27]. After tissue injury, MSCs release paracrine factors that promote tissue repair and modulate inflammation. However, the senescence of MSCs impairs their physiological function and alters the composition of their secretome, leading to the development of the senescence-associated secretory phenotype (SASP) [28].

In a previous study, we demonstrated that the SASP of senescent MSCs can induce secondary senescence in other MSCs. This function was dependent on the presence of two SASP components, IGFBP4 and IGFBP7, as neutralizing antibodies against these factors abolished the induction of secondary senescence. Furthermore, incubation of healthy MSCs with either IGFBP4 or IGFBP7 alone resulted in cellular senescence [14].

Given that IGFBP5 is also present in the SASP of MSCs, we aimed to investigate whether it possesses the same biological property.

### Paracrine pro-senescence effect of IGFBP5

**Fig. 1:**
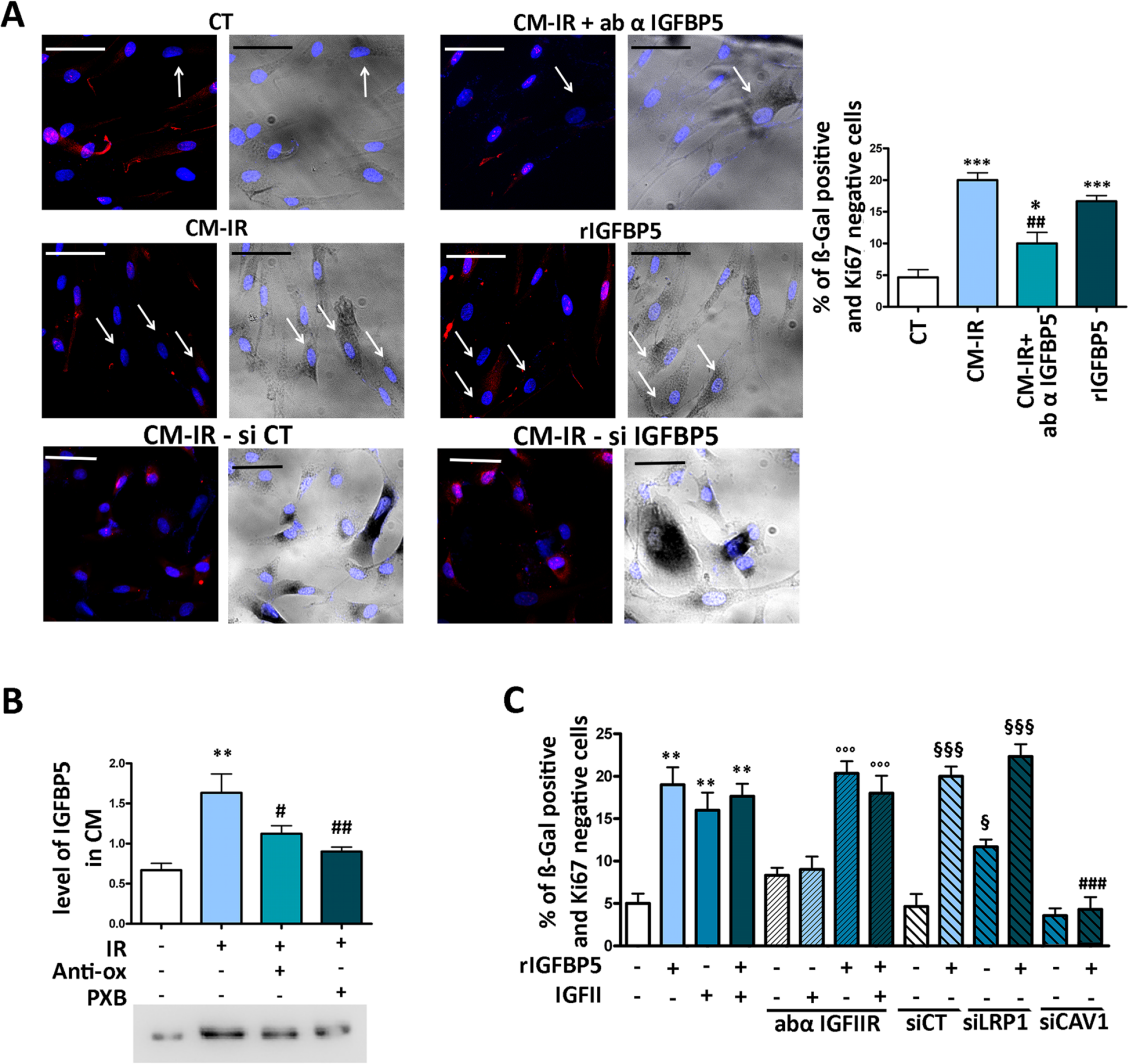
Release of IGFBP5 in SASP and its effect on senescence. **A** Representative micrographs of MSCs stained to identify nuclei (DAPI), Ki67 (red), and to evaluate β-galactosidase activity (dark gray). The white arrows indicate senescent cells, which are β-galactosidase positive (β-gal +) and Ki67 negative (Ki67-). We employed a Leica CTR500 microscope, which was equipped with a DCF3000G digital monochrome camera. The β-galactosidase activity was captured as a gray-stain using this configuration. This experimental method allowed us to identify cells that exhibited a visible light signal β-galactosidase along with others expressing fluorescent signals within the same cell. CT: untreated cells; CM-IR: cells treated with conditioned medium (CM) collected from irradiated (IR) cells; CM-IR + ab αIGFBP5: cells treated with CM in the presence of IGFBP5 neutralizing antibodies; rIGFBP5: cells incubated with recombinant IGFBP5. The scale bar corresponds to 100 microns. The graph shows the percentage of senescent cells under different experimental conditions. The symbols *** *p* < 0.001 and * *p* < 0.05 indicate statistical significance between the control (CT) and treated samples. The symbol ## *p* < 0.01 indicates statistical significance between the CM-IR sample, chosen as a reference, and CM-IR + ab αIGFBP5. **B** The graph shows the level of IGFBP5 in CM of MSCs 24 h following irradiation (IR). IR: irradiated MSCs; Anti-oxi: the irradiated MSCs were treated with an anti-oxidant mixture; PXB: irradiated cells were incubated with a drug inhibiting COX2 activity. The symbol ** *p* < 0.01 indicates statistical significance between the control (CT) and irradiated samples. The symbols ## *p* < 0.01 and # *p* < 0.05 indicate statistical significance between the IR sample (second column), chosen as a reference, and IR + Anti-oxi or IR + PXB. The western blot under the graph shows a representative image of IGFBP5 immunodetection. **C** The graph shows the percentage of senescent cells (β-galactosidase positive and Ki67 negative) under different experimental conditions. rIGFBP5: cells incubated with recombinant IGFBP5; IGFII: cell treated with IGFII either in presence or absence of antibody against IGFIIR (abα IGFIIR); siLRP1: siRNA targeting LRP1 mRNA; siCAV1: siRNA targeting CAVEOLIN-1; siCTR: control siRNA. The symbol ** *p* < 0.01 indicates statistical significance between the control (first column) and samples (from second to fourth columns) treated with rIGFBP5 and/or IGFII. The symbol °°° *p* < 0.001 indicates statistical significance between cells treated with abα IGFIIR alone (chosen as reference, see fifth column) and the other samples treated with the antibody in presence of rIGFBP5 and/or IGFII (from sixth to eight columns). The symbols §§§ *p* < 0.001 indicate and § *p* < 0.05 indicate statistical significance between the sample treated with siCT (chosen as the reference, see ninth column) and the other siRNA treated samples, reported in columns from the 10th to 12th. The symbol ## *p* < 0.01 indicates the statistical difference between the sample treated with genistein (Gen) in presence of IGFBP5 and the one treated with IGFBP5 only (second column from left), chosen as reference

We subjected human MSCs at passage 3 to a dose of 2000 mGy X-ray radiation to induce senescence, as reported in Supplementary file 1. Subsequently, we collected the SASP according to the methods outlined. In the SASP of irradiated cells we detected an increase in IGFBP5 levels (Fig. 1B). Healthy MSCs were then incubated for 72 h with a mixture consisting of 50% SASP and 50% culture medium, either in the presence or absence of IGFBP5 neutralizing antibodies. We evaluated the percentage of senescent cells based on β-galactosidase positivity and Ki67 negativity. The inactivation of IGFBP5 using neutralizing antibodies significantly reduced the percentage of senescent cells (Fig. 1A). Conversely, we observed an increase in senescence in healthy MSC cultures incubated with 35 ng/ml of IGFBP5 for 72 h (Fig. 1A). These results suggest that IGFBP5 can play an active role in triggering senescence through a paracrine mechanism.

To assess whether the function of IGFBP5 is cell type and stress-specific or if it plays a more general role, we treated human dermal fibroblasts (HDF) with 300 μM H_2_O_2_ for 30 min. Subsequently, 48 h later, we collected the SASP, and repeated experiments outlined in Fig. 1A. The results (Supplementary File  5) confirmed that IGFBP5 may play a general role in senescence.

One common characteristic of stressors that induce senescence is their ability to elevate the levels of reactive oxygen species (ROS) within cells. This increase in ROS levels is typically followed by the release of prostaglandins, with PGE2 being a specific example [11, 29, 30]. In line with this, we found that the levels of IGFBP5 in the medium collected from irradiated MSCs significantly decreased when cells were incubated with antioxidants or Parecoxib, an anti-COX2 drug, following irradiation (Fig. 1B). This result aligns with the notion that IGFBP5 secretion is induced by ROS-Prostaglandin signaling, as we previously demonstrated for IGFBP4 [11].

### IGBP5 signaling circuits associated with senescence

The incubation of MSC cultures with IGFBP5 promotes the onset of senescence. Interestingly, the percentage of senescent cells in IGFBP5-treated cultures remained unchanged even with the supplementation of IGFII (Fig. 1C), which we previously demonstrated can induce senescence in healthy MSCs by binding to its IGFIIR cognate receptor [11] (Fig. 1C). Furthermore, the IGFBP5-induced senescence was not affected by the simultaneous presence of IGFII and/or IGFIIR neutralizing antibodies (Fig. 1C). These data suggest that IGFBP5 has an IGFII-independent pro-senescence activity.

Another possible pathway for IGFBP5-induced senescence could involve interactions with the LRP1 receptor. LRP1 is a versatile member of the LDL receptor family and is expressed in various tissues. It fulfills two primary biological roles: the internalization of numerous ligands and the regulation of cell signaling pathways [31]. To investigate this, we silenced LRP1 expression using specific siRNAs (Supplementary file 3A) and incubated MSCs with IGFBP5 for 72 h to evaluate its effects on senescence. Surprisingly, silencing LRP1 significantly increased the percentage of senescent cells in IGFBP5-treated cultures (Fig. 1C). This result suggests that the LRP1 signaling pathway does not play a role in the senescence induced by IGFBP5. Another hypothesis could propose that the suppression of LRP1, which potentially removes a viable receptor for IGFBP5, results in an increase in the quantity of accessible IGFBP5 molecules for binding with various receptors. Some of these receptors may be involved in signaling pathways related to senescence.

### IGFBP5 reaches nuclei via caveolae

We also examined the possibility that IGFBP5 internalization via caveolae could be the route through which senescence is induced.

Caveolin gene family members serve as the primary structural proteins found within caveolae. These members are small integral membrane proteins that become embedded in the inner leaflet of caveolae. Notably, the expression of CAVEOLIN-1 has been identified as both essential and adequate for the creation of well-defined morphological caveolae [32].

The silencing of CAVEOLIN-1 with siRNA (Supplementary file  3C) impaired the senescence process triggered by IGFBP5 in MSC cultures (Fig. 1C). This outcome suggests that the caveolae pathway might serve as the mechanism by which IGFBP5 induces senescence.

**Fig. 2:**
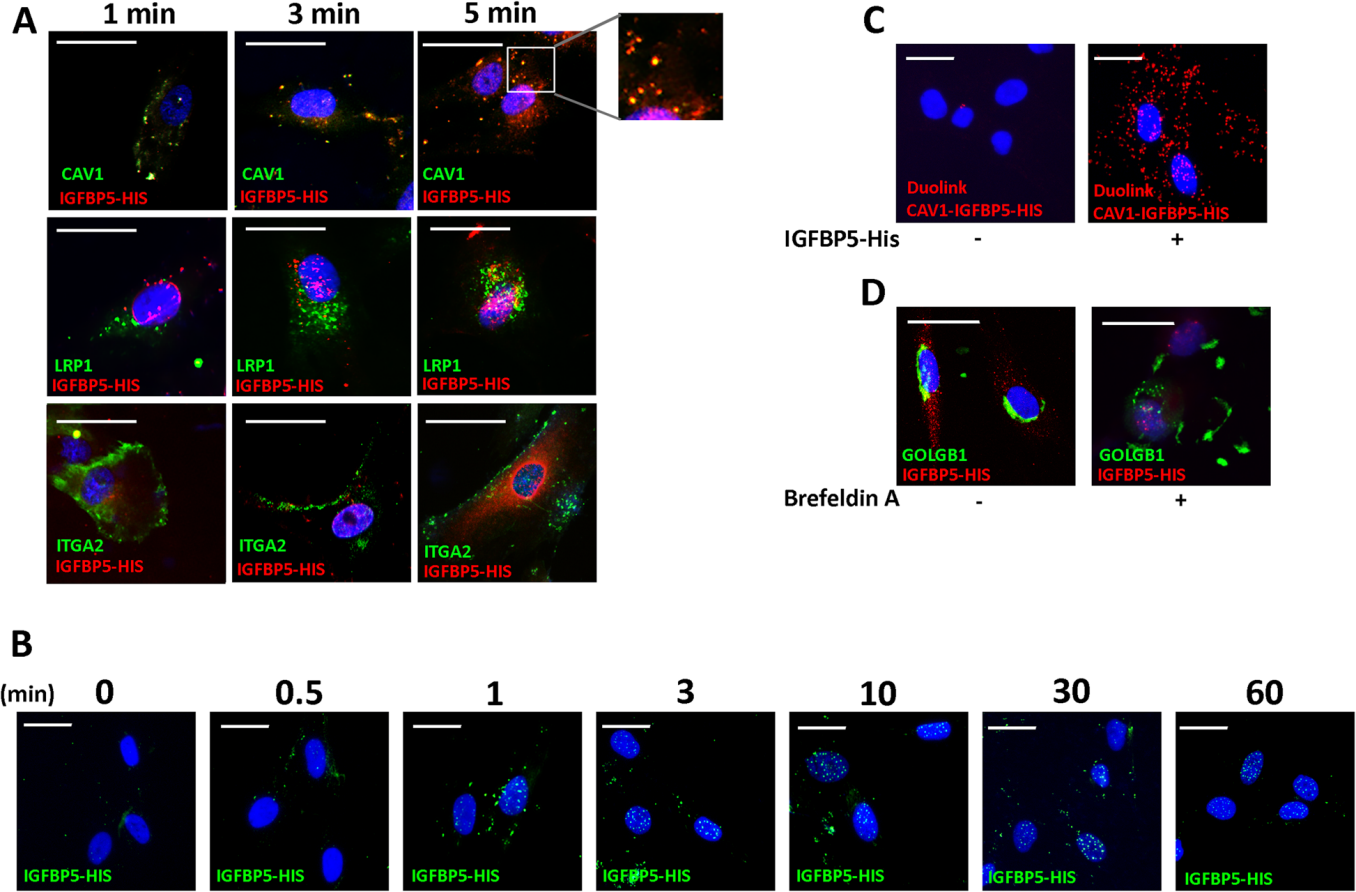
IGFBP5 internalization. **A** Representative images of MSCs incubated with His-tag-IGFBP5 (IGFBP5-HIS) and stained to identify nuclei (DAPI) and His-tag-IGFBP5 (red). Additionally, CAVEOLIN-1 (CAV1), LRP1, or ITGA2 were stained green. The pictures were taken at 1, 3, and 5 min after IGFBP5 incubation. The inset shows magnified images of IGFBP5 and CAVEOLIN-1 co-localization. The scale bar corresponds to 100 microns. **B** Representative images of MSCs incubated with His-tag-IGFBP5 and stained to identify nuclei (DAPI) and His-tag-IGFBP5 (green). The pictures were taken at 0, 0.5, 1, 3, 10, 30 and 60 min after IGFBP5 incubation. The scale bar corresponds to 100 microns.**C** Representative images of Duolink assay to identify physical proximity between IGFBP5 and CAVEOLIN-1. The red staining indicates a close interaction between IGFBP5 and CAVEOLIN-1 5 min following MSCs incubation with His-tag-IGFBP5. The nuclei were stained with DAPI (blue). The scale bar corresponds to 100 microns. **D** Representative images of MSCs incubated with His-tag-IGFBP5, either in presence or absence of Brefeldin A, and stained to identify nuclei (DAPI) and His-tag-IGFBP5 (red). Additionally, GOLGB1 was stained green. The pictures were taken at 3 min after IGFBP5 incubation. The scale bar corresponds to 100 microns

We then followed the internal route of IGFBP5. In our experiment, we exposed MSCs to IGFBP5 for different durations (1, 3, and 5 min) and subsequently conducted immunocytochemistry analysis. The results of this investigation revealed co-localization of IGFBP5 with CAVEOLIN-1, with a maximum signal intensity observed at 1 and 3 min (Fig. 2A).

IGFBP5 did not co-localize with LRP1, which can represent another caveolae-associated receptor [31], nor with ITGA2, an integrin involved in clathrin-mediated endocytosis [33] (Fig. 2A).

To further validate the proximity between IGFBP5 and CAVEOLIN-1, we performed the Duolink® Proximity Ligation Assay. This assay enables the detection of natural protein–protein interactions by emitting a fluorescent signal for each individual interaction event, which can be visualized through microscopy. Remarkably, we observed a robust signal in MSC cells cultured in the presence of IGFBP5, while the signal was nearly absent in samples grown without IGFBP5 (Fig. 2C). The silencing of CAVEOLIN-1 with siRNA did not permit the uptake of IGFBP5 and its localization within nuclei (Supplementary file  2A). This result further confirms that IGFBP5 enters cells via the caveolar route through interaction with CAVEOLIN-1.

Taken together, these findings strongly indicate that IGFBP5 can enter cells via the caveolae pathway.

To determine the ultimate location of internalized IGFBP5, we conducted an experiment where MSCs were exposed to IGFBP5 for varying durations: 30 s, 1 min, 3 min, 10 min, 30 min, and 60 min. Interestingly, we observed a significant IGFBP5 signal within the nuclei of the cells at the 10-min mark, followed by a gradual decrease in signal intensity thereafter (Fig. 2B).

We observed nuclear localization of recombinant His-tagged IGFBP5 following its application to normal MSCs, which subsequently became senescent. However, this result does not prove that native IGFBP5 at physiological levels in the SASP undergoes nuclear translocation when taken up by target cells. Then, we added non-tagged IGFBP5 to MSC culture and followed the uptake. Furthermore, we added the conditioned medium (CM) of senescent MSCs (gamma ray irradiated), which contains IGFBP5, to healthy MSCs and followed the uptake. In this scenario, we cannot discriminate between endogenous and external IGFBP5, so nuclear localization could be due to internal and/or exogenous IGFBP5. The experiment with His-tagged IGFBP5 suggests that only exogenous IGFBP5 can translocate within the nucleus (Supplementary file 2B-C). The performed experiments align with the previous ones.

Globally, our observations suggest that IGFBP5 likely reaches the nuclei via a direct process of caveolae-mediated nuclear internalization. This conclusion is supported by the lack of colocalization between IGFBP5 and markers for early/late endosomes or the Golgi apparatus. Additionally, when we treated the cells with Brefeldin A (BFA), a compound known to inhibit protein transport from the endoplasmic reticulum (ER) and disrupt the structure of the Golgi complex [34], we did not observe any hindrance in the nuclear localization of IGFBP5 (Fig. 2D).

**Fig. 3:**
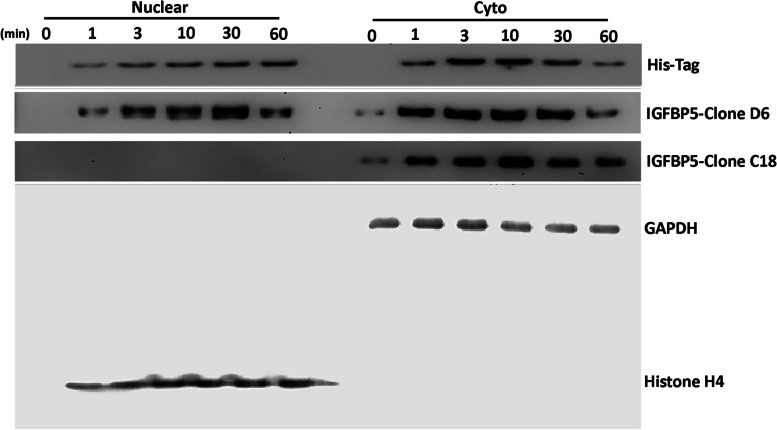
Western blot analysis of IGFBP5. Experiments were carried out by cell fractionation into cytoplasmic (cyto) and nuclear fractions. The image shows IGFBP5 levels at different time points following the incubation of MSCs culture with His-tagged IGFBP5. Three different primary antibodies against IGFBP5 were used (see main text). GAPDH and Histone H4 were used as cytoplasmic and nuclear markers, respectively

The presence of IGFBP5 within nuclei was further validated by subcellular western blot analysis (Fig. 3). The results of this experiment also suggest that only the secreted form of IGFBP5 is capable of reaching the nucleus. In detail, we utilized three different antibodies: an anti-His-tag polyclonal antibody against the His-tag-IGFBP5 recombinant protein, an anti-IGFBP5 monoclonal antibody (clone D6) that targets the internal domain of IGFBP5 and detects both the recombinant and native forms, and an anti-IGFBP5 monoclonal antibody (clone C-18) that targets the C-terminal domain of native IGFBP5 exclusively.

After incubating MSC cultures with the His-tag-IGFBP5 recombinant protein, the anti-His-tag antibody detected the protein within the cytoplasm after 1 min of incubation, and subsequently, the protein gradually accumulated within the nuclei (Fig. 3). The C-18 antibody revealed the presence of native endogenous IGFBP5 solely in the cytoplasm of MSCs throughout the experimental timeframe (Fig. 3). The D-6 antibody, which targets both IGFBP5 isoforms, exhibited a similar pattern as the anti-His-tag antibody. The only distinction was that at time zero, before the addition of external IGFBP5, the antibody identified a cytoplasmic signal, which corresponds to native IGFBP5 (Fig. 3).

### Is IGFBP5 an autocrine or paracrine factor?

The cells directly affected by genotoxic stimuli, which induce senescence, release IGFBP5. This protein can act either as an autocrine factor, reinforcing the senescence process, or as a paracrine factor, promoting senescence in healthy cells, or both.

**Fig. 4:**
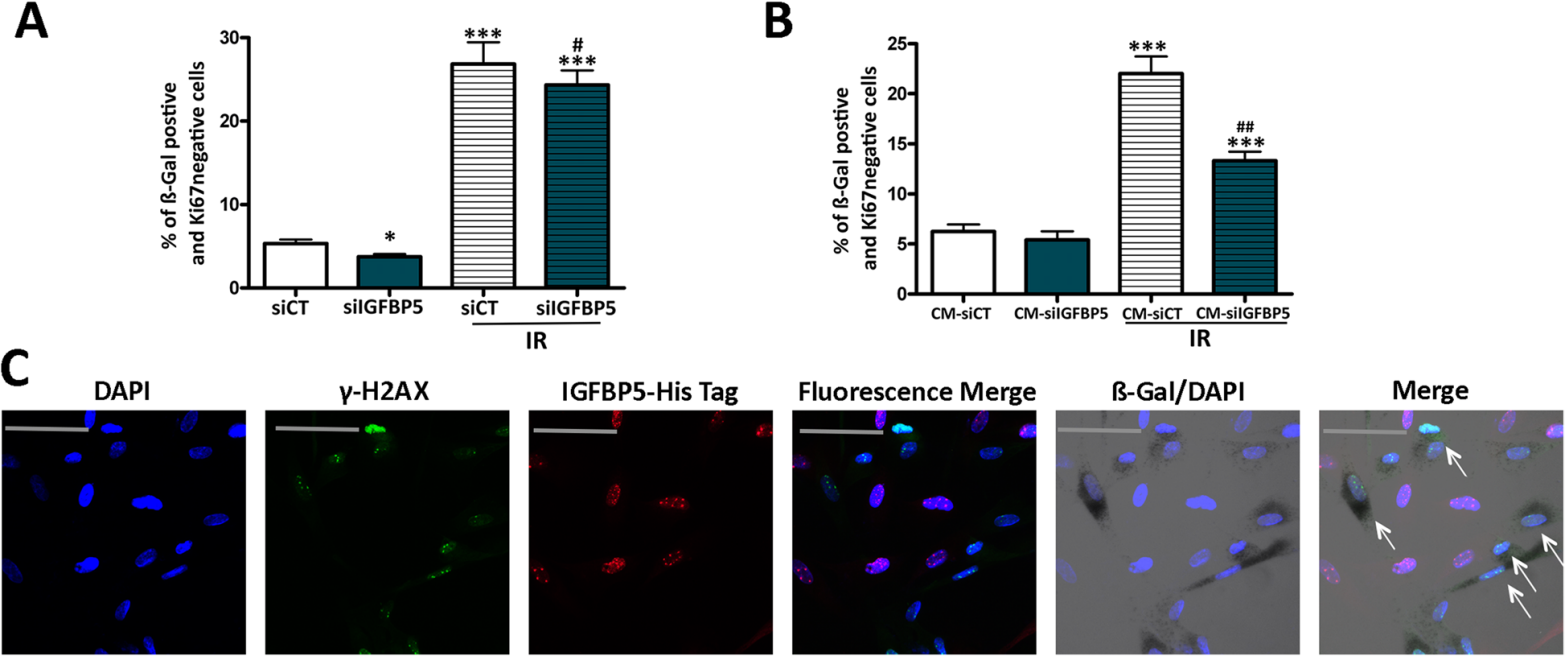
Paracrine action of IGFBP5. **A** MSCs transfected with control siRNA or IGFBP5-siRNA were X-ray irradiated, and senescence was evaluated 48 h later. The graph depicts the percentage of senescent cells under different experimental conditions. The symbols *** *p* < 0.001 and * *p* < 0.05 indicate statistical significance between the control (siCT) and other samples. The # (*p* < 0.05) indicateds statistical significance between irradiated siCT versus irradiated siIGFBP5 (**B**) Healthy MSCs were incubated for 48 h with conditioned media (CM) obtained from the previously mentioned samples. The graph illustrates the percentage of senescent cells under different experimental conditions. The symbol *** *p* < 0.001 indicates statistical significance between the control (siCT) and other samples. The ## (*p* < 0.01) indicateds statistical significance between irradiated siCT versus irradiated siIGFBP5. **C** Representative images of cells stained with anti-γH2AX (green) and His-tag IGFBP5 (red) are shown. Cell nuclei were stained with DAPI. The β-galactosidase activity was evidenced as dark gray. We employed a Leica CTR500 microscope, which was equipped with a DCF3000G digital monochrome camera. The β-galactosidase activity was captured as a gray-stain using this configuration. The arrows show cells that were β-galactosidase/γH2AX positive and IGFBP5 negative

We silenced IGFBP5 with siRNA and subsequently irradiated MSCs to induce senescence (Supplementary file 3D, E). The exogenous stress induced senescence in MSCs with silenced IGFBP5 (Fig. 4A). This indicates that IGFBP5 is mainly involved in secondary senescence through a paracrine mechanism, as already suggested by the experiment reported in Fig. 1A, which shows that blocking IGFBP5 in SASP reduces the senescence process. We further confirmed this hypothesis by incubating healthy MSCs with SASP obtained from irradiated MSCs with silenced IGFBP5 (Fig. 4B). The SASP of cells with silenced IGFBP5 exhibited a reduced capacity to induce secondary senescence.

We then conducted an experiment to further validate the involvement of IGFBP5 in secondary senescence. We incubated irradiated MSC cultures with His-tag-IGFBP5 recombinant protein and monitored the internalization process (Fig. 4C). The nuclear accumulation of IGFBP5 occurred only in cells that were γH2AX (-), which is a marker of cells with damaged DNA [35]. The γH2AX ( +) cells, which were directly damaged by X-rays, did not exhibit significant uptake of IGFBP5. This result indicates that IGFBP5 primarily acts as a paracrine factor.

### IGFBP5 acts through RARα/RXRα

**Fig. 5:**
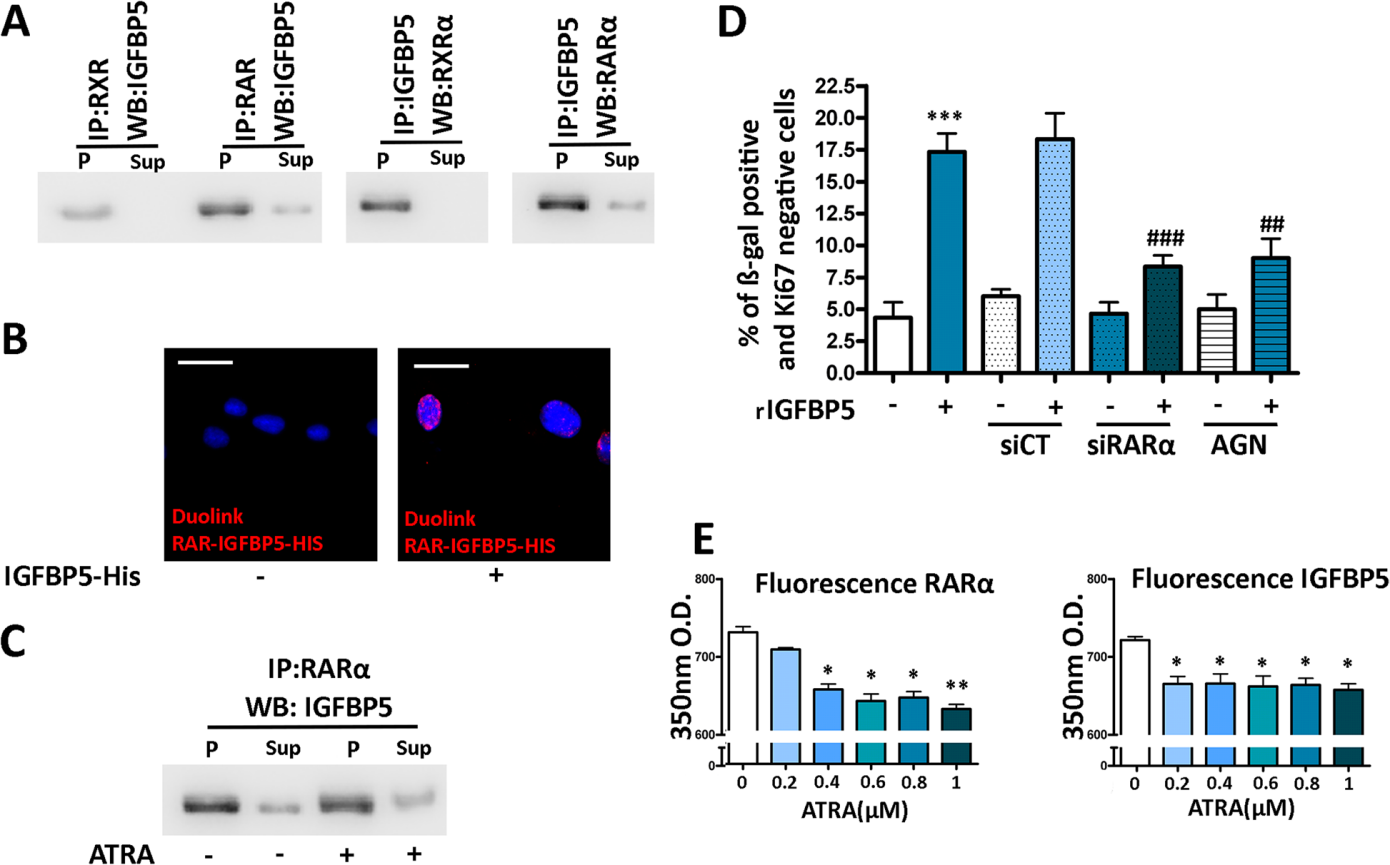
Interaction between IGFBP5 and retinoic acid receptors. **A** Cell lysates were immunoprecipitated with either anti-RXRα or anti-RARα antibodies and then subjected to western blot analysis using anti-IGFBP5 antibody. Reciprocal immunoprecipitation (IP) was performed with anti-IGFBP5 antibody, followed by western blots (WB) using either anti-RXRα or anti-RARα antibodies. P and Sup refer to the pellet and supernatant, respectively, of the immunoprecipitation reaction. **B** Representative images of the Duolink assay to identify the physical proximity between IGFBP5 and RARα. The red staining indicates a close interaction between IGFBP5 and RARα ten minutes after MSCs were incubated with His-tagged IGFBP5. The nuclei were stained with DAPI (blue). The scale bar corresponds to 100 microns. **C** Recombinant RARα, immobilized on protein A beads, was incubated with IGFBP5 in the presence or absence of ATRA, followed by western blot analysis using anti-IGFBP5 antibody. P and Sup refer to the pellet and supernatant, respectively, of the reaction. **D** The graph shows the percentage of senescent cells following incubation with IGFBP5 under different experimental conditions. The symbol *** *p* < 0.001 indicates statistical significance between untreated cells and samples incubated with IGFBP5 (first and second column). The symbols ### *p* < 0.001 and ## *p* < 0.01 indicate the statistically significance between the sample treated with IGFBP5 (second column from left), which was chosen as reference, and the others with IGFBP5 in combination with further treatments. **E** Fluorescence quenching assay. The graphs show the ultraviolet peak emission of IGFBP5 and RARα, due to tryptophan, phenylalanine, and tyrosine, either in the absence or presence of increasing amounts of ATRA, which acted as the quencher. Data are reported with standard deviation. The symbols * *p* < 0.05, ** *p* < 0.01 indicate statistical significance between samples incubated with and without ATRA. The latter condition was chosen as the reference

The presence of IGFBP5 within nuclei suggests a potential role in the regulation of gene expression. Previous studies have shown that IGFBP5 can interact with retinoid receptors known as RARs (retinoic acid receptors) and RXRs (retinoid X receptors), which function as transcription factors in response to specific signaling molecules [36, 37]. This interaction between IGFBP5 and retinoid receptors has been examined in a breast cancer cell line [37]. To further confirm this finding in a normal primary cell line, we incubated MSCs with His-tag-IGFBP5 recombinant protein, isolated cellular protein lysates, and performed immunoprecipitation using an anti-His-tag antibody. The RARα and RXRα proteins co-immunoprecipitated with IGFBP5 Reciprocally, we identified IGFBP5 in the RARα and RXRα immunoprecipitated (Fig. 5A, Supplementary File 3F).

The RXR receptors can function as dimers with themselves or other receptors such as RAR receptors, proliferator-activated receptor (PPAR), vitamin D receptor (VDR), and thyroid hormone receptor (TR) [38]. Analyzing the complex interactions of RXRs can be challenging. Therefore, we adopted a heuristic approach and focused on RARα-related signaling. Considering that IGFBP5 interacts with both RARα and RXRα proteins, we specifically focused on RAR receptors as they exclusively act as heterodimers by interacting with RXRs. The Duolink® Proximity Ligation Assay further validated the close proximity between IGFBP5 and RARα within nuclei (Fig. 5B).

We then examined whether RARα plays a role in IGFBP5-induced senescence. We treated MSCs with IGFBP5 in the presence of siRNA targeting RARα mRNA (Supplementary file 3B) or with AG193109, an effective RARα antagonist [39]. In both cases, the level of senescence was significantly lower compared to cells treated with IGFBP5 alone (Fig. 5D).

### RAR ligands and IGFBP5

All-trans retinoic acid (ATRA) has been proposed as the natural specific ligand for RARα, and it is found in various tissues as well as in serum [40, 41]. The question arises: Does the binding of ATRA with RARα interfere with the IGFBP5-RARα interaction? To investigate this, we conducted an experiment where RARα, immobilized on protein A beads, was incubated with IGFBP5 in the presence or absence of ATRA. The levels of bound IGFBP5 were assessed by western blotting, and we observed no significant modification in IGFBP5 levels under both experimental conditions (Fig. 5C, Supplementary file 3F). This result suggests that ATRA binding to RARα occurs on a protein domain different from the one involved in the interaction between IGFBP5 and RARα.

Another aspect worth considering is whether ATRA interacts with IGFBP5. To preliminary explore this, we employed a fluorescence quenching assay [42]. In this assay, dynamic quenching occurs when a fluorophore, in its excited state, loses its activation upon contact with another molecule, known as the quencher, without any chemical modification occurring in either molecule during the interaction. We employed quenching of protein intrinsic fluorescence due to tryptophan, phenylalanine, and tyrosine to study the binding of ATRA to IGFBP5 (Fig. 5E) [43]. As a reference, we also analyzed quenching following the interaction of ATRA with RARα. The incubation of RARα with ATRA resulted in a quenching of 350 nM emission fluorescence. Additionally, ATRA exhibited quenching of IGFBP5 fluorescence, suggesting a possible interaction between the protein and the ligand (Fig. 5E).

### IGFBP5 and RAR/RXR responsive genes

Upon interaction with ATRA, RAR/RXR heterodimers undergo significant structural alterations and coordinate the transcription of specific sets of genes by binding to distinct DNA response elements known as RA response elements (RAREs). Additionally, they recruit cofactor complexes that modify the nearby chromatin structure and/or facilitate the involvement of the basal transcription machinery [36].

We subsequently aimed to investigate the potential involvement of IGFBP5 in the regulatory mechanism governing retinoic acid-responsive genes. Numerous genes, including those implicated in the senescence common executive program possess RARE elements in their promoter/enhancer regions. We focused our attention on those that are involved in senescence of human MSCs: (RB2 (P130), TP53, CDKN1A (P21) and CDKN1B (P27). Indeed, we demonstrated that RB1-P16 is dispensable for senescence of human MSCs, since silencing of RB1 induced senescence of MSCs [44].

To delve into the details, we employed the JASPAR database to analyze the promoter regions of these genes, aiming to identify potential RARE elements. Our analysis revealed multiple RARE sequences within the promoter regions of the analyzed genes, spanning from -1,000 to + 100 nucleotides (Supplementary file  4).

Subsequently, we treated MSCs with either IGFBP5, ATRA, or both molecules and assessed their impact on the transcription of the aforementioned genes after one hour. This time point was chosen as mRNA transcription regulation is expected to occur early following treatment with factors that act at the transcriptional level.

**Fig. 6:**
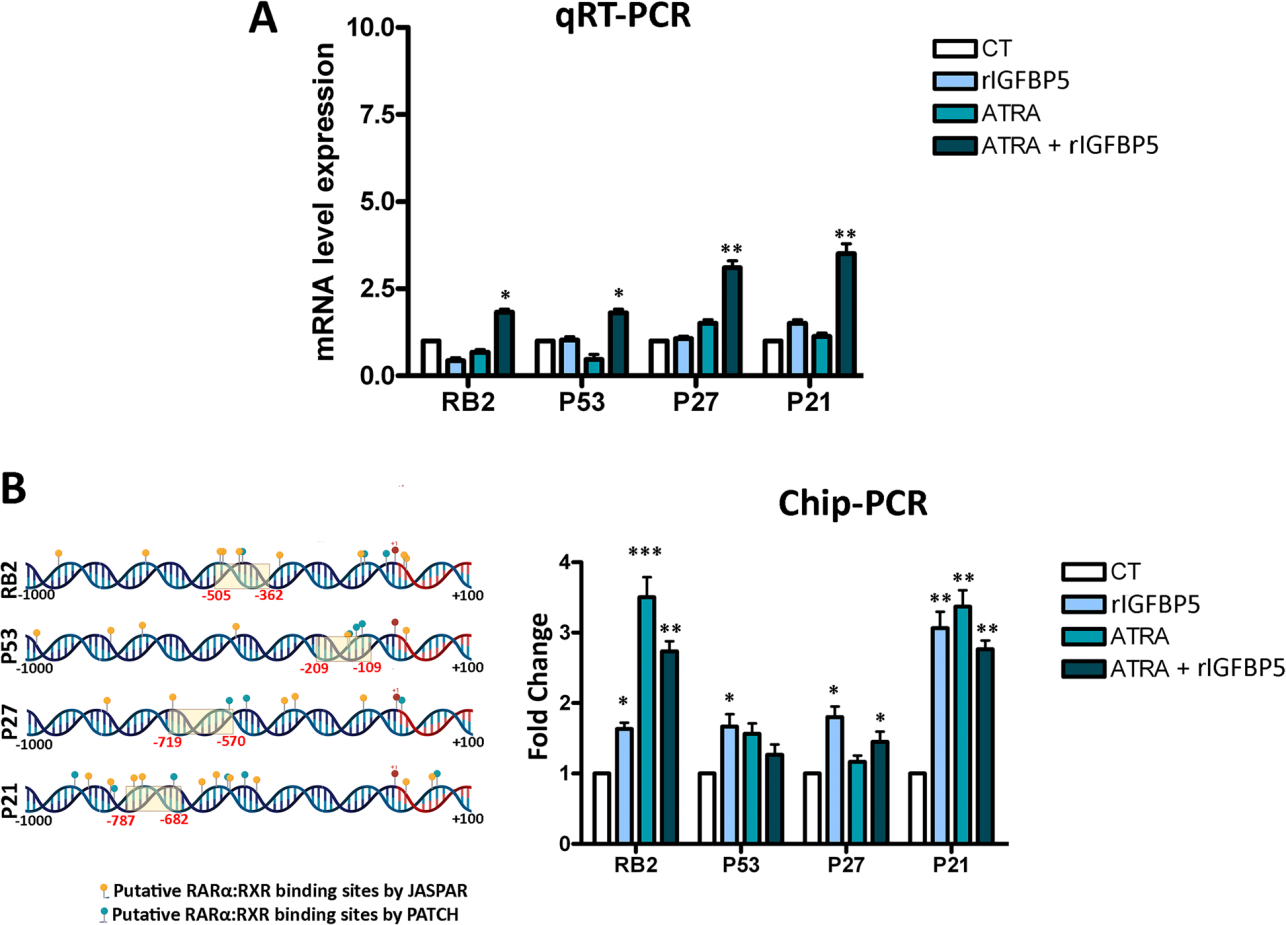
Gene expression and CHIP analysis of senescence master regulators. **A** Histogram showing the mRNA expression levels of the indicated genes under different experimental conditions as detected by qRT-PCR. The mRNA levels were normalized to GAPDH mRNA expression, which served as an internal control. CT: untreated samples; rIGFBP5, ATRA, and ATRA + rIGFBP5 indicate samples incubated for one hour with the indicated molecules. The symbols * p < 0.05, ** *p* < 0.01, *** *p* < 0.001 indicate statistical significance between samples incubated with rIGFBP5 and/or ATRA and the untreated cells (CT), with the latter condition chosen as the reference. **B** The picture shows the putative RARE motifs, identified by JASPAR or PATCH, on the promoters of the indicated genes. For each gene promoter, the analyzed regions span from -1,000 to + 100 nucleotides. The dashed yellow rectangles represent the CHIP-PCR analyzed regions for each promoter. Data were normalized using the input percent method. The histogram displays the fold changes in CHIP values in cells treated with rIGFBP5, ATRA, or both, with respect to control samples (CT) set at a value of 1. The symbols * *p* < 0.05, ** *p* < 0.01, *** *p* < 0.001 indicate statistical significance between samples incubated with rIGFBP5 and/or ATRA and the CT

Although the impact of either IGFBP5 or ATRA on the constituents of this pathway was relatively insignificant, the simultaneous provision of both factors substantially elevated the expression levels of RB2 (P130), TP53, CDKN1A (P21), and CDKN1B (P27), which play a causative role in human MSC senescence [45] (Fig. 6A).

The nuclear presence of IGFBP5 and its interaction with retinoic receptors led us to hypothesize that IGFBP5 may indirectly bind to the RARE element on the promoter of retinoic receptor target genes. Similarly, as we did with mRNA expression analysis, we added IGFBP5 or ATRA or both compounds to MSC culture. One hour later, we performed CHIP-PCR by IGFBP5 immunoprecipitation and subsequent PCR to identify some of the RAREs present in the indicated genes. Interestingly, IGFBP5 was found to be associated with the RAREs of the analyzed genes under different experimental conditions (supplementation of IGFBP5 or ATRA or both), but not in the control experiment (Fig. 6B).

### Could IGBP5 be involved in signaling stress events?

**Fig. 7:**
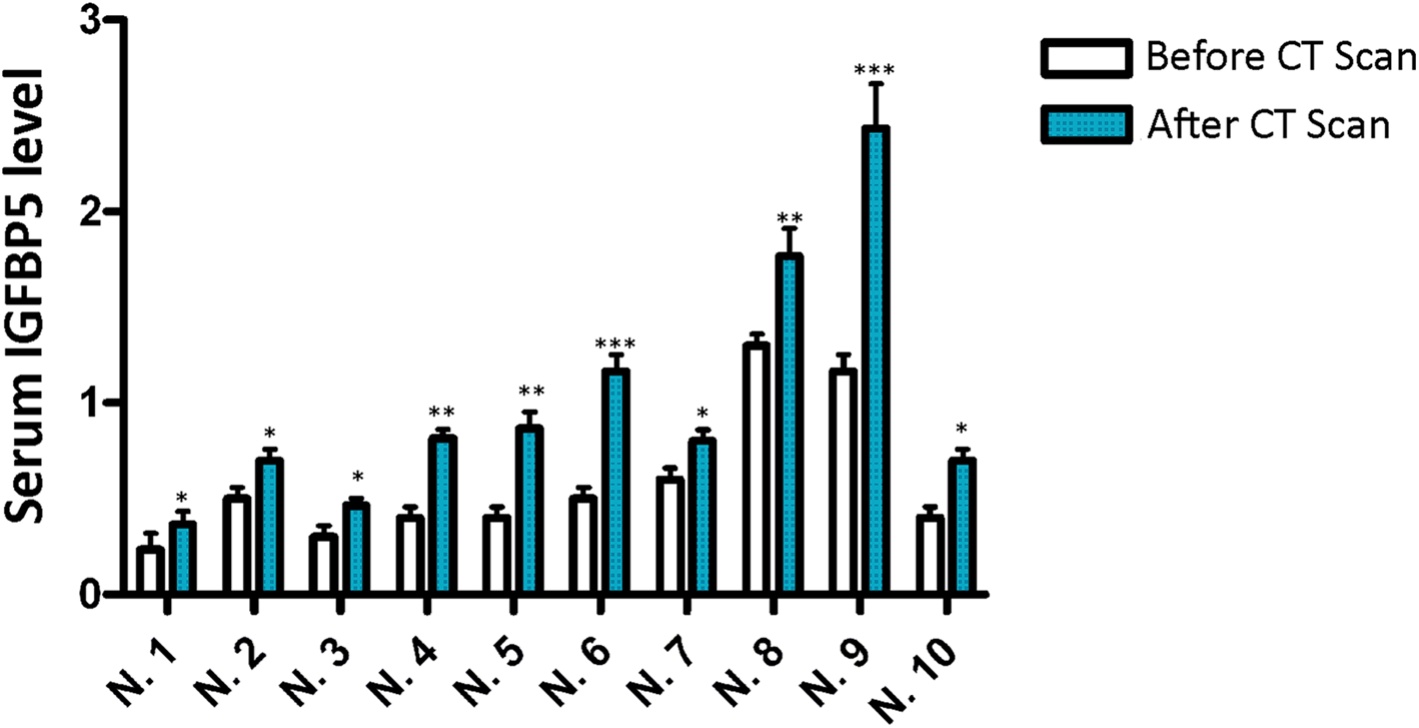
Serum IGFBP5 in patients undergoing medical irradiation. The picture illustrates the ELISA analysis of IGFBP5 in the sera of 10 patients before and 48 h after an abdominal CT scan. Each patient is identified by a number. The data are expressed as arbitrary units (A.U.). For every patient, the significant difference between samples harvested before and after CT is denoted with ** (*p* < 0.01) or * (*p* < 0.05)

Genotoxic damage affecting cells in our body can induce senescence and the release of SASP factors. These factors, when reaching the blood vessels, can promote long-distance effects. It is reasonable to hypothesize that following a stress event, such as radiation exposure, serum levels of IGFBP5 may increase due to senescence of MSCs as well as other cells. Based on this premise, we evaluated changes in the serum levels of IGFBP5 after an abdominal or thorax CT scan. In 10 patients, we observed a significant increase in IGFBP5 levels 48 h after the CT analysis, as detected by ELISA assay (Fig. 7).

## Discussion

The SASP is the primary mechanism through which senescence spreads to neighboring cells, contributing to both organismal aging and cancer development. SASP consists of various factors that regulate multiple functions, such as inducing secondary senescence, modulating immune system activity, remodeling the extracellular matrix, altering tissue structure, and promoting cancer progression. Identifying key factors within SASP is crucial for comprehending the biological underpinnings of senescence and developing effective strategies to counter cellular senescence. Our research findings provide compelling evidence that IGFBP5 acts as a central regulator of secondary senescence, corroborating previous studies that have associated IGFBPs with senescent cells extensively [7, 46]. Notably, previous findings reveal that IGFBPs, when introduced either to cell culture medium or living organisms, can trigger senescence at both the cellular and organismal levels [11, 16, 47].

We have gained detailed insights into the mechanisms by which IGFBP5 operates. This factor is released by primary senescent cells, particularly those damaged by genotoxic agents, following an increase in ROS and prostaglandin release. It is worth noting that this pathway may represent a common mechanism employed by senescent cells to release certain SASP factors. Our previous study demonstrated that PGE2-signaling is involved in IGFBP4 secretion, and other research has indicated that COX2 regulates SASP composition [11, 48].

IGFBP5 induces senescence through an IGF-independent pathway, entering the cell nuclei via caveolae and interacting with retinoic acid receptors. There have been conflicting findings regarding the nuclear localization of IGFBP5. Some studies suggest that IGFBP5 does not enter cell nuclei when added to the culture medium of certain cell types [49], while others indicate that IGFBP5 can enter the nucleus through a nuclear localization sequence-dependent pathway mediated by importin beta nuclear transport factor [24]. Our research aligns with the latter investigation, although the discrepancies observed in previous studies may be attributed to the absence of specific nuclear transporters in certain cell types or biases in cellular trafficking analysis [49].

Through the use of specific antibodies, we have identified interesting aspects of IGFBP5's mode of action. Our results suggest that the secreted form of IGFBP5 enters the nucleus, while the intracellular form localizes in the cytoplasm. Further investigation is required to elucidate the mechanism that allows only the secreted IGFBP5 to enter the nuclei. The caveolae endocytosis pathway could potentially play a role in this phenomenon. It is conceivable that the pleiotropic effects of IGFBP5 are related to its subcellular localization. Notably, nuclear IGFBP5 has been shown to reduce cell proliferation and migration in breast cancer cells, whereas cytoplasmic IGFBP5 increases cell growth and migration [4]. Our observations of reduced proliferation and senescence induction in healthy cultures following IGFBP5 treatment, upon internalization and nuclear localization, align with these findings.

Our research provides evidence that IGFBP5 acts as a paracrine factor rather than an autocrine factor to reinforce senescence. Specifically, when supplemented to irradiated cell MSC cultures, IGFBP5 selectively enters healthy cells rather than senescent cells with damaged DNA. This selectivity may be attributed to dysregulated caveolin-mediated endocytosis observed in senescent cells [50]. Consequently, IGFBP5 promotes secondary senescence in healthy cells but does not exert its effects on already senescent cells.

The proximity of molecules frequently governs the functioning of biochemical processes, playing a crucial role in triggering specific outcomes [51]. While our preliminary studies on the interaction between IGFBP5 and RARα, as well as between IGFBP5 and ATRA, are inconclusive for identifying specific molecular binding and chemical reactions, our findings suggest a potential proximity between IGFBP5, ATRA, and RARα that could have biological implications. Specifically, ATRA and IGFBP5 appear to reinforce the transcription of genes involved in the senescence program, which contain RAREs in their promoters and are potentially responsive to retinoic acid receptor signaling.

Although the effect of either IGFBP5 or ATRA on the key components of senescence circuit was minimal, the simultaneous supplementation of both factors significantly upregulated the expression of P21, P27, P53, and RB2. This increased expression of mRNAs will subsequently result in increased production of the corresponding proteins, which typically occurs during the initial stages of senescence onset. Our data suggest that the cooperation of IGFBP5 and ATRA strengthens the signaling pathway leading to senescence initiation and progression. Additional evidence from chromatin immunoprecipitation data supports this interpretation, as IGFBP5 was found on the RAREs of the aforementioned genes. Although IGFBP5 lacks a RARE binding domain, its presence on RAREs suggests interaction with RARα and/or RXRα.

Retinoic receptors regulate the expression of numerous genes involved in various biological processes, such as proliferation, development, differentiation, apoptosis, senescence, and cancer [36, 52, 53]. The mechanism through which retinoic receptors select a specific subgroup of genes among their targets is not fully understood. However, there are several molecular mediators and factors that can either positively promote the interaction between retinoic receptors and specific targets or hinder the interaction with other factors, thereby selecting a subset of genes on which retinoic receptors will act [36, 52, 53]. In this context, it is conceivable that IGFBP5 may sustain the senescence process by facilitating the interaction of retinoic receptors with genes regulating senescence.

## Conclusions

**Fig. 8:**
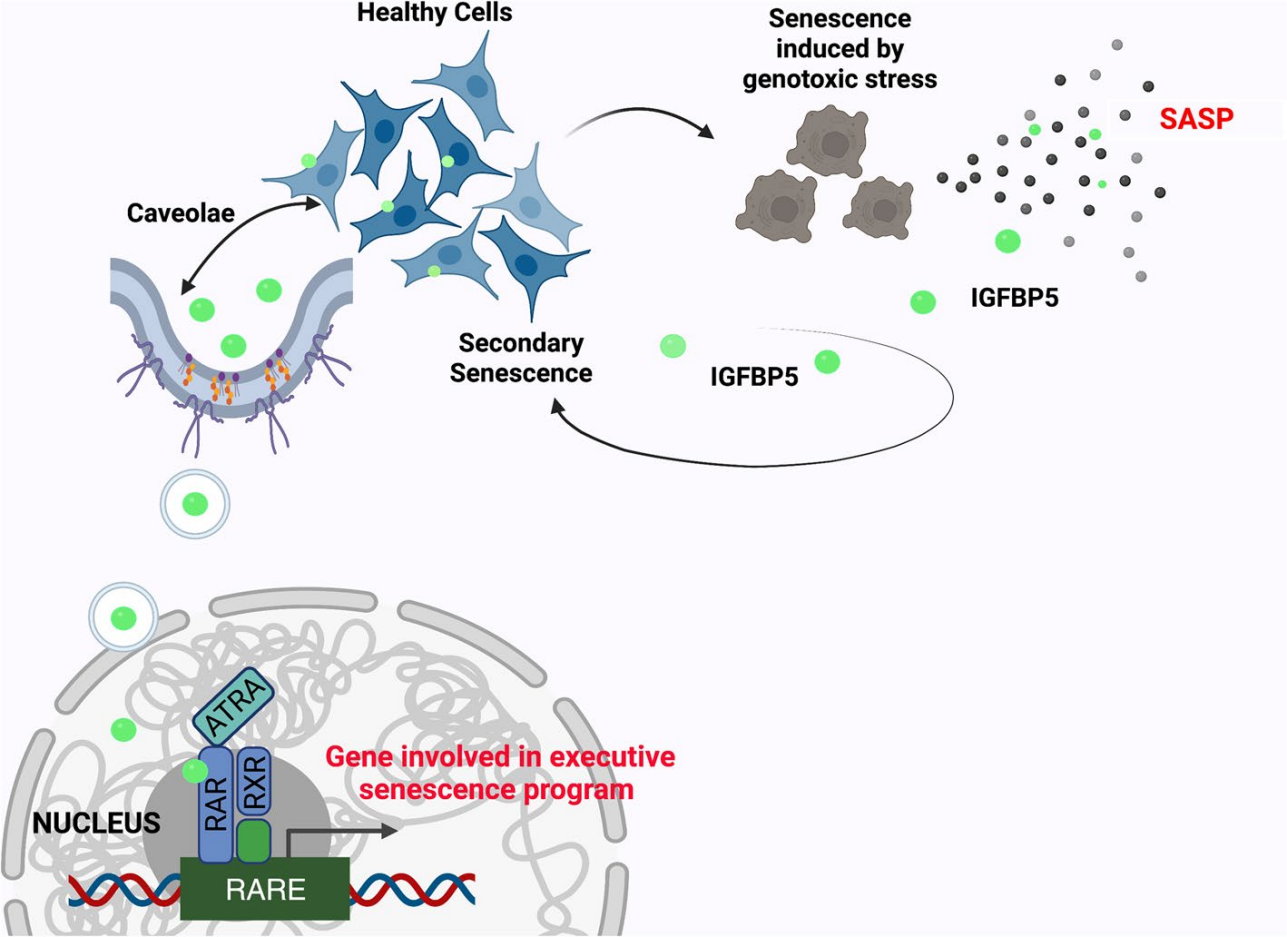
IGFBP5 signaling in senescence. Healthy cells may become senescent cells following genotoxic stress and then release SASP, which contains IGFBP5. The paracrine action of IGFBP5 may induce secondary senescence in healthy cells not directly affected by genotoxic injury. IGFBP5 can enter cell nuclei through caveolae-dependent endocytosis. Within nuclei, IGFBP5 can interact with RAR/RXR heterodimers and contribute to the transcriptional regulation of genes involved in the executive senescence program

Our research demonstrates that IGFBP5 plays a causative role in promoting senescence and can induce senescence in neighboring cells. We have gained insights into the mechanisms underlying IGFBP5's pro-senescence effects, including its release following genotoxic stress, involvement in ROS-prostaglandin signaling, internalization via caveolae, and interaction with RARα. Fig. 8 illustrates a potential signaling pathway for IGFBP5-induced senescence. Further experiments are needed to confirm this hypothesis. These findings enhance our understanding of the complex role of IGFBP5 in senescence and highlight its potential as a therapeutic target for age-related diseases and cancer. Indeed, our investigation into the serum levels of IGFBP5 following exposure to low-dose radiation (LDR) suggests that IGFBP5 could be considered a systemic “alert effector”. Furthermore, it provides evidence that even mild stressful events may lead to the release of harmful health mediators. These findings indicate the need for a dedicated therapeutic strategy to counteract such events. Additionally, this poses an important public health issue since we are commonly exposed to LDR at levels both consciously and accidentally, exceeding natural background levels.

## Materials and Methods

### MSC cultures

Mesenchymal Stromal Cells (MSCs) cultures were obtained from LONZA (Switzerland) at passage 3/4. We plated 1–2.5 × 105 cells/cm2 in alpha-MEM (GIBCO, Italy) supplemented with 10% Embryonic screened Euromed serum (ES) (Euroclone, Italy) and 3 ng/mL βFGF (PeproTech, UK).

### Acute induction of senescence

Exponentially growing cells were irradiated with 2,000 mGy X-rays at room temperature. X-rays were administered by a Mevatron machine (Siemens, Italy) operating at 6 MeV. Following irradiation, cells were further cultivated for the described biological assays.

### In situ senescence-associated beta-galactosidase assay in combination with either Ki67 or γH2AX and His-Tag-IGFBP5 immunodetection

For the beta-galactosidase assay, 20,000 cells per well were seeded in 24-well plates with a glass coverslip. After the treatments, the cells were fixed in a 2% formaldehyde solution for 10 min. Then, cells were washed with PBS 1X (Microgem, Italy) and incubated at 37°C overnight with a staining solution composed of citric acid/phosphate buffer (pH 6), K4Fe(CN)6, K3Fe(CN)6, NaCl, MgCl2, and X-Gal. Next, the cells were permeabilized with 0.3% Triton-X100 (Roche, Switzerland) on ice for 5 min, followed by adding a blocking solution (5% FBS in PBS and 0.1% Triton-X100) for 1 h at room temperature (RT). Subsequently, the cells were incubated with the following primary antibodies: Ki67 (1:200, sc7846, SantaCruz Biotech, TX, USA), or γH2AX (1:600, 9718S, Cell Signaling, MA, USA), and HisTag (1:1000, E-AB-48003, ElabScience, TX, USA) at 4°C overnight in the blocking solution. The TRITC or FITC-conjugated secondary antibody, goat anti-mouse (1:400, Gtx-Mu-003D594), was obtained from ImmunoReagents (Raleigh, NC, USA). Nuclear staining was performed using DAPI mounting medium (ab104139, ABCAM, UK), and micrographs were captured under a fluorescence microscope (Leica DM2000-DMC5400, Leica, Germany).

### Senescent conditioned media (CM) preparation for evaluation of paracrine effects and detection of IGFBP5

After irradiation, cells were cultivated for three days in the growing medium, then to prepare CM, the cultures were extensively washed with PBS 1X and transferred to a chemically defined, serum-free culture medium for an overnight incubation. Subsequently, the CMs were harvested and centrifuged at 2,000 rpm for 30 min to remove cells and debris. CM for paracrine treatment of healthy MSCs was directly added to MSC cultures in a 1:1 ratio with fresh DMEM supplemented with 10% ES. Three days later, senescence was evaluated. The detection of IGFBP5 in CM was performed as follows. For every 1 mL of CM, 10 μL of StrataClean Resin (Agilent Technologies, CA, USA) was added, and the mixture was incubated at 4°C overnight with rotation to precipitate the proteins. Then, the CM was centrifuged at 10,000 g for 10 min, and the pellet was washed twice with TBS 1X and resuspended in 10 μL of Lysis buffer and 10 μL of Laemmli 2X (Sigma-Aldrich, MO, USA). The solution was boiled for 3 min and loaded onto a polyacrylamide gel. Western Blotting was performed using the anti-IGFBP5 antibody (sc-515116, Santa Cruz Biotechnology, TX, USA).

### Effects of oxidative stress on IGFBP5 release

The IGFBP5 release following oxidative stress was evaluated by irradiating MSCs with 2,000 mGy, either in the presence or absence of 1X antioxidant supplement (anti-oxi) (Sigma-Aldrich, MO, USA) or 200 μM Parecoxib (PXB) (Dynastat, Pharmacia Europe, UK) for 24 h at 37°C. Subsequently, the CM was collected and treated as described above.

### Effect of recombinant IGFBP5, IGFII, and inactivation of IGFIIR on MSC senescence

Recombinant human IGFBP5 (rIGFBP5) (35 ng/ml, 100–05) and/or IGFII (25 ng/ml, 100–12) were added to MSC cultures. These recombinant proteins were acquired from PeproTech (UK).

In experiments involving IGFIIR inactivation, cells were treated with 2 μg/ml of an antibody against IGFIIR (sc-25462, Santa Cruz Biotechnology, TX, USA) 30 min before adding the recombinant proteins. After the treatments, senescence-associated beta-galactosidase assay in conjunction with Ki67 immunocytochemistry was performed as described above.

### Evaluation of intake pathway of IGFBP5

To assess the pathways involved in IGFBP5 intake, MSCs were incubated with rIGFBP5-His Tag (PKSH032598, Elabscience TX, USA) at different time points while inhibiting different endocytosis-related pathways. Specifically, the caveolae and Golgi pathways were inhibited by treating the cells with 20 μM Genistein (Sigma-Aldrich, MO, USA) or 10 μM Brefeldin A (Sigma-Aldrich, MO, USA), respectively. The cells were treated with drugs 30 min before adding the recombinant protein.

LRP1 pathway was evaluated using siRNA technology. Specifically, 48 h before adding the recombinant protein, MSCs were incubated with 100 pMoles of siRNA (sc-40101) against LRP1 (siLRP1, Santa Cruz Biotechnology, TX, USA) or a control siRNA (sc-37007) (siCTRL) using Lipofectamine 3000 (Invitrogen, CA, USA) for 6 h. The down-regulation of mRNA targets was assessed using RT-PCR (Supplementary file 3). After treatments, immunocytochemistry or Duolink PLA was carried out as described below.

### Immunocytochemistry

Primary antibodies targeting His-Tag (E-AB-48003), CAV1 (E-AB-70344), LRP1 (E-AB-66274), ITGA2 (E-AB-15930), and GOLGB1 (E-AB-92244) were obtained from Elabscience (TX, USA). The cells were fixed in a 4% Formaldehyde solution for 15 min at room temperature (RT). All antibodies were used according to the manufacturer's instructions. The secondary antibodies FITC or TRITC conjugates were obtained from ImmunoReagents (NC, USA). Nuclear staining was performed using DAPI mounting medium (ab104139, ABCAM, UK), and micrographs were taken under a fluorescence microscope (Leica, Germany).

### Evaluation of IGFBP5 and RARα on senescence

To investigate the effects of IGFBP5 and RARα on senescence, the cells were treated with rIGFBP5 with or without 10 nM All-Trans Retinoic Acid (ATRA) from Sigma Aldrich (MO, USA). We used siRNA (sc-29465) obtained from Santa Cruz Biotechnology (TX, USA) to silence RARα. Additionally, we inhibited RARα receptors with 1 μM AGN 193109 from Sigma Aldrich (MO, USA).

After the treatments, the senescence-associated beta-galactosidase assay in combination with Ki67 immunocytochemistry was performed, as described above.

### Duolink PLA fluorescence

MSCs were cultured on cover slides in a 24 multi-wells plate. The Duolink assay was performed following the manufacturer's instructions (Sigma Aldrich, MO, USA) using His-Tag (Elabscience, TX, USA) and CAV1 (Elabscience, TX, USA), or RARα (E-AB-93398, Elabscience, TX, USA) as primary antibodies. Micrographs were captured under a fluorescence microscope (Leica, Germany).

### Nuclear cytoplasmic cell fractionation

The MSCs were lysed in a buffer containing 0.5% Nonidet P40 and incubated on ice for 5 min. The samples were then centrifuged at 500 g for 5 min. The supernatant, representing the cytoplasmic fraction, was collected and stored for further analysis. The pellet, containing the nuclear fraction, was washed three times with the lysis buffer and then resuspended in the same buffer before further centrifugation at 17,500 g. The resulting pellet represents the nuclear fraction.

### Immunoprecipitation

MSC cells were lysed in a buffer containing 0.1% Triton (Bio-Rad, CA, USA) for 30 min on ice. 20 μg of cell lysate was incubated with 2 μg of either anti-IGFBP5, anti-RXRα, or anti-RARα antibodies each for 12 h at 4°C with gentle rotation. Protein A/G magnetic beads (MedChemExpress, NJ, USA) were washed with PBS 1X and then incubated with the cell lysate plus the antibodies for 4 h at 4°C. The beads were subsequently washed three times with lysis buffer to remove any non-specific binding. The Ag-Ab complex was eluted from the beads by heating or boiling the samples for 5 min in loading buffer with denaturant SDS. The obtained protein samples were then analyzed by western blot.

### RARα/IGFBP5 Binding assay

The RARα/IGFBP5 binding was evaluated using a Pierce™ Co-Immunoprecipitation Kit (ThermoFisher, MA, USA). The anti RARα antibody was immobilized on agarose beads according to the manufacturer's instructions. Next, the RARα recombinant protein (TP761558, Origene, MD, USA), which was diluted in Co-Immunoprecipitation binding buffer, was added to the beads-antibody mixture with rIGFBP5 in the presence or absence of ATRA (10nM, Sigma Aldrich, MO, USA). The reaction mix was incubated for 1 h at 22°C with gentle rotation, and the samples were promptly washed at 4°C. The protein complex was released and collected following centrifugation, and then evaluated by western blot.

### Western blot analysis

The cells were lysed in a buffer containing 0.1% Triton (Bio-Rad, CA, USA) for 30 min on ice. Each lysate (20 μg) was then electrophoresed in a polyacrylamide gel and transferred onto a nitrocellulose membrane. The primary antibodies used were IGFBP5-D6 (cod. sc-515116, Santa Cruz Biotechnology, TX, USA), IGFBP5-C18 (sc6006, Santa Cruz Biotechnology, TX, USA), His-Tag (Elabscience, TX, USA), Histone H4 (2935S, Cell Signaling, MA, USA), GAPDH (Sigma Aldrich, MO, USA), RARα (E-AB-93398, Elabscience, TX, USA), and RXRα (sc515929, Santa Cruz Biotechnology, MO, USA). Immunoreactive signals were detected using a horseradish peroxidase-conjugated secondary antibody (ImmunoReagents, NC, USA) and reacted with ECL plus reagent (Merck Millipore, MA, USA). All antibodies were used according to the manufacturer's instructions.

### Chromatin immunoprecipitation (CHIP) assay

The Simple CHIP Enzymatic Chromatin IP kit from Cell Signaling Technology (MA, USA) was utilized following the manufacturer's instructions. In summary, to crosslink proteins to DNA, cells were treated with 0.068% ethylene glycolbis [succinimidyl succinate] (EGS) for 20 min, followed by 1% formaldehyde in PBS 1X. Next, the samples were treated with 125 mM glycine and incubated in a buffer containing DTT and protease inhibitors. This step was followed by micrococcal nuclease digestion and sonication. After centrifugation, the lysates' supernatants were collected in clean tubes. Anti-IGFBP5 antibody (5 μg) or non-immune IgG (5 μg) was added to supernatants and incubated overnight at 4°C on a rotating device. Following the antibody incubation, protein G agarose beads were added to the samples and further incubated for 2 h at 4°C. The DNA samples were then eluted from the antibody/protein G beads by incubation in a specific elution buffer for 30 min at 65°C and transferred to new tubes. The samples were further treated with proteinase K and purified on spin columns. The obtained immunoprecipitated DNA samples were used for Real-Time PCR (qPCR) amplification as described below.

The putative binding sequences on the promoters of the key genes associated with senescence were acquired from JASPAR (http://jaspar.genereg.net) and Patch 1.0 (http://gene-regulation.com). These databases serve as the leading open-access repositories of matrix profiles, describing the DNA-binding patterns of transcription factors (TFs) and other proteins that interact with DNA in a sequence-specific manner.

For CHIP analysis, primer pairs were designed using the sequence of human promoters and OligoArchitect™ software (Sigma Aldrich, MO, USA). PCR amplification was performed using the BrightGreen 2X qPCR MasterMix (ABM, Canada) and run on a LineGene 9600 (Bioer Technology, PRC). All reagents were used following the manufacturer's instructions. Quantitative IP efficiency was evaluated using the Percent Input Method, as per the following equation: Percent Input = 2% × 2^(CT(2% Input Sample) – CT(IP Sample)).

### RT-PCR and qPCR

Total RNA was extracted from cell cultures using Trizol (Sigma Aldrich, MO, USA). The mRNA levels were determined by RT-PCR, employing the 5X ALL-IN-ONE RT MASTERMIX (cod G486, ABM, Canada). Real-time PCR assays were conducted using BrightGreen 2X qPCR MasterMix (ABM, Canada) and run on a LineGene 9600 instrument (Bioer Technology, PRC). All reagents were used following the manufacturer's instructions.

### Fluorescence quenching assay

Quantitative analysis of the interaction between ATRA and IGFBP5, as well as between ATRA and RARα, was performed using a fluorimetric titration method. Briefly, a solution of IGFBP5 or RARα (1 μM) was titrated in a cuvette by adding successive amounts of ATRA solution, resulting in final concentrations of 0.2, 0.4, 0.6, 0.8, and 1 μM. Fluorescence emission spectra were recorded from 300 to 400 nm with excitation at 295 nm, and fluorescence intensity was measured at an emission wavelength of 350 nm.

### Analysis of IGFBP5 in the sera of patients following CT scan

We recruited a total of 10 male patients, aged between 18 and 65 years, who underwent an abdominal CT scan using the Siemens Somatom Definition Flash instrument. The maximum computer tomography dose index (CTDI) administered was 30 mGy. Inclusion criteria for the study were males within the specified age range, while exclusion criteria encompassed patients with diabetes, obesity, severe cardiovascular diseases, or cancer.

We opted for exclusively male participants to minimize variability, considering previous research indicating that estrogen fluctuations can impact IGF-associated pathways [54]. Additionally, our choice of exclusion criteria aimed at further reducing variability by excluding conditions that could potentially alter IGFBP levels [21, 55, 56].

Before conducting the CT scan, we collected blood samples from each patient. Another set of blood samples was taken 48 h after the CT scan. All patients provided their informed consent after receiving a concise description of the research's objectives.

We analyzed the collected sera using ELISA to determine the levels of IGFBP5 (Human IGFBP-5 DuoSet ELISA—Catalog #: DY875, R&D Systems, Italy) following manufacturer’s instructions.

This research received ethical approval from the Regione Campania Ethical Committee under authorization number 379/C.E. Campania Centro.

### Data analysis involving numbers and patterns

To assess statistical significance, we performed a rigorous examination of the data using various methods, including ANOVA, Student's t-test, and Tukey tests. All the collected data were carefully processed and analyzed using the statistical software package GraphPad Prism version 5.01, developed by GraphPad Software, based in San Diego, CA, USA.

### Supplementary Information

**Additional file 1:** **Supplementary File 1.** Effect of X-ray treatment on MSC biology.

**Additional file 2:** **Supplementary File 2**. Silencing of CAVEOLIN-1 blocks IGFBP5 uptake.

**Additional file 3: Supplementary File 3**. Effectiveness of siRNAs and negative controls of immunoprecipitation experiments**.**

**Additional file 4:** **Supplementary file 4.** JASPAR and PATCH promoter analysis.

**Additional file 5:** **Supplementary file 5.** Release of IGFBP5 in SASP of HDF following peroxide hydrogen stress.

**Additional file 6.**

## Data Availability

The main data supporting the findings of this study are available within the article and as SI Appendix. Unprocessed western blot images and numerical data are available in SI Appendix (Supplementary file 6).

## Abbreviations

ATRA: All-trans retinoic acid
ER: Endoplasmic reticulum
IGFs: Insulin growth factors
MSCs: Mesenchymal stromal cells
PPAR: Proliferator-activated receptor
RARs: Retinoic acid receptors
ROS: Reactive oxygen species
RXRs: Retinoid X receptors
SASP: Senescence-associated secretory phenotype
VDR: Vitamin D receptor
