# Supplementary figures and images for: IGFBP5 is released by senescent cells and is internalized by healthy cells, promoting their senescence through interaction with retinoic receptors
Source: Cell Commun Signal. 2024 Feb 13;22:122. doi: 10.1186/s12964-024-01469-1 (PMC10863175; doi:10.1186/s12964-024-01469-1)

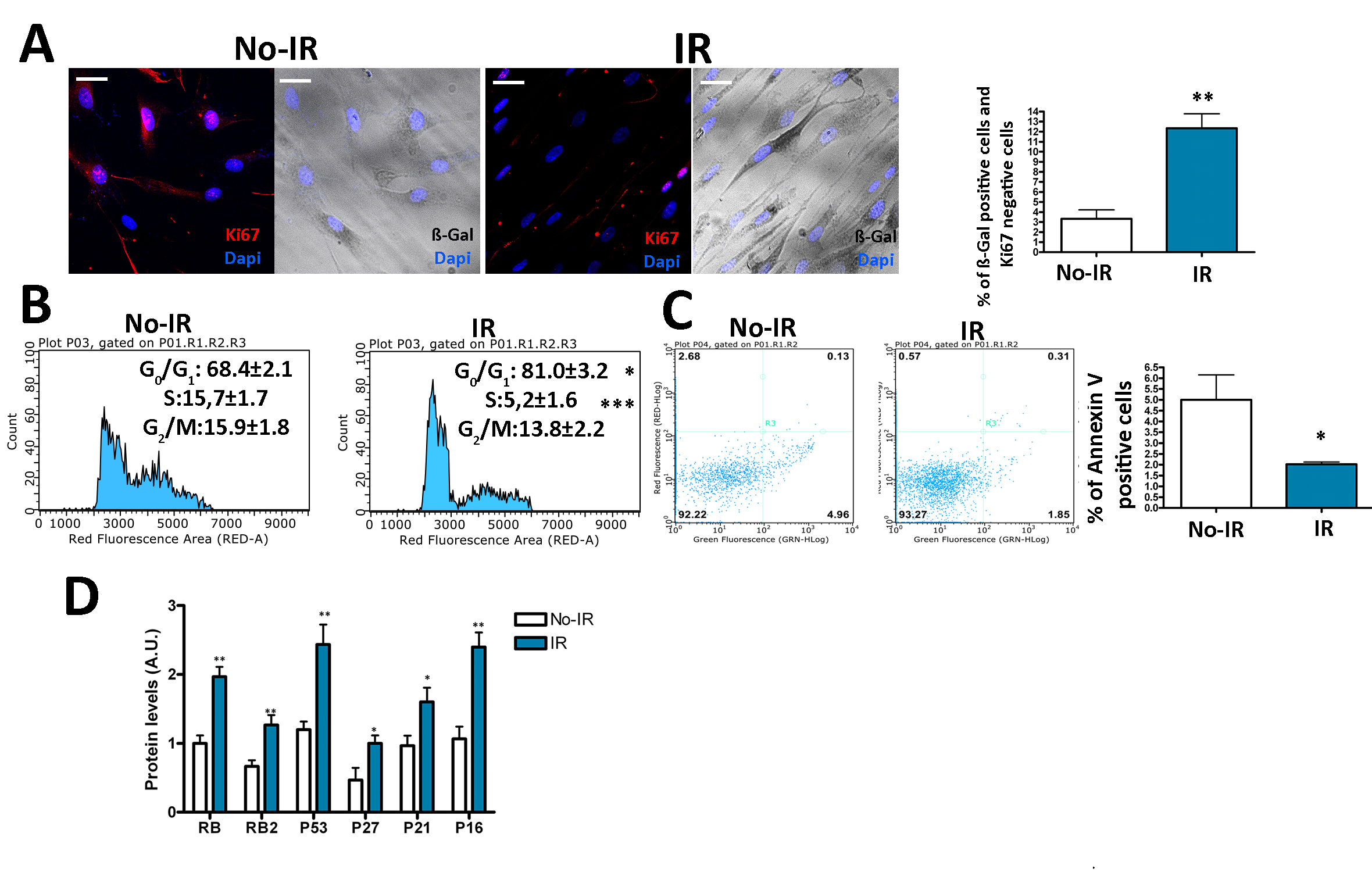

Supplement: Supplementary file 1 — Additional file 1: Supplementary File 1. Effect of X-ray treatment on MSC biology. [file 12964_2024_1469_MOESM1_ESM.tif]

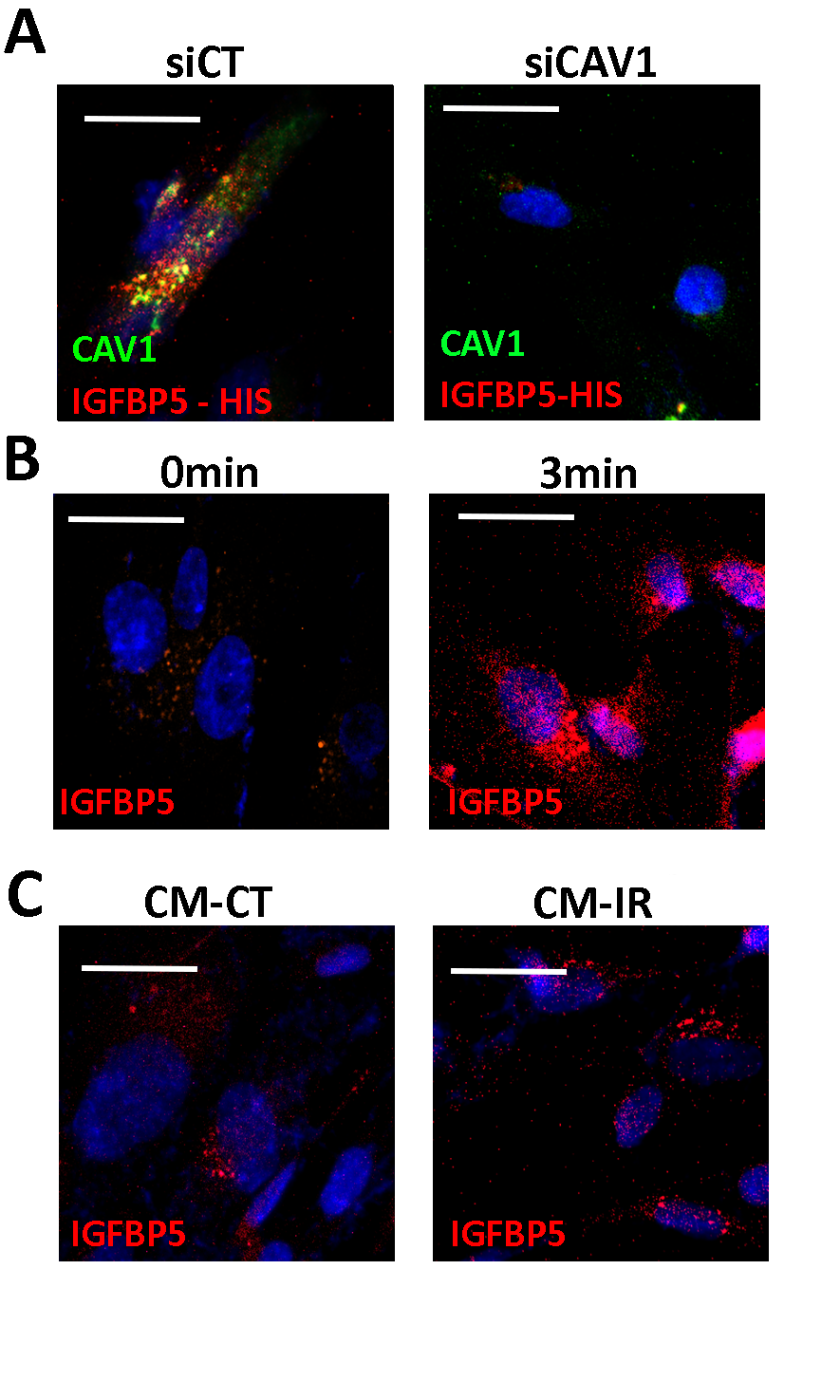

Supplement: Supplementary file 2 — Additional file 2: Supplementary File 2. Silencing of CAVEOLIN-1 blocks IGFBP5 uptake. [file 12964_2024_1469_MOESM2_ESM.tif]

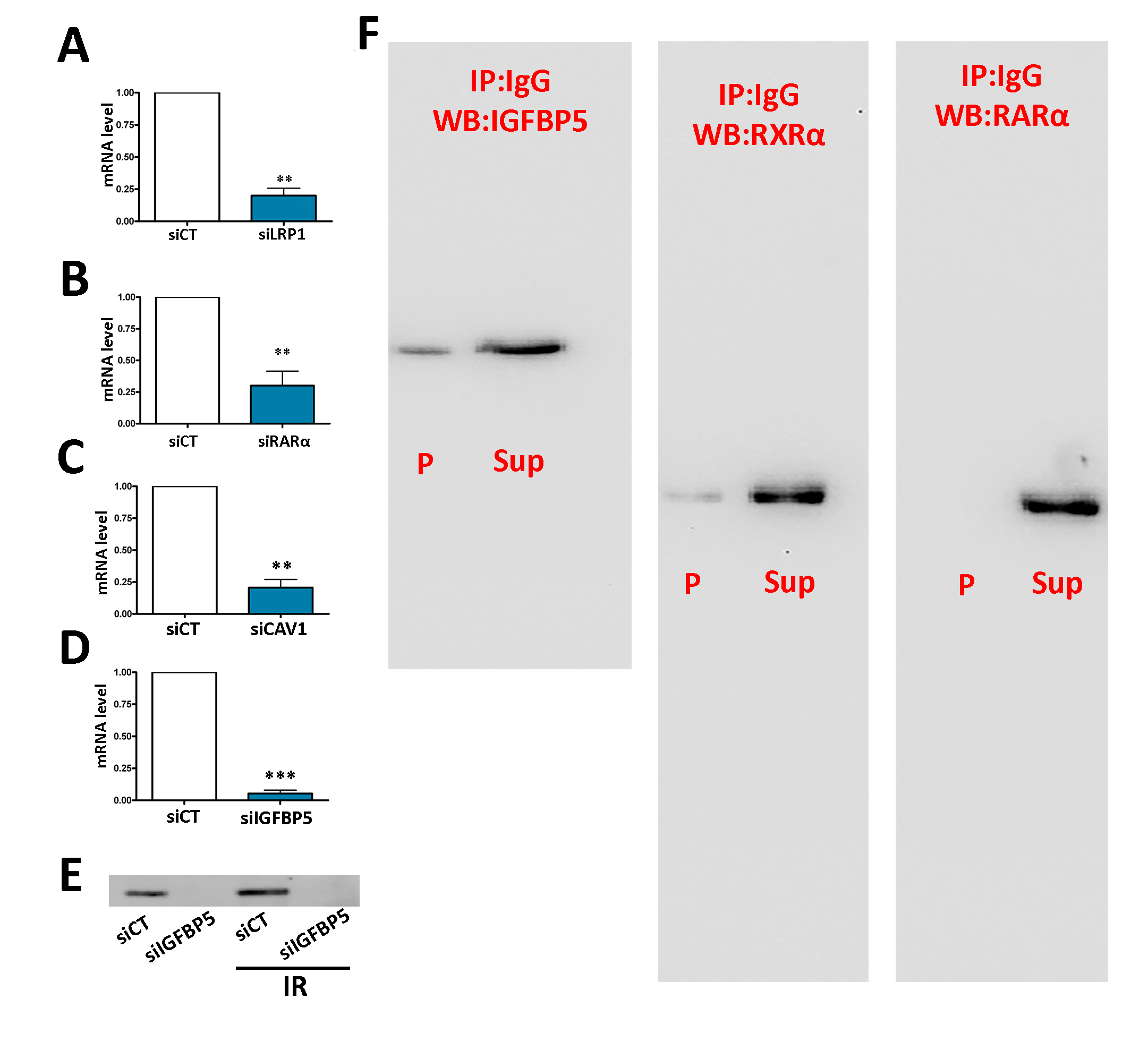

Supplement: Supplementary file 3 — Additional file 3: Supplementary File 3. Effectiveness of siRNAs and negative controls of immunoprecipitation experiments. [file 12964_2024_1469_MOESM3_ESM.tif]

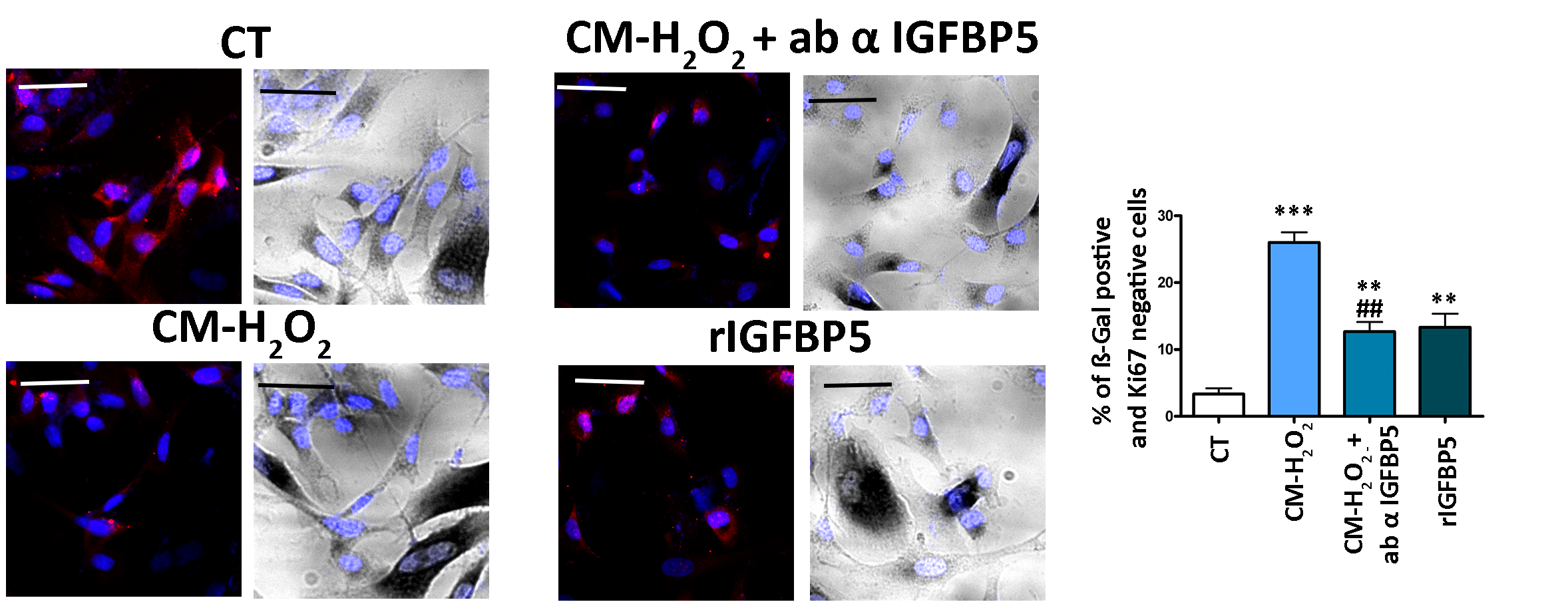

Supplement: Supplementary file 5 — Additional file 5: Supplementary file 5. Release of IGFBP5 in SASP of HDF following peroxide hydrogen stress. [file 12964_2024_1469_MOESM5_ESM.tif]
