# Supplementary material for: IGFBP5 is released by senescent cells and is internalized by healthy cells, promoting their senescence through interaction with retinoic receptors
Source: Cell Commun Signal. 2024 Feb 13;22:122. doi: 10.1186/s12964-024-01469-1 (PMC10863175; doi:10.1186/s12964-024-01469-1)
Supplement: Supplementary file 6 — Additional file 6. [file 12964_2024_1469_MOESM6_ESM.pdf]

# Supplementary File 6

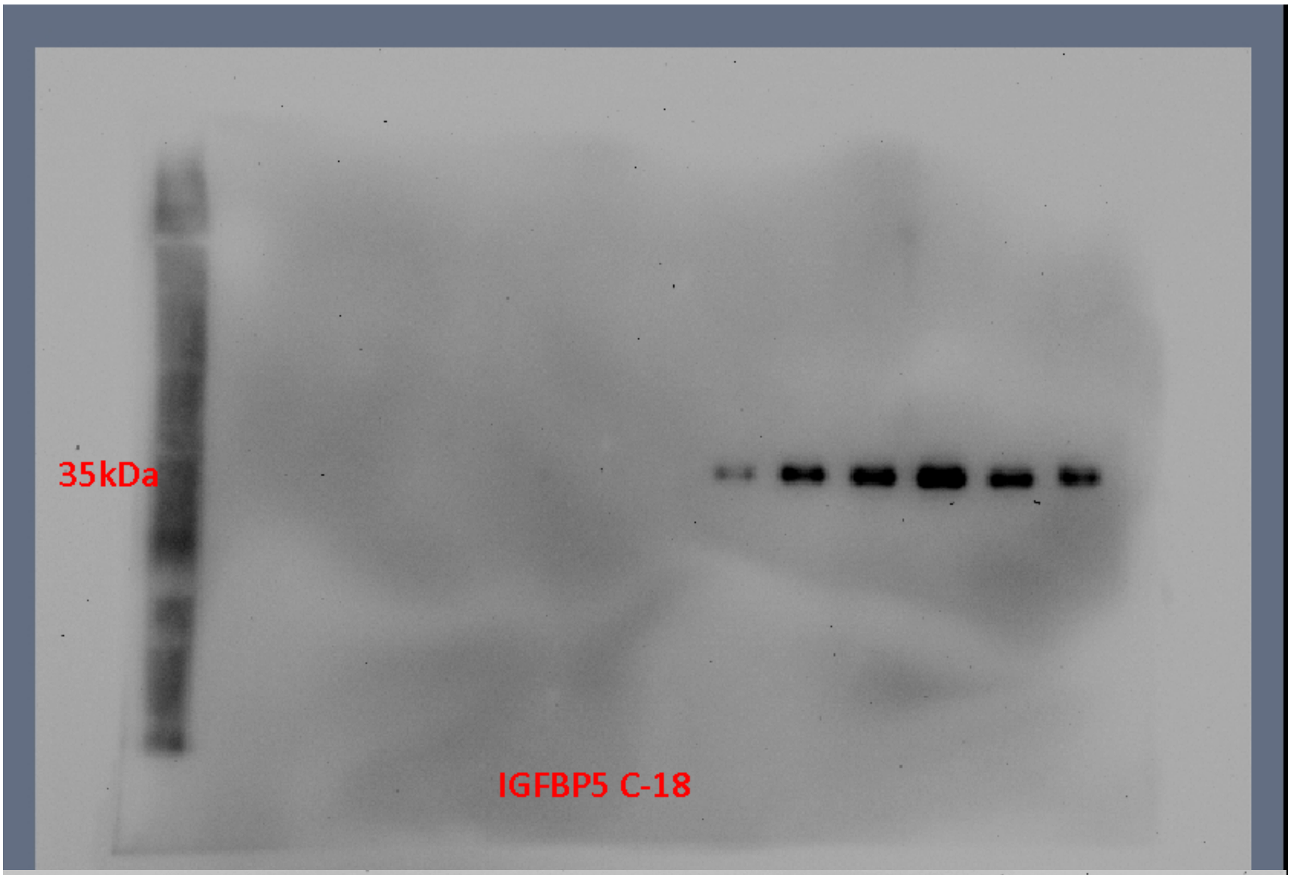
35kDa
IGFBP5 C-18

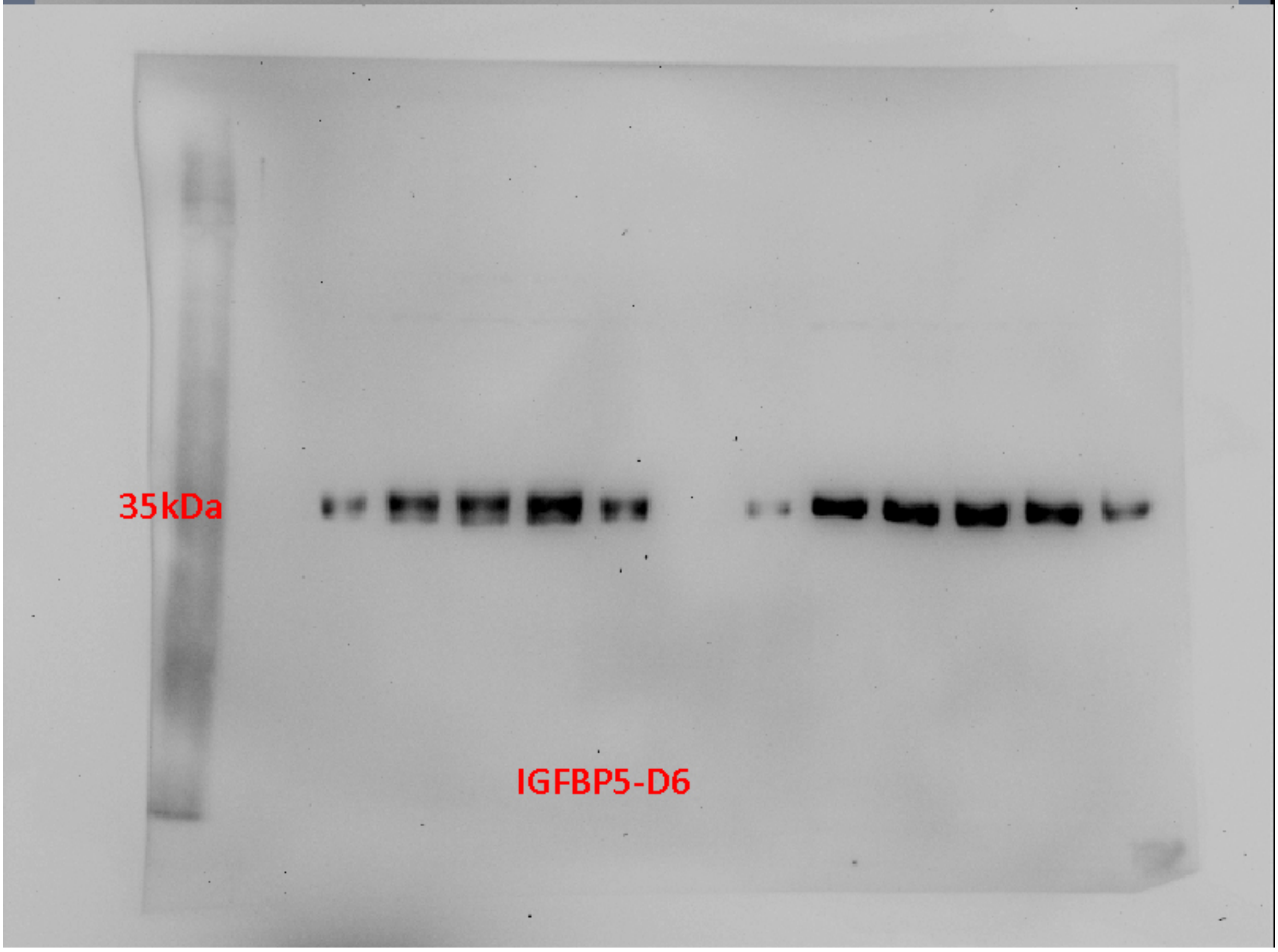
35kDa
IGFBP5-D6

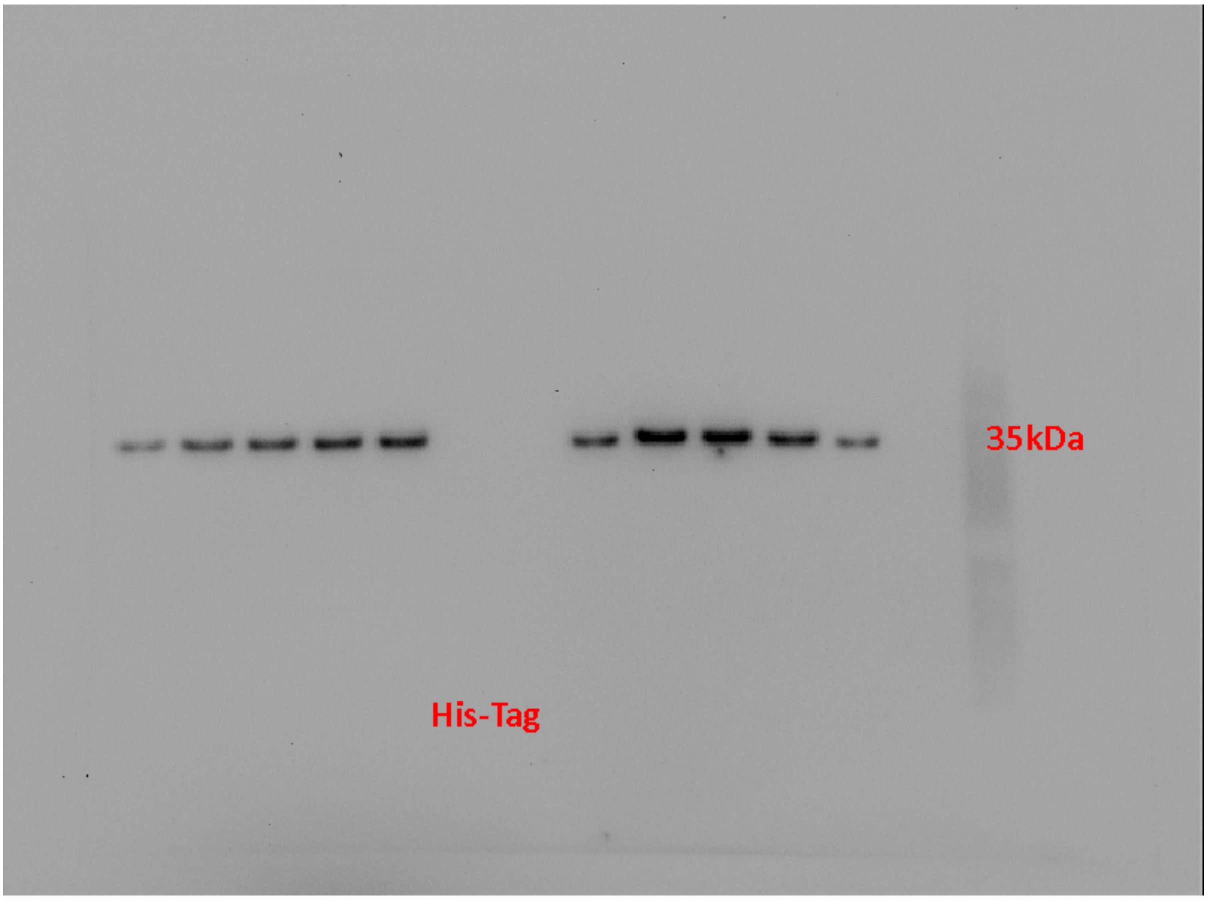
35kDa
His-Tag

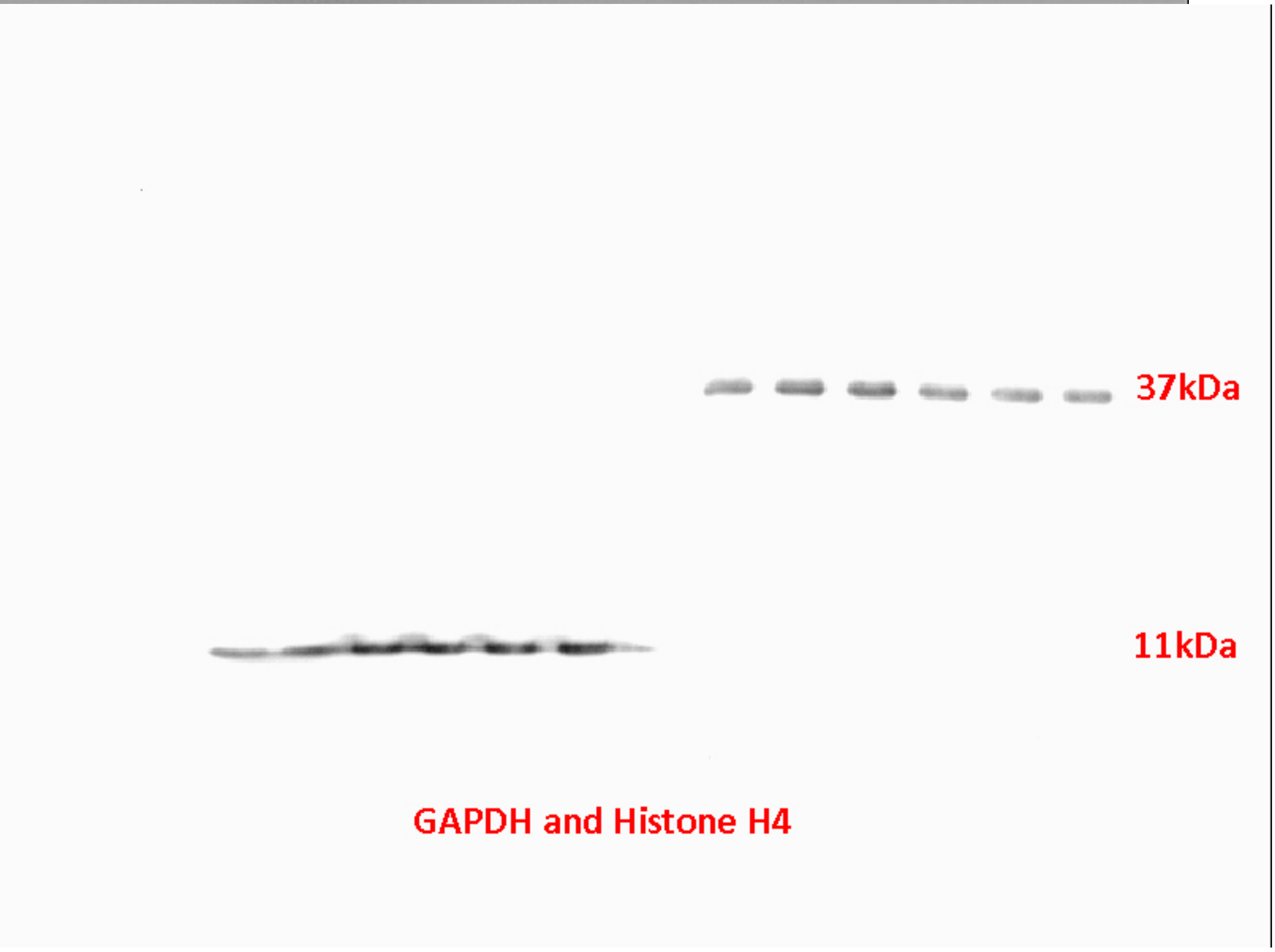
37kDa
11kDa
GAPDH and Histone H4

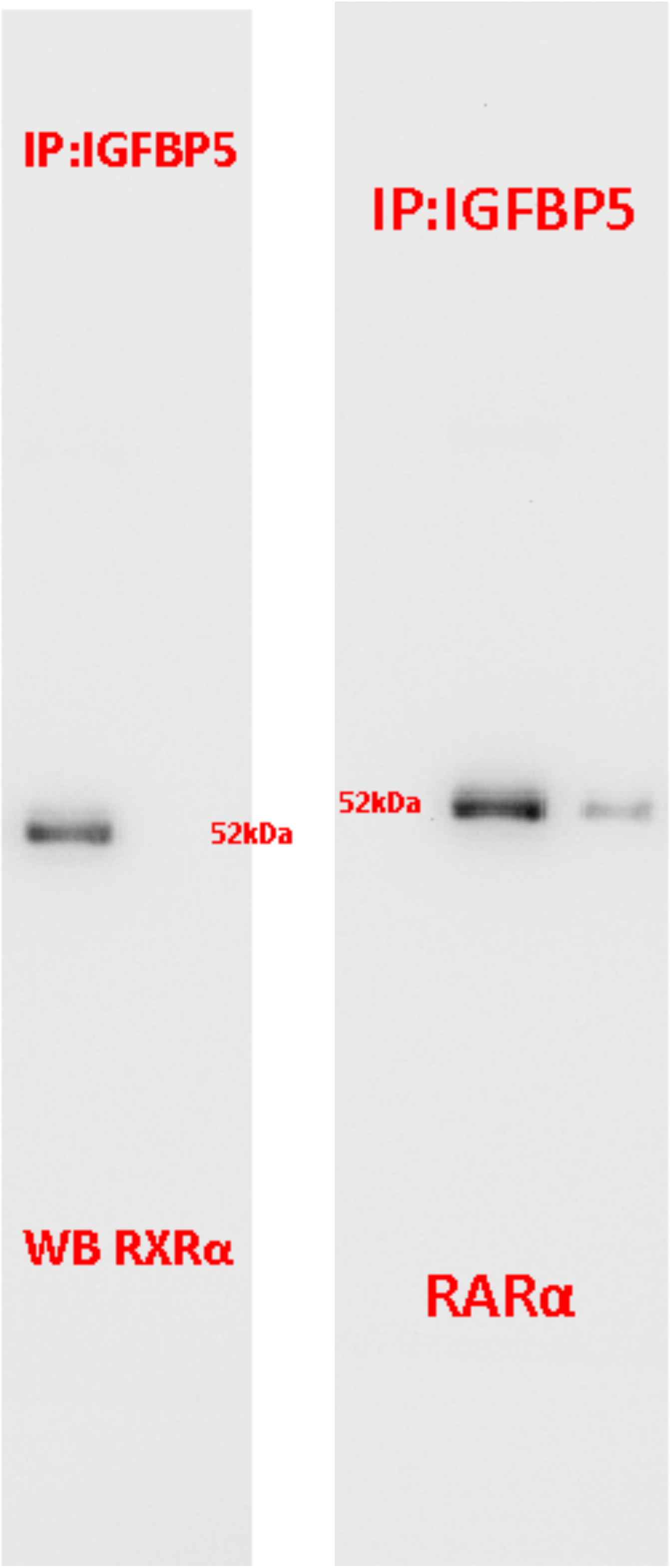
IP:IGFBP5
52kDa
WB RXRα
IP:IGFBP5
52kDa
RARα

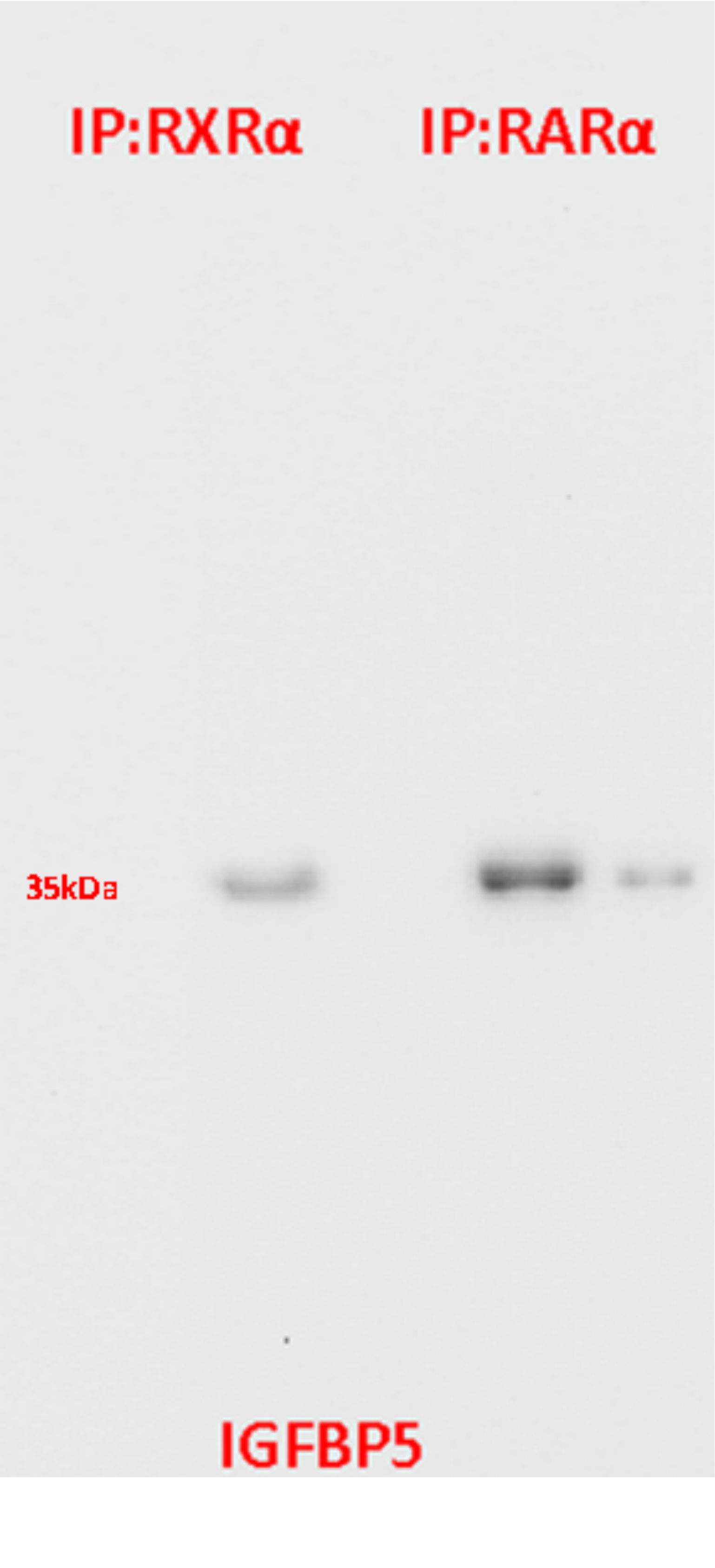
IP:RXRα
IP:RARα
35kDa
IGFBP5
