# Supplementary material for: IGFBP5 is released by senescent cells and is internalized by healthy cells, promoting their senescence through interaction with retinoic receptors
Source: Cell Commun Signal. 2024 Feb 13;22:122. doi: 10.1186/s12964-024-01469-1 (PMC10863175; doi:10.1186/s12964-024-01469-1)
Supplement: Supplementary file 4 — Additional file 4: Supplementary file 4. JASPAR and PATCH promoter analysis. [file 12964_2024_1469_MOESM4_ESM.pdf]

Supplementary File 4

P21 – CDKN1A
JASPAR RARA/RXRA MOTIF

in H. sapiens SEARCH

Access EPDnew
- H. sapiens
- H. sapiens non-coding
- M. mulatta
- M. musculus
- M. musculus non-coding
- R. norvegicus
- C. familiaris
- G. gallus
- D. melanogaster
- A. mellifera
- D. rerio
- C. elegans
- A. thaliana
- Z. mays
- S. cerevisiae
- S. pombe
- P. falciparum
- Select / Download
- Promoter analysis tools
- FTP site

Access EPD
- Promoter elements
- Select / Download
- FTP site

Access MGA Database
- MGA-Search
- MGA Data Overview
- MGA FTP site

Documents

Other Resources

References

What is new

Contact us

Please help us make EPD better! Take our **short survey** to suggest new tools or organism covered. It will take only a few minutes.

General Information

[Help]

Promoter ID:	**CDKN1A_1**
Promoter type:	region
Organism:	Homo sapiens (Human)
Gene Symbol:	CDKN1A
Description of the gene:	cyclin dependent kinase inhibitor 1A
Sequence:	gcccgggcggggcggtgtatatcagggccgcgctgagctgcgccagctGAGGTGTGAGC
Position in the genome:	Chromosome [NC_000006.12]; Strand [+]; Position [36678714]
Ensembl:	ENSG00000124762
RefSeq:	NM_078467
NCBI Gene:	CDKN1A
GeneCards:	CDKN1A

Promoter Image [View it at UCSC]

[Help]

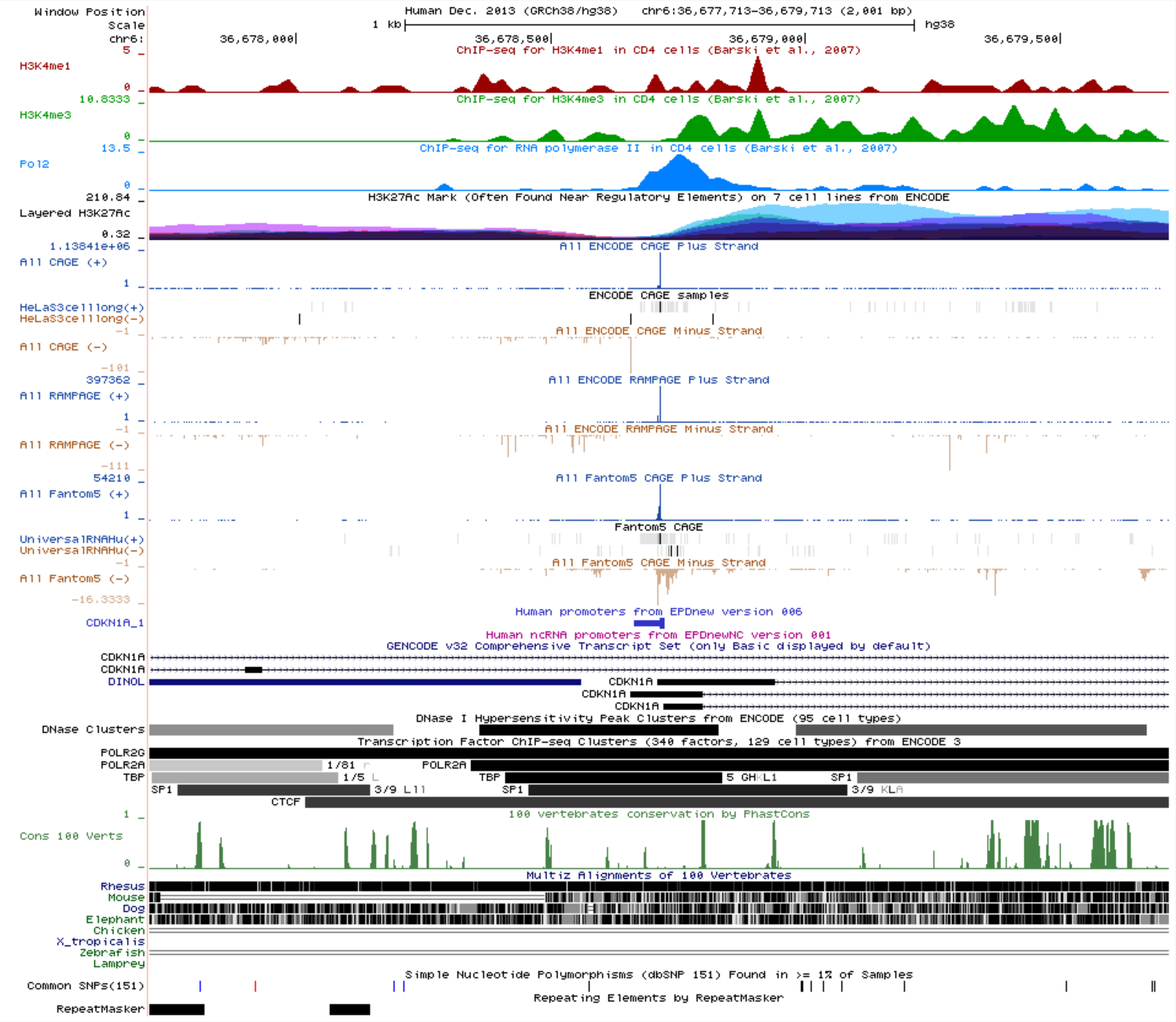

Other viewers

[Help]

EPD view at UCSC (hg38):	chr6:36676713–36680713 (European mirror)
EPD view at UCSC (hg19):	chr6:36644490–36648490 (European mirror)
SwissRegulon (hg19):	chr6:36644491–36648491
FANTOM5 (hg38):	chr6:36676714–36680714
DBTSS/KERO (hg38):	chr6:36676714–36680714
refTSS (hg38):	chr6:36676713–36680713 (European mirror)

Selective viewer showing the 3 Fantom5 tissues with the highest expression of **CDKN1A_1** (European mirror)

GM12878 viewer (European mirror)

Sequence Retrieval Tool

[Help]

Get sequence CDKN1A_1 **from** -1000 **to** 100 bp relative to TSS GET SEQUENCE

☐ lower case upstream TSS

```
>FP009682 CDKN1A_1 :+U EU:NC; range -1000 to 100.
AGAGAGCCCCGGGCAGGAGGCAAAAGTCCTGTGTTCCAACTATAGTCATTTCTTTGCTGC
ATGATCTGAGTTAGGTCACCAGACTTCTCTGAGCCCCAGTTTCCCCAGCAGTGTATACGG
GCTATGTGGGGAGTATTCAGGAGACAGACAACTCACTCGTCAAATCCTCCCCTTCCTGGC
CAACAAAGCTGCTGCAACCACAGGGATTTCTTCTGTTCAGGTGAGTGTAGGGTGTAGGGA
GATTGGTTCAATGTCCAATTCTTCTGTTTCCCTGGAGATCAGGTTGCCCTTTTTTGGTAG
TCTCTCCAATTCCCTCCTTCCCGGAAGCATGTGACAATCAACAACTTTGTATACTTAAGT
TCAGTGGACCTCAATTTCCTCATCTGTGAAATAAACGGGACTGAAAAATCATTCTGGCCT
CAAGATGCTTTGTTGGGGTGTCTAGGTGCTCCAGGTGCTTCTGGGAGAGGTGACCTAGTG
AGGGATCAGTGGGAATAGAGGTGATATTGTGGGGCTTTTCTGGAAATTGCAGAGAGGTGC
ATCGTTTTTATAATTTATGAATTTTTATGTATTAATGTCATCCTCCTGATCTTTTCAGCT
GCATTGGGTAAATCCTTGCCTGCCAGAGTGGGTCAGCGGTGAGCCAGAAAGGGGGCTCAT
TCTAACAGTGCTGTGTCCTCCTGGAGAGTGCCAACTCATTCTCCAAGTAAAAAAAGCCAG
ATTTGTGGCTCACTTCGTGGGGAAATGTGTCCAGCGCACCAACGCAGGCGAGGGACTGGG
GGAGGAGGGAAGTGCCCTCCTGCAGCACGCGAGGTTCCGGGACCGGCTGGCCTGCTGGAA
CTCGGCCAGGCTCAGCTGGCTCGGCGCTGGGCAGCCAGGAGCCTGGGCCCCGGGGAGGGC
GGTCCCGGGCGGCGCGGTGGGCCGAGCGCGGGTCCCGCCTCCTTGAGGCGGGCCCGGGCG
GGGCGGTTGTATATCAGGGCCGCGCTGAGCTGCGCCAGCTGAGGTGTGAGCAGCTGCCGA
AGTCAGTTCCTTGTGGAGCCGGAGCTGGGCGCGGATTCGCCGAGGCACCGAGGCACTCAG
AGGAGGTGAGAGAGCGGCGGC
```

Search Motif Tool

[Help]

Library: Transcription Factor Motifs (JASPAR) Motif: RARA::RXRA

From: -1000 To: 100 bp relative to TSS and a cut-off (p-value) of: 0.001 SEARCH

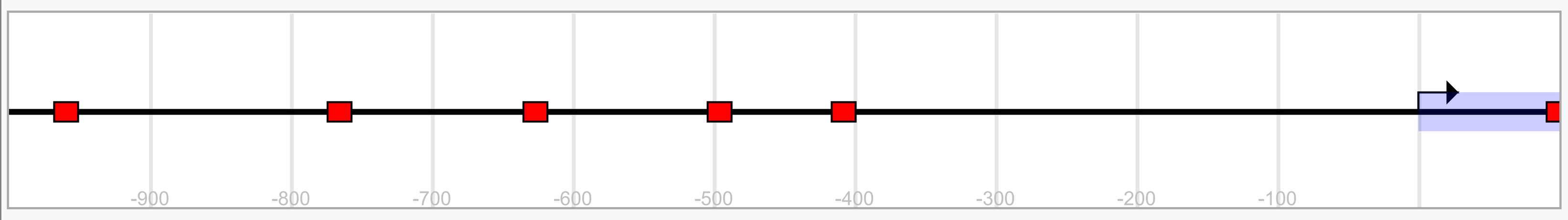

RARA::RXRA [p-value = 0.001]: -968, -774, -635, -505, -417, 91

Expression Profile Tool

[Help]

CDKN1A_1 is expressed in **1038 samples** with an **average expression of 2191.64 tags** per 10M.

The TSSs distribution around the EPDnew annotated start site (click on the histogram bars to explore samples):

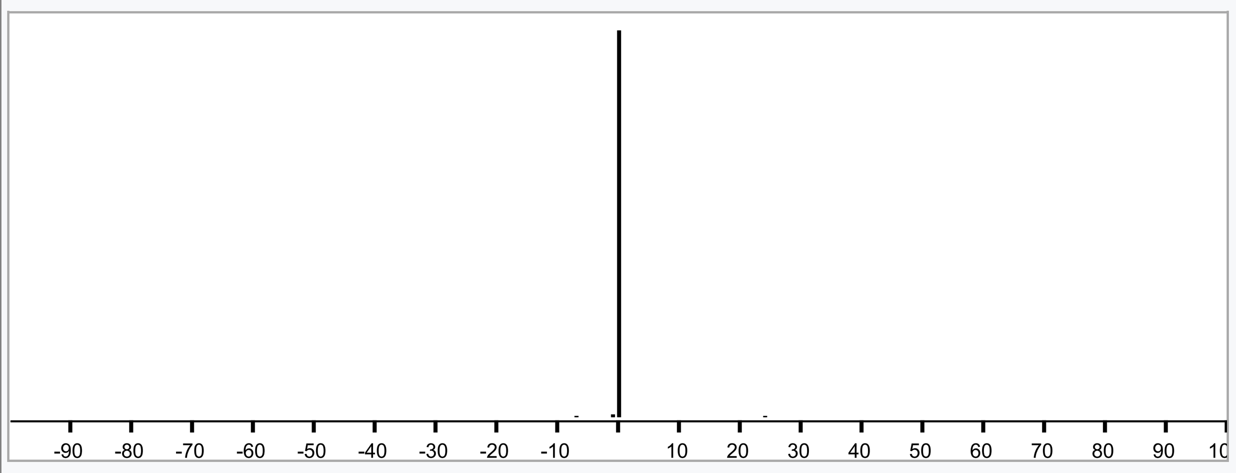

P21 – CDKN1A
JASPAR RARA/RXRG MOTIF

Access EPDnew
- *H. sapiens*
- *H. sapiens non-coding*
- *M. mulatta*
- *M. musculus*
- *M. musculus non-coding*
- *R. norvegicus*
- *C. familiaris*
- *G. gallus*
- *D. melanogaster*
- *A. mellifera*
- *D. rerio*
- *C. elegans*
- *A. thaliana*
- *Z. mays*
- *S. cerevisiae*
- *S. pombe*
- *P. falciparum*
- Select / Download
- Promoter analysis tools
- FTP site

Access EPD
- Promoter elements
- Select / Download
- FTP site

Access MGA Database
- MGA-Search
- MGA Data Overview
- MGA FTP site

Documents
Other Resources
References
What is new
Contact us

in H. sapiens SEARCH

Please help us make EPD better! Take our short survey to suggest new tools or organism covered. It will take only a few minutes.

General Information [Help]

Promoter ID:	CDKN1A_1
Promoter type:	region
Organism:	Homo sapiens (Human)
Gene Symbol:	CDKN1A
Description of the gene:	cyclin dependent kinase inhibitor 1A
Sequence:	gcccgggcggggcggttgtatatcagggccgcgctgagctgcgccagctGAGGTGTGAGC
Position in the genome:	Chromosome [NC_000006.12]; Strand [+]; Position [36678714]
Ensembl:	ENSG00000124762
RefSeq:	NM_078467
NCBI Gene:	CDKN1A
GeneCards:	CDKN1A

Promoter Image [View it at UCSC] [Help]

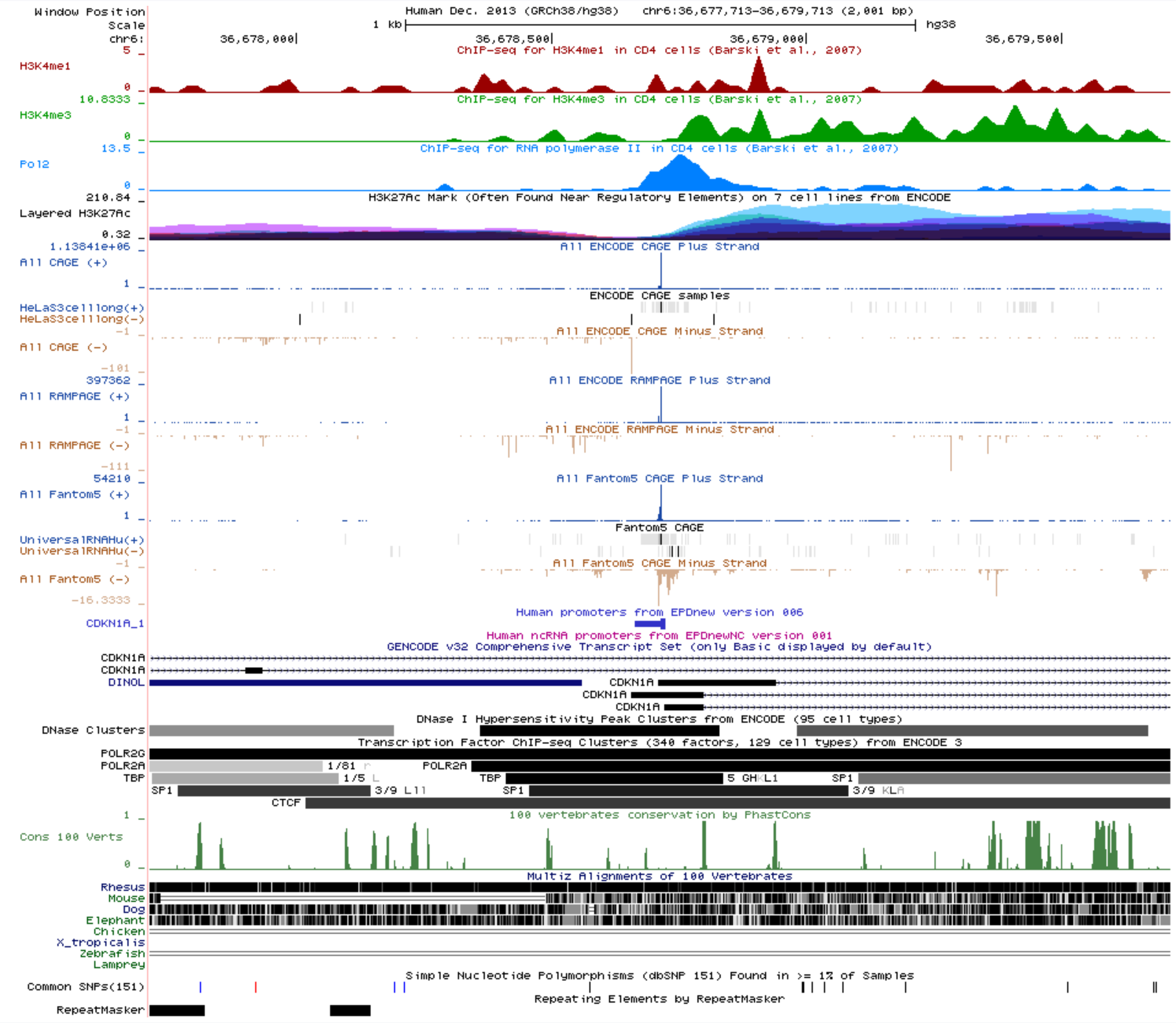

Other viewers [Help]

EPD view at UCSC (hg38):	chr6:36676713–36680713 (European mirror)
EPD view at UCSC (hg19):	chr6:36644490–36648490 (European mirror)
SwissRegulon (hg19):	chr6:36644491–36648491
FANTOM5 (hg38):	chr6:36676714–36680714
DBTSS/KERO (hg38):	chr6:36676714–36680714
refTSS (hg38):	chr6:36676713–36680713 (European mirror)

Selective viewer showing the 3 Fantom5 tissues with the highest expression of **CDKN1A_1** (European mirror)
GM12878 viewer (European mirror)

Sequence Retrieval Tool [Help]

Get sequence CDKN1A_1 **from** -1000 **to** 100 bp relative to TSS GET SEQUENCE
☐ lower case upstream TSS

```
>FP009682 CDKN1A_1 :+U EU:NC; range -1000 to 100.
AGAGAGCCCCGGGCAGGAGGCAAAAGTCCTGTGTTCCAACTATAGTCATTTCTTTGCTGC
ATGATCTGAGTTAGGTCACCAGACTTCTCTGAGCCCCAGTTTCCCCAGCAGTGTATACGG
GCTATGTGGGGAGTATTCAGGAGACAGACAACTCACTCGTCAAATCCTCCCCTTCCTGGC
CAACAAAGCTGCTGCAACCACAGGGATTTCTTCTGTTCAGGTGAGTGTAGGGTGTAGGGA
GATTGGTTCAATGTCCAATTCTTCTGTTTCCCTGGAGATCAGGTTGCCCTTTTTTGGTAG
TCTCTCCAATTCCCTCCTTCCCGGAAGCATGTGACAATCAACAACTTTGTATACTTAAGT
TCAGTGGACCTCAATTTCCTCATCTGTGAAATAAACGGGACTGAAAAATCATTCTGGCCT
CAAGATGCTTTGTTGGGGTGTCTAGGTGCTCCAGGTGCTTCTGGGAGAGGTGACCTAGTG
AGGGATCAGTGGGAATAGAGGTGATATTGTGGGGCTTTTCTGGAAATTGCAGAGAGGTGC
ATCGTTTTTATAATTTATGAATTTTTATGTATTAATGTCATCCTCCTGATCTTTTCAGCT
GCATTGGGTAAATCCTTGCCTGCCAGAGTGGGTCAGCGGTGAGCCAGAAAGGGGGCTCAT
TCTAACAGTGCTGTGTCCTCCTGGAGAGTGCCAACTCATTCTCCAAGTAAAAAAAGCCAG
ATTTGTGGCTCACTTCGTGGGGAAATGTGTCCAGCGCACCAACGCAGGCGAGGGACTGGG
GGAGGAGGGAAGTGCCCTCCTGCAGCACGCGAGGTTCCGGGACCGGCTGGCCTGCTGGAA
CTCGGCCAGGCTCAGCTGGCTCGGCGCTGGGCAGCCAGGAGCCTGGGCCCCGGGGAGGGC
GGTCCCGGGCGGCGCGGTGGGCCGAGCGCGGGTCCCGCCTCCTTGAGGCGGGCCCGGGCG
GGGCGGTTGTATATCAGGGCCGCGCTGAGCTGCGCCAGCTGAGGTGTGAGCAGCTGCCGA
AGTCAGTTCCTTGTGGAGCCGGAGCTGGGCGCGGATTCGCCGAGGCACCGAGGCACTCAG
AGGAGGTGAGAGAGCGGCGGC
```

Search Motif Tool [Help]

Library: Transcription Factor Motifs (JASPAR Motif: RARA::RXRG
From: -1000 To: 100 bp relative to TSS and a cut-off (p-value) of: 0.001 SEARCH

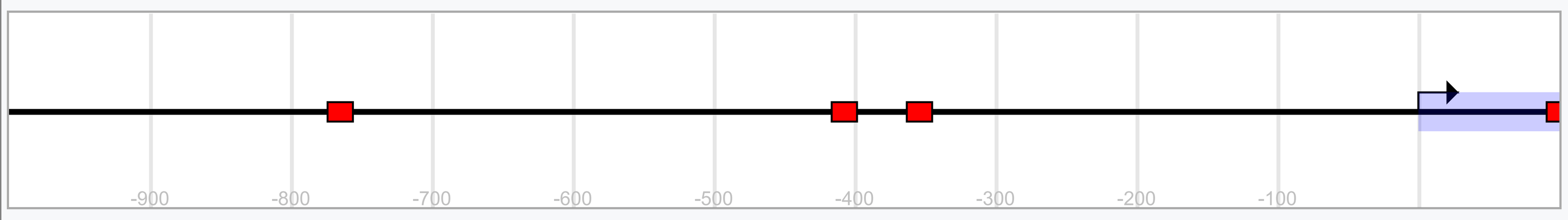

RARA::RXRG [p-value = 0.001]: -774, -417, -363, 91

Expression Profile Tool [Help]

CDKN1A_1 is expressed in **1038 samples** with an **average expression of 2191.64 tags** per 10M.

The TSSs distribution around the EPDnew annotated start site (click on the histogram bars to explore samples):

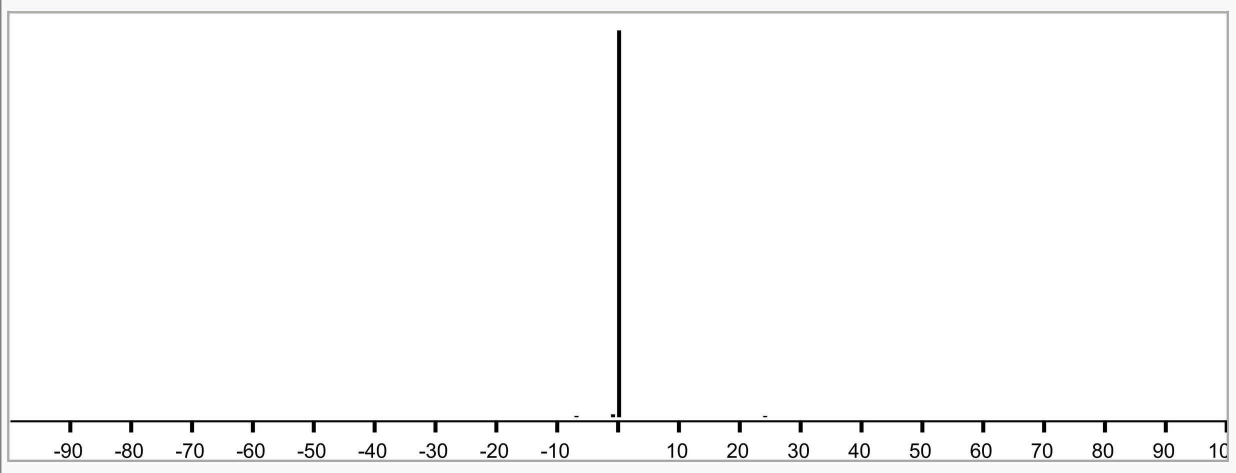

P21 – CDKN1A
JASPAR RARA MOTIF

in H. sapiens SEARCH

Access EPDnew
- H. sapiens
- H. sapiens non-coding
- M. mulatta
- M. musculus
- M. musculus non-coding
- R. norvegicus
- C. familiaris
- G. gallus
- D. melanogaster
- A. mellifera
- D. rerio
- C. elegans
- A. thaliana
- Z. mays
- S. cerevisiae
- S. pombe
- P. falciparum
- Select / Download
- Promoter analysis tools
- FTP site

Access EPD
- Promoter elements
- Select / Download
- FTP site

Access MGA Database
- MGA-Search
- MGA Data Overview
- MGA FTP site

Documents
Other Resources
References
What is new
Contact us

Please help us make EPD better! Take our **short survey** to suggest new tools or organism covered. It will take only a few minutes.

General Information [Help]

Promoter ID:	**CDKN1A_1**
Promoter type:	region
Organism:	Homo sapiens (Human)
Gene Symbol:	CDKN1A
Description of the gene:	cyclin dependent kinase inhibitor 1A
Sequence:	gcccgggcgggggcggtgtatatcagggccgcgctgagctgcgccagctGAGGTGTGAGC
Position in the genome:	Chromosome [NC_000006.12]; Strand [+]; Position [36678714]
Ensembl:	ENSG00000124762
RefSeq:	NM_078467
NCBI Gene:	CDKN1A
GeneCards:	CDKN1A

Promoter Image [View it at UCSC] [Help]

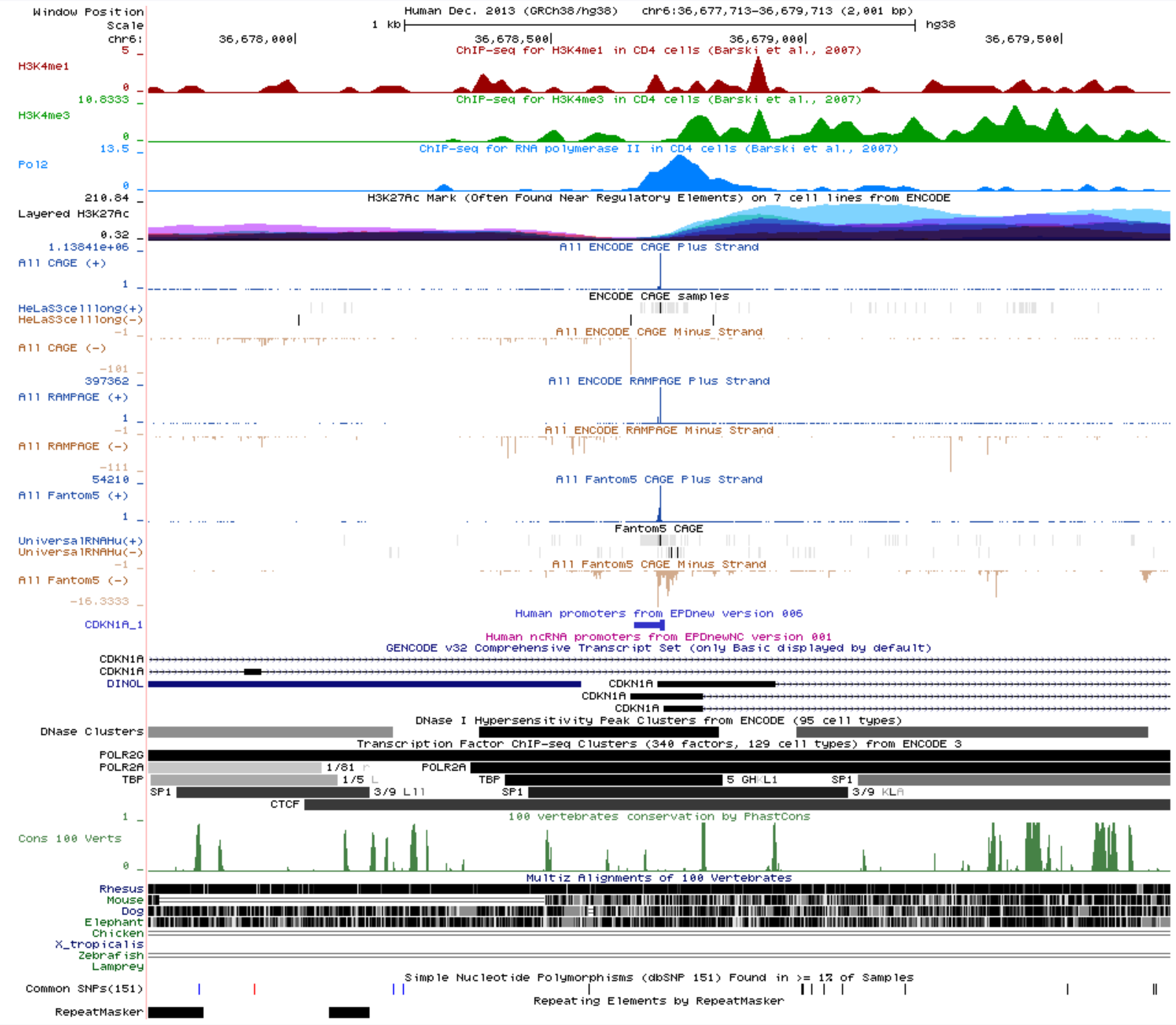

Other viewers [Help]

EPD view at UCSC (hg38):	chr6:36676713–36680713 (European mirror)
EPD view at UCSC (hg19):	chr6:36644490–36648490 (European mirror)
SwissRegulon (hg19):	chr6:36644491–36648491
FANTOM5 (hg38):	chr6:36676714–36680714
DBTSS/KERO (hg38):	chr6:36676714–36680714
refTSS (hg38):	chr6:36676713–36680713 (European mirror)

Selective viewer showing the 3 Fantom5 tissues with the highest expression of **CDKN1A_1** (European mirror)
GM12878 viewer (European mirror)

Sequence Retrieval Tool [Help]

Get sequence CDKN1A_1 **from** -1000 **to** 100 bp relative to TSS GET SEQUENCE
☐ lower case upstream TSS

```
>FP009682 CDKN1A_1 :+U EU:NC; range -1000 to 100.
AGAGAGCCCCGGGCAGGAGGCAAAAGTCCTGTGTTCCAACTATAGTCATTTCTTTGCTGC
ATGATCTGAGTTAGGTCACCAGACTTCTCTGAGCCCCAGTTTCCCCAGCAGTGTATACGG
GCTATGTGGGGAGTATTCAGGAGACAGACAACTCACTCGTCAAATCCTCCCCTTCCTGGC
CAACAAAGCTGCTGCAACCACAGGGATTTCTTCTGTTCAGGTGAGTGTAGGGTGTAGGGA
GATTGGTTCAATGTCCAATTCTTCTGTTTCCCTGGAGATCAGGTTGCCCTTTTTTGGTAG
TCTCTCCAATTCCCTCCTTCCCGGAAGCATGTGACAATCAACAACTTTGTATACTTAAGT
TCAGTGGACCTCAATTTCCTCATCTGTGAAATAAACGGGACTGAAAAATCATTCTGGCCT
CAAGATGCTTTGTTGGGGTGTCTAGGTGCTCCAGGTGCTTCTGGGAGAGGTGACCTAGTG
AGGGATCAGTGGGAATAGAGGTGATATTGTGGGGCTTTTCTGGAAATTGCAGAGAGGTGC
ATCGTTTTTATAATTTATGAATTTTTATGTATTAATGTCATCCTCCTGATCTTTTCAGCT
GCATTGGGTAAATCCTTGCCTGCCAGAGTGGGTCAGCGGTGAGCCAGAAAGGGGGCTCAT
TCTAACAGTGCTGTGTCCTCCTGGAGAGTGCCAACTCATTCTCCAAGTAAAAAAAGCCAG
ATTTGTGGCTCACTTCGTGGGAAATGTGTCCAGCGCACCAACGCAGGCGAGGGACTGGG
GGAGGAGGGAAGTGCCCTCCTGCAGCACGCGAGGTTCCGGGACCGGCTGGCCTGCTGGAA
CTCGGCCAGGCTCAGCTGGCTCGGCGCTGGGCAGCCAGGAGCCTGGGCCCCGGGGAGGGC
GGTCCCGGGCGGCGCGGTGGGCCGAGCGCGGGTCCCGCCTCCTTGAGGCGGGCCCGGGCG
GGGCGGTTGTATATCAGGGCCGCGCTGAGCTGCGCCAGCTGAGGTGTGAGCAGCTGCCGA
AGTCAGTTCCTTGTGGAGCCGGAGCTGGGCGCGGATTCGCCGAGGCACCGAGGCACTCAG
AGGAGGTGAGAGAGCGGCGGC
```

Search Motif Tool [Help]

Library: Transcription Factor Motifs (JASPAR Motif: RARA
From: -1000 To: 100 bp relative to TSS and a cut-off (p-value) of: 0.001 SEARCH

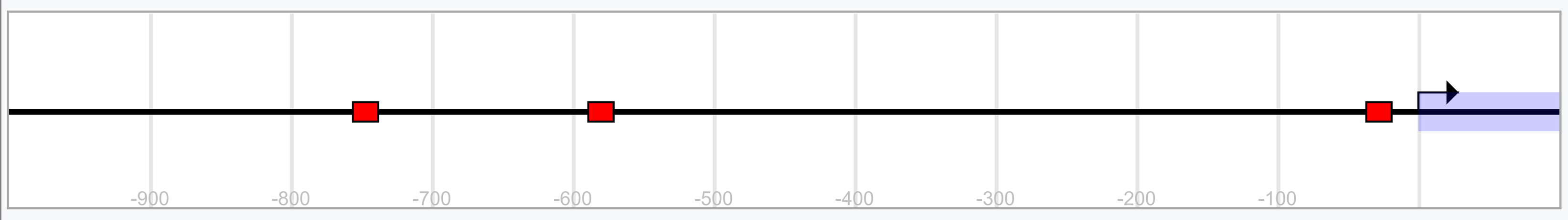

RARA [p-value = 0.001]: -756, -589, -37

Expression Profile Tool [Help]

CDKN1A_1 is expressed in **1038 samples** with an **average expression of 2191.64 tags** per 10M.

The TSSs distribution around the EPDnew annotated start site (click on the histogram bars to explore samples):

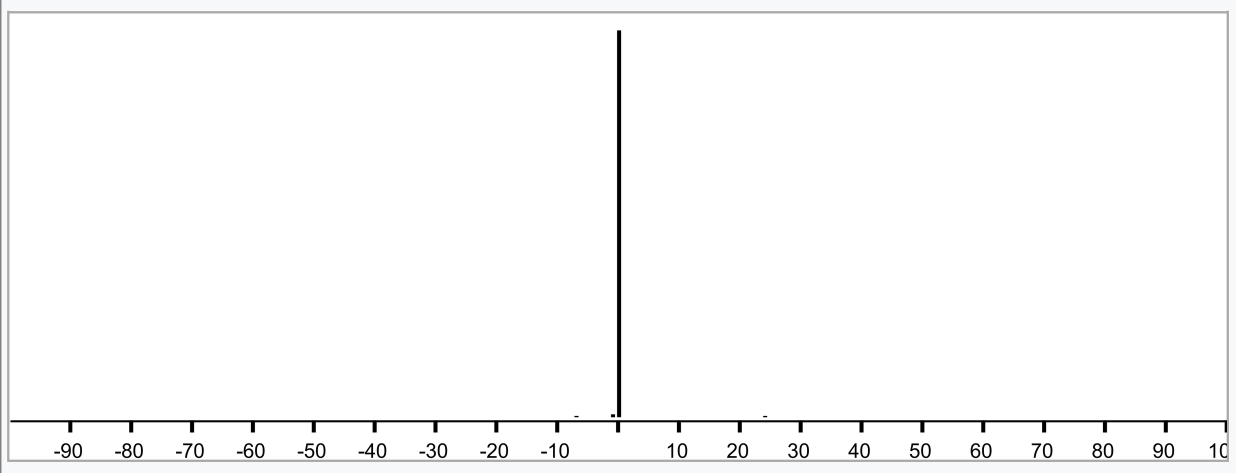

Last update October 2019

P21
PATCH RARE MOTIFS

Thank you for using Patch at Host - It's Fri, 16.4.2021 - 12:44 MEZ

Start a new search

Name of your search:	default
Name of your sequence:	default.seq
Sites searched for:	all
Maximum number of mismatches:	2
Mismatch penalty:	100
Lower score boundary:	87.5

Scanning sequence: FP009682 CDKN1A_1 :+U EU:NC; range -1000 to 100.
View a graphic of the following search results

Identifier	Position	Mismatches	Score	Binding Factor	Sequence (Search Pattern)
DROME$EVE_08	2 (+)	0	100.00	GAGA factor	GAGAG
DROME$EVE_13	2 (+)	0	100.00	GAGA factor	GAGAG
DROME$FTZ_32	2 (+)	0	100.00	GAGA factor	GAGAG
AS$GAGA_03	2 (+)	0	100.00	GAGA factor	GAGAG
DROME$EVE_09	2 (-)	0	100.00	GAGA factor	CTCTC
DROME$FTZ_32	2 (-)	0	100.00	GAGA factor	CTCTC
MOUSE$M1H2KB_03	6 (+)	1	87.50	AP-2alphaA, AP-2alphaB	GCCCAGGGC
PA$PY_26	6 (+)	0	100.00	T-Ag	GCCCC
MAIZE$ADH11S_06	6 (+)	0	100.00	GCBP-1, Sp1	GCCCC
HS$MT2A_05	6 (-)	1	87.50	AP-2, AP-2alphaA, AP-2alphaB, AP-2beta, AP-2gamma	GCCCGCGGC
MOUSE$M1H2KB_03	6 (-)	1	87.50	AP-2alphaA, AP-2alphaB	GCCCAGGGC
PA$PY_23	6 (-)	0	100.00	T-Ag	GGGGC
SV$SV40_04	6 (-)	0	100.00	T-Ag	GGGGC
CHICK$ACC_02	6 (-)	1	87.50		GCCGGGGGC
SV$SV40_63	6 (-)	0	100.00	T-Ag	GGGGC
MAIZE$ADH1P_02	7 (+)	0	100.00		CCCCGG
Y$MAL2R_01	7 (+)	0	100.00	MIG1	CCCCGG
Y$MAL63_01	7 (+)	0	100.00	MIG1	CCCCGG
HS$CETP_02	10 (+)	0	100.00	LXR-alpha, LXR-beta, RXR-alpha	CGGGCA
HS$CYCD1_15	10 (-)	0	100.00	Sp1, Sp2, Sp3, Sp4	GCCCG
CHICK$BAG_03	11 (+)	0	100.00		GGGCA
RAT$NF1_01	11 (+)	0	100.00	LF-A1	GGGCA
PIG$UPA_09	11 (+)	0	100.00	Sp1	GGGCAG
HS$PFKM_03	11 (+)	1	88.89		GGGGAGGAGG
HS$CATHD_01	11 (+)	0	100.00	ER-alpha, Sp1	GGGCA
RAT$VEGF_01	11 (+)	0	100.00	ER-alpha, ER-beta	GGGCA
CHICK$BAG_03	11 (-)	0	100.00		TGCCC
RAT$VEGF_02	11 (-)	0	100.00	ER-alpha, ER-beta	TGCCC
MOUSE$IGKL_05	12 (+)	1	88.89	E12, E47, MyoD	GGCAGGTGGC
MOUSE$IGH_58	13 (+)	1	88.89	c-Ets-1 68	GCAGGAAGCA
HS$EGFR_20	13 (-)	0	100.00		TCCTGC
MOMLV$MOMULV_09	13 (-)	0	100.00	LVc	CCTGC
MOMLV$MOMULV_10	13 (-)	0	100.00	LVc	CCTGC
RADLV$RLV_03	13 (-)	0	100.00	LVc	CCTGC
HS$AACS_02	15 (+)	0	100.00	ARP-1	AGGAGG
AS$SPZ1_06	16 (+)	1	87.50	Spz1	GGAGGGAAA
Y$RPT2_01	16 (+)	1	87.50	RPN4	GGTGGCAAA
Y$RPT5_01	16 (+)	1	87.50	RPN4	GGTGGCAAA
Y$RPT4_01	16 (+)	1	87.50	RPN4	GGTGGCAAA
Y$RPT6_01	16 (+)	1	87.50	RPN4	GGTGGCAAA
HT1$HTLV1_08	16 (-)	0	100.00	c-Ets-1 68	CCTCC
PA$PY_24	17 (+)	0	100.00	T-Ag	GAGGC
PA$PY_25	17 (+)	0	100.00	T-Ag	GAGGC
SV$SV40_02	17 (+)	0	100.00	T-Ag	GAGGC
CHICK$ITGB3_01	17 (+)	0	100.00	RXR-beta, VDR	GAGGCA
SV$SV40_01	17 (-)	0	100.00	T-Ag	GCCTC
SV$SV40_02	17 (-)	0	100.00	T-Ag	GCCTC
SV$SV40_63	17 (-)	0	100.00	T-Ag	GCCTC
MOUSE$AFEP_14	18 (-)	0	100.00	C/EBPalpha, HNF-1, HNF-1A	TTGCCT
HS$CD2_04	18 (-)	1	88.89		ACTTTTTCCT
Y$ANB1_05	18 (-)	0	100.00	MOT3	TTGCCT
MMTV$MMTV_48	19 (-)	0	100.00	AP-2alpha, AP-2alphaA	TTTGCC
RAT$TAT_04	24 (+)	0	100.00	GR	AAGTCC
AS$PR_03	32 (+)	0	100.00	PR B	TGTTCC
AS$TR_07	33 (+)	0	100.00	T3R-alpha	GTTCC
AS$TR_10	33 (+)	0	100.00	T3R-alpha	GTTCC
HS$IL4_02	33 (-)	0	100.00		GGAAC
AS$STAT5A_51	34 (+)	1	87.50	STAT5A	TTCCGACTA
AS$STAT5A_57	34 (+)	1	87.50	STAT5A	TTCCGACTA
AS$STAT5A_70	34 (+)	1	87.50	STAT5A	TTCCAAGTA
Y$CYC1_09	36 (-)	0	100.00	hap2, HAP2, HAP3	GTTGG
Y$CYC1_10	36 (-)	0	100.00	CP1A, HAP2	GTTGG
Y$CYC1_11	36 (-)	0	100.00	HAP3, NF-YA	GTTGG
HS$PR264_06	37 (+)	0	100.00	c-Myb	CAACT
AS$EN1_05	37 (-)	0	100.00	En-1	ATAGTTG
Y$HIS3_03	43 (+)	0	100.00	GCN4	TAGTCA
AS$MSX1_04	43 (+)	1	87.50	Msx-1	TAGTGATTT
Y$HIS4_14	43 (-)	0	100.00	GCN4	TGACTA
RAT$CCSP_04	43 (-)	0	100.00	Fra-1, JunB, JunD	TGACTA
NT$CHN50_01	44 (+)	0	100.00	WRKY1, WRKY3, WRKY4	AGTCA
MOUSE$IL4_01	46 (+)	0	100.00	NF-CLE0a, NF-CLE0b	TCATTT
HS$GMCSF_03	47 (+)	0	100.00	YY1	CATTT
HS$HH4_07	47 (-)	0	100.00	HiNF-A	AGAAATG
Y$SUC2_06	48 (-)	0	100.00	MED8	AAGAAAT
Y$HXT1_01	48 (-)	0	100.00	MED8	AAGAAAT
HS$CMYC_13	49 (-)	1	87.50		GAAAAGAAA
HS$TNFA_04	49 (-)	0	100.00		AGAAA
I$HSF_01	49 (-)	0	90.00	HSF	AGAAN
F$HSF_01	49 (-)	0	90.00	HSF	AGAAN
FY$MFM1_02	50 (-)	1	87.50	mat1-Mc, ste11	AACAAAGAA
AS$HB_08	51 (-)	1	88.89	Hb	GCAGCAAAAA
HS$CYCD1_08	52 (+)	0	100.00	TCF-4E	CTTTGCT
MOUSE$THY1_06	54 (-)	0	100.00		CAGCAA
MOUSE$M2EAK_03	55 (-)	0	100.00	NF-Y	CAGCA
DROME$ADH_06	56 (+)	0	100.00	Adf-1	GCTGC
HS$CMYC_16	56 (+)	0	100.00	CTCF	GCTGC
AS$FTZ_52	56 (+)	0	100.00	Ftz	GCTGCA
DROME$ADH_14	56 (-)	0	100.00	Adf-1	GCAGC
MAIZE$C1_04	58 (+)	0	100.00	Vp1	TGCATG
RAPE$NAPA_01	58 (-)	0	100.00	ABI3, FUS3	CATGCA
MOUSE$IGH_55	61 (-)	0	100.00		ATCAT
MOUSE$TTPA_01	62 (+)	0	100.00	AP-1	TGATC
AS$GR_06	62 (+)	0	100.00	GR	TGATCT
HS$GHA_03	62 (-)	0	100.00	GR, GR-alpha, GR-beta	AGATCA
AD$E3_07	67 (-)	0	100.00	AP-1	TAACTCA
RAT$CYTOP_03	68 (+)	1	87.50		GAGTTGGGT
HS$SHP1_01	68 (+)	0	100.00	FXR, RXR-alpha	GAGTTA
MOUSE$RVL3_03	69 (-)	0	100.00	RXR-alpha	TAACT
AD$E4_25	70 (+)	1	87.50	ATF	GTTACGTCA
AD$E4_06	70 (-)	1	87.50	ATF, atf1, c-Jun, CRE-BP1, deltaCREB, E4BP4	TGACGTAAC
AD$E4_09	70 (-)	1	87.50	E4F	TGACGTAAC
E4F$CONS	70 (-)	1	87.50	E4F	TGACGTAAC
CHICK$OA_02	73 (+)	0	100.00	ARP-1, COUP, COUP-TF1, PPAR-alpha, RXR-alpha	AGGTCA
XENLA$VITA2_02	73 (+)	0	100.00	ER-alpha, ER-beta1, NHP-1, T3R-alpha1, T3R-beta1	AGGTCA
PPAR$CONS_01	73 (+)	0	100.00	PPAR-alpha	AGGTCA
AS$GR_12	73 (+)	0	100.00	GR-alpha, GR-beta	AGGTCA
RAT$CRBP2_01	73 (+)	0	100.00	COUP-TF1, PPAR-alpha, RAR-alpha1, RAR-beta, RXR-alpha, RXR-beta, TGIF	AGGTCA
RAT$CRBP1_01	73 (+)	0	100.00	COUP-TF1, RAR-beta, RXR-alpha	AGGTCA
AS$RAR_02	73 (+)	0	100.00	RAR-beta, RXR-alpha	AGGTCA
MOUSE$CRABP2_02	73 (+)	0	100.00	RXR-alpha, RXR-gamma	AGGTCA
AS$RXR_01	73 (+)	0	100.00	COUP-TF1, PPAR-alpha, RXR-alpha	AGGTCA
AS$RXR_02	73 (+)	0	100.00	COUP-TF1, RXR-alpha	AGGTCA
AS$RAR_03	73 (+)	0	100.00	RAR-alpha1, RXR-beta, T3R-beta1	AGGTCA
AS$NGFIB_01	73 (+)	0	100.00	NGFI-B	AGGTCA
MOUSE$CRBP1_01	73 (+)	0	100.00	RAR-alpha1, RAR-beta2, RAR-gamma1	AGGTCA
AS$RAR_07	73 (+)	0	100.00	RAR-alpha1, RXR-alpha	AGGTCA
AS$RAR_08	73 (+)	0	100.00	RAR-alpha1, RXR-alpha	AGGTCA
AS$RAR_09	73 (+)	0	100.00	RAR-alpha1, RXR-alpha	AGGTCA
AS$RAR_10	73 (+)	0	100.00	RAR-alpha1, RXR-alpha	AGGTCA
AS$RAR_11	73 (+)	0	100.00	RAR-alpha1, RXR-alpha	AGGTCA
AS$RAR_12	73 (+)	0	100.00	RAR-alpha1, RXR-alpha	AGGTCA
AS$RAR_13	73 (+)	0	100.00	RAR-alpha1, RXR-alpha	AGGTCA
AS$RAR_14	73 (+)	0	100.00	RAR-alpha1, RXR-alpha	AGGTCA
AS$RAR_15	73 (+)	0	100.00	RAR-alpha1, RXR-alpha	AGGTCA
AS$RAR_16	73 (+)	0	100.00	RAR-alpha1, RXR-alpha	AGGTCA
AS$RAR_17	73 (+)	0	100.00	RAR-alpha1, RXR-alpha	AGGTCA
AS$RAR_18	73 (+)	0	100.00	RAR-alpha1, RXR-alpha	AGGTCA
AS$RAR_21	73 (+)	0	100.00	RAR-alpha1, RXR-alpha	AGGTCA
AS$TR_15	73 (+)	0	100.00	RXR-alpha, T3R-beta1	AGGTCA
AS$RAR_23	73 (+)	0	100.00	RAR-alpha1, RXR-alpha	AGGTCA
AS$TR_16	73 (+)	0	100.00	T3R-beta1	AGGTCA
AS$TR_17	73 (+)	0	100.00	T3R-beta1	AGGTCA
AS$TR_18	73 (+)	0	100.00	RXR-alpha, T3R-beta1	AGGTCA
AS$RXR_03	73 (+)	0	100.00	RXR-alpha	AGGTCA
MOUSE$PCP2_01	73 (+)	0	100.00	RXR-beta, T3R-alpha1, T3R-beta1	AGGTCA
AS$RXR_04	73 (+)	0	100.00	ARP-1, RXR-alpha	AGGTCA
AS$RXR_05	73 (+)	0	100.00	ARP-1, RAR-alpha1, RXR-alpha	AGGTCA
AS$RXR_6	73 (+)	0	100.00	RXR-alpha, TAF(II)28	AGGTCA
AS$TR_20	73 (+)	0	100.00	RAR-beta, RXR-alpha, T3R-alpha1, T3R-beta1, T3R-beta2	AGGTCA
AS$TR_34	73 (+)	0	100.00	T3R-alpha1, T3R-alpha2	AGGTCA
MOUSE$MYOD_04	73 (+)	0	100.00	ARP-1, RXR-alpha, RXR-gamma, T3R-alpha	AGGTCA
AS$LXRA_01	73 (+)	0	100.00	LXR-alpha, RXR-alpha	AGGTCA
RAT$FAS_04	73 (+)	0	100.00	LXR-alpha, RXR-alpha	AGGTCA
AS$LXRB_01	73 (+)	0	100.00	LXR-beta, RXR-alpha	AGGTCA
AS$RXR_08	73 (+)	0	100.00	RXR-alpha	AGGTCA
AS$LXRB_02	73 (+)	0	100.00	LXR-beta, RXR-alpha	AGGTCA
AS$FXR_01	73 (+)	0	100.00	FXR, RXR-alpha	AGGTCA
AS$VDR_01	73 (+)	0	100.00	RXR-alpha, VDR	AGGTCA
AS$GCNF_02	73 (+)	0	100.00	GCNF	AGGTCA
AS$GCNF_03	73 (+)	0	100.00	GCNF	AGGTCA
AS$GCNF_05	73 (+)	0	100.00	GCNF	AGGTCA
AS$SXR_01	73 (+)	0	100.00	RXR-alpha, SXR	AGGTCA
HS$CYP3A4_01	73 (+)	0	100.00	PXR-1, RXR-alpha, RXR-beta, SXR	AGGTCA
AS$BXR2_01	73 (+)	0	100.00	BXR-beta	AGGTCA
AS$BXR2_02	73 (+)	0	100.00	BXR-beta	AGGTCA
AS$ERR1_03	73 (+)	0	100.00	ERR1	AGGTCA
AS$ERR1_04	73 (+)	0	100.00	ERR1	AGGTCA
AS$ERR1_05	73 (+)	0	100.00	ERR1	AGGTCA
AS$ERR1_07	73 (+)	0	100.00	ERR1	AGGTCA
AS$ERR1_08	73 (+)	0	100.00	ERR1	AGGTCA
AS$ERR1_09	73 (+)	0	100.00	ERR1	AGGTCA
AS$ERR1_11	73 (+)	0	100.00	ERR1	AGGTCA
AS$ERR1_13	73 (+)	0	100.00	ERR1	AGGTCA
AS$ERR1_15	73 (+)	0	100.00	ERR1	AGGTCA
AS$ERR1_17	73 (+)	0	100.00	ERR1	AGGTCA
AS$ERR1_20	73 (+)	0	100.00	ERR1	AGGTCA
AS$ERR1_21	73 (+)	0	100.00	ERR1	AGGTCA
AS$ERR1_22	73 (+)	0	100.00	ERR1	AGGTCA
AS$ERR1_24	73 (+)	0	100.00	ERR1	AGGTCA
AS$ERR1_25	73 (+)	0	100.00	ERR1	AGGTCA
AS$TR_35	73 (+)	0	100.00	T3R-alpha	AGGTCA
AS$REVERBA_01	73 (+)	0	100.00	REVERB-alpha	AGGTCA
AS$REVERBA_02	73 (+)	0	100.00	REVERB-alpha	AGGTCA
AS$REVERBA_03	73 (+)	0	100.00	REVERB-alpha	AGGTCA
AS$REVERBA_04	73 (+)	0	100.00	REVERB-alpha	AGGTCA
AS$REVERBA_05	73 (+)	0	100.00	REVERB-alpha	AGGTCA
MOUSE$CRBP1_02	73 (+)	0	100.00	REVERB-alpha	AGGTCA
MOUSE$PTHR_01	73 (+)	0	100.00	COUP-TF1, RAR-alpha1, RXR-alpha	AGGTCA
RAT$CYP3A23_01	73 (+)	0	100.00	PXR-1	AGGTCA
MOUSE$AQP7_01	73 (+)	0	100.00	PPAR-alpha, PPAR-gamma, RXR-alpha	AGGTCA
XENLA$VITA2_02	73 (-)	0	100.00	ER-alpha, ER-beta1, NHP-1, T3R-alpha1, T3R-beta1	TGACCT
AS$GR_12	73 (-)	0	100.00	GR-alpha, GR-beta	TGACCT
CHICK$OA_12	73 (-)	0	100.00	ER-alpha	TGACCT
CHICK$OA_13	73 (-)	0	100.00	ER-alpha	TGACCT
CHICK$OA_15	73 (-)	0	100.00	ER-alpha	TGACCT
MOUSE$CRABP2_01	73 (-)	0	100.00	RXR-alpha, RXR-gamma	TGACCT
AS$TR_16	73 (-)	0	100.00	T3R-beta1	TGACCT
AS$TR_17	73 (-)	0	100.00	T3R-beta1	TGACCT
AS$TR_18	73 (-)	0	100.00	RXR-alpha, T3R-beta1	TGACCT
RAT$AOX_01	73 (-)	0	100.00	PPAR-alpha, RXR-alpha	TGACCT
BOVIN$OT_01	73 (-)	0	100.00	SF-1	TGACCT
AS$SF1_01	73 (-)	0	100.00	SF-1	TGACCT
AS$FXR_01	73 (-)	0	100.00	FXR, RXR-alpha	TGACCT
HS$SHP1_01	73 (-)	0	100.00	FXR, RXR-alpha	TGACCT
MOUSE$CRABP1_01	73 (-)	0	100.00	TR2-11	TGACCT
CHICK$OA_03	74 (+)	0	100.00	ER-alpha	GGTCA
CHICK$OA_04	74 (+)	0	100.00	c-Fos, c-Jun, ER-alpha	GGTCA
AS$RAR_04	74 (+)	0	100.00	RAR-alpha1, T3R-alpha	GGTCA
AS$RAR_06	74 (+)	0	100.00	RAR-alpha1	GGTCA
AS$TR_08	74 (+)	0	100.00	T3R-alpha	GGTCA
AS$TR_09	74 (+)	0	100.00	RAR-alpha1, T3R-alpha	GGTCA
AS$TR_10	74 (+)	0	100.00	T3R-alpha	GGTCA
NT$CHN50_01	74 (+)	0	100.00	WRKY1, WRKY3, WRKY4	GGTCA
MOUSE$AFEP_13	74 (-)	0	100.00	NP-III	GTGACC
HS$ADH3_01	74 (-)	0	100.00	CAR, RAR-alpha1, RAR-beta, RXR-alpha	TGACC
CHICK$OA_14	74 (-)	0	100.00	ER-alpha	TGACC
MOUSE$FCGR3A_01	74 (-)	1	90.00	TFE3, USF1	GTCTGCTGACC
AS$ZIC3_10	74 (-)	1	87.50	Zic3	CGGGTGACC
HS$MMP1_06	75 (+)	0	100.00		GTCAC
HS$P53_03	75 (-)	0	100.00	Pax-2, Pax-5, Pax-8	GTGAC
HS$APOA2_06	76 (+)	0	100.00		TCACC
AS$ZIC2_12	77 (-)	1	87.50	Zic2	TGTCTGGTG
MOUSE$GSHPX1_01	84 (+)	0	100.00	PU.1	CTTCTC
RAT$RPK_01	84 (-)	1	87.50	GATA-1	TCAGATAAG
I$HSF_01	84 (-)	0	90.00	HSF	AGAAN
F$HSF_01	84 (-)	0	90.00	HSF	AGAAN
RAT$POMC_03	87 (-)	0	100.00	GR	CAGAG
TDNA$NOS_01	90 (+)	0	100.00	ASF-1, OBF3.1, TGA1a, TGA1b	TGAGC
PA$PY_26	93 (+)	0	100.00	T-Ag	GCCCC
MAIZE$ADH11S_06	93 (+)	0	100.00	GCBP-1, Sp1	GCCCC
PA$PY_23	93 (-)	0	100.00	T-Ag	GGGGC
SV$SV40_04	93 (-)	0	100.00	T-Ag	GGGGC
SV$SV40_63	93 (-)	0	100.00	T-Ag	GGGGC
Y$GAL3_01	94 (+)	0	100.00	MIG1	CCCCAG
Y$GAL4_01	94 (+)	0	100.00	MIG1	CCCCAG
Y$HAP4_01	94 (+)	0	100.00	MIG1	CCCCAG
SV$SV40_37	95 (-)	0	100.00		CTGGG
CHICK$STN1_02	96 (+)	0	100.00	LBP-1, NF-1 (-like proteins)	CCAGT
HS$PAI_07	96 (+)	0	100.00		CCAGT
MOUSE$TS_04	98 (+)	1	87.50		AGTTTCCCA
AS$AREB6_44	99 (+)	0	100.00	AREB6	GTTTC
AS$AREB6_46	99 (+)	0	100.00	AREB6	GTTTC
AS$AREB6_48	99 (+)	0	100.00	AREB6	GTTTC
AS$AREB6_49	99 (+)	0	100.00	AREB6	GTTTC
AS$AREB6_50	99 (+)	0	100.00	AREB6	GTTTC
AS$AREB6_52	99 (+)	0	100.00	AREB6	GTTTC
AS$AREB6_53	99 (+)	0	100.00	AREB6	GTTTC
CHICK$APOVLDL_04	99 (-)	0	100.00	C/EBPalpha	GGGGAAAC
HS$IFI616_01	100 (-)	0	100.00	ISGF-3	GGAAA
MOUSE$IL5_02	100 (-)	0	100.00	NF-ATc, NF-ATp, NF-ATx	GGAAA
HS$IL4_01	100 (-)	0	100.00		GGAAA
MOUSE$IL4_06	100 (-)	0	100.00		GGAAA
MOUSE$IL4_07	100 (-)	0	100.00		GGAAA
MOUSE$IL4_08	100 (-)	0	100.00		GGAAA
HS$IL5_02	100 (-)	0	100.00		GGAAA
HS$IL13_01	100 (-)	0	100.00		GGAAA
MOUSE$CD40L_01	100 (-)	0	100.00		GGAAA
MOUSE$CD40L_02	100 (-)	0	100.00		GGAAA
MOUSE$CD40L_03	100 (-)	0	100.00		GGAAA
MOUSE$CD40L_04	100 (-)	0	100.00		GGAAA
HS$CD40L_01	100 (-)	0	100.00		GGAAA
HS$CD40L_02	100 (-)	0	100.00		GGAAA
HS$IFNG_03	100 (-)	0	100.00		GGAAA
HS$IFNG_04	100 (-)	0	100.00		GGAAA
HS$TNFA_05	100 (-)	0	100.00		GGAAA
MOUSE$MCP3_01	100 (-)	0	100.00		GGAAA
HS$TNFSF6_01	100 (-)	0	100.00		GGAAA
MOUSE$M1H2KB_07	102 (+)	0	100.00	MBP-1 (1), NF-kappaB1, NF-kappaB2, NF-kappaB2 precursor	TCCCCA
MOUSE$M1H2KB_02	102 (-)	0	100.00	AP-2alpha, AP-2alphaA, AP-2alphaB	TGGGGA
MOUSE$M1H2KB_07	102 (-)	0	100.00	MBP-1 (1), NF-kappaB1, NF-kappaB2, NF-kappaB2 precursor	TGGGGA
Y$GAL3_01	103 (+)	0	100.00	MIG1	CCCCAG
Y$GAL4_01	103 (+)	0	100.00	MIG1	CCCCAG
Y$HAP4_01	103 (+)	0	100.00	MIG1	CCCCAG
SV$SV40_36	103 (-)	0	100.00	AP-2alphaA, AP-2alphaB, AP-2beta	TGCTGGGG
SV$SV40_37	104 (-)	0	100.00		CTGGG
CHICK$STN1_01	105 (+)	0	100.00	LBP-1, MyoD	CCAGC
CHICK$STN1_03	105 (+)	0	100.00	LBP-1, NF-1 (-like proteins)	CCAGC
MOUSE$M2EAK_03	106 (+)	0	100.00	NF-Y	CAGCA
AS$mEMBP_04	109 (-)	0	100.00	EmBP-1a	CACTG
SIF$CONS	116 (-)	0	91.67	SIF	CCCGTM
HS$CYCD1_15	118 (-)	0	100.00	Sp1, Sp2, Sp3, Sp4	GCCCG
RAT$CAT_01	125 (+)	1	87.50		TGGGGGGAG
HS$IL3_08	125 (+)	0	100.00	AML1, AML1a, AML1c	TGTGGG
V$MZF1_01	125 (+)	0	87.50	MZF1	NGNGGGGA
Y$MEL1_02	126 (+)	0	100.00	MIG1	GTGGGG
Y$MAL61_03	126 (+)	0	100.00	MIG1	GTGGGG
Y$MAL61_04	126 (+)	0	100.00	MIG1	GTGGGG
MZF1$CONS_01	126 (+)	0	92.86	MZF-1	GNGGGGA
MOUSE$M1H2KB_02	127 (+)	0	100.00	AP-2alpha, AP-2alphaA, AP-2alphaB	TGGGGA
MOUSE$M1H2KB_07	127 (+)	0	100.00	MBP-1 (1), NF-kappaB1, NF-kappaB2, NF-kappaB2 precursor	TGGGGA
AS$ZIC_12	127 (+)	0	100.00	Zic1	TGGGGAGTA
MOUSE$M1H2KB_07	127 (-)	0	100.00	MBP-1 (1), NF-kappaB1, NF-kappaB2, NF-kappaB2 precursor	TCCCCA
AS$SPZ1_27	128 (+)	1	87.50	Spz1	GGGGGGTAT
MOUSE$HOXA5_01	129 (-)	0	100.00	HOXA5	ACTCCC
AS$ZIC2_08	130 (+)	1	87.50	Zic2	GGAGTAGTC
RAT$GHF1_01	132 (+)	0	100.00	PTF	AGTAT
RAT$GHF1_01	132 (-)	0	100.00	PTF	ATACT
Y$GAL3_01	134 (+)	0	100.00	MIG1	TATTC
MOUSE$PCP2_01	139 (+)	0	100.00	RXR-beta, T3R-alpha1, T3R-beta1	AGGAGA
RAT$ANP_01	140 (+)	0	100.00		GGAGAC
Y$ADH2_01	140 (-)	0	100.00	ADR1	TCTCC
AS$ARF1_01	141 (+)	0	100.00	ARF1, ARF5	GAGACA
PEA$IAA45_07	141 (+)	0	100.00	ARF1	GAGACA
YL$XPR2_01	141 (-)	0	100.00		GTCTC
AS$ARF1_01	141 (-)	0	100.00	ARF1, ARF5	TGTCTC
AS$ARF1_05	141 (-)	0	100.00	ARF1, ARF4, ARF5, ARF6, ARF7, ARF8	TGTCTC
HS$PAI1_03	142 (+)	1	87.50		AGCCAGACA
AS$TGIF_09	142 (-)	0	100.00	TGIF	TGTCT
AS$MEIS1_10	142 (-)	0	100.00	Meis-1a, Meis-1b	CTGTCT
HS$GG_12	143 (-)	0	100.00	NF-E	CTGTC
DROME$EVE_10	143 (-)	0	100.00	GAGA factor	CTGTC
AS$TGIF_09	146 (-)	0	100.00	TGIF	TGTCT
AS$SRY_09	147 (+)	1	88.89	SRY	GACAAATCAC
AS$ZIC3_02	147 (-)	1	87.50	Zic3	TGTGTTGTC
HS$PR264_06	149 (+)	0	100.00	c-Myb	CAACT
DROME$UBX_22	150 (+)	1	88.89	Zeste	CACTCACTCG
DROME$UBX_10	154 (+)	0	100.00	Zeste	CACTCG
AS$WZF1_01	154 (+)	0	100.00	WZF1	CACTC
PV$PHSB_02	154 (+)	1	87.50	ROM1, ROM2	CACACGTCA
WHEAT$H3_02	154 (+)	0	100.00	WZF1	CACTC
WHEAT$H3_03	154 (+)	0	100.00	WZF1	CACTC
DROME$WHLO_01	154 (-)	0	100.00	Zeste	CGAGTG
DROME$ZESTE_01	154 (-)	0	100.00	Zeste	CGAGTG
AS$NCX_34	157 (+)	1	88.89	Ncx	TGGTCAAATC
Y$HIS3_01	157 (-)	0	100.00	GCN4	TGACGA
HT1$HTLV1_20	157 (-)	0	100.00	TAF-I, TAF-II, Tax	TGACGA
RAT$SMAD7_03	157 (-)	0	100.00		TGACGA
HS$VIP_01	158 (+)	0	100.00	c-Fos, c-Jun, CRE-BP1, CREB	CGTCA
CAMV$35SR_01	158 (-)	0	100.00	ASF-1, OBF4, OBF5, SARP, TGA1, TGA1a, TGA1b, TGA2, TGA2.1, TGA2.2, TGA3, TGA6, ZAP1	TGACG
PIG$UPA_02	158 (-)	0	100.00	CREB	TGACG
PIG$UPA_03	158 (-)	0	100.00	CREB, CREBbeta	TGACG
HS$INS_04	158 (-)	0	100.00	CREB	TGACG
HS$PL_12	158 (-)	0	100.00		TGACG
AS$PAX2_69	159 (+)	1	87.50	Pax-2.1, Pax-2.2	GTCAAATCA
AS$TWRKY_01	159 (-)	0	100.00	WRKY3, WRKY4	TTGAC
AS$WRKY_01	159 (-)	0	100.00	WRKY1, WRKY2	TTGAC
AS$WRKY_02	159 (-)	0	100.00	WRKY1, WRKY2	TTGAC
AS$WRKY_03	159 (-)	0	100.00	WRKY1, WRKY2	TTGAC
AS$WRKY_04	159 (-)	0	100.00	WRKY1, WRKY2	TTGAC
AT$RLK4_01	159 (-)	0	100.00	WRKY18	TTGAC
AT$RLK4_02	159 (-)	0	100.00	WRKY18	TTGAC
DROME$UBX_24	160 (-)	0	100.00	LEF-1	TTTGA
HS$EGFR_14	161 (+)	0	100.00		CAAAT
MAIZE$PMS1_01	161 (+)	0	100.00		CAAAT
HS$IGH_04	161 (-)	0	100.00		ATTTG
AS$SPZ1_16	162 (-)	0	100.00	Spz1	GGAGGATTT
CHICK$H5_05	163 (+)	1	88.89	H4TF-2	AGTCCTCCCC
AS$ARR_01	163 (-)	0	100.00	ARR1, ARR2	AGGATT
HT1$HTLV1_08	166 (+)	0	100.00	c-Ets-1 68	CCTCC
MOUSE$AQP7_01	168 (-)	0	100.00	PPAR-alpha, PPAR-gamma, RXR-alpha	AGGGGA
AS$VDR_04	169 (-)	0	100.00	VDR	AGGGG
MOUSE$B29_02	169 (-)	1	90.91	NERF-1a	GGCAGGAAGGGG
Y$GLK1_02	169 (-)	0	100.00	MSN2, MSN4	AGGGG
MOUSE$JUNB_01	170 (+)	1	87.50		CGCTTCCTG
XENLA$RPL14_01	172 (+)	0	100.00	HrpF, XrpFI	CTTCC
Y$TPI_01	172 (+)	0	100.00	GCR1	CTTCC
MOUSE$RPL32_01	172 (-)	0	100.00	f(alpha)-f(epsilon), HrpF, XrpFI	GGAAG
PEA3$CONS	172 (-)	0	91.67	c-Ets-1 54, c-Ets-1 68, c-Ets-2 58-64, PEA3	AGGAAR
HS$TIMP1_02	172 (-)	0	100.00	c-Ets-1	CCAGGAAG
MOUSE$TIMP1_02	172 (-)	0	100.00	c-Ets-1	CAGGAAG
HS$TNFA_02	172 (-)	0	100.00		GGAAG
HS$TNFA_03	172 (-)	0	100.00		GGAAG
AS$ELK1_13	173 (+)	0	100.00	Elk-1, SAP-1a, SAP-1b, SRF	TTCCTG
DROME$E74_10	173 (-)	0	100.00	E74A	AGGAA
DROME$E74_11	173 (-)	0	100.00	E74A	AGGAA
E74A$CONS_01	173 (-)	0	90.00	E74A	MGGAA
MOUSE$DAX1_02	174 (+)	1	87.50	SF-1	TCATGGCCA
HS$GRH_01	177 (+)	0	100.00	AP-2, AP-2alphaA, AP-2alphaB, NF-1	TGGCC
HS$GRH_01	177 (-)	0	100.00	AP-2, AP-2alphaA, AP-2alphaB, NF-1	GGCCA
RAT$TAT_15	177 (-)	0	100.00	HNF-3alpha	GGCCA
HS$GRH_01	178 (+)	0	100.00	AP-2, AP-2alphaA, AP-2alphaB, NF-1	GGCCA
RAT$TAT_15	178 (+)	0	100.00	HNF-3alpha	GGCCA
HS$GRH_01	178 (-)	0	100.00	AP-2, AP-2alphaA, AP-2alphaB, NF-1	TGGCC
HBV$SA_01	179 (+)	0	100.00	NF-1	GCCAAC
MOUSE$AP2_07	179 (-)	0	100.00	NF-1	TTGGC
MOUSE$HTF9_04	179 (-)	1	88.89		CTTTATTGGC
Y$CYC1_09	180 (-)	0	100.00	hap2, HAP2, HAP3	GTTGG
Y$CYC1_10	180 (-)	0	100.00	CP1A, HAP2	GTTGG
Y$CYC1_11	180 (-)	0	100.00	HAP3, NF-YA	GTTGG
AS$SRY_21	181 (+)	1	88.89	SRY	AAACAAAGCT
AS$HOXA3_08	181 (-)	1	87.50	HOXA3	CCTTTGTTG
HS$CD3E_01	182 (+)	0	100.00	LEF-1, mat1-Mc, Sox-13, Sox-5, SRY, TCF-1, TCF-1A, TCF-1B, TCF-1C, TCF-1E, TCF-1F, TCF-1G	AACAAAG
MOUSE$SRY_01	182 (+)	0	100.00	SRY	AACAAAG
HS$SRY_01	182 (+)	0	100.00	SRY	AACAAAG
HS$ADA_08	182 (+)	0	100.00	LEF-1	AACAAAG
AS$TCF1_01	182 (+)	0	100.00	TCF-1B	AACAAAG
AS$TCF1_02	182 (+)	0	100.00	TCF-1B	AACAAAG
AS$SOX15_02	182 (+)	0	100.00	Sox15	AACAAAG
MOUSE$LY49A_01	182 (-)	0	100.00	TCF-1(P)	CTTTGTT
XENLA$ENGRAILED2_03	182 (-)	0	100.00	LEF-1	CTTTGTT
AS$FTZ_49	185 (+)	0	100.00	Ftz	AAAGCT
HS$PAI_07	186 (+)	0	100.00		AAGCTG
DROME$ADH_06	188 (+)	0	100.00	Adf-1	GCTGC
HS$CMYC_16	188 (+)	0	100.00	CTCF	GCTGC
DROME$ADH_14	188 (-)	0	100.00	Adf-1	GCAGC
MOUSE$M2EAK_03	190 (-)	0	100.00	NF-Y	CAGCA
DROME$ADH_06	191 (+)	0	100.00	Adf-1	GCTGC
HS$CMYC_16	191 (+)	0	100.00	CTCF	GCTGC
AS$FTZ_52	191 (+)	0	100.00	Ftz	GCTGCA
DROME$ADH_14	191 (-)	0	100.00	Adf-1	GCAGC
AS$ZIC2_10	192 (-)	1	87.50	Zic2	TGGTTGCTG
HBV$HBVE_14	194 (-)	0	100.00	EF-C	GTTGC
RAT$CMOS_02	195 (+)	0	100.00		CAACCAC
MOUSE$IGH_61	195 (-)	1	87.50	core-binding factor	CTGTGGTTT
AS$EN1_03	195 (-)	0	100.00	En-1	GTGGTTG
AS$AML1_03	196 (+)	0	100.00	PEBP2alphaB1, PEBP2alphaB2	AACCACA
MOUSE$MPO_01	196 (+)	0	100.00	AML1, AML3, PEBP2alphaB1	AACCACA
MOUSE$IAP_01	197 (-)	0	100.00		GTGGT
MOUSE$IAP_03	197 (-)	0	100.00	EBP-80	GTGGT
AS$AML1_01	197 (-)	0	100.00	AML1	TGTGGT
V$AML1_01	197 (-)	0	100.00	AML-1a	TGTGGT
AS$ZIC3_26	202 (+)	1	87.50	Zic3	AGGGAGTTC
MOUSE$IL2_10	203 (-)	1	87.50	NF-AT3, NF-ATc, NF-ATp, NF-ATx	AGAAATTCC
Y$CUP1_05	204 (+)	1	88.89	CUP2	GCATTTCTTC
HS$HH4_01	205 (+)	0	100.00	H4TF-1	GATTTC
Y$SUC2_06	206 (-)	0	100.00	MED8	AAGAAAT
Y$HXT1_01	206 (-)	0	100.00	MED8	AAGAAAT
HS$TNFA_04	207 (-)	0	100.00		AGAAA
I$HSF_01	207 (-)	0	90.00	HSF	AGAAN
F$HSF_01	207 (-)	0	90.00	HSF	AGAAN
MOUSE$RAS1_02	209 (+)	0	100.00	GR	TCTTCT
I$HSF_01	210 (-)	0	90.00	HSF	AGAAN
F$HSF_01	210 (-)	0	90.00	HSF	AGAAN
MOMLV$MOMULV_01	213 (-)	0	100.00	LVa	GAACAG
MOMLV$MOMULV_02	213 (-)	0	100.00	LVa	GAACAG
RAT$CYTOPB_01	214 (+)	0	100.00	GR	TGTTCA
AS$GR_07	214 (+)	0	100.00	GR	TGTTCA
MOUSE$CRISP1_03	214 (+)	0	100.00	AR	TGTTCA
AS$RAR_05	215 (+)	0	100.00	RAR-alpha1	GTTCA
AS$RAR_06	215 (+)	0	100.00	RAR-alpha1	GTTCA
AS$TR_06	215 (+)	0	100.00	T3R-alpha	GTTCA
AS$TR_07	215 (+)	0	100.00	T3R-alpha	GTTCA
AS$TR_08	215 (+)	0	100.00	T3R-alpha	GTTCA
AS$TR_11	215 (+)	0	100.00	T3R-alpha	GTTCA
MOUSE$RARA2_01	215 (+)	0	100.00	RAR-alpha1, RAR-beta2, RAR-gamma1	GTTCA
HS$ADH3_01	215 (-)	0	100.00	CAR, RAR-alpha1, RAR-beta, RXR-alpha	TGAAC
DELTAEF1$CONS_01	217 (-)	0	92.86	deltaEF1	CACCTNA
AS$AREB6_05	217 (-)	1	87.50	AREB6	CACACCTGA
AS$AREB6_23	217 (-)	0	100.00	AREB6	TCACCTGA
AS$AREB6_25	217 (-)	0	100.00	AREB6	TCACCTGA
MOUSE$CD4_03	218 (+)	0	100.00	E12, HEB	CAGGTG
DROME$HB_12	218 (+)	0	100.00	Ac, AS-C T3, Da, Sc	CAGGTG
DROME$AC_01	218 (+)	0	100.00	Ac, AS-C T3, Da, Sc	CAGGTG
AS$SNAIL_01	218 (+)	0	100.00	Sn	CAGGTG
AS$LMO2_01	218 (+)	0	100.00	CLIM2, E12, E47, GATA-1, Lmo2, Tal-1	CAGGTG
HS$VEGF_02	218 (+)	0	100.00		CAGGT
MOUSE$ACRD_02	218 (-)	0	100.00	E12, E47, MyoD, myogenin	CACCTG
XENLA$AC_05	218 (-)	0	100.00	EMF1, MyoD	CACCTG
HS$CXCR4_02	218 (-)	0	100.00	c-Myc, USF2	CACCTG
PV$PHASL_02	218 (-)	0	100.00	CAN	CACCTG
HS$RARG_01	219 (+)	0	100.00	RAR-alpha1, RAR-beta, RAR-gamma, RXR-alpha	AGGTGA
HSV1$TK_12	219 (+)	0	100.00	RAR-alpha1, T3R-alpha	AGGTGA
MOUSE$IBABP_01	219 (+)	0	100.00	FXR, RXR-alpha	AGGTGA
AS$ERR1_16	219 (+)	0	100.00	ERR1	AGGTGA
RAT$ME_08	219 (+)	0	100.00	PPAR-gamma, RXR-gamma	AGGTGA
HS$CAMHC_01	219 (-)	0	100.00	Hp55, Hp65, p58, RAR-alpha1, RXR-beta, T3R-alpha, T3R-beta1, T3R-beta2	TCACCT
HS$CYP24_02	219 (-)	0	100.00	VDR	TCACCT
RAT$CYP24_01	219 (-)	0	100.00	VDR	TCACCT
HS$PR264_01	220 (+)	0	100.00	c-Myb	GGTGAG
HS$APOA2_06	220 (-)	0	100.00		TCACC
AS$GHBF_02	220 (-)	1	87.50	G/HBF-1	AGACTCACC
Y$ILV1_02	222 (+)	0	100.00	GCN4	TGAGTG
DROME$WHLO_02	222 (+)	0	100.00	Zeste	TGAGTG
DROME$WHLO_05	222 (+)	0	100.00	Zeste	TGAGTG
DROME$DPP_03	222 (+)	0	100.00	Zeste	TGAGTG
DROME$UBX_03	222 (-)	0	100.00	Zeste	CACTCA
DROME$UBX_04	222 (-)	0	100.00	Zeste	CACTCA
DROME$WHLO_03	222 (-)	0	100.00	Zeste	CACTCA
DROME$WHLO_06	222 (-)	0	100.00	Zeste	CACTCA
DROME$WHLO_07	222 (-)	0	100.00	Zeste	CACTCA
DROME$WHLO_08	222 (-)	0	100.00	Zeste	CACTCA
DROME$WHLO_09	222 (-)	0	100.00	Zeste	CACTCA
DROME$DPP_02	222 (-)	0	100.00	Zeste	CACTCA
DROME$DPP_04	222 (-)	0	100.00	Zeste	CACTCA
AS$WZF1_01	223 (-)	0	100.00	WZF1	CACTC
WHEAT$H3_02	223 (-)	0	100.00	WZF1	CACTC
WHEAT$H3_03	223 (-)	0	100.00	WZF1	CACTC
FY$RPL27A2_01	224 (+)	1	88.89		AGGGTAGGGT
CHICK$PTH_01	230 (+)	0	100.00	RXR-alpha, VDR	GGGTGT
HS$BG_01	230 (-)	0	100.00	CACCC-binding factor	CACCC
HS$GG_13	230 (-)	0	100.00	gammaCAC1, gammaCAC2	CACCC
HS$GG_14	230 (-)	0	100.00	CACCC-binding factor, Sp1	CACCC
HS$GP2B_01	230 (-)	0	100.00	CACCC-binding factor	CACCC
RAT$TOA_02	230 (-)	0	100.00	CACCC-binding factor	CACCC
HS$EG_06	230 (-)	0	100.00	Sp1	CACCC
AS$CACCC_01	230 (-)	0	100.00	CACCC-binding factor	CACCC
HS$CYP24_01	230 (-)	0	100.00	VDR	ACACCC
HS$CS1_04	236 (+)	0	100.00	T3R	AGGGAG
HS$P21WAF1_05	236 (+)	0	100.00	RXR-alpha, VDR	AGGGAG
AS$VDR_08	236 (+)	0	100.00	VDR	AGGGAG
AS$LYF1_05	237 (+)	0	100.00	LyF-1	GGGAGA
CHICK$ITGB3_01	237 (+)	0	100.00	RXR-beta, VDR	GGGAGA
AS$CRX_01	237 (-)	1	90.91	Crx	AACTAATCTCCC
Y$ADH2_01	238 (-)	0	100.00	ADR1	TCTCC
HS$GG_26	240 (+)	0	100.00	GATA-1	AGATTG
MOUSE$BMG_04	240 (-)	0	100.00	GATA-1	AATCT
HS$HH1_02	241 (+)	0	100.00	H1TF1, HiNF-B	GATTGG
HS$HSP70_06	241 (+)	0	100.00	CCAAT-binding factor	GATTGG
HS$HSP70_09	241 (+)	0	100.00	CP1, CTF	GATTGG
MOUSE$A11COL_01	242 (+)	0	100.00	CBF (2), IF2	ATTGG
MOUSE$A21COL_03	242 (+)	0	100.00	CCAAT-binding factor	ATTGG
MOUSE$A21COL_04	242 (+)	0	100.00	CBF (2), CBF-A, CBF-B	ATTGG
MOUSE$A21COL_05	242 (+)	0	100.00	EFI	ATTGG
AD2$E2L_03	242 (+)	0	100.00	CRF	ATTGG
HS$GHA_06	242 (+)	0	100.00	NF-1	ATTGG
SP$H2B1_02	242 (+)	0	100.00	CBF (1)	ATTGG
SP$H2B1_03	242 (+)	0	100.00	CDF	ATTGG
HS$HSP70_02	242 (+)	0	100.00	CTF	ATTGG
HS$HSP70_07	242 (+)	0	100.00	CBTF, CP1, CTF	ATTGG
AD$MLP_03	242 (+)	0	100.00	CP1A, NF-YA	ATTGGTT
MOUSE$M2EAK_07	242 (+)	0	100.00	NF-Y	ATTGG
MOUSE$M2EAK_08	242 (+)	0	100.00	NF-Y	ATTGG
RSV$RSV_05	242 (+)	0	100.00		ATTGG
HSV1$TK_04	242 (+)	0	100.00	C/EBPalpha	ATTGG
HSV1$TK_05	242 (+)	0	100.00	NF-Y'	ATTGG
AS$HAP23_01	242 (+)	0	100.00	HAP2, HAP3, HAP4	ATTGG
MOUSE$M1H2KB_12	242 (+)	0	100.00	EFI	ATTGG
RAT$TH2A_02	242 (+)	0	100.00		ATTGG
RAT$TH2A_04	242 (+)	0	100.00		ATTGG
HS$TK_02	242 (+)	0	100.00	CP1	ATTGG
MOUSE$A11COL_07	242 (+)	0	100.00	NF-1, Sp1	ATTGG
HS$CDC2_07	242 (+)	0	100.00	CBAF, CBF-B, CP1A	ATTGG
XENLA$GATA2_01	242 (+)	0	100.00	CBTF	ATTGG
RAT$NAT_05	242 (+)	0	100.00		ATTGG
HS$CDC2_12	242 (+)	0	100.00	CBAF	ATTGG
HS$BAC_03	242 (-)	0	100.00	SRF	CCAAT
RAT$ALBU_22	242 (-)	0	100.00	ACF	AACCAAT
AMULV$AMULV_01	242 (-)	0	100.00		CCAAT
EBV$DSL_06	242 (-)	0	100.00		CCAAT
MOUSE$AAG_04	242 (-)	0	100.00	alpha-CP1, alpha-CP2a, alpha-CP2b, alpha-IRP	CCAAT
CHICK$BAG_06	242 (-)	0	100.00	TGGCA-binding protein	CCAAT
HS$GG_15	242 (-)	0	100.00	NF-E	CCAAT
HS$GG_16	242 (-)	0	100.00	CDP2, Clox, CUTL1, Cutl1	CCAAT
HS$GG_17	242 (-)	0	100.00	gammaCAAT	CCAAT
HS$GG_18	242 (-)	0	100.00	CP1	CCAAT
HS$ZG_07	242 (-)	0	100.00	CP2	CCAAT
HS$ZG_08	242 (-)	0	100.00	CP1	CCAAT
HS$HH1_03	242 (-)	0	100.00	H1TF2	CCAAT
MOUSE$M2IAB_01	242 (-)	0	100.00	PU.1	CCAAT
PIG$UPA_12	242 (-)	0	100.00	NF-1	CCAAT
RADLV$RLV_10	242 (-)	0	100.00		CCAAT
MULV$MULV_03	242 (-)	0	100.00		CCAAT
MOUSE$GLUT4_04	242 (-)	0	100.00		CCAAT
MOMLV$MOMULV_15	242 (-)	0	100.00		CCAAT
RAT$ALDB_02	242 (-)	0	100.00	CBF (2), CP2	CCAAT
HS$GG_20	242 (-)	0	100.00	CP1, NF-E3	CCAAT
RAT$TH2B_01	242 (-)	0	100.00		CCAAT
RAT$TH2B_02	242 (-)	0	100.00		CCAAT
HS$GG_21	242 (-)	0	100.00	CP1	CCAAT
MOUSE$LY6E_01	242 (-)	0	100.00		CCAAT
RAT$NEU_01	242 (-)	0	100.00		CCAAT
HS$GHA_10	242 (-)	0	100.00	alpha-CBF	CCAAT
HS$GG_33	242 (-)	0	100.00	CP1, NFE-6	AACCAAT
NEUCR$CPC1_02	242 (-)	0	100.00		CCAAT
HS$EG_07	242 (-)	0	100.00	CP1	CCAAT
HS$GP2B_13	242 (-)	0	100.00		CCAAT
MOUSE$BMG_10	242 (-)	0	100.00		CCAAT
HS$CYBH_01	242 (-)	0	100.00	CDP2, Clox, CP1, CUTL1, Cutl1	CCAAT
HBV$S_04	242 (-)	0	100.00		CCAAT
HS$FN_06	242 (-)	0	100.00		CCAAT
MOUSE$E2F1_02	242 (-)	0	100.00		CCAAT
HS$A24COL_01	242 (-)	0	100.00		CCAAT
MOUSE$EKLF_01	242 (-)	0	100.00	AP-1, GATA-1	CCAAT
HS$PTH_05	242 (-)	1	87.50	factor C complex	TGAACCTAT
AS$RAR_01	245 (+)	0	100.00	RAR-gamma, RXR-alpha	GGTTCA
MOUSE$RARB_01	245 (+)	0	100.00	RAR-beta, RXR-alpha, RXR-beta	GGTTCA
DROME$HSP27_05	245 (+)	0	100.00	FXR, RXR-alpha	GGTTCA
MOUSE$SPP1_01	245 (+)	0	100.00	RXR-alpha, VDR	GGTTCA
HS$P21WAF1_05	245 (+)	0	100.00	RXR-alpha, VDR	GGTTCA
HS$CABD9_01	245 (+)	0	100.00	RXR-alpha, VDR	GGTTCA
AS$SXR_02	245 (+)	0	100.00	RXR-alpha, SXR	GGTTCA
AS$SXR_03	245 (+)	0	100.00	RXR-alpha, SXR	GGTTCA
AS$SXR_04	245 (+)	0	100.00	RXR-alpha, SXR	GGTTCA
RAT$3KAT_01	245 (-)	0	100.00	PPAR-alpha, RXR-alpha	TGAACC
RAT$CYP24_02	245 (-)	0	100.00	VDR	TGAACC
AS$RAR_05	246 (+)	0	100.00	RAR-alpha1	GTTCA
AS$RAR_06	246 (+)	0	100.00	RAR-alpha1	GTTCA
AS$TR_06	246 (+)	0	100.00	T3R-alpha	GTTCA
AS$TR_07	246 (+)	0	100.00	T3R-alpha	GTTCA
AS$TR_08	246 (+)	0	100.00	T3R-alpha	GTTCA
AS$TR_11	246 (+)	0	100.00	T3R-alpha	GTTCA
MOUSE$RARA2_01	246 (+)	0	100.00	RAR-alpha1, RAR-beta2, RAR-gamma1	GTTCA
HS$ADH3_01	246 (-)	0	100.00	CAR, RAR-alpha1, RAR-beta, RXR-alpha	TGAAC
DROME$HSP23_01	247 (+)	0	100.00	EcR	TTCAATG
HS$CMYB_01	247 (-)	0	100.00	c-Myb	ATTGAA
HS$CDC2_10	247 (-)	0	100.00		TTGAA
HS$CYCA_06	247 (-)	0	100.00		TTGAA
HS$CDC25C_05	247 (-)	0	100.00		TTGAA
HS$EGFR_15	248 (+)	0	100.00		TCAAT
PSAM$U7SN_04	248 (-)	0	100.00		ATTGA
RAT$BCAS_05	250 (-)	1	88.89	C/EBPbeta, C/EBPdelta	ATTGGACAAT
HS$CRH_01	252 (-)	0	100.00	GR	TGGACA
HS$BAC_03	255 (+)	0	100.00	SRF	CCAAT
AMULV$AMULV_01	255 (+)	0	100.00		CCAAT
EBV$DSL_06	255 (+)	0	100.00		CCAAT
MOUSE$AAG_04	255 (+)	0	100.00	alpha-CP1, alpha-CP2a, alpha-CP2b, alpha-IRP	CCAAT
CHICK$BAG_06	255 (+)	0	100.00	TGGCA-binding protein	CCAAT
HS$GG_15	255 (+)	0	100.00	NF-E	CCAAT
HS$GG_16	255 (+)	0	100.00	CDP2, Clox, CUTL1, Cutl1	CCAAT
HS$GG_17	255 (+)	0	100.00	gammaCAAT	CCAAT
HS$GG_18	255 (+)	0	100.00	CP1	CCAAT
HS$ZG_07	255 (+)	0	100.00	CP2	CCAAT
HS$ZG_08	255 (+)	0	100.00	CP1	CCAAT
HS$HH1_03	255 (+)	0	100.00	H1TF2	CCAAT
MOUSE$M2IAB_01	255 (+)	0	100.00	PU.1	CCAAT
PIG$UPA_12	255 (+)	0	100.00	NF-1	CCAAT
RADLV$RLV_10	255 (+)	0	100.00		CCAAT
MULV$MULV_03	255 (+)	0	100.00		CCAAT
MOUSE$GLUT4_04	255 (+)	0	100.00		CCAAT
MOMLV$MOMULV_15	255 (+)	0	100.00		CCAAT
RAT$ALDB_02	255 (+)	0	100.00	CBF (2), CP2	CCAAT
HS$GG_20	255 (+)	0	100.00	CP1, NF-E3	CCAAT
RAT$TH2B_01	255 (+)	0	100.00		CCAAT
RAT$TH2B_02	255 (+)	0	100.00		CCAAT
HS$GG_21	255 (+)	0	100.00	CP1	CCAAT
MOUSE$LY6E_01	255 (+)	0	100.00		CCAAT
RAT$NEU_01	255 (+)	0	100.00		CCAAT
HS$GHA_10	255 (+)	0	100.00	alpha-CBF	CCAAT
NEUCR$CPC1_02	255 (+)	0	100.00		CCAAT
HS$EG_07	255 (+)	0	100.00	CP1	CCAAT

HS$EG_07	255 (+)	0	100.00	CP1	CCAAT
HS$GP2B_13	255 (+)	0	100.00		CCAAT
MOUSE$BMG_10	255 (+)	0	100.00		CCAAT
HS$CYBH_01	255 (+)	0	100.00	CDP2, Clox, CP1, CUTL1, Cutl1	CCAAT
HBV$S_04	255 (+)	0	100.00		CCAAT
HS$FN_06	255 (+)	0	100.00		CCAAT
MOUSE$E2F1_02	255 (+)	0	100.00		CCAAT
HS$A24COL_01	255 (+)	0	100.00		CCAAT
MOUSE$EKLF_01	255 (+)	0	100.00	AP-1, GATA-1	CCAAT
MOUSE$A11COL_01	255 (-)	0	100.00	CBF (2), IF2	ATTGG
MOUSE$A21COL_03	255 (-)	0	100.00	CCAAT-binding factor	ATTGG
MOUSE$A21COL_04	255 (-)	0	100.00	CBF (2), CBF-A, CBF-B	ATTGG
MOUSE$A21COL_05	255 (-)	0	100.00	EFI	ATTGG
AD2$E2L_03	255 (-)	0	100.00	CRF	ATTGG
HS$GHA_06	255 (-)	0	100.00	NF-1	ATTGG
SP$H2B1_02	255 (-)	0	100.00	CBF (1)	ATTGG
SP$H2B1_03	255 (-)	0	100.00	CDF	ATTGG
HS$HSP70_02	255 (-)	0	100.00	CTF	ATTGG
HS$HSP70_07	255 (-)	0	100.00	CBTF, CP1, CTF	ATTGG
MOUSE$M2EAK_07	255 (-)	0	100.00	NF-Y	ATTGG
MOUSE$M2EAK_08	255 (-)	0	100.00	NF-Y	ATTGG
RSV$RSV_05	255 (-)	0	100.00		ATTGG
HSV1$TK_04	255 (-)	0	100.00	C/EBPalpha	ATTGG
HSV1$TK_05	255 (-)	0	100.00	NF-Y'	ATTGG
AS$HAP23_01	255 (-)	0	100.00	HAP2, HAP3, HAP4	ATTGG
MOUSE$M1H2KB_12	255 (-)	0	100.00	EFI	ATTGG
RAT$TH2A_02	255 (-)	0	100.00		ATTGG
RAT$TH2A_04	255 (-)	0	100.00		ATTGG
HS$TK_02	255 (-)	0	100.00	CP1	ATTGG
MOUSE$A11COL_07	255 (-)	0	100.00	NF-1, Sp1	ATTGG
HS$CDC2_07	255 (-)	0	100.00	CBAF, CBF-B, CP1A	ATTGG
XENLA$GATA2_01	255 (-)	0	100.00	CBTF	ATTGG
RAT$NAT_05	255 (-)	0	100.00		ATTGG
HS$CDC2_12	255 (-)	0	100.00	CBAF	ATTGG
Y$MAL61_04	256 (-)	0	100.00	MIG1	AATTG
CHICK$LYS_08	258 (+)	1	88.89	GR	ATTCCTCTGT
CHICK$LYS_09	258 (+)	1	88.89	PR-alpha, PR-beta	ATTCCTCTGT
CHICK$LYS_12	258 (-)	1	90.00	GR	AACAGAGGAAT
I$HSF_01	258 (-)	0	90.00	HSF	AGAAN
F$HSF_01	258 (-)	0	90.00	HSF	AGAAN
MOUSE$RAS1_02	260 (+)	0	100.00	GR	TCTTCT
I$HSF_01	261 (-)	0	90.00	HSF	AGAAN
F$HSF_01	261 (-)	0	90.00	HSF	AGAAN
MOUSE$CDX2_01	265 (+)	0	100.00	FOXM1a, FOXM1b, HNF-3alpha, HNF-3B	TGTTT
RAT$FABPI_04	265 (+)	0	100.00	FOXM1a, FOXM1b, HNF-3alpha, HNF-3B	TGTTT
HS$ALP_02	265 (+)	0	100.00	HNF-3alpha, HNF-3B	TGTTT
MOUSE$ECADH_03	265 (-)	0	100.00	FOXM1a, FOXM1b, HNF-3alpha, HNF-3B	AAACA
AS$AREB6_44	266 (+)	0	100.00	AREB6	GTTTC
AS$AREB6_46	266 (+)	0	100.00	AREB6	GTTTC
AS$AREB6_48	266 (+)	0	100.00	AREB6	GTTTC
AS$AREB6_49	266 (+)	0	100.00	AREB6	GTTTC
AS$AREB6_50	266 (+)	0	100.00	AREB6	GTTTC
AS$AREB6_52	266 (+)	0	100.00	AREB6	GTTTC
AS$AREB6_53	266 (+)	0	100.00	AREB6	GTTTC
HS$IFI616_01	267 (-)	0	100.00	ISGF-3	GGAAA
MOUSE$IL5_02	267 (-)	0	100.00	NF-ATc, NF-ATp, NF-ATx	GGAAA
HS$IL4_01	267 (-)	0	100.00		GGAAA
MOUSE$IL4_06	267 (-)	0	100.00		GGAAA
MOUSE$IL4_07	267 (-)	0	100.00		GGAAA
MOUSE$IL4_08	267 (-)	0	100.00		GGAAA
HS$IL5_02	267 (-)	0	100.00		GGAAA
HS$IL13_01	267 (-)	0	100.00		GGAAA
MOUSE$CD40L_01	267 (-)	0	100.00		GGAAA
MOUSE$CD40L_02	267 (-)	0	100.00		GGAAA
MOUSE$CD40L_03	267 (-)	0	100.00		GGAAA
MOUSE$CD40L_04	267 (-)	0	100.00		GGAAA
HS$CD40L_01	267 (-)	0	100.00		GGAAA
HS$CD40L_02	267 (-)	0	100.00		GGAAA
HS$IFNG_03	267 (-)	0	100.00		GGAAA
HS$IFNG_04	267 (-)	0	100.00		GGAAA
HS$TNFA_05	267 (-)	0	100.00		GGAAA
MOUSE$MCP3_01	267 (-)	0	100.00		GGAAA
HS$TNFSF6_01	267 (-)	0	100.00		GGAAA
AS$STAT5A_52	268 (+)	1	87.50	STAT5A	TTCCGTGGA
AS$STAT5A_66	269 (+)	1	87.50	STAT5A	TTCCTGGAG
AS$STAT5A_67	269 (+)	1	87.50	STAT5A	TACCTGGAG
Y$ADH2_01	274 (-)	0	100.00	ADR1	TCTCC
HS$BG_28	275 (-)	1	87.50		CCTAATCTC
HS$GHA_03	276 (+)	0	100.00	GR, GR-alpha, GR-beta	AGATCA
AS$GR_06	276 (-)	0	100.00	GR	TGATCT
MOUSE$TTPA_01	277 (-)	0	100.00	AP-1	TGATC
AS$ZIC2_16	280 (+)	1	87.50	Zic2	TAGGTTGCC
HS$VEGF_02	280 (+)	0	100.00		CAGGT
HBV$HBVE_14	283 (+)	0	100.00	EF-C	GTTGC
HBV$HBVE_14	283 (-)	0	100.00	EF-C	GGCAAC
CHICK$BAG_03	285 (+)	0	100.00		TGCCC
MAIZE$PEPC_01	285 (+)	0	100.00	MNB1b, MNF1	TGCCCTT
MOUSE$CRABP2_01	285 (+)	0	100.00	RXR-alpha, RXR-gamma	TGCCCT
HS$CABD9_01	285 (+)	0	100.00	RXR-alpha, VDR	TGCCCT
RAT$VEGF_02	285 (+)	0	100.00	ER-alpha, ER-beta	TGCCC
CHICK$CYP2H1_05	285 (+)	0	100.00	CXR, RXR-gamma	TGCCCT
CHICK$BAG_03	285 (-)	0	100.00		GGGCA
RAT$NF1_01	285 (-)	0	100.00	LF-A1	GGGCA
AS$RAR_16	285 (-)	0	100.00	RAR-alpha1, RXR-alpha	AGGGCA
HS$CATHD_01	285 (-)	0	100.00	ER-alpha, Sp1	GGGCA
RAT$VEGF_01	285 (-)	0	100.00	ER-alpha, ER-beta	GGGCA
HS$ALDA_06	285 (-)	0	100.00	TR2-11	AGGGCA
HS$BAC_04	288 (+)	1	88.89	Elk-1, SAP-1a, SAP-1b, SRF	CCTTTTATGG
HS$VLC1_01	288 (+)	1	88.89	SRF, SRF (504 AA)	CCTTTTATGG
CHICK$AAC_10	288 (+)	1	88.89	SRF	CCTTCTTTGG
HS$PDGFA_01	288 (+)	1	88.89		CCTTTTATGG
AT$SUP_01	288 (+)	1	88.89	AG, AP1, AP3/PI	CCATTTTTGG
AS$HOXA3_08	288 (+)	1	87.50	HOXA3	CCTTTGTTG
HS$AAC_04	288 (-)	1	88.89	SRF, SRF (504 AA)	CCAAATAAGG
HS$AAC_05	288 (-)	1	88.89	SRF (504 AA)	CCAAATAAGG
HS$AAC_06	288 (-)	1	88.89	SRF (504 AA)	CCAAATAAGG
XENLA$AC_03	288 (-)	1	88.89	CArG box-binding protein, SRF	CCAAATAAGG
MOUSE$SRF_02	288 (-)	1	88.89	SRF (504 AA)	CCATAAAAGG
Y$CLB2_04	288 (-)	1	88.89	MCM1	TCAAAAAAGG
RAT$AMHC_04	289 (-)	1	87.50	aMEF-2, MEF-2 (516 AA)	CTAAAAAAG
VIV$VISNA_02	290 (+)	0	100.00		TTTTTTG
Y$SUC2_01	290 (+)	0	100.00	MIG1	TTTTT
AS$PF1_01	290 (+)	0	100.00	PF1	TTTTT
AS$PF1_02	290 (+)	0	100.00	PF1	TTTTT
Y$SUC2_01	291 (+)	0	100.00	MIG1	TTTTT
AS$STAT5A_61	291 (+)	1	87.50	STAT5A	TTCTTGGTA
AS$PF1_01	291 (+)	0	100.00	PF1	TTTTT
AS$PF1_02	291 (+)	0	100.00	PF1	TTTTT
AS$ZIC2_18	294 (+)	1	87.50	Zic2	TTGGTATTC
TO$E4_02	296 (-)	0	100.00		CTACC
YL$XPR2_01	300 (+)	0	100.00		GTCTC
DROME$EVE_09	302 (+)	0	100.00	GAGA factor	CTCTC
DROME$FTZ_32	302 (+)	0	100.00	GAGA factor	CTCTC
DROME$EVE_08	302 (-)	0	100.00	GAGA factor	GAGAG
DROME$EVE_13	302 (-)	0	100.00	GAGA factor	GAGAG
DROME$FTZ_32	302 (-)	0	100.00	GAGA factor	GAGAG
AS$GAGA_03	302 (-)	0	100.00	GAGA factor	GAGAG
Y$ADH2_01	303 (+)	0	100.00	ADR1	TCTCC
HS$BAC_03	306 (+)	0	100.00	SRF	CCAAT
AMULV$AMULV_01	306 (+)	0	100.00		CCAAT
EBV$DSL_06	306 (+)	0	100.00		CCAAT
MOUSE$AAG_04	306 (+)	0	100.00	alpha-CP1, alpha-CP2a, alpha-CP2b, alpha-IRP	CCAAT
CHICK$BAG_06	306 (+)	0	100.00	TGGCA-binding protein	CCAAT
HS$GG_15	306 (+)	0	100.00	NF-E	CCAAT
HS$GG_16	306 (+)	0	100.00	CDP2, Clox, CUTL1, Cutl1	CCAAT
HS$GG_17	306 (+)	0	100.00	gammaCAAT	CCAAT
HS$GG_18	306 (+)	0	100.00	CP1	CCAAT
HS$ZG_07	306 (+)	0	100.00	CP2	CCAAT
HS$ZG_08	306 (+)	0	100.00	CP1	CCAAT
HS$HH1_03	306 (+)	0	100.00	H1TF2	CCAAT
MOUSE$M2IAB_01	306 (+)	0	100.00	PU.1	CCAAT
PIG$UPA_12	306 (+)	0	100.00	NF-1	CCAAT
RADLV$RLV_10	306 (+)	0	100.00		CCAAT
MULV$MULV_03	306 (+)	0	100.00		CCAAT
MOUSE$GLUT4_04	306 (+)	0	100.00		CCAAT
MOMLV$MOMULV_15	306 (+)	0	100.00		CCAAT
RAT$ALDB_02	306 (+)	0	100.00	CBF (2), CP2	CCAAT
HS$GG_20	306 (+)	0	100.00	CP1, NF-E3	CCAAT
RAT$TH2B_01	306 (+)	0	100.00		CCAAT
RAT$TH2B_02	306 (+)	0	100.00		CCAAT
HS$GG_21	306 (+)	0	100.00	CP1	CCAAT
MOUSE$LY6E_01	306 (+)	0	100.00		CCAAT
RAT$NEU_01	306 (+)	0	100.00		CCAAT
HS$GHA_10	306 (+)	0	100.00	alpha-CBF	CCAAT
NEUCR$CPC1_02	306 (+)	0	100.00		CCAAT
HS$EG_07	306 (+)	0	100.00	CP1	CCAAT
HS$GP2B_13	306 (+)	0	100.00		CCAAT
MOUSE$BMG_10	306 (+)	0	100.00		CCAAT
HS$CYBH_01	306 (+)	0	100.00	CDP2, Clox, CP1, CUTL1, Cutl1	CCAAT
HBV$S_04	306 (+)	0	100.00		CCAAT
HS$FN_06	306 (+)	0	100.00		CCAAT
MOUSE$E2F1_02	306 (+)	0	100.00		CCAAT
HS$A24COL_01	306 (+)	0	100.00		CCAAT
MOUSE$EKLF_01	306 (+)	0	100.00	AP-1, GATA-1	CCAAT
MOUSE$A11COL_01	306 (-)	0	100.00	CBF (2), IF2	ATTGG
MOUSE$A21COL_03	306 (-)	0	100.00	CCAAT-binding factor	ATTGG
MOUSE$A21COL_04	306 (-)	0	100.00	CBF (2), CBF-A, CBF-B	ATTGG
MOUSE$A21COL_05	306 (-)	0	100.00	EFI	ATTGG
AD2$E2L_03	306 (-)	0	100.00	CRF	ATTGG
HS$GHA_06	306 (-)	0	100.00	NF-1	ATTGG
SP$H2B1_02	306 (-)	0	100.00	CBF (1)	ATTGG
SP$H2B1_03	306 (-)	0	100.00	CDF	ATTGG
HS$HSP70_02	306 (-)	0	100.00	CTF	ATTGG
HS$HSP70_07	306 (-)	0	100.00	CBTF, CP1, CTF	ATTGG
MOUSE$M2EAK_07	306 (-)	0	100.00	NF-Y	ATTGG
MOUSE$M2EAK_08	306 (-)	0	100.00	NF-Y	ATTGG
RSV$RSV_05	306 (-)	0	100.00		ATTGG
HSV1$TK_04	306 (-)	0	100.00	C/EBPalpha	ATTGG
HSV1$TK_05	306 (-)	0	100.00	NF-Y'	ATTGG
AS$HAP23_01	306 (-)	0	100.00	HAP2, HAP3, HAP4	ATTGG
MOUSE$M1H2KB_12	306 (-)	0	100.00	EFI	ATTGG
RAT$TH2A_02	306 (-)	0	100.00		ATTGG
RAT$TH2A_04	306 (-)	0	100.00		ATTGG
HS$TK_02	306 (-)	0	100.00	CP1	ATTGG
MOUSE$A11COL_07	306 (-)	0	100.00	NF-1, Sp1	ATTGG
HS$CDC2_07	306 (-)	0	100.00	CBAF, CBF-B, CP1A	ATTGG
XENLA$GATA2_01	306 (-)	0	100.00	CBTF	ATTGG
RAT$NAT_05	306 (-)	0	100.00		ATTGG
AS$NCX_29	306 (-)	0	100.00	Ncx	AGGGAATTGG
HS$CDC2_12	306 (-)	0	100.00	CBAF	ATTGG
Y$MAL61_04	307 (-)	0	100.00	MIG1	AATTG
RAT$ALAL_01	308 (-)	1	87.50	MAF	GAGGGAAGT
SHEEP$BLG_08	308 (-)	1	87.50	MAF	GAGGGAAGT
AS$IK_07	309 (-)	0	100.00	Ik-1, Ik-2	GGGAAT
AS$SPZ1_01	309 (-)	1	87.50	Spz1	GGAGGGTAT
AS$SPZ1_04	309 (-)	1	87.50	Spz1	GGAGGGTAT
AS$SPZ1_06	309 (-)	1	87.50	Spz1	GGAGGGAAA
AS$SPZ1_07	309 (-)	1	87.50	Spz1	GGAGGGAAC
AS$SPZ1_14	309 (-)	1	87.50	Spz1	GGAGGGTAT
AS$SPZ1_21	309 (-)	1	87.50	Spz1	GGAGGGATT
AS$SPZ1_25	309 (-)	1	87.50	Spz1	GGCGGGAAT
CTCF$CONS	312 (+)	0	100.00	CTCF	CCCTC
HS$DG_03	312 (+)	1	88.89		CCTTCCTTCC
HS$CMYC_18	312 (-)	1	87.50	MAZ, Sp1	GAGGGAGGG
HT1$HTLV1_08	313 (+)	0	100.00	c-Ets-1 68	CCTCC
HS$PFKM_03	313 (-)	1	88.89		GGGGAGGAGG
HS$AACS_02	313 (-)	0	100.00	ARP-1	AGGAGG
HS$GX_WT1_02	314 (+)	1	87.50	WT1 -KTS, WT1 I, WT1 I -KTS, WT1 I-del2, WT1-del2	CTCCCTCCC
Y$GLK1_01	314 (-)	0	100.00	GCR1	GAAGGAG
XENLA$RPL14_01	317 (+)	0	100.00	HrpF, XrpFI	CTTCC
Y$TPI_01	317 (+)	0	100.00	GCR1	CTTCC
MOUSE$RPL32_01	317 (-)	0	100.00	f(alpha)-f(epsilon), HrpF, XrpFI	GGAAG
HS$CDC2_06	317 (-)	0	100.00	c-Ets-2	GGGAAG
HS$TNFA_02	317 (-)	0	100.00		GGAAG
HS$TNFA_03	317 (-)	0	100.00		GGAAG
STAT$CONS_01	318 (+)	0	88.89	STAT1, STAT1alpha, STAT1beta, STAT2, STAT3, STAT4, STAT5, STAT5A, STAT5B, STAT6	TTCCCRKAA
AS$STAT_01	318 (+)	1	87.50	STAT6	TTCCGGGAA
AS$STAT_02	318 (+)	1	87.50	STAT6	TTCCGGGAA
AS$STAT_03	318 (+)	1	87.50	STAT6	TTCCCGTAA
AS$STAT_06	318 (+)	1	87.50	STAT6	TTCCCAGAA
AS$STAT_10	318 (+)	0	100.00	STAT6	TTCCCGGAA
AS$STAT_11	318 (+)	1	87.50	STAT6	TTCCCCGAA
AS$STAT_12	318 (+)	1	87.50	STAT6	TTCTCGGAA
AS$STAT_13	318 (+)	1	87.50	STAT6	TTCCCGTAA
AS$STAT_14	318 (+)	1	87.50	STAT6	TTCCCAGAA
AS$STAT5A_34	318 (+)	1	87.50	STAT5A	TTCCAGGAA
AS$STAT5A_38	318 (+)	1	87.50	STAT5A	TTCCGGGAA
AS$STAT5A_39	318 (+)	0	100.00	STAT5A	TTCCCGGAA
AS$STAT5A_41	318 (+)	1	87.50	STAT5A	TTCCCAGAA
AS$STAT5A_44	318 (+)	1	87.50	STAT5A	TTCCGGGAA
AS$STAT5A_45	318 (+)	1	87.50	STAT5A	TTCCTGGAA
AS$STAT5A_47	318 (+)	1	87.50	STAT5A	TTCCCCGAA
HS$P21WAF1_09	318 (+)	0	100.00	STAT1, STAT3	TTCCCGGAA
AS$STAT3_01	318 (+)	1	87.50	STAT3	TTCCCGTAA
V$STAT_01	318 (+)	0	88.89	STATx	TTCCCRKAA
AS$STAT_01	318 (-)	0	100.00	STAT6	TTCCGGGAA
AS$STAT_02	318 (-)	0	100.00	STAT6	TTCCGGGAA
AS$STAT_10	318 (-)	1	87.50	STAT6	TTCCCGGAA
AS$STAT5A_34	318 (-)	1	87.50	STAT5A	TTCCAGGAA
AS$STAT5A_35	318 (-)	1	87.50	STAT5A	TTCCGAGAA
AS$STAT5A_38	318 (-)	0	100.00	STAT5A	TTCCGGGAA
AS$STAT5A_39	318 (-)	1	87.50	STAT5A	TTCCCGGAA
AS$STAT5A_44	318 (-)	0	100.00	STAT5A	TTCCGGGAA
AS$STAT5A_45	318 (-)	1	87.50	STAT5A	TTCCTGGAA
AS$STAT5A_64	318 (-)	1	87.50	STAT5A	TTCCGGGAG
HS$P21WAF1_09	318 (-)	1	87.50	STAT1, STAT3	TTCCCGGAA
AS$ETS1_05	320 (+)	1	88.89	c-Ets-1	ACCGGAAGCA
AS$CETS1P54_02	320 (+)	1	88.89	c-Ets-1 54	ACCGGAAGCA
AS$ELK1_02	321 (+)	0	100.00	Elk-1, SRF	CCGGAA
AS$ELK1_03	321 (+)	0	100.00	Elk-1, SRF	CCGGAA
AS$ELK1_04	321 (+)	0	100.00	Elk-1, SRF	CCGGAA
AS$ELK1_05	321 (+)	0	100.00	Elk-1, SRF	CCGGAA
AS$ELK1_06	321 (+)	0	100.00	Elk-1, SRF	CCGGAA
AS$ELK1_08	321 (+)	0	100.00	Elk-1, SRF	CCGGAA
AS$ELK1_13	321 (+)	0	100.00	Elk-1, SAP-1a, SAP-1b, SRF	CCGGAA
ELK1$CONS_03	321 (+)	0	87.50		CCGGAARY
MOUSE$RPL30_02	321 (-)	1	87.50	f(alpha)-f(epsilon)	CGCTTCCGG
AS$ELK1_10	321 (-)	0	100.00	Elk-1, SRF	TTCCGG
E74A$CONS_01	322 (+)	0	90.00	E74A	MGGAA
MOUSE$RPL32_01	323 (+)	0	100.00	f(alpha)-f(epsilon), HrpF, XrpFI	GGAAG
HS$TNFA_02	323 (+)	0	100.00		GGAAG
HS$TNFA_03	323 (+)	0	100.00		GGAAG
XENLA$RPL14_01	323 (-)	0	100.00	HrpF, XrpFI	CTTCC
Y$TPI_01	323 (-)	0	100.00	GCR1	CTTCC
Y$FAS1_01	327 (+)	1	90.00	FBF1	GCATGTGAAAA
Y$FAS2_01	327 (-)	1	90.00	FBF1	TTTTCACATGC
MOUSE$IGH_10	328 (+)	0	100.00	muEBP-C2, TFE3-S	CATGTG
MOUSE$IGKL_10	328 (+)	0	100.00	muEBP-C2	CATGTG
HS$P53_01	328 (+)	0	100.00		CATGTG
HS$P53_02	328 (+)	0	100.00		CATGTG
RAT$FAS_03	328 (+)	0	100.00	SREBP-1, USF-1, USF2	CATGTG
MOUSE$TBX2_01	328 (+)	0	100.00	Mitf, USF-1	CATGTG
HS$CDC25A_03	328 (+)	0	100.00	c-Myc, Max	CATGTG
HS$CDC25A_04	328 (+)	0	100.00	c-Myc, Max	CATGTG
RAT$ADH1_01	328 (-)	0	100.00	USF-1	CACATG
AD$MLP_40	328 (-)	0	100.00	USF1	CACATG
CPF1$CONS	328 (-)	0	87.50	CBF1	RTCACRTG
Y$LPD1_01	328 (-)	0	100.00		GTCACATG
GFII$CONS	328 (-)	0	87.50	GFII	RTCACRTG
AS$CDE1_01	328 (-)	0	100.00	CBF1	GTCACATG
Y$INO1_02	329 (+)	1	87.50	INO2, NBF	ATGTGAAAA
HS$P53_03	331 (+)	0	100.00	Pax-2, Pax-5, Pax-8	GTGAC
HS$MMP1_06	331 (-)	0	100.00		GTCAC
PEA$IAA45_07	331 (-)	0	100.00	ARF1	TGTCAC
AS$MEIS1_04	332 (+)	0	100.00	Meis-1a, Meis-1b	TGACAA
POT$PR10a_01	332 (+)	0	100.00	PBF-1, PBF-2 (p24)	TGACA
AS$TGIF_01	332 (-)	0	100.00	TGIF	TGTCA
AS$TGIF_02	332 (-)	0	100.00	TGIF	TGTCA
AS$TGIF_03	332 (-)	0	100.00	TGIF	TGTCA
AS$TGIF_04	332 (-)	0	100.00	TGIF	TGTCA
AS$TGIF_05	332 (-)	0	100.00	TGIF	TGTCA
AS$TGIF_06	332 (-)	0	100.00	TGIF	TGTCA
AS$TGIF_07	332 (-)	0	100.00	TGIF	TGTCA
AS$TGIF_08	332 (-)	0	100.00	TGIF	TGTCA
AS$TGIF_10	332 (-)	0	100.00	TGIF	TGTCA
AS$TGIF_11	332 (-)	0	100.00	TGIF	TGTCA
AS$TGIF_12	332 (-)	0	100.00	TGIF	TGTCA
AS$TGIF_13	332 (-)	0	100.00	TGIF	TGTCA
AS$TGIF_14	332 (-)	0	100.00	TGIF	TGTCA
AS$TGIF_15	332 (-)	0	100.00	TGIF	TGTCA
POT$PR10a_01	332 (-)	0	100.00	PBF-1, PBF-2 (p24)	TGTCA
AS$FOXJ2_34	334 (+)	1	88.89	FOXJ2 (long isoform)	ACAATAAACA
MOUSE$BMG_01	336 (-)	0	100.00	GATA-1	TGATT
LPV$LPV_03	337 (+)	0	100.00	Pu box binding factor	ATCAA
HS$PR264_02	342 (+)	0	100.00	c-Myb	CAACTT
HS$PR264_06	342 (+)	0	100.00	c-Myb	CAACT
DROME$YP1_03	344 (-)	1	87.50	DSXF, DSXM	CTACAAAGT
SV$SV40_20	345 (+)	1	87.50	POU2F1	CTTTGCATA
SV$SV40_28	345 (+)	1	87.50	POU2F1	CTTTGCATA
XENLA$ENGRAILED2_01	345 (+)	0	100.00	LEF-1	CTTTGTA
MOUSE$ECADH_04	345 (+)	0	100.00	LEF-1	CTTTGTA
RAT$GHF1_01	351 (+)	0	100.00	PTF	ATACT
RAT$GHF1_01	351 (-)	0	100.00	PTF	AGTAT
AS$NKX3A_01	352 (-)	0	100.00	NKX3A	TAAGTA
AS$NKX3A_02	352 (-)	0	100.00	NKX3A	TAAGTA
AS$NKX3A_03	352 (-)	0	100.00	NKX3A	TAAGTA
AS$NKX3A_04	352 (-)	0	100.00	NKX3A	TAAGTA
AS$NKX3A_06	352 (-)	0	100.00	NKX3A	TAAGTA
AS$NKX3A_07	352 (-)	0	100.00	NKX3A	TAAGTA
AS$NKX3A_08	352 (-)	0	100.00	NKX3A	TAAGTA
AS$NKX3A_10	352 (-)	0	100.00	NKX3A	TAAGTA
AS$NKX3A_12	352 (-)	0	100.00	NKX3A	TAAGTA
AS$NKX3A_14	352 (-)	0	100.00	NKX3A	TAAGTA
AS$NKX3A_15	352 (-)	0	100.00	NKX3A	TAAGTA
AS$NKX3A_16	352 (-)	0	100.00	NKX3A	TAAGTA
AS$NKX3A_17	352 (-)	0	100.00	NKX3A	TAAGTA
AS$NKX3A_18	352 (-)	0	100.00	NKX3A	TAAGTA
HS$PR264_04	357 (-)	0	100.00	c-Myb	GAACTT
AS$PAX2_50	357 (-)	1	87.50	Pax-2.1, Pax-2.2	ACTGAACCT
AS$RAR_01	358 (+)	0	100.00	RAR-gamma, RXR-alpha	AGTTCA
RAT$CRBP2_01	358 (+)	0	100.00	COUP-TF1, PPAR-alpha, RAR-alpha1, RAR-beta, RXR-alpha, RXR-beta, TGIF	AGTTCA
MOUSE$CRABP2_02	358 (+)	0	100.00	RXR-alpha, RXR-gamma	AGTTCA
AS$RAR_08	358 (+)	0	100.00	RAR-alpha1, RXR-alpha	AGTTCA
MOUSE$RARB_01	358 (+)	0	100.00	RAR-beta, RXR-alpha, RXR-beta	AGTTCA
MMTV$MMTV_49	358 (+)	0	100.00	LXR-alpha, RXR-alpha, SXR	AGTTCA
RAT$CYP7A_05	358 (+)	0	100.00	FXR, LXR-alpha, LXR-beta, RXR-alpha	AGTTCA
AS$LXRAB_01	358 (+)	0	100.00	LXR-alpha, LXR-beta, RXR-alpha	AGTTCA
RAT$CYP3A1_01	358 (+)	0	100.00	PXR-1, PXR-2, RXR-alpha	AGTTCA
RAT$CYP3A2_01	358 (+)	0	100.00	PXR-1, PXR-2, RXR-alpha	AGTTCA
AS$SXR_02	358 (+)	0	100.00	RXR-alpha, SXR	AGTTCA
AS$SXR_03	358 (+)	0	100.00	RXR-alpha, SXR	AGTTCA
AS$SXR_04	358 (+)	0	100.00	RXR-alpha, SXR	AGTTCA
MOUSE$PTHR_01	358 (+)	0	100.00	COUP-TF1, RAR-alpha1, RXR-alpha	AGTTCA
RAT$POMC_05	358 (-)	0	100.00	GR	TGAACT
MOUSE$RVL3_02	358 (-)	0	100.00	RAR-beta, RAR-gamma, RXR-alpha	TGAACT
HS$CYP3A4_01	358 (-)	0	100.00	PXR-1, RXR-alpha, RXR-beta, SXR	TGAACT
HS$CYP3A4_04	358 (-)	0	100.00	PXR-1, RXR-alpha	TGAACT
CHICK$CYP2H1_05	358 (-)	0	100.00	CXR, RXR-gamma	TGAACT
AS$RAR_05	359 (+)	0	100.00	RAR-alpha1	GTTCA
AS$RAR_06	359 (+)	0	100.00	RAR-alpha1	GTTCA
AS$TR_06	359 (+)	0	100.00	T3R-alpha	GTTCA
AS$TR_07	359 (+)	0	100.00	T3R-alpha	GTTCA
AS$TR_08	359 (+)	0	100.00	T3R-alpha	GTTCA
AS$TR_11	359 (+)	0	100.00	T3R-alpha	GTTCA
MOUSE$RARA2_01	359 (+)	0	100.00	RAR-alpha1, RAR-beta2, RAR-gamma1	GTTCA
HS$ADH3_01	359 (-)	0	100.00	CAR, RAR-alpha1, RAR-beta, RXR-alpha	TGAAC
AS$STAT5A_52	360 (+)	1	87.50	STAT5A	TTCCGTGGA
AS$mEMBP_04	362 (-)	0	100.00	EmBP-1a	CACTG
EBV$IR4_05	364 (-)	0	100.00	R	GTCCAC
EBV$IR4_06	364 (-)	0	100.00	R	GTCCAC
CHICK$LYS_15	365 (-)	1	87.50	RXR-alpha, RXR-beta, T3R-alpha, T3R-beta1, v-ErbA	TGAGGTCAA
MOUSE$CRABP1_01	366 (+)	0	100.00	TR2-11	GGACCT
HS$HH4_02	366 (-)	0	100.00	H4TF-2	GGTCC
AS$TR_11	366 (-)	0	100.00	T3R-alpha	GGTCC
HS$EGFR_15	371 (+)	0	100.00		TCAAT
PSAM$U7SN_04	371 (-)	0	100.00		ATTGA
RAT$TH_03	371 (-)	0	100.00	ARIX	AATTGA
Y$MAL61_04	372 (-)	0	100.00	MIG1	AATTG
HS$INS_05	374 (-)	0	100.00		GGAAAT
XENLA$VITA2_03	374 (-)	1	87.50	C/EBPalpha, HNF-1, HNF-3alpha, HNF3(-like)	TGAGGTAAT
RAT$SPI_03	374 (-)	1	87.50	C/EBPalpha	TTAGGAAAT
RAT$SPI23_03	374 (-)	1	87.50	C/EBPalpha	TTAGGAAAT
Y$SUC2_05	374 (-)	0	100.00	MED8	AGGAAAT
HS$GMCSF_09	375 (+)	0	100.00	NF-ATp, NFAT-1	TTTCCTC
MOUSE$GMCSF_06	375 (+)	0	100.00	NFAT-1	TTTCCTC
HS$IFI616_01	375 (-)	0	100.00	ISGF-3	GGAAA
MOUSE$UPA_01	375 (-)	0	100.00	PEA3	AGGAAA
PEA3$CONS	375 (-)	0	91.67	c-Ets-1 54, c-Ets-1 68, c-Ets-2 58-64, PEA3	AGGAAR
MOUSE$IL5_02	375 (-)	0	100.00	NF-ATc, NF-ATp, NF-ATx	GGAAA
HS$IL4_01	375 (-)	0	100.00		GGAAA
MOUSE$IL4_06	375 (-)	0	100.00		GGAAA
MOUSE$IL4_07	375 (-)	0	100.00		GGAAA
MOUSE$IL4_08	375 (-)	0	100.00		GGAAA
HS$IL5_02	375 (-)	0	100.00		GGAAA
HS$IL13_01	375 (-)	0	100.00		GGAAA
MOUSE$CD40L_01	375 (-)	0	100.00		GGAAA
MOUSE$CD40L_02	375 (-)	0	100.00		GGAAA
MOUSE$CD40L_03	375 (-)	0	100.00		GGAAA
MOUSE$CD40L_04	375 (-)	0	100.00		GGAAA
HS$CD40L_01	375 (-)	0	100.00		GGAAA
HS$CD40L_02	375 (-)	0	100.00		GGAAA
HS$IFNG_03	375 (-)	0	100.00		GGAAA
HS$IFNG_04	375 (-)	0	100.00		GGAAA
HS$TNFA_05	375 (-)	0	100.00		GGAAA
MOUSE$MCP3_01	375 (-)	0	100.00		GGAAA
HS$TNFSF6_01	375 (-)	0	100.00		GGAAA
HS$ADH2_09	375 (-)	0	100.00	GR	AGGAAA
MOUSE$KROX_01	376 (+)	0	100.00	Elk-1, SAP-1a, SAP-1b, SRF	TTCCTC
MOUSE$FCGR3A_02	376 (+)	0	100.00	PU.1	TTCCTC
DROME$E74_10	376 (-)	0	100.00	E74A	AGGAA
DROME$E74_11	376 (-)	0	100.00	E74A	AGGAA
E74A$CONS_01	376 (-)	0	90.00	E74A	MGGAA
MOUSE$EPOR_03	379 (+)	1	87.50	GATA-1	CTTATCTGT
MOUSE$ACRG_01	381 (+)	0	100.00	MyoD	CATCTG
HS$CFOS_18	381 (+)	0	100.00	E12	CATCTG
HS$IGKL_11	381 (+)	0	100.00		CATCTG
RAT$MRF4_02	381 (+)	0	100.00	E12, myogenin	CATCTG
AS$TAL1_01	381 (-)	0	100.00	E47, ITF-2, Tal-1, Tal-1beta	CAGATG
HS$ADH2_04	383 (+)	1	88.89	C/EBPalpha	TCTGTGCAAT
RAT$SPI_01	384 (+)	1	87.50	C/EBPalpha	CTGAGAAAT
Y$INO1_01	384 (+)	1	87.50	NBF	ATGTGAAAT
AS$FOXJ2_30	388 (+)	1	88.89	FOXJ2 (long isoform)	GAAATAAACA
Y$GAL1_15	388 (-)	0	100.00	MIG1	TTATTTC
RAT$PL_07	389 (+)	0	100.00		AAATAAA
AS$TBP_03	389 (+)	0	100.00	TBP	AAATAAA
MOUSE$HOXA5_04	389 (+)	0	100.00		AAATAA
MOUSE$HOXA5_05	389 (+)	0	100.00		AAATAA
MOUSE$HOXA5_06	389 (+)	0	100.00		AAATAA
HS$MSH2_01	389 (-)	0	100.00	HNF-3alpha, HNF-3B	TATTT
HS$EG_08	391 (+)	0	100.00		ATAAA
HS$GFAP_01	391 (+)	0	100.00	TFIID	ATAAA
HS$GFAP_02	391 (+)	0	100.00	TBP	ATAAA
Y$PDC1_02	391 (-)	0	100.00	MIG1	TTTAT
Y$HAP4_01	391 (-)	0	100.00	MIG1	TTTAT
AS$FREAC2_01	392 (+)	0	100.00	FOXF2	TAAAC
HS$PR264_08	393 (+)	0	100.00	c-Myb	AAACGG
EBV$IR4_04	397 (+)	0	100.00	R	GGGAC
EBV$IR4_05	397 (+)	0	100.00	R	GGGAC
AS$DL_27	400 (+)	1	90.00	Dl	ACAGAAAAATC
DROME$TWI_05	401 (+)	1	88.89	Dl	CAGAAAAATC
RAT$SPI_01	401 (+)	1	87.50	C/EBPalpha	CTGAGAAAT
AS$PAX2_45	403 (+)	1	87.50	Pax-2.1, Pax-2.2	TAAAAATCA
NT$PR1A_03	403 (+)	0	100.00	GT-1	GAAAAA
Y$GAL4_01	403 (-)	0	100.00	MIG1	TTTTC
Y$SUC2_01	404 (-)	0	100.00	MIG1	TTTTT
AS$PF1_01	404 (-)	0	100.00	PF1	TTTTT
AS$PF1_02	404 (-)	0	100.00	PF1	TTTTT
Y$MAL61_03	405 (-)	0	100.00	MIG1	ATTTT
MOUSE$BMG_01	407 (-)	0	100.00	GATA-1	TGATT
MOUSE$IGH_55	408 (+)	0	100.00		ATCAT
ASPN$ABAA_03	410 (+)	0	100.00	abaA	CATTCT
ASPN$ABAA_04	410 (+)	0	100.00	abaA	CATTCT
ASPN$ABAA_05	410 (+)	0	100.00	abaA	CATTCT
ASPN$BRLA_03	410 (+)	0	100.00	abaA	CATTCT
ASPN$RODA_05	410 (+)	0	100.00	abaA	CATTCT
ABAA$CONS_01	410 (+)	0	91.67	abaA	CATTCY
ASPN$BRLA_05	410 (+)	0	100.00	abaA	CATTCT
ASPN$YA_02	410 (-)	0	100.00	abaA	AGAATG
I$HSF_01	411 (-)	0	90.00	HSF	AGAAN
F$HSF_01	411 (-)	0	90.00	HSF	AGAAN
AS$STAT5A_53	412 (-)	1	87.50	STAT5A	AGTCCAGAA
HS$GRH_01	415 (+)	0	100.00	AP-2, AP-2alphaA, AP-2alphaB, NF-1	TGGCC
HSV1$TK_12	415 (+)	0	100.00	RAR-alpha1, T3R-alpha	TGGCCT
HS$GRH_01	415 (-)	0	100.00	AP-2, AP-2alphaA, AP-2alphaB, NF-1	GGCCA
RAT$TAT_15	415 (-)	0	100.00	HNF-3alpha	GGCCA
SV$SV40_01	417 (+)	0	100.00	T-Ag	GCCTC
SV$SV40_02	417 (+)	0	100.00	T-Ag	GCCTC
SV$SV40_63	417 (+)	0	100.00	T-Ag	GCCTC
PA$PY_24	417 (-)	0	100.00	T-Ag	GAGGC
PA$PY_25	417 (-)	0	100.00	T-Ag	GAGGC
SV$SV40_02	417 (-)	0	100.00	T-Ag	GAGGC
XENLA$ACY_01	422 (+)	0	100.00	SRF	AAGAT
XENLA$ACY_01	422 (-)	0	100.00	SRF	ATCTT
FY$MFM1_02	426 (-)	1	87.50	mat1-Mc, ste11	AACAAAGAA
AS$HOXA3_08	427 (+)	1	87.50	HOXA3	CCTTTGTTG
MOUSE$LY49A_01	428 (+)	0	100.00	TCF-1(P)	CTTTGTT
XENLA$ENGRAILED2_03	428 (+)	0	100.00	LEF-1	CTTTGTT
HS$CD3E_01	428 (-)	0	100.00	LEF-1, mat1-Mc, Sox-13, Sox-5, SRY, TCF-1, TCF-1A, TCF-1B, TCF-1C, TCF-1E, TCF-1F, TCF-1G	AACAAAG
MOUSE$SRY_01	428 (-)	0	100.00	SRY	AACAAAG
HS$SRY_01	428 (-)	0	100.00	SRY	AACAAAG
HS$ADA_08	428 (-)	0	100.00	LEF-1	AACAAAG
AS$TCF1_01	428 (-)	0	100.00	TCF-1B	AACAAAG
AS$TCF1_02	428 (-)	0	100.00	TCF-1B	AACAAAG
AS$SOX15_02	428 (-)	0	100.00	Sox15	AACAAAG
Y$CYC1_09	432 (+)	0	100.00	hap2, HAP2, HAP3	GTTGG
Y$CYC1_10	432 (+)	0	100.00	CP1A, HAP2	GTTGG
Y$CYC1_11	432 (+)	0	100.00	HAP3, NF-YA	GTTGG
RAT$ANTEN_01	432 (-)	0	100.00	C/EBPalpha, C/EBPbeta	CCCAAC
MOUSE$IGLL_05	432 (-)	1	87.50	LyF-1	CATCCCAAC
RAT$LHB_03	432 (-)	1	87.50	Egr-1	CACCCCCAC
BPV1$BPV1_13	433 (+)	0	100.00	AP-2alphaA, AP-2alphaB	TTGGGG
Y$ACS1_02	433 (+)	0	100.00	ADR1	TTGGGG
BPV1$BPV1_13	433 (-)	0	100.00	AP-2alphaA, AP-2alphaB	CCCCAA
RAT$ME_01	434 (+)	0	100.00	T3R-alpha, T3R-beta1, T3R-beta2	TGGGGT
HS$AG_15	435 (+)	0	100.00	CAC-binding protein	GGGGTG
CHICK$PTH_01	436 (+)	0	100.00	RXR-alpha, VDR	GGGTGT
HS$BG_01	436 (-)	0	100.00	CACCC-binding factor	CACCC
HS$GG_13	436 (-)	0	100.00	gammaCAC1, gammaCAC2	CACCC
HS$GG_14	436 (-)	0	100.00	CACCC-binding factor, Sp1	CACCC
HS$GP2B_01	436 (-)	0	100.00	CACCC-binding factor	CACCC
RAT$TOA_02	436 (-)	0	100.00	CACCC-binding factor	CACCC
HS$EG_06	436 (-)	0	100.00	Sp1	CACCC
AS$CACCC_01	436 (-)	0	100.00	CACCC-binding factor	CACCC
HS$CYP24_01	436 (-)	0	100.00	VDR	ACACCC
MAIZE$C1_05	438 (+)	0	100.00		GTGTC
AS$TGIF_09	439 (+)	0	100.00	TGIF	TGTCT
CHICK$D1CR_03	443 (-)	0	100.00	deltaEF1	CACCTA
AS$ERR1_10	444 (+)	0	100.00	ERR1	AGGTGC
AS$ZIC3_31	445 (-)	1	87.50	Zic3	TGGGGCACC
RAT$A1I3_04	446 (+)	0	100.00		GTGCT
RAT$A1I3_04	446 (-)	0	100.00		AGCAC
RAT$VEGF_02	447 (-)	0	100.00	ER-alpha, ER-beta	GAGCA
AS$STAT5A_39	447 (-)	1	87.50	STAT5A	CCCGGAGCA
RAT$MLC_04	448 (-)	0	100.00	MAPF2, YY1	GGAGC
AS$STAT5A_67	449 (-)	1	87.50	STAT5A	TACCTGGAG
AS$AREB6_31	451 (-)	0	100.00	AREB6	GCACCTGG
AS$AREB6_35	451 (-)	0	100.00	AREB6	GCACCTGG
AS$AREB6_38	451 (-)	0	100.00	AREB6	GCACCTGG
MOUSE$CD4_03	452 (+)	0	100.00	E12, HEB	CAGGTG
DROME$HB_12	452 (+)	0	100.00	Ac, AS-C T3, Da, Sc	CAGGTG
DROME$AC_01	452 (+)	0	100.00	Ac, AS-C T3, Da, Sc	CAGGTG
AS$SNAIL_01	452 (+)	0	100.00	Sn	CAGGTG
AS$LMO2_01	452 (+)	0	100.00	CLIM2, E12, E47, GATA-1, Lmo2, Tal-1	CAGGTG
HS$VEGF_02	452 (+)	0	100.00		CAGGT
MOUSE$ACRD_02	452 (-)	0	100.00	E12, E47, MyoD, myogenin	CACCTG
RADLV$RLV_07	452 (-)	0	100.00	Rad-1	GCACCTG
XENLA$AC_05	452 (-)	0	100.00	EMF1, MyoD	CACCTG
HS$CXCR4_02	452 (-)	0	100.00	c-Myc, USF2	CACCTG
PV$PHASL_02	452 (-)	0	100.00	CAN	CACCTG
AS$ERR1_10	453 (+)	0	100.00	ERR1	AGGTGC
RAT$A1I3_04	455 (+)	0	100.00		GTGCT
RAT$A1I3_04	455 (-)	0	100.00		AGCAC
I$HSF_01	458 (-)	0	90.00	HSF	AGAAN
F$HSF_01	458 (-)	0	90.00	HSF	AGAAN
AS$STAT5A_64	459 (+)	1	87.50	STAT5A	TTCCGGGAG
AS$STAT_06	459 (-)	1	87.50	STAT6	TTCCCAGAA
AS$STAT_14	459 (-)	1	87.50	STAT6	TTCCCAGAA
AS$STAT5A_41	459 (-)	1	87.50	STAT5A	TTCCCAGAA
MOUSE$TDT_02	460 (+)	1	87.50	LyF-1	TTTGGGAGA
MOUSE$IGLL_02	460 (+)	1	87.50	LyF-1	TCAGGGAGA
MOUSE$IGLL_06	460 (+)	1	87.50	LyF-1	TTTGGGAGA
AS$LYF1_01	460 (+)	1	87.50	LyF-1	TTTGGGAGA
AS$LYF1_04	460 (+)	1	87.50	LyF-1	TTTGGGAGA
AS$LYF1_06	460 (+)	1	87.50	LyF-1	TCTGGGAGG
AS$LYF1_07	460 (+)	1	87.50	LyF-1	CCTGGGAGA
MOUSE$IGLL_03	460 (-)	1	87.50	LyF-1	CCTCCCAGA
HS$LCK_01	460 (-)	1	87.50	LyF-1	TCTCCCAGG
SV$SV40_37	461 (+)	0	100.00		CTGGG
RAT$AMGL_02	461 (+)	0	100.00	IL-6 RE-BP, STAT1, STAT5A, STAT5B, STAT6	CTGGGA
HS$APOB_10	462 (+)	0	100.00	AP-2alphaA, AP-2alphaB	TGGGAG
HS$CDH1_01	462 (+)	0	100.00	LUN-1	TGGGA
HS$TLN_01	462 (+)	0	100.00	LUN-1	TGGGA
HS$A11COL_07	462 (-)	0	100.00		TCTCCCA
HS$CDH1_01	462 (-)	0	100.00	LUN-1	TCCCA
HS$TLN_01	462 (-)	0	100.00	LUN-1	TCCCA
AS$LYF1_05	463 (+)	0	100.00	LyF-1	GGGAGA
CHICK$ITGB3_01	463 (+)	0	100.00	RXR-beta, VDR	GGGAGA
Y$ADH2_01	464 (-)	0	100.00	ADR1	TCTCC
DROME$EVE_08	465 (+)	0	100.00	GAGA factor	GAGAG
DROME$EVE_13	465 (+)	0	100.00	GAGA factor	GAGAG
DROME$FTZ_32	465 (+)	0	100.00	GAGA factor	GAGAG
AS$GAGA_03	465 (+)	0	100.00	GAGA factor	GAGAG
DROME$EVE_09	465 (-)	0	100.00	GAGA factor	CTCTC
DROME$FTZ_32	465 (-)	0	100.00	GAGA factor	CTCTC
PA$PY_12	466 (+)	0	100.00		AGAGG
AS$AREB6_28	466 (-)	0	100.00	AREB6	TCACCTCT
HS$RARG_01	468 (+)	0	100.00	RAR-alpha1, RAR-beta, RAR-gamma, RXR-alpha	AGGTGA
HSV1$TK_12	468 (+)	0	100.00	RAR-alpha1, T3R-alpha	AGGTGA
MOUSE$IBABP_01	468 (+)	0	100.00	FXR, RXR-alpha	AGGTGA
AS$ERR1_16	468 (+)	0	100.00	ERR1	AGGTGA
RAT$ME_08	468 (+)	0	100.00	PPAR-gamma, RXR-gamma	AGGTGA
HS$CAMHC_01	468 (-)	0	100.00	Hp55, Hp65, p58, RAR-alpha1, RXR-beta, T3R-alpha, T3R-beta1, T3R-beta2	TCACCT
HS$CYP24_02	468 (-)	0	100.00	VDR	TCACCT
RAT$CYP24_01	468 (-)	0	100.00	VDR	TCACCT
HS$APOA2_06	469 (-)	0	100.00		TCACC
MOUSE$AFEP_13	470 (+)	0	100.00	NP-III	GTGACC
HS$P53_03	470 (+)	0	100.00	Pax-2, Pax-5, Pax-8	GTGAC
HS$MMP1_06	470 (-)	0	100.00		GTCAC
XENLA$VITA2_02	471 (+)	0	100.00	ER-alpha, ER-beta1, NHP-1, T3R-alpha1, T3R-beta1	TGACCT
HS$ADH3_01	471 (+)	0	100.00	CAR, RAR-alpha1, RAR-beta, RXR-alpha	TGACC
AS$GR_12	471 (+)	0	100.00	GR-alpha, GR-beta	TGACCT
CHICK$OA_12	471 (+)	0	100.00	ER-alpha	TGACCT
CHICK$OA_13	471 (+)	0	100.00	ER-alpha	TGACCT
CHICK$OA_14	471 (+)	0	100.00	ER-alpha	TGACC
CHICK$OA_15	471 (+)	0	100.00	ER-alpha	TGACCT
MOUSE$CRABP2_01	471 (+)	0	100.00	RXR-alpha, RXR-gamma	TGACCT
AS$TR_16	471 (+)	0	100.00	T3R-beta1	TGACCT
AS$TR_17	471 (+)	0	100.00	T3R-beta1	TGACCT
AS$TR_18	471 (+)	0	100.00	RXR-alpha, T3R-beta1	TGACCT
RAT$AOX_01	471 (+)	0	100.00	PPAR-alpha, RXR-alpha	TGACCT
AS$ATFCEBPB_01	471 (+)	1	87.50	c-Fos, c-Jun, C/EBPbeta	TGACGTAGT
BOVIN$OT_01	471 (+)	0	100.00	SF-1	TGACCT
AS$SF1_01	471 (+)	0	100.00	SF-1	TGACCT
AS$FXR_01	471 (+)	0	100.00	FXR, RXR-alpha	TGACCT
HS$SHP1_01	471 (+)	0	100.00	FXR, RXR-alpha	TGACCT
MOUSE$CRABP1_01	471 (+)	0	100.00	TR2-11	TGACCT
RAT$LHB_06	471 (+)	1	87.50	SF-1	TGACCTTGT
CHICK$OA_02	471 (-)	0	100.00	ARP-1, COUP, COUP-TF1, PPAR-alpha, RXR-alpha	AGGTCA
CHICK$OA_03	471 (-)	0	100.00	ER-alpha	GGTCA
CHICK$OA_04	471 (-)	0	100.00	c-Fos, c-Jun, ER-alpha	GGTCA
XENLA$VITA2_02	471 (-)	0	100.00	ER-alpha, ER-beta1, NHP-1, T3R-alpha1, T3R-beta1	AGGTCA
PPAR$CONS_01	471 (-)	0	100.00	PPAR-alpha	AGGTCA
AS$GR_12	471 (-)	0	100.00	GR-alpha, GR-beta	AGGTCA
RAT$CRBP2_01	471 (-)	0	100.00	COUP-TF1, PPAR-alpha, RAR-alpha1, RAR-beta, RXR-alpha, RXR-beta, TGIF	AGGTCA
RAT$CRBP1_01	471 (-)	0	100.00	COUP-TF1, RAR-beta, RXR-alpha	AGGTCA
AS$RAR_02	471 (-)	0	100.00	RAR-beta, RXR-alpha	AGGTCA
MOUSE$CRABP2_02	471 (-)	0	100.00	RXR-alpha, RXR-gamma	AGGTCA
AS$RXR_01	471 (-)	0	100.00	COUP-TF1, PPAR-alpha, RXR-alpha	AGGTCA
AS$RXR_02	471 (-)	0	100.00	COUP-TF1, RXR-alpha	AGGTCA
AS$RAR_03	471 (-)	0	100.00	RAR-alpha1, RXR-beta, T3R-beta1	AGGTCA
AS$RAR_04	471 (-)	0	100.00	RAR-alpha1, T3R-alpha	GGTCA
AS$RAR_06	471 (-)	0	100.00	RAR-alpha1	GGTCA
AS$TR_08	471 (-)	0	100.00	T3R-alpha	GGTCA
AS$TR_09	471 (-)	0	100.00	RAR-alpha1, T3R-alpha	GGTCA
AS$TR_10	471 (-)	0	100.00	T3R-alpha	GGTCA
AS$NGFIB_01	471 (-)	0	100.00	NGFI-B	AGGTCA
MOUSE$CRBP1_01	471 (-)	0	100.00	RAR-alpha1, RAR-beta2, RAR-gamma1	AGGTCA
AS$RAR_07	471 (-)	0	100.00	RAR-alpha1, RXR-alpha	AGGTCA
AS$RAR_08	471 (-)	0	100.00	RAR-alpha1, RXR-alpha	AGGTCA
AS$RAR_09	471 (-)	0	100.00	RAR-alpha1, RXR-alpha	AGGTCA
AS$RAR_10	471 (-)	0	100.00	RAR-alpha1, RXR-alpha	AGGTCA
AS$RAR_11	471 (-)	0	100.00	RAR-alpha1, RXR-alpha	AGGTCA
AS$RAR_12	471 (-)	0	100.00	RAR-alpha1, RXR-alpha	AGGTCA
AS$RAR_13	471 (-)	0	100.00	RAR-alpha1, RXR-alpha	AGGTCA
AS$RAR_14	471 (-)	0	100.00	RAR-alpha1, RXR-alpha	AGGTCA
AS$RAR_15	471 (-)	0	100.00	RAR-alpha1, RXR-alpha	AGGTCA
AS$RAR_16	471 (-)	0	100.00	RAR-alpha1, RXR-alpha	AGGTCA
AS$RAR_17	471 (-)	0	100.00	RAR-alpha1, RXR-alpha	AGGTCA
AS$RAR_18	471 (-)	0	100.00	RAR-alpha1, RXR-alpha	AGGTCA
AS$RAR_21	471 (-)	0	100.00	RAR-alpha1, RXR-alpha	AGGTCA
AS$TR_15	471 (-)	0	100.00	RXR-alpha, T3R-beta1	AGGTCA
AS$RAR_23	471 (-)	0	100.00	RAR-alpha1, RXR-alpha	AGGTCA
AS$TR_16	471 (-)	0	100.00	T3R-beta1	AGGTCA
AS$TR_17	471 (-)	0	100.00	T3R-beta1	AGGTCA
AS$TR_18	471 (-)	0	100.00	RXR-alpha, T3R-beta1	AGGTCA
AS$RXR_03	471 (-)	0	100.00	RXR-alpha	AGGTCA
MOUSE$PCP2_01	471 (-)	0	100.00	RXR-beta, T3R-alpha1, T3R-beta1	AGGTCA
AS$RXR_04	471 (-)	0	100.00	ARP-1, RXR-alpha	AGGTCA
AS$RXR_05	471 (-)	0	100.00	ARP-1, RAR-alpha1, RXR-alpha	AGGTCA
AS$RXR_6	471 (-)	0	100.00	RXR-alpha, TAF(II)28	AGGTCA
AS$TR_20	471 (-)	0	100.00	RAR-beta, RXR-alpha, T3R-alpha1, T3R-beta1, T3R-beta2	AGGTCA
AS$TR_34	471 (-)	0	100.00	T3R-alpha1, T3R-alpha2	AGGTCA
MOUSE$MYOD_04	471 (-)	0	100.00	ARP-1, RXR-alpha, RXR-gamma, T3R-alpha	AGGTCA

				T3R-alpha	
NT$CHN50_01	471 (-)	0	100.00	WRKY1, WRKY3, WRKY4	GGTCA
AS$LXRA_01	471 (-)	0	100.00	LXR-alpha, RXR-alpha	AGGTCA
RAT$FAS_04	471 (-)	0	100.00	LXR-alpha, RXR-alpha	AGGTCA
AS$LXRB_01	471 (-)	0	100.00	LXR-beta, RXR-alpha	AGGTCA
AS$RXR_08	471 (-)	0	100.00	RXR-alpha	AGGTCA
AS$LXRB_02	471 (-)	0	100.00	LXR-beta, RXR-alpha	AGGTCA
AS$FXR_01	471 (-)	0	100.00	FXR, RXR-alpha	AGGTCA
AS$VDR_01	471 (-)	0	100.00	RXR-alpha, VDR	AGGTCA
AS$GCNF_02	471 (-)	0	100.00	GCNF	AGGTCA
AS$GCNF_03	471 (-)	0	100.00	GCNF	AGGTCA
AS$GCNF_05	471 (-)	0	100.00	GCNF	AGGTCA
AS$SXR_01	471 (-)	0	100.00	RXR-alpha, SXR	AGGTCA
HS$CYP3A4_01	471 (-)	0	100.00	PXR-1, RXR-alpha, RXR-beta, SXR	AGGTCA
AS$BXR2_01	471 (-)	0	100.00	BXR-beta	AGGTCA
AS$BXR2_02	471 (-)	0	100.00	BXR-beta	AGGTCA
AS$ERR1_03	471 (-)	0	100.00	ERR1	AGGTCA
AS$ERR1_04	471 (-)	0	100.00	ERR1	AGGTCA
AS$ERR1_05	471 (-)	0	100.00	ERR1	AGGTCA
AS$ERR1_07	471 (-)	0	100.00	ERR1	AGGTCA
AS$ERR1_08	471 (-)	0	100.00	ERR1	AGGTCA
AS$ERR1_09	471 (-)	0	100.00	ERR1	AGGTCA
AS$ERR1_11	471 (-)	0	100.00	ERR1	AGGTCA
AS$ERR1_13	471 (-)	0	100.00	ERR1	AGGTCA
AS$ERR1_15	471 (-)	0	100.00	ERR1	AGGTCA
AS$ERR1_17	471 (-)	0	100.00	ERR1	AGGTCA
AS$ERR1_20	471 (-)	0	100.00	ERR1	AGGTCA
AS$ERR1_21	471 (-)	0	100.00	ERR1	AGGTCA
AS$ERR1_22	471 (-)	0	100.00	ERR1	AGGTCA
AS$ERR1_24	471 (-)	0	100.00	ERR1	AGGTCA
AS$ERR1_25	471 (-)	0	100.00	ERR1	AGGTCA
AS$TR_35	471 (-)	0	100.00	T3R-alpha	AGGTCA
AS$REVERBA_01	471 (-)	0	100.00	REVERB-alpha	AGGTCA
AS$REVERBA_02	471 (-)	0	100.00	REVERB-alpha	AGGTCA
AS$REVERBA_03	471 (-)	0	100.00	REVERB-alpha	AGGTCA
AS$REVERBA_04	471 (-)	0	100.00	REVERB-alpha	AGGTCA
AS$REVERBA_05	471 (-)	0	100.00	REVERB-alpha	AGGTCA
MOUSE$CRBP1_02	471 (-)	0	100.00	REVERB-alpha	AGGTCA
MOUSE$PTHR_01	471 (-)	0	100.00	COUP-TF1, RAR-alpha1, RXR-alpha	AGGTCA
RAT$CYP3A23_01	471 (-)	0	100.00	PXR-1	AGGTCA
MOUSE$AQP7_01	471 (-)	0	100.00	PPAR-alpha, PPAR-gamma, RXR-alpha	AGGTCA
DROME$ADH_34	472 (-)	1	87.50	FTZ-F1	CACAAGGTC
MOUSE$HOXB1_01	478 (+)	1	87.50	Exd, HOXA1, HOXB1, Pbx-1a, Pbx-1b, PKNOX1	GTGATGGAT
MOUSE$PBGD_01	478 (-)	0	100.00	CAC-binding protein	CCCTCAC
CTCF$CONS	480 (-)	0	100.00	CTCF	CCCTC
HS$GHA_07	483 (-)	0	100.00	GR, GR-alpha, GR-beta	TGATCC
MOUSE$TTPA_01	484 (-)	0	100.00	AP-1	TGATC
DROME$ESPL_01	487 (+)	1	87.50	RBP-Jkappa	CTGTGGGAA
AS$mEMBP_04	487 (-)	0	100.00	EmBP-1a	CACTG
RAT$CYTOPB_02	489 (-)	0	100.00	RBP-Jkappa	TTCCCAC
AS$IK_01	490 (+)	0	100.00	Ik-1, Ik-2, Ik-3	TGGGAAT
AS$IK_02	490 (+)	0	100.00	Ik-1, Ik-2, Ik-3, Ik-4	TGGGAAT
AS$IK_03	490 (+)	0	100.00	Ik-1, Ik-2, Ik-3	TGGGAAT
AS$IK_04	490 (+)	0	100.00	Ik-1, Ik-2, Ik-3, Ik-4	TGGGAAT
AS$IK_06	490 (+)	0	100.00	Ik-1, Ik-2	TGGGAA
HS$CDH1_01	490 (+)	0	100.00	LUN-1	TGGGA
HS$TLN_01	490 (+)	0	100.00	LUN-1	TGGGA
HS$CDH1_01	490 (-)	0	100.00	LUN-1	TCCCA
HS$TLN_01	490 (-)	0	100.00	LUN-1	TCCCA
AS$IK_07	491 (+)	0	100.00	Ik-1, Ik-2	GGGAAT
Y$GAL3_01	493 (-)	0	100.00	MIG1	TATTC
PA$PY_12	497 (+)	0	100.00		AGAGG
AS$AREB6_28	497 (-)	0	100.00	AREB6	TCACCTCT
HS$RARG_01	499 (+)	0	100.00	RAR-alpha1, RAR-beta, RAR-gamma, RXR-alpha	AGGTGA
HSV1$TK_12	499 (+)	0	100.00	RAR-alpha1, T3R-alpha	AGGTGA
MOUSE$IBABP_01	499 (+)	0	100.00	FXR, RXR-alpha	AGGTGA
AS$ERR1_16	499 (+)	0	100.00	ERR1	AGGTGA
RAT$ME_08	499 (+)	0	100.00	PPAR-gamma, RXR-gamma	AGGTGA
HS$CAMHC_01	499 (-)	0	100.00	Hp55, Hp65, p58, RAR-alpha1, RXR-beta, T3R-alpha, T3R-beta1, T3R-beta2	TCACCT
HS$CYP24_02	499 (-)	0	100.00	VDR	TCACCT
RAT$CYP24_01	499 (-)	0	100.00	VDR	TCACCT
HS$APOA2_06	500 (-)	0	100.00		TCACC
NEUCR$NIT3_01	501 (-)	0	100.00	NIT2	TATCAC
HS$ADH2_10	501 (-)	0	100.00	GR	TATCAC
MOUSE$AAG_01	502 (+)	0	100.00	GATA-1, GATA-2, GATA-3	TGATA
MOUSE$AAG_06	502 (+)	0	100.00	GATA-1	TGATA
DBP$CONS_01	502 (+)	1	87.50	DBP	TGATTTTGT
MOUSE$BMG_05	502 (-)	0	100.00	factor B2, GATA-1	TATCA
AS$MRF2_08	504 (-)	0	100.00	MRF-2	CACAATAT
AS$MRF2_09	504 (-)	0	100.00	MRF-2	CACAATAT
HS$IL3_08	508 (+)	0	100.00	AML1, AML1a, AML1c	TGTGGG
Y$MEL1_02	509 (+)	0	100.00	MIG1	GTGGGG
Y$MAL61_03	509 (+)	0	100.00	MIG1	GTGGGG
Y$MAL61_04	509 (+)	0	100.00	MIG1	GTGGGG
PA$PY_23	511 (+)	0	100.00	T-Ag	GGGGC
SV$SV40_04	511 (+)	0	100.00	T-Ag	GGGGC
AS$NFKAPPAB_18	511 (+)	1	88.89	NF-kappaB	GGGGCTTTCC
AS$NFKAPPAB_38	511 (+)	1	88.89	NF-kappaB	GGGGCTTTCC
SV$SV40_63	511 (+)	0	100.00	T-Ag	GGGGC
PA$PY_26	511 (-)	0	100.00	T-Ag	GCCCC
MAIZE$ADH11S_06	511 (-)	0	100.00	GCBP-1, Sp1	GCCCC
Y$GAL4_01	516 (+)	0	100.00	MIG1	TTTTC
AS$STAT5A_45	517 (+)	1	87.50	STAT5A	TTCCTGGAA
HS$TNFA_04	517 (-)	0	100.00		AGAAA
AS$STAT5A_34	517 (-)	1	87.50	STAT5A	TTCCAGGAA
I$HSF_01	517 (-)	0	90.00	HSF	AGAAN
F$HSF_01	517 (-)	0	90.00	HSF	AGAAN
AS$STAT_06	518 (-)	1	87.50	STAT6	TTCCCAGAA
AS$STAT_14	518 (-)	1	87.50	STAT6	TTCCCAGAA
AS$STAT5A_41	518 (-)	1	87.50	STAT5A	TTCCCAGAA
CHICK$LYS_19	519 (+)	1	87.50	AP-3, C/EBPalpha	TTTGGAAAT
RAT$AMGL_01	520 (+)	0	100.00	IL-6 RE-BP	CTGGAA
HAPF1$CONS	520 (+)	0	92.86	C/EBPbeta	CTGGRAA
AS$MSX1_02	521 (+)	1	87.50	Msx-1	TGGTAATTG
HS$IGKL_01	521 (-)	0	100.00		TTTCCA
HS$IFI616_01	522 (+)	0	100.00	ISGF-3	GGAAA
HS$INS_05	522 (+)	0	100.00		GGAAAT
MOUSE$IL5_02	522 (+)	0	100.00	NF-ATc, NF-ATp, NF-ATx	GGAAA
HS$IL4_01	522 (+)	0	100.00		GGAAA
MOUSE$IL4_06	522 (+)	0	100.00		GGAAA
MOUSE$IL4_07	522 (+)	0	100.00		GGAAA
MOUSE$IL4_08	522 (+)	0	100.00		GGAAA
HS$IL5_02	522 (+)	0	100.00		GGAAA
HS$IL13_01	522 (+)	0	100.00		GGAAA
MOUSE$CD40L_01	522 (+)	0	100.00		GGAAA
MOUSE$CD40L_02	522 (+)	0	100.00		GGAAA
MOUSE$CD40L_03	522 (+)	0	100.00		GGAAA
MOUSE$CD40L_04	522 (+)	0	100.00		GGAAA
HS$CD40L_01	522 (+)	0	100.00		GGAAA
HS$CD40L_02	522 (+)	0	100.00		GGAAA
HS$IFNG_03	522 (+)	0	100.00		GGAAA
HS$IFNG_04	522 (+)	0	100.00		GGAAA
HS$TNFA_05	522 (+)	0	100.00		GGAAA
MOUSE$MCP3_01	522 (+)	0	100.00		GGAAA
HS$TNFSF6_01	522 (+)	0	100.00		GGAAA
RAT$CYTOPB_03	522 (-)	1	87.50	NF-kappaB	GAAATTTCC
CHICK$LYS_20	525 (+)	1	88.89	AP-3, C/EBPalpha, POU2F1	AATTGCAAAG
Y$MAL61_04	525 (+)	0	100.00	MIG1	AATTG
CHICK$ACRA_06	526 (-)	0	100.00		GCAAT
RSV$RSV_04	526 (-)	0	100.00	C/EBP, C/EBPalpha, C/EBPbeta, C/EBPbeta(p20), C/EBPbeta(p34), C/EBPgamma, EFII	GCAAT
HS$ADH2_04	526 (-)	1	88.89	C/EBPalpha	TCTGTGCAAT
RAT$POMC_03	530 (+)	0	100.00	GR	CAGAG
DROME$EVE_08	532 (+)	0	100.00	GAGA factor	GAGAG
DROME$EVE_13	532 (+)	0	100.00	GAGA factor	GAGAG
DROME$FTZ_32	532 (+)	0	100.00	GAGA factor	GAGAG
AS$GAGA_03	532 (+)	0	100.00	GAGA factor	GAGAG
DROME$EVE_09	532 (-)	0	100.00	GAGA factor	CTCTC
DROME$FTZ_32	532 (-)	0	100.00	GAGA factor	CTCTC
PA$PY_12	533 (+)	0	100.00		AGAGG
AS$AREB6_04	533 (-)	1	87.50	AREB6	TGCACCTGT
AS$ERR1_10	535 (+)	0	100.00	ERR1	AGGTGC
MOUSE$MT1_01	537 (-)	0	100.00	Sp1	TGCAC
AS$HB_03	544 (-)	1	88.89	Hb	TCATAAAAAC
Y$SUC2_01	545 (+)	0	100.00	MIG1	TTTTT
AS$PF1_01	545 (+)	0	100.00	PF1	TTTTT
AS$PF1_02	545 (+)	0	100.00	PF1	TTTTT
Y$MAL2R_01	546 (+)	0	100.00	MIG1	TTTTA
AS$FOXJ1_04	546 (+)	0	100.00	FOXJ1	TTTTA
DROME$AC5C_01	546 (-)	0	100.00	B factor, TBP	TATAAAA
HS$HSP70_11	546 (-)	0	100.00	TBP, TFIID	TATAAAA
AD$MLP_11	546 (-)	0	100.00	TFIIA, TFIIA-alpha/beta precursor (major), TFIIA-alpha/beta precursor (minor), TFIIA-gamma, TFIIB, TFIID, TFIIE, TFIIF, TFIIF-alpha, TFIIF-beta	TATAAAA
AD$MLP_12	546 (-)	0	100.00		TATAAAA
AD$MLP_13	546 (-)	0	100.00	En, TBP, TFIID	TATAAAA
AD$MLP_14	546 (-)	0	100.00	TFIID	TATAAAA
AD$MLP_15	546 (-)	0	100.00		TATAAAA
AD$MLP_16	546 (-)	0	100.00	TBP, TBP-1, TRF	TATAAAA
AD$MLP_36	546 (-)	0	100.00	TFIID	TATAAAA
AD$MLP_39	546 (-)	0	100.00	NC2, TBP	TATAAAA
HPV$HPV18_06	546 (-)	0	100.00	E2, TBP	TATAAAA
AS$E2TBP_01	546 (-)	0	100.00	E2, TBP	TATAAAA
Y$PDC1_02	547 (+)	0	100.00	MIG1	TTTAT
Y$HAP4_01	547 (+)	0	100.00	MIG1	TTTAT
AD$MLP_29	547 (-)	0	100.00	TFIID	TATAAA
TBP$CONS	547 (-)	0	100.00	TBP	TATAAA
HS$EG_08	547 (-)	0	100.00		ATAAA
HS$GFAP_01	547 (-)	0	100.00	TFIID	ATAAA
HS$GFAP_02	547 (-)	0	100.00	TBP	ATAAA
Y$CUP1_07	547 (-)	0	100.00	TBP	TATAAA
MOUSE$MT1_04	547 (-)	0	100.00	TBP	TATAAA
AS$TBP_02	547 (-)	0	100.00	TBP	TATAAA
XENLA$MYODA_02	547 (-)	0	100.00	TBP	TATAAA
RAT$MRF4_03	547 (-)	0	100.00	TBP	TATAAA
MOUSE$MCK_07	548 (+)	1	88.89	aMEF-2, MEF-2 (516 AA)	TTATAATTAA
CHICK$BAC_05	548 (-)	0	100.00	ETF	TATAA
AD$E3_06	548 (-)	0	100.00	TFIID	TATAA
HIV1$HIV1_12	548 (-)	0	100.00	DBF4, NC1, TBP, TFIIA, TFIIA-alpha/beta precursor (major), TFIIA-alpha/beta precursor (minor), TFIIA-gamma, TFIIB, TFIID, TMF	TATAA
HIV1$HIV1_13	548 (-)	0	100.00	UBP-1	TATAA
HS$ASCC_04	548 (-)	0	100.00		TATAA
CHICK$BAC_05	549 (+)	0	100.00	ETF	TATAA
AD$E3_06	549 (+)	0	100.00	TFIID	TATAA
HIV1$HIV1_12	549 (+)	0	100.00	DBF4, NC1, TBP, TFIIA, TFIIA-alpha/beta precursor (major), TFIIA-alpha/beta precursor (minor), TFIIA-gamma, TFIIB, TFIID, TMF	TATAA
HIV1$HIV1_13	549 (+)	0	100.00	UBP-1	TATAA
HS$ASCC_04	549 (+)	0	100.00		TATAA
DROME$CF2_01	549 (-)	1	87.50	CF2-I	TATATTATA
AS$POU3F2_01	550 (-)	1	91.67	POU3F2	ATGCATAAATTAT
AS$POU6F1_03	550 (-)	1	90.00	POU6F1 (c2)	GCATAAATTAT
AS$POU6F1_04	550 (-)	1	90.00	POU6F1 (c2)	GCATAAATTAT
AS$POU6F1_06	550 (-)	1	90.00	POU6F1 (c2)	GCATAAATTAT
AS$POU6F1_10	550 (-)	1	90.00	POU6F1 (c2)	ACATAAATTAT
AS$POU6F1_13	550 (-)	1	90.00	POU6F1 (c2)	GCATAAATTAT
Y$SUC2_02	551 (-)	0	100.00	MIG1	AATTA
RAT$DBH_01	551 (-)	0	100.00	ARIX, c-Fos, c-Jun, CREB, CREMtau	AATTA
HS$GRH_03	553 (-)	0	100.00	POU1F1a	TAAAT
MOUSE$GLUT4_01	553 (-)	1	87.50	C/EBPalpha	TTCAGAAAT
HS$CS1_03	553 (-)	1	88.89	POU1F1a	ATGCATAAAT
HS$CD40_01	553 (-)	1	87.50	AKNA	TTAATAAAT
DROME$FTZ_30	554 (+)	0	100.00	Cad	TTTATG
Y$PDC1_02	554 (+)	0	100.00	MIG1	TTTAT
Y$HAP4_01	554 (+)	0	100.00	MIG1	TTTAT
AS$MEIS1BHOXA9_09	554 (+)	0	100.00	HOXA9, Meis-1b	TTTATGA
DROME$FTZ_29	554 (-)	0	100.00	Cad	CATAAA
HS$EG_08	554 (-)	0	100.00		ATAAA
HS$GFAP_01	554 (-)	0	100.00	TFIID	ATAAA
HS$GFAP_02	554 (-)	0	100.00	TBP	ATAAA
AS$MTTFA_10	555 (+)	0	100.00	mtTFA	TTATG
AS$MTTFA_11	555 (+)	0	100.00	mtTFA	TTATG
RAT$PFK_08	555 (-)	1	88.89	HNF-6alpha, HNF-6beta, OC-2	AAATCCATAA
Y$HXK1_01	556 (+)	1	87.50	MED8	TATGCATTT
RAT$PL_05	557 (-)	1	88.89	POU1F1a	AAAAATGCAT
CAEEL$MEC3_02	557 (-)	0	100.00	unc-86	AAATTCAT
MOUSE$E1_01	558 (-)	1	88.89	POU2F1a, POU2F1b, POU2F1c, POU5F1	TTAAAATTCA
AS$TBP_48	558 (-)	1	90.00	TBP	ATATAAATTCA
Y$MAL61_03	561 (+)	0	100.00	MIG1	ATTTT
AS$MSX1_07	561 (+)	1	87.50	Msx-1	ACTTTTATG
DROME$UBX_17	561 (-)	1	88.89	Hb	ACATAAAAAA
AS$HOXA3_04	561 (-)	0	100.00	HOXA3	CATAAAAAT
Y$SUC2_01	562 (+)	0	100.00	MIG1	TTTTT
AS$PF1_01	562 (+)	0	100.00	PF1	TTTTT
AS$PF1_02	562 (+)	0	100.00	PF1	TTTTT
Y$MAL2R_01	563 (+)	0	100.00	MIG1	TTTTA
AS$FOXJ1_04	563 (+)	0	100.00	FOXJ1	TTTTA
CHICK$VIT2_17	563 (-)	0	100.00		CATAAAA
AS$E2TBP_02	563 (-)	1	87.50	TBP	TATATAAAA
DROME$FTZ_30	564 (+)	0	100.00	Cad	TTTATG
Y$PDC1_02	564 (+)	0	100.00	MIG1	TTTAT
Y$HAP4_01	564 (+)	0	100.00	MIG1	TTTAT
DROME$FTZ_29	564 (-)	0	100.00	Cad	CATAAA
HS$EG_08	564 (-)	0	100.00		ATAAA
HS$GFAP_01	564 (-)	0	100.00	TFIID	ATAAA
HS$GFAP_02	564 (-)	0	100.00	TBP	ATAAA
AS$MTTFA_10	565 (+)	0	100.00	mtTFA	TTATG
AS$MTTFA_11	565 (+)	0	100.00	mtTFA	TTATG
HS$CD40_01	567 (-)	1	87.50	AKNA	TTAATAAAT
Y$MEL1_02	571 (+)	0	100.00	MIG1	ATTAA
CHICK$MGF_01	571 (+)	0	100.00	Gbx2	ATTAA
CHICK$MGF_02	571 (-)	0	100.00	Gbx2	TTAAT
CHICK$MGF_02	572 (+)	0	100.00	Gbx2	TTAAT
AS$NKX61_01	572 (+)	0	100.00	Nkx6-1	TTAATGT
Y$MEL1_02	572 (-)	0	100.00	MIG1	ATTAA
CHICK$MGF_01	572 (-)	0	100.00	Gbx2	ATTAA
HS$GMCSF_04	573 (-)	0	100.00		CATTA
MAIZE$B32_02	573 (-)	1	88.89	Opaque-2	GATGACATGA
AS$MEIS1_14	575 (+)	0	100.00	Meis-1a, Meis-1b	ATGTCA
AS$MEIS1BHOXA9_09	575 (-)	0	100.00	HOXA9, Meis-1b	TGACAT
AS$MEIS1BHOXA9_11	575 (-)	0	100.00	HOXA9, Meis-1b	TGACAT
AS$TGIF_01	576 (+)	0	100.00	TGIF	TGTCA
AS$TGIF_02	576 (+)	0	100.00	TGIF	TGTCA
AS$TGIF_03	576 (+)	0	100.00	TGIF	TGTCA
AS$TGIF_04	576 (+)	0	100.00	TGIF	TGTCA
AS$TGIF_05	576 (+)	0	100.00	TGIF	TGTCA
AS$TGIF_06	576 (+)	0	100.00	TGIF	TGTCA
AS$TGIF_07	576 (+)	0	100.00	TGIF	TGTCA
AS$TGIF_08	576 (+)	0	100.00	TGIF	TGTCA
AS$TGIF_10	576 (+)	0	100.00	TGIF	TGTCA
AS$TGIF_11	576 (+)	0	100.00	TGIF	TGTCA
AS$TGIF_12	576 (+)	0	100.00	TGIF	TGTCA
AS$TGIF_13	576 (+)	0	100.00	TGIF	TGTCA
AS$TGIF_14	576 (+)	0	100.00	TGIF	TGTCA
AS$TGIF_15	576 (+)	0	100.00	TGIF	TGTCA
POT$PR10a_01	576 (+)	0	100.00	PBF-1, PBF-2 (p24)	TGTCA
POT$PR10a_01	576 (-)	0	100.00	PBF-1, PBF-2 (p24)	TGACA
Y$ENO2_03	579 (+)	0	100.00	GCR1	CATCC
Y$TPI_03	579 (+)	0	100.00	GCR1	CATCC
HS$MMP3_01	579 (-)	0	100.00	NIP, PEA3	GGATG
HS$GG_25	581 (-)	0	100.00	PPUR	AGGAGGA
HT1$HTLV1_08	582 (+)	0	100.00	c-Ets-1 68	CCTCC
HS$AACS_02	582 (-)	0	100.00	ARP-1	AGGAGG
MOUSE$TTPA_01	587 (+)	0	100.00	AP-1	TGATC
AS$GR_06	587 (+)	0	100.00	GR	TGATCT
HS$GHA_03	587 (-)	0	100.00	GR, GR-alpha, GR-beta	AGATCA
XENLA$ACY_01	589 (+)	0	100.00	SRF	ATCTT
XENLA$ACY_01	589 (-)	0	100.00	SRF	AAGAT
Y$GAL4_01	592 (+)	0	100.00	MIG1	TTTTC
AS$AREB6_01	594 (+)	1	87.50	AREB6	TTCACCTGC
MOUSE$ICER_01	595 (+)	1	87.50	CREMtau, CREMtau1, CREMtau2, CREMtaualpha, ICER-II, ICER-IIgamma	TGAGCTGCA
RAT$EAI_08	596 (+)	0	100.00	XPF-1	CAGCTG
HS$PK_04	596 (+)	0	100.00	AP-4	CAGCTG
AS$AP4_01	596 (+)	0	100.00	AP-4	CAGCTG
MOUSE$CD4_03	596 (+)	0	100.00	E12, HEB	CAGCTG
RAT$AMY2A_02	596 (+)	0	100.00	XPF-1	CAGCTG
MOUSE$ACRG_02	596 (+)	0	100.00	MyoD	CAGCTG
AS$HEN1_01	596 (+)	0	100.00	E12, HEN1	CAGCTG
RAT$EAI_08	596 (-)	0	100.00	XPF-1	CAGCTG
HS$PK_04	596 (-)	0	100.00	AP-4	CAGCTG
AS$AP4_01	596 (-)	0	100.00	AP-4	CAGCTG
MOUSE$CD4_03	596 (-)	0	100.00	E12, HEB	CAGCTG
RAT$AMY2A_02	596 (-)	0	100.00	XPF-1	CAGCTG
MOUSE$ACRG_02	596 (-)	0	100.00	MyoD	CAGCTG
AS$HEN1_01	596 (-)	0	100.00	E12, HEN1	CAGCTG
DROME$ADH_06	598 (+)	0	100.00	Adf-1	GCTGC
HS$CMYC_16	598 (+)	0	100.00	CTCF	GCTGC
AS$FTZ_52	598 (+)	0	100.00	Ftz	GCTGCA
DROME$ADH_14	598 (-)	0	100.00	Adf-1	GCAGC
MOUSE$A11COL_01	603 (+)	0	100.00	CBF (2), IF2	ATTGG
MOUSE$A21COL_03	603 (+)	0	100.00	CCAAT-binding factor	ATTGG
MOUSE$A21COL_04	603 (+)	0	100.00	CBF (2), CBF-A, CBF-B	ATTGG
MOUSE$A21COL_05	603 (+)	0	100.00	EFI	ATTGG
AD2$E2L_03	603 (+)	0	100.00	CRF	ATTGG
HS$GHA_06	603 (+)	0	100.00	NF-1	ATTGG
SP$H2B1_02	603 (+)	0	100.00	CBF (1)	ATTGG
SP$H2B1_03	603 (+)	0	100.00	CDF	ATTGG
HS$HSP70_02	603 (+)	0	100.00	CTF	ATTGG
HS$HSP70_07	603 (+)	0	100.00	CBTF, CP1, CTF	ATTGG
MOUSE$M2EAK_07	603 (+)	0	100.00	NF-Y	ATTGG
MOUSE$M2EAK_08	603 (+)	0	100.00	NF-Y	ATTGG
RSV$RSV_05	603 (+)	0	100.00		ATTGG
HSV1$TK_04	603 (+)	0	100.00	C/EBPalpha	ATTGG
HSV1$TK_05	603 (+)	0	100.00	NF-Y'	ATTGG
AS$HAP23_01	603 (+)	0	100.00	HAP2, HAP3, HAP4	ATTGG
MOUSE$M1H2KB_12	603 (+)	0	100.00	EFI	ATTGG
RAT$TH2A_02	603 (+)	0	100.00		ATTGG
RAT$TH2A_04	603 (+)	0	100.00		ATTGG
HS$TK_02	603 (+)	0	100.00	CP1	ATTGG
MOUSE$A11COL_07	603 (+)	0	100.00	NF-1, Sp1	ATTGG
HS$CDC2_07	603 (+)	0	100.00	CBAF, CBF-B, CP1A	ATTGG
XENLA$VITB1_11	603 (+)	0	100.00	C/EBP	ATTGGGTAAA
XENLA$GATA2_01	603 (+)	0	100.00	CBTF	ATTGG
RAT$NAT_05	603 (+)	0	100.00		ATTGG
HS$CDC2_12	603 (+)	0	100.00	CBAF	ATTGG
HS$BAC_03	603 (-)	0	100.00	SRF	CCAAT
AMULV$AMULV_01	603 (-)	0	100.00		CCAAT
EBV$DSL_06	603 (-)	0	100.00		CCAAT
MOUSE$AAG_04	603 (-)	0	100.00	alpha-CP1, alpha-CP2a, alpha-CP2b, alpha-IRP	CCAAT
CHICK$BAG_06	603 (-)	0	100.00	TGGCA-binding protein	CCAAT
HS$GG_15	603 (-)	0	100.00	NF-E	CCAAT
HS$GG_16	603 (-)	0	100.00	CDP2, Clox, CUTL1, Cutl1	CCAAT
HS$GG_17	603 (-)	0	100.00	gammaCAAT	CCAAT
HS$GG_18	603 (-)	0	100.00	CP1	CCAAT
HS$ZG_07	603 (-)	0	100.00	CP2	CCAAT
HS$ZG_08	603 (-)	0	100.00	CP1	CCAAT
HS$HH1_03	603 (-)	0	100.00	H1TF2	CCAAT
MOUSE$M2IAB_01	603 (-)	0	100.00	PU.1	CCAAT
PIG$UPA_12	603 (-)	0	100.00	NF-1	CCAAT
RADLV$RLV_10	603 (-)	0	100.00		CCAAT
MULV$MULV_03	603 (-)	0	100.00		CCAAT
MOUSE$GLUT4_04	603 (-)	0	100.00		CCAAT
MOMLV$MOMULV_15	603 (-)	0	100.00		CCAAT
RAT$ALDB_02	603 (-)	0	100.00	CBF (2), CP2	CCAAT
HS$GG_20	603 (-)	0	100.00	CP1, NF-E3	CCAAT
RAT$TH2B_01	603 (-)	0	100.00		CCAAT
RAT$TH2B_02	603 (-)	0	100.00		CCAAT
HS$GG_21	603 (-)	0	100.00	CP1	CCAAT
MOUSE$LY6E_01	603 (-)	0	100.00		CCAAT
RAT$NEU_01	603 (-)	0	100.00		CCAAT
HS$GHA_10	603 (-)	0	100.00	alpha-CBF	CCAAT
NEUCR$CPC1_02	603 (-)	0	100.00		CCAAT
HS$EG_07	603 (-)	0	100.00	CP1	CCAAT
HS$GP2B_13	603 (-)	0	100.00		CCAAT
MOUSE$BMG_10	603 (-)	0	100.00		CCAAT
HS$CYBH_01	603 (-)	0	100.00	CDP2, Clox, CP1, CUTL1, Cutl1	CCAAT
HBV$S_04	603 (-)	0	100.00		CCAAT
HS$FN_06	603 (-)	0	100.00		CCAAT
MOUSE$E2F1_02	603 (-)	0	100.00		CCAAT
HS$A24COL_01	603 (-)	0	100.00		CCAAT
MOUSE$EKLF_01	603 (-)	0	100.00	AP-1, GATA-1	CCAAT
AS$ZIC3_30	605 (+)	1	87.50	Zic3	TGGGTAACT
RICE$PHYA_04	607 (+)	0	100.00	GT-2	GGTAAAT
RICE$PHYA_05	607 (+)	0	100.00	GT-2, PF1	GGTAAAT
NT$PR1A_04	608 (-)	0	100.00	GT-1	ATTTAC
HS$GRH_03	609 (+)	0	100.00	POU1F1a	TAAAT
AS$ARR_01	611 (-)	0	100.00	ARR1, ARR2	AGGATT
HS$APOB_11	614 (+)	0	100.00	LF-A1	CCTTGC
AMV$AMV_02	615 (+)	0	100.00	C/EBPalpha	CTTGC
RAV0$RAV0_01	615 (-)	0	100.00	C/EBPalpha	GCAAG
MOUSE$AFEP_14	616 (+)	0	100.00	C/EBPalpha, HNF-1, HNF-1A	TTGCCT
Y$ANB1_05	616 (+)	0	100.00	MOT3	TTGCCT
Y$CYC1_16	617 (-)	0	100.00	MOT3	CAGGCA
HS$EGFR_19	618 (+)	0	100.00		GCCTGC
MOMLV$MOMULV_09	619 (+)	0	100.00	LVc	CCTGC
MOMLV$MOMULV_10	619 (+)	0	100.00	LVc	CCTGC
RADLV$RLV_03	619 (+)	0	100.00	LVc	CCTGC
MOUSE$IGLL_03	619 (+)	1	87.50	LyF-1	CCTCCCAGA
AS$LYF1_06	619 (-)	1	87.50	LyF-1	TCTGGGAGG
AS$MEIS1AHOXA9_11	620 (-)	0	100.00	HOXA9, Meis-1a	TGGCAG
HS$ALBU_03	621 (-)	0	100.00	NF-1/L	TGGCA
HS$RBP_01	621 (-)	0	100.00	NF-1/L	TGGCA
RAT$A1I3_02	621 (-)	0	100.00	NF-1	TGGCA
RAT$AFEP_11	621 (-)	0	100.00	GR, NF-1	TGGCA
MMTV$MMTV_43	621 (-)	0	100.00	NF-1	TGGCA
RAT$INS2_03	622 (-)	1	88.89		CCCCTCTGGC
RAT$POMC_03	624 (+)	0	100.00	GR	CAGAG
AS$WZF1_01	626 (-)	0	100.00	WZF1	CACTC
WHEAT$H3_02	626 (-)	0	100.00	WZF1	CACTC
WHEAT$H3_03	626 (-)	0	100.00	WZF1	CACTC
HS$GG_40	629 (+)	0	100.00		TGGGTC
MOUSE$RARB_03	629 (+)	0	100.00		TGGGTCA
AS$CF1_04	629 (+)	1	87.50	CF1	GGGGTCAGC
AS$CF1_32	629 (+)	1	87.50	CF1	GGGGTCAGC
HS$DPOLA_01	630 (+)	0	100.00	AP-1	GGGTCA
HS$CS1_04	630 (+)	0	100.00	T3R	GGGTCA
HS$RARG_01	630 (+)	0	100.00	RAR-alpha1, RAR-beta, RAR-gamma, RXR-alpha	GGGTCA
AS$RXR_6	630 (+)	0	100.00	RXR-alpha, TAF(II)28	GGGTCA
AS$LXRA_01	630 (+)	0	100.00	LXR-alpha, RXR-alpha	GGGTCA
RAT$FAS_04	630 (+)	0	100.00	LXR-alpha, RXR-alpha	GGGTCA
HS$CETP_02	630 (+)	0	100.00	LXR-alpha, LXR-beta, RXR-alpha	GGGTCA
HS$APOE_12	630 (+)	0	100.00	LXR-alpha, LXR-beta, RXR-alpha	GGGTCA
CHICK$PTH_01	630 (+)	0	100.00	RXR-alpha, VDR	GGGTCA
HS$ALDA_06	630 (+)	0	100.00	TR2-11	GGGTCA
RAT$ME_08	630 (+)	0	100.00	PPAR-gamma, RXR-gamma	GGGTCA
HS$PLTP_01	630 (+)	0	100.00	FOR1, FOR2, RXR-alpha	GGGTCA
HS$APOB_11	630 (-)	0	100.00	LF-A1	TGACCC
MOUSE$LB1_01	630 (-)	0	100.00	RAR-alpha1, RAR-beta, RAR-gamma	TGACCC
MOUSE$GFCR_02	630 (-)	0	100.00	RAR-alpha1, RORalpha1, RXR-beta2	TGACCC
CHICK$ACCA_01	630 (-)	0	100.00	LXR-alpha, LXR-beta, RXR-alpha, T3R-alpha	TGACCC
MOUSE$SHP1_01	630 (-)	0	100.00	FXR, RXR-alpha	TGACCC
HS$CYP3A4_04	630 (-)	0	100.00	PXR-1, RXR-alpha	TGACCC
RAT$PEPCK_22	630 (-)	0	100.00	COUP, RAR-alpha1, USF-1, USF2	TGACCC
HS$PLTP_01	630 (-)	0	100.00	FOR1, FOR2, RXR-alpha	TGACCC
CHICK$OA_03	631 (+)	0	100.00	ER-alpha	GGTCA
CHICK$OA_04	631 (+)	0	100.00	c-Fos, c-Jun, ER-alpha	GGTCA
AS$RAR_04	631 (+)	0	100.00	RAR-alpha1, T3R-alpha	GGTCA
AS$RAR_06	631 (+)	0	100.00	RAR-alpha1	GGTCA
AS$TR_08	631 (+)	0	100.00	T3R-alpha	GGTCA
AS$TR_09	631 (+)	0	100.00	RAR-alpha1, T3R-alpha	GGTCA
AS$TR_10	631 (+)	0	100.00	T3R-alpha	GGTCA
NT$CHN50_01	631 (+)	0	100.00	WRKY1, WRKY3, WRKY4	GGTCA
HS$ADH3_01	631 (-)	0	100.00	CAR, RAR-alpha1, RAR-beta, RXR-alpha	TGACC
CHICK$OA_14	631 (-)	0	100.00	ER-alpha	TGACC
RAT$EAI_09	632 (+)	0	100.00		GTCAG
AS$ZIC_14	636 (+)	1	87.50	Zic1	GCGGTGATC
HS$PR264_01	638 (+)	0	100.00	c-Myb	GGTGAG
HS$APOA2_06	638 (-)	0	100.00		TCACC
TDNA$NOS_01	640 (+)	0	100.00	ASF-1, OBF3.1, TGA1a, TGA1b	TGAGC
EBV$BZLF1_04	640 (+)	0	100.00	Zta	TGAGCCA
RAT$ARGL_03	642 (+)	0	100.00	NF-1	AGCCAG
HS$PAI1_03	642 (+)	1	87.50		AGCCAGACA
MOUSE$CMYC_01	646 (+)	0	100.00	myc-PRF	AGAAAGGG
HS$TNFA_04	646 (+)	0	100.00		AGAAA
I$HSF_01	646 (+)	0	90.00	HSF	AGAAN
F$HSF_01	646 (+)	0	90.00	HSF	AGAAN
Y$GSY2_02	648 (+)	0	100.00		AAAGGGG
LP$LPS1B_01	649 (-)	1	87.50		AGCCCCCCT
AS$VDR_03	650 (+)	0	100.00	VDR	AGGGGG
AS$VDR_04	650 (+)	0	100.00	VDR	AGGGG
AS$VDR_05	650 (+)	0	100.00	VDR	AGGGGG
AS$VDR_07	650 (+)	0	100.00	VDR	AGGGGG
AS$VDR_09	650 (+)	0	100.00	VDR	AGGGGG
Y$GLK1_02	650 (+)	0	100.00	MSN2, MSN4	AGGGG
HS$CYCD1_14	651 (-)	0	100.00	Sp1, Sp2, Sp3, Sp4	CCCCC
HS$CYCD1_15	651 (-)	0	100.00	Sp1, Sp2, Sp3, Sp4	CCCCC
PA$PY_23	652 (+)	0	100.00	T-Ag	GGGGC
SV$SV40_04	652 (+)	0	100.00	T-Ag	GGGGC
SV$SV40_63	652 (+)	0	100.00	T-Ag	GGGGC
PA$PY_26	652 (-)	0	100.00	T-Ag	GCCCC
MAIZE$ADH11S_06	652 (-)	0	100.00	GCBP-1, Sp1	GCCCC
AS$CF1_34	653 (+)	1	87.50	CF1	GGGGTCATT
TDNA$NOS_01	655 (-)	0	100.00	ASF-1, OBF3.1, TGA1a, TGA1b	TGAGC
ASPN$ABAA_03	658 (+)	0	100.00	abaA	CATTCT
ASPN$ABAA_04	658 (+)	0	100.00	abaA	CATTCT
ASPN$ABAA_05	658 (+)	0	100.00	abaA	CATTCT
ASPN$BRLA_03	658 (+)	0	100.00	abaA	CATTCT
ASPN$RODA_05	658 (+)	0	100.00	abaA	CATTCT
ABAA$CONS_01	658 (+)	0	91.67	abaA	CATTCY
ASPN$BRLA_05	658 (+)	0	100.00	abaA	CATTCT
ASPN$YA_02	658 (-)	0	100.00	abaA	AGAATG
I$HSF_01	659 (-)	0	90.00	HSF	AGAAN
F$HSF_01	659 (-)	0	90.00	HSF	AGAAN
AS$MEIS1AHOXA9_07	663 (+)	0	100.00	HOXA9, Meis-1a	TAACAG
AS$mEMBP_04	666 (-)	0	100.00	EmBP-1a	CACTG
RAT$A1I3_04	668 (+)	0	100.00		GTGCT
RAT$A1I3_04	668 (-)	0	100.00		AGCAC
MOUSE$M2EAK_03	669 (-)	0	100.00	NF-Y	CAGCA
HS$CGB_01	670 (-)	1	88.89		AGGACACACC
RAT$A2UG_11	672 (-)	0	100.00	GR	GACACA
MAIZE$C1_05	673 (+)	0	100.00		GTGTC
RAT$AFEP_06	674 (+)	0	100.00	GR	TGTCCT
HS$GRH_07	674 (+)	0	100.00	GR	TGTCCT
HS$MT2A_01	674 (+)	0	100.00	GR	TGTCCT
CHICK$PR_01	674 (+)	0	100.00	PR-alpha, PR-beta	TGTCCT
CHICK$PR_02	674 (+)	0	100.00	PR-alpha, PR-beta	TGTCCT
AS$PR_01	674 (+)	0	100.00	PR B	TGTCCT
HS$CAMHC_01	674 (+)	0	100.00	Hp55, Hp65, p58, RAR-alpha1, RXR-beta, T3R-alpha, T3R-beta1, T3R-beta2	TGTCCT
RAT$AOX_01	674 (+)	0	100.00	PPAR-alpha, RXR-alpha	TGTCCT
MOUSE$5HT1_01	674 (+)	0	100.00	GR, MR	TGTCCT
RAT$OC_01	674 (-)	0	100.00	RXR-alpha, VDR	AGGACA
CHICK$PR_04	674 (-)	0	100.00	PR-alpha, PR-beta	AGGACA
AS$RAR_15	674 (-)	0	100.00	RAR-alpha1, RXR-alpha	AGGACA
RAT$OC_05	674 (-)	0	100.00	RXR-alpha, VDR	AGGACA
AS$ERR1_12	674 (-)	0	100.00	ERR1	AGGACA
AS$ERR1_14	674 (-)	0	100.00	ERR1	AGGACA
AS$ERR1_23	674 (-)	0	100.00	ERR1	AGGACA
RAT$SGK_02	674 (-)	0	100.00	GR	AGGACA
RAT$ALDH3_01	674 (-)	0	100.00	GR	AGGACA
HS$GG_25	676 (-)	0	100.00	PPUR	AGGAGGA
HT1$HTLV1_08	677 (+)	0	100.00	c-Ets-1 68	CCTCC
HS$PDGFB_04	677 (+)	1	88.89		CCTCCTGGCG
HS$AACS_02	677 (-)	0	100.00	ARP-1	AGGAGG
AS$STAT5A_66	678 (+)	1	87.50	STAT5A	TTCCTGGAG
Y$ADH2_01	683 (-)	0	100.00	ADR1	TCTCC
DROME$EVE_08	684 (+)	0	100.00	GAGA factor	GAGAG
DROME$EVE_13	684 (+)	0	100.00	GAGA factor	GAGAG
DROME$FTZ_32	684 (+)	0	100.00	GAGA factor	GAGAG
AS$GAGA_03	684 (+)	0	100.00	GAGA factor	GAGAG
DROME$EVE_09	684 (-)	0	100.00	GAGA factor	CTCTC
DROME$FTZ_32	684 (-)	0	100.00	GAGA factor	CTCTC
AS$WZF1_01	686 (-)	0	100.00	WZF1	CACTC
WHEAT$H3_02	686 (-)	0	100.00	WZF1	CACTC
WHEAT$H3_03	686 (-)	0	100.00	WZF1	CACTC
XENLA$U2SN_11	688 (+)	0	100.00		GTGCC
EBV$IR4_05	688 (-)	0	100.00	R	GGCAC
EBV$IR4_06	688 (-)	0	100.00	R	GGCAC
RAT$OMP_07	688 (-)	0	100.00		TGGCAC
CAMV$35SR_06	688 (-)	0	100.00	MNB1b	TGGCAC
HS$ALBU_02	689 (-)	0	100.00	NF-1/L	TTGGCA
HS$ALBU_03	689 (-)	0	100.00	NF-1/L	TGGCA
HS$RBP_01	689 (-)	0	100.00	NF-1/L	TGGCA
RAT$A1I3_02	689 (-)	0	100.00	NF-1	TGGCA
RAT$AFEP_11	689 (-)	0	100.00	GR, NF-1	TGGCA
MMTV$MMTV_43	689 (-)	0	100.00	NF-1	TGGCA
HBV$SA_01	690 (+)	0	100.00	NF-1	GCCAAC
PV$DLEC2_02	690 (+)	1	87.50	ROM1, ROM2	GCCACCTCA
PV$DLEC2_03	690 (+)	1	87.50	ROM1, ROM2	GCCACCTCA
PV$PHSB_01	690 (+)	1	87.50	ROM1, ROM2	GCCACCTCA
MOUSE$AP2_07	690 (-)	0	100.00	NF-1	TTGGC
Y$CYC1_09	691 (-)	0	100.00	hap2, HAP2, HAP3	GTTGG
Y$CYC1_10	691 (-)	0	100.00	CP1A, HAP2	GTTGG
Y$CYC1_11	691 (-)	0	100.00	HAP3, NF-YA	GTTGG
HS$PR264_06	692 (+)	0	100.00	c-Myb	CAACT
ASPN$ABAA_03	697 (+)	0	100.00	abaA	CATTCT
ASPN$ABAA_04	697 (+)	0	100.00	abaA	CATTCT
ASPN$ABAA_05	697 (+)	0	100.00	abaA	CATTCT
ASPN$BRLA_03	697 (+)	0	100.00	abaA	CATTCT
ASPN$RODA_05	697 (+)	0	100.00	abaA	CATTCT
ABAA$CONS_01	697 (+)	0	91.67	abaA	CATTCY
ASPN$BRLA_05	697 (+)	0	100.00	abaA	CATTCT
ASPN$YA_02	697 (-)	0	100.00	abaA	AGAATG
I$HSF_01	698 (-)	0	90.00	HSF	AGAAN
F$HSF_01	698 (-)	0	90.00	HSF	AGAAN
Y$ADH2_01	700 (+)	0	100.00	ADR1	TCTCC
AS$STAT5A_70	701 (+)	1	87.50	STAT5A	TTCCAAGTA
AS$STAT5A_67	701 (-)	1	87.50	STAT5A	TACCTGGAG
HS$NPY_03	703 (+)	0	100.00	AP-1, CCAAT-binding factor	CCAAG
AS$NKX3A_09	704 (+)	0	100.00	NKX3A	CAAGTA
HS$TSHB_04	705 (+)	0	100.00	T3R-beta1	AAGTAA
AS$HB_13	705 (+)	1	88.89	Hb	AAATAAAAAA
DROME$UBX_19	706 (+)	1	88.89	Hb	CGTAAAAAAA
DROME$KR_24	706 (+)	1	87.50	Hb	AGTAAAAAC
MOUSE$ADA_06	708 (+)	0	100.00	TBP	TAAAAAA
Y$MAL2R_01	708 (-)	0	100.00	MIG1	TTTTA
AS$FOXJ1_04	708 (-)	0	100.00	FOXJ1	TTTTA
Y$SUC2_01	709 (-)	0	100.00	MIG1	TTTTT
AS$PF1_01	709 (-)	0	100.00	PF1	TTTTT
AS$PF1_02	709 (-)	0	100.00	PF1	TTTTT
Y$SUC2_01	710 (-)	0	100.00	MIG1	TTTTT
AS$PF1_01	710 (-)	0	100.00	PF1	TTTTT
AS$PF1_02	710 (-)	0	100.00	PF1	TTTTT
Y$SUC2_01	711 (-)	0	100.00	MIG1	TTTTT
AS$PF1_01	711 (-)	0	100.00	PF1	TTTTT
AS$PF1_02	711 (-)	0	100.00	PF1	TTTTT
RAT$ARGL_03	715 (+)	0	100.00	NF-1	AGCCAG
MOUSE$BMG_04	719 (-)	0	100.00	GATA-1	AATCT
HS$IGH_04	721 (+)	0	100.00		ATTTG
Y$SMK1_02	721 (+)	1	87.50	SUM1	ATTTGTGAC
HS$EGFR_14	721 (-)	0	100.00		CAAAT
MAIZE$PMS1_01	721 (-)	0	100.00		CAAAT
Y$SPS4_01	721 (-)	1	87.50	NDT80	GCCACAAAA
RAT$A2UG_13	724 (+)	1	87.50	GR	TGTGGCACA
EBV$BZLF1_04	726 (-)	0	100.00	Zta	TGAGCCA
TDNA$NOS_01	728 (-)	0	100.00	ASF-1, OBF3.1, TGA1a, TGA1b	TGAGC
HS$IFNB_02	730 (-)	0	100.00	IRF-1, IRF-2	AAGTGA
HS$IFNB_09	730 (-)	0	100.00		AAGTGA
AS$ZIC2_17	734 (+)	1	87.50	Zic2	TTCGTGGTG
MAIZE$ADH1P_01	736 (+)	0	100.00		CGTGG
MAIZE$ADH1P_03	736 (+)	0	100.00		CGTGG
V$MZF1_01	736 (+)	0	87.50	MZF1	NGNGGGGA
Y$MEL1_02	737 (+)	0	100.00	MIG1	GTGGGG
Y$MAL61_03	737 (+)	0	100.00	MIG1	GTGGGG
Y$MAL61_04	737 (+)	0	100.00	MIG1	GTGGGG
MZF1$CONS_01	737 (+)	0	92.86	MZF-1	GNGGGGA
MOUSE$M1H2KB_02	738 (+)	0	100.00	AP-2alpha, AP-2alphaA, AP-2alphaB	TGGGGA
MOUSE$M1H2KB_07	738 (+)	0	100.00	MBP-1 (1), NF-kappaB1, NF-kappaB2, NF-kappaB2 precursor	TGGGGA
MOUSE$M1H2KB_07	738 (-)	0	100.00	MBP-1 (1), NF-kappaB1, NF-kappaB2, NF-kappaB2 precursor	TCCCCA
HS$IFI616_01	741 (+)	0	100.00	ISGF-3	GGAAA
HS$INS_05	741 (+)	0	100.00		GGAAAT
MOUSE$IL5_02	741 (+)	0	100.00	NF-ATc, NF-ATp, NF-ATx	GGAAA
HS$IL4_01	741 (+)	0	100.00		GGAAA
MOUSE$IL4_06	741 (+)	0	100.00		GGAAA
MOUSE$IL4_07	741 (+)	0	100.00		GGAAA
MOUSE$IL4_08	741 (+)	0	100.00		GGAAA
HS$IL5_02	741 (+)	0	100.00		GGAAA
HS$IL13_01	741 (+)	0	100.00		GGAAA
MOUSE$CD40L_01	741 (+)	0	100.00		GGAAA
MOUSE$CD40L_02	741 (+)	0	100.00		GGAAA
MOUSE$CD40L_03	741 (+)	0	100.00		GGAAA
MOUSE$CD40L_04	741 (+)	0	100.00		GGAAA
HS$CD40L_01	741 (+)	0	100.00		GGAAA
HS$CD40L_02	741 (+)	0	100.00		GGAAA
HS$IFNG_03	741 (+)	0	100.00		GGAAA
HS$IFNG_04	741 (+)	0	100.00		GGAAA
HS$TNFA_05	741 (+)	0	100.00		GGAAA
MOUSE$MCP3_01	741 (+)	0	100.00		GGAAA
HS$TNFSF6_01	741 (+)	0	100.00		GGAAA
HS$GMCSF_03	743 (-)	0	100.00	YY1	CATTT
RAT$A2UG_11	746 (-)	0	100.00	GR	GACACA
MAIZE$C1_05	747 (+)	0	100.00		GTGTC
HS$CRH_01	748 (-)	0	100.00	GR	TGGACA
Y$LYS1_01	750 (+)	1	87.50	LYS14	TCCAGCGGA
CHICK$STN1_01	751 (+)	0	100.00	LBP-1, MyoD	CCAGC
CHICK$STN1_03	751 (+)	0	100.00	LBP-1, NF-1 (-like proteins)	CCAGC
AS$SPZ1_11	752 (-)	1	87.50	Spz1	GGTGCGCAG
RAT$CYP24_02	755 (+)	0	100.00	VDR	CGCACC
AS$HOXA3_14	756 (-)	1	87.50	HOXA3	CGTTTGTGC
Y$CYC1_09	759 (-)	0	100.00	hap2, HAP2, HAP3	GTTGG
Y$CYC1_10	759 (-)	0	100.00	CP1A, HAP2	GTTGG
Y$CYC1_11	759 (-)	0	100.00	HAP3, NF-YA	GTTGG
AS$MATALPHA2_12	760 (-)	1	88.89	MATalpha2	GCCTGCGATG
HS$EGFR_19	764 (-)	0	100.00		GCCTGC
MOMLV$MOMULV_09	764 (-)	0	100.00	LVc	CCTGC
MOMLV$MOMULV_10	764 (-)	0	100.00	LVc	CCTGC
RADLV$RLV_03	764 (-)	0	100.00	LVc	CCTGC
DROME$EVE_10	768 (-)	0	100.00	GAGA factor	CTCGC
AS$SPZ1_13	769 (+)	1	87.50	Spz1	CGAGGAACT
CTCF$CONS	770 (-)	0	100.00	CTCF	CCCTC
EBV$IR4_04	772 (+)	0	100.00	R	GGGAC
EBV$IR4_05	772 (+)	0	100.00	R	GGGAC
HS$TERT_05	772 (-)	1	87.50	Sp1	CCCAGCCCC
HS$TERT_06	772 (-)	1	87.50	Sp1	CCCAGCCCC
CHICK$STN1_02	775 (-)	0	100.00	LBP-1, NF-1 (-like proteins)	CCAGT
HS$PAI_07	775 (-)	0	100.00		CCAGT
SV$SV40_37	776 (+)	0	100.00		CTGGG
CHICK$BAG_11	776 (-)	1	87.50	PPYR	CTCCCCCGG
HS$APOB_26	776 (-)	0	100.00		CTCCTCCCCCAG
Y$GAL3_01	776 (-)	0	100.00	MIG1	CCCCAG
Y$GAL4_01	776 (-)	0	100.00	MIG1	CCCCAG
Y$HAP4_01	776 (-)	0	100.00	MIG1	CCCCAG
AS$ZIC3_20	777 (+)	1	87.50	Zic3	TGGGGGAGC
HS$AAC_10	778 (+)	1	88.89	Sp1	GGGGGAGGGG
H4TF1$CONS	778 (+)	1	87.50	H4TF-1	GGGGGAGGG
HS$A24COL_02	778 (-)	1	90.00		CCCCCTCCCCC
CHICK$CA2_02	778 (-)	0	100.00	RXR-alpha, VDR	TCCCCC
HS$CYCD1_14	778 (-)	0	100.00	Sp1, Sp2, Sp3, Sp4	CCCCC
HS$CYCD1_15	778 (-)	0	100.00	Sp1, Sp2, Sp3, Sp4	CCCCC
HS$PFKM_03	779 (+)	0	100.00		GGGGAGGAGG
HS$MIP_03	779 (+)	1	87.50	Sp1	GGGGAGGGG
HS$ZG_06	779 (-)	1	87.50	Sp1	CCCCTCCCC
H4TF1$CONS	781 (+)	1	87.50	H4TF-1	GGGGGAGGG
HT1$HTLV1_08	781 (-)	0	100.00	c-Ets-1 68	CCTCC
CHICK$BAG_07	782 (+)	1	87.50		GAGGAGGGG
HS$APOB_06	782 (+)	0	100.00	C/EBPalpha	GAGGAGGG
AS$ZIC_24	782 (+)	1	87.50	Zic1	GAGGAGGGC
HS$EGFR_04	782 (-)	1	88.89	Sp1	ATCCCTCCTC
HS$AACS_02	783 (+)	0	100.00	ARP-1	AGGAGG
AS$SPZ1_06	784 (+)	1	87.50	Spz1	GGAGGGAAA
AS$SPZ1_07	784 (+)	1	87.50	Spz1	GGAGGGAAC
AS$SPZ1_12	784 (+)	1	87.50	Spz1	GGAGGGTAG
HT1$HTLV1_08	784 (-)	0	100.00	c-Ets-1 68	CCTCC
PA$PY_11	785 (+)	1	87.50		GAGGGCAGT
PA$PY_14	785 (+)	1	87.50		GAGGGCAGT
RAT$ALAL_01	785 (+)	0	100.00	MAF	GAGGGAAGT
SHEEP$BLG_08	785 (+)	0	100.00	MAF	GAGGGAAGT
PA$PY_11	785 (-)	1	87.50		ACTGCCCTC
PA$PY_14	785 (-)	1	87.50		ACTGCCCTC
CTCF$CONS	785 (-)	0	100.00	CTCF	CCCTC
HS$CDC2_06	787 (+)	0	100.00	c-Ets-2	GGGAAG
AS$NFKAPPAB_29	787 (+)	1	88.89	NF-kappaB	GGGAAGTCCC
AS$NFKAPPAB_33	787 (+)	1	88.89	NF-kappaB	GGGAAGTACC
MOUSE$M2EAD_01	787 (-)	1	88.89	NF-kappaB, NF-kappaB1	GGGACTTCCC
AS$NFKAPPAB_08	787 (-)	1	88.89	NF-kappaB	GGGACTTCCC
MOUSE$RPL32_01	788 (+)	0	100.00	f(alpha)-f(epsilon), HrpF, XrpFI	GGAAG
HS$TNFA_02	788 (+)	0	100.00		GGAAG
HS$TNFA_03	788 (+)	0	100.00		GGAAG
XENLA$RPL14_01	788 (-)	0	100.00	HrpF, XrpFI	CTTCC
Y$TPI_01	788 (-)	0	100.00	GCR1	CTTCC
MOUSE$IL2RA_03	788 (-)	0	100.00	c-Ets-1, Elf-1	ACTTCC
AS$SPZ1_22	789 (-)	1	87.50	Spz1	GGGCGCTTC
PA$PY_11	791 (+)	1	87.50		ACTGCCCTC
PA$PY_14	791 (+)	1	87.50		ACTGCCCTC
PA$PY_11	791 (-)	1	87.50		GAGGGCAGT
PA$PY_14	791 (-)	1	87.50		GAGGGCAGT
XENLA$U2SN_11	792 (+)	0	100.00		GTGCC
EBV$IR4_05	792 (-)	0	100.00	R	GGCAC
EBV$IR4_06	792 (-)	0	100.00	R	GGCAC
AS$SPZ1_07	792 (-)	1	87.50	Spz1	GGAGGGAAC
CHICK$BAG_03	793 (+)	0	100.00		TGCCC
MOUSE$CRABP2_01	793 (+)	0	100.00	RXR-alpha, RXR-gamma	TGCCCT
HS$CABD9_01	793 (+)	0	100.00	RXR-alpha, VDR	TGCCCT
RAT$VEGF_02	793 (+)	0	100.00	ER-alpha, ER-beta	TGCCC
CHICK$CYP2H1_05	793 (+)	0	100.00	CXR, RXR-gamma	TGCCCT
CHICK$BAG_03	793 (-)	0	100.00		GGGCA
RAT$NF1_01	793 (-)	0	100.00	LF-A1	GGGCA
AS$RAR_16	793 (-)	0	100.00	RAR-alpha1, RXR-alpha	AGGGCA
HS$CATHD_01	793 (-)	0	100.00	ER-alpha, Sp1	GGGCA
RAT$VEGF_01	793 (-)	0	100.00	ER-alpha, ER-beta	GGGCA
HS$ALDA_06	793 (-)	0	100.00	TR2-11	AGGGCA
AS$ZIC_24	794 (-)	1	87.50	Zic1	GAGGAGGGC
CTCF$CONS	795 (+)	0	100.00	CTCF	CCCTC
HT1$HTLV1_08	796 (+)	0	100.00	c-Ets-1 68	CCTCC

HS$AACS_02	796 (-)	0	100.00	ARP-1	AGGAGG
HS$EGFR_20	798 (+)	0	100.00		TCCTGC
MOMLV$MOMULV_09	799 (+)	0	100.00	LVc	CCTGC
MOMLV$MOMULV_10	799 (+)	0	100.00	LVc	CCTGC
RADLV$RLV_03	799 (+)	0	100.00	LVc	CCTGC
AS$FTZ_52	801 (-)	0	100.00	Ftz	GCTGCA
DROME$ADH_14	802 (+)	0	100.00	Adf-1	GCAGC
DROME$ADH_06	802 (-)	0	100.00	Adf-1	GCTGC
HS$CMYC_16	802 (-)	0	100.00	CTCF	GCTGC
MOUSE$M2EAK_03	803 (+)	0	100.00	NF-Y	CAGCA
RAT$A1I3_04	804 (+)	0	100.00		AGCAC
RAT$A1I3_04	804 (-)	0	100.00		GTGCT
RAT$CYTOP_04	806 (+)	0	100.00	AhR, Arnt	CACGC
DAUCE$DC3_04	806 (+)	0	100.00	DPBF-1, DPBF-2	CACGCG
AS$AHRARNT_50	806 (-)	0	100.00	AhR, Arnt	GCGTG
Y$CDC9_02	807 (+)	0	100.00	DSC1	ACGCGA
Y$CDC6_01	807 (+)	0	100.00	MCBF	ACGCGA
AS$ZIC2_29	808 (+)	1	87.50	Zic2	GGCGAGGTT
AS$ZIC3_14	808 (+)	1	87.50	Zic3	CGGGAGGTT
DROME$EVE_10	809 (-)	0	100.00	GAGA factor	CTCGC
AS$TR_07	814 (+)	0	100.00	T3R-alpha	GTTCC
AS$TR_10	814 (+)	0	100.00	T3R-alpha	GTTCC
HS$IL4_02	814 (-)	0	100.00		GGAAC
AS$ELK1_10	815 (+)	0	100.00	Elk-1, SRF	TTCCGG
AS$STAT_01	815 (+)	1	87.50	STAT6	TTCCGGGAA
AS$STAT_02	815 (+)	1	87.50	STAT6	TTCCGGGAA
AS$STAT5A_38	815 (+)	1	87.50	STAT5A	TTCCGGGAA
AS$STAT5A_44	815 (+)	1	87.50	STAT5A	TTCCGGGAA
AS$STAT5A_64	815 (+)	1	87.50	STAT5A	TTCCGGGAG
E74A$CONS_01	815 (-)	0	90.00	E74A	MGGAA
AS$ELK1_02	815 (-)	0	100.00	Elk-1, SRF	CCGGAA
AS$ELK1_03	815 (-)	0	100.00	Elk-1, SRF	CCGGAA
AS$ELK1_04	815 (-)	0	100.00	Elk-1, SRF	CCGGAA
AS$ELK1_05	815 (-)	0	100.00	Elk-1, SRF	CCGGAA
AS$ELK1_06	815 (-)	0	100.00	Elk-1, SRF	CCGGAA
AS$ELK1_08	815 (-)	0	100.00	Elk-1, SRF	CCGGAA
AS$ELK1_13	815 (-)	0	100.00	Elk-1, SAP-1a, SAP-1b, SRF	CCGGAA
AS$STAT_10	815 (-)	1	87.50	STAT6	TTCCCGGAA
AS$STAT5A_39	815 (-)	1	87.50	STAT5A	TTCCCGGAA
HS$P21WAF1_09	815 (-)	1	87.50	STAT1, STAT3	TTCCCGGAA
Y$BAP2_01	818 (+)	1	87.50	LEU3	CGGAACCGG
EBV$IR4_04	819 (+)	0	100.00	R	GGGAC
EBV$IR4_05	819 (+)	0	100.00	R	GGGAC
HS$HH4_02	820 (-)	0	100.00	H4TF-2	GGTCC
AS$TR_11	820 (-)	0	100.00	T3R-alpha	GGTCC
AS$ZIC_21	821 (-)	1	87.50	Zic1	CAGCTGGTC
AS$ZIC3_15	821 (-)	1	87.50	Zic3	CAGCTGGTC
MOUSE$IGH_16	823 (+)	1	87.50	E47, HEB, INSAF, MyoD, myogenin, Tal-1	CCAGCTGGC
RAT$MLC_02	823 (+)	1	87.50	HEB, MyoD, myogenin	CCAGCTGGC
QUAIL$STN1_02	825 (-)	0	100.00		CCAGCC
AD$MLP_19	826 (-)	0	100.00		GGCCAGC
CHICK$STN1_01	826 (-)	0	100.00	LBP-1, MyoD	CCAGC
CHICK$STN1_03	826 (-)	0	100.00	LBP-1, NF-1 (-like proteins)	CCAGC
HS$GRH_01	828 (+)	0	100.00	AP-2, AP-2alphaA, AP-2alphaB, NF-1	TGGCC
HSV1$TK_12	828 (+)	0	100.00	RAR-alpha1, T3R-alpha	TGGCCT
HS$GRH_01	828 (-)	0	100.00	AP-2, AP-2alphaA, AP-2alphaB, NF-1	GGCCA
RAT$TAT_15	828 (-)	0	100.00	HNF-3alpha	GGCCA
HS$EGFR_19	830 (+)	0	100.00		GCCTGC
MOUSE$IGH_16	830 (-)	1	87.50	E47, HEB, INSAF, MyoD, myogenin, Tal-1	CCAGCTGGC
RAT$MLC_02	830 (-)	1	87.50	HEB, MyoD, myogenin	CCAGCTGGC
MOMLV$MOMULV_09	831 (+)	0	100.00	LVc	CCTGC
MOMLV$MOMULV_10	831 (+)	0	100.00	LVc	CCTGC
RADLV$RLV_03	831 (+)	0	100.00	LVc	CCTGC
MOUSE$TDT_03	831 (-)	1	87.50	LyF-1	TTCAGCAGG
MOUSE$M2EAK_03	833 (-)	0	100.00	NF-Y	CAGCA
CHICK$STN1_01	834 (-)	0	100.00	LBP-1, MyoD	CCAGC
CHICK$STN1_03	834 (-)	0	100.00	LBP-1, NF-1 (-like proteins)	CCAGC
RAT$AMGL_01	835 (+)	0	100.00	IL-6 RE-BP	CTGGAA
MOUSE$ALBU_13	835 (+)	1	88.89	ANF	CTTGAACTCG
HS$IL4_02	837 (+)	0	100.00		GGAAC
AS$TR_07	837 (-)	0	100.00	T3R-alpha	GTTCC
AS$TR_10	837 (-)	0	100.00	T3R-alpha	GTTCC
HS$CYP24_01	838 (+)	0	100.00	VDR	GAACTC
HS$GRH_01	844 (+)	0	100.00	AP-2, AP-2alphaA, AP-2alphaB, NF-1	GGCCA
RAT$TAT_15	844 (+)	0	100.00	HNF-3alpha	GGCCA
HS$GRH_01	844 (-)	0	100.00	AP-2, AP-2alphaA, AP-2alphaB, NF-1	TGGCC
HS$CYCB1_02	844 (-)	0	100.00	AP-2alphaA, AP-2alphaB	GCCTGGCC
Y$GAL1_10	848 (-)	0	100.00	GAL4	AGCCT
HS$APOB_11	848 (-)	0	100.00	LF-A1	GAGCCT
TDNA$NOS_01	850 (-)	0	100.00	ASF-1, OBF3.1, TGA1a, TGA1b	TGAGC
MOUSE$AAMY_08	850 (-)	1	87.50	PTF1-beta	CAGCTGTGC
RAT$ATP1A2_01	850 (-)	1	87.50		CAGCTGGGC
V$AP4_Q6	851 (+)	0	90.00	AP-4	CWCAGCTGGN
MOUSE$IGH_16	852 (+)	1	87.50	E47, HEB, INSAF, MyoD, myogenin, Tal-1	CCAGCTGGC
RAT$MLC_02	852 (+)	1	87.50	HEB, MyoD, myogenin	CCAGCTGGC
RAT$EAI_08	853 (+)	0	100.00	XPF-1	CAGCTG
HS$PK_04	853 (+)	0	100.00	AP-4	CAGCTG
AS$AP4_01	853 (+)	0	100.00	AP-4	CAGCTG
MOUSE$CD4_03	853 (+)	0	100.00	E12, HEB	CAGCTG
RAT$AMY2A_02	853 (+)	0	100.00	XPF-1	CAGCTG
MOUSE$ACRG_02	853 (+)	0	100.00	MyoD	CAGCTG
AS$HEN1_01	853 (+)	0	100.00	E12, HEN1	CAGCTG
RAT$EAI_08	853 (-)	0	100.00	XPF-1	CAGCTG
HS$PK_04	853 (-)	0	100.00	AP-4	CAGCTG
AS$AP4_01	853 (-)	0	100.00	AP-4	CAGCTG
MOUSE$CD4_03	853 (-)	0	100.00	E12, HEB	CAGCTG
RAT$AMY2A_02	853 (-)	0	100.00	XPF-1	CAGCTG
MOUSE$ACRG_02	853 (-)	0	100.00	MyoD	CAGCTG
AS$HEN1_01	853 (-)	0	100.00	E12, HEN1	CAGCTG
CHICK$STN1_01	855 (-)	0	100.00	LBP-1, MyoD	CCAGC
CHICK$STN1_03	855 (-)	0	100.00	LBP-1, NF-1 (-like proteins)	CCAGC
RAT$ARGL_03	856 (-)	0	100.00	NF-1	AGCCAG
HS$TERT_05	863 (-)	1	87.50	Sp1	CCCAGCCCC
HS$TERT_06	863 (-)	1	87.50	Sp1	CCCAGCCCC
MOUSE$M1H2KB_03	864 (-)	1	87.50	AP-2alphaA, AP-2alphaB	GCCCAGGGC
CHICK$STN1_01	866 (-)	0	100.00	LBP-1, MyoD	CCAGC
CHICK$STN1_03	866 (-)	0	100.00	LBP-1, NF-1 (-like proteins)	CCAGC
SV$SV40_37	867 (+)	0	100.00		CTGGG
SV$SV40_63	868 (+)	0	100.00	T-Ag	TGGGC
CHICK$BAG_03	869 (+)	0	100.00		GGGCA
RAT$NF1_01	869 (+)	0	100.00	LF-A1	GGGCA
PIG$UPA_09	869 (+)	0	100.00	Sp1	GGGCAG
HS$CATHD_01	869 (+)	0	100.00	ER-alpha, Sp1	GGGCA
RAT$VEGF_01	869 (+)	0	100.00	ER-alpha, ER-beta	GGGCA
CHICK$BAG_03	869 (-)	0	100.00		TGCCC
RAT$VEGF_02	869 (-)	0	100.00	ER-alpha, ER-beta	TGCCC
AS$ZIC3_04	869 (-)	1	87.50	Zic3	TGGGTGCCC
AS$P113_01	870 (+)	0	100.00	P113	GGCAGCC
DROME$ADH_14	871 (+)	0	100.00	Adf-1	GCAGC
DROME$ADH_06	871 (-)	0	100.00	Adf-1	GCTGC
HS$CMYC_16	871 (-)	0	100.00	CTCF	GCTGC
RAT$ARGL_03	873 (+)	0	100.00	NF-1	AGCCAG
RAT$MLC_04	878 (+)	0	100.00	MAPF2, YY1	GGAGC
HS$GMCSF_10	878 (+)	0	100.00	NF-ATp	GGAGCC
HS$APOB_11	879 (+)	0	100.00	LF-A1	GAGCCT
Y$GAL1_10	880 (+)	0	100.00	GAL4	AGCCT
SV$SV40_37	883 (+)	0	100.00		CTGGG
SV$SV40_63	884 (+)	0	100.00	T-Ag	TGGGC
MOUSE$PAC1R_01	885 (+)	0	100.00	ZAC-1a	GGGCCC
MOUSE$PAC1R_01	885 (-)	0	100.00	ZAC-1a	GGGCCC
HS$GG_36	886 (-)	0	100.00	Sp1	GGGGCC
PA$PY_26	887 (+)	0	100.00	T-Ag	GCCCC
MAIZE$ADH11S_06	887 (+)	0	100.00	GCBP-1, Sp1	GCCCC
PA$PY_23	887 (-)	0	100.00	T-Ag	GGGGC
SV$SV40_04	887 (-)	0	100.00	T-Ag	GGGGC
SV$SV40_63	887 (-)	0	100.00	T-Ag	GGGGC
MAIZE$ADH1P_02	888 (+)	0	100.00		CCCCGG
Y$MAL2R_01	888 (+)	0	100.00	MIG1	CCCCGG
Y$MAL63_01	888 (+)	0	100.00	MIG1	CCCCGG
CHICK$BAG_11	889 (-)	1	87.50	PPYR	CTCCCCCGG
MAIZE$ADH1P_02	890 (-)	0	100.00		CCCCGG
Y$MAL2R_01	890 (-)	0	100.00	MIG1	CCCCGG
Y$MAL63_01	890 (-)	0	100.00	MIG1	CCCCGG
H4TF1$CONS	891 (+)	1	87.50	H4TF-1	GGGGGAGGG
HS$GFAP_04	891 (+)	1	88.89	AP-1, AP-2alphaA, AP-2alphaB, NF-1	CGGGGTGGGC
HS$MIP_03	892 (+)	1	87.50	Sp1	GGGGAGGGG
HS$DHFR_08	892 (+)	1	87.50	Sp1	GGGGCGGGC
AS$ZIC_24	892 (+)	1	87.50	Zic1	GAGGAGGGC
AS$ZIC2_09	892 (+)	1	87.50	Zic2	GGGGGGGGC
HS$ZG_06	892 (-)	1	87.50	Sp1	CCCCTCCCC
MOUSE$CMYC_04	893 (+)	0	100.00	MAZ	GGGAGGG
MOUSE$CD4_04	893 (+)	0	100.00	MAZ	GGGAGGG
HS$GX_WT1_02	893 (-)	1	87.50	WT1 -KTS, WT1 I, WT1 I -KTS, WT1 I-del2, WT1-del2	CTCCCTCCC
MOUSE$THY1_04	893 (-)	0	100.00	R1, R2, Sp1	CCCTCCC
HS$FREAC4_01	893 (-)	1	87.50	WT1	CGCCCTCTC
HT1$HTLV1_08	894 (-)	0	100.00	c-Ets-1 68	CCTCC
PA$PY_11	895 (+)	1	87.50		GAGGGCAGT
PA$PY_14	895 (+)	1	87.50		GAGGGCAGT
PA$PY_11	895 (-)	1	87.50		ACTGCCCTC
PA$PY_14	895 (-)	1	87.50		ACTGCCCTC
CTCF$CONS	895 (-)	0	100.00	CTCF	CCCTC
TTK88$CONS	896 (+)	0	92.86	Ttk 88K	AGGGYGG
HS$P21WAF1_03	896 (+)	0	100.00	Sp1	AGGGCGG
HS$CYP24_02	896 (-)	0	100.00	VDR	CGCCCT
RAT$CYP24_01	896 (-)	0	100.00	VDR	CGCCCT
HS$APOE_08	897 (+)	0	100.00	Sp1	GGGCGG
HA$HMGCR_03	897 (+)	0	100.00		GGGCGG
HSV1$IE3_04	897 (+)	0	100.00	Sp1	GGGCGG
HSV1$IE3_07	897 (+)	0	100.00	Sp1	GGGCGG
HSV1$IE3_09	897 (+)	0	100.00	Sp1	GGGCGG
HSV1$IE4_01	897 (+)	0	100.00	Sp1	GGGCGG
HSV1$IE4_03	897 (+)	0	100.00	Sp1	GGGCGG
SV$SV40_06	897 (+)	0	100.00	LSF	GGGCGG
SV$SV40_07	897 (+)	0	100.00	ETF, Sp1	GGGCGG
SV$SV40_13	897 (+)	0	100.00	Sp1	GGGCGG
HS$U2SN_03	897 (+)	0	100.00	Sp1	GGGCGG
HS$U2SN_05	897 (+)	0	100.00	Sp1	GGGCGG
PIG$UPA_10	897 (+)	0	100.00	Sp1	GGGCGG
PIG$UPA_11	897 (+)	0	100.00	Sp1	GGGCGG
PIG$UPA_14	897 (+)	0	100.00	Sp1	GGGCGG
MOUSE$GLUT4_05	897 (+)	0	100.00	Sp1	GGGCGG
AD5$E1A_15	897 (+)	0	100.00	Sp1	GGGCGG
HS$NPY_01	897 (+)	0	100.00	Sp1	GGGCGG
LSF$CONS	897 (+)	0	87.50	LSF	GGGCGGNNNNGGGCGG
RAT$IGF2_01	897 (+)	0	100.00	Sp1	GGGCGG
HS$TK_01	897 (+)	0	100.00	CP1	GGGCGG
HS$A24COL_03	897 (+)	0	100.00	Sp1	GGGCGG
HS$TK_07	897 (+)	0	100.00	Sp1	GGGCGG
HS$TNFRSF6_02	897 (+)	1	88.89	NF-kappaB, NF-kappaB1	GGGCGTTCCC
HS$TNFRSF6_03	897 (+)	1	88.89	Sp1	GGGCGTTCCC
CHICK$ACRA_07	897 (-)	0	100.00	Sp1	CCGCCC
MOUSE$APRT_01	897 (-)	0	100.00	Sp1	CCGCCC
RAT$GF_07	897 (-)	0	100.00	Sp1	CCGCCC
HA$HMGCR_03	897 (-)	0	100.00		CCGCCC
HSV1$IE3_05	897 (-)	0	100.00	Sp1	CCGCCC
HSV1$IE4_04	897 (-)	0	100.00	Sp1	CCGCCC
MOUSE$EPOR_02	897 (-)	0	100.00	Sp1	CCGCCC
RAT$CDC2_02	897 (-)	0	100.00		CCGCCC
MOUSE$E2F1_01	897 (-)	0	100.00		CCGCCC
HS$CD11B_01	897 (-)	0	100.00	Sp1	CGCCC
HS$TERT_04	897 (-)	0	100.00	Sp1	CCGCCC
HS$TERT_07	897 (-)	0	100.00	Sp1	CCGCCC
HS$CDC25C_04	898 (+)	0	100.00		GGCGG
HS$HH4_02	901 (+)	0	100.00	H4TF-2	GGTCC
AS$TR_11	901 (+)	0	100.00	T3R-alpha	GGTCC
RAT$GF_03	901 (-)	1	87.50	USF1	CCCGTGACC
EBV$IR4_04	902 (-)	0	100.00	R	GGGAC
EBV$IR4_05	902 (-)	0	100.00	R	GGGAC
HS$P21WAF1_12	905 (+)	1	91.67		CCGGGCGGGGCGG
HS$HSP70_10	906 (+)	1	88.89	Sp1	GGGGCGGCGC
HSV1$TK_03	906 (+)	1	88.89	Sp1	GGGGCGGCGC
RAT$FAS_02	906 (+)	1	88.89	Sp1	TGGGCGGCGC
HS$RAS1_02	906 (-)	1	88.89	Sp1	GCCCCGCCCG
MOUSE$SREBP1C_02	906 (-)	0	100.00	LXR-alpha, LXR-beta, RXR-alpha	CGCCCG
MOUSE$GSC_01	906 (-)	1	87.50	SMAD-4	GGCCGCCCG
HS$CYCD1_15	906 (-)	0	100.00	Sp1, Sp2, Sp3, Sp4	GCCCG
HS$APOE_08	907 (+)	0	100.00	Sp1	GGGCGG
HA$HMGCR_03	907 (+)	0	100.00		GGGCGG
HSV1$IE3_04	907 (+)	0	100.00	Sp1	GGGCGG
HSV1$IE3_07	907 (+)	0	100.00	Sp1	GGGCGG
HSV1$IE3_09	907 (+)	0	100.00	Sp1	GGGCGG
HSV1$IE4_01	907 (+)	0	100.00	Sp1	GGGCGG
HSV1$IE4_03	907 (+)	0	100.00	Sp1	GGGCGG
SV$SV40_06	907 (+)	0	100.00	LSF	GGGCGG
SV$SV40_07	907 (+)	0	100.00	ETF, Sp1	GGGCGG
SV$SV40_13	907 (+)	0	100.00	Sp1	GGGCGG
HS$U2SN_03	907 (+)	0	100.00	Sp1	GGGCGG
HS$U2SN_05	907 (+)	0	100.00	Sp1	GGGCGG
PIG$UPA_10	907 (+)	0	100.00	Sp1	GGGCGG
PIG$UPA_11	907 (+)	0	100.00	Sp1	GGGCGG
PIG$UPA_14	907 (+)	0	100.00	Sp1	GGGCGG
MOUSE$GLUT4_05	907 (+)	0	100.00	Sp1	GGGCGG
AD5$E1A_15	907 (+)	0	100.00	Sp1	GGGCGG
HS$NPY_01	907 (+)	0	100.00	Sp1	GGGCGG
CHICK$BAC_06	907 (+)	1	88.89	GCF	GGGCGGGGCG
RAT$IGF2_01	907 (+)	0	100.00	Sp1	GGGCGG
HS$TK_01	907 (+)	0	100.00	CP1	GGGCGG
HS$A24COL_03	907 (+)	0	100.00	Sp1	GGGCGG
AS$SP1_17	907 (+)	1	88.89	Sp1	GGGCGGGGCG
HS$TK_07	907 (+)	0	100.00	Sp1	GGGCGG
CHICK$ACRA_07	907 (-)	0	100.00	Sp1	CCGCCC
MOUSE$APRT_01	907 (-)	0	100.00	Sp1	CCGCCC
RAT$GF_07	907 (-)	0	100.00	Sp1	CCGCCC
HA$HMGCR_03	907 (-)	0	100.00		CCGCCC
HSV1$IE3_05	907 (-)	0	100.00	Sp1	CCGCCC
HSV1$IE4_04	907 (-)	0	100.00	Sp1	CCGCCC
MOUSE$EPOR_02	907 (-)	0	100.00	Sp1	CCGCCC
RAT$CDC2_02	907 (-)	0	100.00		CCGCCC
MOUSE$E2F1_01	907 (-)	0	100.00		CCGCCC
HS$CD11B_01	907 (-)	0	100.00	Sp1	CGCCC
HS$TERT_04	907 (-)	0	100.00	Sp1	CCGCCC
HS$TERT_07	907 (-)	0	100.00	Sp1	CCGCCC
HS$CDC25C_04	908 (+)	0	100.00		GGCGG
AS$GCCBOX_01	908 (-)	0	100.00	ERF2, ERF3, ERF4	GCCGCC
AS$GCCBOX_02	908 (-)	0	100.00	Pti4	GCCGCC
CHICK$BLCR_02	909 (+)	0	100.00	NF-E4, Sp1	GCGGC
HSV1$TK_08	911 (+)	0	100.00	ZF5	GGCGCG
HS$GAST_02	911 (+)	1	88.89		GGCGGGGTGG
AS$ALFIN1_01	912 (+)	1	87.50	Alfin1	GAGCGGTGG
HS$GFAP_04	913 (+)	1	88.89	AP-1, AP-2alphaA, AP-2alphaB, NF-1	CGGGGTGGGC
HS$CDC2_09	913 (+)	0	100.00		CGCGG
HS$CYCA_05	913 (+)	0	100.00		CGCGG
HS$CYCD1_14	913 (-)	0	100.00	Sp1, Sp2, Sp3, Sp4	CCGCG
AS$ZIC_01	914 (+)	1	87.50	Zic1	GCGGTGGTC
BPV1$BPV1_24	916 (+)	0	100.00	Sp1	GGTGG
HS$BG_48	916 (+)	0	100.00	CAC-binding protein	GGTGG
MOUSE$CEBPA_06	916 (+)	0	100.00	Sp1	GGTGG
HS$GPB_02	916 (+)	0	100.00	Sp1	GGTGGG
HT1$HTLV1_08	916 (-)	0	100.00	c-Ets-1 68	CCACC
HS$BG_52	916 (-)	0	100.00		CCACC
HS$BG_14	916 (-)	0	100.00		CCACC
HS$BG_44	916 (-)	0	100.00	CAC-binding protein	CCACC
SV$SV40_63	918 (+)	0	100.00	T-Ag	TGGGC
RAT$MT1_04	922 (-)	1	87.50		TGCGCTCGG
DROME$EVE_04	924 (-)	0	100.00	GAGA factor	CGCTC
DROME$EVE_08	924 (-)	0	100.00	GAGA factor	CGCTC
DROME$EVE_10	924 (-)	0	100.00	GAGA factor	CGCTC
HS$FN_08	926 (-)	0	100.00	AP-2	CCCGCGC
HS$CDC2_09	927 (+)	0	100.00		CGCGG
HS$CYCA_05	927 (+)	0	100.00		CGCGG
HS$CYCD1_14	927 (-)	0	100.00	Sp1, Sp2, Sp3, Sp4	CCGCG
HS$P21WAF1_04	930 (+)	0	100.00	Sp1	GGGTCCCGCCTCCTTGAGGCGGGCCCGGGCG
HS$HH4_02	931 (+)	0	100.00	H4TF-2	GGTCC
AS$TR_11	931 (+)	0	100.00	T3R-alpha	GGTCC
XENLA$U2SN_08	932 (-)	1	88.89	octamer-binding factor, Sp1	GAGGCGGGGC
XENLA$U2SN_09	932 (-)	1	88.89	octamer-binding factor, Sp1	GAGGCGGGGC
HA$DHFR_05	932 (-)	1	88.89	Sp1	GAGGCGGGGC
AS$SP1_10	932 (-)	1	88.89	Sp1	GAGGCGGGCC
EBV$IR4_04	932 (-)	0	100.00	R	GGGAC
EBV$IR4_05	932 (-)	0	100.00	R	GGGAC
AS$SP1_33	932 (-)	1	88.89	Sp1	GAGGCGGGCC
RAT$OMP_06	933 (-)	1	87.50	Sp1	GAGGCGGGC
HS$GAPDH_01	934 (+)	0	100.00	IRE-ABP, SRY	CCCGCCTC
HS$P21WAF1_06	934 (+)	0	100.00	SMAD-3, SMAD-4	CCCGCC
HS$P21WAF1_07	934 (+)	0	100.00		CCCGCC
HSV1$IE3_06	934 (-)	0	100.00	Sp1	GGCGGG
RAT$NF1_02	934 (-)	0	100.00	Sp1	GGCGGG
HS$NPY_02	934 (-)	0	100.00	Sp1	GGCGGG
MOUSE$TS_05	934 (-)	1	88.89	Sp1	AAGAGGCGGG
HS$MIP_02	934 (-)	0	100.00	Sp1	GGCGGG
HS$TPI_04	935 (-)	0	100.00	Sp1	AGGCGG
HS$CDC25C_04	935 (-)	0	100.00		GGCGG
HS$CDC25C_02	936 (-)	0	100.00		GAGGCG
SV$SV40_01	937 (+)	0	100.00	T-Ag	GCCTC
SV$SV40_02	937 (+)	0	100.00	T-Ag	GCCTC
SV$SV40_63	937 (+)	0	100.00	T-Ag	GCCTC
PA$PY_24	937 (-)	0	100.00	T-Ag	GAGGC
PA$PY_25	937 (-)	0	100.00	T-Ag	GAGGC
SV$SV40_02	937 (-)	0	100.00	T-Ag	GAGGC
MOUSE$RPL7_01	937 (-)	1	87.50	delta factor	CATGGAGGC
HT1$HTLV1_08	938 (+)	0	100.00	c-Ets-1 68	CCTCC
HS$AACS_02	938 (-)	0	100.00	ARP-1	AGGAGG
PA$PY_24	945 (+)	0	100.00	T-Ag	GAGGC
PA$PY_25	945 (+)	0	100.00	T-Ag	GAGGC
SV$SV40_02	945 (+)	0	100.00	T-Ag	GAGGC
XENLA$U2SN_08	945 (+)	1	88.89	octamer-binding factor, Sp1	GAGGCGGGGC
XENLA$U2SN_09	945 (+)	1	88.89	octamer-binding factor, Sp1	GAGGCGGGGC
HA$DHFR_05	945 (+)	1	88.89	Sp1	GAGGCGGGGC
AS$SP1_10	945 (+)	0	100.00	Sp1	GAGGCGGGCC
RAT$OMP_06	945 (+)	0	100.00	Sp1	GAGGCGGGC
HS$CDC25C_02	945 (+)	0	100.00		GAGGCG
AS$SP1_33	945 (+)	0	100.00	Sp1	GAGGCGGGCC
HS$DHFR_08	945 (+)	1	87.50	Sp1	GGGGCGGGC
AS$ZIC_24	945 (+)	1	87.50	Zic1	GAGGAGGGC
SV$SV40_01	945 (-)	0	100.00	T-Ag	GCCTC
SV$SV40_02	945 (-)	0	100.00	T-Ag	GCCTC
HS$GAPDH_01	945 (-)	0	100.00	IRE-ABP, SRY	CCCGCCTC
SV$SV40_63	945 (-)	0	100.00	T-Ag	GCCTC
HS$TPI_04	946 (+)	0	100.00	Sp1	AGGCGG
AS$ZIC3_22	946 (+)	1	87.50	Zic3	AGGGGGGCC
MOUSE$MCK_11	946 (-)	1	88.89	p53	GGGCCTGCCT
HSV1$IE3_06	947 (+)	0	100.00	Sp1	GGCGGG
RAT$NF1_02	947 (+)	0	100.00	Sp1	GGCGGG
HS$NPY_02	947 (+)	0	100.00	Sp1	GGCGGG
HS$CDC25C_04	947 (+)	0	100.00		GGCGG
HS$MIP_02	947 (+)	0	100.00	Sp1	GGCGGG
HS$P21WAF1_06	947 (-)	0	100.00	SMAD-3, SMAD-4	CCCGCC
HS$P21WAF1_07	947 (-)	0	100.00		CCCGCC
HS$CYCD1_15	949 (-)	0	100.00	Sp1, Sp2, Sp3, Sp4	GCCCG
HS$ASCC_01	950 (+)	1	88.89	Sp1	GGGGCCGGGC
MOUSE$PAC1R_01	950 (+)	0	100.00	ZAC-1a	GGGCCC
MOUSE$PAC1R_01	950 (-)	0	100.00	ZAC-1a	GGGCCC
MOUSE$JUND_09	952 (+)	1	87.50	Sp1	GCGCGGGCG
HS$CYCD1_15	952 (+)	0	100.00	Sp1, Sp2, Sp3, Sp4	GCCCG
RAT$IGF1R_05	952 (-)	1	87.50	WT1 -KTS, WT1 I -KTS	CGCCCGCGC
CHICK$GATA1_09	954 (+)	1	88.89		GCGGGCGGGG
HS$P21WAF1_12	954 (+)	0	100.00		CCGGGCGGGGCGG
HS$TK_03	954 (-)	1	87.50	E2F	CCCGCCAGG
AD2$E1B_03	955 (+)	1	88.89	BTEB1, MAZ, Sp1	GGGGCGGGGC
HS$MT2A_10	955 (+)	1	87.50	Sp1	GGGGCGGGG
HS$RAS1_03	955 (+)	1	88.89	Sp1	GGGGCGGGGC
HS$RAS1_04	955 (+)	1	88.89	Sp1	GGGGCGGGGC
AD2$E1B_02	955 (+)	1	88.89	Sp1, TBP, TFIID	GGGGCGGGGC
HA$DHFR_04	955 (+)	1	88.89	Sp1	TGGGCGGGGC
AS$SP1_02	955 (+)	1	88.89	Sp1	GGGGCGGGGC
RAT$GSTP_09	955 (+)	1	87.50		GGGGCGGGG
MOUSE$PFK_05	955 (+)	1	87.50		GGGGCGGGG
MOUSE$ADA_02	955 (+)	1	88.89	Sp1	TGGGCGGGGC
HS$FN_07	955 (+)	1	87.50	Sp1	GGGGCGGGG
HS$DHFR_05	955 (+)	1	88.89	Sp1	GGGGCGGGGC
HS$DHFR_06	955 (+)	1	88.89	Sp1	GGGGCGGGGC
HS$A11COL_01	955 (-)	1	88.89	Sp1	GCCCCGCCCC
HS$RAS1_02	955 (-)	0	100.00	Sp1	GCCCCGCCCG
HS$RAS1_06	955 (-)	1	88.89	Sp1	GCCCCGCCCC
RAT$ME_02	955 (-)	1	88.89	Sp1	GCCCCGCCCC
HS$CYCA_01	955 (-)	1	88.89		GCCCCGCCCC
MOUSE$SREBP1C_02	955 (-)	0	100.00	LXR-alpha, LXR-beta, RXR-alpha	CGCCCG
HS$CYCD1_15	955 (-)	0	100.00	Sp1, Sp2, Sp3, Sp4	GCCCG
HS$APOE_08	956 (+)	0	100.00	Sp1	GGGCGG
HA$HMGCR_03	956 (+)	0	100.00		GGGCGG
HSV1$IE3_04	956 (+)	0	100.00	Sp1	GGGCGG
HSV1$IE3_07	956 (+)	0	100.00	Sp1	GGGCGG
HSV1$IE3_09	956 (+)	0	100.00	Sp1	GGGCGG
HSV1$IE4_01	956 (+)	0	100.00	Sp1	GGGCGG
HSV1$IE4_03	956 (+)	0	100.00	Sp1	GGGCGG
SV$SV40_06	956 (+)	0	100.00	LSF	GGGCGG
SV$SV40_07	956 (+)	0	100.00	ETF, Sp1	GGGCGG
SV$SV40_13	956 (+)	0	100.00	Sp1	GGGCGG
HS$U2SN_03	956 (+)	0	100.00	Sp1	GGGCGG
HS$U2SN_05	956 (+)	0	100.00	Sp1	GGGCGG
PIG$UPA_10	956 (+)	0	100.00	Sp1	GGGCGG
PIG$UPA_11	956 (+)	0	100.00	Sp1	GGGCGG
PIG$UPA_14	956 (+)	0	100.00	Sp1	GGGCGG
MOUSE$GLUT4_05	956 (+)	0	100.00	Sp1	GGGCGG
AD5$E1A_15	956 (+)	0	100.00	Sp1	GGGCGG
HS$NPY_01	956 (+)	0	100.00	Sp1	GGGCGG
CHICK$BAC_06	956 (+)	0	100.00	GCF	GGGCGGGGCG
RAT$IGF2_01	956 (+)	0	100.00	Sp1	GGGCGG
HS$TK_01	956 (+)	0	100.00	CP1	GGGCGG
AS$SP1_15	956 (+)	0	100.00	POU2F1, Sp1	GGGCGGGG
HS$A24COL_03	956 (+)	0	100.00	Sp1	GGGCGG
AS$SP1_17	956 (+)	0	100.00	Sp1	GGGCGGGGCG
AS$ADR1_16	956 (+)	0	100.00	ADR1	GGGCGGGG
HS$TK_07	956 (+)	0	100.00	Sp1	GGGCGG
AS$ZIC2_09	956 (+)	1	87.50	Zic2	GGGGGGGGC
CHICK$ACRA_07	956 (-)	0	100.00	Sp1	CCGCCC
MOUSE$APRT_01	956 (-)	0	100.00	Sp1	CCGCCC
RAT$GF_07	956 (-)	0	100.00	Sp1	CCGCCC
HA$HMGCR_03	956 (-)	0	100.00		CCGCCC
HSV1$IE3_05	956 (-)	0	100.00	Sp1	CCGCCC
HSV1$IE4_04	956 (-)	0	100.00	Sp1	CCGCCC
MOUSE$TPA_01	956 (-)	1	87.50	Sp1	ACCCCGCCC
MOUSE$EPOR_02	956 (-)	0	100.00	Sp1	CCGCCC
AS$E2SP1_01	956 (-)	0	100.00	E2, Sp1	CCCCGCCC
RAT$CDC2_02	956 (-)	0	100.00		CCGCCC
MOUSE$E2F1_01	956 (-)	0	100.00		CCGCCC
HS$CD11B_01	956 (-)	0	100.00	Sp1	CGCCC
HS$TERT_04	956 (-)	0	100.00	Sp1	CCGCCC
HS$TERT_07	956 (-)	0	100.00	Sp1	CCGCCC
HSV1$IE3_06	957 (+)	0	100.00	Sp1	GGCGGG
RAT$NF1_02	957 (+)	0	100.00	Sp1	GGCGGG
HS$NPY_02	957 (+)	0	100.00	Sp1	GGCGGG
HS$GAST_02	957 (+)	1	88.89		GGCGGGGTGG
HS$CDC25C_04	957 (+)	0	100.00		GGCGG
HS$MIP_02	957 (+)	0	100.00	Sp1	GGCGGG
HS$TK_06	957 (+)	0	100.00	Sp1	GGCGGGG
HS$TK_09	957 (-)	0	100.00	Sp1	CCCCGCC
HS$P21WAF1_06	957 (-)	0	100.00	SMAD-3, SMAD-4	CCCGCC
HS$P21WAF1_07	957 (-)	0	100.00		CCCGCC
Y$SUC2_01	958 (-)	0	100.00	MIG1	CCCCGC
Y$PDC1_02	958 (-)	0	100.00	MIG1	CCCCGC
PA$PY_23	960 (+)	0	100.00	T-Ag	GGGGC
SV$SV40_04	960 (+)	0	100.00	T-Ag	GGGGC
SV$SV40_63	960 (+)	0	100.00	T-Ag	GGGGC
AS$ZIC2_20	960 (+)	1	87.50	Zic2	GGGGGGGTT
RAT$HSC70_01	960 (+)	0	100.00	Sp1, Sp3	GGGGCGG
PA$PY_26	960 (-)	0	100.00	T-Ag	GCCCC
HS$TK_08	960 (-)	0	100.00	Sp1	CCGCCCC
MAIZE$ADH11S_06	960 (-)	0	100.00	GCBP-1, Sp1	GCCCC
HS$APOE_08	961 (+)	0	100.00	Sp1	GGGCGG
HA$HMGCR_03	961 (+)	0	100.00		GGGCGG
HSV1$IE3_04	961 (+)	0	100.00	Sp1	GGGCGG
HSV1$IE3_07	961 (+)	0	100.00	Sp1	GGGCGG
HSV1$IE3_09	961 (+)	0	100.00	Sp1	GGGCGG
HSV1$IE4_01	961 (+)	0	100.00	Sp1	GGGCGG
HSV1$IE4_03	961 (+)	0	100.00	Sp1	GGGCGG
SV$SV40_06	961 (+)	0	100.00	LSF	GGGCGG
SV$SV40_07	961 (+)	0	100.00	ETF, Sp1	GGGCGG
SV$SV40_13	961 (+)	0	100.00	Sp1	GGGCGG
HS$U2SN_03	961 (+)	0	100.00	Sp1	GGGCGG
HS$U2SN_05	961 (+)	0	100.00	Sp1	GGGCGG
PIG$UPA_10	961 (+)	0	100.00	Sp1	GGGCGG
PIG$UPA_11	961 (+)	0	100.00	Sp1	GGGCGG
PIG$UPA_14	961 (+)	0	100.00	Sp1	GGGCGG
MOUSE$GLUT4_05	961 (+)	0	100.00	Sp1	GGGCGG
AD5$E1A_15	961 (+)	0	100.00	Sp1	GGGCGG
HS$NPY_01	961 (+)	0	100.00	Sp1	GGGCGG
RAT$IGF2_01	961 (+)	0	100.00	Sp1	GGGCGG
HS$TK_01	961 (+)	0	100.00	CP1	GGGCGG
HS$A24COL_03	961 (+)	0	100.00	Sp1	GGGCGG
HS$TK_07	961 (+)	0	100.00	Sp1	GGGCGG
CHICK$ACRA_07	961 (-)	0	100.00	Sp1	CCGCCC
MOUSE$APRT_01	961 (-)	0	100.00	Sp1	CCGCCC
RAT$GF_07	961 (-)	0	100.00	Sp1	CCGCCC
HA$HMGCR_03	961 (-)	0	100.00		CCGCCC
HSV1$IE3_05	961 (-)	0	100.00	Sp1	CCGCCC
HSV1$IE4_04	961 (-)	0	100.00	Sp1	CCGCCC
MOUSE$EPOR_02	961 (-)	0	100.00	Sp1	CCGCCC
RAT$CDC2_02	961 (-)	0	100.00		CCGCCC
MOUSE$E2F1_01	961 (-)	0	100.00		CCGCCC
HS$CD11B_01	961 (-)	0	100.00	Sp1	CGCCC
HS$TERT_04	961 (-)	0	100.00	Sp1	CCGCCC
HS$TERT_07	961 (-)	0	100.00	Sp1	CCGCCC
HS$CDC25C_04	962 (+)	0	100.00		GGCGG
AS$GAMYB_05	962 (-)	0	100.00	GAMYB	CAACCGCC
AS$CF1_37	963 (+)	1	87.50	CF1	GGGGTTGTA
RAT$GLU_04	970 (+)	0	100.00		TATAT
HS$HH4_08	970 (+)	0	100.00	HiNF-D, HiNF-M, HiNF-P, TFIID, TMF	TATAT
MOUSE$SRF_03	970 (+)	0	100.00	SRF (504 AA)	TATAT
MOUSE$BMG_05	972 (+)	0	100.00	factor B2, GATA-1	TATCA
MOUSE$AAG_01	972 (-)	0	100.00	GATA-1, GATA-2, GATA-3	TGATA
MOUSE$AAG_06	972 (-)	0	100.00	GATA-1	TGATA
AS$ZF5_25	976 (+)	1	90.00	ZF5	AGGGTCGCGCT
AS$ZF5_35	976 (+)	1	90.00	ZF5	AGGGCCGCGCA
HS$EGFR_22	978 (-)	0	100.00	GCF	CAGCGCGGCC
HS$CAVEOLIN1_03	978 (-)	0	100.00	Sp1	GCGGCC
HS$MT2A_09	979 (-)	1	88.89	AP-4	TCAGCGCGGG
CHICK$BLCR_02	979 (-)	0	100.00	NF-E4, Sp1	GCGGC
HS$CYCD1_14	980 (+)	0	100.00	Sp1, Sp2, Sp3, Sp4	CCGCG
HS$CDC2_09	980 (-)	0	100.00		CGCGG
HS$CYCA_05	980 (-)	0	100.00		CGCGG
P$CDC5_01	981 (-)	0	90.91	CDC5	NGCTCAGCGCN
TDNA$NOS_01	986 (+)	0	100.00	ASF-1, OBF3.1, TGA1a, TGA1b	TGAGC
MOUSE$ICER_01	986 (+)	1	87.50	CREMtau, CREMtau1, CREMtau2, CREMtaualpha, ICER-II, ICER-IIgamma	TGAGCTGCA
DROME$ADH_06	989 (+)	0	100.00	Adf-1	GCTGC
HS$CMYC_16	989 (+)	0	100.00	CTCF	GCTGC
DROME$ADH_14	989 (-)	0	100.00	Adf-1	GCAGC
RAT$TAT_27	990 (+)	1	87.50	CREB, CREBbeta	CTGCGTCAG
RAT$INS1_10	993 (+)	1	87.50		CGCCATCTG
MOUSE$IGH_16	994 (-)	1	87.50	E47, HEB, INSAF, MyoD, myogenin, Tal-1	CCAGCTGGC
RAT$MLC_02	994 (-)	1	87.50	HEB, MyoD, myogenin	CCAGCTGGC
V$AP4_Q6	994 (-)	0	90.00	AP-4	CWCAGCTGGN
CHICK$STN1_01	995 (+)	0	100.00	LBP-1, MyoD	CCAGC
CHICK$STN1_03	995 (+)	0	100.00	LBP-1, NF-1 (-like proteins)	CCAGC
PA$PY_09	995 (-)	1	88.89	AP-4	CCGCAGCTGG
SV$SV40_48	995 (-)	1	88.89	AP-4	CCACAGCTGG
RAT$EAI_08	996 (+)	0	100.00	XPF-1	CAGCTG
HS$PK_04	996 (+)	0	100.00	AP-4	CAGCTG
AS$AP4_01	996 (+)	0	100.00	AP-4	CAGCTG
MOUSE$CD4_03	996 (+)	0	100.00	E12, HEB	CAGCTG
RAT$AMY2A_02	996 (+)	0	100.00	XPF-1	CAGCTG
MOUSE$ACRG_02	996 (+)	0	100.00	MyoD	CAGCTG
AS$HEN1_01	996 (+)	0	100.00	E12, HEN1	CAGCTG
RAT$EAI_08	996 (-)	0	100.00	XPF-1	CAGCTG
HS$PK_04	996 (-)	0	100.00	AP-4	CAGCTG
AS$AP4_01	996 (-)	0	100.00	AP-4	CAGCTG
MOUSE$CD4_03	996 (-)	0	100.00	E12, HEB	CAGCTG
RAT$AMY2A_02	996 (-)	0	100.00	XPF-1	CAGCTG
MOUSE$ACRG_02	996 (-)	0	100.00	MyoD	CAGCTG
AS$HEN1_01	996 (-)	0	100.00	E12, HEN1	CAGCTG
DELTAEF1$CONS_01	1000 (-)	0	92.86	deltaEF1	CACCTNA
PV$PHSB_02	1000 (-)	1	87.50	ROM1, ROM2	CACACGTCA
AS$AREB6_05	1000 (-)	1	87.50	AREB6	CACACCTGA
MOUSE$TRP1_03	1004 (+)	0	100.00	Tbx2	GTGTGA
MOUSE$TRP1_04	1004 (+)	0	100.00	Tbx2	GTGTGA
TDNA$NOS_01	1007 (+)	0	100.00	ASF-1, OBF3.1, TGA1a, TGA1b	TGAGC
RAT$VEGF_02	1008 (+)	0	100.00	ER-alpha, ER-beta	GAGCA
AS$ZIC_21	1008 (-)	1	87.50	Zic1	CAGCTGGTC
AS$ZIC3_15	1008 (-)	1	87.50	Zic3	CAGCTGGTC
DROME$ADH_14	1010 (+)	0	100.00	Adf-1	GCAGC
DROME$ADH_06	1010 (-)	0	100.00	Adf-1	GCTGC
HS$CMYC_16	1010 (-)	0	100.00	CTCF	GCTGC
MAIZE$BZ1_03	1010 (-)	1	87.50	B-Peru	GGCAGGTGC
RAT$EAI_08	1011 (+)	0	100.00	XPF-1	CAGCTG
HS$PK_04	1011 (+)	0	100.00	AP-4	CAGCTG
AS$AP4_01	1011 (+)	0	100.00	AP-4	CAGCTG
MOUSE$CD4_03	1011 (+)	0	100.00	E12, HEB	CAGCTG
RAT$AMY2A_02	1011 (+)	0	100.00	XPF-1	CAGCTG
MOUSE$ACRG_02	1011 (+)	0	100.00	MyoD	CAGCTG
AS$HEN1_01	1011 (+)	0	100.00	E12, HEN1	CAGCTG
RAT$EAI_08	1011 (-)	0	100.00	XPF-1	CAGCTG
HS$PK_04	1011 (-)	0	100.00	AP-4	CAGCTG
AS$AP4_01	1011 (-)	0	100.00	AP-4	CAGCTG
MOUSE$CD4_03	1011 (-)	0	100.00	E12, HEB	CAGCTG
RAT$AMY2A_02	1011 (-)	0	100.00	XPF-1	CAGCTG
MOUSE$ACRG_02	1011 (-)	0	100.00	MyoD	CAGCTG
AS$HEN1_01	1011 (-)	0	100.00	E12, HEN1	CAGCTG
Y$CAR1_01	1012 (+)	1	87.50	repressor of CAR1 expression	AGCCGCCGA
Y$TOP1_01	1012 (+)	1	87.50	repressor of CAR1 expression	AGCCGCCGA
Y$CAR2_01	1012 (+)	1	87.50	repressor of CAR1 expression	AGCCGCCGA
DROME$ADH_06	1013 (+)	0	100.00	Adf-1	GCTGC
HS$CMYC_16	1013 (+)	0	100.00	CTCF	GCTGC
DROME$ADH_14	1013 (-)	0	100.00	Adf-1	GCAGC
Y$MEL1_01	1017 (+)	0	100.00	GAL4	CCGAA
Y$MEL1_01	1017 (-)	0	100.00	GAL4	TTCGG
Y$HIS3_03	1020 (+)	0	100.00	GCN4	AAGTCA
Y$ILV1_03	1020 (+)	0	100.00	GCN4	AAGTCA
RAT$CRBP1_01	1020 (+)	0	100.00	COUP-TF1, RAR-beta, RXR-alpha	AAGTCA
AS$RAR_11	1020 (+)	0	100.00	RAR-alpha1, RXR-alpha	AAGTCA
AS$DSF_01	1020 (+)	0	100.00	DSF	AAGTCA
AS$DSF_02	1020 (+)	0	100.00	DSF	AAGTCA
AS$DSF_03	1020 (+)	0	100.00	DSF	AAGTCA
AS$DSF_04	1020 (+)	0	100.00	DSF	AAGTCA
AS$DSF_06	1020 (+)	0	100.00	DSF	AAGTCA
AS$DSF_07	1020 (+)	0	100.00	DSF	AAGTCA
AS$DSF_08	1020 (+)	0	100.00	DSF	AAGTCA
AS$DSF_09	1020 (+)	0	100.00	DSF	AAGTCA
AS$DSF_06	1020 (-)	0	100.00	DSF	TGACTT
AS$DSF_07	1020 (-)	0	100.00	DSF	TGACTT
AS$DSF_08	1020 (-)	0	100.00	DSF	TGACTT
AS$DSF_09	1020 (-)	0	100.00	DSF	TGACTT
NT$CHN50_01	1021 (+)	0	100.00	WRKY1, WRKY3, WRKY4	AGTCA
RAT$EAI_09	1022 (+)	0	100.00		GTCAG
SV$SV40_59	1022 (-)	0	100.00		AACTGAC
AS$VMYB_21	1022 (-)	1	88.89	v-Myb	AGCAACTGAC
DROME$MDG1_01	1023 (+)	0	100.00		TCAGTT
HS$IFNB_03	1023 (-)	0	100.00		AACTGA
HS$PR264_02	1024 (+)	0	100.00	c-Myb	CAGTTC
HS$PR264_11	1024 (+)	0	100.00	c-Myb	CAGTTC
AS$SPZ1_13	1025 (-)	1	87.50	Spz1	CGAGGAACT
AS$TR_07	1026 (+)	0	100.00	T3R-alpha	GTTCC
AS$TR_10	1026 (+)	0	100.00	T3R-alpha	GTTCC
HS$IL4_02	1026 (-)	0	100.00		GGAAC
HS$CDC2_02	1027 (+)	0	100.00	c-Ets-2	TTCCTT
AS$STAT5A_49	1027 (+)	1	87.50	STAT5A	TTCCGTGTG
AS$STAT5A_55	1027 (+)	1	87.50	STAT5A	TTCCTGGTG
DROME$E74_10	1027 (-)	0	100.00	E74A	AGGAA
DROME$E74_11	1027 (-)	0	100.00	E74A	AGGAA
E74A$CONS_01	1027 (-)	0	90.00	E74A	MGGAA
HS$CDC2_01	1027 (-)	0	100.00	c-Ets-2	AAGGAA
MOUSE$HES1_01	1030 (+)	0	100.00	HES-1	CTTGTG
AS$ALFIN1_06	1033 (+)	0	100.00	Alfin1	GTGGAG
RAT$GSTP_07	1035 (+)	1	87.50	NF-1A, SF-A	GGAGCAGGA
RAT$MLC_04	1035 (+)	0	100.00	MAPF2, YY1	GGAGC
HS$GMCSF_10	1035 (+)	0	100.00	NF-ATp	GGAGCC
HS$RAS1_01	1036 (-)	1	88.89	Sp1	GCTCCGCCTC
HS$TERT_03	1036 (-)	1	87.50	Sp1	CTCCGCCTC
PA$PY_09	1039 (+)	1	88.89	AP-4	CCGCAGCTGG
RAT$MLC_04	1041 (+)	0	100.00	MAPF2, YY1	GGAGC
HS$TERT_05	1041 (-)	1	87.50	Sp1	CCCAGCCCC
HS$TERT_06	1041 (-)	1	87.50	Sp1	CCCAGCCCC
RAT$ATP1A2_01	1042 (+)	1	87.50		CAGCTGGGC
YL$XPR2_04	1042 (-)	1	88.89		CGCCCACCTC
CHICK$STN1_01	1044 (-)	0	100.00	LBP-1, MyoD	CCAGC
CHICK$STN1_03	1044 (-)	0	100.00	LBP-1, NF-1 (-like proteins)	CCAGC
SV$SV40_37	1045 (+)	0	100.00		CTGGG
SV$SV40_63	1046 (+)	0	100.00	T-Ag	TGGGC
AS$ZIC_19	1046 (+)	1	87.50	Zic1	TGGGTGCGG
HS$APP_01	1047 (+)	0	100.00		GGGCGC
HS$CD11B_01	1047 (-)	0	100.00	Sp1	CGCCC
HSV1$TK_08	1048 (+)	0	100.00	ZF5	GGCGCG
HS$CDC2_09	1050 (+)	0	100.00		CGCGG
HS$CYCA_05	1050 (+)	0	100.00		CGCGG
HS$CYCD1_14	1050 (-)	0	100.00	Sp1, Sp2, Sp3, Sp4	CCGCG
Y$CHA1_01	1051 (+)	0	100.00	CHA4	GCGGA
Y$CHA1_01	1051 (-)	0	100.00	CHA4	TCCGC
AS$STAT5A_62	1056 (+)	1	87.50	STAT5A	TTCCCCGAG
AS$STAT5A_63	1056 (+)	1	87.50	STAT5A	TTCACCGAG
AS$STAT5A_67	1056 (+)	1	87.50	STAT5A	TTCGCAGAG
ASPN$AMDS_01	1056 (-)	0	100.00	AmdR, AnCF	TCGGCGAA
PA$PY_24	1062 (+)	0	100.00	T-Ag	GAGGC
PA$PY_25	1062 (+)	0	100.00	T-Ag	GAGGC
SV$SV40_02	1062 (+)	0	100.00	T-Ag	GAGGC
CHICK$ITGB3_01	1062 (+)	0	100.00	RXR-beta, VDR	GAGGCA
SV$SV40_01	1062 (-)	0	100.00	T-Ag	GCCTC
SV$SV40_02	1062 (-)	0	100.00	T-Ag	GCCTC
SV$SV40_63	1062 (-)	0	100.00	T-Ag	GCCTC
HS$M1A11_01	1063 (+)	1	87.50	ZFX	AGGCCCCGA
EBV$IR4_05	1064 (+)	0	100.00	R	GGCAC
EBV$IR4_06	1064 (+)	0	100.00	R	GGCAC
XENLA$U2SN_11	1064 (-)	0	100.00		GTGCC
AS$SPZ1_13	1069 (+)	1	87.50	Spz1	CGAGGAACT
PA$PY_24	1070 (+)	0	100.00	T-Ag	GAGGC
PA$PY_25	1070 (+)	0	100.00	T-Ag	GAGGC
SV$SV40_02	1070 (+)	0	100.00	T-Ag	GAGGC
CHICK$ITGB3_01	1070 (+)	0	100.00	RXR-beta, VDR	GAGGCA
SV$SV40_01	1070 (-)	0	100.00	T-Ag	GCCTC
SV$SV40_02	1070 (-)	0	100.00	T-Ag	GCCTC
SV$SV40_63	1070 (-)	0	100.00	T-Ag	GCCTC
EBV$IR4_05	1072 (+)	0	100.00	R	GGCAC
EBV$IR4_06	1072 (+)	0	100.00	R	GGCAC
RAT$MT1_06	1072 (-)	1	87.50		CTGAGTGCA
XENLA$U2SN_11	1072 (-)	0	100.00		GTGCC
DROME$UBX_03	1074 (+)	0	100.00	Zeste	CACTCA
DROME$UBX_04	1074 (+)	0	100.00	Zeste	CACTCA
AS$WZF1_01	1074 (+)	0	100.00	WZF1	CACTC
DROME$WHLO_03	1074 (+)	0	100.00	Zeste	CACTCA
DROME$WHLO_06	1074 (+)	0	100.00	Zeste	CACTCA
DROME$WHLO_07	1074 (+)	0	100.00	Zeste	CACTCA
DROME$WHLO_08	1074 (+)	0	100.00	Zeste	CACTCA
DROME$WHLO_09	1074 (+)	0	100.00	Zeste	CACTCA
DROME$DPP_02	1074 (+)	0	100.00	Zeste	CACTCA
DROME$DPP_04	1074 (+)	0	100.00	Zeste	CACTCA
WHEAT$H3_02	1074 (+)	0	100.00	WZF1	CACTC
WHEAT$H3_03	1074 (+)	0	100.00	WZF1	CACTC
Y$ILV1_02	1074 (-)	0	100.00	GCN4	TGAGTG
DROME$WHLO_02	1074 (-)	0	100.00	Zeste	TGAGTG
DROME$WHLO_05	1074 (-)	0	100.00	Zeste	TGAGTG
DROME$DPP_03	1074 (-)	0	100.00	Zeste	TGAGTG
RAT$POMC_03	1078 (+)	0	100.00	GR	CAGAG
PA$PY_12	1079 (+)	0	100.00		AGAGG
HS$TCRA_01	1079 (-)	1	88.89	c-Ets-1	CACATCCTCT
CHICK$BAG_07	1080 (+)	1	87.50		GAGGAGGGG
HS$AACS_02	1081 (+)	0	100.00	ARP-1	AGGAGG
HT1$HTLV1_08	1082 (-)	0	100.00	c-Ets-1 68	CCTCC
HS$RARG_01	1084 (+)	0	100.00	RAR-alpha1, RAR-beta, RAR-gamma, RXR-alpha	AGGTGA
HSV1$TK_12	1084 (+)	0	100.00	RAR-alpha1, T3R-alpha	AGGTGA
MOUSE$IBABP_01	1084 (+)	0	100.00	FXR, RXR-alpha	AGGTGA
AS$ERR1_16	1084 (+)	0	100.00	ERR1	AGGTGA
RAT$ME_08	1084 (+)	0	100.00	PPAR-gamma, RXR-gamma	AGGTGA
HS$CAMHC_01	1084 (-)	0	100.00	Hp55, Hp65, p58, RAR-alpha1, RXR-beta, T3R-alpha, T3R-beta1, T3R-beta2	TCACCT
HS$CYP24_02	1084 (-)	0	100.00	VDR	TCACCT
RAT$CYP24_01	1084 (-)	0	100.00	VDR	TCACCT
HS$PR264_01	1085 (+)	0	100.00	c-Myb	GGTGAG
HS$APOA2_06	1085 (-)	0	100.00		TCACC
Y$HIS7_01	1086 (-)	1	87.50	ABF1	CTCTCTCTC
DROME$EVE_08	1088 (+)	0	100.00	GAGA factor	GAGAG
DROME$EVE_13	1088 (+)	0	100.00	GAGA factor	GAGAG
DROME$FTZ_32	1088 (+)	0	100.00	GAGA factor	GAGAG
AS$GAGA_03	1088 (+)	0	100.00	GAGA factor	GAGAG
DROME$EVE_09	1088 (-)	0	100.00	GAGA factor	CTCTC
DROME$FTZ_32	1088 (-)	0	100.00	GAGA factor	CTCTC
HS$FREAC4_01	1088 (-)	1	87.50	WT1	CGCCCTCTC
Y$HIS7_01	1088 (-)	1	87.50	ABF1	CTCTCTCTC
DROME$EVE_08	1090 (+)	0	100.00	GAGA factor	GAGAG
DROME$EVE_13	1090 (+)	0	100.00	GAGA factor	GAGAG
DROME$FTZ_32	1090 (+)	0	100.00	GAGA factor	GAGAG
AS$GAGA_03	1090 (+)	0	100.00	GAGA factor	GAGAG
DROME$EVE_09	1090 (-)	0	100.00	GAGA factor	CTCTC
DROME$FTZ_32	1090 (-)	0	100.00	GAGA factor	CTCTC
RICE$RAB16A_02	1091 (-)	1	88.89		CCGCCGCGCT
AS$ALFIN1_01	1092 (+)	1	87.50	Alfin1	GAGCGGTGG
DROME$EVE_04	1092 (-)	0	100.00	GAGA factor	CGCTC
DROME$EVE_08	1092 (-)	0	100.00	GAGA factor	CGCTC
DROME$EVE_10	1092 (-)	0	100.00	GAGA factor	CGCTC
CHICK$BLCR_02	1094 (+)	0	100.00	NF-E4, Sp1	GCGGC
MOUSE$CEBPA_02	1095 (-)	0	100.00	CUP	GCCGCCG
HS$CDC25C_04	1096 (+)	0	100.00		GGCGG
AS$GCCBOX_01	1096 (-)	0	100.00	ERF2, ERF3, ERF4	GCCGCC
AS$GCCBOX_02	1096 (-)	0	100.00	Pti4	GCCGCC
CHICK$BLCR_02	1097 (+)	0	100.00	NF-E4, Sp1	GCGGC

Number of sites found: 2619

Start a new search

P27 – CDKN1B
JASPAR RARA/RXRA MOTIF

Access EPDnew
- *H. sapiens*
- *H. sapiens non-coding*
- *M. mulatta*
- *M. musculus*
- *M. musculus non-coding*
- *R. norvegicus*
- *C. familiaris*
- *G. gallus*
- *D. melanogaster*
- *A. mellifera*
- *D. rerio*
- *C. elegans*
- *A. thaliana*
- *Z. mays*
- *S. cerevisiae*
- *S. pombe*
- *P. falciparum*
- Select / Download
- Promoter analysis tools
- FTP site

Access EPD
- Promoter elements
- Select / Download
- FTP site

Access MGA Database
- MGA-Search
- MGA Data Overview
- MGA FTP site

Documents
Other Resources
References
What is new
Contact us

in H. sapiens SEARCH

Please help us make EPD better! Take our **short survey** to suggest new tools or organism covered. It will take only a few minutes.

General Information

[Help]

Promoter ID:	**CDKN1B_1**
Promoter type:	region
Organism:	Homo sapiens (Human)
Gene Symbol:	CDKN1B
Description of the gene:	cyclin dependent kinase inhibitor 1B
Sequence:	tctcctcctctgtttaaatagactcgccgtgtcaatcattttcttcttcGTCAGCCTCCC
Position in the genome:	Chromosome [NC_000012.12]; Strand [+]; Position [12717375]
Ensembl:	ENSG00000111276
RefSeq:	NM_004064
NCBI Gene:	CDKN1B
GeneCards:	CDKN1B

Promoter Image [View it at UCSC]

[Help]

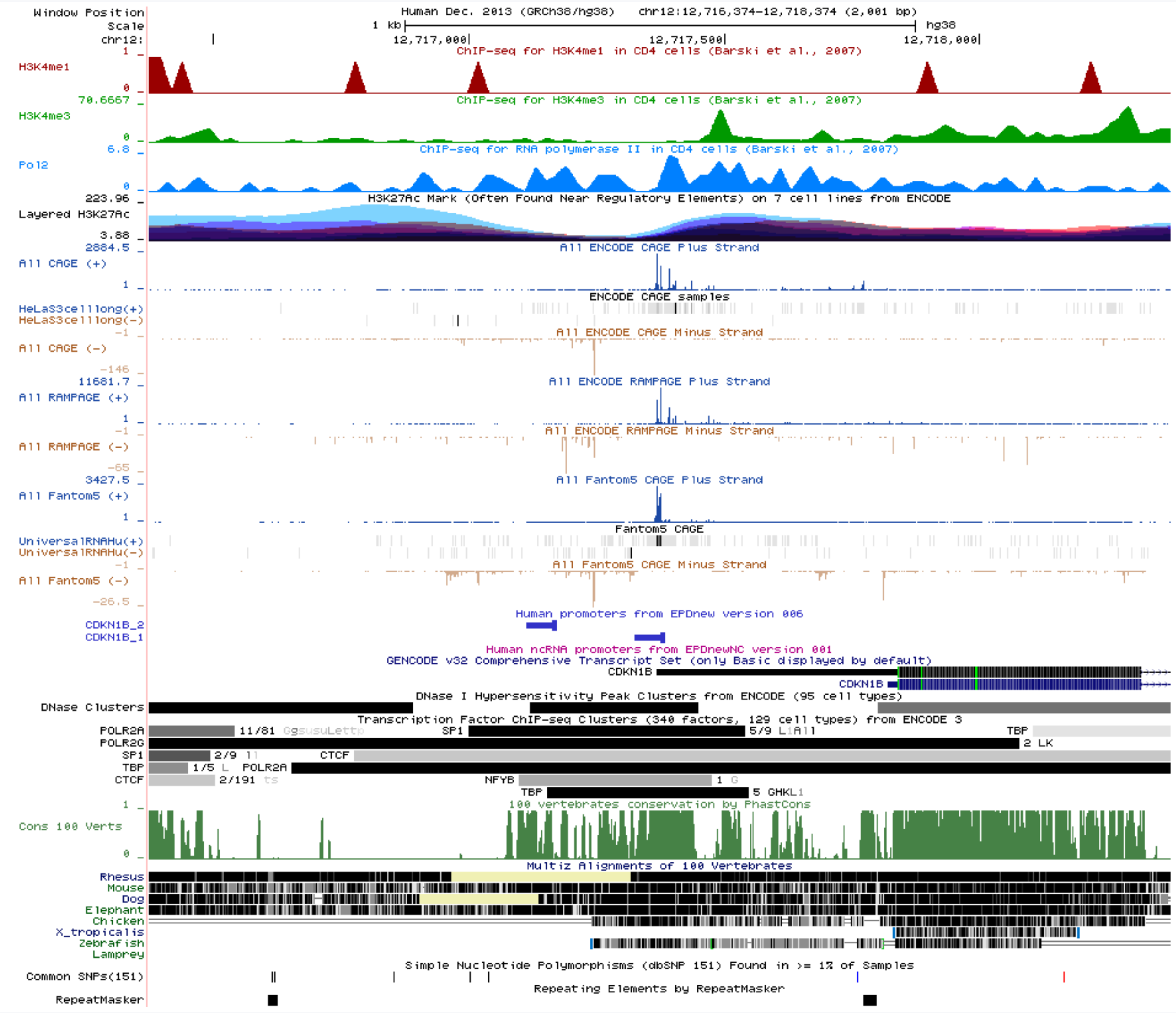

Other viewers

[Help]

EPD view at UCSC (hg38):	chr12:12715374–12719374 (European mirror)
EPD view at UCSC (hg19):	chr12:12868308–12872308 (European mirror)
SwissRegulon (hg19):	chr12:12868309–12872309
FANTOM5 (hg38):	chr12:12715375–12719375
DBTSS/KERO (hg38):	chr12:12715375–12719375
refTSS (hg38):	chr12:12715374–12719374 (European mirror)

Selective viewer showing the 3 Fantom5 tissues with the highest expression of **CDKN1B_1** (European mirror)

GM12878 viewer (European mirror)

Sequence Retrieval Tool

[Help]

Get sequence CDKN1B_1 **from** -1000 **to** 100 bp relative to TSS GET SEQUENCE

lower case upstream TSS

```
>FP017363 CDKN1B_1 :+U EU:NC; range -1000 to 100.
AGGAAGGAAGGAGCTGTTGTATTTGGCGGCTGGACTCAGGTAGAGGAAACTGCTACAATC
CCGGGAAAGAACAGAAAAGTAGAAAGGACGAGTTCCCACACGCAGCCAATGTCCATGGC
CTTAACTGTGCTTGGGAAGGAAGATCCTGGGCCAGGGGTGTACCCTCGTTTTTCAAAACT
AAACGTGTCTGAGACAGCTACAAAGTTTATTAAGGGACTTGAGAGACTAGAGTTTTTTGT
TTTTTTTTTTTAATCTTGAGTTCCTTTCTTATTTTCATTGAGGGAGAGCTTGAGTTCATG
ATAAGTGCCGCGTCTACTCCTGGCTAATTTCTAAAAGAAAGACGTTCGCTTTGGCTTCTT
CCCTAGGCCCCCAGCCTCCCCAGGGATGGCAGAAACTTCTGGGTTAAGGCTGAGCGAACC
ATTGCCCACTGCCTCCACCAGCCCCCAGCAAAGGCACGCCGGCGGGGGGGCGCCCAGCCC
CCCCAGCAAACGCTCCGCGGCCTCCCCCGCAGACCACGAGGTGGGGGCCGCTGGGGAGGG
CCGAGCTGGGGGCAGCTCGCCACCCCGGCTCCTAGCGAGCTGCCGGCGACCTTCGCGGTC
CTCTGGTCCAGGTCCCGGCTTCCCGGGCGAGGAGCGGGAGGGAGGTCGGGGCTTAGGCGC
CGCGGCGAACCCGCCAACGCAGCGCCGGGCCCCGAACCTCAGGCCCCGCCCCAGGTTCCC
GGCCGTTTGGCTAGTTTGTTTGTCTTAATTTTAATTTCTCCGAGGCCAGCCAGAGCAGGT
TTGTTGGCAGCAGTACCCCTCCAGCAGTCACGCGACCAGCCAATCTCCCGGCGGCGCTCG
GGGAGGCGGCGCGCTCGGGAACGAGGGGAGGTGGCGGAACCGCGCCGGGGCCACCTTAAG
GCCGCGCTCGCCAGCCTCGGCGGGGCGGCTCCCGCCGCCGCAACCAATGGATCTCCTCCT
CTGTTTAAATAGACTCGCCGTGTCAATCATTTTCTTCTTCGTCAGCCTCCCTTCCACCGC
CATATTGGGCCACTAAAAAAAGGGGGCTCGTCTTTTCGGGGTGTTTTTCTCCCCCTCCCC
TGTCCCCGCTTGCTCACGGCT
```

Search Motif Tool

[Help]

Library: Transcription Factor Motifs (JASPAR Motif: RARA::RXRA

From: -1000 To: 100 bp relative to TSS and a cut-off (p-value) of: 0.001 SEARCH

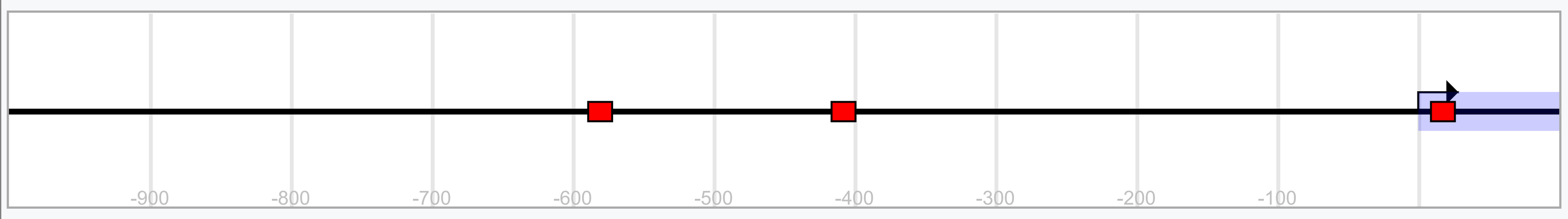

RARA::RXRA [p-value = 0.001]: -589, -417, 9

Expression Profile Tool

[Help]

CDKN1B_1 is expressed in **1035 samples** with an **average expression of 187.178 tags** per 10M.

The TSSs distribution around the EPDnew annotated start site (click on the histogram bars to explore samples):

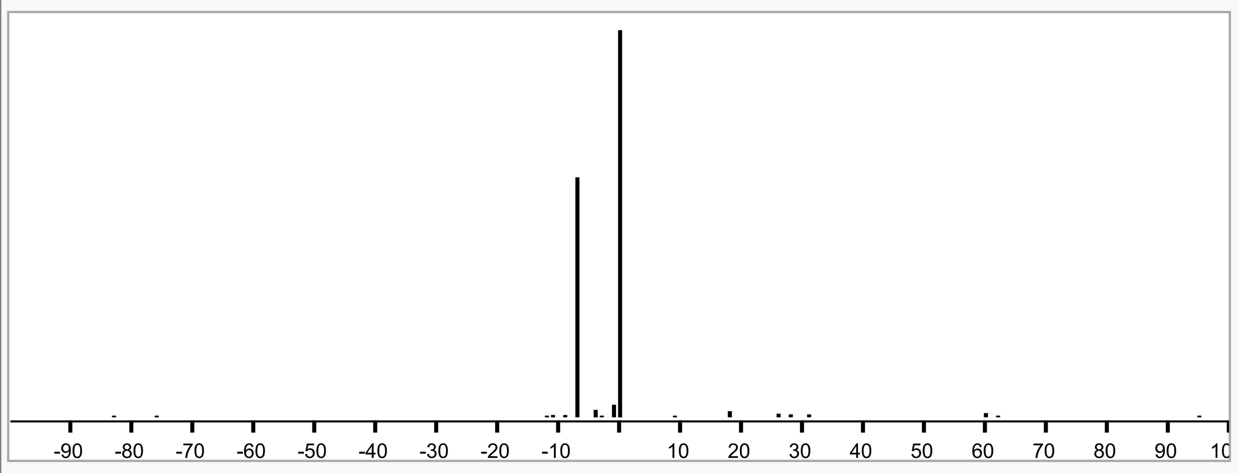

Last update October 2019

P27 – CDKN1B
JASPAR RARA/RXRG MOTIF

Access EPDnew
- *H. sapiens*
- *H. sapiens non-coding*
- *M. mulatta*
- *M. musculus*
- *M. musculus non-coding*
- *R. norvegicus*
- *C. familiaris*
- *G. gallus*
- *D. melanogaster*
- *A. mellifera*
- *D. rerio*
- *C. elegans*
- *A. thaliana*
- *Z. mays*
- *S. cerevisiae*
- *S. pombe*
- *P. falciparum*
- Select / Download
- Promoter analysis tools
- FTP site

Access EPD
- Promoter elements
- Select / Download
- FTP site

Access MGA Database
- MGA-Search
- MGA Data Overview
- MGA FTP site

Documents
Other Resources
References
What is new
Contact us

in H. sapiens SEARCH

Please help us make EPD better! Take our **short survey** to suggest new tools or organism covered. It will take only a few minutes.

General Information

[Help]

Promoter ID:	**CDKN2A_1**
Promoter type:	region
Organism:	Homo sapiens (Human)
Gene Symbol:	CDKN2A
Description of the gene:	cyclin dependent kinase inhibitor 2A
Sequence:	accagagggtggggcggaccgcgtgcgctcggcggctgcggagagggggAGAGCAGGCAG
Position in the genome:	Chromosome [NC_000009.12]; Strand [-]; Position [21974856]
RefSeq:	NM_058195
NCBI Gene:	CDKN2A
GeneCards:	CDKN2A

Promoter Image [View it at UCSC]

[Help]

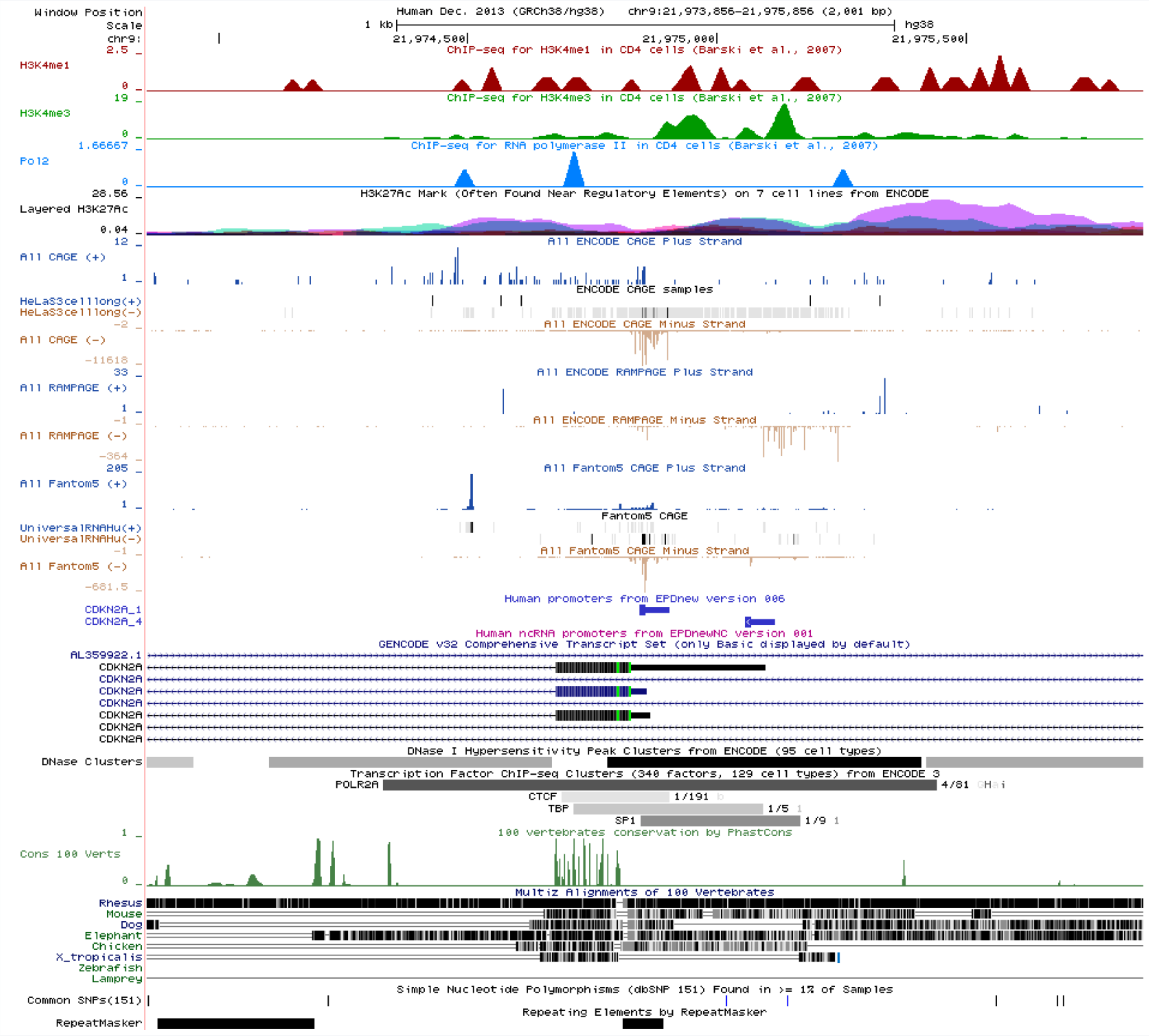

Other viewers

[Help]

EPD view at UCSC (hg38):	chr9:21972856–21976856 (European mirror)
EPD view at UCSC (hg19):	chr9:21972855–21976855 (European mirror)
SwissRegulon (hg19):	chr9:21972855–21976855
FANTOM5 (hg38):	chr9:21972856–21976856
DBTSS/KERO (hg38):	chr9:21972856–21976856
refTSS (hg38):	chr9:21972856–21976856 (European mirror)

Selective viewer showing the 3 Fantom5 tissues with the highest expression of **CDKN2A_1** (European mirror)
GM12878 viewer (European mirror)

Sequence Retrieval Tool

[Help]

Get sequence CDKN2A_1 **from** -1000 **to** 100 bp relative to TSS GET SEQUENCE
☐ lower case upstream TSS

```
>FP013138 CDKN2A_1 :+U EU:NC; range -1000 to 100.
GTGTGAAGGAGACAGGACAGTATTTGAAGCTGGTCTTTGGATCACTGTGCAACTCTGCTT
CTAGAACACTGAGCACTTTTTCTGGTCTAGGAATTATGACTTTGAGAATGGAGTCCGTCC
TTCCAATGACTCCCTCCCCATTTTCCTATCTGCCTACAGGCAGAATTCTCCCCCGTCCGT
ATTAAATAAACCTCATCTTTTCAGAGTCTGCTCTTATACCAGGCAATGTACACGTCTGAG
AAACCCTTGCCCCAGACAGCCGTTTTACACGCAGGAGGGGAAGGGGAGGGGAAGGAGAGA
GCAGTCCGACTCTCCAAAAGGAATCCTTTGAACTAGGGTTTCTGACTTAGTGAACCCCGC
GCTCCTGAAAATCAAGGGTTGAGGGGGTAGGGGGACACTTTCTAGTCGTACAGGTGATTT
CGATTCTCGGTGGGGCTCTCACAACTAGGAAAGAATAGTTTTGCTTTTTCTTATGATTAA
AAGAAGAAGCCATACTTTCCCTATGACACCAAACACCCCGATTCAATTTGGCAGTTAGGA
AGGTTGTATCGCGGAGGAAGGAAACGGGGCGGGGGCGGATTTCTTTTTAACAGAGTGAAC
GCACTCAAACACGCCTTTGCTGGCAGGCGGGGGAGCGCGGCTGGGAGCAGGGAGGCCGGA
GGGCGGTGTGGGGGGCAGGTGGGGAGGAGCCCAGTCCTCCTTCCTTGCCAACGCTGGCTC
TGGCGAGGGCTGCTTCCGGCTGGTGCCCCCGGGGGAGACCCAACCTGGGGCGACTTCAGG
GGTGCCACATTCGCTAAGTGCTCGGAGTTAATAGCACCTCCTCCGAGCACTCGCTCACGG
CGTCCCCTTGCCTGGAAAGATACCGCGGTCCCTCCAGAGGATTTGAGGGACAGGGTCGGA
GGGGGCTCTTCCGCCAGCACCGGAGGAAGAAAGAGGAGGGGCTGGCTGGTCACCAGAGGG
TGGGGCGGACCGCGTGCGCTCGGCGGCTGCGGAGAGGGGGAGAGCAGGCAGCGGGCGGCG
GGGAGCAGCATGGAGCCGGCGGCGGGGAGCAGCATGGAGCCTTCGGCTGACTGGCTGGCC
ACGGCCGCGGCCCGGGGTCGG
```

Search Motif Tool

[Help]

Library: Transcription Factor Motifs (JASPAR Motif: RARA::RXRG

From: -1000 To: 100 bp relative to TSS and a cut-off (p-value) of: 0.001 SEARCH

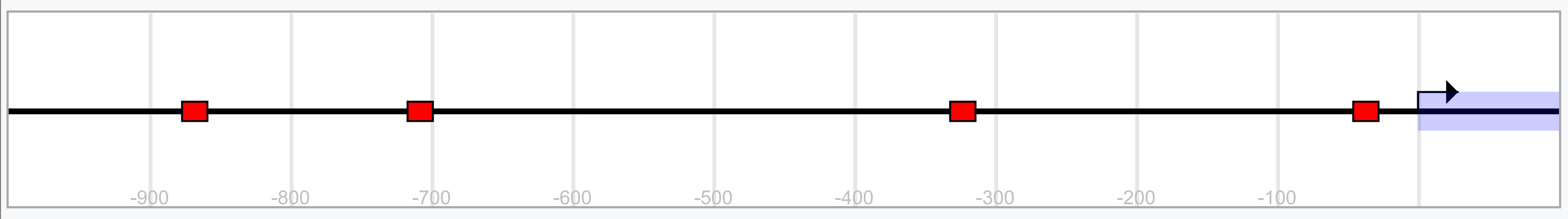

RARA::RXRG [p-value = 0.001]: -46, -332, -717, -877

Expression Profile Tool

[Help]

CDKN2A_1 is expressed in **517 samples** with an **average expression of 198.985 tags** per 10M.

The TSSs distribution around the EPDnew annotated start site (click on the histogram bars to explore samples):

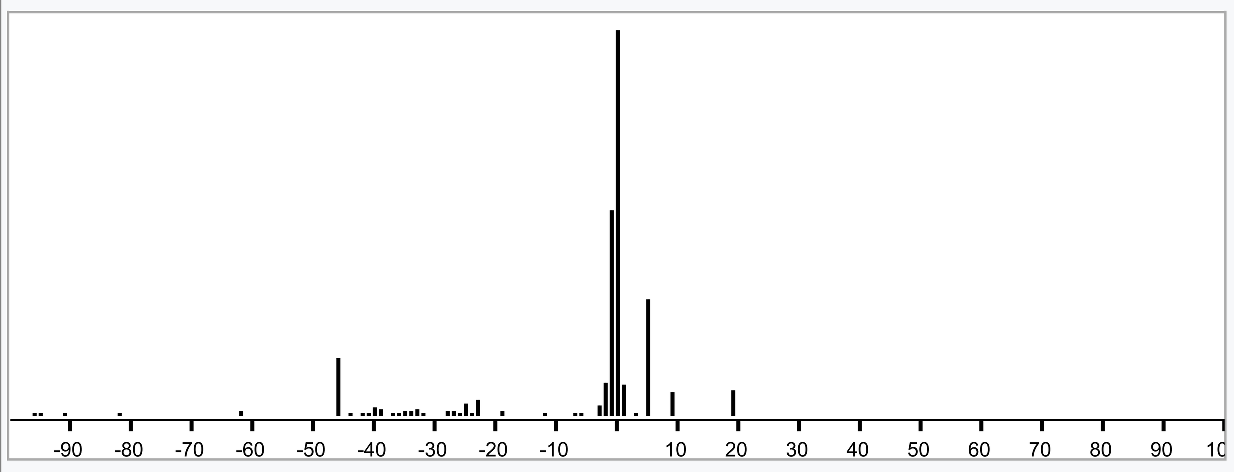

Last update October 2019

P27 – CDKN1B
JASPAR RARA MOTIF

in H. sapiens SEARCH

Access EPDnew
- H. sapiens
- H. sapiens non-coding
- M. mulatta
- M. musculus
- M. musculus non-coding
- R. norvegicus
- C. familiaris
- G. gallus
- D. melanogaster
- A. mellifera
- D. rerio
- C. elegans
- A. thaliana
- Z. mays
- S. cerevisiae
- S. pombe
- P. falciparum
- Select / Download
- Promoter analysis tools
- FTP site

Access EPD
- Promoter elements
- Select / Download
- FTP site

Access MGA Database
- MGA-Search
- MGA Data Overview
- MGA FTP site

Documents
Other Resources
References
What is new
Contact us

Please help us make EPD better! Take our short survey to suggest new tools or organism covered. It will take only a few minutes.

General Information

[Help]

Promoter ID:	**CDKN2A_1**
Promoter type:	region
Organism:	Homo sapiens (Human)
Gene Symbol:	CDKN2A
Description of the gene:	cyclin dependent kinase inhibitor 2A
Sequence:	accagagggtggggcggaccgcgtgcgctcggcggctgcggagagggggAGAGCAGGCAG
Position in the genome:	Chromosome [NC_000009.12]; Strand [-]; Position [21974856]
RefSeq:	NM_058195
NCBI Gene:	CDKN2A
GeneCards:	CDKN2A

Promoter Image [View it at UCSC]

[Help]

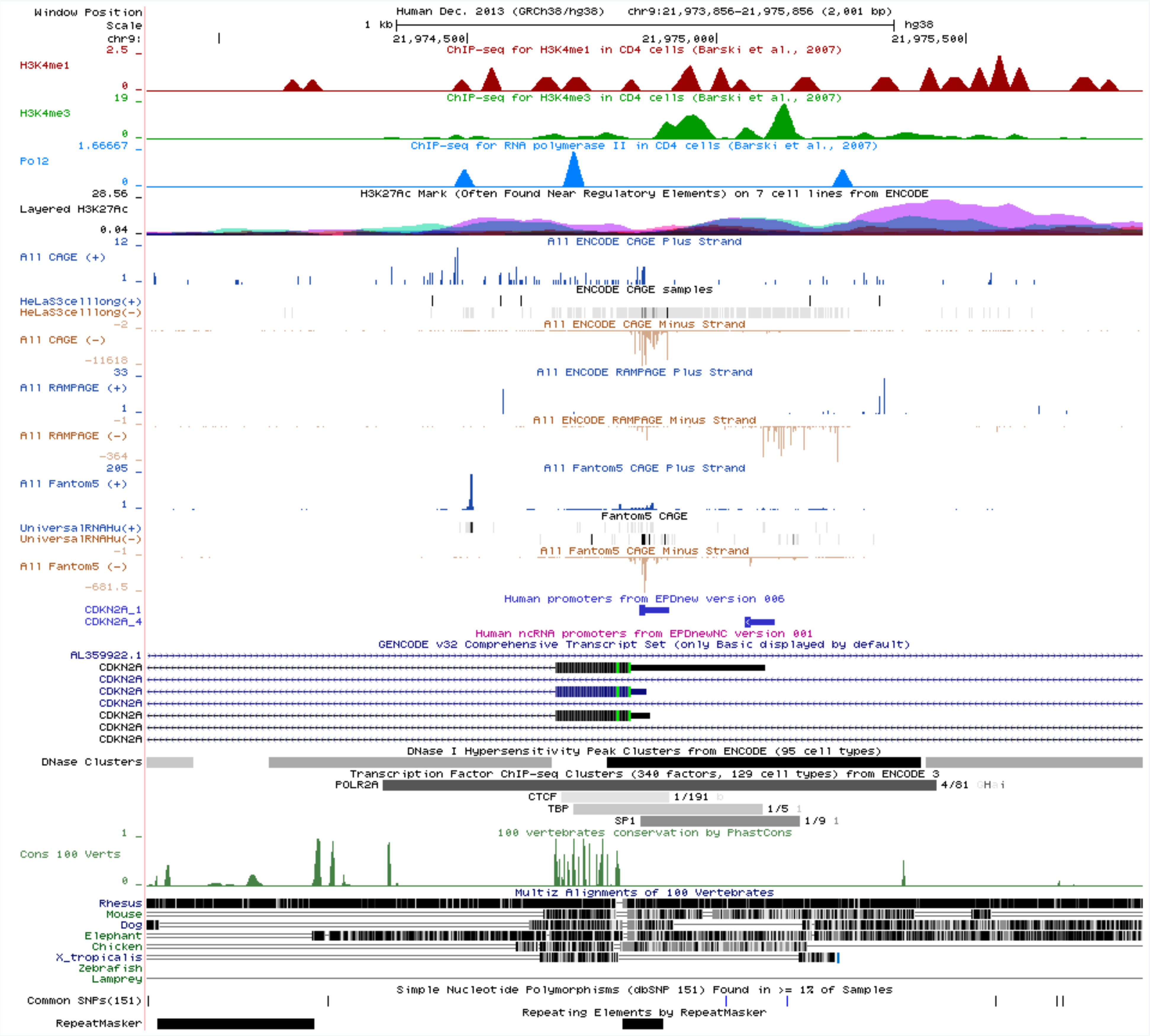

Other viewers

[Help]

EPD view at UCSC (hg38):	chr9:21972856–21976856 (European mirror)
EPD view at UCSC (hg19):	chr9:21972855–21976855 (European mirror)
SwissRegulon (hg19):	chr9:21972855–21976855
FANTOM5 (hg38):	chr9:21972856–21976856
DBTSS/KERO (hg38):	chr9:21972856–21976856
refTSS (hg38):	chr9:21972856–21976856 (European mirror)

Selective viewer showing the 3 Fantom5 tissues with the highest expression of **CDKN2A_1** (European mirror)
GM12878 viewer (European mirror)

Sequence Retrieval Tool

[Help]

Get sequence CDKN2A_1 **from** -1000 **to** 100 bp relative to TSS GET SEQUENCE
lower case upstream TSS

```
>FP013138 CDKN2A_1 :+U EU:NC; range -1000 to 100.
GTGTGAAGGAGACAGGACAGTATTTGAAGCTGGTCTTTGGATCACTGTGCAACTCTGCTT
CTAGAACACTGAGCACTTTTTCTGGTCTAGGAATTATGACTTTGAGAATGGAGTCCGTCC
TTCCAATGACTCCCTCCCCATTTTCCTATCTGCCTACAGGCAGAATTCTCCCCCGTCCGT
ATTAAATAAACCTCATCTTTTCAGAGTCTGCTCTTATACCAGGCAATGTACACGTCTGAG
AAACCCTTGCCCCAGACAGCCGTTTTACACGCAGGAGGGGAAGGGGAGGGGAAGGAGAGA
GCAGTCCGACTCTCCAAAAGGAATCCTTTGAACTAGGGTTTCTGACTTAGTGAACCCCGC
GCTCCTGAAAATCAAGGGTTGAGGGGGTAGGGGACACTTTCTAGTCGTACAGGTGATTT
CGATTCTCGGTGGGGCTCTCACAACTAGGAAAGAATAGTTTTGCTTTTTCTTATGATTAA
AAGAAGAAGCCATACTTTCCCTATGACACCAAACACCCCGATTCAATTTGGCAGTTAGGA
AGGTTGTATCGCGGAGGAAGGAAACGGGGCGGGGGCGGATTTCTTTTTAACAGAGTGAAC
GCACTCAAACACGCCTTTGCTGGCAGGCGGGGGAGCGCGGCTGGGAGCAGGGAGGCCGGA
GGGCGGTGTGGGGGGCAGGTGGGGAGGAGCCCAGTCCTCCTTCCTTGCCAACGCTGGCTC
TGGCGAGGGCTGCTTCCGGCTGGTGCCCCCGGGGGAGACCCAACCTGGGGCGACTTCAGG
GGTGCCACATTCGCTAAGTGCTCGGAGTTAATAGCACCTCCTCCGAGCACTCGCTCACGG
CGTCCCCTTGCCTGGAAAGATACCGCGGTCCCTCCAGAGGATTTGAGGGACAGGGTCGGA
GGGGGCTCTTCCGCCAGCACCGGAGGAAGAAAGAGGAGGGGCTGGCTGGTCACCAGAGGG
TGGGGCGGACCGCGTGCGCTCGGCGGCTGCGGAGAGGGGGAGAGCAGGCAGCGGGCGGCG
GGGAGCAGCATGGAGCCGGCGGCGGGGAGCAGCATGGAGCCTTCGGCTGACTGGCTGGCC
ACGGCCGCGGCCCGGGGTCGG
```

Search Motif Tool

[Help]

Library: Transcription Factor Motifs (JASPAR Motif: RARA
From: -1000 To: 100 bp relative to TSS and a cut-off (p-value) of: 0.001 SEARCH

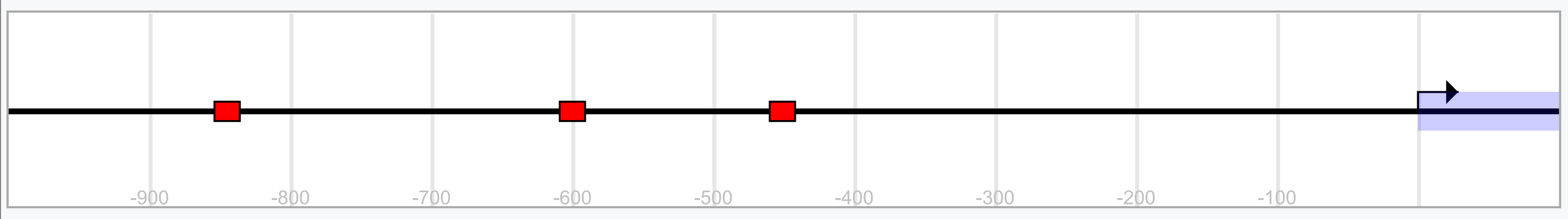

RARA [p-value = 0.001]: -460, -609, -854

Expression Profile Tool

[Help]

CDKN2A_1 is expressed in **517 samples** with an **average expression of 198.985 tags** per 10M.

The TSSs distribution around the EPDnew annotated start site (click on the histogram bars to explore samples):

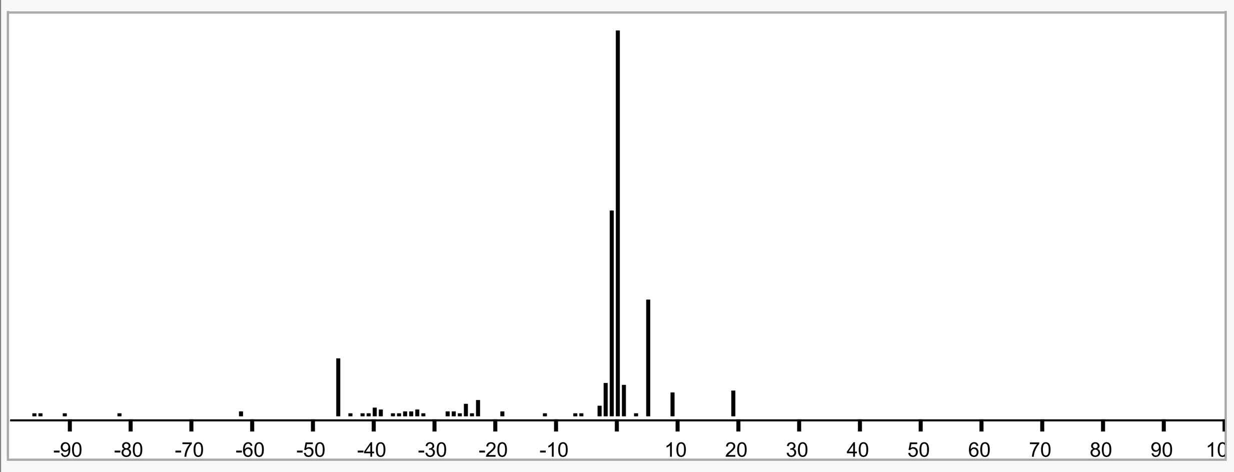

P27
PATCH RARE MOTIFS

Thank you for using Patch at Host - It's Fri, 16.4.2021 - 12:45 MEZ

Start a new search

Name of your search:	default
Name of your sequence:	default.seq
Sites searched for:	all
Maximum number of mismatches:	2
Mismatch penalty:	100
Lower score boundary:	87.5

Scanning sequence: FP017363 CDKN1B_1 :+U EU:NC; range -1000 to 100.
View a graphic of the following search results

Identifier	Position	Mismatches	Score	Binding Factor	Sequence (Search Pattern)
PEA3$CONS	1 (+)	0	91.67	c-Ets-1 54, c-Ets-1 68, c-Ets-2 58-64, PEA3	AGGAAR
DROME$E74_10	1 (+)	0	100.00	E74A	AGGAA
DROME$E74_11	1 (+)	0	100.00	E74A	AGGAA
E74A$CONS_01	1 (+)	0	90.00	E74A	MGGAA
MOUSE$NE_01	1 (+)	1	88.89	PU.1	AGGGAGGAAG
MOUSE$RPL32_01	2 (+)	0	100.00	f(alpha)-f(epsilon), HrpF, XrpFI	GGAAG
HS$TNFA_02	2 (+)	0	100.00		GGAAG
HS$TNFA_03	2 (+)	0	100.00		GGAAG
XENLA$RPL14_01	2 (-)	0	100.00	HrpF, XrpFI	CTTCC
MOUSE$SURF2_04	2 (-)	1	90.91		CTGCTTCCTTCC
HS$DG_03	2 (-)	0	100.00		CCTTCCTTCC
Y$TPI_01	2 (-)	0	100.00	GCR1	CTTCC
MOUSE$M1H2KB_11	3 (+)	0	100.00		GAAGGAA
HS$CDC2_01	4 (+)	0	100.00	c-Ets-2	AAGGAA
HS$CDC2_02	4 (-)	0	100.00	c-Ets-2	TTCCTT
PEA3$CONS	5 (+)	0	91.67	c-Ets-1 54, c-Ets-1 68, c-Ets-2 58-64, PEA3	AGGAAR
DROME$E74_10	5 (+)	0	100.00	E74A	AGGAA
DROME$E74_11	5 (+)	0	100.00	E74A	AGGAA
E74A$CONS_01	5 (+)	0	90.00	E74A	MGGAA
MOUSE$RPL32_01	6 (+)	0	100.00	f(alpha)-f(epsilon), HrpF, XrpFI	GGAAG
HS$TNFA_02	6 (+)	0	100.00		GGAAG
HS$TNFA_03	6 (+)	0	100.00		GGAAG
XENLA$RPL14_01	6 (-)	0	100.00	HrpF, XrpFI	CTTCC
Y$TPI_01	6 (-)	0	100.00	GCR1	CTTCC
Y$GLK1_01	7 (+)	0	100.00	GCR1	GAAGGAG
RAT$MLC_04	10 (+)	0	100.00	MAPF2, YY1	GGAGC
HS$TCRBL_09	17 (-)	0	100.00		AATACAA
Y$ENO1_01	20 (+)	0	100.00	RAP1	TATTTGG
Y$ENO1_02	20 (+)	0	100.00	EBF1, RAP1	TATTTGG
HS$MSH2_01	20 (+)	0	100.00	HNF-3alpha, HNF-3B	TATTT
HS$IGH_04	21 (+)	0	100.00		ATTTG
HS$EGFR_14	21 (-)	0	100.00		CAAAT
MAIZE$PMS1_01	21 (-)	0	100.00		CAAAT
AS$E2F_10	22 (+)	0	100.00	E2F-5	TTTGGCGG
HS$P107_02	22 (+)	0	100.00	E2F	TTTGGCG
MOUSE$POLA2_02	22 (+)	1	87.50	E2F-1	TTTGGCGGG
HS$CDC6_01	22 (+)	0	100.00	E2F	TTTGGCGG
MOUSE$AP2_07	23 (+)	0	100.00	NF-1	TTGGC
Y$HOP1_01	23 (+)	1	87.50		TGGGCGGCT
Y$CAR1_01	23 (-)	1	87.50	repressor of CAR1 expression	AGCCGCCGA
Y$TOP1_01	23 (-)	1	87.50	repressor of CAR1 expression	AGCCGCCGA
Y$PYK1_02	23 (-)	1	87.50	repressor of CAR1 expression	AGCCGCCAT
Y$CAR2_01	23 (-)	1	87.50	repressor of CAR1 expression	AGCCGCCGA
Y$CYB2_01	23 (-)	1	87.50	repressor of CAR1 expression	AACCGCCAA
Y$HSF1_01	23 (-)	0	100.00	repressor of CAR1 expression	AGCCGCCAA
HS$CDC25C_04	25 (+)	0	100.00		GGCGG
NT$PRB1B_01	25 (+)	0	100.00	EBP	GGCGGCT
AS$GCCBOX_01	25 (-)	0	100.00	ERF2, ERF3, ERF4	GCCGCC
AS$GCCBOX_02	25 (-)	0	100.00	Pti4	GCCGCC
CHICK$BLCR_02	26 (+)	0	100.00	NF-E4, Sp1	GCGGC
QUAIL$STN1_02	28 (-)	0	100.00		CCAGCC
CHICK$STN1_01	29 (-)	0	100.00	LBP-1, MyoD	CCAGC
CHICK$STN1_03	29 (-)	0	100.00	LBP-1, NF-1 (-like proteins)	CCAGC
CHICK$CA2_02	31 (+)	0	100.00	RXR-alpha, VDR	TGGACT
RAT$MT1_06	31 (-)	1	87.50		CTGAGTGCA
RAT$PEPCK_17	31 (-)	0	100.00	GR	AGTCCA
AS$TR_34	31 (-)	0	100.00	T3R-alpha1, T3R-alpha2	AGTCCA
DROME$WHLO_11	33 (-)	0	100.00	Zeste	TGAGTC
HS$GMCSF_01	36 (+)	0	100.00	NF-GMa, NF-GMb	TCAGGTA
HS$GMCSF_02	36 (+)	0	100.00	CD28RC, NF-GMa	TCAGGTA
NFGMB$CONS	36 (+)	0	92.86	NF-GMb	TCAGRTA
MOUSE$IGLL_02	36 (+)	1	87.50	LyF-1	TCAGGGAGA
AS$AREB6_08	36 (-)	1	87.50	AREB6	TGTACCTGA
AS$AREB6_10	36 (-)	1	87.50	AREB6	TTTACCTGA
HS$VEGF_02	37 (+)	0	100.00		CAGGT
TO$E4_02	39 (-)	0	100.00		CTACC
PA$PY_12	42 (+)	0	100.00		AGAGG
MOUSE$KROX_01	43 (-)	0	100.00	Elk-1, SAP-1a, SAP-1b, SRF	TTCCTC
MOUSE$FCGR3A_02	43 (-)	0	100.00	PU.1	TTCCTC
HS$GMCSF_09	43 (-)	0	100.00	NF-ATp, NFAT-1	TTTCCTC
MOUSE$GMCSF_06	43 (-)	0	100.00	NFAT-1	TTTCCTC
MOUSE$UPA_01	44 (+)	0	100.00	PEA3	AGGAAA
PEA3$CONS	44 (+)	0	91.67	c-Ets-1 54, c-Ets-1 68, c-Ets-2 58-64, PEA3	AGGAAR
DROME$E74_10	44 (+)	0	100.00	E74A	AGGAA
DROME$E74_11	44 (+)	0	100.00	E74A	AGGAA
E74A$CONS_01	44 (+)	0	90.00	E74A	MGGAA
HS$ADH2_09	44 (+)	0	100.00	GR	AGGAAA
HS$IFI616_01	45 (+)	0	100.00	ISGF-3	GGAAA
MOUSE$IL5_02	45 (+)	0	100.00	NF-ATc, NF-ATp, NF-ATx	GGAAA
HS$IL4_01	45 (+)	0	100.00		GGAAA
MOUSE$IL4_06	45 (+)	0	100.00		GGAAA
MOUSE$IL4_07	45 (+)	0	100.00		GGAAA
MOUSE$IL4_08	45 (+)	0	100.00		GGAAA
HS$IL5_02	45 (+)	0	100.00		GGAAA
HS$IL13_01	45 (+)	0	100.00		GGAAA
MOUSE$CD40L_01	45 (+)	0	100.00		GGAAA
MOUSE$CD40L_02	45 (+)	0	100.00		GGAAA
MOUSE$CD40L_03	45 (+)	0	100.00		GGAAA
MOUSE$CD40L_04	45 (+)	0	100.00		GGAAA
HS$CD40L_01	45 (+)	0	100.00		GGAAA
HS$CD40L_02	45 (+)	0	100.00		GGAAA
HS$IFNG_03	45 (+)	0	100.00		GGAAA
HS$IFNG_04	45 (+)	0	100.00		GGAAA
HS$TNFA_05	45 (+)	0	100.00		GGAAA
MOUSE$MCP3_01	45 (+)	0	100.00		GGAAA
HS$TNFSF6_01	45 (+)	0	100.00		GGAAA
AS$AREB6_44	46 (-)	0	100.00	AREB6	GTTTC
AS$AREB6_46	46 (-)	0	100.00	AREB6	GTTTC
AS$AREB6_48	46 (-)	0	100.00	AREB6	GTTTC
AS$AREB6_49	46 (-)	0	100.00	AREB6	GTTTC
AS$AREB6_50	46 (-)	0	100.00	AREB6	GTTTC
AS$AREB6_52	46 (-)	0	100.00	AREB6	GTTTC
AS$AREB6_53	46 (-)	0	100.00	AREB6	GTTTC
AS$CF1_37	54 (-)	1	87.50	CF1	GGGGTTGTA
AS$STAT_01	59 (+)	1	87.50	STAT6	TTCCGGGAA
AS$STAT_02	59 (+)	1	87.50	STAT6	TTCCGGGAA
AS$STAT5A_38	59 (+)	1	87.50	STAT5A	TTCCGGGAA
AS$STAT5A_44	59 (+)	1	87.50	STAT5A	TTCCGGGAA
AS$STAT_10	59 (-)	1	87.50	STAT6	TTCCCGGAA
AS$STAT5A_39	59 (-)	1	87.50	STAT5A	TTCCCGGAA
AS$STAT5A_60	59 (-)	1	88.89	STAT5A	TTTCTCGGGA
HS$P21WAF1_09	59 (-)	1	87.50	STAT1, STAT3	TTCCCGGAA
HS$IFI616_01	64 (+)	0	100.00	ISGF-3	GGAAA
MOUSE$IL5_02	64 (+)	0	100.00	NF-ATc, NF-ATp, NF-ATx	GGAAA
HS$IL4_01	64 (+)	0	100.00		GGAAA
MOUSE$IL4_06	64 (+)	0	100.00		GGAAA
MOUSE$IL4_07	64 (+)	0	100.00		GGAAA
MOUSE$IL4_08	64 (+)	0	100.00		GGAAA
HS$IL5_02	64 (+)	0	100.00		GGAAA
HS$IL13_01	64 (+)	0	100.00		GGAAA
MOUSE$CD40L_01	64 (+)	0	100.00		GGAAA
MOUSE$CD40L_02	64 (+)	0	100.00		GGAAA
MOUSE$CD40L_03	64 (+)	0	100.00		GGAAA
MOUSE$CD40L_04	64 (+)	0	100.00		GGAAA
HS$CD40L_01	64 (+)	0	100.00		GGAAA
HS$CD40L_02	64 (+)	0	100.00		GGAAA
HS$IFNG_03	64 (+)	0	100.00		GGAAA
HS$IFNG_04	64 (+)	0	100.00		GGAAA
HS$TNFA_05	64 (+)	0	100.00		GGAAA
MOUSE$MCP3_01	64 (+)	0	100.00		GGAAA
HS$TNFSF6_01	64 (+)	0	100.00		GGAAA
HCMV$IE1_16	64 (-)	0	100.00	NF-1	CTTTCC
OAT$PHYA3_03	65 (+)	0	100.00		GAAAGAA
CHICK$LYS_01	68 (+)	0	100.00	GR, GR-alpha, GR-beta, PR, PR A	AGAACA
FELV$FELV_04	68 (+)	0	100.00	GR	AGAACAG
AS$GR_02	68 (+)	0	100.00	GR-alpha, GR-beta	AGAACA
AS$GR_03	68 (+)	0	100.00	GR-alpha, GR-beta	AGAACA
AS$GR_04	68 (+)	0	100.00	GR, GR-alpha, GR-beta	AGAACA
HIV1$HIV1_22	68 (+)	0	100.00	GR	AGAACA
AS$GR_05	68 (+)	0	100.00	GR	AGAACA
AS$GR_11	68 (+)	0	100.00	GR-alpha, GR-beta	AGAACA
RAT$GSTYA2_01	68 (+)	0	100.00	GR	AGAACA
I$HSF_01	68 (+)	0	90.00	HSF	AGAAN
F$HSF_01	68 (+)	0	90.00	HSF	AGAAN
MMTV$MMTV_03	68 (-)	0	100.00	AR, GR	TGTTCT
MMTV$MMTV_05	68 (-)	0	100.00	GR	TGTTCT
MMTV$MMTV_06	68 (-)	0	100.00	GR, PR B	TGTTCT
MMTV$MMTV_08	68 (-)	0	100.00	GR, PR B	TGTTCT
MMTV$MMTV_09	68 (-)	0	100.00	GR, PR B	TGTTCT
MMTV$MMTV_10	68 (-)	0	100.00	GR, PR B	TGTTCT
MMTV$MMTV_12	68 (-)	0	100.00	PR-alpha, PR-beta	TGTTCT
MMTV$MMTV_14	68 (-)	0	100.00	PR B	TGTTCT
MMTV$MMTV_19	68 (-)	0	100.00	GR, PR B	TGTTCT
MMTV$MMTV_22	68 (-)	0	100.00	PR B, PR-alpha, PR-beta	TGTTCT
MMTV$MMTV_23	68 (-)	0	100.00	GR	TGTTCT
MMTV$MMTV_24	68 (-)	0	100.00	PR-alpha, PR-beta	TGTTCT
MOUSE$RAS1_01	68 (-)	0	100.00	GR	TGTTCT
RAT$TAT_02	68 (-)	0	100.00	GR, GR-alpha, GR-beta, PR-alpha, PR-beta	TGTTCT
RABBIT$UG_02	68 (-)	0	100.00	GR	TGTTCT
RABBIT$UG_04	68 (-)	0	100.00	GR	TGTTCT
RABBIT$UG_05	68 (-)	0	100.00	GR	TGTTCT
CHICK$PR_03	68 (-)	0	100.00	PR-alpha, PR-beta	TGTTCT
AS$GR_02	68 (-)	0	100.00	GR-alpha, GR-beta	TGTTCT
AS$GR_03	68 (-)	0	100.00	GR-alpha, GR-beta	TGTTCT
AS$GR_04	68 (-)	0	100.00	GR, GR-alpha, GR-beta	TGTTCT
AS$GR_05	68 (-)	0	100.00	GR	TGTTCT
AS$GR_07	68 (-)	0	100.00	GR	TGTTCT
AS$GR_08	68 (-)	0	100.00	GR	TGTTCT
AS$GR_09	68 (-)	0	100.00	GR	TGTTCT
AS$GR_10	68 (-)	0	100.00	GR	TGTTCT
AS$GR_11	68 (-)	0	100.00	GR-alpha, GR-beta	TGTTCT
RAT$ANF_02	68 (-)	0	100.00	GR	TGTTCT
MOUSE$CRISP1_01	68 (-)	0	100.00	AR	TGTTCT
MOUSE$CRISP1_02	68 (-)	0	100.00	AR	TGTTCT
MOUSE$CRISP3_01	68 (-)	0	100.00	AR	TGTTCT
RAT$SGK_02	68 (-)	0	100.00	GR	TGTTCT
MOMLV$MOMULV_01	69 (+)	0	100.00	LVa	GAACAG
MOMLV$MOMULV_02	69 (+)	0	100.00	LVa	GAACAG
HS$CMYC_13	69 (+)	1	87.50		GAAAAGAAA
AS$HB_09	69 (+)	1	88.89	Hb	GAACAAAAAA
MOUSE$EPOR_03	71 (-)	1	87.50	GATA-1	CTTATCTGT
AS$MSX1_07	72 (-)	1	87.50	Msx-1	ACTTTTATG
HS$TNFA_04	73 (+)	0	100.00		AGAAA
I$HSF_01	73 (+)	0	90.00	HSF	AGAAN
F$HSF_01	73 (+)	0	90.00	HSF	AGAAN
Y$GAL4_01	74 (-)	0	100.00	MIG1	TTTTC
Y$PHR1_01	76 (+)	0	100.00	PRP, RPH1	AAAGTA
Y$PHR1_01	76 (-)	0	100.00	PRP, RPH1	TACTTT
MOUSE$CRISP1_02	78 (+)	0	100.00	AR	AGTAGA
CAMV$35SR_06	79 (+)	0	100.00	MNB1b	GTAGAA
MAIZE$PEPC_01	79 (+)	0	100.00	MNB1b, MNF1	GTAGAA
MOUSE$CMYC_01	81 (+)	0	100.00	myc-PRF	AGAAAGGG
HS$TNFA_04	81 (+)	0	100.00		AGAAA
I$HSF_01	81 (+)	0	90.00	HSF	AGAAN
F$HSF_01	81 (+)	0	90.00	HSF	AGAAN
EBV$IR4_04	86 (+)	0	100.00	R	GGGAC
EBV$IR4_05	86 (+)	0	100.00	R	GGGAC
HS$CYP24_01	91 (-)	0	100.00	VDR	GAACTC
AS$ZIC3_11	91 (-)	1	87.50	Zic3	TGGGGACTC
AS$TR_07	93 (+)	0	100.00	T3R-alpha	GTTCC
AS$TR_10	93 (+)	0	100.00	T3R-alpha	GTTCC
HS$IL4_02	93 (-)	0	100.00		GGAAC
RAT$CYTOPB_02	94 (+)	0	100.00	RBP-Jkappa	TTCCCAC
AS$IK_06	94 (-)	0	100.00	Ik-1, Ik-2	TGGGAA
DROME$ESPL_01	94 (-)	1	87.50	RBP-Jkappa	CTGTGGGAA
HS$CDH1_01	95 (+)	0	100.00	LUN-1	TCCCA
HS$TLN_01	95 (+)	0	100.00	LUN-1	TCCCA
HS$CMYC_12	95 (-)	1	88.89		GCGTGGGGGA
HS$CDH1_01	95 (-)	0	100.00	LUN-1	TGGGA
HS$TLN_01	95 (-)	0	100.00	LUN-1	TGGGA
MAIZE$ADH1P_04	96 (+)	1	87.50		CCCACAGGC
HS$IL3_08	96 (-)	0	100.00	AML1, AML1a, AML1c	TGTGGG
AS$NCX_30	97 (-)	1	88.89	Ncx	CTGCGAGTGG
RAT$CYTOP_04	100 (+)	0	100.00	AhR, Arnt	CACGC
AS$AHRARNT_50	100 (-)	0	100.00	AhR, Arnt	GCGTG
DROME$ADH_14	103 (+)	0	100.00	Adf-1	GCAGC
DROME$ADH_06	103 (-)	0	100.00	Adf-1	GCTGC
HS$CMYC_16	103 (-)	0	100.00	CTCF	GCTGC
HS$AG_01	105 (+)	0	100.00	CTF, CTF-1, CTF-2, CTF-3, EFI	AGCCAAT
HS$BG_02	105 (+)	0	100.00	CTF	AGCCAAT
ASPN$AMDS_01	105 (+)	0	100.00	AmdR, AnCF	AGCCAAT
ASPN$AMDS_02	105 (+)	0	100.00	AnCF	AGCCAAT
HSV1$TK_02	105 (-)	0	100.00	CP1, CTF, NF-1	ATTGGCT
HS$DPOLA_03	105 (-)	0	100.00	CP1, CTF	ATTGGCT
MOUSE$AAG_05	106 (+)	0	100.00	alpha-CP1, CCAAT-binding factor	GCCAAT
HS$CJUN_02	106 (+)	0	100.00	CTF	GCCAATG
AD2$ORI_01	106 (+)	0	100.00	CTF-1, DBP, NF-1	GCCAAT
MOUSE$JUND_04	106 (+)	0	100.00		GCCAAT
MOUSE$AP2_07	106 (-)	0	100.00	NF-1	TTGGC
HS$BAC_03	107 (+)	0	100.00	SRF	CCAAT
AMULV$AMULV_01	107 (+)	0	100.00		CCAAT
EBV$DSL_06	107 (+)	0	100.00		CCAAT
MOUSE$AAG_04	107 (+)	0	100.00	alpha-CP1, alpha-CP2a, alpha-CP2b, alpha-IRP	CCAAT
CHICK$BAG_06	107 (+)	0	100.00	TGGCA-binding protein	CCAAT
HS$GG_15	107 (+)	0	100.00	NF-E	CCAAT
HS$GG_16	107 (+)	0	100.00	CDP2, Clox, CUTL1, Cutl1	CCAAT
HS$GG_17	107 (+)	0	100.00	gammaCAAT	CCAAT
HS$GG_18	107 (+)	0	100.00	CP1	CCAAT
HS$ZG_07	107 (+)	0	100.00	CP2	CCAAT
HS$ZG_08	107 (+)	0	100.00	CP1	CCAAT
HS$HH1_03	107 (+)	0	100.00	H1TF2	CCAAT
MOUSE$M2IAB_01	107 (+)	0	100.00	PU.1	CCAAT
PIG$UPA_12	107 (+)	0	100.00	NF-1	CCAAT
RADLV$RLV_10	107 (+)	0	100.00		CCAAT
MULV$MULV_03	107 (+)	0	100.00		CCAAT
MOUSE$GLUT4_04	107 (+)	0	100.00		CCAAT
MOMLV$MOMULV_15	107 (+)	0	100.00		CCAAT
RAT$ALDB_02	107 (+)	0	100.00	CBF (2), CP2	CCAAT
HS$GG_20	107 (+)	0	100.00	CP1, NF-E3	CCAAT
RAT$TH2B_01	107 (+)	0	100.00		CCAAT
RAT$TH2B_02	107 (+)	0	100.00		CCAAT
HS$GG_21	107 (+)	0	100.00	CP1	CCAAT
MOUSE$LY6E_01	107 (+)	0	100.00		CCAAT
RAT$NEU_01	107 (+)	0	100.00		CCAAT
HS$GHA_10	107 (+)	0	100.00	alpha-CBF	CCAAT
NEUCR$CPC1_02	107 (+)	0	100.00		CCAAT
HS$EG_07	107 (+)	0	100.00	CP1	CCAAT
HS$GP2B_13	107 (+)	0	100.00		CCAAT
MOUSE$BMG_10	107 (+)	0	100.00		CCAAT
HS$CYBH_01	107 (+)	0	100.00	CDP2, Clox, CP1, CUTL1, Cutl1	CCAAT
HBV$S_04	107 (+)	0	100.00		CCAAT
HS$FN_06	107 (+)	0	100.00		CCAAT
MOUSE$E2F1_02	107 (+)	0	100.00		CCAAT
HS$A24COL_01	107 (+)	0	100.00		CCAAT
MOUSE$EKLF_01	107 (+)	0	100.00	AP-1, GATA-1	CCAAT
MOUSE$A11COL_01	107 (-)	0	100.00	CBF (2), IF2	ATTGG
MOUSE$A21COL_03	107 (-)	0	100.00	CCAAT-binding factor	ATTGG
MOUSE$A21COL_04	107 (-)	0	100.00	CBF (2), CBF-A, CBF-B	ATTGG
MOUSE$A21COL_05	107 (-)	0	100.00	EFI	ATTGG
AD2$E2L_03	107 (-)	0	100.00	CRF	ATTGG
HS$GHA_06	107 (-)	0	100.00	NF-1	ATTGG
SP$H2B1_02	107 (-)	0	100.00	CBF (1)	ATTGG
SP$H2B1_03	107 (-)	0	100.00	CDF	ATTGG
HS$HSP70_02	107 (-)	0	100.00	CTF	ATTGG
HS$HSP70_07	107 (-)	0	100.00	CBTF, CP1, CTF	ATTGG
MOUSE$M2EAK_07	107 (-)	0	100.00	NF-Y	ATTGG
MOUSE$M2EAK_08	107 (-)	0	100.00	NF-Y	ATTGG
RSV$RSV_05	107 (-)	0	100.00		ATTGG
HSV1$TK_04	107 (-)	0	100.00	C/EBPalpha	ATTGG
HSV1$TK_05	107 (-)	0	100.00	NF-Y'	ATTGG
AS$HAP23_01	107 (-)	0	100.00	HAP2, HAP3, HAP4	ATTGG
MOUSE$M1H2KB_12	107 (-)	0	100.00	EFI	ATTGG
RAT$TH2A_02	107 (-)	0	100.00		ATTGG
RAT$TH2A_04	107 (-)	0	100.00		ATTGG
HS$TK_02	107 (-)	0	100.00	CP1	ATTGG
MOUSE$A11COL_07	107 (-)	0	100.00	NF-1, Sp1	ATTGG
HS$CDC2_07	107 (-)	0	100.00	CBAF, CBF-B, CP1A	ATTGG
XENLA$GATA2_01	107 (-)	0	100.00	CBTF	ATTGG
RAT$NAT_05	107 (-)	0	100.00		ATTGG
HS$CDC2_12	107 (-)	0	100.00	CBAF	ATTGG
HS$CRH_01	111 (-)	0	100.00	GR	TGGACA
CHICK$OA_05	114 (+)	0	100.00		CCATG
CHICK$OA_05	115 (-)	0	100.00		CCATG
HS$GRH_01	117 (+)	0	100.00	AP-2, AP-2alphaA, AP-2alphaB, NF-1	TGGCC
HSV1$TK_12	117 (+)	0	100.00	RAR-alpha1, T3R-alpha	TGGCCT
HS$GRH_01	117 (-)	0	100.00	AP-2, AP-2alphaA, AP-2alphaB, NF-1	GGCCA
RAT$TAT_15	117 (-)	0	100.00	HNF-3alpha	GGCCA
HS$CYP7A_02	117 (-)	1	87.50	FTF	TCAAGGCCA
MOUSE$EPOR_03	121 (+)	1	87.50	GATA-1	CTTATCTGT
AS$AREB6_06	121 (+)	1	87.50	AREB6	CTTACCTGT
AS$CMYB_02	123 (+)	0	100.00	c-Myb	TAACTG
MOUSE$RVL3_03	123 (+)	0	100.00	RXR-alpha	TAACT
NT$PR1A_02	123 (+)	0	100.00	MYB1	TAACTG
MAIZE$BZ1_02	123 (+)	0	100.00	C1 (long form), MYB2	TAACTG
AS$CMYB_01	123 (-)	0	100.00	c-Myb	CAGTTA
AS$CMYB_02	123 (-)	0	100.00	c-Myb	CAGTTA
HS$PR264_03	123 (-)	0	100.00	c-Myb	CAGTTA
AS$ZIC2_25	125 (+)	1	87.50	Zic2	ACGGTGCTT
RAT$RPK_02	127 (+)	0	100.00	Sp1	TGTGC
RAT$A2UG_08	127 (-)	0	100.00	GR	AGCACA
RAT$A1I3_04	128 (+)	0	100.00		GTGCT
RAT$A1I3_04	128 (-)	0	100.00		AGCAC
RAT$GRH_18	131 (+)	0	100.00	T3R-alpha	CTTGGG
HS$NPY_03	131 (-)	0	100.00	AP-1, CCAAT-binding factor	CCAAG
HS$CD8A_02	131 (-)	1	87.50	LyF-1	CCTCCCAAG
AS$LYF1_03	132 (+)	0	100.00	LyF-1	TTGGGA
AS$IK_06	133 (+)	0	100.00	Ik-1, Ik-2	TGGGAA
HS$CDH1_01	133 (+)	0	100.00	LUN-1	TGGGA
HS$TLN_01	133 (+)	0	100.00	LUN-1	TGGGA
HS$CDH1_01	133 (-)	0	100.00	LUN-1	TCCCA
HS$TLN_01	133 (-)	0	100.00	LUN-1	TCCCA
HS$CDC2_06	134 (+)	0	100.00	c-Ets-2	GGGAAG
MOUSE$RPL32_01	135 (+)	0	100.00	f(alpha)-f(epsilon), HrpF, XrpFI	GGAAG
HS$TNFA_02	135 (+)	0	100.00		GGAAG
HS$TNFA_03	135 (+)	0	100.00		GGAAG
XENLA$RPL14_01	135 (-)	0	100.00	HrpF, XrpFI	CTTCC
HS$DG_03	135 (-)	1	88.89		CCTTCCTTCC
Y$TPI_01	135 (-)	0	100.00	GCR1	CTTCC
MOUSE$M1H2KB_11	136 (+)	0	100.00		GAAGGAA
HS$CDC2_01	137 (+)	0	100.00	c-Ets-2	AAGGAA
HS$CDC2_02	137 (-)	0	100.00	c-Ets-2	TTCCTT
PEA3$CONS	138 (+)	0	91.67	c-Ets-1 54, c-Ets-1 68, c-Ets-2 58-64, PEA3	AGGAAR
TIN1$CONS	138 (+)	1	87.50	TIN-1	AGGAAGTTC
DROME$E74_10	138 (+)	0	100.00	E74A	AGGAA
DROME$E74_11	138 (+)	0	100.00	E74A	AGGAA
E74A$CONS_01	138 (+)	0	90.00	E74A	MGGAA
MOUSE$PGK1_01	138 (+)	1	88.89	TIN-1	AGGAAGTTCC
AS$ZIC2_19	138 (+)	1	87.50	Zic2	AGGAAGGTC
MOUSE$RPL32_01	139 (+)	0	100.00	f(alpha)-f(epsilon), HrpF, XrpFI	GGAAG
HS$TNFA_02	139 (+)	0	100.00		GGAAG
HS$TNFA_03	139 (+)	0	100.00		GGAAG
XENLA$RPL14_01	139 (-)	0	100.00	HrpF, XrpFI	CTTCC
Y$TPI_01	139 (-)	0	100.00	GCR1	CTTCC
XENLA$ACY_01	141 (+)	0	100.00	SRF	AAGAT
XENLA$ACY_01	141 (-)	0	100.00	SRF	ATCTT
SV$SV40_37	147 (+)	0	100.00		CTGGG
SV$SV40_63	148 (+)	0	100.00	T-Ag	TGGGC
HS$GRH_01	150 (+)	0	100.00	AP-2, AP-2alphaA, AP-2alphaB, NF-1	GGCCA
RAT$TAT_15	150 (+)	0	100.00	HNF-3alpha	GGCCA
HS$GRH_01	150 (-)	0	100.00	AP-2, AP-2alphaA, AP-2alphaB, NF-1	TGGCC
Y$GSY2_01	152 (+)	0	100.00		CCAGGGG
MOUSE$THY1_05	153 (-)	1	87.50		CCACCCCTG
AS$VDR_04	154 (+)	0	100.00	VDR	AGGGG
Y$GLK1_02	154 (+)	0	100.00	MSN2, MSN4	AGGGG
HS$AG_15	155 (+)	0	100.00	CAC-binding protein	GGGGTG
CHICK$PTH_01	156 (+)	0	100.00	RXR-alpha, VDR	GGGTGT
HS$BG_01	156 (-)	0	100.00	CACCC-binding factor	CACCC
HS$GG_13	156 (-)	0	100.00	gammaCAC1, gammaCAC2	CACCC
HS$GG_14	156 (-)	0	100.00	CACCC-binding factor, Sp1	CACCC
HS$GP2B_01	156 (-)	0	100.00	CACCC-binding factor	CACCC
RAT$TOA_02	156 (-)	0	100.00	CACCC-binding factor	CACCC
HS$EG_06	156 (-)	0	100.00	Sp1	CACCC
AS$CACCC_01	156 (-)	0	100.00	CACCC-binding factor	CACCC
HS$CYP24_01	156 (-)	0	100.00	VDR	ACACCC
RAT$GSTYA2_01	159 (+)	0	100.00	GR	TGTACC
RAT$CYTOPB_01	159 (-)	0	100.00	GR	GGTACA
MOUSE$CRISP1_03	159 (-)	0	100.00	AR	GGTACA
CTCF$CONS	163 (+)	0	100.00	CTCF	CCCTC
Y$SUC2_01	169 (+)	0	100.00	MIG1	TTTTT
AS$PF1_01	169 (+)	0	100.00	PF1	TTTTT
AS$PF1_02	169 (+)	0	100.00	PF1	TTTTT
NT$PR1A_03	169 (-)	0	100.00	GT-1	GAAAAA
Y$GAL4_01	170 (+)	0	100.00	MIG1	TTTTC
MOUSE$MBP_04	172 (+)	0	100.00	TFIID	TTCAAA
HS$CDC2_10	172 (-)	0	100.00		TTGAA
HS$CYCA_06	172 (-)	0	100.00		TTGAA
HS$CDC25C_05	172 (-)	0	100.00		TTGAA
DROME$UBX_24	173 (-)	0	100.00	LEF-1	TTTGA
MOUSE$CRISP3_01	175 (+)	0	100.00	AR	AAAACT
HS$FIX_04	177 (+)	0	100.00	AR, HNF-4alpha	AACTAA
AS$FREAC2_01	180 (+)	0	100.00	FOXF2	TAAAC
RICE$EM_01	183 (+)	0	100.00	OSBZ8, TRAB1	ACGTG
RICE$EM_02	183 (+)	0	100.00	OSBZ8	ACGTG
AS$mEMBP_15	183 (-)	0	100.00	EmBP-1a	CACGT
MAIZE$C1_05	185 (+)	0	100.00		GTGTC
AS$TGIF_09	186 (+)	0	100.00	TGIF	TGTCT
HS$PTH_04	190 (+)	0	100.00	Ref-1	TGAGAC
HS$PTH_04	190 (-)	0	100.00	Ref-1	GTCTCA
RAT$ANP_01	190 (-)	0	100.00		GTCTCA
AS$ARF1_01	191 (+)	0	100.00	ARF1, ARF5	GAGACA
PEA$IAA45_07	191 (+)	0	100.00	ARF1	GAGACA
YL$XPR2_01	191 (-)	0	100.00		GTCTC
AS$ARF1_01	191 (-)	0	100.00	ARF1, ARF5	TGTCTC
AS$ARF1_05	191 (-)	0	100.00	ARF1, ARF4, ARF5, ARF6, ARF7, ARF8	TGTCTC
AS$TGIF_09	192 (-)	0	100.00	TGIF	TGTCT
AS$MEIS1_10	192 (-)	0	100.00	Meis-1a, Meis-1b	CTGTCT
HS$GG_12	193 (-)	0	100.00	NF-E	CTGTC
DROME$EVE_10	193 (-)	0	100.00	GAGA factor	CTGTC
DROME$YP1_02	198 (+)	1	87.50	DSXF, DSXM	CTACAATGT
DROME$YP1_03	198 (+)	0	100.00	DSXF, DSXM	CTACAAAGT
XENLA$ENGRAILED2_01	199 (-)	0	100.00	LEF-1	CTTTGTA
MOUSE$ECADH_04	199 (-)	0	100.00	LEF-1	CTTTGTA
HS$MMP3_03	201 (+)	1	91.67	FOXF1	CAATGTTTATTAA
AS$FREAC2_01	205 (-)	0	100.00	FOXF2	TAAAC
HS$CD40_01	205 (-)	1	87.50	AKNA	TTAATAAAT
Y$PDC1_02	206 (+)	0	100.00	MIG1	TTTAT
Y$HAP4_01	206 (+)	0	100.00	MIG1	TTTAT
AS$MEIS1BHOXA9_07	206 (+)	0	100.00	HOXA9, Meis-1b	TTTATTA
HS$EG_08	206 (-)	0	100.00		ATAAA
HS$GFAP_01	206 (-)	0	100.00	TFIID	ATAAA
HS$GFAP_02	206 (-)	0	100.00	TBP	ATAAA
BOVIN$IRBP_03	207 (-)	0	100.00	Crx	TTAATAA
Y$MEL1_02	209 (+)	0	100.00	MIG1	ATTAA
CHICK$MGF_01	209 (+)	0	100.00	Gbx2	ATTAA
CHICK$MGF_02	209 (-)	0	100.00	Gbx2	TTAAT
EBV$IR4_04	214 (+)	0	100.00	R	GGGAC
EBV$IR4_05	214 (+)	0	100.00	R	GGGAC
RAT$TAT_04	215 (-)	0	100.00	GR	AAGTCC
DROME$EVE_08	221 (+)	0	100.00	GAGA factor	GAGAG
DROME$EVE_13	221 (+)	0	100.00	GAGA factor	GAGAG
DROME$FTZ_32	221 (+)	0	100.00	GAGA factor	GAGAG
AS$GAGA_03	221 (+)	0	100.00	GAGA factor	GAGAG
DROME$EVE_09	221 (-)	0	100.00	GAGA factor	CTCTC
DROME$FTZ_32	221 (-)	0	100.00	GAGA factor	CTCTC
YL$XPR2_01	223 (-)	0	100.00		GTCTC
MOUSE$CRISP3_01	231 (-)	0	100.00	AR	AAAACT
AS$HFH2_14	232 (+)	1	90.91	FOXD3	GTTTGTTGTTTT
VIV$VISNA_02	233 (+)	0	100.00		TTTTTTG
Y$SUC2_01	233 (+)	0	100.00	MIG1	TTTTT
AS$PF1_01	233 (+)	0	100.00	PF1	TTTTT
AS$PF1_02	233 (+)	0	100.00	PF1	TTTTT
FY$MFM1_02	233 (-)	1	87.50	mat1-Mc, ste11	AACAAAGAA
AS$HB_09	233 (-)	1	88.89	Hb	GAACAAAAAA
AS$HB_13	233 (-)	1	88.89	Hb	AAATAAAAAA
Y$SUC2_01	234 (+)	0	100.00	MIG1	TTTTT
RAT$KINK_02	234 (+)	1	88.89	HNF-3alpha, HNF-3B, HNF-3gamma	TGTTTGTTTT
AS$PF1_01	234 (+)	0	100.00	PF1	TTTTT
AS$PF1_02	234 (+)	0	100.00	PF1	TTTTT
AS$FOXJ2_27	234 (-)	1	88.89	FOXJ2 (long isoform)	AAAACAAATA
MOUSE$HNF3B_02	235 (+)	1	87.50	HNF-3alpha, HNF-3B	GTTTGTTTT
HS$CYCD1_09	235 (+)	0	100.00	TCF-4E	TTTTGTT
HS$PTHRO_03	235 (+)	1	90.91	FOXJ1	TTTTGTTTGTTT
HS$APP_03	235 (+)	1	90.91	FOXJ1	TTTTGTTTGTTT
AS$FOXJ1_05	236 (+)	0	100.00	FOXJ1	TTTGTTT
RAT$IGFBP1_01	236 (-)	0	100.00	HNF-3alpha, HNF-3B, HNF-3gamma	AAACAAA
AS$FOXO3_01	236 (-)	0	100.00	FOXO3a	AAAACAAA
DROME$EVE_29	237 (+)	1	88.89	Hb	TTATTTTTTT
PEA$IAA45_06	237 (-)	0	100.00		AAAACAA
MOUSE$CDX2_01	238 (+)	0	100.00	FOXM1a, FOXM1b, HNF-3alpha, HNF-3B	TGTTT
RAT$FABPI_04	238 (+)	0	100.00	FOXM1a, FOXM1b, HNF-3alpha, HNF-3B	TGTTT
HS$ALP_02	238 (+)	0	100.00	HNF-3alpha, HNF-3B	TGTTT
RAT$KINK_02	238 (+)	1	88.89	HNF-3alpha, HNF-3B, HNF-3gamma	TGTTTGTTTT
MOUSE$ECADH_03	238 (-)	0	100.00	FOXM1a, FOXM1b, HNF-3alpha, HNF-3B	AAACA
AS$FOXJ2_25	238 (-)	1	88.89	FOXJ2 (long isoform)	AAAATAAACA
AS$FOXJ2_26	238 (-)	1	88.89	FOXJ2 (long isoform)	AAAATAAACA
AS$FOXJ2_38	238 (-)	1	88.89	FOXJ2 (long isoform)	AAAATAAACA
HS$ADH2_11	238 (-)	0	100.00	GR	AAAACA
MOUSE$HNF3B_02	239 (+)	1	87.50	HNF-3alpha, HNF-3B	GTTTGTTTT
Y$SUC2_01	240 (+)	0	100.00	MIG1	TTTTT
DROME$EVE_29	240 (+)	1	88.89	Hb	TTATTTTTTT
AS$PF1_01	240 (+)	0	100.00	PF1	TTTTT
AS$PF1_02	240 (+)	0	100.00	PF1	TTTTT
AS$HB_13	240 (-)	1	88.89	Hb	AAATAAAAAA
Y$SUC2_01	241 (+)	0	100.00	MIG1	TTTTT
DROME$EVE_29	241 (+)	1	88.89	Hb	TTATTTTTTT
AS$PF1_01	241 (+)	0	100.00	PF1	TTTTT
AS$PF1_02	241 (+)	0	100.00	PF1	TTTTT
AS$HB_13	241 (-)	1	88.89	Hb	AAATAAAAAA
Y$SUC2_01	242 (+)	0	100.00	MIG1	TTTTT
DROME$EVE_29	242 (+)	1	88.89	Hb	TTATTTTTTT
AS$PF1_01	242 (+)	0	100.00	PF1	TTTTT
AS$PF1_02	242 (+)	0	100.00	PF1	TTTTT
AS$HB_13	242 (-)	1	88.89	Hb	AAATAAAAAA
Y$SUC2_01	243 (+)	0	100.00	MIG1	TTTTT
AS$PF1_01	243 (+)	0	100.00	PF1	TTTTT
AS$PF1_02	243 (+)	0	100.00	PF1	TTTTT
Y$SUC2_01	244 (+)	0	100.00	MIG1	TTTTT
RAT$MLCC_01	244 (+)	1	88.89	aMEF-2, MEF-2 (516 AA)	TTATTTTTAA
AS$PF1_01	244 (+)	0	100.00	PF1	TTTTT
AS$PF1_02	244 (+)	0	100.00	PF1	TTTTT
RAT$AMHC_03	244 (-)	1	88.89	aMEF-2, MEF-2 (516 AA)	TTAAAAATAA
DROME$TM1_01	244 (-)	1	88.89	D-MEF2	TTAAAAATAA
Y$SUC2_01	245 (+)	0	100.00	MIG1	TTTTT
AS$PF1_01	245 (+)	0	100.00	PF1	TTTTT
AS$PF1_02	245 (+)	0	100.00	PF1	TTTTT
Y$SUC2_01	246 (+)	0	100.00	MIG1	TTTTT
DROME$UBX_18	246 (+)	1	88.89	Hb	TTTTTTAATG
AS$PF1_01	246 (+)	0	100.00	PF1	TTTTT
AS$PF1_02	246 (+)	0	100.00	PF1	TTTTT
MOUSE$ADA_06	246 (-)	0	100.00	TBP	TAAAAAA
Y$SUC2_01	247 (+)	0	100.00	MIG1	TTTTT
AS$PF1_01	247 (+)	0	100.00	PF1	TTTTT
AS$PF1_02	247 (+)	0	100.00	PF1	TTTTT
Y$MAL2R_01	248 (+)	0	100.00	MIG1	TTTTA
AS$FOXJ1_04	248 (+)	0	100.00	FOXJ1	TTTTA
Y$MAL63_01	249 (+)	0	100.00	MIG1	TTTAA
CHICK$MGF_02	250 (+)	0	100.00	Gbx2	TTAAT
Y$MEL1_02	250 (-)	0	100.00	MIG1	ATTAA
CHICK$MGF_01	250 (-)	0	100.00	Gbx2	ATTAA
SP$SPEC2A_02	251 (+)	0	100.00	SpOtx	TAATCT
HS$ACTH_03	251 (+)	1	87.50	SF-1	TAACCTTGA
SP$SPEC2A_02	251 (-)	0	100.00	SpOtx	AGATTA
MOUSE$CTNC_01	251 (-)	0	100.00	GATA-4	AGATTA
BOVIN$LHB_02	251 (-)	0	100.00	Pitx1	AGATTA
MOUSE$BMG_04	252 (+)	0	100.00	GATA-1	AATCT
XENLA$ACY_01	253 (+)	0	100.00	SRF	ATCTT
XENLA$ACY_01	253 (-)	0	100.00	SRF	AAGAT
DROME$KNI_04	256 (-)	1	87.50	Tll	GGAAGTCAA
HS$CYP24_01	258 (-)	0	100.00	VDR	GAACTC
PU1$CONS	259 (-)	1	87.50	PU.1, Pu.1	AGAGGAACT
AS$PU1_01	259 (-)	1	87.50	PU.1	AGAGGAACT
AS$TR_07	260 (+)	0	100.00	T3R-alpha	GTTCC
AS$TR_10	260 (+)	0	100.00	T3R-alpha	GTTCC
HS$IL4_02	260 (-)	0	100.00		GGAAC
HS$CDC2_02	261 (+)	0	100.00	c-Ets-2	TTCCTT
DROME$E74_10	261 (-)	0	100.00	E74A	AGGAA
DROME$E74_11	261 (-)	0	100.00	E74A	AGGAA
E74A$CONS_01	261 (-)	0	90.00	E74A	MGGAA
HS$CDC2_01	261 (-)	0	100.00	c-Ets-2	AAGGAA
AS$SRY_02	264 (-)	0	100.00	SRY	AAGAAAG
AS$HB_13	265 (-)	1	88.89	Hb	AAATAAAAAA
HS$TNFA_04	265 (-)	0	100.00		AGAAA
I$HSF_01	265 (-)	0	90.00	HSF	AGAAN
F$HSF_01	265 (-)	0	90.00	HSF	AGAAN
HS$ADH2_11	267 (+)	0	100.00	GR	TCTTAT
MOUSE$HOXA5_04	269 (-)	0	100.00		AAATAA
MOUSE$HOXA5_05	269 (-)	0	100.00		AAATAA
MOUSE$HOXA5_06	269 (-)	0	100.00		AAATAA
HS$MSH2_01	270 (+)	0	100.00	HNF-3alpha, HNF-3B	TATTT
MOUSE$IL2_12	270 (-)	0	100.00	NF-AT3, NF-ATc, NF-ATp, NF-ATx	TGAAAATA
MOUSE$IGH_35	271 (+)	1	87.50	POU2F1	ATTTGCATT
MOUSE$IGH_36	271 (+)	1	87.50	POU2F1a, POU2F1b, POU2F1c, POU2F2, POU2F2 (Oct-2.1), POU2F2 (Oct-2.3), POU2F2 (Oct-2.4), POU2F2 (Oct-2.6), POU2F2B, POU3F1, POU5F1 (Oct-5)	ATTTGCATT
HS$IGH_05	271 (+)	0	100.00		ATTTTCAT
Y$MAL61_03	271 (+)	0	100.00	MIG1	ATTTT
Y$GAL4_01	272 (+)	0	100.00	MIG1	TTTTC
HS$PL_10	272 (-)	0	100.00	POU1F1a	ATGAAAA
DROME$EN_05	273 (-)	1	87.50	En	TCAATTAAA
AS$STAT5A_65	274 (+)	1	87.50	STAT5A	TTCATAGAG
PSAM$U7SN_04	277 (+)	0	100.00		ATTGA
HS$EGFR_15	277 (-)	0	100.00		TCAAT
MOUSE$IGLL_02	279 (+)	1	87.50	LyF-1	TCAGGGAGA
HS$CMYC_18	280 (+)	1	87.50	MAZ, Sp1	GAGGGAGGG
HS$TCRA_01	280 (-)	1	88.89	c-Ets-1	GCTCTCCCGC
CTCF$CONS	280 (-)	0	100.00	CTCF	CCCTC
Y$HIS7_01	280 (-)	1	87.50	ABF1	CTCTCTCTC
HS$CS1_04	281 (+)	0	100.00	T3R	AGGGAG
HS$P21WAF1_05	281 (+)	0	100.00	RXR-alpha, VDR	AGGGAG
AS$VDR_08	281 (+)	0	100.00	VDR	AGGGAG
AS$LYF1_05	282 (+)	0	100.00	LyF-1	GGGAGA
CHICK$ITGB3_01	282 (+)	0	100.00	RXR-beta, VDR	GGGAGA
Y$ADH2_01	283 (-)	0	100.00	ADR1	TCTCC
DROME$EVE_08	284 (+)	0	100.00	GAGA factor	GAGAG
DROME$EVE_13	284 (+)	0	100.00	GAGA factor	GAGAG
DROME$FTZ_32	284 (+)	0	100.00	GAGA factor	GAGAG
AS$GAGA_03	284 (+)	0	100.00	GAGA factor	GAGAG
DROME$EVE_09	284 (-)	0	100.00	GAGA factor	CTCTC
DROME$FTZ_32	284 (-)	0	100.00	GAGA factor	CTCTC
AS$EN1_10	286 (+)	0	100.00	En-1	GAGCTTG
HS$CYP24_01	292 (-)	0	100.00	VDR	GAACTC
AS$RAR_01	293 (+)	0	100.00	RAR-gamma, RXR-alpha	AGTTCA
RAT$CRBP2_01	293 (+)	0	100.00	COUP-TF1, PPAR-alpha, RAR-alpha1, RAR-beta, RXR-alpha, RXR-beta, TGIF	AGTTCA
MOUSE$CRABP2_02	293 (+)	0	100.00	RXR-alpha, RXR-gamma	AGTTCA
AS$RAR_08	293 (+)	0	100.00	RAR-alpha1, RXR-alpha	AGTTCA
MOUSE$RARB_01	293 (+)	0	100.00	RAR-beta, RXR-alpha, RXR-beta	AGTTCA
MMTV$MMTV_49	293 (+)	0	100.00	LXR-alpha, RXR-alpha, SXR	AGTTCA
RAT$CYP7A_05	293 (+)	0	100.00	FXR, LXR-alpha, LXR-beta, RXR-alpha	AGTTCA
AS$LXRAB_01	293 (+)	0	100.00	LXR-alpha, LXR-beta, RXR-alpha	AGTTCA
RAT$CYP3A1_01	293 (+)	0	100.00	PXR-1, PXR-2, RXR-alpha	AGTTCA
RAT$CYP3A2_01	293 (+)	0	100.00	PXR-1, PXR-2, RXR-alpha	AGTTCA
AS$SXR_02	293 (+)	0	100.00	RXR-alpha, SXR	AGTTCA
AS$SXR_03	293 (+)	0	100.00	RXR-alpha, SXR	AGTTCA
AS$SXR_04	293 (+)	0	100.00	RXR-alpha, SXR	AGTTCA
MOUSE$PTHR_01	293 (+)	0	100.00	COUP-TF1, RAR-alpha1, RXR-alpha	AGTTCA
RAT$POMC_05	293 (-)	0	100.00	GR	TGAACT
MOUSE$RVL3_02	293 (-)	0	100.00	RAR-beta, RAR-gamma, RXR-alpha	TGAACT
HS$CYP3A4_01	293 (-)	0	100.00	PXR-1, RXR-alpha, RXR-beta, SXR	TGAACT
HS$CYP3A4_04	293 (-)	0	100.00	PXR-1, RXR-alpha	TGAACT
CHICK$CYP2H1_05	293 (-)	0	100.00	CXR, RXR-gamma	TGAACT
AS$RAR_05	294 (+)	0	100.00	RAR-alpha1	GTTCA
AS$RAR_06	294 (+)	0	100.00	RAR-alpha1	GTTCA
AS$TR_06	294 (+)	0	100.00	T3R-alpha	GTTCA
AS$TR_07	294 (+)	0	100.00	T3R-alpha	GTTCA
AS$TR_08	294 (+)	0	100.00	T3R-alpha	GTTCA
AS$TR_11	294 (+)	0	100.00	T3R-alpha	GTTCA
MOUSE$RARA2_01	294 (+)	0	100.00	RAR-alpha1, RAR-beta2, RAR-gamma1	GTTCA
HS$ADH3_01	294 (-)	0	100.00	CAR, RAR-alpha1, RAR-beta, RXR-alpha	TGAAC
MOUSE$M1H2KB_11	296 (+)	0	100.00		TCATGAT
HS$BG_06	298 (-)	0	100.00	GATA-1	CTTATCAT
NEUCR$NIT3_02	298 (-)	0	100.00	NIT2	TATCAT
MOUSE$IGH_55	298 (-)	0	100.00		ATCAT
MOUSE$AAG_01	299 (+)	0	100.00	GATA-1, GATA-2, GATA-3	TGATA
MOUSE$AAG_06	299 (+)	0	100.00	GATA-1	TGATA
HS$ZG_05	299 (+)	0	100.00	GATA-1	TGATAA
HS$GP2B_05	299 (+)	0	100.00	GATA-1	TGATAAG
CHICK$GATA1_01	299 (+)	0	100.00	GATA-1	TGATAA
MOUSE$TCRBL_02	299 (+)	0	100.00	GATA-3	TGATAA
RAT$BNP_01	299 (+)	0	100.00	GATA-4	TGATAA
MOUSE$BMG_05	299 (-)	0	100.00	factor B2, GATA-1	TATCA
HS$AG_06	299 (-)	0	100.00	GATA-1	TTATCA
MOUSE$PBGD_09	299 (-)	0	100.00	GATA-1	TTATCA
MOUSE$EKLF_02	299 (-)	0	100.00	GATA-1	TTATCA
MOUSE$GSHPX1_10	299 (-)	0	100.00	GATA-1	CTTATCA
HS$SELP_02	299 (-)	0	100.00	GATA-2	TTATCA
CAEEL$PHA4_01	299 (-)	0	100.00	elt-2	TTATCA
MOUSE$AAG_02	300 (+)	0	100.00		GATAAG
EBV$BHLF1_05	300 (+)	0	100.00	TBP	GATAA
Y$DAL3_01	300 (+)	0	100.00	DAL80	GATAA
RAT$AR_01	300 (+)	0	100.00	NF-kappaB	GATAAG
Y$ARS1_05	300 (-)	0	100.00	ABF2	TTATC
AS$MTTFA_01	300 (-)	0	100.00	mtTFA	TTATC
AS$MTTFA_02	300 (-)	0	100.00	mtTFA	TTATC
AS$MTTFA_03	300 (-)	0	100.00	mtTFA	TTATC
AS$MTTFA_04	300 (-)	0	100.00	mtTFA	TTATC
AS$MTTFA_05	300 (-)	0	100.00	mtTFA	TTATC
AS$MTTFA_06	300 (-)	0	100.00	mtTFA	TTATC
AS$MTTFA_07	300 (-)	0	100.00	mtTFA	TTATC
AS$MTTFA_08	300 (-)	0	100.00	mtTFA	TTATC
AS$MTTFA_09	300 (-)	0	100.00	mtTFA	TTATC
AS$SPZ1_02	301 (-)	1	87.50	Spz1	GGCGCTTAT
AS$NKX3A_05	302 (+)	0	100.00	NKX3A	TAAGTG
AS$NKX3A_19	302 (+)	0	100.00	NKX3A	TAAGTG
XENLA$U2SN_11	305 (+)	0	100.00		GTGCC
EBV$IR4_05	305 (-)	0	100.00	R	GGCAC
EBV$IR4_06	305 (-)	0	100.00	R	GGCAC
CHICK$BLCR_02	307 (-)	0	100.00	NF-E4, Sp1	GCGGC
HS$CYCD1_14	308 (+)	0	100.00	Sp1, Sp2, Sp3, Sp4	CCGCG
HS$CDC2_09	308 (-)	0	100.00		CGCGG
HS$CYCA_05	308 (-)	0	100.00		CGCGG
AS$ZIC2_08	312 (-)	1	87.50	Zic2	GGAGTAGTC
MOUSE$CRISP1_02	313 (-)	0	100.00	AR	AGTAGA
AS$S8_38	318 (-)	1	90.91	S8	AATTAGCCTGGA
RAT$ARGL_03	320 (-)	0	100.00	NF-1	AGCCAG
HS$GG_22	324 (+)	0	100.00		CTAAT
RAT$OPSIN_01	324 (+)	0	100.00		CTAAT
XENLA$BMP4_01	324 (+)	0	100.00	Xvent-2	CTAATT
AS$XVENT2_01	324 (+)	0	100.00	Xvent-2	CTAATT
Y$SUC2_02	325 (-)	0	100.00	MIG1	AATTA
RAT$DBH_01	325 (-)	0	100.00	ARIX, c-Fos, c-Jun, CREB, CREMtau	AATTA
DROME$YP1_03	326 (-)	1	87.50	DSXF, DSXM	TTACAAATT
MOUSE$GLUT4_01	327 (-)	1	87.50	C/EBPalpha	TTCAGAAAT
CHICK$LYS_19	327 (-)	1	87.50	AP-3, C/EBPalpha	TTTGGAAAT
RAT$KINK_01	327 (-)	0	100.00	C/EBPalpha, C/EBPbeta	TTTAGAAAT
HS$TNFA_04	328 (-)	0	100.00		AGAAA
I$HSF_01	328 (-)	0	90.00	HSF	AGAAN
F$HSF_01	328 (-)	0	90.00	HSF	AGAAN
AS$PAX2_61	331 (+)	0	100.00	Pax-2.1, Pax-2.2	CTAAAAGAA
HS$CMYC_13	332 (+)	1	87.50		GAAAAGAAA
Y$MAL2R_01	332 (-)	0	100.00	MIG1	TTTTA
AS$FOXJ1_04	332 (-)	0	100.00	FOXJ1	TTTTA
MAIZE$CYPPDK1_03	334 (+)	0	100.00	Dof2, MNB1a	AAAGAAAG
AS$SRY_02	335 (+)	0	100.00	SRY	AAGAAAG
HS$TNFA_04	336 (+)	0	100.00		AGAAA
I$HSF_01	336 (+)	0	90.00	HSF	AGAAN
F$HSF_01	336 (+)	0	90.00	HSF	AGAAN
CAMV$35SR_02	340 (+)	0	100.00	HBP-1	AGACGT
CAMV$35SR_02	340 (-)	0	100.00	HBP-1	ACGTCT
HS$TF_12	344 (+)	1	87.50	EBP40, EBP45	GTTTGCTTT
AS$FTZ_51	347 (-)	0	100.00	Ftz	AAAGCG
RAT$CYTBE_01	350 (-)	0	100.00		AGCCAAA
MOUSE$AP2_07	351 (+)	0	100.00	NF-1	TTGGC
MOUSE$NE_01	355 (-)	1	88.89	PU.1	AGGGAGGAAG
I$HSF_01	355 (-)	0	90.00	HSF	AGAAN
F$HSF_01	355 (-)	0	90.00	HSF	AGAAN
HS$APOB_10	357 (-)	0	100.00	AP-2alphaA, AP-2alphaB	GGGAAGA
XENLA$RPL14_01	358 (+)	0	100.00	HrpF, XrpFI	CTTCC
Y$TPI_01	358 (+)	0	100.00	GCR1	CTTCC
MOUSE$RPL32_01	358 (-)	0	100.00	f(alpha)-f(epsilon), HrpF, XrpFI	GGAAG
HS$CDC2_06	358 (-)	0	100.00	c-Ets-2	GGGAAG
HS$TNFA_02	358 (-)	0	100.00		GGAAG

HS$TNFA_02	358 (-)	0	100.00		GGAAG
HS$TNFA_03	358 (-)	0	100.00		GGAAG
SV$SV40_63	364 (-)	0	100.00	T-Ag	GCCTA
HS$HNF4A_01	364 (-)	1	87.50	Sp1	GGGGGCCTT
AS$SPZ1_15	364 (-)	1	87.50	Spz1	GGGGGCCTC
HS$M1A11_01	365 (+)	1	87.50	ZFX	AGGCCCCGA
HS$M1A11_01	365 (+)	1	87.50	ZFX	AGGGCCCCA
HS$GG_36	366 (-)	0	100.00	Sp1	GGGGCC
PA$PY_26	367 (+)	0	100.00	T-Ag	GCCCC
MAIZE$ADH11S_06	367 (+)	0	100.00	GCBP-1, Sp1	GCCCC
PA$PY_23	367 (-)	0	100.00	T-Ag	GGGGC
SV$SV40_04	367 (-)	0	100.00	T-Ag	GGGGC
CHICK$ACC_02	367 (-)	1	87.50		GCCGGGGGC
SV$SV40_63	367 (-)	0	100.00	T-Ag	GGGGC
HS$CYCD1_14	368 (+)	0	100.00	Sp1, Sp2, Sp3, Sp4	CCCCC
HS$CYCD1_15	368 (+)	0	100.00	Sp1, Sp2, Sp3, Sp4	CCCCC
Y$GAL3_01	369 (+)	0	100.00	MIG1	CCCCAG
Y$GAL4_01	369 (+)	0	100.00	MIG1	CCCCAG
Y$HAP4_01	369 (+)	0	100.00	MIG1	CCCCAG
HS$ZG_01	369 (-)	1	90.91	Sp1	GGGAGGGTGGGG
CHICK$BG_01	369 (-)	0	100.00	TFIID	GGCTGGGG
HS$TERT_05	370 (+)	1	87.50	Sp1	CCCAGCCCC
HS$TERT_06	370 (+)	1	87.50	Sp1	CCCAGCCCC
SV$SV40_37	370 (-)	0	100.00		CTGGG
HS$MIP_04	370 (-)	0	100.00	Sp1	GGCTGGG
CHICK$STN1_01	371 (+)	0	100.00	LBP-1, MyoD	CCAGC
CHICK$STN1_03	371 (+)	0	100.00	LBP-1, NF-1 (-like proteins)	CCAGC
QUAIL$STN1_02	371 (+)	0	100.00		CCAGCC
HS$GX_WT1_01	372 (-)	1	87.50	WT1 -KTS, WT1 I, WT1 I -KTS, WT1 I-del2, WT1-del2	GTGAGGCTG
MOUSE$MYOD_04	372 (-)	0	100.00	ARP-1, RXR-alpha, RXR-gamma, T3R-alpha	AGGCTG
Y$GAL1_10	373 (+)	0	100.00	GAL4	AGCCT
SV$SV40_01	374 (+)	0	100.00	T-Ag	GCCTC
SV$SV40_02	374 (+)	0	100.00	T-Ag	GCCTC
SV$SV40_63	374 (+)	0	100.00	T-Ag	GCCTC
PA$PY_24	374 (-)	0	100.00	T-Ag	GAGGC
PA$PY_25	374 (-)	0	100.00	T-Ag	GAGGC
SV$SV40_02	374 (-)	0	100.00	T-Ag	GAGGC
AS$ZIC2_07	374 (-)	1	87.50	Zic2	TGGGGGGGC
HT1$HTLV1_08	375 (+)	0	100.00	c-Ets-1 68	CCTCC
HS$CD8A_02	375 (+)	1	87.50	LyF-1	CCTCCCAAG
CHICK$BAG_11	376 (+)	1	87.50	PPYR	CTCCCCCGG
RAT$OCNC_01	376 (-)	1	90.00	COE1, COE2, COE3, Olf-1	TCCCTAGGGAG
MOUSE$M1H2KB_07	377 (+)	0	100.00	MBP-1 (1), NF-kappaB1, NF-kappaB2, NF-kappaB2 precursor	TCCCCA
RAT$5006_01	377 (+)	1	90.00	Olf-1	TCCCCAGGGAG
MOUSE$M1H2KB_02	377 (-)	0	100.00	AP-2alpha, AP-2alphaA, AP-2alphaB	TGGGGA
MOUSE$M1H2KB_07	377 (-)	0	100.00	MBP-1 (1), NF-kappaB1, NF-kappaB2, NF-kappaB2 precursor	TGGGGA
Y$GAL3_01	378 (+)	0	100.00	MIG1	CCCCAG
Y$GAL4_01	378 (+)	0	100.00	MIG1	CCCCAG
Y$HAP4_01	378 (+)	0	100.00	MIG1	CCCCAG
HS$MIP_05	379 (+)	0	100.00	AP-2alphaA, AP-2alphaB	CCCAGGG
SV$SV40_37	379 (-)	0	100.00		CTGGG
MOUSE$THY1_05	381 (-)	1	87.50		CCACCCCTG
HS$CEA_05	382 (-)	1	87.50		TCCATCCCT
AS$ZIC_20	383 (+)	1	87.50	Zic1	GGGATGGCC
HS$MMP3_01	384 (+)	0	100.00	NIP, PEA3	GGATG
Y$ENO2_03	384 (-)	0	100.00	GCR1	CATCC
Y$TPI_03	384 (-)	0	100.00	GCR1	CATCC
MOUSE$RPL32_04	385 (-)	1	87.50	delta factor, factor delta	GCTGCCATC
HS$ALBU_03	387 (+)	0	100.00	NF-1/L	TGGCA
HS$RBP_01	387 (+)	0	100.00	NF-1/L	TGGCA
RAT$A1I3_02	387 (+)	0	100.00	NF-1	TGGCA
RAT$AFEP_11	387 (+)	0	100.00	GR, NF-1	TGGCA
MMTV$MMTV_43	387 (+)	0	100.00	NF-1	TGGCA
AS$MEIS1AHOXA9_11	387 (+)	0	100.00	HOXA9, Meis-1a	TGGCAG
AS$PAX2_38	390 (+)	1	87.50	Pax-2.1, Pax-2.2	CAGAAACTC
AS$PAX2_39	390 (+)	1	87.50	Pax-2.1, Pax-2.2	CAGAAACTC
HS$TNFA_04	391 (+)	0	100.00		AGAAA
I$HSF_01	391 (+)	0	90.00	HSF	AGAAN
F$HSF_01	391 (+)	0	90.00	HSF	AGAAN
AS$AREB6_44	392 (-)	0	100.00	AREB6	GTTTC
AS$AREB6_46	392 (-)	0	100.00	AREB6	GTTTC
AS$AREB6_48	392 (-)	0	100.00	AREB6	GTTTC
AS$AREB6_49	392 (-)	0	100.00	AREB6	GTTTC
AS$AREB6_50	392 (-)	0	100.00	AREB6	GTTTC
AS$AREB6_52	392 (-)	0	100.00	AREB6	GTTTC
AS$AREB6_53	392 (-)	0	100.00	AREB6	GTTTC
RAT$SPI23_02	395 (-)	1	87.50	C/EBPalpha	CCCAGAAAT
I$HSF_01	396 (-)	0	90.00	HSF	AGAAN
F$HSF_01	396 (-)	0	90.00	HSF	AGAAN
AS$STAT5A_68	397 (-)	1	87.50	STAT5A	TACCCAGAA
SV$SV40_37	399 (+)	0	100.00		CTGGG
SOYBN$BCGA_05	400 (-)	0	100.00	SEF3	AACCCA
MOUSE$SHP1_01	401 (+)	0	100.00	FXR, RXR-alpha	GGGTTA
RAT$SHP1_01	401 (+)	0	100.00	FXR, RXR-alpha	GGGTTA
MOUSE$SREBP1C_01	401 (-)	0	100.00	LXR-alpha, LXR-beta, RXR-alpha	TAACCC
MOUSE$SREBP1C_02	401 (-)	0	100.00	LXR-alpha, LXR-beta, RXR-alpha	TAACCC
MOUSE$SREBP1C_03	401 (-)	0	100.00	LXR-alpha, RXR-alpha	TAACCC
CHICK$ACCA_01	401 (-)	0	100.00	LXR-alpha, LXR-beta, RXR-alpha, T3R-alpha	TAACCC
RAT$SHP1_01	401 (-)	0	100.00	FXR, RXR-alpha	TAACCC
PEA$RS3A_03	402 (+)	0	100.00	GT-1, GT-1a, SBF-1	GGTTAA
MOUSE$MYOD_04	407 (+)	0	100.00	ARP-1, RXR-alpha, RXR-gamma, T3R-alpha	AGGCTG
Y$GAL1_10	407 (-)	0	100.00	GAL4	AGCCT
TDNA$NOS_01	411 (+)	0	100.00	ASF-1, OBF3.1, TGA1a, TGA1b	TGAGC
DROME$UBX_08	411 (+)	0	100.00	Zeste	TGAGCG
DROME$E74_07	411 (+)	0	100.00	Zeste	TGAGCG
DROME$E74_08	411 (+)	0	100.00	Zeste	TGAGCG
DROME$WHLO_04	411 (+)	0	100.00	Zeste	TGAGCG
DROME$ZESTE_02	411 (+)	0	100.00	Zeste	TGAGCG
DROME$UBX_06	411 (-)	0	100.00	Zeste	CGCTCA
DROME$EVE_04	412 (-)	0	100.00	GAGA factor	CGCTC
DROME$EVE_08	412 (-)	0	100.00	GAGA factor	CGCTC
DROME$EVE_10	412 (-)	0	100.00	GAGA factor	CGCTC
AS$PAX2_53	413 (+)	1	87.50	Pax-2.1, Pax-2.2	AGCAAACCA
AS$NCX_25	418 (-)	1	88.89	Ncx	GGGCAAGGGT
AS$NCX_26	418 (-)	1	88.89	Ncx	GGGCAATGAT
CHICK$ACRA_06	421 (-)	0	100.00		GCAAT
RSV$RSV_04	421 (-)	0	100.00	C/EBP, C/EBPalpha, C/EBPbeta, C/EBPbeta(p20), C/EBPbeta(p34), C/EBPgamma, EFII	GCAAT
RAT$KINT1_01	421 (-)	1	87.50	C/EBPalpha, C/EBPbeta, C/EBPdelta	TTGGGCAAT
CHICK$BAG_03	423 (+)	0	100.00		TGCCC
RAT$VEGF_02	423 (+)	0	100.00	ER-alpha, ER-beta	TGCCC
CHICK$BAG_03	423 (-)	0	100.00		GGGCA
RAT$NF1_01	423 (-)	0	100.00	LF-A1	GGGCA
HS$CATHD_01	423 (-)	0	100.00	ER-alpha, Sp1	GGGCA
RAT$VEGF_01	423 (-)	0	100.00	ER-alpha, ER-beta	GGGCA
SV$SV40_63	424 (-)	0	100.00	T-Ag	TGGGC
AS$mEMBP_04	427 (+)	0	100.00	EmBP-1a	CACTG
HS$HNF4A_01	427 (-)	0	100.00	Sp1	GAGGCAGTG
AS$ETS1_02	428 (-)	1	87.50	Ets-1 deltaVII, PU.1	GGAGGAAGT
CHICK$ITGB3_01	430 (-)	0	100.00	RXR-beta, VDR	GAGGCA
SV$SV40_01	431 (+)	0	100.00	T-Ag	GCCTC
SV$SV40_02	431 (+)	0	100.00	T-Ag	GCCTC
SV$SV40_63	431 (+)	0	100.00	T-Ag	GCCTC
PA$PY_24	431 (-)	0	100.00	T-Ag	GAGGC
PA$PY_25	431 (-)	0	100.00	T-Ag	GAGGC
SV$SV40_02	431 (-)	0	100.00	T-Ag	GAGGC
HT1$HTLV1_08	432 (+)	0	100.00	c-Ets-1 68	CCTCC
AS$ALFIN1_06	433 (-)	0	100.00	Alfin1	GTGGAG
HT1$HTLV1_08	435 (+)	0	100.00	c-Ets-1 68	CCACC
HS$BG_52	435 (+)	0	100.00		CCACC
HS$BG_14	435 (+)	0	100.00		CCACC
HS$BG_44	435 (+)	0	100.00	CAC-binding protein	CCACC
BPV1$BPV1_24	435 (-)	0	100.00	Sp1	GGTGG
HS$BG_48	435 (-)	0	100.00	CAC-binding protein	GGTGG
MOUSE$CEBPA_06	435 (-)	0	100.00	Sp1	GGTGG
HS$ZG_06	436 (+)	1	87.50	Sp1	CACCACCCC
AS$ZIC2_27	436 (-)	1	87.50	Zic2	GGGCCGGTG
HS$TERT_05	437 (+)	1	87.50	Sp1	CCCAGCCCC
HS$TERT_06	437 (+)	1	87.50	Sp1	CCCAGCCCC
CHICK$STN1_01	438 (+)	0	100.00	LBP-1, MyoD	CCAGC
CHICK$STN1_03	438 (+)	0	100.00	LBP-1, NF-1 (-like proteins)	CCAGC
QUAIL$STN1_02	438 (+)	0	100.00		CCAGCC
PA$PY_26	441 (+)	0	100.00	T-Ag	GCCCC
MAIZE$ADH11S_06	441 (+)	0	100.00	GCBP-1, Sp1	GCCCC
PA$PY_23	441 (-)	0	100.00	T-Ag	GGGGC
SV$SV40_04	441 (-)	0	100.00	T-Ag	GGGGC
CHICK$ACC_02	441 (-)	1	87.50		GCCGGGGGC
SV$SV40_63	441 (-)	0	100.00	T-Ag	GGGGC
HS$CYCD1_14	442 (+)	0	100.00	Sp1, Sp2, Sp3, Sp4	CCCCC
HS$CYCD1_15	442 (+)	0	100.00	Sp1, Sp2, Sp3, Sp4	CCCCC
Y$GAL3_01	443 (+)	0	100.00	MIG1	CCCCAG
Y$GAL4_01	443 (+)	0	100.00	MIG1	CCCCAG
Y$HAP4_01	443 (+)	0	100.00	MIG1	CCCCAG
SV$SV40_36	443 (-)	0	100.00	AP-2alphaA, AP-2alphaB, AP-2beta	TGCTGGGG
SV$SV40_37	444 (-)	0	100.00		CTGGG
CHICK$STN1_01	445 (+)	0	100.00	LBP-1, MyoD	CCAGC
CHICK$STN1_03	445 (+)	0	100.00	LBP-1, NF-1 (-like proteins)	CCAGC
MOUSE$M2EAK_03	446 (+)	0	100.00	NF-Y	CAGCA
MOUSE$THY1_06	446 (+)	0	100.00		CAGCAA
AS$HOXA3_08	446 (-)	1	87.50	HOXA3	CCTTTGTTG
HS$CYCD1_08	447 (-)	0	100.00	TCF-4E	CTTTGCT
Y$GLK1_03	450 (+)	1	87.50	MED8	AAATGCACG
EBV$IR4_05	453 (+)	0	100.00	R	GGCAC
EBV$IR4_06	453 (+)	0	100.00	R	GGCAC
XENLA$U2SN_11	453 (-)	0	100.00		GTGCC
RAT$CYTOP_04	455 (+)	0	100.00	AhR, Arnt	CACGC
AS$AHRARNT_50	455 (-)	0	100.00	AhR, Arnt	GCGTG
CHICK$GATA1_09	458 (+)	1	88.89		GCGGGCGGGG
HSV1$IE3_06	461 (+)	0	100.00	Sp1	GGCGGG
RAT$NF1_02	461 (+)	0	100.00	Sp1	GGCGGG
HS$NPY_02	461 (+)	0	100.00	Sp1	GGCGGG
HS$GAST_02	461 (+)	1	88.89		GGCGGGGTGG
RAT$NEU_03	461 (+)	1	88.89		GGTGGGGGGG
HS$CDC25C_04	461 (+)	0	100.00		GGCGG
HS$MIP_02	461 (+)	0	100.00	Sp1	GGCGGG
HS$TK_06	461 (+)	0	100.00	Sp1	GGCGGGG
HS$TK_09	461 (-)	0	100.00	Sp1	CCCCGCC
HS$P21WAF1_06	461 (-)	0	100.00	SMAD-3, SMAD-4	CCCGCC
HS$P21WAF1_07	461 (-)	0	100.00		CCCGCC
MOUSE$HOX14_01	462 (+)	1	87.50	Krox-20, Krox-24	GCGGGGGCG
HS$IGF2_02	462 (+)	1	87.50	WT1	GCGGGGGCG
AS$NGFIC_01	462 (+)	1	87.50	NGFI-C	GCGGGGGCG
MOUSE$JUND_05	462 (+)	1	87.50	Sp1	GCGGGGGCG
RAT$IGF1R_04	462 (+)	1	87.50	WT1, WT1 -KTS, WT1 I, WT1 I -KTS	GCGGGGGCG
Y$SUC2_01	462 (-)	0	100.00	MIG1	CCCCGC
WTZFP$CONS	462 (-)	1	87.50	WT1, WT1 -KTS, WT1 I, WT1 I -KTS, WT1 I-del2, WT1-del2	CGCCCCCGC
HS$IGF2_01	462 (-)	1	87.50	WT1	CGCCCCCGC
HS$IGF2_03	462 (-)	1	87.50	WT1	CGCCCCCGC
Y$PDC1_02	462 (-)	0	100.00	MIG1	CCCCGC
AS$ZIC2_07	463 (+)	1	87.50	Zic2	TGGGGGGGC
AS$ZIC2_09	463 (+)	1	87.50	Zic2	GGGGGGGGC
AS$ZIC3_05	463 (+)	1	87.50	Zic3	CGGGGGGTC
AS$VDR_06	463 (+)	0	100.00	VDR	CGGGGG
MOUSE$HOX14_01	464 (+)	1	87.50	Krox-20, Krox-24	GCGGGGGCG
HS$HSP70_10	464 (+)	1	88.89	Sp1	GGGGCGGCGC
HSV1$TK_03	464 (+)	1	88.89	Sp1	GGGGCGGCGC
AS$BGP1_02	464 (+)	0	100.00	BGP1	GGGGGGG
HS$IGF2_02	464 (+)	1	87.50	WT1	GCGGGGGCG
AS$NGFIC_01	464 (+)	1	87.50	NGFI-C	GCGGGGGCG
MOUSE$JUND_05	464 (+)	1	87.50	Sp1	GCGGGGGCG
LP$LPS1B_02	464 (+)	1	87.50	IF1	TGGGGGGCG
RAT$PARP_01	464 (+)	1	90.00		GGGGTGGCGCC
RAT$IGF1R_01	464 (+)	1	87.50	WT1, WT1 -KTS, WT1 I, WT1 I -KTS	GTGGGGGCG
RAT$IGF1R_04	464 (+)	1	87.50	WT1, WT1 -KTS, WT1 I, WT1 I -KTS	GCGGGGGCG
AS$SPZ1_08	464 (+)	1	87.50	Spz1	GGGGGGTCG
AS$ZIC3_17	464 (+)	1	87.50	Zic3	GGGGGGGTG
WTZFP$CONS	464 (-)	1	87.50	WT1, WT1 -KTS, WT1 I, WT1 I -KTS, WT1 I-del2, WT1-del2	CGCCCCCGC
HS$IGF2_01	464 (-)	1	87.50	WT1	CGCCCCCGC
HS$IGF2_03	464 (-)	1	87.50	WT1	CGCCCCCGC
HS$CYCD1_14	464 (-)	0	100.00	Sp1, Sp2, Sp3, Sp4	CCCCC
HS$CYCD1_15	464 (-)	0	100.00	Sp1, Sp2, Sp3, Sp4	CCCCC
AS$ZIC2_09	465 (+)	1	87.50	Zic2	GGGGGGGGC
AS$ZAC_01	465 (+)	1	88.89	ZAC	GGGGGGCCCC
HS$CYCD1_14	465 (-)	0	100.00	Sp1, Sp2, Sp3, Sp4	CCCCC
HS$CYCD1_15	465 (-)	0	100.00	Sp1, Sp2, Sp3, Sp4	CCCCC
YL$XPR2_03	466 (-)	0	100.00		CGCCCCC
HS$CYCD1_14	466 (-)	0	100.00	Sp1, Sp2, Sp3, Sp4	CCCCC
HS$CYCD1_15	466 (-)	0	100.00	Sp1, Sp2, Sp3, Sp4	CCCCC
PA$PY_23	467 (+)	0	100.00	T-Ag	GGGGC
SV$SV40_04	467 (+)	0	100.00	T-Ag	GGGGC
SV$SV40_63	467 (+)	0	100.00	T-Ag	GGGGC
AS$SPZ1_18	467 (+)	1	87.50	Spz1	GGGGCGCCG
HS$DPOLB_03	467 (-)	1	87.50		GTGCGCCCC
PA$PY_26	467 (-)	0	100.00	T-Ag	GCCCC
MAIZE$ADH11S_06	467 (-)	0	100.00	GCBP-1, Sp1	GCCCC
HS$APP_01	468 (+)	0	100.00		GGGCGC
HS$CD11B_01	468 (-)	0	100.00	Sp1	CGCCC
AS$ZIC3_04	468 (-)	1	87.50	Zic3	TGGGTGCCC
HS$AAC_14	469 (-)	1	90.00	Sp1	GGCAGGGCGCC
HS$APP_01	470 (-)	0	100.00		GGGCGC
HS$CD11B_01	471 (+)	0	100.00	Sp1	CGCCC
CHICK$BAC_06	471 (-)	1	88.89	GCF	GGGCGGGGCG
AS$SP1_17	471 (-)	1	88.89	Sp1	GGGCGGGGCG
HS$A11COL_01	472 (+)	1	88.89	Sp1	GCCCCGCCCC
HS$RAS1_06	472 (+)	1	88.89	Sp1	GCCCCGCCCC
RAT$ME_02	472 (+)	1	88.89	Sp1	GCCCCGCCCC
HS$ASCC_03	472 (+)	1	88.89	Sp1	GCCCTGCCCC
HS$CYCA_01	472 (+)	1	88.89		GCCCCGCCCC
AD2$E1B_03	472 (-)	1	88.89	BTEB1, MAZ, Sp1	GGGGCGGGGC
HS$RAS1_03	472 (-)	1	88.89	Sp1	GGGGCGGGGC
HS$RAS1_04	472 (-)	1	88.89	Sp1	GGGGCGGGGC
AD2$E1B_02	472 (-)	1	88.89	Sp1, TBP, TFIID	GGGGCGGGGC
AS$SP1_02	472 (-)	1	88.89	Sp1	GGGGCGGGGC
AS$SP1_13	472 (-)	1	88.89	Sp1	GGGGCAGGGC
HS$ASCC_01	472 (-)	1	88.89	Sp1	GGGGCCGGGC
HS$DHFR_05	472 (-)	1	88.89	Sp1	GGGGCGGGGC
HS$DHFR_06	472 (-)	1	88.89	Sp1	GGGGCGGGGC
AS$SP1_36	472 (-)	1	88.89	Sp1	GGGGCAGGGC
SV$SV40_63	472 (-)	0	100.00	T-Ag	TGGGC
HS$TERT_05	473 (+)	0	100.00	Sp1	CCCAGCCCC
HS$TERT_06	473 (+)	0	100.00	Sp1	CCCAGCCCC
MOUSE$A11COL_01	473 (-)	1	88.89	CBF (2), IF2	GGGGGCCGGG
HS$MT2A_10	473 (-)	1	87.50	Sp1	GGGGCGGGG
SV$SV40_37	473 (-)	0	100.00		CTGGG
RAT$GSTP_09	473 (-)	1	87.50		GGGGCGGGG
MOUSE$PFK_05	473 (-)	1	87.50		GGGGCGGGG
HS$FN_07	473 (-)	1	87.50	Sp1	GGGGCGGGG
HS$MIP_04	473 (-)	0	100.00	Sp1	GGCTGGG
CHICK$STN1_01	474 (+)	0	100.00	LBP-1, MyoD	CCAGC
CHICK$STN1_03	474 (+)	0	100.00	LBP-1, NF-1 (-like proteins)	CCAGC
QUAIL$STN1_02	474 (+)	0	100.00		CCAGCC
AS$ZIC3_17	475 (-)	1	87.50	Zic3	GGGGGGGTG
LP$LPS1B_01	476 (+)	1	87.50		AGCCCCCCT
RAT$IGF1R_07	476 (+)	1	87.50	WT1 -KTS, WT1 I -KTS	AGCCCCCGC
AS$ZIC2_20	476 (-)	1	87.50	Zic2	GGGGGGGTT
PA$PY_26	477 (+)	0	100.00	T-Ag	GCCCC
MAIZE$ADH11S_06	477 (+)	0	100.00	GCBP-1, Sp1	GCCCC
PA$PY_23	477 (-)	0	100.00	T-Ag	GGGGC
SV$SV40_04	477 (-)	0	100.00	T-Ag	GGGGC
SV$SV40_63	477 (-)	0	100.00	T-Ag	GGGGC
AS$ZIC_16	477 (-)	1	87.50	Zic1	TGCGGGGGC
AS$ZIC2_07	477 (-)	0	100.00	Zic2	TGGGGGGGC
AS$ZIC2_09	477 (-)	1	87.50	Zic2	GGGGGGGGC
AS$ZIC3_20	477 (-)	1	87.50	Zic3	TGGGGGAGC
HS$CYCD1_14	478 (+)	0	100.00	Sp1, Sp2, Sp3, Sp4	CCCCC
HS$CYCD1_15	478 (+)	0	100.00	Sp1, Sp2, Sp3, Sp4	CCCCC
AS$BGP1_02	478 (-)	0	100.00	BGP1	GGGGGGG
RAT$NEU_03	478 (-)	1	88.89		GGTGGGGGGG
HS$CYCD1_14	479 (+)	0	100.00	Sp1, Sp2, Sp3, Sp4	CCCCC
HS$CYCD1_15	479 (+)	0	100.00	Sp1, Sp2, Sp3, Sp4	CCCCC
HS$CYCD1_14	480 (+)	0	100.00	Sp1, Sp2, Sp3, Sp4	CCCCC
HS$CYCD1_15	480 (+)	0	100.00	Sp1, Sp2, Sp3, Sp4	CCCCC
Y$GAL3_01	481 (+)	0	100.00	MIG1	CCCCAG
Y$GAL4_01	481 (+)	0	100.00	MIG1	CCCCAG
Y$HAP4_01	481 (+)	0	100.00	MIG1	CCCCAG
SV$SV40_36	481 (-)	0	100.00	AP-2alphaA, AP-2alphaB, AP-2beta	TGCTGGGG
SV$SV40_37	482 (-)	0	100.00		CTGGG
CHICK$STN1_01	483 (+)	0	100.00	LBP-1, MyoD	CCAGC
CHICK$STN1_03	483 (+)	0	100.00	LBP-1, NF-1 (-like proteins)	CCAGC
MOUSE$M2EAK_03	484 (+)	0	100.00	NF-Y	CAGCA
MOUSE$THY1_06	484 (+)	0	100.00		CAGCAA
MOUSE$AFEP_15	485 (-)	0	100.00	C/EBPalpha	GTTTGCT
MOUSE$AFEP_16	485 (-)	0	100.00	HNF-1A	GTTTGCT
AS$AREB6_42	487 (-)	0	100.00	AREB6	GTTTG
AS$AREB6_43	487 (-)	0	100.00	AREB6	GTTTG
AS$AREB6_45	487 (-)	0	100.00	AREB6	GTTTG
AS$AREB6_47	487 (-)	0	100.00	AREB6	GTTTG
AS$AREB6_51	487 (-)	0	100.00	AREB6	GTTTG
HS$PR264_05	488 (-)	0	100.00	c-Myb	GCGTTT
AS$SPZ1_10	488 (-)	1	87.50	Spz1	GGAGCGTTA
AS$ZIC_28	489 (-)	1	87.50	Zic1	CGGATCGTT
DROME$EVE_04	491 (+)	0	100.00	GAGA factor	CGCTC
DROME$EVE_08	491 (+)	0	100.00	GAGA factor	CGCTC
DROME$EVE_10	491 (+)	0	100.00	GAGA factor	CGCTC
RAT$MLC_04	492 (-)	0	100.00	MAPF2, YY1	GGAGC
Y$CHA1_01	494 (+)	0	100.00	CHA4	TCCGC
Y$CHA1_01	494 (-)	0	100.00	CHA4	GCGGA
HS$CYCD1_14	495 (+)	0	100.00	Sp1, Sp2, Sp3, Sp4	CCGCG
HS$CDC2_09	495 (-)	0	100.00		CGCGG
HS$CYCA_05	495 (-)	0	100.00		CGCGG
HS$CDC2_09	496 (+)	0	100.00		CGCGG
HS$CYCA_05	496 (+)	0	100.00		CGCGG
HS$CYCD1_14	496 (-)	0	100.00	Sp1, Sp2, Sp3, Sp4	CCGCG
CHICK$BLCR_02	497 (+)	0	100.00	NF-E4, Sp1	GCGGC
HS$CAVEOLIN1_03	497 (+)	0	100.00	Sp1	GCGGCC
AS$RAR_14	498 (-)	0	100.00	RAR-alpha1, RXR-alpha	AGGCCG
AS$ZIC3_19	499 (-)	1	87.50	Zic3	CGGGAGGCC
SV$SV40_01	500 (+)	0	100.00	T-Ag	GCCTC
SV$SV40_02	500 (+)	0	100.00	T-Ag	GCCTC
SV$SV40_63	500 (+)	0	100.00	T-Ag	GCCTC
PA$PY_24	500 (-)	0	100.00	T-Ag	GAGGC
PA$PY_25	500 (-)	0	100.00	T-Ag	GAGGC
SV$SV40_02	500 (-)	0	100.00	T-Ag	GAGGC
H4TF1$CONS	500 (-)	1	87.50	H4TF-1	GGGGGAGGG
AS$ZIC2_09	500 (-)	1	87.50	Zic2	GGGGGGGGC
HT1$HTLV1_08	501 (+)	0	100.00	c-Ets-1 68	CCTCC
EGR2$CONS	501 (+)	1	88.89	EGR2	CCGCCCCCGC
AS$ZIC_25	501 (-)	1	87.50	Zic1	CGGGTGAGG
CHICK$BAG_11	502 (+)	1	87.50	PPYR	CTCCCCCGG
WTZFP$CONS	502 (+)	1	87.50	WT1, WT1 -KTS, WT1 I, WT1 I -KTS, WT1 I-del2, WT1-del2	CGCCCCCGC
HS$IGF2_01	502 (+)	1	87.50	WT1	CGCCCCCGC
HS$IGF2_03	502 (+)	1	87.50	WT1	CGCCCCCGC
MOUSE$HOX14_01	502 (-)	1	87.50	Krox-20, Krox-24	GCGGGGGCG
HS$IGF2_02	502 (-)	1	87.50	WT1	GCGGGGGCG
AS$NGFIC_01	502 (-)	1	87.50	NGFI-C	GCGGGGGCG
MOUSE$JUND_05	502 (-)	1	87.50	Sp1	GCGGGGGCG
RAT$IGF1R_04	502 (-)	1	87.50	WT1, WT1 -KTS, WT1 I, WT1 I -KTS	GCGGGGGCG
CHICK$CA2_02	503 (+)	0	100.00	RXR-alpha, VDR	TCCCCC
AS$ZIC_16	503 (-)	1	87.50	Zic1	TGCGGGGGC
HS$CYCD1_14	504 (+)	0	100.00	Sp1, Sp2, Sp3, Sp4	CCCCC
HS$CYCD1_15	504 (+)	0	100.00	Sp1, Sp2, Sp3, Sp4	CCCCC
AS$VDR_06	504 (-)	0	100.00	VDR	CGGGGG
Y$SUC2_01	505 (+)	0	100.00	MIG1	CCCCGC
Y$PDC1_02	505 (+)	0	100.00	MIG1	CCCCGC
HS$TCRA_08	507 (+)	0	100.00	c-Ets-1, LEF-1, PEBP2alphaA1	CCGCA
AS$FTZ_54	511 (+)	0	100.00	Ftz	AGACCA
AS$ZIC2_06	512 (-)	1	87.50	Zic2	CACGTGGTC
AS$RITA1_08	513 (+)	1	88.89	RITA-1	ACCACGTGGT
AS$BZIPGB_09	513 (+)	1	88.89	TAF-1	ACCACGTGGT
MOUSE$IAP_01	513 (-)	0	100.00		GTGGT
MOUSE$IAP_03	513 (-)	0	100.00	EBP-80	GTGGT
AS$RITA1_08	513 (-)	1	88.89	RITA-1	ACCACGTGGT
AS$BZIPGB_09	513 (-)	1	88.89	TAF-1	ACCACGTGGT
MAIZE$ADH1P_01	514 (+)	0	100.00		CGTGG
MAIZE$ADH1P_03	514 (-)	0	100.00		CGTGG
HSV2$VMW65_01	515 (+)	0	100.00	USF1	CACGAG
MOUSE$HES1_02	515 (+)	0	100.00	HES-1	CACGAG
MOUSE$HES1_03	515 (+)	0	100.00	HES-1	CACGAG
CHICK$BAG_07	518 (+)	1	87.50		GAGGAGGGG
HS$APOE_06	518 (-)	1	87.50		CCCCACCTC
YL$XPR2_04	518 (-)	1	88.89		CGCCCACCTC
BPV1$BPV1_24	520 (+)	0	100.00	Sp1	GGTGG
HS$BG_43	520 (+)	0	100.00	CAC-binding protein	GGTGGGG
HS$BG_48	520 (+)	0	100.00	CAC-binding protein	GGTGG
MOUSE$CEBPA_06	520 (+)	0	100.00	Sp1	GGTGG
HS$GPB_02	520 (+)	0	100.00	Sp1	GGTGGG
AS$ZIC2_09	520 (+)	1	87.50	Zic2	GGGGGGGGC
HT1$HTLV1_08	520 (-)	0	100.00	c-Ets-1 68	CCACC
HS$BG_52	520 (-)	0	100.00		CCACC
HS$BG_14	520 (-)	0	100.00		CCACC
HS$BG_44	520 (-)	0	100.00	CAC-binding protein	CCACC
MOUSE$JUND_07	521 (+)	1	87.50		GCGGGGGGC
Y$MEL1_02	521 (+)	0	100.00	MIG1	GTGGGG
Y$MAL61_03	521 (+)	0	100.00	MIG1	GTGGGG
Y$MAL61_04	521 (+)	0	100.00	MIG1	GTGGGG
RAT$IGF1R_01	521 (+)	1	87.50	WT1, WT1 -KTS, WT1 I, WT1 I -KTS	GTGGGGGCG
RAT$IGF1R_03	521 (+)	1	87.50	WT1 -KTS, WT1 I -KTS	GCGGGGGCC
RAT$IGF1R_08	521 (+)	1	87.50	WT1 -KTS, WT1 I -KTS	GCGGGGGCG
MESAT$PRP2_01	521 (+)	0	100.00	Alfin1	GTGGGGG
LP$LPS1B_02	522 (+)	1	87.50	IF1	TGGGGGGCG
AS$ZIC3_12	522 (+)	1	87.50	Zic3	TGGGTGCCG
AS$SPZ1_15	523 (+)	1	87.50	Spz1	GGGGGCCTC
HS$CYCD1_14	523 (-)	0	100.00	Sp1, Sp2, Sp3, Sp4	CCCCC
HS$CYCD1_15	523 (-)	0	100.00	Sp1, Sp2, Sp3, Sp4	CCCCC
PA$PY_23	524 (+)	0	100.00	T-Ag	GGGGC
SV$SV40_04	524 (+)	0	100.00	T-Ag	GGGGC
HS$GG_36	524 (+)	0	100.00	Sp1	GGGGCC
SV$SV40_63	524 (+)	0	100.00	T-Ag	GGGGC
PA$PY_26	524 (-)	0	100.00	T-Ag	GCCCC
MAIZE$ADH11S_06	524 (-)	0	100.00	GCBP-1, Sp1	GCCCC
AS$ZIC2_27	525 (+)	1	87.50	Zic2	GGGCCGGTG
HS$CAVEOLIN1_03	526 (-)	0	100.00	Sp1	GCGGCC
CHICK$BLCR_02	527 (-)	0	100.00	NF-E4, Sp1	GCGGC
CHICK$STN1_01	530 (-)	0	100.00	LBP-1, MyoD	CCAGC
CHICK$STN1_03	530 (-)	0	100.00	LBP-1, NF-1 (-like proteins)	CCAGC
SV$SV40_37	531 (+)	0	100.00		CTGGG
Y$GAL3_01	531 (-)	0	100.00	MIG1	CCCCAG
Y$GAL4_01	531 (-)	0	100.00	MIG1	CCCCAG
Y$HAP4_01	531 (-)	0	100.00	MIG1	CCCCAG
HS$CD8A_02	531 (-)	1	87.50	LyF-1	CCTCCCAAG
MOUSE$M1H2KB_02	532 (+)	0	100.00	AP-2alpha, AP-2alphaA, AP-2alphaB	TGGGGA
MOUSE$M1H2KB_07	532 (+)	0	100.00	MBP-1 (1), NF-kappaB1, NF-kappaB2, NF-kappaB2 precursor	TGGGGA
H4TF1$CONS	532 (+)	1	87.50	H4TF-1	GGGGGAGGG
MOUSE$M1H2KB_07	532 (-)	0	100.00	MBP-1 (1), NF-kappaB1, NF-kappaB2, NF-kappaB2 precursor	TCCCCA
HS$AAC_11	532 (-)	1	90.00	Sp1	GCCCCTCCCCA
HS$MIP_03	533 (+)	1	87.50	Sp1	GGGGAGGGG
HS$DHFR_08	533 (+)	1	87.50	Sp1	GGGGCGGGC
AS$ZIC_24	533 (+)	1	87.50	Zic1	GAGGAGGGC
AS$ZIC2_09	533 (+)	1	87.50	Zic2	GGGGGGGGC
HS$ZG_06	533 (-)	1	87.50	Sp1	CCCCTCCCC
MOUSE$CMYC_04	534 (+)	0	100.00	MAZ	GGGAGGG
MOUSE$CD4_04	534 (+)	0	100.00	MAZ	GGGAGGG
AS$ZIC_20	534 (+)	1	87.50	Zic1	GGGATGGCC
MOUSE$THY1_04	534 (-)	0	100.00	R1, R2, Sp1	CCCTCCC
HS$AAC_16	535 (-)	1	88.89	Sp1	GCGCCCCTCC
HT1$HTLV1_08	535 (-)	0	100.00	c-Ets-1 68	CCTCC
CTCF$CONS	536 (-)	0	100.00	CTCF	CCCTC
HS$EGFR_22	539 (-)	1	88.89	GCF	CAGCGCGGCC
RAT$ATP1A2_01	540 (-)	1	87.50		CAGCTGGGC
HS$APOE_06	543 (-)	1	87.50		CCCCACCTC
CHICK$ACC_02	545 (+)	1	87.50		GCCGGGGGC
CHICK$STN1_01	545 (-)	0	100.00	LBP-1, MyoD	CCAGC
CHICK$STN1_03	545 (-)	0	100.00	LBP-1, NF-1 (-like proteins)	CCAGC
SV$SV40_37	546 (+)	0	100.00		CTGGG
Y$GAL3_01	546 (-)	0	100.00	MIG1	CCCCAG
Y$GAL4_01	546 (-)	0	100.00	MIG1	CCCCAG
Y$HAP4_01	546 (-)	0	100.00	MIG1	CCCCAG
RAT$CAT_01	547 (+)	1	87.50		TGGGGGGAG
AS$CF1_04	548 (+)	1	87.50	CF1	GGGGTCAGC
AS$CF1_32	548 (+)	1	87.50	CF1	GGGGTCAGC
HS$CYCD1_14	548 (-)	0	100.00	Sp1, Sp2, Sp3, Sp4	CCCCC
HS$CYCD1_15	548 (-)	0	100.00	Sp1, Sp2, Sp3, Sp4	CCCCC
PA$PY_23	549 (+)	0	100.00	T-Ag	GGGGC
SV$SV40_04	549 (+)	0	100.00	T-Ag	GGGGC
SV$SV40_63	549 (+)	0	100.00	T-Ag	GGGGC
HS$OC_03	549 (+)	0	100.00	VDR	GGGGCA
PA$PY_26	549 (-)	0	100.00	T-Ag	GCCCC
HS$PBGD_05	549 (-)	0	100.00	CAC-binding protein, NF-E2	CTGCCCC
MAIZE$ADH11S_06	549 (-)	0	100.00	GCBP-1, Sp1	GCCCC
CHICK$BAG_03	550 (+)	0	100.00		GGGCA
RAT$NF1_01	550 (+)	0	100.00	LF-A1	GGGCA
PIG$UPA_09	550 (+)	0	100.00	Sp1	GGGCAG
HS$CATHD_01	550 (+)	0	100.00	ER-alpha, Sp1	GGGCA
RAT$VEGF_01	550 (+)	0	100.00	ER-alpha, ER-beta	GGGCA
CHICK$BAG_03	550 (-)	0	100.00		TGCCC
RAT$VEGF_02	550 (-)	0	100.00	ER-alpha, ER-beta	TGCCC
DROME$ADH_14	552 (+)	0	100.00	Adf-1	GCAGC
DROME$ADH_06	552 (-)	0	100.00	Adf-1	GCTGC
HS$CMYC_16	552 (-)	0	100.00	CTCF	GCTGC
DROME$EVE_10	556 (+)	0	100.00	GAGA factor	CTCGC
RAT$ME_05	556 (+)	0	100.00		CTCGCCACCC
FACT1$CONS	557 (-)	0	100.00	F-ACT1	TGGCGA
HS$ZG_06	558 (+)	1	87.50	Sp1	CACCACCCC
HIV1$HIV1_26	558 (-)	1	87.50	HMBP	GGAGTGGCG
HT1$HTLV1_08	560 (+)	0	100.00	c-Ets-1 68	CCACC
MOUSE$THY1_05	560 (+)	1	87.50		CCACCCCTG
HS$BG_52	560 (+)	0	100.00		CCACC
HS$BG_14	560 (+)	0	100.00		CCACC
HS$BG_44	560 (+)	0	100.00	CAC-binding protein	CCACC
HS$MIP_07	560 (+)	0	100.00	Sp1	CCACCC
MOUSE$P53_09	560 (+)	0	100.00	ETF	CCACCC
HS$GAST_02	560 (-)	1	88.89		GGCGGGGTGG
BPV1$BPV1_24	560 (-)	0	100.00	Sp1	GGGTGG
BPV1$BPV1_24	560 (-)	0	100.00	Sp1	GGTGG
HS$AG_12	560 (-)	0	100.00	CACCC-binding factor	GGGTGG
AS$RAR_07	560 (-)	0	100.00	RAR-alpha1, RXR-alpha	GGGTGG
HS$BG_48	560 (-)	0	100.00	CAC-binding protein	GGTGG
MOUSE$CEBPA_06	560 (-)	0	100.00	Sp1	GGTGG
AS$SPZ1_09	560 (-)	1	87.50	Spz1	CCGGGGTAG
HS$BG_01	561 (+)	0	100.00	CACCC-binding factor	CACCC
HS$GG_13	561 (+)	0	100.00	gammaCAC1, gammaCAC2	CACCC
HS$GG_14	561 (+)	0	100.00	CACCC-binding factor, Sp1	CACCC
HS$GP2B_01	561 (+)	0	100.00	CACCC-binding factor	CACCC
RAT$TOA_02	561 (+)	0	100.00	CACCC-binding factor	CACCC
HS$EG_06	561 (+)	0	100.00	Sp1	CACCC
AS$CACCC_01	561 (+)	0	100.00	CACCC-binding factor	CACCC
HS$AG_15	561 (-)	0	100.00	CAC-binding protein	GGGGTG
MAIZE$ADH1P_02	563 (+)	0	100.00		CCCCGG
Y$MAL2R_01	563 (+)	0	100.00	MIG1	CCCCGG
Y$MAL63_01	563 (+)	0	100.00	MIG1	CCCCGG
HS$GMCSF_10	567 (-)	0	100.00	NF-ATp	GGAGCC
RAT$MLC_04	568 (-)	0	100.00	MAPF2, YY1	GGAGC
DROME$EVE_10	575 (-)	0	100.00	GAGA factor	CTCGC
Y$ILV2_02	578 (+)	1	87.50	repressor of CAR1 expression	AGCCGCCGG
DROME$ADH_06	579 (+)	0	100.00	Adf-1	GCTGC
HS$CMYC_16	579 (+)	0	100.00	CTCF	GCTGC
DROME$ADH_14	579 (-)	0	100.00	Adf-1	GCAGC
Y$ACS1_03	581 (+)	1	87.50	UME6	TGCCGCCGA
Y$HSP70_02	581 (+)	1	87.50	URSF	TCGGCGGCA
Y$SSA1_01	581 (-)	1	87.50	repressor of CAR1 expression	TCGGCGGCA
CHICK$AAC_06	585 (+)	0	100.00	delta factor, F-ACT1, MAPF2, YY1	GGCGAC
CHICK$AAC_07	585 (+)	0	100.00	YY1	GGCGAC
AS$RAR_09	587 (-)	0	100.00	RAR-alpha1, RXR-alpha	AGGTCG
AS$ZIC_11	592 (+)	1	87.50	Zic1	TACGCGGTC
HS$CYCA_11	593 (+)	0	100.00	E2F+p107	TCGCGG
HS$CDC2_09	594 (+)	0	100.00		CGCGG
HS$CYCA_05	594 (+)	0	100.00		CGCGG
HS$CYCD1_14	594 (-)	0	100.00	Sp1, Sp2, Sp3, Sp4	CCGCG
AS$ZIC3_23	595 (+)	1	87.50	Zic3	GGGGTCCTC
HS$HH4_02	597 (+)	0	100.00	H4TF-2	GGTCC
AS$TR_11	597 (+)	0	100.00	T3R-alpha	GGTCC
PA$PY_12	600 (-)	0	100.00		AGAGG
RAT$POMC_03	601 (-)	0	100.00	GR	CAGAG
AS$RAR_12	604 (+)	0	100.00	RAR-alpha1, RXR-alpha	TGGTCC
HS$HH4_02	605 (+)	0	100.00	H4TF-2	GGTCC
AS$TR_11	605 (+)	0	100.00	T3R-alpha	GGTCC
HS$VEGF_02	609 (+)	0	100.00		CAGGT
MOUSE$CRABP1_01	610 (-)	0	100.00	TR2-11	GGACCT
HS$HH4_02	611 (+)	0	100.00	H4TF-2	GGTCC
AS$TR_11	611 (+)	0	100.00	T3R-alpha	GGTCC
EBV$IR4_04	612 (-)	0	100.00	R	GGGAC
EBV$IR4_05	612 (-)	0	100.00	R	GGGAC
AS$ZIC3_19	617 (-)	1	87.50	Zic3	CGGGAGGCC
XENLA$RPL14_01	619 (+)	0	100.00	HrpF, XrpFI	CTTCC
Y$TPI_01	619 (+)	0	100.00	GCR1	CTTCC
MOUSE$RPL32_01	619 (-)	0	100.00	f(alpha)-f(epsilon), HrpF, XrpFI	GGAAG
HS$CDC2_06	619 (-)	0	100.00	c-Ets-2	GGGAAG
HS$TNFA_02	619 (-)	0	100.00		GGAAG
HS$TNFA_03	619 (-)	0	100.00		GGAAG
CHICK$BAG_11	623 (-)	1	87.50	PPYR	CTCCCCCGG
RAT$CYCS_03	624 (+)	1	88.89	Sp1	AGGGCGAGGA
AS$ZIC_25	624 (+)	1	87.50	Zic1	CGGGTGAGG
MOUSE$SREBP1C_02	624 (-)	0	100.00	LXR-alpha, LXR-beta, RXR-alpha	CGCCCG
HS$CYCD1_15	624 (-)	0	100.00	Sp1, Sp2, Sp3, Sp4	GCCCG
HS$CD11B_01	625 (-)	0	100.00	Sp1	CGCCC
DROME$EVE_10	627 (-)	0	100.00	GAGA factor	CTCGC
CHICK$BAG_07	629 (+)	1	87.50		GAGGAGGGG
HS$SREC_02	629 (-)	1	88.89	Sp1	CCCGCCCCTC
RAT$GSTP_07	631 (+)	1	87.50	NF-1A, SF-A	GGAGCAGGA
RAT$MLC_04	631 (+)	0	100.00	MAPF2, YY1	GGAGC
DROME$EVE_04	632 (-)	0	100.00	GAGA factor	CGCTC
DROME$EVE_08	632 (-)	0	100.00	GAGA factor	CGCTC
DROME$EVE_10	632 (-)	0	100.00	GAGA factor	CGCTC
EG$CCR_01	633 (+)	0	100.00	LMC1, LMC2	AGCGGG
H4TF1$CONS	634 (+)	1	87.50	H4TF-1	GGGGGAGGG
HS$CMYC_18	634 (+)	1	87.50	MAZ, Sp1	GAGGGAGGG
HS$CMYC_17	635 (-)	0	100.00	CNBP, hnRNP K, MAZ, MAZi, Sp1	CCCTCCCG
MOUSE$CMYC_04	636 (+)	0	100.00	MAZ	GGGAGGG
MOUSE$CD4_04	636 (+)	0	100.00	MAZ	GGGAGGG
HS$GX_WT1_02	636 (-)	0	100.00	WT1 -KTS, WT1 I, WT1 I -KTS, WT1 I-del2, WT1-del2	CTCCCTCCC
MOUSE$THY1_04	636 (-)	0	100.00	R1, R2, Sp1	CCCTCCC
HT1$HTLV1_08	637 (-)	0	100.00	c-Ets-1 68	CCTCC
RAT$ALAL_01	638 (+)	1	87.50	MAF	GAGGGAAGT
SHEEP$BLG_08	638 (+)	1	87.50	MAF	GAGGGAAGT
HS$CMYC_18	638 (+)	1	87.50	MAZ, Sp1	GAGGGAGGG
CTCF$CONS	638 (-)	0	100.00	CTCF	CCCTC
HS$CS1_04	639 (+)	0	100.00	T3R	AGGGAG
HS$P21WAF1_05	639 (+)	0	100.00	RXR-alpha, VDR	AGGGAG
AS$ZIC2_19	639 (+)	1	87.50	Zic2	AGGAAGGTC
AS$ZIC3_26	639 (+)	1	87.50	Zic3	AGGGAGTTC
AS$VDR_08	639 (+)	0	100.00	VDR	AGGGAG
HS$SREC_01	639 (+)	0	100.00	EZF-2	AGGGAGG
HS$SREC_01	639 (-)	0	100.00	EZF-2	CCTCCCT
AS$SPZ1_08	640 (+)	1	87.50	Spz1	GGGGGGTCG
MOUSE$SCC_06	640 (+)	1	87.50	SF-1	GGGAGGTCA
HT1$HTLV1_08	641 (-)	0	100.00	c-Ets-1 68	CCTCC
HS$HH4_05	642 (+)	1	88.89	HiNF-C	GAGGGCGGGG
MOUSE$M1H2LD_01	642 (+)	1	88.89	RAR-beta, RXR-beta	GAGGTCAGGG
HS$APOE_06	642 (-)	1	87.50		CCCCACCTC
AS$RAR_09	643 (+)	0	100.00	RAR-alpha1, RXR-alpha	AGGTCG
AS$ADR1_14	644 (+)	0	100.00	ADR1	GGTCGGGG
HSV1$GD_01	645 (-)	0	100.00	ICP4	CCGAC
AT$COR15A_01	645 (-)	0	100.00	ANT, CBF1, CBF2, CBF3	CCGAC
AT$RD29B_01	645 (-)	0	100.00	CBF1	CCGAC
AT$RD29A_01	645 (-)	0	100.00	CBF1, DREB1A, DREB2A	CCGAC
AT$COR78_01	645 (-)	0	100.00	ANT, CBF1, CBF2, CBF3	CCGAC
AT$COR15B_01	645 (-)	0	100.00	CBF1, CBF2, CBF3	CCGAC
HS$M1A11_01	646 (-)	1	87.50	ZFX	AGGCCCCGA
PA$PY_23	648 (+)	0	100.00	T-Ag	GGGGC
SV$SV40_04	648 (+)	0	100.00	T-Ag	GGGGC
SV$SV40_63	648 (+)	0	100.00	T-Ag	GGGGC
PA$PY_26	648 (-)	0	100.00	T-Ag	GCCCC
MAIZE$ADH11S_06	648 (-)	0	100.00	GCBP-1, Sp1	GCCCC
RAT$POMC_15	650 (-)	0	100.00	Otx1, Otx2, Pitx1, Pitx3	CTAAGCC
HS$P53_04	651 (-)	1	87.50	Egr-1	CGCCTACGC
MOUSE$E2F1_04	653 (-)	1	87.50	E2F-1	GGCGCGTAA
SV$SV40_63	654 (-)	0	100.00	T-Ag	GCCTA
RICE$RAB16A_05	655 (-)	1	90.00		CCGCCGCGCCT
RAT$IGF1R_09	658 (-)	1	87.50	WT1 -KTS, WT1 I -KTS	GCGGCGGCG
RAT$IGF1R_09	659 (+)	1	87.50	WT1 -KTS, WT1 I -KTS	GCGGCGGCG
CHICK$BLCR_02	659 (-)	0	100.00	NF-E4, Sp1	GCGGC
HS$CYCD1_14	660 (+)	0	100.00	Sp1, Sp2, Sp3, Sp4	CCGCG
HS$CDC2_09	660 (-)	0	100.00		CGCGG
HS$CYCA_05	660 (-)	0	100.00		CGCGG
HS$CDC2_09	661 (+)	0	100.00		CGCGG
HS$CYCA_05	661 (+)	0	100.00		CGCGG
MOUSE$E2F1_03	661 (+)	1	87.50	E2F-1	CGCGGCAAA
HS$CYCD1_14	661 (-)	0	100.00	Sp1, Sp2, Sp3, Sp4	CCGCG
CHICK$BLCR_02	662 (+)	0	100.00	NF-E4, Sp1	GCGGC
AS$ZIC2_28	664 (-)	1	87.50	Zic2	GGGTTTGCC
AS$SPZ1_19	667 (-)	1	87.50	Spz1	GGTGGGTTC
Y$CYB2_01	669 (+)	1	87.50	repressor of CAR1 expression	AACCGCCAA
Y$HSF1_01	669 (+)	1	87.50	repressor of CAR1 expression	AGCCGCCAA
HS$P21WAF1_06	670 (+)	0	100.00	SMAD-3, SMAD-4	CCCGCC
HS$P21WAF1_07	670 (+)	0	100.00		CCCGCC
HSV1$IE3_06	670 (-)	0	100.00	Sp1	GGCGGG
RAT$NF1_02	670 (-)	0	100.00	Sp1	GGCGGG
HS$NPY_02	670 (-)	0	100.00	Sp1	GGCGGG
HS$MIP_02	670 (-)	0	100.00	Sp1	GGCGGG
MOUSE$POLA2_02	670 (-)	1	87.50	E2F-1	TTTGGCGGG
MOUSE$BMYB_01	670 (-)	1	87.50	E2F, E2F-1, E2F-3, E2F-4	CTTGGCGGG
HS$CDC25C_04	671 (-)	0	100.00		GGCGG
HS$P53_04	672 (+)	1	87.50	Egr-1	CGCCTACGC
MOUSE$ADA_01	672 (-)	1	87.50	Egr-1	GCGTGGGCG
HBV$SA_01	673 (+)	0	100.00	NF-1	GCCAAC
MOUSE$AP2_07	673 (-)	0	100.00	NF-1	TTGGC
Y$CYC1_09	674 (-)	0	100.00	hap2, HAP2, HAP3	GTTGG
Y$CYC1_10	674 (-)	0	100.00	CP1A, HAP2	GTTGG
Y$CYC1_11	674 (-)	0	100.00	HAP3, NF-YA	GTTGG
PV$GRP18_02	675 (-)	1	87.50	VSF-1	GCTCCGTTG
DROME$ADH_14	679 (+)	0	100.00	Adf-1	GCAGC
DROME$ADH_06	679 (-)	0	100.00	Adf-1	GCTGC
HS$CMYC_16	679 (-)	0	100.00	CTCF	GCTGC
HS$CANP_01	681 (-)	1	88.89	GCF	TCCCGGCGCT
HS$ASCC_01	684 (-)	1	88.89	Sp1	GGGGCCGGGC
HS$CYCD1_15	686 (-)	0	100.00	Sp1, Sp2, Sp3, Sp4	GCCCG
HS$M1A11_01	687 (+)	1	87.50	ZFX	AGGCCCCGA
MOUSE$PAC1R_01	687 (+)	0	100.00	ZAC-1a	GGGCCC
MOUSE$PAC1R_01	687 (-)	0	100.00	ZAC-1a	GGGCCC
AS$STAT5A_51	688 (+)	1	87.50	STAT5A	TGCCCCGAA
HS$GG_36	688 (-)	0	100.00	Sp1	GGGGCC
PA$PY_26	689 (+)	0	100.00	T-Ag	GCCCC
MAIZE$ADH11S_06	689 (+)	0	100.00	GCBP-1, Sp1	GCCCC
PA$PY_23	689 (-)	0	100.00	T-Ag	GGGGC
SV$SV40_04	689 (-)	0	100.00	T-Ag	GGGGC
SV$SV40_63	689 (-)	0	100.00	T-Ag	GGGGC
Y$MEL1_01	692 (+)	0	100.00	GAL4	CCGAA
Y$MEL1_01	692 (-)	0	100.00	GAL4	TTCGG
HT1$HTLV1_14	697 (+)	0	100.00	AP-2alphaA, AP-2alphaB	CCTCAGGC
MOUSE$GFAP_03	697 (+)	0	100.00		CCTCAGGC
RAT$IGF1R_07	701 (+)	1	87.50	WT1 -KTS, WT1 I -KTS	AGCCCCCGC
HS$M1A11_01	701 (+)	1	87.50	ZFX	AGGCCCCGA
CHICK$BAC_06	702 (-)	1	88.89	GCF	GGGCGGGGCG
HS$GG_36	702 (-)	0	100.00	Sp1	GGGGCC
AS$SP1_17	702 (-)	1	88.89	Sp1	GGGCGGGGCG
HS$A11COL_01	703 (+)	0	100.00	Sp1	GCCCCGCCCC
PA$PY_26	703 (+)	0	100.00	T-Ag	GCCCC
HS$RAS1_02	703 (+)	1	88.89	Sp1	GCCCCGCCCG
HS$RAS1_05	703 (+)	1	88.89	Sp1	CCCCCGCCCC
HS$RAS1_06	703 (+)	0	100.00	Sp1	GCCCCGCCCC
HS$AAC_11	703 (+)	1	90.00	Sp1	GCCCCTCCCCA
HS$AAC_18	703 (+)	1	88.89	Sp1	GACCCGCCCC
MOUSE$TPA_01	703 (+)	1	87.50	Sp1	ACCCCGCCC
RAT$ME_02	703 (+)	0	100.00	Sp1	GCCCCGCCCC
MOUSE$RPS16_01	703 (+)	1	88.89	Sp1	GCTCCGCCCC
HS$ASCC_03	703 (+)	1	88.89	Sp1	GCCCTGCCCC
HS$CYCA_01	703 (+)	0	100.00		GCCCCGCCCC
MAIZE$ADH11S_06	703 (+)	0	100.00	GCBP-1, Sp1	GCCCC
MOUSE$DHFR_01	703 (-)	1	88.89	Sp1	GGGGCGGAGC
AD2$E1B_03	703 (-)	0	100.00	BTEB1, MAZ, Sp1	GGGGCGGGGC
HS$HSP70_10	703 (-)	1	88.89	Sp1	GGGGCGGCGC
PA$PY_23	703 (-)	0	100.00	T-Ag	GGGGC
HS$RAS1_03	703 (-)	0	100.00	Sp1	GGGGCGGGGC
HS$RAS1_04	703 (-)	0	100.00	Sp1	GGGGCGGGGC
SV$SV40_04	703 (-)	0	100.00	T-Ag	GGGGC
HSV1$TK_03	703 (-)	1	88.89	Sp1	GGGGCGGCGC
XENLA$U2SN_08	703 (-)	1	88.89	octamer-binding factor, Sp1	GAGGCGGGGC
XENLA$U2SN_09	703 (-)	1	88.89	octamer-binding factor, Sp1	GAGGCGGGGC
AD2$E1B_02	703 (-)	0	100.00	Sp1, TBP, TFIID	GGGGCGGGGC
HA$DHFR_04	703 (-)	1	88.89	Sp1	TGGGCGGGGC
HA$DHFR_05	703 (-)	1	88.89	Sp1	GAGGCGGGGC
AS$SP1_02	703 (-)	0	100.00	Sp1	GGGGCGGGGC
AS$SP1_13	703 (-)	1	88.89	Sp1	GGGGCAGGGC
HS$ASCC_01	703 (-)	1	88.89	Sp1	GGGGCCGGGC
HS$ASCC_02	703 (-)	1	88.89	Sp1	GGGGCGGGGT
MOUSE$ADA_02	703 (-)	1	88.89	Sp1	TGGGCGGGGC
HS$DHFR_05	703 (-)	0	100.00	Sp1	GGGGCGGGGC
HS$DHFR_06	703 (-)	0	100.00	Sp1	GGGGCGGGGC
RAT$IGFBP2_04	703 (-)	1	88.89	Sp1	GGGGCGGGGA
AS$SP1_36	703 (-)	1	88.89	Sp1	GGGGCAGGGC
SV$SV40_63	703 (-)	0	100.00	T-Ag	GGGGC
AS$ZIC2_09	703 (-)	1	87.50	Zic2	GGGGGGGGC
HS$KIT_01	703 (-)	1	88.89	Sp1	GGGGCGTGGC
HS$ZG_06	704 (+)	1	87.50	Sp1	CCCCTCCCC
Y$SUC2_01	704 (+)	0	100.00	MIG1	CCCCGC
AS$E2SP1_01	704 (+)	0	100.00	E2, Sp1	CCCCGCCC
Y$PDC1_02	704 (+)	0	100.00	MIG1	CCCCGC
AS$CP2_03	704 (+)	1	90.00	CP2	CCCAGCCCCAG
HS$TK_09	704 (+)	0	100.00	Sp1	CCCCGCC
HS$TERT_05	704 (+)	1	87.50	Sp1	CCCAGCCCC
HS$TERT_06	704 (+)	1	87.50	Sp1	CCCAGCCCC
HS$MT2A_10	704 (-)	0	100.00	Sp1	GGGGCGGGG
RAT$GSTP_09	704 (-)	0	100.00		GGGGCGGGG
MOUSE$PFK_05	704 (-)	0	100.00		GGGGCGGGG
HS$FN_07	704 (-)	0	100.00	Sp1	GGGGCGGGG

[illegible]	704 (-)	0	100.00	Sp1	[illegible]
AS$SP1_15	704 (-)	0	100.00	POU2F1, Sp1	GGGCGGGG
HS$MIP_03	704 (-)	1	87.50	Sp1	GGGGAGGGG
AS$ADR1_16	704 (-)	0	100.00	ADR1	GGGCGGGG
HS$TK_06	704 (-)	0	100.00	Sp1	GGCGGGG
HS$DHFR_08	704 (-)	1	87.50	Sp1	GGGGCGGGC
MOUSE$JUND_02	705 (+)	0	100.00	Sp1	CCCGCCCC
HS$P21WAF1_06	705 (+)	0	100.00	SMAD-3, SMAD-4	CCCGCC
HS$P21WAF1_07	705 (+)	0	100.00		CCCGCC
CHICK$BAG_02	705 (-)	0	100.00	Sp1	GGGGCGGG
HSV1$IE3_06	705 (-)	0	100.00	Sp1	GGCGGG
RAT$NF1_02	705 (-)	0	100.00	Sp1	GGCGGG
HS$NPY_02	705 (-)	0	100.00	Sp1	GGCGGG
HS$MIP_02	705 (-)	0	100.00	Sp1	GGCGGG
CHICK$ACRA_07	706 (+)	0	100.00	Sp1	CCGCCC
MOUSE$APRT_01	706 (+)	0	100.00	Sp1	CCGCCC
RAT$GF_07	706 (+)	0	100.00	Sp1	CCGCCC
HA$HMGCR_03	706 (+)	0	100.00		CCGCCC
HSV1$IE3_05	706 (+)	0	100.00	Sp1	CCGCCC
HSV1$IE4_04	706 (+)	0	100.00	Sp1	CCGCCC
MOUSE$EPOR_02	706 (+)	0	100.00	Sp1	CCGCCC
RAT$CDC2_02	706 (+)	0	100.00		CCGCCC
MOUSE$E2F1_01	706 (+)	0	100.00		CCGCCC
HS$TK_08	706 (+)	0	100.00	Sp1	CCGCCCC
HS$TERT_04	706 (+)	0	100.00	Sp1	CCGCCC
HS$TERT_07	706 (+)	0	100.00	Sp1	CCGCCC
HS$APOE_08	706 (-)	0	100.00	Sp1	GGGCGG
HA$HMGCR_03	706 (-)	0	100.00		GGGCGG
HSV1$IE3_04	706 (-)	0	100.00	Sp1	GGGCGG
HSV1$IE3_07	706 (-)	0	100.00	Sp1	GGGCGG
HSV1$IE3_09	706 (-)	0	100.00	Sp1	GGGCGG
HSV1$IE4_01	706 (-)	0	100.00	Sp1	GGGCGG
HSV1$IE4_03	706 (-)	0	100.00	Sp1	GGGCGG
SV$SV40_06	706 (-)	0	100.00	LSF	GGGCGG
SV$SV40_07	706 (-)	0	100.00	ETF, Sp1	GGGCGG
SV$SV40_13	706 (-)	0	100.00	Sp1	GGGCGG
HS$U2SN_03	706 (-)	0	100.00	Sp1	GGGCGG
HS$U2SN_05	706 (-)	0	100.00	Sp1	GGGCGG
PIG$UPA_10	706 (-)	0	100.00	Sp1	GGGCGG
PIG$UPA_11	706 (-)	0	100.00	Sp1	GGGCGG
PIG$UPA_14	706 (-)	0	100.00	Sp1	GGGCGG
MOUSE$GLUT4_05	706 (-)	0	100.00	Sp1	GGGCGG
AD5$E1A_15	706 (-)	0	100.00	Sp1	GGGCGG
HS$NPY_01	706 (-)	0	100.00	Sp1	GGGCGG
RAT$IGF2_01	706 (-)	0	100.00	Sp1	GGGCGG
HS$TK_01	706 (-)	0	100.00	CP1	GGGCGG
HS$CDC25C_04	706 (-)	0	100.00		GGCGG
HS$A24COL_03	706 (-)	0	100.00	Sp1	GGGCGG
HS$TK_07	706 (-)	0	100.00	Sp1	GGGCGG
RAT$HSC70_01	706 (-)	0	100.00	Sp1, Sp3	GGGGCGG
HS$CD11B_01	707 (+)	0	100.00	Sp1	CGCCC
Y$SUC2_02	707 (-)	1	87.50	MIG1	CCGGGGGCG
PA$PY_26	708 (+)	0	100.00	T-Ag	GCCCC
MAIZE$ADH11S_06	708 (+)	0	100.00	GCBP-1, Sp1	GCCCC
PA$PY_23	708 (-)	0	100.00	T-Ag	GGGGC
SV$SV40_04	708 (-)	0	100.00	T-Ag	GGGGC
SV$SV40_63	708 (-)	0	100.00	T-Ag	GGGGC
Y$GAL3_01	709 (+)	0	100.00	MIG1	CCCCAG
Y$GAL4_01	709 (+)	0	100.00	MIG1	CCCCAG
Y$HAP4_01	709 (+)	0	100.00	MIG1	CCCCAG
SV$SV40_37	710 (-)	0	100.00		CTGGG
HS$VEGF_02	712 (+)	0	100.00		CAGGT
AS$TR_07	715 (+)	0	100.00	T3R-alpha	GTTCC
AS$TR_10	715 (+)	0	100.00	T3R-alpha	GTTCC
HS$IL4_02	715 (-)	0	100.00		GGAAC
AS$VMYB_02	721 (-)	1	88.89	v-Myb	CCAAACTGCC
HS$PR264_08	723 (-)	0	100.00	c-Myb	AAACGG
AS$AREB6_42	725 (+)	0	100.00	AREB6	GTTTG
AS$AREB6_43	725 (+)	0	100.00	AREB6	GTTTG
AS$AREB6_45	725 (+)	0	100.00	AREB6	GTTTG
AS$AREB6_47	725 (+)	0	100.00	AREB6	GTTTG
AS$AREB6_51	725 (+)	0	100.00	AREB6	GTTTG
CHICK$LYS_24	726 (+)	1	87.50	AP-3, C/EBPalpha, TGGCA-binding protein	TTTGGCAAG
RAT$CYTBE_01	726 (-)	0	100.00		AGCCAAA
MOUSE$AP2_07	727 (+)	0	100.00	NF-1	TTGGC
AS$ZIC3_03	728 (+)	1	87.50	Zic3	TGGGTAGTT
AS$HFH2_28	730 (+)	1	90.91	FOXD3	GCTTGTTTGTTT
RICE$GLUB1_02	732 (-)	0	100.00	MYB5	AACAAACTA
AS$FOXJ2_01	733 (-)	1	88.89	FOXJ2 (long isoform)	CAAACAAACA
AS$FOXJ2_10	733 (-)	1	88.89	FOXJ2 (long isoform)	CAAACAAACA
MOUSE$HNF3B_02	734 (+)	1	87.50	HNF-3alpha, HNF-3B	GTTTGTTTT
AS$HFH2_27	734 (+)	1	90.91	FOXD3	GTTTGTTTGTTT
AS$AREB6_42	734 (+)	0	100.00	AREB6	GTTTG
AS$AREB6_43	734 (+)	0	100.00	AREB6	GTTTG
AS$AREB6_45	734 (+)	0	100.00	AREB6	GTTTG
AS$AREB6_47	734 (+)	0	100.00	AREB6	GTTTG
AS$AREB6_51	734 (+)	0	100.00	AREB6	GTTTG
RICE$GLUB1_03	734 (-)	0	100.00	MYB5	AACAAAC
AS$FOXJ1_05	735 (+)	0	100.00	FOXJ1	TTTGTTT
RAT$IGFBP1_01	735 (-)	0	100.00	HNF-3alpha, HNF-3B, HNF-3gamma	AAACAAA
AS$SRY_07	736 (-)	1	88.89	SRY	AGAGAAACAA
MOUSE$CDX2_01	737 (+)	0	100.00	FOXM1a, FOXM1b, HNF-3alpha, HNF-3B	TGTTT
RAT$FABPI_04	737 (+)	0	100.00	FOXM1a, FOXM1b, HNF-3alpha, HNF-3B	TGTTT
HS$ALP_02	737 (+)	0	100.00	HNF-3alpha, HNF-3B	TGTTT
RAT$KINK_02	737 (+)	1	88.89	HNF-3alpha, HNF-3B, HNF-3gamma	TGTTTGTTTT
MOUSE$HOXC8_03	737 (+)	0	100.00		TGTTTGT
MOUSE$ECADH_03	737 (-)	0	100.00	FOXM1a, FOXM1b, HNF-3alpha, HNF-3B	AAACA
MOUSE$HNF3B_02	738 (+)	1	87.50	HNF-3alpha, HNF-3B	GTTTGTTTT
AS$AREB6_42	738 (+)	0	100.00	AREB6	GTTTG
AS$AREB6_43	738 (+)	0	100.00	AREB6	GTTTG
AS$AREB6_45	738 (+)	0	100.00	AREB6	GTTTG
AS$AREB6_47	738 (+)	0	100.00	AREB6	GTTTG
AS$AREB6_51	738 (+)	0	100.00	AREB6	GTTTG
AS$TGIF_09	741 (+)	0	100.00	TGIF	TGTCT
RAT$MLCC_01	745 (+)	1	88.89	aMEF-2, MEF-2 (516 AA)	TTATTTTTAA
CHICK$MGF_02	745 (+)	0	100.00	Gbx2	TTAAT
AS$NKX61_02	745 (+)	0	100.00	Nkx6-1	TTAATTT
MOUSE$E1_01	745 (-)	1	88.89	POU2F1a, POU2F1b, POU2F1c, POU5F1	TTAAAATTCA
MOUSE$MCK_07	745 (-)	1	88.89	aMEF-2, MEF-2 (516 AA)	TTATAATTAA
RAT$AMHC_03	745 (-)	1	88.89	aMEF-2, MEF-2 (516 AA)	TTAAAAATAA
Y$MEL1_02	745 (-)	0	100.00	MIG1	ATTAA
DROME$TM1_01	745 (-)	1	88.89	D-MEF2	TTAAAAATAA
CHICK$MGF_01	745 (-)	0	100.00	Gbx2	ATTAA
Y$SUC2_02	746 (-)	0	100.00	MIG1	AATTA
RAT$DBH_01	746 (-)	0	100.00	ARIX, c-Fos, c-Jun, CREB, CREMtau	AATTA
Y$MAL61_03	748 (+)	0	100.00	MIG1	ATTTT
RAT$PL_15	748 (-)	0	100.00	F2F, POU1F1a	TAAAAT
Y$MAL2R_01	749 (+)	0	100.00	MIG1	TTTTA
AS$FOXJ1_04	749 (+)	0	100.00	FOXJ1	TTTTA
Y$MAL63_01	750 (+)	0	100.00	MIG1	TTTAA
CHICK$MGF_02	751 (+)	0	100.00	Gbx2	TTAAT
AS$NKX61_02	751 (+)	0	100.00	Nkx6-1	TTAATTT
Y$MEL1_02	751 (-)	0	100.00	MIG1	ATTAA
CHICK$MGF_01	751 (-)	0	100.00	Gbx2	ATTAA
Y$SUC2_02	752 (-)	0	100.00	MIG1	AATTA
RAT$DBH_01	752 (-)	0	100.00	ARIX, c-Fos, c-Jun, CREB, CREMtau	AATTA
RAT$SPI_01	754 (-)	1	87.50	C/EBPalpha	CTGAGAAAT
HS$TNFA_04	755 (-)	0	100.00		AGAAA
I$HSF_01	755 (-)	0	90.00	HSF	AGAAN
F$HSF_01	755 (-)	0	90.00	HSF	AGAAN
AS$STAT5A_62	756 (+)	1	87.50	STAT5A	TTCCCCGAG
AS$STAT5A_63	756 (+)	1	87.50	STAT5A	TTCACCGAG
Y$ADH2_01	757 (+)	0	100.00	ADR1	TCTCC
HS$LCK_01	757 (+)	1	87.50	LyF-1	TCTCCCAGG
AS$LYF1_07	757 (-)	1	87.50	LyF-1	CCTGGGAGA
AS$STAT5A_43	758 (+)	1	87.50	STAT5A	TTCCGAGGC
Y$ACS1_02	758 (+)	0	100.00	ADR1	CTCCGA
AS$STAT5A_46	759 (+)	1	87.50	STAT5A	TACGAGGCC
PA$PY_24	762 (+)	0	100.00	T-Ag	GAGGC
PA$PY_25	762 (+)	0	100.00	T-Ag	GAGGC
SV$SV40_02	762 (+)	0	100.00	T-Ag	GAGGC
SV$SV40_01	762 (-)	0	100.00	T-Ag	GCCTC
SV$SV40_02	762 (-)	0	100.00	T-Ag	GCCTC
SV$SV40_63	762 (-)	0	100.00	T-Ag	GCCTC
HSV1$TK_12	763 (-)	0	100.00	RAR-alpha1, T3R-alpha	TGGCCT
HS$GRH_01	764 (+)	0	100.00	AP-2, AP-2alphaA, AP-2alphaB, NF-1	GGCCA
AD$MLP_19	764 (+)	0	100.00		GGCCAGC
RAT$TAT_15	764 (+)	0	100.00	HNF-3alpha	GGCCA
HS$GRH_01	764 (-)	0	100.00	AP-2, AP-2alphaA, AP-2alphaB, NF-1	TGGCC
CHICK$STN1_01	766 (+)	0	100.00	LBP-1, MyoD	CCAGC
CHICK$STN1_03	766 (+)	0	100.00	LBP-1, NF-1 (-like proteins)	CCAGC
QUAIL$STN1_02	766 (+)	0	100.00		CCAGCC
HS$GP2B_09	766 (-)	0	100.00	HNF-1A, HNF-1B, HNF-1C	TGGCTGG
RAT$ARGL_03	768 (+)	0	100.00	NF-1	AGCCAG
RAT$POMC_03	771 (+)	0	100.00	GR	CAGAG
MOUSE$CRISP1_04	772 (+)	0	100.00	AR	AGAGCA
MOUSE$CRISP3_02	772 (+)	0	100.00	AR	AGAGCA
RAT$VEGF_02	773 (+)	0	100.00	ER-alpha, ER-beta	GAGCA
MOMLV$MOMULV_09	775 (-)	0	100.00	LVc	CCTGC
MOMLV$MOMULV_10	775 (-)	0	100.00	LVc	CCTGC
RADLV$RLV_03	775 (-)	0	100.00	LVc	CCTGC
HS$VEGF_02	776 (+)	0	100.00		CAGGT
AS$GAMYB_16	778 (-)	0	100.00	GAMYB	AACAAACC
AS$AREB6_42	779 (+)	0	100.00	AREB6	GTTTG
AS$AREB6_43	779 (+)	0	100.00	AREB6	GTTTG
AS$AREB6_45	779 (+)	0	100.00	AREB6	GTTTG
AS$AREB6_47	779 (+)	0	100.00	AREB6	GTTTG
AS$AREB6_51	779 (+)	0	100.00	AREB6	GTTTG
RICE$GLUB1_03	779 (-)	0	100.00	MYB5	AACAAAC
RAT$AC3_02	782 (+)	1	87.50		TGCTGGCAG
RAT$OCNC_02	782 (-)	1	87.50		CTGCCAGCA
Y$CYC1_09	783 (+)	0	100.00	hap2, HAP2, HAP3	GTTGG
Y$CYC1_10	783 (+)	0	100.00	CP1A, HAP2	GTTGG
Y$CYC1_11	783 (+)	0	100.00	HAP3, NF-YA	GTTGG
MOUSE$RPL32_04	783 (-)	1	87.50	delta factor, factor delta	GCTGCCATC
HBV$SA_01	783 (-)	0	100.00	NF-1	GCCAAC
HS$ALBU_02	784 (+)	0	100.00	NF-1/L	TTGGCA
MOUSE$AP2_07	784 (+)	0	100.00	NF-1	TTGGC
HS$ALBU_03	785 (+)	0	100.00	NF-1/L	TGGCA
HS$RBP_01	785 (+)	0	100.00	NF-1/L	TGGCA
RAT$A1I3_02	785 (+)	0	100.00	NF-1	TGGCA
RAT$AFEP_11	785 (+)	0	100.00	GR, NF-1	TGGCA
MMTV$MMTV_43	785 (+)	0	100.00	NF-1	TGGCA
AS$MEIS1AHOXA9_11	785 (+)	0	100.00	HOXA9, Meis-1a	TGGCAG
DROME$ADH_14	787 (+)	0	100.00	Adf-1	GCAGC
DROME$ADH_06	787 (-)	0	100.00	Adf-1	GCTGC
HS$CMYC_16	787 (-)	0	100.00	CTCF	GCTGC
MOUSE$M2EAK_03	788 (+)	0	100.00	NF-Y	CAGCA
HS$GHA_05	792 (+)	0	100.00	GR, GR-alpha, GR-beta	AGTACC
Y$GAL1_15	794 (-)	0	100.00	MIG1	GGGGTA
AS$SPZ1_17	794 (-)	1	87.50	Spz1	GGAGGGATA
AS$ZIC_07	794 (-)	1	87.50	Zic1	GGGGGGGTA
AS$ZIC_17	794 (-)	1	87.50	Zic1	GGGGGGGTA
AS$ZIC2_21	794 (-)	1	87.50	Zic2	GGGGGGGTA
AS$ZIC3_21	794 (-)	1	87.50	Zic3	GGGGGGGTA
HS$NPY_04	796 (+)	0	100.00	Sp1	CCCCTCC
AS$ADR1_01	796 (-)	0	100.00	ADR1	TGGAGGGG
AS$VDR_04	796 (-)	0	100.00	VDR	AGGGG
Y$GLK1_02	796 (-)	0	100.00	MSN2, MSN4	AGGGG
CTCF$CONS	797 (+)	0	100.00	CTCF	CCCTC
HT1$HTLV1_08	798 (+)	0	100.00	c-Ets-1 68	CCTCC
CHICK$STN1_01	801 (+)	0	100.00	LBP-1, MyoD	CCAGC
CHICK$STN1_03	801 (+)	0	100.00	LBP-1, NF-1 (-like proteins)	CCAGC
MOUSE$M2EAK_03	802 (+)	0	100.00	NF-Y	CAGCA
Y$ADE4_03	803 (-)	1	87.50	GCN4	ATGACTGCT
HS$NPY_03	804 (-)	0	100.00	AP-1, CCAAT-binding factor	TGACTGC
Y$HIS4_13	805 (+)	0	100.00	GCN4	CAGTCA
FY$RPL82_01	805 (+)	0	100.00		CAGTCACG
FY$RPL32_01	805 (+)	0	100.00		CAGTCACG
NT$CHN50_01	806 (+)	0	100.00	WRKY1, WRKY3, WRKY4	AGTCA
HS$MMP1_06	807 (+)	0	100.00		GTCAC
DROME$AC_02	807 (+)	1	88.89	H	GGCACGCGAC
HA$HMGCR_04	807 (-)	0	100.00	USF	CGTGAC
HS$P53_03	807 (-)	0	100.00	Pax-2, Pax-5, Pax-8	GTGAC
RAT$CYTOP_04	809 (+)	0	100.00	AhR, Arnt	CACGC
AD$MLP_10	809 (+)	1	87.50	USF	CACGTGACC
DAUCE$DC3_04	809 (+)	0	100.00	DPBF-1, DPBF-2	CACGCG
AS$AHRARNT_50	809 (-)	0	100.00	AhR, Arnt	GCGTG
Y$CDC9_02	810 (+)	0	100.00	DSC1	ACGCGA
Y$CDC6_01	810 (+)	0	100.00	MCBF	ACGCGA
AS$ZIC_03	814 (-)	1	87.50	Zic1	TGGCTTGTC
CHICK$STN1_01	816 (+)	0	100.00	LBP-1, MyoD	CCAGC
CHICK$STN1_03	816 (+)	0	100.00	LBP-1, NF-1 (-like proteins)	CCAGC
QUAIL$STN1_02	816 (+)	0	100.00		CCAGCC
HS$GP2B_09	816 (-)	0	100.00	HNF-1A, HNF-1B, HNF-1C	TGGCTGG
HS$AG_01	818 (+)	0	100.00	CTF, CTF-1, CTF-2, CTF-3, EFI	AGCCAAT
HS$BG_02	818 (+)	0	100.00	CTF	AGCCAAT
ASPN$AMDS_01	818 (+)	0	100.00	AmdR, AnCF	AGCCAAT
ASPN$AMDS_02	818 (+)	0	100.00	AnCF	AGCCAAT
HSV1$TK_02	818 (-)	0	100.00	CP1, CTF, NF-1	ATTGGCT
HS$DPOLA_03	818 (-)	0	100.00	CP1, CTF	ATTGGCT
MOUSE$AAG_05	819 (+)	0	100.00	alpha-CP1, CCAAT-binding factor	GCCAAT
AD2$ORI_01	819 (+)	0	100.00	CTF-1, DBP, NF-1	GCCAAT
RAT$INS1_14	819 (+)	1	88.89	CCAAT-binding factor	GCCAATCTGC
MOUSE$JUND_04	819 (+)	0	100.00		GCCAAT
MOUSE$AP2_07	819 (-)	0	100.00	NF-1	TTGGC
HS$BAC_03	820 (+)	0	100.00	SRF	CCAAT
AMULV$AMULV_01	820 (+)	0	100.00		CCAAT
EBV$DSL_06	820 (+)	0	100.00		CCAAT
MOUSE$AAG_04	820 (+)	0	100.00	alpha-CP1, alpha-CP2a, alpha-CP2b, alpha-IRP	CCAAT
CHICK$BAG_06	820 (+)	0	100.00	TGGCA-binding protein	CCAAT
HS$GG_15	820 (+)	0	100.00	NF-E	CCAAT
HS$GG_16	820 (+)	0	100.00	CDP2, Clox, CUTL1, Cutl1	CCAAT
HS$GG_17	820 (+)	0	100.00	gammaCAAT	CCAAT
HS$GG_18	820 (+)	0	100.00	CP1	CCAAT
HS$ZG_07	820 (+)	0	100.00	CP2	CCAAT
HS$ZG_08	820 (+)	0	100.00	CP1	CCAAT
HS$HH1_03	820 (+)	0	100.00	H1TF2	CCAAT
MOUSE$M2IAB_01	820 (+)	0	100.00	PU.1	CCAAT
PIG$UPA_12	820 (+)	0	100.00	NF-1	CCAAT
RADLV$RLV_10	820 (+)	0	100.00		CCAAT
MULV$MULV_03	820 (+)	0	100.00		CCAAT
MOUSE$GLUT4_04	820 (+)	0	100.00		CCAAT
MOMLV$MOMULV_15	820 (+)	0	100.00		CCAAT
RAT$ALDB_02	820 (+)	0	100.00	CBF (2), CP2	CCAAT
HS$GG_20	820 (+)	0	100.00	CP1, NF-E3	CCAAT
RAT$TH2B_01	820 (+)	0	100.00		CCAAT
RAT$TH2B_02	820 (+)	0	100.00		CCAAT
HS$GG_21	820 (+)	0	100.00	CP1	CCAAT
MOUSE$LY6E_01	820 (+)	0	100.00		CCAAT
RAT$NEU_01	820 (+)	0	100.00		CCAAT
HS$GHA_10	820 (+)	0	100.00	alpha-CBF	CCAAT
NEUCR$CPC1_02	820 (+)	0	100.00		CCAAT
HS$EG_07	820 (+)	0	100.00	CP1	CCAAT
HS$GP2B_13	820 (+)	0	100.00		CCAAT
MOUSE$BMG_10	820 (+)	0	100.00		CCAAT
HS$CYBH_01	820 (+)	0	100.00	CDP2, Clox, CP1, CUTL1, Cutl1	CCAAT
HBV$S_04	820 (+)	0	100.00		CCAAT
HS$FN_06	820 (+)	0	100.00		CCAAT
MOUSE$E2F1_02	820 (+)	0	100.00		CCAAT
HS$A24COL_01	820 (+)	0	100.00		CCAAT
MOUSE$EKLF_01	820 (+)	0	100.00	AP-1, GATA-1	CCAAT
MOUSE$A11COL_01	820 (-)	0	100.00	CBF (2), IF2	ATTGG
MOUSE$A21COL_03	820 (-)	0	100.00	CCAAT-binding factor	ATTGG
MOUSE$A21COL_04	820 (-)	0	100.00	CBF (2), CBF-A, CBF-B	ATTGG
MOUSE$A21COL_05	820 (-)	0	100.00	EFI	ATTGG
AD2$E2L_03	820 (-)	0	100.00	CRF	ATTGG
HS$GHA_06	820 (-)	0	100.00	NF-1	ATTGG
HS$HH1_02	820 (-)	0	100.00	H1TF1, HiNF-B	GATTGG
SP$H2B1_02	820 (-)	0	100.00	CBF (1)	ATTGG
SP$H2B1_03	820 (-)	0	100.00	CDF	ATTGG
HS$HSP70_02	820 (-)	0	100.00	CTF	ATTGG
HS$HSP70_06	820 (-)	0	100.00	CCAAT-binding factor	GATTGG
HS$HSP70_07	820 (-)	0	100.00	CBTF, CP1, CTF	ATTGG
HS$HSP70_09	820 (-)	0	100.00	CP1, CTF	GATTGG
MOUSE$M2EAK_07	820 (-)	0	100.00	NF-Y	ATTGG
MOUSE$M2EAK_08	820 (-)	0	100.00	NF-Y	ATTGG
RSV$RSV_05	820 (-)	0	100.00		ATTGG
HSV1$TK_04	820 (-)	0	100.00	C/EBPalpha	ATTGG
HSV1$TK_05	820 (-)	0	100.00	NF-Y'	ATTGG
AS$HAP23_01	820 (-)	0	100.00	HAP2, HAP3, HAP4	ATTGG
MOUSE$M1H2KB_12	820 (-)	0	100.00	EFI	ATTGG
RAT$TH2A_02	820 (-)	0	100.00		ATTGG
RAT$TH2A_04	820 (-)	0	100.00		ATTGG
HS$TK_02	820 (-)	0	100.00	CP1	ATTGG
MOUSE$A11COL_07	820 (-)	0	100.00	NF-1, Sp1	ATTGG
HS$CDC2_07	820 (-)	0	100.00	CBAF, CBF-B, CP1A	ATTGG
XENLA$GATA2_01	820 (-)	0	100.00	CBTF	ATTGG
RAT$NAT_05	820 (-)	0	100.00		ATTGG
HS$CDC2_12	820 (-)	0	100.00	CBAF	ATTGG
HS$GG_26	821 (-)	0	100.00	GATA-1	AGATTG
MOUSE$BMG_04	822 (+)	0	100.00	GATA-1	AATCT
AS$ZIC3_14	822 (-)	1	87.50	Zic3	CGGGAGGTT
Y$ADH2_01	824 (+)	0	100.00	ADR1	TCTCC
AS$LYF1_05	824 (-)	0	100.00	LyF-1	GGGAGA
CHICK$ITGB3_01	824 (-)	0	100.00	RXR-beta, VDR	GGGAGA
Y$SUC2_02	828 (+)	1	87.50	MIG1	CCGGGGGCG
RAT$IGF1R_09	828 (+)	1	87.50	WT1 -KTS, WT1 I -KTS	GCGGCGGCG
Y$ILV2_02	828 (-)	1	87.50	repressor of CAR1 expression	AGCCGCCGG
MOUSE$CEBPA_02	829 (-)	0	100.00	CUP	GCCGCCG
HS$CDC25C_04	830 (+)	0	100.00		GGCGG
AS$GCCBOX_01	830 (-)	0	100.00	ERF2, ERF3, ERF4	GCCGCC
AS$GCCBOX_02	830 (-)	0	100.00	Pti4	GCCGCC
CHICK$BLCR_02	831 (+)	0	100.00	NF-E4, Sp1	GCGGC
RAT$MT1_04	833 (+)	1	87.50		TGCGCTCGG
DROME$EVE_04	835 (+)	0	100.00	GAGA factor	CGCTC
DROME$EVE_08	835 (+)	0	100.00	GAGA factor	CGCTC
DROME$EVE_10	835 (+)	0	100.00	GAGA factor	CGCTC
AS$STAT5A_62	837 (-)	1	87.50	STAT5A	TTCCCCGAG
AS$LYF1_06	838 (+)	1	87.50	LyF-1	TCTGGGAGG
MOUSE$IGLL_03	838 (-)	1	87.50	LyF-1	CCTCCCAGA
AS$ZIC_08	839 (+)	1	87.50	Zic1	CGGGGAGTC
HS$TPI_01	840 (+)	1	90.00	Sp1	GGGGCGGCGGC
HS$PFKM_03	840 (+)	1	88.89		GGGGAGGAGG
HS$MIP_03	840 (+)	1	87.50	Sp1	GGGGAGGGG
HS$ZG_06	840 (-)	1	87.50	Sp1	CCCCTCCCC
HS$AAC_13	840 (-)	1	90.00	Sp1	GCCCCCTCCCC
HT1$HTLV1_08	842 (-)	0	100.00	c-Ets-1 68	CCTCC
HS$HSP70_10	843 (+)	1	88.89	Sp1	GGGGCGGCGC
PA$PY_24	843 (+)	0	100.00	T-Ag	GAGGC
PA$PY_25	843 (+)	0	100.00	T-Ag	GAGGC
SV$SV40_02	843 (+)	0	100.00	T-Ag	GAGGC
HSV1$TK_03	843 (+)	1	88.89	Sp1	GGGGCGGCGC
XENLA$U2SN_08	843 (+)	1	88.89	octamer-binding factor, Sp1	GAGGCGGGGC
XENLA$U2SN_09	843 (+)	1	88.89	octamer-binding factor, Sp1	GAGGCGGGGC
HA$DHFR_05	843 (+)	1	88.89	Sp1	GAGGCGGGGC
HS$CDC25C_02	843 (+)	0	100.00		GAGGCG
RAT$IGF1R_09	843 (+)	1	87.50	WT1 -KTS, WT1 I -KTS	GCGGCGGCG
HS$RAS1_01	843 (-)	1	88.89	Sp1	GCTCCGCCTC
SV$SV40_01	843 (-)	0	100.00	T-Ag	GCCTC
SV$SV40_02	843 (-)	0	100.00	T-Ag	GCCTC
SV$SV40_63	843 (-)	0	100.00	T-Ag	GCCTC
HS$TERT_03	843 (-)	1	87.50	Sp1	CTCCGCCTC
HS$TPI_04	844 (+)	0	100.00	Sp1	AGGCGG
HS$CDC25C_04	845 (+)	0	100.00		GGCGG
AS$GCCBOX_01	845 (-)	0	100.00	ERF2, ERF3, ERF4	GCCGCC
AS$GCCBOX_02	845 (-)	0	100.00	Pti4	GCCGCC
CHICK$BLCR_02	846 (+)	0	100.00	NF-E4, Sp1	GCGGC
HSV1$TK_08	848 (+)	0	100.00	ZF5	GGCGCG
RAT$MT1_04	850 (+)	1	87.50		TGCGCTCGG
DROME$EVE_04	852 (+)	0	100.00	GAGA factor	CGCTC
DROME$EVE_08	852 (+)	0	100.00	GAGA factor	CGCTC
DROME$EVE_10	852 (+)	0	100.00	GAGA factor	CGCTC
AS$NCX_15	857 (+)	1	88.89	Ncx	AGGAACGAGG
HS$IL4_02	858 (+)	0	100.00		GGAAC
AS$TR_07	858 (-)	0	100.00	T3R-alpha	GTTCC
AS$TR_10	858 (-)	0	100.00	T3R-alpha	GTTCC
V$MZF1_01	862 (+)	0	87.50	MZF1	NGNGGGGA
AS$SP1_04	863 (+)	1	88.89	Sp1	GAGGGGTGGT
MZF1$CONS_01	863 (+)	0	92.86	MZF-1	GNGGGGA
AS$SP1_27	863 (+)	1	88.89	Sp1	GAGGGGTGGT
CTCF$CONS	863 (-)	0	100.00	CTCF	CCCTC
HS$GPC_06	863 (-)	1	90.00	Sp1	CCCCTCCCCTC
AS$VDR_04	864 (+)	0	100.00	VDR	AGGGG
Y$GLK1_02	864 (+)	0	100.00	MSN2, MSN4	AGGGG
MOUSE$AQP7_01	864 (+)	0	100.00	PPAR-alpha, PPAR-gamma, RXR-alpha	AGGGGA
HS$PFKM_03	865 (+)	1	88.89		GGGGAGGAGG
HS$MIP_03	865 (+)	1	87.50	Sp1	GGGGAGGGG
AS$ZIC3_17	865 (+)	1	87.50	Zic3	GGGGGGGTG
HS$ZG_06	865 (-)	1	87.50	Sp1	CACCACCCC
HS$ZG_06	865 (-)	1	87.50	Sp1	CCCCTCCCC
HS$AAC_13	865 (-)	1	90.00	Sp1	GCCCCCTCCCC
MOUSE$IGKL_05	866 (+)	1	88.89	E12, E47, MyoD	GGCAGGTGGC
HT1$HTLV1_08	867 (-)	0	100.00	c-Ets-1 68	CCTCC
PV$DLEC2_02	867 (-)	1	87.50	ROM1, ROM2	GCCACCTCA
PV$DLEC2_03	867 (-)	1	87.50	ROM1, ROM2	GCCACCTCA
PV$PHSB_01	867 (-)	1	87.50	ROM1, ROM2	GCCACCTCA
HS$APOE_06	868 (-)	1	87.50		CCCCACCTC
Y$ENO1_03	868 (-)	1	87.50	repressor of CAR1 expression	AGCCACCTC
BPV1$BPV1_24	870 (+)	0	100.00	Sp1	GGTGG
HS$BG_48	870 (+)	0	100.00	CAC-binding protein	GGTGG
MOUSE$CEBPA_06	870 (+)	0	100.00	Sp1	GGTGG
HT1$HTLV1_08	870 (-)	0	100.00	c-Ets-1 68	CCACC
HS$BG_52	870 (-)	0	100.00		CCACC
HS$BG_14	870 (-)	0	100.00		CCACC
HS$BG_44	870 (-)	0	100.00	CAC-binding protein	CCACC
AS$ZIC3_32	872 (+)	1	87.50	Zic3	TGGCGGTAC
HS$CDC25C_04	873 (+)	0	100.00		GGCGG
Y$CHA1_01	874 (+)	0	100.00	CHA4	GCGGA
Y$CHA1_01	874 (-)	0	100.00	CHA4	TCCGC
E74A$CONS_01	875 (+)	0	90.00	E74A	MGGAA
Y$BAP2_01	875 (+)	1	87.50	LEU3	CGGAACCGG
HS$IL4_02	876 (+)	0	100.00		GGAAC
AS$TR_07	876 (-)	0	100.00	T3R-alpha	GTTCC
AS$TR_10	876 (-)	0	100.00	T3R-alpha	GTTCC
AS$ZIC2_29	878 (-)	1	87.50	Zic2	GGCGAGGTT
HS$CYCD1_14	880 (+)	0	100.00	Sp1, Sp2, Sp3, Sp4	CCGCG
HS$CDC2_09	880 (-)	0	100.00		CGCGG
HS$CYCA_05	880 (-)	0	100.00		CGCGG
HSV1$TK_08	881 (-)	0	100.00	ZF5	GGCGCG
CHICK$ACC_02	884 (+)	1	87.50		GCCGGGGGC
MOUSE$JUND_07	884 (+)	1	87.50		GCGGGGGCC
RAT$IGF1R_03	884 (+)	1	87.50	WT1 -KTS, WT1 I -KTS	GCGGGGGCC
RAT$IGF1R_08	884 (+)	1	87.50	WT1 -KTS, WT1 I -KTS	GCGGGGGCC
MAIZE$ADH1P_02	885 (-)	0	100.00		CCCCGG
Y$MAL2R_01	885 (-)	0	100.00	MIG1	CCCCGG
Y$MAL63_01	885 (-)	0	100.00	MIG1	CCCCGG
PA$PY_23	887 (+)	0	100.00	T-Ag	GGGGC
SV$SV40_04	887 (+)	0	100.00	T-Ag	GGGGC
HS$GG_36	887 (+)	0	100.00	Sp1	GGGGCC
SV$SV40_63	887 (+)	0	100.00	T-Ag	GGGGC
AS$CF1_19	887 (+)	1	87.50	CF1	GGGGTCACC
AS$CF1_23	887 (+)	1	87.50	CF1	GGGGTCACC
AS$CF1_25	887 (+)	1	87.50	CF1	GGGGTCACC
AS$CF1_27	887 (+)	1	87.50	CF1	GGGGTCACC
PA$PY_26	887 (-)	0	100.00	T-Ag	GCCCC
MAIZE$ADH11S_06	887 (-)	0	100.00	GCBP-1, Sp1	GCCCC
HS$GRH_01	889 (+)	0	100.00	AP-2, AP-2alphaA, AP-2alphaB, NF-1	GGCCA
RAT$TAT_15	889 (+)	0	100.00	HNF-3alpha	GGCCA
HS$GRH_01	889 (-)	0	100.00	AP-2, AP-2alphaA, AP-2alphaB, NF-1	TGGCC
PV$DLEC2_02	890 (+)	1	87.50	ROM1, ROM2	GCCACCTCA
PV$DLEC2_03	890 (+)	1	87.50	ROM1, ROM2	GCCACCTCA
PV$PHSB_01	890 (+)	1	87.50	ROM1, ROM2	GCCACCTCA
HT1$HTLV1_08	891 (+)	0	100.00	c-Ets-1 68	CCACC
HS$BG_52	891 (+)	0	100.00		CCACC
HS$BG_14	891 (+)	0	100.00		CCACC
HS$BG_44	891 (+)	0	100.00	CAC-binding protein	CCACC
BPV1$BPV1_24	891 (-)	0	100.00	Sp1	GGTGG
HS$BG_48	891 (-)	0	100.00	CAC-binding protein	GGTGG
MOUSE$CEBPA_06	891 (-)	0	100.00	Sp1	GGTGG
DELTAEF1$CONS_01	892 (+)	0	92.86	deltaEF1	CACCTNA
AS$PAX2_62	895 (+)	1	87.50	Pax-2.1, Pax-2.2	CTTAAAGCC
AS$RAR_14	899 (+)	0	100.00	RAR-alpha1, RXR-alpha	AGGCCG
HS$EGFR_22	900 (-)	1	88.89	GCF	CAGCGCGGCC
HS$CAVEOLIN1_03	900 (-)	0	100.00	Sp1	GCGGCC
CHICK$BLCR_02	901 (-)	0	100.00	NF-E4, Sp1	GCGGC
HS$CYCD1_14	902 (+)	0	100.00	Sp1, Sp2, Sp3, Sp4	CCGCG
HS$CDC2_09	902 (-)	0	100.00		CGCGG
HS$CYCA_05	902 (-)	0	100.00		CGCGG
DROME$EVE_04	905 (+)	0	100.00	GAGA factor	CGCTC
DROME$EVE_08	905 (+)	0	100.00	GAGA factor	CGCTC
DROME$EVE_10	905 (+)	0	100.00	GAGA factor	CGCTC
DROME$EVE_10	907 (+)	0	100.00	GAGA factor	CTCGC
RAT$ME_05	907 (+)	1	88.89		CTCGCCACCC
FACT1$CONS	908 (-)	0	100.00	F-ACT1	TGGCGA
RAT$OMP_06	910 (-)	1	87.50	Sp1	GAGGCGGGC
CHICK$STN1_01	911 (+)	0	100.00	LBP-1, MyoD	CCAGC
CHICK$STN1_03	911 (+)	0	100.00	LBP-1, NF-1 (-like proteins)	CCAGC
QUAIL$STN1_02	911 (+)	0	100.00		CCAGCC
MOUSE$MYOD_04	912 (-)	0	100.00	ARP-1, RXR-alpha, RXR-gamma, T3R-alpha	AGGCTG
Y$GAL1_10	913 (+)	0	100.00	GAL4	AGCCT
SV$SV40_01	914 (+)	0	100.00	T-Ag	GCCTC
SV$SV40_02	914 (+)	0	100.00	T-Ag	GCCTC
SV$SV40_63	914 (+)	0	100.00	T-Ag	GCCTC
PA$PY_24	914 (-)	0	100.00	T-Ag	GAGGC
PA$PY_25	914 (-)	0	100.00	T-Ag	GAGGC
SV$SV40_02	914 (-)	0	100.00	T-Ag	GAGGC
MOUSE$BMYB_01	916 (+)	1	87.50	E2F, E2F-1, E2F-3, E2F-4	CTTGGCGGG
HA$DHFR_04	917 (+)	1	88.89	Sp1	TGGGCGGGGC
MOUSE$ADA_02	917 (+)	1	88.89	Sp1	TGGGCGGGGC
CHICK$BAC_06	918 (+)	1	88.89	GCF	GGGCGGGGCG
AS$SP1_17	918 (+)	1	88.89	Sp1	GGGCGGGGCG
HSV1$IE3_06	919 (+)	0	100.00	Sp1	GGCGGG
RAT$NF1_02	919 (+)	0	100.00	Sp1	GGCGGG
HS$NPY_02	919 (+)	0	100.00	Sp1	GGCGGG
HS$GAST_02	919 (+)	1	88.89		GGCGGGGTGG
HS$CDC25C_04	919 (+)	0	100.00		GGCGG
HS$MIP_02	919 (+)	0	100.00	Sp1	GGCGGG
HS$TK_06	919 (+)	0	100.00	Sp1	GGCGGGG
HS$TK_09	919 (-)	0	100.00	Sp1	CCCCGCC
HS$P21WAF1_06	919 (-)	0	100.00	SMAD-3, SMAD-4	CCCGCC
HS$P21WAF1_07	919 (-)	0	100.00		CCCGCC
Y$SUC2_01	920 (-)	0	100.00	MIG1	CCCCGC
Y$PDC1_02	920 (-)	0	100.00	MIG1	CCCCGC
HS$HSP70_10	922 (+)	1	88.89	Sp1	GGGGCGGCGC
PA$PY_23	922 (+)	0	100.00	T-Ag	GGGGC
SV$SV40_04	922 (+)	0	100.00	T-Ag	GGGGC
HSV1$TK_03	922 (+)	1	88.89	Sp1	GGGGCGGCGC
HS$HSP70_12	922 (+)	1	88.89	Sp1	GGGGCGGCAC
SV$SV40_63	922 (+)	0	100.00	T-Ag	GGGGC
RAT$HSC70_01	922 (+)	0	100.00	Sp1, Sp3	GGGGCGG
Y$HOP1_01	922 (+)	1	87.50		TGGGCGGCT
PA$PY_26	922 (-)	0	100.00	T-Ag	GCCCC
HS$AAC_18	922 (-)	1	88.89	Sp1	GACCCGCCCC
HS$TK_08	922 (-)	0	100.00	Sp1	CCGCCCC
MAIZE$ADH11S_06	922 (-)	0	100.00	GCBP-1, Sp1	GCCCC
HS$APOE_08	923 (+)	0	100.00	Sp1	GGGCGG
HA$HMGCR_03	923 (+)	0	100.00		GGGCGG
HSV1$IE3_04	923 (+)	0	100.00	Sp1	GGGCGG
HSV1$IE3_07	923 (+)	0	100.00	Sp1	GGGCGG
HSV1$IE3_09	923 (+)	0	100.00	Sp1	GGGCGG
HSV1$IE4_01	923 (+)	0	100.00	Sp1	GGGCGG
HSV1$IE4_03	923 (+)	0	100.00	Sp1	GGGCGG
SV$SV40_06	923 (+)	0	100.00	LSF	GGGCGG
SV$SV40_07	923 (+)	0	100.00	ETF, Sp1	GGGCGG
SV$SV40_13	923 (+)	0	100.00	Sp1	GGGCGG
HS$U2SN_03	923 (+)	0	100.00	Sp1	GGGCGG
HS$U2SN_05	923 (+)	0	100.00	Sp1	GGGCGG
PIG$UPA_10	923 (+)	0	100.00	Sp1	GGGCGG
PIG$UPA_11	923 (+)	0	100.00	Sp1	GGGCGG
PIG$UPA_14	923 (+)	0	100.00	Sp1	GGGCGG
MOUSE$GLUT4_05	923 (+)	0	100.00	Sp1	GGGCGG
AD5$E1A_15	923 (+)	0	100.00	Sp1	GGGCGG
HS$NPY_01	923 (+)	0	100.00	Sp1	GGGCGG
RAT$IGF2_01	923 (+)	0	100.00	Sp1	GGGCGG
HS$TK_01	923 (+)	0	100.00	CP1	GGGCGG
HS$A24COL_03	923 (+)	0	100.00	Sp1	GGGCGG
HS$TK_07	923 (+)	0	100.00	Sp1	GGGCGG
CHICK$ACRA_07	923 (-)	0	100.00	Sp1	CCGCCC
MOUSE$APRT_01	923 (-)	0	100.00	Sp1	CCGCCC
RAT$GF_07	923 (-)	0	100.00	Sp1	CCGCCC
HA$HMGCR_03	923 (-)	0	100.00		CCGCCC
HSV1$IE3_05	923 (-)	0	100.00	Sp1	CCGCCC
HSV1$IE4_04	923 (-)	0	100.00	Sp1	CCGCCC
MOUSE$EPOR_02	923 (-)	0	100.00	Sp1	CCGCCC
RAT$CDC2_02	923 (-)	0	100.00		CCGCCC
MOUSE$E2F1_01	923 (-)	0	100.00		CCGCCC
HS$CD11B_01	923 (-)	0	100.00	Sp1	CGCCC
HS$TERT_04	923 (-)	0	100.00	Sp1	CCGCCC
HS$TERT_07	923 (-)	0	100.00	Sp1	CCGCCC
HS$CDC25C_04	924 (+)	0	100.00		GGCGG
NT$PRB1B_01	924 (+)	0	100.00	EBP	GGCGGCT
AS$GCCBOX_01	924 (-)	0	100.00	ERF2, ERF3, ERF4	GCCGCC
AS$GCCBOX_02	924 (-)	0	100.00	Pti4	GCCGCC
CHICK$BLCR_02	925 (+)	0	100.00	NF-E4, Sp1	GCGGC
MOUSE$JUND_07	927 (-)	1	87.50		GCGGGGGCC
RAT$IGF1R_03	927 (-)	1	87.50	WT1 -KTS, WT1 I -KTS	GCGGGGGCC
RAT$IGF1R_08	927 (-)	1	87.50	WT1 -KTS, WT1 I -KTS	GCGGGGGCC
HS$GMCSF_10	927 (-)	0	100.00	NF-ATp	GGAGCC
RAT$MLC_04	928 (-)	0	100.00	MAPF2, YY1	GGAGC
HS$P21WAF1_06	931 (+)	0	100.00	SMAD-3, SMAD-4	CCCGCC
HS$P21WAF1_07	931 (+)	0	100.00		CCCGCC
HSV1$IE3_06	931 (-)	0	100.00	Sp1	GGCGGG
RAT$NF1_02	931 (-)	0	100.00	Sp1	GGCGGG
HS$NPY_02	931 (-)	0	100.00	Sp1	GGCGGG
HS$MIP_02	931 (-)	0	100.00	Sp1	GGCGGG
EGR2$CONS	932 (+)	1	88.89	EGR2	CCGCCCCCGC
HS$CDC25C_04	932 (-)	0	100.00		GGCGG
WTZFP$CONS	933 (+)	1	87.50	WT1, WT1 -KTS, WT1 I, WT1 I -KTS, WT1 I-del2, WT1-del2	CGCCCCCGC
HS$IGF2_01	933 (+)	1	87.50	WT1	CGCCCCCGC
HS$IGF2_03	933 (+)	1	87.50	WT1	CGCCCCCGC
MOUSE$HOX14_01	933 (-)	1	87.50	Krox-20, Krox-24	GCGGGGGCG
HS$IGF2_02	933 (-)	1	87.50	WT1	GCGGGGGCG
AS$NGFIC_01	933 (-)	1	87.50	NGFI-C	GCGGGGGCG
MOUSE$JUND_05	933 (-)	1	87.50	Sp1	GCGGGGGCG
RAT$IGF1R_04	933 (-)	1	87.50	WT1, WT1 -KTS, WT1 I, WT1 I -KTS	GCGGGGGCG
RAT$IGF1R_09	933 (-)	0	100.00	WT1 -KTS, WT1 I -KTS	GCGGCGGCG
MOUSE$CEBPA_02	934 (+)	0	100.00	CUP	GCCGCCG
AS$GCCBOX_01	934 (+)	0	100.00	ERF2, ERF3, ERF4	GCCGCC
AS$GCCBOX_02	934 (+)	0	100.00	Pti4	GCCGCC
CHICK$BLCR_02	934 (-)	0	100.00	NF-E4, Sp1	GCGGC
AS$ZIC_16	934 (-)	1	87.50	Zic1	TGCGGGGGC
HS$CDC25C_04	935 (-)	0	100.00		GGCGG
CHICK$BLCR_02	937 (-)	0	100.00	NF-E4, Sp1	GCGGC
HS$TCRA_08	938 (+)	0	100.00	c-Ets-1, LEF-1, PEBP2alphaA1	CCGCA
AS$ZIC_19	938 (-)	1	87.50	Zic1	TGGGTGCGG
AS$ZIC2_10	938 (-)	1	87.50	Zic2	TGGTTGCTG
RAT$CYTOP_09	939 (+)	0	100.00	XF1, XF2	CGCAAC
HBV$HBVE_14	940 (-)	0	100.00	EF-C	GTTGC
RAT$ALBU_22	942 (+)	0	100.00	ACF	AACCAAT
HS$GG_33	942 (+)	0	100.00	CP1, NFE-6	AACCAAT
AD$MLP_03	942 (-)	0	100.00	CP1A, NF-YA	ATTGGTT
Y$LYS9_02	943 (+)	1	87.50	LYS14	TCCAATGGA
HS$BAC_03	944 (+)	0	100.00	SRF	CCAAT
AMULV$AMULV_01	944 (+)	0	100.00		CCAAT
EBV$DSL_06	944 (+)	0	100.00		CCAAT
MOUSE$AAG_04	944 (+)	0	100.00	alpha-CP1, alpha-CP2a, alpha-CP2b, alpha-IRP	CCAAT
CHICK$BAG_06	944 (+)	0	100.00	TGGCA-binding protein	CCAAT
HS$GG_15	944 (+)	0	100.00	NF-E	CCAAT
HS$GG_16	944 (+)	0	100.00	CDP2, Clox, CUTL1, Cutl1	CCAAT
HS$GG_17	944 (+)	0	100.00	gammaCAAT	CCAAT
HS$GG_18	944 (+)	0	100.00	CP1	CCAAT
HS$ZG_07	944 (+)	0	100.00	CP2	CCAAT
HS$ZG_08	944 (+)	0	100.00	CP1	CCAAT
HS$HH1_03	944 (+)	0	100.00	H1TF2	CCAAT
MOUSE$M2IAB_01	944 (+)	0	100.00	PU.1	CCAAT
PIG$UPA_12	944 (+)	0	100.00	NF-1	CCAAT
RADLV$RLV_10	944 (+)	0	100.00		CCAAT
MULV$MULV_03	944 (+)	0	100.00		CCAAT
MOUSE$GLUT4_04	944 (+)	0	100.00		CCAAT
MOMLV$MOMULV_15	944 (+)	0	100.00		CCAAT
RAT$ALDB_02	944 (+)	0	100.00	CBF (2), CP2	CCAAT
HS$GG_20	944 (+)	0	100.00	CP1, NF-E3	CCAAT
RAT$TH2B_01	944 (+)	0	100.00		CCAAT
RAT$TH2B_02	944 (+)	0	100.00		CCAAT
HS$GG_21	944 (+)	0	100.00	CP1	CCAAT
MOUSE$LY6E_01	944 (+)	0	100.00		CCAAT
RAT$NEU_01	944 (+)	0	100.00		CCAAT
HS$GHA_10	944 (+)	0	100.00	alpha-CBF	CCAAT
NEUCR$CPC1_02	944 (+)	0	100.00		CCAAT
HS$EG_07	944 (+)	0	100.00	CP1	CCAAT
HS$GP2B_13	944 (+)	0	100.00		CCAAT
MOUSE$BMG_10	944 (+)	0	100.00		CCAAT
HS$CYBH_01	944 (+)	0	100.00	CDP2, Clox, CP1, CUTL1, Cutl1	CCAAT
HBV$S_04	944 (+)	0	100.00		CCAAT
HS$FN_06	944 (+)	0	100.00		CCAAT
MOUSE$E2F1_02	944 (+)	0	100.00		CCAAT
HS$A24COL_01	944 (+)	0	100.00		CCAAT
MOUSE$EKLF_01	944 (+)	0	100.00	AP-1, GATA-1	CCAAT
MOUSE$A11COL_01	944 (-)	0	100.00	CBF (2), IF2	ATTGG
MOUSE$A21COL_03	944 (-)	0	100.00	CCAAT-binding factor	ATTGG
MOUSE$A21COL_04	944 (-)	0	100.00	CBF (2), CBF-A, CBF-B	ATTGG
MOUSE$A21COL_05	944 (-)	0	100.00	EFI	ATTGG
AD2$E2L_03	944 (-)	0	100.00	CRF	ATTGG
HS$GHA_06	944 (-)	0	100.00	NF-1	ATTGG
SP$H2B1_02	944 (-)	0	100.00	CBF (1)	ATTGG
SP$H2B1_03	944 (-)	0	100.00	CDF	ATTGG
HS$HSP70_02	944 (-)	0	100.00	CTF	ATTGG
HS$HSP70_07	944 (-)	0	100.00	CBTF, CP1, CTF	ATTGG
MOUSE$M2EAK_07	944 (-)	0	100.00	NF-Y	ATTGG
MOUSE$M2EAK_08	944 (-)	0	100.00	NF-Y	ATTGG
RSV$RSV_05	944 (-)	0	100.00		ATTGG
HSV1$TK_04	944 (-)	0	100.00	C/EBPalpha	ATTGG
HSV1$TK_05	944 (-)	0	100.00	NF-Y'	ATTGG
AS$HAP23_01	944 (-)	0	100.00	HAP2, HAP3, HAP4	ATTGG
MOUSE$M1H2KB_12	944 (-)	0	100.00	EFI	ATTGG
RAT$TH2A_02	944 (-)	0	100.00		ATTGG
RAT$TH2A_04	944 (-)	0	100.00		ATTGG
HS$TK_02	944 (-)	0	100.00	CP1	ATTGG
MOUSE$A11COL_07	944 (-)	0	100.00	NF-1, Sp1	ATTGG
HS$CDC2_07	944 (-)	0	100.00	CBAF, CBF-B, CP1A	ATTGG
XENLA$GATA2_01	944 (-)	0	100.00	CBTF	ATTGG
RAT$NAT_05	944 (-)	0	100.00		ATTGG
HS$CDC2_12	944 (-)	0	100.00	CBAF	ATTGG
Y$ADH2_01	952 (+)	0	100.00	ADR1	TCTCC
MOUSE$PCP2_01	952 (-)	0	100.00	RXR-beta, T3R-alpha1, T3R-beta1	AGGAGA
HS$TERT_03	953 (+)	1	87.50	Sp1	CTCCGCCTC
CHICK$BAG_07	953 (-)	1	87.50		GAGGAGGGG
HS$GG_25	954 (-)	0	100.00	PPUR	AGGAGGA
HT1$HTLV1_08	955 (+)	0	100.00	c-Ets-1 68	CCTCC
HS$AACS_02	955 (-)	0	100.00	ARP-1	AGGAGG
PA$PY_12	958 (-)	0	100.00		AGAGG
RAT$POMC_03	959 (-)	0	100.00	GR	CAGAG
RAT$HNF1_03	960 (-)	1	88.89	CWH-1, CWH-2, CWH-3, FOXG1, HNF-3B	TGTAAACAGA
MOUSE$CDX2_01	962 (+)	0	100.00	FOXM1a, FOXM1b, HNF-3alpha, HNF-3B	TGTTT
RAT$FABPI_04	962 (+)	0	100.00	FOXM1a, FOXM1b, HNF-3alpha, HNF-3B	TGTTT
HS$ALP_02	962 (+)	0	100.00	HNF-3alpha, HNF-3B	TGTTT
MOUSE$ECADH_03	962 (-)	0	100.00	FOXM1a, FOXM1b, HNF-3alpha, HNF-3B	AAACA
AS$FREAC2_01	963 (-)	0	100.00	FOXF2	TAAAC
MOUSE$WAP_06	964 (+)	0	100.00		TTTAAA
BOMMO$SER1_02	964 (+)	1	87.50		TTTACATAG
Y$MAL63_01	964 (+)	0	100.00	MIG1	TTTAA
HS$CYCB1_03	964 (+)	0	100.00		TTTAAAT
MOUSE$WAP_06	964 (-)	0	100.00		TTTAAA
Y$HO_08	964 (-)	1	87.50	PHO2	CAATTTAAA
DROME$ADH_09	965 (-)	0	100.00	TBP	TATTTAA
Y$LEU2_02	965 (-)	0	100.00	TBP, TFIIA, TOA1, TOA2	TATTTAA
Y$MAL63_01	965 (-)	0	100.00	MIG1	TTTAA
HS$GRH_03	966 (+)	0	100.00	POU1F1a	TAAAT
BOVIN$IRBP_04	966 (-)	0	100.00	Crx	CTATTTA
HS$MSH2_01	967 (-)	0	100.00	HNF-3alpha, HNF-3B	TATTT
AS$GHBF_02	971 (+)	1	87.50	G/HBF-1	AGACTCACC
AS$ZIC_08	972 (-)	1	87.50	Zic1	CGGGGAGTC
DROME$EVE_10	974 (+)	0	100.00	GAGA factor	CTCGC
PL$H2A_01	976 (+)	1	87.50		CACCGTGTC
AS$PAX4_20	978 (-)	1	92.86	Pax-4a	AAATGATTGACACTG
RAT$MHC_01	980 (+)	1	88.89	NFe	GTGTCAGTCA
CHICK$OA_02	980 (+)	0	100.00	ARP-1, COUP, COUP-TF1, PPAR-alpha, RXR-alpha	GTGTCA
NFE$CONS	980 (+)	1	88.89	NFe	GTGTCAGTCA
MAIZE$C1_05	980 (+)	0	100.00		GTGTC
AS$ATHB5_02	980 (+)	1	90.91	ATHB-5	GTGCCAATCATT
AS$MEIS1_02	980 (-)	0	100.00	Meis-1a, Meis-1b	TGACAC
AS$MEIS1BHOXA9_10	980 (-)	0	100.00	HOXA9, Meis-1b	TGACAC
AS$MEIS1BHOXA9_12	980 (-)	0	100.00	HOXA9, Meis-1b	TGACAC
AS$MEIS1BHOXA9_13	980 (-)	0	100.00	HOXA9, Meis-1b	TGACAC
AS$MEIS1BHOXA9_14	980 (-)	0	100.00	HOXA9, Meis-1b	TGACAC
AS$PCE_01	980 (-)	1	88.89	Meis-1a, Meis-1c, Pbx-1a, Pbx-1b, PKNOX1	TGATTGACAG
AS$TGIF_01	981 (+)	0	100.00	TGIF	TGTCA
AS$TGIF_02	981 (+)	0	100.00	TGIF	TGTCA
AS$TGIF_03	981 (+)	0	100.00	TGIF	TGTCA
AS$TGIF_04	981 (+)	0	100.00	TGIF	TGTCA
AS$TGIF_05	981 (+)	0	100.00	TGIF	TGTCA
AS$TGIF_06	981 (+)	0	100.00	TGIF	TGTCA
AS$TGIF_07	981 (+)	0	100.00	TGIF	TGTCA
AS$TGIF_08	981 (+)	0	100.00	TGIF	TGTCA
AS$TGIF_10	981 (+)	0	100.00	TGIF	TGTCA
AS$TGIF_11	981 (+)	0	100.00	TGIF	TGTCA
AS$TGIF_12	981 (+)	0	100.00	TGIF	TGTCA
AS$TGIF_13	981 (+)	0	100.00	TGIF	TGTCA
AS$TGIF_14	981 (+)	0	100.00	TGIF	TGTCA
AS$TGIF_15	981 (+)	0	100.00	TGIF	TGTCA
POT$PR10a_01	981 (+)	0	100.00	PBF-1, PBF-2 (p24)	TGTCA
POT$PR10a_01	981 (-)	0	100.00	PBF-1, PBF-2 (p24)	TGACA
AS$TWRKY_01	982 (-)	0	100.00	WRKY3, WRKY4	TTGAC
AS$WRKY_01	982 (-)	0	100.00	WRKY1, WRKY2	TTGAC
AS$WRKY_02	982 (-)	0	100.00	WRKY1, WRKY2	TTGAC
AS$WRKY_03	982 (-)	0	100.00	WRKY1, WRKY2	TTGAC
AS$WRKY_04	982 (-)	0	100.00	WRKY1, WRKY2	TTGAC
AT$RLK4_01	982 (-)	0	100.00	WRKY18	TTGAC
AT$RLK4_02	982 (-)	0	100.00	WRKY18	TTGAC
HS$EGFR_15	983 (+)	0	100.00		TCAAT
DROME$EN_01	983 (+)	1	88.89	En, Eve, Ftz, Prd, Zen-1, Zen-2	TCAATCAATT
PSAM$U7SN_04	983 (-)	0	100.00		ATTGA
AS$HOX1_02	984 (+)	1	87.50	HOX1, HOX2, HOX3, HOX4, HOX5, HOX6, HOX7	CAATCATTG
AS$ATHB2_02	984 (+)	1	87.50	ATHB-2, GLABRA2	CAATCATTG
AS$HDE_02	984 (-)	1	87.50	CPHB-1	CAATGATTG
AS$ATHB2_01	984 (-)	1	87.50	ATHB-2, GLABRA2	CAATGATTG
MOUSE$BMG_01	985 (-)	0	100.00	GATA-1	TGATT
MOUSE$IGH_55	986 (+)	0	100.00		ATCAT
MOUSE$IL4_01	987 (+)	0	100.00	NF-CLE0a, NF-CLE0b	TCATTT
HS$GMCSF_03	988 (+)	0	100.00	YY1	CATTT
Y$MAL61_03	989 (+)	0	100.00	MIG1	ATTTT
Y$GAL4_01	990 (+)	0	100.00	MIG1	TTTTC
HS$TNFA_04	991 (-)	0	100.00		AGAAA
I$HSF_01	991 (-)	0	90.00	HSF	AGAAN
F$HSF_01	991 (-)	0	90.00	HSF	AGAAN
MOUSE$RAS1_02	993 (-)	0	100.00	GR	TCTTCT
I$HSF_01	994 (-)	0	90.00	HSF	AGAAN
F$HSF_01	994 (-)	0	90.00	HSF	AGAAN
RAT$TAT_27	997 (+)	1	87.50	CREB, CREBbeta	CTGCGTCAG
Y$HIS3_01	999 (-)	0	100.00	GCN4	TGACGA
HT1$HTLV1_20	999 (-)	0	100.00	TAF-I, TAF-II, Tax	TGACGA
RAT$SMAD7_03	999 (-)	0	100.00		TGACGA
TDNA$NOS_01	1000 (+)	0	100.00	ASF-1, OBF3.1, TGA1a, [illegible]	CGTCAG

TDNA$NOS_01	1000 (+)	0	100.00	ASF-1, OBF3.1, TGA1a, TGA1b	CGTCAG
HS$VIP_01	1000 (+)	0	100.00	c-Fos, c-Jun, CRE-BP1, CREB	CGTCA
NFS$CONS	1000 (+)	0	92.86	NF-S	YGTCAGC
CAMV$35SR_01	1000 (-)	0	100.00	ASF-1, OBF4, OBF5, SARP, TGA1, TGA1a, TGA1b, TGA2, TGA2.1, TGA2.2, TGA3, TGA6, ZAP1	TGACG
PIG$UPA_02	1000 (-)	0	100.00	CREB	TGACG
PIG$UPA_03	1000 (-)	0	100.00	CREB, CREBbeta	TGACG
HS$INS_04	1000 (-)	0	100.00	CREB	TGACG
HS$PL_12	1000 (-)	0	100.00		TGACG
RAT$EAI_09	1001 (+)	0	100.00		GTCAG
HS$GX_WT1_01	1003 (-)	1	87.50	WT1 -KTS, WT1 I, WT1 I -KTS, WT1 I-del2, WT1-del2	GTGAGGCTG
MOUSE$MYOD_04	1003 (-)	0	100.00	ARP-1, RXR-alpha, RXR-gamma, T3R-alpha	AGGCTG
Y$GAL1_10	1004 (+)	0	100.00	GAL4	AGCCT
LP$LPS1B_01	1004 (+)	1	87.50		AGCCCCCCT
SV$SV40_01	1005 (+)	0	100.00	T-Ag	GCCTC
SV$SV40_02	1005 (+)	0	100.00	T-Ag	GCCTC
SV$SV40_63	1005 (+)	0	100.00	T-Ag	GCCTC
PA$PY_24	1005 (-)	0	100.00	T-Ag	GAGGC
PA$PY_25	1005 (-)	0	100.00	T-Ag	GAGGC
SV$SV40_02	1005 (-)	0	100.00	T-Ag	GAGGC
HT1$HTLV1_08	1006 (+)	0	100.00	c-Ets-1 68	CCTCC
HS$DG_03	1006 (+)	1	88.89		CCTTCCTTCC
HS$SREC_01	1006 (+)	0	100.00	EZF-2	CCTCCCT
HS$SREC_01	1006 (-)	0	100.00	EZF-2	AGGGAGG
HS$GX_WT1_02	1007 (+)	1	87.50	WT1 -KTS, WT1 I, WT1 I -KTS, WT1 I-del2, WT1-del2	CTCCCTCCC
HS$CS1_04	1007 (-)	0	100.00	T3R	AGGGAG
HS$P21WAF1_05	1007 (-)	0	100.00	RXR-alpha, VDR	AGGGAG
AS$VDR_08	1007 (-)	0	100.00	VDR	AGGGAG
HS$GG_28	1010 (-)	0	100.00	PPUR	TGGAAGG
XENLA$RPL14_01	1011 (+)	0	100.00	HrpF, XrpFI	CTTCC
Y$TPI_01	1011 (+)	0	100.00	GCR1	CTTCC
MOUSE$RPL32_01	1011 (-)	0	100.00	f(alpha)-f(epsilon), HrpF, XrpFI	GGAAG
HS$TNFA_02	1011 (-)	0	100.00		GGAAG
HS$TNFA_03	1011 (-)	0	100.00		GGAAG
HT1$HTLV1_08	1014 (+)	0	100.00	c-Ets-1 68	CCACC
HS$BG_52	1014 (+)	0	100.00		CCACC
HS$BG_14	1014 (+)	0	100.00		CCACC
HS$BG_44	1014 (+)	0	100.00	CAC-binding protein	CCACC
BPV1$BPV1_24	1014 (-)	0	100.00	Sp1	GGTGG
HS$BG_48	1014 (-)	0	100.00	CAC-binding protein	GGTGG
MOUSE$CEBPA_06	1014 (-)	0	100.00	Sp1	GGTGG
HS$CDC25C_04	1017 (-)	0	100.00		GGCGG
MULV$MULV_01	1018 (+)	0	100.00	UCRF-L	CGCCAT
MULV$MULV_02	1018 (+)	1	87.50	YY1	CGCCATTTT
HS$CFOS_06	1020 (+)	1	88.89	SRF (504 AA)	CCATATTAGG
HS$CFOS_16	1020 (+)	1	88.89	MCM1	CCATATTAGG
AS$SRF_02	1020 (+)	1	88.89	MCM1, SRF, STE12	CCATATTAGG
CHICK$AAC_03	1020 (-)	1	88.89	MF3, SRF, YY1	CCAAATATGG
CF1$CONS_01	1020 (-)	0	91.67	YY1	ANATGG
MOUSE$SAACT_01	1020 (-)	0	100.00	YY1	ATATGG
AS$HOXA3_13	1021 (+)	1	87.50	HOXA3	CATGTTGGG
MOUSE$A11COL_01	1024 (+)	0	100.00	CBF (2), IF2	ATTGG
MOUSE$A21COL_03	1024 (+)	0	100.00	CCAAT-binding factor	ATTGG
MOUSE$A21COL_04	1024 (+)	0	100.00	CBF (2), CBF-A, CBF-B	ATTGG
MOUSE$A21COL_05	1024 (+)	0	100.00	EFI	ATTGG
AD2$E2L_03	1024 (+)	0	100.00	CRF	ATTGG
HS$GHA_06	1024 (+)	0	100.00	NF-1	ATTGG
SP$H2B1_02	1024 (+)	0	100.00	CBF (1)	ATTGG
SP$H2B1_03	1024 (+)	0	100.00	CDF	ATTGG
HS$HSP70_02	1024 (+)	0	100.00	CTF	ATTGG
HS$HSP70_07	1024 (+)	0	100.00	CBTF, CP1, CTF	ATTGG
MOUSE$M2EAK_07	1024 (+)	0	100.00	NF-Y	ATTGG
MOUSE$M2EAK_08	1024 (+)	0	100.00	NF-Y	ATTGG
RSV$RSV_05	1024 (+)	0	100.00		ATTGG
HSV1$TK_04	1024 (+)	0	100.00	C/EBPalpha	ATTGG
HSV1$TK_05	1024 (+)	0	100.00	NF-Y'	ATTGG
AS$HAP23_01	1024 (+)	0	100.00	HAP2, HAP3, HAP4	ATTGG
MOUSE$M1H2KB_12	1024 (+)	0	100.00	EFI	ATTGG
RAT$TH2A_02	1024 (+)	0	100.00		ATTGG
RAT$TH2A_04	1024 (+)	0	100.00		ATTGG
HS$TK_02	1024 (+)	0	100.00	CP1	ATTGG
MOUSE$A11COL_07	1024 (+)	0	100.00	NF-1, Sp1	ATTGG
HS$CDC2_07	1024 (+)	0	100.00	CBAF, CBF-B, CP1A	ATTGG
XENLA$GATA2_01	1024 (+)	0	100.00	CBTF	ATTGG
RAT$NAT_05	1024 (+)	0	100.00		ATTGG
HS$CDC2_12	1024 (+)	0	100.00	CBAF	ATTGG
HS$BAC_03	1024 (-)	0	100.00	SRF	CCAAT
AMULV$AMULV_01	1024 (-)	0	100.00		CCAAT
EBV$DSL_06	1024 (-)	0	100.00		CCAAT
MOUSE$AAG_04	1024 (-)	0	100.00	alpha-CP1, alpha-CP2a, alpha-CP2b, alpha-IRP	CCAAT
CHICK$BAG_06	1024 (-)	0	100.00	TGGCA-binding protein	CCAAT
HS$GG_15	1024 (-)	0	100.00	NF-E	CCAAT
HS$GG_16	1024 (-)	0	100.00	CDP2, Clox, CUTL1, Cutl1	CCAAT
HS$GG_17	1024 (-)	0	100.00	gammaCAAT	CCAAT
HS$GG_18	1024 (-)	0	100.00	CP1	CCAAT
HS$ZG_07	1024 (-)	0	100.00	CP2	CCAAT
HS$ZG_08	1024 (-)	0	100.00	CP1	CCAAT
HS$HH1_03	1024 (-)	0	100.00	H1TF2	CCAAT
MOUSE$M2IAB_01	1024 (-)	0	100.00	PU.1	CCAAT
PIG$UPA_12	1024 (-)	0	100.00	NF-1	CCAAT
RADLV$RLV_10	1024 (-)	0	100.00		CCAAT
MULV$MULV_03	1024 (-)	0	100.00		CCAAT
MOUSE$GLUT4_04	1024 (-)	0	100.00		CCAAT
MOMLV$MOMULV_15	1024 (-)	0	100.00		CCAAT
RAT$ALDB_02	1024 (-)	0	100.00	CBF (2), CP2	CCAAT
HS$GG_20	1024 (-)	0	100.00	CP1, NF-E3	CCAAT
RAT$TH2B_01	1024 (-)	0	100.00		CCAAT
RAT$TH2B_02	1024 (-)	0	100.00		CCAAT
HS$GG_21	1024 (-)	0	100.00	CP1	CCAAT
MOUSE$LY6E_01	1024 (-)	0	100.00		CCAAT
RAT$NEU_01	1024 (-)	0	100.00		CCAAT
HS$GHA_10	1024 (-)	0	100.00	alpha-CBF	CCAAT
NEUCR$CPC1_02	1024 (-)	0	100.00		CCAAT
HS$EG_07	1024 (-)	0	100.00	CP1	CCAAT
HS$GP2B_13	1024 (-)	0	100.00		CCAAT
MOUSE$BMG_10	1024 (-)	0	100.00		CCAAT
HS$CYBH_01	1024 (-)	0	100.00	CDP2, Clox, CP1, CUTL1, Cutl1	CCAAT
HBV$S_04	1024 (-)	0	100.00		CCAAT
HS$FN_06	1024 (-)	0	100.00		CCAAT
MOUSE$E2F1_02	1024 (-)	0	100.00		CCAAT
HS$A24COL_01	1024 (-)	0	100.00		CCAAT
MOUSE$EKLF_01	1024 (-)	0	100.00	AP-1, GATA-1	CCAAT
HS$CRP_03	1025 (-)	1	88.89	C/EBPalpha, C/EBPbeta, C/EBPdelta	AGTGGCGCAA
SV$SV40_63	1026 (+)	0	100.00	T-Ag	TGGGC
HS$GRH_01	1028 (+)	0	100.00	AP-2, AP-2alphaA, AP-2alphaB, NF-1	GGCCA
RAT$TAT_15	1028 (+)	0	100.00	HNF-3alpha	GGCCA
HS$GRH_01	1028 (-)	0	100.00	AP-2, AP-2alphaA, AP-2alphaB, NF-1	TGGCC
Y$SPS4_01	1029 (+)	1	87.50	NDT80	GCCACAAAA
AS$HB_15	1031 (+)	1	88.89	Hb	CACTAAAAAT
DROME$UBX_18	1031 (-)	1	88.89	Hb	TTTTTTAATG
RAT$AMHC_04	1033 (+)	1	87.50	aMEF-2, MEF-2 (516 AA)	CTAAAAAAG
AS$MEF2_07	1033 (+)	1	87.50	MEF-2	CTAAAAATA
AS$PAX2_61	1033 (+)	1	87.50	Pax-2.1, Pax-2.2	CTAAAAGAA
MOUSE$ADA_06	1034 (+)	0	100.00	TBP	TAAAAAA
Y$CLB2_04	1034 (+)	1	88.89	MCM1	TCAAAAAAGG
Y$MAL2R_01	1034 (-)	0	100.00	MIG1	TTTTA
AS$FOXJ1_04	1034 (-)	0	100.00	FOXJ1	TTTTA
Y$SUC2_01	1035 (-)	0	100.00	MIG1	TTTTT
AS$PF1_01	1035 (-)	0	100.00	PF1	TTTTT
AS$PF1_02	1035 (-)	0	100.00	PF1	TTTTT
Y$SUC2_01	1036 (-)	0	100.00	MIG1	TTTTT
AS$PF1_01	1036 (-)	0	100.00	PF1	TTTTT
AS$PF1_02	1036 (-)	0	100.00	PF1	TTTTT
Y$SUC2_01	1037 (-)	0	100.00	MIG1	TTTTT
AS$PF1_01	1037 (-)	0	100.00	PF1	TTTTT
AS$PF1_02	1037 (-)	0	100.00	PF1	TTTTT
Y$GSY2_02	1039 (+)	0	100.00		AAAGGGG
LP$LPS1B_01	1040 (-)	1	87.50		AGCCCCCCT
AS$VDR_03	1041 (+)	0	100.00	VDR	AGGGGG
AS$VDR_04	1041 (+)	0	100.00	VDR	AGGGG
AS$VDR_05	1041 (+)	0	100.00	VDR	AGGGGG
AS$VDR_07	1041 (+)	0	100.00	VDR	AGGGGG
AS$VDR_09	1041 (+)	0	100.00	VDR	AGGGGG
Y$GLK1_02	1041 (+)	0	100.00	MSN2, MSN4	AGGGG
AS$SPZ1_08	1042 (+)	1	87.50	Spz1	GGGGGGTCG
HS$CYCD1_14	1042 (-)	0	100.00	Sp1, Sp2, Sp3, Sp4	CCCCC
HS$CYCD1_15	1042 (-)	0	100.00	Sp1, Sp2, Sp3, Sp4	CCCCC
PA$PY_23	1043 (+)	0	100.00	T-Ag	GGGGC
SV$SV40_04	1043 (+)	0	100.00	T-Ag	GGGGC
SV$SV40_63	1043 (+)	0	100.00	T-Ag	GGGGC
PA$PY_26	1043 (-)	0	100.00	T-Ag	GCCCC
MAIZE$ADH11S_06	1043 (-)	0	100.00	GCBP-1, Sp1	GCCCC
Y$GAL4_01	1053 (+)	0	100.00	MIG1	TTTTC
Y$MEL1_01	1055 (+)	0	100.00	GAL4	TTCGG
AS$ZIC2_17	1055 (+)	1	87.50	Zic2	TTCGTGGTG
Y$MEL1_01	1055 (-)	0	100.00	GAL4	CCGAA
AS$SP1_05	1056 (+)	1	88.89	Sp1	TCGGGGTGGT
AS$SP1_28	1056 (+)	1	88.89	Sp1	TCGGGGTGGT
HS$AG_15	1058 (+)	0	100.00	CAC-binding protein	GGGGTG
AS$SPZ1_05	1058 (+)	1	87.50	Spz1	GGGGGGTTT
AS$ZIC2_11	1058 (+)	1	87.50	Zic2	GGGGTGTTG
CHICK$PTH_01	1059 (+)	0	100.00	RXR-alpha, VDR	GGGTGT
HS$BG_01	1059 (-)	0	100.00	CACCC-binding factor	CACCC
HS$GG_13	1059 (-)	0	100.00	gammaCAC1, gammaCAC2	CACCC
HS$GG_14	1059 (-)	0	100.00	CACCC-binding factor, Sp1	CACCC
HS$GP2B_01	1059 (-)	0	100.00	CACCC-binding factor	CACCC
RAT$TOA_02	1059 (-)	0	100.00	CACCC-binding factor	CACCC
HS$EG_06	1059 (-)	0	100.00	Sp1	CACCC
AS$CACCC_01	1059 (-)	0	100.00	CACCC-binding factor	CACCC
HS$CYP24_01	1059 (-)	0	100.00	VDR	ACACCC
MOUSE$CDX2_01	1062 (+)	0	100.00	FOXM1a, FOXM1b, HNF-3alpha, HNF-3B	TGTTT
RAT$FABPI_04	1062 (+)	0	100.00	FOXM1a, FOXM1b, HNF-3alpha, HNF-3B	TGTTT
HS$ALP_02	1062 (+)	0	100.00	HNF-3alpha, HNF-3B	TGTTT
MOUSE$ECADH_03	1062 (-)	0	100.00	FOXM1a, FOXM1b, HNF-3alpha, HNF-3B	AAACA
HS$ADH2_11	1062 (-)	0	100.00	GR	AAAACA
DL$CONS_02	1063 (-)	0	88.89	Dl	GRGAAAANC
Y$SUC2_01	1064 (+)	0	100.00	MIG1	TTTTT
AS$PF1_01	1064 (+)	0	100.00	PF1	TTTTT
AS$PF1_02	1064 (+)	0	100.00	PF1	TTTTT
AS$HB_07	1064 (-)	1	88.89	Hb	GGGAAAAAAA
NT$PR1A_03	1064 (-)	0	100.00	GT-1	GAAAAA
Y$GAL4_01	1065 (+)	0	100.00	MIG1	TTTTC
HS$TNFA_04	1066 (-)	0	100.00		AGAAA
AS$ZIC2_23	1066 (-)	1	87.50	Zic2	GGGGAGATA
I$HSF_01	1066 (-)	0	90.00	HSF	AGAAN
F$HSF_01	1066 (-)	0	90.00	HSF	AGAAN
Y$ADH2_01	1068 (+)	0	100.00	ADR1	TCTCC
AS$LYF1_05	1068 (-)	0	100.00	LyF-1	GGGAGA
CHICK$ITGB3_01	1068 (-)	0	100.00	RXR-beta, VDR	GGGAGA
HS$TERT_03	1069 (+)	1	87.50	Sp1	CTCCGCCTC
RAT$IGF1R_06	1069 (-)	1	87.50	WT1 -KTS, WT1 I -KTS	GAGGGGGAA
RAT$IGF1R_10	1069 (-)	1	87.50	WT1 -KTS, WT1 I -KTS	GAGGGGGAA
HS$AAC_13	1070 (+)	1	90.00	Sp1	GCCCCCTCCCC
CHICK$CA2_02	1070 (+)	0	100.00	RXR-alpha, VDR	TCCCCC
HS$RAS1_05	1071 (+)	1	88.89	Sp1	CCCCCGCCCC
HS$GX_WT1_02	1071 (+)	1	87.50	WT1 -KTS, WT1 I, WT1 I -KTS, WT1 I-del2, WT1-del2	CTCCCTCCC
HS$A24COL_02	1071 (+)	1	90.00		CCCCCTCCCCC
HS$PFKM_07	1071 (+)	1	88.89		ACCCCTCCCC
HS$CYCD1_14	1071 (+)	0	100.00	Sp1, Sp2, Sp3, Sp4	CCCCC
HS$CYCD1_15	1071 (+)	0	100.00	Sp1, Sp2, Sp3, Sp4	CCCCC
HS$PFKM_03	1071 (-)	1	88.89		GGGGAGGAGG
AS$VDR_03	1071 (-)	0	100.00	VDR	AGGGGG
AS$VDR_05	1071 (-)	0	100.00	VDR	AGGGGG
AS$VDR_07	1071 (-)	0	100.00	VDR	AGGGGG
AS$VDR_09	1071 (-)	0	100.00	VDR	AGGGGG
HS$ZG_06	1072 (+)	0	100.00	Sp1	CCCCTCCCC
HS$NPY_04	1072 (+)	0	100.00	Sp1	CCCCTCC
HS$GPC_06	1072 (+)	1	90.00	Sp1	CCCCTCCCCTC
MOUSE$P53_07	1072 (+)	0	100.00	ETF	CCCCTCCC
HS$MIP_06	1072 (+)	0	100.00	Sp1, Sp3	CCCCTCCC
RAT$CYP2D5_01	1072 (+)	0	100.00	Sp1	CCCCTCCC
CHICK$BAG_07	1072 (-)	1	87.50		GAGGAGGGG
HS$MT2A_10	1072 (-)	1	87.50	Sp1	GGGGCGGGG
HS$AAC_10	1072 (-)	1	88.89	Sp1	GGGGGAGGGG
RAT$GSTP_09	1072 (-)	1	87.50		GGGGCGGGG
MOUSE$PFK_05	1072 (-)	1	87.50		GGGGCGGGG
HS$FN_07	1072 (-)	1	87.50	Sp1	GGGGCGGGG
HS$MIP_03	1072 (-)	0	100.00	Sp1	GGGGAGGGG
AS$VDR_04	1072 (-)	0	100.00	VDR	AGGGG
Y$GLK1_02	1072 (-)	0	100.00	MSN2, MSN4	AGGGG
CTCF$CONS	1073 (+)	0	100.00	CTCF	CCCTC
MOUSE$THY1_04	1073 (+)	0	100.00	R1, R2, Sp1	CCCTCCC
H4TF1$CONS	1073 (-)	1	87.50	H4TF-1	GGGGGAGGG
MOUSE$CMYC_04	1073 (-)	0	100.00	MAZ	GGGAGGG
MOUSE$CD4_04	1073 (-)	0	100.00	MAZ	GGGAGGG
HT1$HTLV1_08	1074 (+)	0	100.00	c-Ets-1 68	CCTCC
MOUSE$THY1_05	1074 (+)	1	87.50		CCACCCCTG
MOUSE$AQP7_01	1076 (-)	0	100.00	PPAR-alpha, PPAR-gamma, RXR-alpha	AGGGGA
AS$VDR_04	1077 (-)	0	100.00	VDR	AGGGG
Y$GLK1_02	1077 (-)	0	100.00	MSN2, MSN4	AGGGG
AS$CF1_18	1079 (-)	1	87.50	CF1	GGGGTCAGG
Y$ACT1_01	1080 (+)	1	87.50	REB1	CTGTCACCG
HS$GG_12	1080 (+)	0	100.00	NF-E	CTGTC
DROME$EVE_10	1080 (+)	0	100.00	GAGA factor	CTGTC
AS$MEIS1_13	1080 (+)	0	100.00	Meis-1a, Meis-1b	CTGTCC
RAT$A2UG_10	1081 (+)	0	100.00	GR	TGTCCC
AS$PR_01	1081 (-)	0	100.00	PR B	GGGACA
EBV$IR4_04	1082 (-)	0	100.00	R	GGGAC
EBV$IR4_05	1082 (-)	0	100.00	R	GGGAC
MZF1$CONS_01	1083 (-)	0	92.86	MZF-1	GNGGGGA
V$MZF1_01	1083 (-)	0	87.50	MZF1	NGNGGGGA
Y$SUC2_01	1084 (+)	0	100.00	MIG1	CCCCGC
Y$PDC1_02	1084 (+)	0	100.00	MIG1	CCCCGC
EG$CCR_01	1085 (-)	0	100.00	LMC1, LMC2	AGCGGG
AMV$AMV_02	1089 (+)	0	100.00	C/EBPalpha	CTTGC
RAV0$RAV0_01	1089 (-)	0	100.00	C/EBPalpha	GCAAG
EBV$DSL_01	1090 (+)	0	100.00	Zta	TTGCTCA
EBV$DSL_07	1090 (-)	0	100.00	Zta	TGAGCAA
RAT$VEGF_02	1091 (-)	0	100.00	ER-alpha, ER-beta	GAGCA
TDNA$NOS_01	1092 (-)	0	100.00	ASF-1, OBF3.1, TGA1a, TGA1b	TGAGC

Number of sites found: 2313

Start a new search

TP53
JASPAR RARA/RXRA MOTIF

SIB | EPD EUKARYOTIC PROMOTER DATABASE

Check out our short survey | Follow @EPD_SIB

Computational Cancer Genomics | ExPASy | EPFL

- **Access EPDnew**
  - *H. sapiens*
  - *H. sapiens non-coding*
  - *M. mulatta*
  - *M. musculus*
  - *M. musculus non-coding*
  - *R. norvegicus*
  - *C. familiaris*
  - *G. gallus*
  - *D. melanogaster*
  - *A. mellifera*
  - *D. rerio*
  - *C. elegans*
  - *A. thaliana*
  - *Z. mays*
  - *S. cerevisiae*
  - *S. pombe*
  - *P. falciparum*
  - Select / Download
  - Promoter analysis tools
  - FTP site
- **Access EPD**
  - Promoter elements
  - Select / Download
  - FTP site
- **Access MGA Database**
  - MGA-Search
  - MGA Data Overview
  - MGA FTP site
- **Documents**
- **Other Resources**
- **References**
- **What is new**
- **Contact us**

in H. sapiens | SEARCH

Please help us make EPD better! Take our short survey to suggest new tools or organism covered. It will take only a few minutes.

General Information

[Help]

Promoter ID:	TP53_1
Promoter type:	region
Organism:	Homo sapiens (Human)
Gene Symbol:	TP53
Description of the gene:	tumor protein p53
Sequence:	tttcccctcccatgtgctcaagactggcgctaaaagttttgagcttctcAAAAGTCTAGA
Position in the genome:	Chromosome [NC_000017.11]; Strand [-]; Position [7687487]
Ensembl:	ENSG00000141510
RefSeq:	NM_001276696
NCBI Gene:	TP53
GeneCards:	TP53

Promoter Image [View it at UCSC]

[Help]

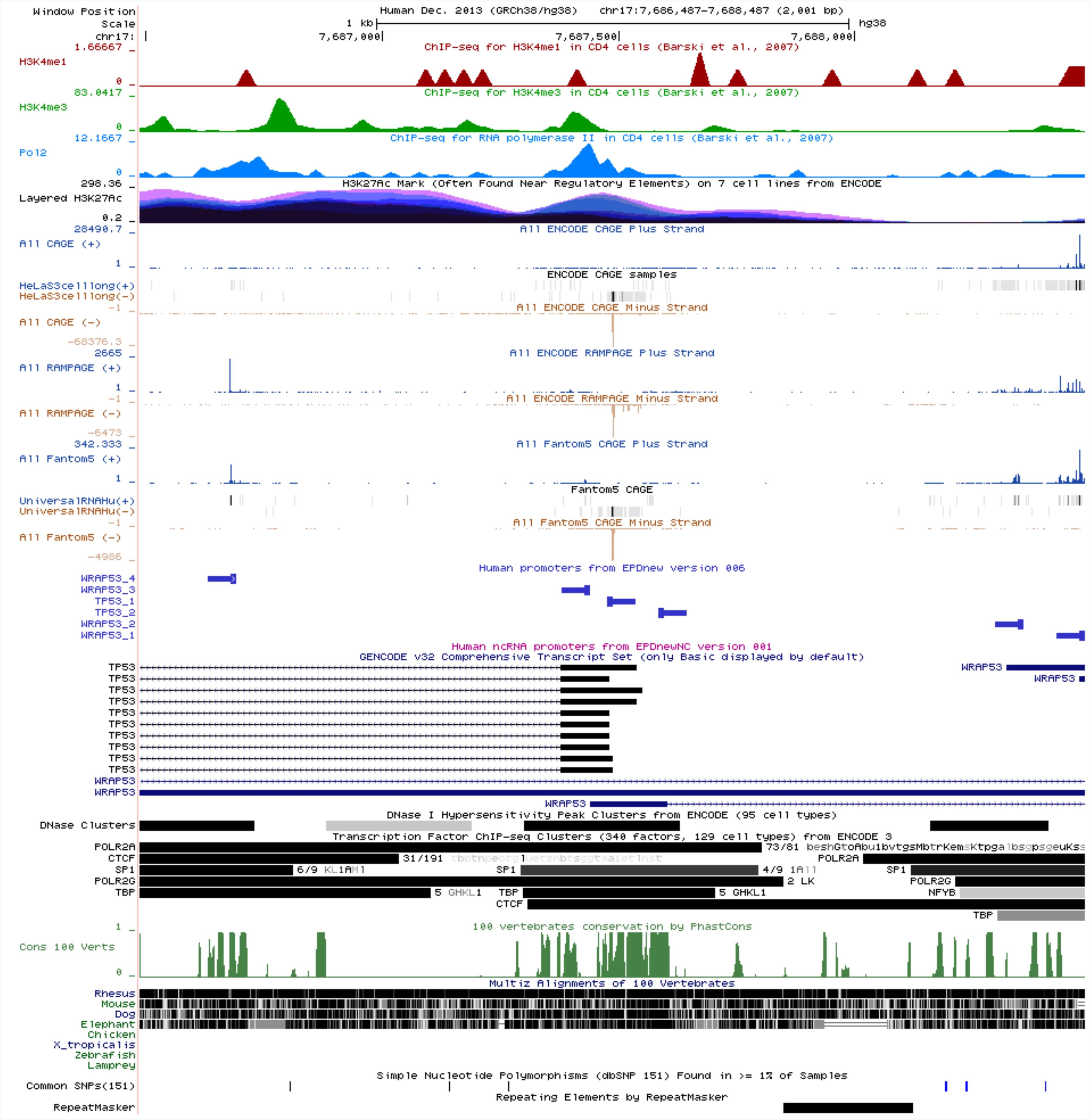

Other viewers

[Help]

EPD view at UCSC (hg38):	chr17:7685487–7689487 (European mirror)
EPD view at UCSC (hg19):	chr17:7588805–7592805 (European mirror)
SwissRegulon (hg19):	chr17:7588805–7592805
FANTOM5 (hg38):	chr17:7685487–7689487
DBTSS/KERO (hg38):	chr17:7685487–7689487
refTSS (hg38):	chr17:7685487–7689487 (European mirror)

Selective viewer showing the 3 Fantom5 tissues with the highest expression of **TP53_1** (European mirror)

GM12878 viewer (European mirror)

Sequence Retrieval Tool

[Help]

Get sequence TP53_1 **from** -1000 **to** 100 bp relative to TSS **GET SEQUENCE**

lower case upstream TSS

```
>FP022657 TP53_1 :+U EU:NC; range -1000 to 100.
TTGAAAGCACTGTGTTCCTTAGCACCGCGGGTCGCTACGGGCCTCTTGCTGTCGCGGGAT
TTCGGTCCACCTTCCGATTGGGCCGCCGCATCCCGGATCAGATTTCGCGGGCGACCCACG
GAACCCGCGGAGCCGGGACGTGAAAGGTTAGAAGGTTTCCCGTTCCCATCAAGCCCTAGG
GCTCCTCGTGGCTGCTGGGAGTTGTAGTCTGAACGCTTCTATCTTGGCGAGAAGCGCCTA
CGCTCCCCCTACCGAGTCCCGCGGTAATTCTTAAAGCACCTGCACCGCCCCCCCGCCGCC
TGCAGAGGGCGCAGCAGGTCTTGCACCTCTTCTGCATCTCATTCTCCAGGCTTCAGACCT
GTCTCCCTCATTCAAAAAATATTTATTATCGAGCTCTTACTTGCTACCCAGCACTGATAT
AGGCACTCAGGAATACAACAATGAATAAGATAGTAGAAAAATTCTATATCCTCATAAGGC
TTACGTTTCCATGTACTGAAAGCAATGAACAAATAAATCTTATCAGAGTGATAAGGGTTG
TGAAGGAGATTAAATAAGATGGTGTGATATAAAGTATCTGGGAGAAAACGTTAGGGTGTG
ATATTACGGAAAGCCTTCCTAAAAAATGACATTTTAACTGATGAGAAGAAAGGATCCAGC
TGAGAGCAAACGCAAAAGCTTTCTTCCTTCCACCCTTCATATTTGACACAATGCAGGATT
CCTCCAAAATGATTTCCACCAATTCTGCCCTCACAGCTCTGGCTTGCAGAATTTTCCACC
CCAAAATGTTAGTATCTACGGCACCAGGTCGGCGAGAATCCTGACTCTGCACCCTCCTCC
CCAACTCCATTTCCTTTGCTTCCTCCGGCAGGCGGATTACTTGCCCTTACTTGTCATGGC
GACTGTCCAGCTTTGTGCCAGGAGCCTCGCAGGGGTTGATGGATTGGGGTTTTCCCCTC
CCATGTGCTCAAGACTGGCGCTAAAAGTTTTGAGCTTCTCAAAAGTCTAGAGCCACCGTC
CAGGGAGCAGGTAGCTGCTGGGCTCCGGGGACACTTTGCGTTCGGGCTGGGAGCGTGCTT
TCCACGACGGTGACACGCTTC
```

Search Motif Tool

[Help]

Library: Transcription Factor Motifs (JASPAR Motif: RARA::RXRA

From: -1000 To: 100 bp relative to TSS and a cut-off (p-value) of: 0.001 **SEARCH**

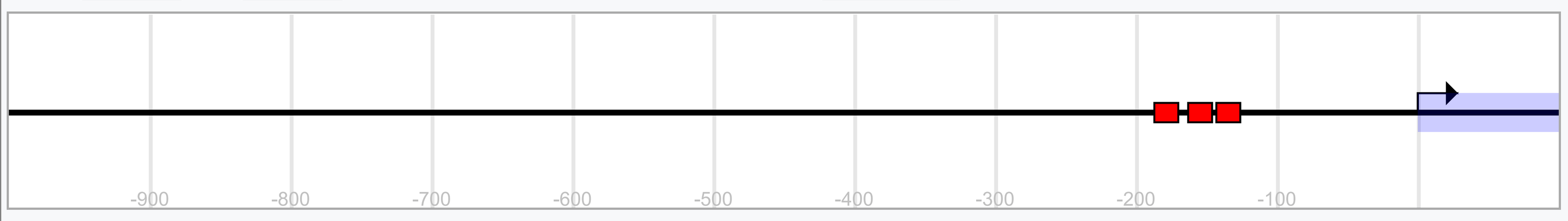

RARA::RXRA [p-value = 0.001]: -143, -163, -187

Expression Profile Tool

[Help]

TP53_1 is expressed in **1037 samples** with an **average expression of 277.229 tags** per 10M.

The TSSs distribution around the EPDnew annotated start site (click on the histogram bars to explore samples):

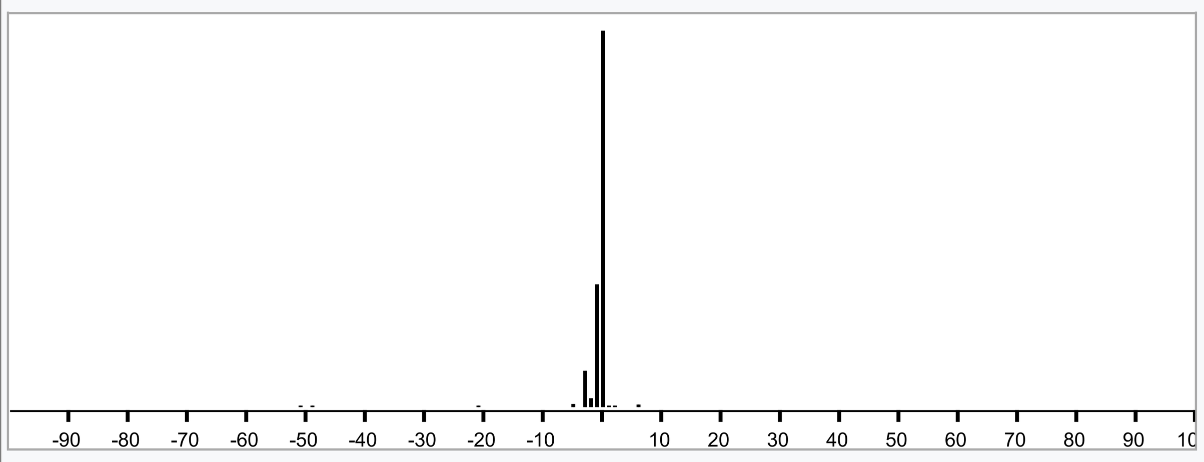

TP53
JASPAR RARA/RXRG MOTIF

Please help us make EPD better! Take our short survey to suggest new tools or organism covered. It will take only a few minutes.

General Information

[Help]

Promoter ID:	TP53_1
Promoter type:	region
Organism:	Homo sapiens (Human)
Gene Symbol:	TP53
Description of the gene:	tumor protein p53
Sequence:	tttcccctcccatgtgctcaagactggcgctaaaagttttgagcttctcAAAAGTCTAGA
Position in the genome:	Chromosome [NC_000017.11]; Strand [-]; Position [7687487]
Ensembl:	ENSG00000141510
RefSeq:	NM_001276696
NCBI Gene:	TP53
GeneCards:	TP53

Promoter Image [View it at UCSC]

[Help]

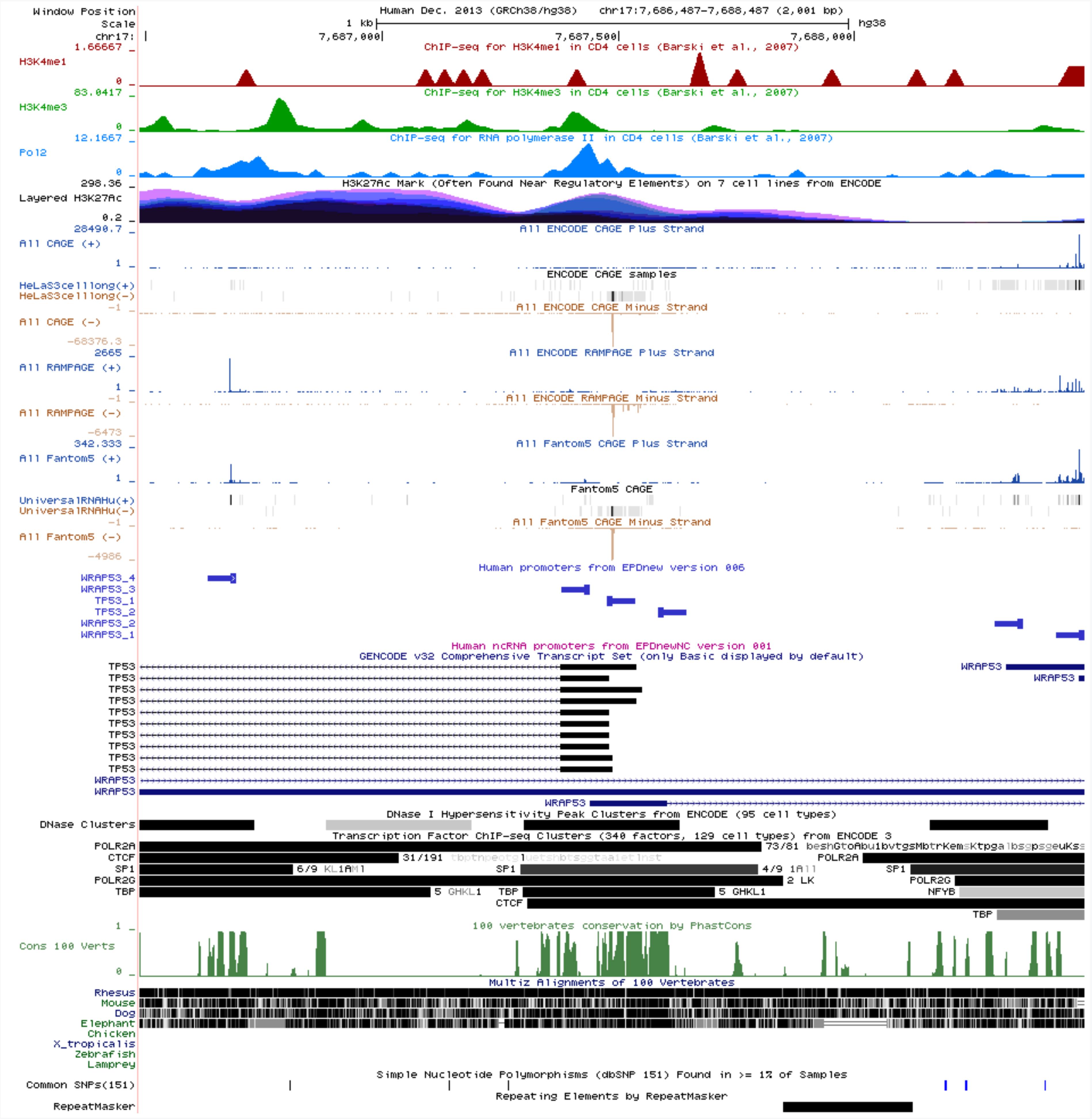

Other viewers

[Help]

EPD view at UCSC (hg38):	chr17:7685487–7689487 (European mirror)
EPD view at UCSC (hg19):	chr17:7588805–7592805 (European mirror)
SwissRegulon (hg19):	chr17:7588805–7592805
FANTOM5 (hg38):	chr17:7685487–7689487
DBTSS/KERO (hg38):	chr17:7685487–7689487
refTSS (hg38):	chr17:7685487–7689487 (European mirror)

Selective viewer showing the 3 Fantom5 tissues with the highest expression of **TP53_1** (European mirror)

GM12878 viewer (European mirror)

Sequence Retrieval Tool

[Help]

Get sequence TP53_1 **from** -1000 **to** 100 bp relative to TSS GET SEQUENCE

lower case upstream TSS

```
>FP022657 TP53_1 :+U EU:NC; range -1000 to 100.
TTGAAAGCACTGTGTTCCTTAGCACCGCGGGTCGCTACGGGCCTCTTGCTGTCGCGGGAT
TTCGGTCCACCTTCCGATTGGGCCGCCGCATCCCGGATCAGATTTCGCGGGCGACCCACG
GAACCCGCGGAGCCGGGACGTGAAAGGTTAGAAGGTTTCCCGTTCCCATCAAGCCCTAGG
GCTCCTCGTGGCTGCTGGGAGTTGTAGTCTGAACGCTTCTATCTTGGCGAGAAGCGCCTA
CGCTCCCCCTACCGAGTCCCGCGGTAATTCTTAAAGCACCTGCACCGCCCCCCCGCCGCC
TGCAGAGGGCGCAGCAGGTCTTGCACCTCTTCTGCATCTCATTCTCCAGGCTTCAGACCT
GTCTCCCTCATTCAAAAAATATTTATTATCGAGCTCTTACTTGCTACCCAGCACTGATAT
AGGCACTCAGGAATACAACAATGAATAAGATAGTAGAAAAATTCTATATCCTCATAAGGC
TTACGTTTCCATGTACTGAAAGCAATGAACAAATAAATCTTATCAGAGTGATAAGGGTTG
TGAAGGAGATTAAATAAGATGGTGTGATATAAAGTATCTGGGAGAAAACGTTAGGGTGTG
ATATTACGGAAAGCCTTCCTAAAAAATGACATTTTAACTGATGAGAAGAAAGGATCCAGC
TGAGAGCAAACGCAAAAGCTTTCTTCCTTCCACCCTTCATATTTGACACAATGCAGGATT
CCTCCAAAATGATTTCCACCAATTCTGCCCTCACAGCTCTGGCTTGCAGAATTTTCCACC
CCAAAATGTTAGTATCTACGGCACCAGGTCGGCGAGAATCCTGACTCTGCACCCTCCTCC
CCAACTCCATTTCCTTTGCTTCCTCCGGCAGGCGGATTACTTGCCCTTACTTGTCATGGC
GACTGTCCAGCTTTGTGCCAGGAGCCTCGCAGGGGTTGATGGGATTGGGGTTTTCCCCTC
CCATGTGCTCAAGACTGGCGCTAAAAGTTTTGAGCTTCTCAAAAGTCTAGAGCCACCGTC
CAGGGAGCAGGTAGCTGCTGGGCTCCGGGGACACTTTGCGTTCGGGCTGGGAGCGTGCTT
TCCACGACGGTGACACGCTTC
```

Search Motif Tool

[Help]

Library: Transcription Factor Motifs (JASPAR Motif: RARA::RXRG

From: -1000 To: 100 bp relative to TSS and a cut-off (p-value) of: 0.001 SEARCH

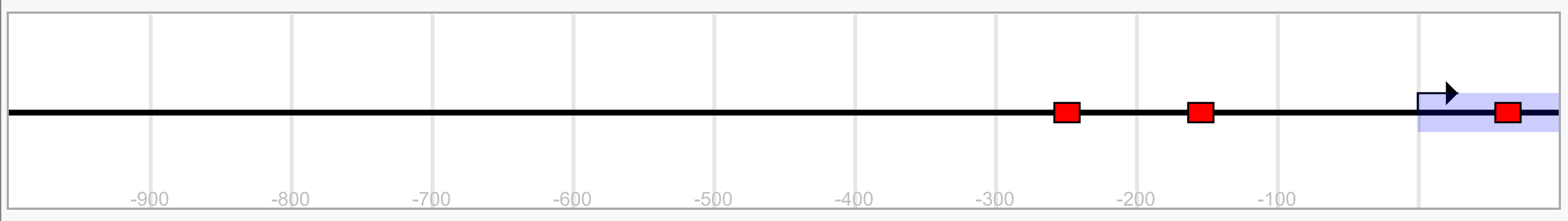

RARA::RXRG [p-value = 0.001]: 55, -163, -258

Expression Profile Tool

[Help]

TP53_1 is expressed in **1037 samples** with an **average expression of 277.229 tags** per 10M.

The TSSs distribution around the EPDnew annotated start site (click on the histogram bars to explore samples):

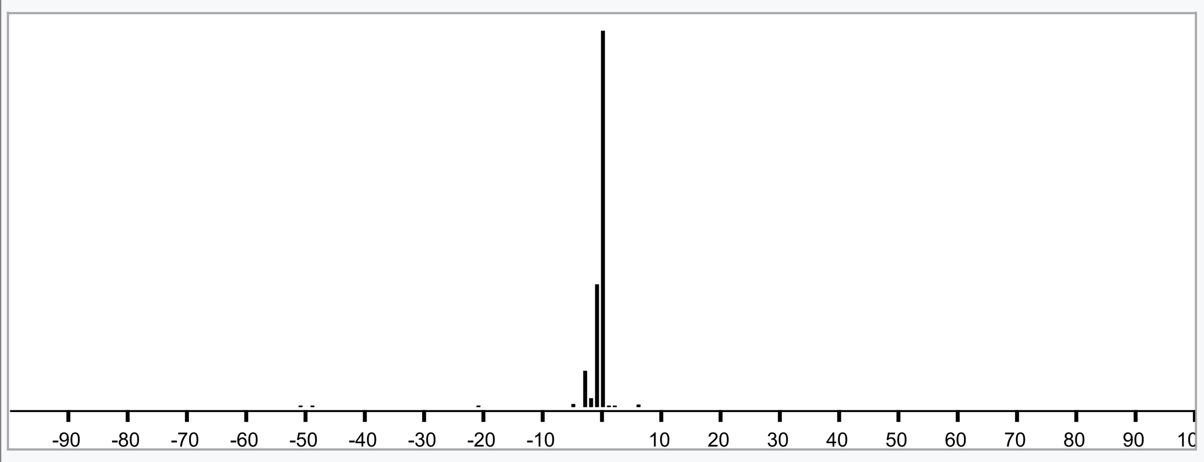

TP53
PATCH RARE MOTIFS

Thank you for using Patch at Host - It's Fri, 16.4.2021 - 12:52 MEZ

Start a new search

Name of your search:	default
Name of your sequence:	default.seq
Sites searched for:	all
Maximum number of mismatches:	2
Mismatch penalty:	100
Lower score boundary:	87.5

Scanning sequence: FP022657 TP53_1 :+U EU:NC; range -1000 to 100.
View a graphic of the following search results

Identifier	Position	Mismatches	Score	Binding Factor	Sequence (Search Pattern)
HS$CDC2_10	1 (+)	0	100.00		TTGAA
HS$CYCA_06	1 (+)	0	100.00		TTGAA
HS$CDC25C_05	1 (+)	0	100.00		TTGAA
HS$CYP3A4_05	1 (+)	1	87.50		TTGAAATCA
AS$PAX2_59	1 (+)	1	87.50	Pax-2.1, Pax-2.2	TTGAAAGAA
AS$ZIC2_25	5 (-)	1	87.50	Zic2	ACGGTGCTT
RAT$A1I3_04	6 (+)	0	100.00		AGCAC
RAT$A1I3_04	6 (-)	0	100.00		GTGCT
AS$mEMBP_04	8 (+)	0	100.00	EmBP-1a	CACTG
AS$MRC_01	8 (-)	0	100.00	Rc	CACAGTG
AS$SRY_10	8 (-)	1	88.89	SRY	AAACACAGTG
AS$PAX2_44	12 (-)	1	87.50	Pax-2.1, Pax-2.2	AATGAACAC
AS$PR_03	13 (+)	0	100.00	PR B	TGTTCC
AS$TR_07	14 (+)	0	100.00	T3R-alpha	GTTCC
AS$TR_10	14 (+)	0	100.00	T3R-alpha	GTTCC
HS$IL4_02	14 (-)	0	100.00		GGAAC
HS$CDC2_02	15 (+)	0	100.00	c-Ets-2	TTCCTT
DROME$E74_10	15 (-)	0	100.00	E74A	AGGAA
DROME$E74_11	15 (-)	0	100.00	E74A	AGGAA
E74A$CONS_01	15 (-)	0	90.00	E74A	MGGAA
HS$CDC2_01	15 (-)	0	100.00	c-Ets-2	AAGGAA
AS$STAT5A_55	16 (-)	1	87.50	STAT5A	TTCTAAGGA
DROME$EVE_01	19 (+)	1	87.50	Eve, Zen-1, Zen-2	TCAGCACCG
DROME$EVE_32	19 (+)	1	87.50	Eve, Ftz, Pax-1, Pax-2, Pax-2a, Pax-2b, Pax-3, Pax-9, Prd, Zen-1, Zen-2	TCAGCACCG
DROME$EVE_32	19 (+)	0	100.00	Eve, Ftz, Pax-1, Pax-2, Pax-2a, Pax-2b, Pax-3, Pax-9, Prd, Zen-1, Zen-2	TTAGCACCG
MESAT$PRP2_04	20 (-)	1	87.50	Alfin1	GCGGTGCTG
RAT$A1I3_04	21 (+)	0	100.00		AGCAC
RAT$A1I3_04	21 (-)	0	100.00		GTGCT
HS$CYCD1_14	25 (+)	0	100.00	Sp1, Sp2, Sp3, Sp4	CCGCG
HS$CDC2_09	25 (-)	0	100.00		CGCGG
HS$CYCA_05	25 (-)	0	100.00		CGCGG
HS$CDC2_09	26 (+)	0	100.00		CGCGG
HS$CYCA_05	26 (+)	0	100.00		CGCGG
HS$CYCD1_14	26 (-)	0	100.00	Sp1, Sp2, Sp3, Sp4	CCGCG
AS$RAR_21	29 (+)	0	100.00	RAR-alpha1, RXR-alpha	GGGTCG
HS$AACS_02	29 (+)	0	100.00	ARP-1	GGGTCG
AS$RAR_20	29 (-)	0	100.00	RAR-alpha1, RXR-alpha	CGACCC
MAIZE$ADH2_03	35 (+)	0	100.00		CTACGG
SIF$CONS	36 (-)	0	91.67	SIF	CCCGTM
HS$CYCD1_15	38 (-)	0	100.00	Sp1, Sp2, Sp3, Sp4	GCCCG
SV$SV40_01	41 (+)	0	100.00	T-Ag	GCCTC
SV$SV40_02	41 (+)	0	100.00	T-Ag	GCCTC
SV$SV40_63	41 (+)	0	100.00	T-Ag	GCCTC
PA$PY_24	41 (-)	0	100.00	T-Ag	GAGGC
PA$PY_25	41 (-)	0	100.00	T-Ag	GAGGC
SV$SV40_02	41 (-)	0	100.00	T-Ag	GAGGC
PA$PY_12	42 (-)	0	100.00		AGAGG
AMV$AMV_02	45 (+)	0	100.00	C/EBPalpha	CTTGC
RAV0$RAV0_01	45 (-)	0	100.00	C/EBPalpha	GCAAG
MOUSE$THY1_06	46 (-)	0	100.00		CAGCAA
MOUSE$M2EAK_03	47 (-)	0	100.00	NF-Y	CAGCA
HS$GG_12	49 (+)	0	100.00	NF-E	CTGTC
DROME$EVE_10	49 (+)	0	100.00	GAGA factor	CTGTC
HS$CYCA_11	52 (+)	0	100.00	E2F+p107	TCGCGG
HS$CDC2_09	53 (+)	0	100.00		CGCGG
HS$CYCA_05	53 (+)	0	100.00		CGCGG
HS$CYCD1_14	53 (-)	0	100.00	Sp1, Sp2, Sp3, Sp4	CCGCG
AS$ZIC3_13	55 (+)	1	87.50	Zic3	CGGGAGTTC
DROME$HB_01	56 (-)	1	87.50	Bcd, Prd	CGTAATCCC
HS$HH4_01	58 (+)	0	100.00	H4TF-1	GATTTC
Y$MEL1_01	61 (+)	0	100.00	GAL4	TTCGG
Y$MEL1_01	61 (-)	0	100.00	GAL4	CCGAA
HS$HH4_02	64 (+)	0	100.00	H4TF-2	GGTCC
AS$TR_11	64 (+)	0	100.00	T3R-alpha	GGTCC
EBV$IR4_05	65 (+)	0	100.00	R	GTCCAC
EBV$IR4_06	65 (+)	0	100.00	R	GTCCAC
PARS$PAL_01	66 (+)	0	100.00		TCCACCT
HT1$HTLV1_08	67 (+)	0	100.00	c-Ets-1 68	CCACC
HS$BG_52	67 (+)	0	100.00		CCACC
HS$BG_14	67 (+)	0	100.00		CCACC
HS$BG_44	67 (+)	0	100.00	CAC-binding protein	CCACC
BPV1$BPV1_24	67 (-)	0	100.00	Sp1	GGTGG
HS$BG_48	67 (-)	0	100.00	CAC-binding protein	GGTGG
MOUSE$CEBPA_06	67 (-)	0	100.00	Sp1	GGTGG
AS$NRF2_07	69 (-)	1	88.89	NRF-2	ACCGGAAGGT
XENLA$RPL14_01	71 (+)	0	100.00	HrpF, XrpFI	CTTCC
Y$TPI_01	71 (+)	0	100.00	GCR1	CTTCC
MOUSE$RPL32_01	71 (-)	0	100.00	f(alpha)-f(epsilon), HrpF, XrpFI	GGAAG
HS$TNFA_02	71 (-)	0	100.00		GGAAG
HS$TNFA_03	71 (-)	0	100.00		GGAAG
HS$BAC_04	72 (+)	0	100.00	Elk-1, SAP-1a, SAP-1b, SRF	TTCCGA
MOUSE$THY1_03	72 (+)	1	88.89	CP1	CTCCGATTGG
E74A$CONS_01	72 (-)	0	90.00	E74A	MGGAA
Y$MLS1_03	75 (+)	1	90.00	CAT8	CCATTGGGCCG
HS$HH1_02	76 (+)	0	100.00	H1TF1, HiNF-B	GATTGG
HS$HSP70_06	76 (+)	0	100.00	CCAAT-binding factor	GATTGG
HS$HSP70_09	76 (+)	0	100.00	CP1, CTF	GATTGG
MOUSE$A11COL_01	77 (+)	0	100.00	CBF (2), IF2	ATTGG
MOUSE$A21COL_03	77 (+)	0	100.00	CCAAT-binding factor	ATTGG
MOUSE$A21COL_04	77 (+)	0	100.00	CBF (2), CBF-A, CBF-B	ATTGG
MOUSE$A21COL_05	77 (+)	0	100.00	EFI	ATTGG
AD2$E2L_03	77 (+)	0	100.00	CRF	ATTGG
HS$GHA_06	77 (+)	0	100.00	NF-1	ATTGG
SP$H2B1_02	77 (+)	0	100.00	CBF (1)	ATTGG
SP$H2B1_03	77 (+)	0	100.00	CDF	ATTGG
HS$HSP70_02	77 (+)	0	100.00	CTF	ATTGG
HS$HSP70_07	77 (+)	0	100.00	CBTF, CP1, CTF	ATTGG
MOUSE$M2EAK_07	77 (+)	0	100.00	NF-Y	ATTGG
MOUSE$M2EAK_08	77 (+)	0	100.00	NF-Y	ATTGG
RSV$RSV_05	77 (+)	0	100.00		ATTGG
HSV1$TK_04	77 (+)	0	100.00	C/EBPalpha	ATTGG
HSV1$TK_05	77 (+)	0	100.00	NF-Y'	ATTGG
AS$HAP23_01	77 (+)	0	100.00	HAP2, HAP3, HAP4	ATTGG
MOUSE$M1H2KB_12	77 (+)	0	100.00	EFI	ATTGG
RAT$TH2A_02	77 (+)	0	100.00		ATTGG
RAT$TH2A_04	77 (+)	0	100.00		ATTGG
HS$TK_02	77 (+)	0	100.00	CP1	ATTGG
MOUSE$A11COL_07	77 (+)	0	100.00	NF-1, Sp1	ATTGG
HS$CDC2_07	77 (+)	0	100.00	CBAF, CBF-B, CP1A	ATTGG
XENLA$GATA2_01	77 (+)	0	100.00	CBTF	ATTGG
RAT$NAT_05	77 (+)	0	100.00		ATTGG
HS$CDC2_12	77 (+)	0	100.00	CBAF	ATTGG
HS$BAC_03	77 (-)	0	100.00	SRF	CCAAT
AMULV$AMULV_01	77 (-)	0	100.00		CCAAT
EBV$DSL_06	77 (-)	0	100.00		CCAAT
MOUSE$AAG_04	77 (-)	0	100.00	alpha-CP1, alpha-CP2a, alpha-CP2b, alpha-IRP	CCAAT
CHICK$BAG_06	77 (-)	0	100.00	TGGCA-binding protein	CCAAT
HS$GG_15	77 (-)	0	100.00	NF-E	CCAAT
HS$GG_16	77 (-)	0	100.00	CDP2, Clox, CUTL1, Cutl1	CCAAT
HS$GG_17	77 (-)	0	100.00	gammaCAAT	CCAAT
HS$GG_18	77 (-)	0	100.00	CP1	CCAAT
HS$ZG_07	77 (-)	0	100.00	CP2	CCAAT
HS$ZG_08	77 (-)	0	100.00	CP1	CCAAT
HS$HH1_03	77 (-)	0	100.00	H1TF2	CCAAT
MOUSE$M2IAB_01	77 (-)	0	100.00	PU.1	CCAAT
PIG$UPA_12	77 (-)	0	100.00	NF-1	CCAAT
RADLV$RLV_10	77 (-)	0	100.00		CCAAT
MULV$MULV_03	77 (-)	0	100.00		CCAAT
MOUSE$GLUT4_04	77 (-)	0	100.00		CCAAT
MOMLV$MOMULV_15	77 (-)	0	100.00		CCAAT
RAT$ALDB_02	77 (-)	0	100.00	CBF (2), CP2	CCAAT
HS$GG_20	77 (-)	0	100.00	CP1, NF-E3	CCAAT
RAT$TH2B_01	77 (-)	0	100.00		CCAAT
RAT$TH2B_02	77 (-)	0	100.00		CCAAT
HS$GG_21	77 (-)	0	100.00	CP1	CCAAT
MOUSE$LY6E_01	77 (-)	0	100.00		CCAAT
RAT$NEU_01	77 (-)	0	100.00		CCAAT
HS$GHA_10	77 (-)	0	100.00	alpha-CBF	CCAAT
NEUCR$CPC1_02	77 (-)	0	100.00		CCAAT
HS$EG_07	77 (-)	0	100.00	CP1	CCAAT
HS$GP2B_13	77 (-)	0	100.00		CCAAT
MOUSE$BMG_10	77 (-)	0	100.00		CCAAT
HS$CYBH_01	77 (-)	0	100.00	CDP2, Clox, CP1, CUTL1, Cutl1	CCAAT
HBV$S_04	77 (-)	0	100.00		CCAAT
HS$FN_06	77 (-)	0	100.00		CCAAT
MOUSE$E2F1_02	77 (-)	0	100.00		CCAAT
HS$A24COL_01	77 (-)	0	100.00		CCAAT
MOUSE$EKLF_01	77 (-)	0	100.00	AP-1, GATA-1	CCAAT
SV$SV40_63	79 (+)	0	100.00	T-Ag	TGGGC
AS$SPZ1_18	80 (+)	1	87.50	Spz1	GGGGCGCCG
MOUSE$JUND_07	81 (-)	1	87.50		GCGGGGGCC
RAT$IGF1R_03	81 (-)	1	87.50	WT1 -KTS, WT1 I -KTS	GCGGGGGCC
RAT$IGF1R_08	81 (-)	1	87.50	WT1 -KTS, WT1 I -KTS	GCGGGGGCC
RAT$IGF1R_09	81 (-)	1	87.50	WT1 -KTS, WT1 I -KTS	GCGGCGGCG
HS$CAVEOLIN1_03	81 (-)	0	100.00	Sp1	GCGGCC
MOUSE$CEBPA_02	82 (+)	0	100.00	CUP	GCCGCCG
AS$GCCBOX_01	82 (+)	0	100.00	ERF2, ERF3, ERF4	GCCGCC
AS$GCCBOX_02	82 (+)	0	100.00	Pti4	GCCGCC
CHICK$BLCR_02	82 (-)	0	100.00	NF-E4, Sp1	GCGGC
AS$ZIC_16	82 (-)	1	87.50	Zic1	TGCGGGGGC
HS$CDC25C_04	83 (-)	0	100.00		GGCGG
CHICK$BLCR_02	85 (-)	0	100.00	NF-E4, Sp1	GCGGC
HS$TCRA_08	86 (+)	0	100.00	c-Ets-1, LEF-1, PEBP2alphaA1	CCGCA
Y$ENO2_03	89 (+)	0	100.00	GCR1	CATCC
Y$TPI_03	89 (+)	0	100.00	GCR1	CATCC
HS$MMP3_01	89 (-)	0	100.00	NIP, PEA3	GGATG
AS$STAT5A_39	92 (+)	1	87.50	STAT5A	CCCGGAGCA
AS$ELK1_07	93 (+)	0	100.00	Elk-1, SAP-1a, SAP-1b, SRF	CCGGAT
HS$GHA_07	95 (-)	0	100.00	GR, GR-alpha, GR-beta	TGATCC
MOUSE$TTPA_01	96 (-)	0	100.00	AP-1	TGATC
CHICK$VIT2_13	97 (+)	0	100.00		ATCAGA
MOUSE$BMG_04	100 (-)	0	100.00	GATA-1	AATCT
HS$HH4_01	101 (+)	0	100.00	H4TF-1	GATTTC
MOUSE$TK_02	102 (+)	1	88.89	Yi	AGTTCGCGGG
AD5$E1A_04	103 (+)	0	100.00	E2F	TTTCGCG
AD5$E1A_05	103 (+)	0	100.00	E2F, E2F+E4	TTTCGCG
AD$E2AE1_24	103 (+)	0	100.00	E2F, E4	TTTCGCG
HS$E2F2_04	103 (+)	0	100.00	E2F	TTTCGCGG
MOUSE$POLA2_02	103 (+)	1	87.50	E2F-1	TTTGGCGGG
HS$CAVEOLIN1_02	103 (+)	1	90.00	DP-1, E2F	TTTGGCGGGCG
Y$CLN2_01	103 (-)	0	100.00	CCBF, SWI4, SWI6	CGCGAAA
AD$E2AE1_24	103 (-)	0	100.00	E2F, E4	CGCGAAA
HS$CDC25A_02	103 (-)	0	100.00	E2F-1	CCGCGAAA
MOUSE$JUND_09	105 (+)	1	87.50	Sp1	GCGCGGGCG
RAT$IGF1R_11	105 (+)	1	87.50	WT1 -KTS, WT1 I -KTS	TCGGGGGCG
HS$CYCA_11	105 (+)	0	100.00	E2F+p107	TCGCGG
RAT$IGF1R_05	105 (-)	1	87.50	WT1 -KTS, WT1 I -KTS	CGCCCGCGC
HS$CDC2_09	106 (+)	0	100.00		CGCGG
HS$CYCA_05	106 (+)	0	100.00		CGCGG
HS$CYCD1_14	106 (-)	0	100.00	Sp1, Sp2, Sp3, Sp4	CCGCG
AS$ZIC3_10	108 (+)	1	87.50	Zic3	CGGGTGACC
MOUSE$SREBP1C_02	108 (-)	0	100.00	LXR-alpha, LXR-beta, RXR-alpha	CGCCCG
MOUSE$GSC_01	108 (-)	1	87.50	SMAD-4	GGCCGCCCG
HS$CYCD1_15	108 (-)	0	100.00	Sp1, Sp2, Sp3, Sp4	GCCCG
HS$CD11B_01	109 (-)	0	100.00	Sp1	CGCCC
CHICK$AAC_06	110 (+)	0	100.00	delta factor, F-ACT1, MAPF2, YY1	GGCGAC
CHICK$AAC_07	110 (+)	0	100.00	YY1	GGCGAC
AS$RAR_20	112 (+)	0	100.00	RAR-alpha1, RXR-alpha	CGACCC
AS$RAR_21	112 (-)	0	100.00	RAR-alpha1, RXR-alpha	GGGTCG
HS$AACS_02	112 (-)	0	100.00	ARP-1	GGGTCG
HS$GG_40	113 (-)	0	100.00		TGGGTC
AS$STAT5A_49	115 (-)	1	87.50	STAT5A	TTCCGTGTG
AS$STAT5A_52	115 (-)	1	87.50	STAT5A	TTCCGTGGA
MAIZE$ADH11S_02	116 (+)	0	100.00		CCACGG
MAIZE$ADH1P_01	116 (-)	0	100.00		CGTGG
MAIZE$ADH1P_03	116 (-)	0	100.00		CGTGG
AS$MSX1_11	117 (-)	1	87.50	Msx-1	CGTTCCGTG
AS$ELK1_09	118 (-)	0	100.00	Elk-1, SAP-1a, SAP-1b, SRF	TTCCGT
E74A$CONS_01	119 (+)	0	90.00	E74A	MGGAA
Y$BAP2_01	119 (+)	1	87.50	LEU3	CGGAACCGG
HS$IL4_02	120 (+)	0	100.00		GGAAC
AS$TR_07	120 (-)	0	100.00	T3R-alpha	GTTCC
AS$TR_10	120 (-)	0	100.00	T3R-alpha	GTTCC
Y$PDR15_01	123 (-)	1	87.50	PDR1	TCCGCGGGA
HS$CYCD1_14	125 (+)	0	100.00	Sp1, Sp2, Sp3, Sp4	CCGCG
HS$CDC2_09	125 (-)	0	100.00		CGCGG
HS$CYCA_05	125 (-)	0	100.00		CGCGG
HS$CDC2_09	126 (+)	0	100.00		CGCGG
HS$CYCA_05	126 (+)	0	100.00		CGCGG
HS$CYCD1_14	126 (-)	0	100.00	Sp1, Sp2, Sp3, Sp4	CCGCG
Y$CHA1_01	127 (+)	0	100.00	CHA4	GCGGA
Y$CHA1_01	127 (-)	0	100.00	CHA4	TCCGC
Y$BAP2_01	128 (+)	1	87.50	LEU3	CGGAACCGG
RAT$MLC_04	129 (+)	0	100.00	MAPF2, YY1	GGAGC
HS$GMCSF_10	129 (+)	0	100.00	NF-ATp	GGAGCC
EBV$IR4_04	135 (+)	0	100.00	R	GGGAC
EBV$IR4_05	135 (+)	0	100.00	R	GGGAC
HS$MHC2IN_01	136 (-)	1	88.89		TTTCACTTCC
HS$ENO1_01	137 (+)	0	100.00	HIF-1	GACGTG
AT$GST6_02	137 (+)	0	100.00	OBF4	GACGTG
RICE$EM_01	138 (+)	0	100.00	OSBZ8, TRAB1	ACGTG
RICE$EM_02	138 (+)	0	100.00	OSBZ8	ACGTG
AS$mEMBP_15	138 (-)	0	100.00	EmBP-1a	CACGT
Y$CHO1_01	138 (-)	1	87.50		CTTTCACAT
AS$AREB6_07	143 (-)	1	87.50	AREB6	CTAACCTGT
HS$IBABP_01	145 (-)	0	100.00	FXR, RXR-alpha	TAACCT
MOUSE$IBABP_01	145 (-)	0	100.00	FXR, RXR-alpha	TAACCT
HS$ALBU_01	146 (+)	0	100.00	HNF-1, HNF-1A, HNF-1B, HNF-1C	GGTTAG
I$HSF_01	150 (+)	0	90.00	HSF	AGAAN
F$HSF_01	150 (+)	0	90.00	HSF	AGAAN
AS$AREB6_44	155 (+)	0	100.00	AREB6	GTTTC
AS$AREB6_46	155 (+)	0	100.00	AREB6	GTTTC
AS$AREB6_48	155 (+)	0	100.00	AREB6	GTTTC
AS$AREB6_49	155 (+)	0	100.00	AREB6	GTTTC
AS$AREB6_50	155 (+)	0	100.00	AREB6	GTTTC
AS$AREB6_52	155 (+)	0	100.00	AREB6	GTTTC
AS$AREB6_53	155 (+)	0	100.00	AREB6	GTTTC
HS$IFI616_01	156 (-)	0	100.00	ISGF-3	GGAAA
MOUSE$IL5_02	156 (-)	0	100.00	NF-ATc, NF-ATp, NF-ATx	GGAAA
HS$IL4_01	156 (-)	0	100.00		GGAAA
MOUSE$IL4_06	156 (-)	0	100.00		GGAAA
MOUSE$IL4_07	156 (-)	0	100.00		GGAAA
MOUSE$IL4_08	156 (-)	0	100.00		GGAAA
HS$IL5_02	156 (-)	0	100.00		GGAAA
HS$IL13_01	156 (-)	0	100.00		GGAAA
MOUSE$CD40L_01	156 (-)	0	100.00		GGAAA
MOUSE$CD40L_02	156 (-)	0	100.00		GGAAA
MOUSE$CD40L_03	156 (-)	0	100.00		GGAAA
MOUSE$CD40L_04	156 (-)	0	100.00		GGAAA
HS$CD40L_01	156 (-)	0	100.00		GGAAA
HS$CD40L_02	156 (-)	0	100.00		GGAAA
HS$IFNG_03	156 (-)	0	100.00		GGAAA
HS$IFNG_04	156 (-)	0	100.00		GGAAA
HS$TNFA_05	156 (-)	0	100.00		GGAAA
MOUSE$MCP3_01	156 (-)	0	100.00		GGAAA
HS$TNFSF6_01	156 (-)	0	100.00		GGAAA
AS$STAT5A_54	157 (-)	1	87.50	STAT5A	TAACGGGAA
AS$TR_07	162 (+)	0	100.00	T3R-alpha	GTTCC
AS$TR_10	162 (+)	0	100.00	T3R-alpha	GTTCC
HS$IL4_02	162 (-)	0	100.00		GGAAC
AS$STAT_04	163 (+)	1	87.50	STAT6	TTCCCGTCA
AS$IK_06	163 (-)	0	100.00	Ik-1, Ik-2	TGGGAA
HS$CDH1_01	164 (+)	0	100.00	LUN-1	TCCCA
HS$TLN_01	164 (+)	0	100.00	LUN-1	TCCCA
MOUSE$AMY_07	164 (-)	0	100.00	PTF1-beta	ATGGGA
HS$CDH1_01	164 (-)	0	100.00	LUN-1	TGGGA
HS$TLN_01	164 (-)	0	100.00	LUN-1	TGGGA
AS$LIM1_06	166 (+)	1	90.91	LIM1	CCATCAAGCTCT
LPV$LPV_03	168 (+)	0	100.00	Pu box binding factor	ATCAA
AS$ZIC3_23	179 (+)	1	87.50	Zic3	GGGGTCCTC
HS$GMCSF_10	180 (-)	0	100.00	NF-ATp	GGAGCC
RAT$MLC_04	181 (-)	0	100.00	MAPF2, YY1	GGAGC
PARS$CHS_02	183 (+)	1	88.89	CG-1, CPRF-1, CPRF-2, CPRF-4a, CPRF-4b	TCCACGTGGC
AS$mEMBP_02	184 (-)	1	87.50	EmBP-1a	GCCACGTGG
HSV2$VMW65_01	185 (+)	0	100.00	USF1	CACGAG
MOUSE$HES1_02	185 (+)	0	100.00	HES-1	CACGAG
MOUSE$HES1_03	185 (+)	0	100.00	HES-1	CACGAG
MAIZE$ADH1P_01	187 (+)	0	100.00		CGTGG
MAIZE$ADH1P_03	187 (+)	0	100.00		CGTGG
HIV1$HIV1_26	187 (+)	0	100.00	HMBP	CGTGGC
AS$mEMBP_17	187 (-)	0	100.00	EmBP-1a	GCCACG
AS$ZIC2_10	189 (+)	1	87.50	Zic2	TGGTTGCTG
DROME$ADH_06	191 (+)	0	100.00	Adf-1	GCTGC
HS$CMYC_16	191 (+)	0	100.00	CTCF	GCTGC
DROME$ADH_14	191 (-)	0	100.00	Adf-1	GCAGC
RAT$AC3_02	193 (+)	1	87.50		TGCTGGCAG
MOUSE$M2EAK_03	193 (-)	0	100.00	NF-Y	CAGCA
RAT$OCNC_02	193 (-)	1	87.50		CTGCCAGCA
CHICK$STN1_01	194 (-)	0	100.00	LBP-1, MyoD	CCAGC
CHICK$STN1_03	194 (-)	0	100.00	LBP-1, NF-1 (-like proteins)	CCAGC
SV$SV40_37	195 (+)	0	100.00		CTGGG
RAT$AMGL_02	195 (+)	0	100.00	IL-6 RE-BP, STAT1, STAT5A, STAT5B, STAT6	CTGGGA
HS$APOB_10	196 (+)	0	100.00	AP-2alphaA, AP-2alphaB	TGGGAG
HS$CDH1_01	196 (+)	0	100.00	LUN-1	TGGGA
HS$TLN_01	196 (+)	0	100.00	LUN-1	TGGGA
HS$CDH1_01	196 (-)	0	100.00	LUN-1	TCCCA
HS$TLN_01	196 (-)	0	100.00	LUN-1	TCCCA
AS$SPZ1_28	197 (+)	1	87.50	Spz1	GGGCGTTGT
AS$SPZ1_30	197 (+)	1	87.50	Spz1	GGGCGTTGT
MOUSE$HOXA5_01	197 (-)	0	100.00	HOXA5	ACTCCC
AS$CF1_37	198 (+)	1	87.50	CF1	GGGGTTGTA
HS$PR264_06	200 (-)	0	100.00	c-Myb	CAACT
ALV$LTR_02	203 (+)	1	87.50	EFII	TGTAGTCTT
AS$STAT5A_51	205 (-)	1	87.50	STAT5A	TTCCGACTA
AS$STAT5A_57	205 (-)	1	87.50	STAT5A	TTCCGACTA
HS$ADH3_01	210 (+)	0	100.00	CAR, RAR-alpha1, RAR-beta, RXR-alpha	TGAAC
AS$MEIS1AHOXA9_12	210 (+)	0	100.00	HOXA9, Meis-1a	TGAACG
AS$RAR_05	210 (-)	0	100.00	RAR-alpha1	GTTCA
AS$RAR_06	210 (-)	0	100.00	RAR-alpha1	GTTCA
AS$TR_06	210 (-)	0	100.00	T3R-alpha	GTTCA
AS$TR_07	210 (-)	0	100.00	T3R-alpha	GTTCA
AS$TR_08	210 (-)	0	100.00	T3R-alpha	GTTCA
AS$TR_11	210 (-)	0	100.00	T3R-alpha	GTTCA
MOUSE$RARA2_01	210 (-)	0	100.00	RAR-alpha1, RAR-beta2, RAR-gamma1	GTTCA
I$HSF_01	216 (-)	0	90.00	HSF	AGAAN
F$HSF_01	216 (-)	0	90.00	HSF	AGAAN
HS$BG_42	218 (+)	0	100.00	GATA-1	TCTATC
HS$BG_49	218 (-)	0	100.00	GATA-1	GATAGA
HS$GG_08	219 (+)	0	100.00	NF-E	CTATC
HS$ZG_02	219 (+)	0	100.00	GATA-1	CTATCT
HS$BG_51	219 (+)	0	100.00	GATA-1	CTATCT
MOUSE$IL5_06	219 (+)	0	100.00	GATA-3	CTATCT
HS$CD8A_01	219 (+)	0	100.00	GATA-3	CTATCT
MOUSE$TCRBL_07	219 (+)	0	100.00	GATA-3	CTATCT
HS$AG_03	219 (-)	0	100.00	GATA-1	AGATAG
HS$BG_47	219 (-)	0	100.00	GATA-1	AGATAG
MOUSE$GSHPX1_06	220 (+)	0	100.00	GATA-1	TATCT
MOUSE$GSHPX1_07	220 (+)	0	100.00	GATA-1	TATCT
MOUSE$GSHPX1_09	220 (+)	0	100.00	GATA-1	TATCT
MOUSE$BMG_02	220 (-)	0	100.00	GATA-1	AGATA
XENLA$ACY_01	221 (+)	0	100.00	SRF	ATCTT
XENLA$ACY_01	221 (-)	0	100.00	SRF	AAGAT
CHICK$OA_01	223 (+)	0	100.00	NF-1 (-like proteins)	CTTGGC
MOUSE$MYB_01	223 (+)	1	87.50	E2F, E2F-1, E2F-3, E2F-4	CTTGGCGGG
HS$NPY_03	223 (-)	0	100.00	AP-1, CCAAT-binding factor	CCAAG
MOUSE$AP2_07	224 (+)	0	100.00	NF-1	TTGGC
MOUSE$BMG_08	224 (-)	0	100.00	NF-1	TCGCCAA
FACT1$CONS	225 (+)	0	100.00	F-ACT1	TGGCGA
AS$STAT5A_44	225 (-)	1	87.50	STAT5A	TTCTCGACA
DROME$EVE_10	227 (-)	0	100.00	GAGA factor	CTCGC
MOUSE$GSHPX1_01	229 (-)	0	100.00	PU.1	CTTCTC
I$HSF_01	230 (+)	0	90.00	HSF	AGAAN
F$HSF_01	230 (+)	0	90.00	HSF	AGAAN
AS$SPZ1_02	230 (-)	1	87.50	Spz1	GGCGCTTAT
AS$SPZ1_22	231 (-)	1	87.50	Spz1	GGGCGCTTC
HS$P53_04	235 (+)	0	100.00	Egr-1	CGCCTACGC
MOUSE$ADA_01	235 (-)	1	87.50	Egr-1	GCGTGGGCG
SV$SV40_63	236 (+)	0	100.00	T-Ag	GCCTA
AS$SPZ1_12	238 (-)	1	87.50	Spz1	GGAGGGTAG
DROME$EVE_04	241 (+)	0	100.00	GAGA factor	CGCTC
DROME$EVE_08	241 (+)	0	100.00	GAGA factor	CGCTC
DROME$EVE_10	241 (+)	0	100.00	GAGA factor	CGCTC
DROME$EVE_11	241 (+)	0	100.00	GAGA factor	CGCTCCC
H4TF1$CONS	241 (-)	1	87.50	H4TF-1	GGGGGAGGG
RAT$MLC_04	242 (-)	0	100.00	MAPF2, YY1	GGAGC
AS$ZIC3_20	242 (-)	1	87.50	Zic3	TGGGGGAGC
RAT$CAT_01	243 (-)	1	87.50		TGGGGGGAG
CHICK$CA2_02	244 (+)	0	100.00	RXR-alpha, VDR	TCCCCC
HS$CYCD1_14	245 (+)	0	100.00	Sp1, Sp2, Sp3, Sp4	CCCCC
HS$CYCD1_15	245 (+)	0	100.00	Sp1, Sp2, Sp3, Sp4	CCCCC
AS$VDR_03	245 (-)	0	100.00	VDR	AGGGGG
AS$VDR_05	245 (-)	0	100.00	VDR	AGGGGG
AS$VDR_07	245 (-)	0	100.00	VDR	AGGGGG
AS$VDR_09	245 (-)	0	100.00	VDR	AGGGGG
AS$ADR1_15	246 (-)	0	100.00	ADR1	GTAGGGG
AS$VDR_04	246 (-)	0	100.00	VDR	AGGGG
Y$GLK1_02	246 (-)	0	100.00	MSN2, MSN4	AGGGG
PV$CHS_05	248 (+)	0	100.00		CCTACC
TO$E4_02	249 (+)	0	100.00		CTACC
HS$EGFR_12	254 (+)	0	100.00	RFP1, Sp1	GAGTCCC
EBV$IR4_04	256 (-)	0	100.00	R	GGGAC
EBV$IR4_05	256 (-)	0	100.00	R	GGGAC
Y$PDR15_01	257 (+)	1	87.50	PDR1	TCCGCGGGA
HS$CYCD1_14	259 (+)	0	100.00	Sp1, Sp2, Sp3, Sp4	CCGCG
HS$CDC2_09	259 (-)	0	100.00		CGCGG
HS$CYCA_05	259 (-)	0	100.00		CGCGG
HS$CDC2_09	260 (+)	0	100.00		CGCGG
HS$CYCA_05	260 (+)	0	100.00		CGCGG
HS$CYCD1_14	260 (-)	0	100.00	Sp1, Sp2, Sp3, Sp4	CCGCG
RICE$PHYA_01	263 (+)	0	100.00	GT-2	GGTAATT
RICE$PHYA_02	263 (+)	0	100.00	GT-2	GGTAATT
RAT$NAT_02	264 (+)	0	100.00	Crx	GTAATTC
AMV$AMV_01	264 (-)	0	100.00	C/EBPalpha	ATTAC
FSV$FSV_01	264 (-)	0	100.00	C/EBPalpha	ATTAC
MOUSE$NCAM_05	265 (+)	1	90.00	Antp, Barx1, HOXA5, Phox2a, Zen-1, Zen-2	TAATTATTAAA
Y$SUC2_02	265 (-)	0	100.00	MIG1	AATTA
RAT$DBH_01	265 (-)	0	100.00	ARIX, c-Fos, c-Jun, CREB, CREMtau	AATTA
I$HSF_01	267 (-)	0	90.00	HSF	AGAAN
F$HSF_01	267 (-)	0	90.00	HSF	AGAAN
AS$TBP_06	269 (-)	0	100.00	TBP	TTTAAGA
AS$PAX2_62	270 (+)	1	87.50	Pax-2.1, Pax-2.2	CTTAAAGCC
Y$MAL63_01	271 (-)	0	100.00	MIG1	TTTAA
RAT$A1I3_04	275 (+)	0	100.00		AGCAC
RAT$A1I3_04	275 (-)	0	100.00		GTGCT
RADLV$RLV_07	276 (+)	0	100.00	Rad-1	GCACCTG
MAIZE$BZ1_03	276 (-)	1	87.50	B-Peru	GGCAGGTGC
AS$ERR1_10	276 (-)	0	100.00	ERR1	AGGTGC
MOUSE$ACRD_02	277 (+)	0	100.00	E12, E47, MyoD, myogenin	CACCTG
MOUSE$MCK_03	277 (+)	0	100.00	ASH-1, ASH-3a, ASH-3b, E12, E47, HEB, ITF-2, MASH-1, MASH-2, MEF1, MRF4, MyoD, myogenin	CACCTGC
XENLA$AC_05	277 (+)	0	100.00	EMF1, MyoD	CACCTG
HS$CXCR4_02	277 (+)	0	100.00	c-Myc, USF2	CACCTG
PV$PHASL_02	277 (+)	0	100.00	CAN	CACCTG
MOUSE$CD4_03	277 (-)	0	100.00	E12, HEB	CAGGTG
DROME$HB_12	277 (-)	0	100.00	Ac, AS-C T3, Da, Sc	CAGGTG
DROME$AC_01	277 (-)	0	100.00	Ac, AS-C T3, Da, Sc	CAGGTG
AS$SNAIL_01	277 (-)	0	100.00	Sn	CAGGTG
AS$LMO2_01	277 (-)	0	100.00	CLIM2, E12, E47, GATA-1, Lmo2, Tal-1	CAGGTG
HS$VEGF_02	278 (-)	0	100.00		CAGGT
MOMLV$MOMULV_09	279 (+)	0	100.00	LVc	CCTGC
MOMLV$MOMULV_10	279 (+)	0	100.00	LVc	CCTGC
RADLV$RLV_03	279 (+)	0	100.00	LVc	CCTGC
MESAT$PRP2_04	280 (-)	1	87.50	Alfin1	GCGGTGCTG
MOUSE$MT1_01	281 (+)	0	100.00	Sp1	TGCAC
HS$A11COL_01	282 (+)	1	88.89	Sp1	GCCCCGCCCC
HS$RAS1_06	282 (+)	1	88.89	Sp1	GCCCCGCCCC
RAT$ME_02	282 (+)	1	88.89	Sp1	GCCCCGCCCC
MOUSE$RPS16_01	282 (+)	1	88.89	Sp1	GCTCCGCCCC
HS$CYCA_01	282 (+)	1	88.89		GCCCCGCCCC
MOUSE$DHFR_01	282 (-)	1	88.89	Sp1	GGGGCGGAGC
AD2$E1B_03	282 (-)	1	88.89	BTEB1, MAZ, Sp1	GGGGCGGGGC
HS$HSP70_10	282 (-)	1	88.89	Sp1	GGGGCGGGGC
HS$RAS1_03	282 (-)	1	88.89	Sp1	GGGGCGGGGC
HS$RAS1_04	282 (-)	1	88.89	Sp1	GGGGCGGGGC
HSV1$TK_03	282 (-)	1	88.89	Sp1	GGGGCGGGGC
AD2$E1B_02	282 (-)	1	88.89	Sp1, TBP, TFIID	GGGGCGGGGC
AS$SP1_02	282 (-)	1	88.89	Sp1	GGGGCGGGGC
HS$DHFR_05	282 (-)	1	88.89	Sp1	GGGGCGGGGC
HS$DHFR_06	282 (-)	1	88.89	Sp1	GGGGCGGGGC
HS$ZG_06	283 (+)	1	87.50	Sp1	CACCACCCC
HS$MT2A_10	283 (-)	1	87.50	Sp1	GGGGCGGGG
RAT$GSTP_09	283 (-)	1	87.50		GGGGCGGGG
MOUSE$PFK_05	283 (-)	1	87.50		GGGGCGGGG
HS$FN_07	283 (-)	1	87.50	Sp1	GGGGCGGGG
AS$ZIC2_27	283 (-)	1	87.50	Zic2	GGGGCGGTG
AS$ZIC3_17	283 (-)	1	87.50	Zic3	GGGGGGGTG
CHICK$ACRA_07	285 (+)	0	100.00	Sp1	CCGCCC
MOUSE$APRT_01	285 (+)	0	100.00	Sp1	CCGCCC
RAT$GF_07	285 (+)	0	100.00	Sp1	CCGCCC
HA$HMGCR_03	285 (+)	0	100.00		CCGCCC
HSV1$IE3_05	285 (+)	0	100.00	Sp1	CCGCCC
HSV1$IE4_04	285 (+)	0	100.00	Sp1	CCGCCC
HS$TGFB1_03	285 (+)	0	100.00	Sp1	CCGCCCCC
EGR2$CONS	285 (+)	1	88.89	EGR2	CCGCCCCCGC
MOUSE$EPOR_02	285 (+)	0	100.00	Sp1	CCGCCC
RAT$CDC2_02	285 (+)	0	100.00		CCGCCC
MOUSE$E2F1_01	285 (+)	0	100.00		CCGCCC
HS$TK_08	285 (+)	0	100.00	Sp1	CCGCCCC
HS$TERT_04	285 (+)	0	100.00	Sp1	CCGCCC
HS$TERT_07	285 (+)	0	100.00	Sp1	CCGCCC
HS$APOE_08	285 (-)	0	100.00	Sp1	GGGCGG
HA$HMGCR_03	285 (-)	0	100.00		GGGCGG
HSV1$IE3_04	285 (-)	0	100.00	Sp1	GGGCGG
HSV1$IE3_07	285 (-)	0	100.00	Sp1	GGGCGG
HSV1$IE3_09	285 (-)	0	100.00	Sp1	GGGCGG
HSV1$IE4_01	285 (-)	0	100.00	Sp1	GGGCGG
HSV1$IE4_03	285 (-)	0	100.00	Sp1	GGGCGG
RAT$MT1_01	285 (-)	0	100.00	Sp1	GGGGGCGG
SV$SV40_06	285 (-)	0	100.00	LSF	GGGCGG
SV$SV40_07	285 (-)	0	100.00	ETF, Sp1	GGGCGG
SV$SV40_13	285 (-)	0	100.00	Sp1	GGGCGG
HS$U2SN_03	285 (-)	0	100.00	Sp1	GGGCGG
HS$U2SN_05	285 (-)	0	100.00	Sp1	GGGCGG
PIG$UPA_10	285 (-)	0	100.00	Sp1	GGGCGG
PIG$UPA_11	285 (-)	0	100.00	Sp1	GGGCGG
PIG$UPA_14	285 (-)	0	100.00	Sp1	GGGCGG
MOUSE$GLUT4_05	285 (-)	0	100.00	Sp1	GGGCGG
AD5$E1A_15	285 (-)	0	100.00	Sp1	GGGCGG
HS$NPY_01	285 (-)	0	100.00	Sp1	GGGCGG
RAT$IGF2_01	285 (-)	0	100.00	Sp1	GGGCGG
HS$TK_01	285 (-)	0	100.00	CP1	GGGCGG
MOUSE$ECADH_02	285 (-)	0	100.00	Sp1	GGGGGCGG
HS$CDC25C_04	285 (-)	0	100.00		GGCGG
HS$A24COL_03	285 (-)	0	100.00	Sp1	GGGCGG
HS$TK_05	285 (-)	0	100.00	Sp1	GGGGGCGG
HS$TK_07	285 (-)	0	100.00	Sp1	GGGCGG
RAT$HSC70_01	285 (-)	0	100.00	Sp1, Sp3	GGGGCGG
WTZFP$CONS	286 (+)	1	87.50	WT1, WT1 -KTS, WT1 I, WT1 I -KTS, WT1 I-del2, WT1-del2	CGCCCCCGC
HS$IGF2_01	286 (+)	1	87.50	WT1	CGCCCCCGC
HS$IGF2_03	286 (+)	1	87.50	WT1	CGCCCCCGC
YL$XPR2_03	286 (+)	0	100.00		CGCCCCC
HS$CD11B_01	286 (+)	0	100.00	Sp1	CGCCC
MOUSE$HOX14_01	286 (-)	1	87.50	Krox-20, Krox-24	GCGGGGGCG
HS$IGF2_02	286 (-)	1	87.50	WT1	GCGGGGGCG
AS$NGFIC_01	286 (-)	1	87.50	NGFI-C	GCGGGGGCG
MOUSE$JUND_05	286 (-)	1	87.50	Sp1	GCGGGGGCG
LP$LPS1B_02	286 (-)	1	87.50	IF1	TGGGGGGCG
RAT$IGF1R_01	286 (-)	1	87.50	WT1, WT1 -KTS, WT1 I, WT1 I -KTS	GTGGGGGCG
RAT$IGF1R_04	286 (-)	1	87.50	WT1, WT1 -KTS, WT1 I, WT1 I -KTS	GCGGGGGCG
AS$SPZ1_08	286 (-)	1	87.50	Spz1	GGGGGGTCG
AS$ZIC3_17	286 (-)	1	87.50	Zic3	GGGGGGGTG
PA$PY_26	287 (+)	0	100.00	T-Ag	GCCCC
MAIZE$ADH11S_06	287 (+)	0	100.00	GCBP-1, Sp1	GCCCC
PA$PY_23	287 (-)	0	100.00	T-Ag	GGGGC
SV$SV40_04	287 (-)	0	100.00	T-Ag	GGGGC
SV$SV40_63	287 (-)	0	100.00	T-Ag	GGGGC
AS$ZIC2_07	287 (-)	1	87.50	Zic2	TGGGGGGGC
AS$ZIC2_09	287 (-)	1	87.50	Zic2	GGGGGGGGC
AS$ZIC3_05	287 (-)	1	87.50	Zic3	CGGGGGGTC
WTZFP$CONS	288 (+)	1	87.50	WT1, WT1 -KTS, WT1 I, WT1 I -KTS, WT1 I-del2, WT1-del2	CGCCCCCGC
HS$IGF2_01	288 (+)	1	87.50	WT1	CGCCCCCGC
HS$IGF2_03	288 (+)	1	87.50	WT1	CGCCCCCGC
HS$CYCD1_14	288 (+)	0	100.00	Sp1, Sp2, Sp3, Sp4	CCCCC
HS$CYCD1_15	288 (+)	0	100.00	Sp1, Sp2, Sp3, Sp4	CCCCC
MOUSE$HOX14_01	288 (-)	1	87.50	Krox-20, Krox-24	GCGGGGGCG
AS$BGP1_02	288 (-)	0	100.00	BGP1	GGGGGGG
HS$IGF2_02	288 (-)	1	87.50	WT1	GCGGGGGCG
HS$GAST_02	288 (-)	1	88.89		GCGGGGGTGG
AS$NGFIC_01	288 (-)	1	87.50	NGFI-C	GCGGGGGCG
MOUSE$JUND_05	288 (-)	1	87.50	Sp1	GCGGGGGCG
RAT$NEU_03	288 (-)	1	88.89		GGTGGGGGGG
RAT$IGF1R_04	288 (-)	1	87.50	WT1, WT1 -KTS, WT1 I, WT1 I -KTS	GCGGGGGCG
HS$CYCD1_14	289 (+)	0	100.00	Sp1, Sp2, Sp3, Sp4	CCCCC
HS$CYCD1_15	289 (+)	0	100.00	Sp1, Sp2, Sp3, Sp4	CCCCC
HS$RAS1_05	290 (+)	1	88.89	Sp1	CCCCCGCCCC
HS$CYCD1_14	290 (+)	0	100.00	Sp1, Sp2, Sp3, Sp4	CCCCC
HS$CYCD1_15	290 (+)	0	100.00	Sp1, Sp2, Sp3, Sp4	CCCCC
AS$VDR_06	290 (-)	0	100.00	VDR	CGGGGG
Y$SUC2_01	291 (+)	0	100.00	MIG1	CCCCGC
Y$PDC1_02	291 (+)	0	100.00	MIG1	CCCCGC
HS$TK_09	291 (+)	0	100.00	Sp1	CCCCGCC
HS$MT2A_10	291 (-)	1	87.50	Sp1	GGGGCGGGG
RAT$GSTP_09	291 (-)	1	87.50		GGGGCGGGG
MOUSE$PFK_05	291 (-)	1	87.50		GGGGCGGGG
HS$FN_07	291 (-)	1	87.50	Sp1	GGGGCGGGG
RAT$IGF1R_09	291 (-)	1	87.50	WT1 -KTS, WT1 I -KTS	GCGGCGGCG
HS$TK_06	291 (-)	0	100.00	Sp1	GGCGGGG
HS$P21WAF1_06	292 (+)	0	100.00	SMAD-3, SMAD-4	CCCGCC
HS$P21WAF1_07	292 (+)	0	100.00		CCCGCC
HSV1$IE3_06	292 (-)	0	100.00	Sp1	GGCGGG
RAT$NF1_02	292 (-)	0	100.00	Sp1	GGCGGG
HS$NPY_02	292 (-)	0	100.00	Sp1	GGCGGG
HS$MIP_02	292 (-)	0	100.00	Sp1	GGCGGG
HS$CDC25C_04	293 (-)	0	100.00		GGCGG
AS$GCCBOX_01	295 (+)	0	100.00	ERF2, ERF3, ERF4	GCCGCC
AS$GCCBOX_02	295 (+)	0	100.00	Pti4	GCCGCC
CHICK$BLCR_02	295 (-)	0	100.00	NF-E4, Sp1	GCGGC
HS$TPI_04	296 (-)	0	100.00	Sp1	AGGCGG
HS$CDC25C_04	296 (-)	0	100.00		GGCGG
HS$CJUN_04	297 (+)	1	88.89	AP-2alphaA, AP-2alphaB	CGCCTGCGGA
Y$CTT1_02	297 (-)	1	87.50	repressor of CAR1 expression	CTGCAGGCT
HS$EGFR_19	298 (+)	0	100.00		GCCTGC
MOMLV$MOMULV_09	299 (+)	0	100.00	LVc	CCTGC
MOMLV$MOMULV_10	299 (+)	0	100.00	LVc	CCTGC
RADLV$RLV_03	299 (+)	0	100.00	LVc	CCTGC
RAT$POMC_03	303 (+)	0	100.00	GR	CAGAG
RAT$INS1_10	303 (-)	1	87.50		CGCCATCTG
HS$FREAC4_01	303 (-)	1	87.50	WT1	CGCCCTCTC
PA$PY_12	304 (+)	0	100.00		AGAGG
CTCF$CONS	305 (-)	0	100.00	CTCF	CCCTC
HS$CYP24_02	306 (-)	0	100.00	VDR	CGCCCT
RAT$CYP24_01	306 (-)	0	100.00	VDR	CGCCCT
HS$APP_01	307 (+)	0	100.00		GGGCGC
HS$CD11B_01	307 (-)	0	100.00	Sp1	CGCCC
HS$MT2A_15	307 (-)	0	100.00	MTF-1	TGCGCCC
DROME$ADH_14	311 (+)	0	100.00	Adf-1	GCAGC
DROME$ADH_06	311 (-)	0	100.00	Adf-1	GCTGC
HS$CMYC_16	311 (-)	0	100.00	CTCF	GCTGC
MOUSE$M2EAK_03	312 (+)	0	100.00	NF-Y	CAGCA
AS$ZIC_21	312 (-)	1	87.50	Zic1	CAGCTGGTC
AS$ZIC3_15	312 (-)	1	87.50	Zic3	CAGCTGGTC
MOMLV$MOMULV_09	314 (-)	0	100.00	LVc	CCTGC
MOMLV$MOMULV_10	314 (-)	0	100.00	LVc	CCTGC
RADLV$RLV_03	314 (-)	0	100.00	LVc	CCTGC
HS$VEGF_02	315 (+)	0	100.00		CAGGT
MOUSE$MYOGN_01	316 (+)	0	100.00	ARP-1	AGGTCT
AS$ERR1_19	316 (+)	0	100.00	ERR1	AGGTCT
RAT$KAT_01	316 (-)	0	100.00	PPAR-alpha, RXR-alpha	AGACCT
AS$SPZ1_24	319 (-)	1	87.50	Spz1	GGTGCTAGA
AMV$AMV_02	320 (+)	0	100.00	C/EBPalpha	CTTGC
RAV0$RAV0_01	320 (-)	0	100.00	C/EBPalpha	GCAAG
MOUSE$MT1_01	322 (+)	0	100.00	Sp1	TGCAC
AS$AREB6_04	322 (+)	1	87.50	AREB6	TGCACCTGT
AS$ERR1_10	323 (-)	0	100.00	ERR1	AGGTGC
PA$PY_12	326 (-)	0	100.00		AGAGG
MOUSE$RAS1_02	328 (+)	0	100.00	GR	TCTTCT
I$HSF_01	329 (-)	0	90.00	HSF	AGAAN
F$HSF_01	329 (-)	0	90.00	HSF	AGAAN
AS$MATA1_04	330 (-)	1	88.89	MATa1	TGATGCAGAA
MOUSE$ICER_01	333 (-)	1	87.50	CREMtau, CREMtau1, CREMtau2, CREMtaualpha, ICER-II, ICER-IIgamma	TGAGCTGCA
HSV1$IE3_03	334 (-)	1	90.00	Cx, POU2F1, Vmw65	TAATGAGATGC
DROME$UBX_21	336 (-)	0	100.00	Zeste	TGAGAT
RAT$AMY2A_01	337 (-)	0	100.00	XPF-1	ATGAGA
ASPN$ABAA_03	340 (+)	0	100.00	abaA	CATTCT
ASPN$ABAA_04	340 (+)	0	100.00	abaA	CATTCT
ASPN$ABAA_05	340 (+)	0	100.00	abaA	CATTCT
ASPN$BRLA_03	340 (+)	0	100.00	abaA	CATTCT
ASPN$RODA_05	340 (+)	0	100.00	abaA	CATTCT
ABAA$CONS_01	340 (+)	0	91.67	abaA	CATTCY
ASPN$BRLA_05	340 (+)	0	100.00	abaA	CATTCT
ASPN$YA_02	340 (-)	0	100.00	abaA	AGAATG
I$HSF_01	341 (-)	0	90.00	HSF	AGAAN
F$HSF_01	341 (-)	0	90.00	HSF	AGAAN
Y$ADH2_01	343 (+)	0	100.00	ADR1	TCTCC
Y$CTT1_02	344 (+)	1	87.50	repressor of CAR1 expression	CTGCAGGCT
Y$GAL1_10	348 (-)	0	100.00	GAL4	AGCCT
AS$AREB6_11	354 (+)	1	87.50	AREB6	CATACCTGT
RAT$KAT_01	355 (+)	0	100.00	PPAR-alpha, RXR-alpha	AGACCT
HS$APAF1_01	355 (+)	1	88.89	p53	AGACATGTCT
MOUSE$MYOGN_01	355 (-)	0	100.00	ARP-1	AGGTCT
AS$ERR1_19	355 (-)	0	100.00	ERR1	AGGTCT
HS$APAF1_01	355 (-)	1	88.89	p53	AGACATGTCT
AS$ZIC_27	356 (-)	1	87.50	Zic1	CGACAGGTC
HS$VEGF_02	357 (-)	0	100.00		CAGGT
HS$GG_12	359 (+)	0	100.00	NF-E	CTGTC
DROME$EVE_10	359 (+)	0	100.00	GAGA factor	CTGTC
AS$MEIS1_10	359 (+)	0	100.00	Meis-1a, Meis-1b	CTGTCT
AS$TGIF_09	360 (+)	0	100.00	TGIF	TGTCT
AS$ARF1_01	360 (+)	0	100.00	ARF1, ARF5	TGTCTC
AS$ARF1_05	360 (+)	0	100.00	ARF1, ARF4, ARF5, ARF6, ARF7, ARF8	TGTCTC
AS$ARF1_01	360 (-)	0	100.00	ARF1, ARF5	GAGACA
PEA$IAA45_07	360 (-)	0	100.00	ARF1	GAGACA
YL$XPR2_01	361 (+)	0	100.00		GTCTC
RAT$ANP_01	361 (-)	0	100.00		GGAGAC
Y$ADH2_01	362 (+)	0	100.00	ADR1	TCTCC
MOUSE$IGLL_02	362 (-)	1	87.50	LyF-1	TCAGGGAGA
AS$LYF1_05	362 (-)	0	100.00	LyF-1	GGGAGA
CHICK$ITGB3_01	362 (-)	0	100.00	RXR-beta, VDR	GGGAGA
HS$CS1_04	363 (-)	0	100.00	T3R	AGGGAG
HS$P21WAF1_05	363 (-)	0	100.00	RXR-alpha, VDR	AGGGAG
AS$VDR_08	363 (-)	0	100.00	VDR	AGGGAG
CTCF$CONS	365 (+)	0	100.00	CTCF	CCCTC
DROME$UBX_18	369 (-)	1	88.89	Hb	TTTTTTAATG
MOUSE$MBP_04	371 (+)	0	100.00	TFIID	TTCAAA
HS$CDC2_10	371 (-)	0	100.00		TTGAA
HS$CYCA_06	371 (-)	0	100.00		TTGAA
HS$CDC25C_05	371 (-)	0	100.00		TTGAA
DROME$UBX_24	372 (-)	0	100.00	LEF-1	TTTGA
HS$IL6_07	373 (+)	1	87.50		CAATAAATA
AS$MEF2_07	373 (+)	1	87.50	MEF-2	CTAAAAATA
VIV$VISNA_02	373 (-)	0	100.00		TTTTTTG
Y$SUC2_01	374 (-)	0	100.00	MIG1	TTTTT
AS$PF1_01	374 (-)	0	100.00	PF1	TTTTT
AS$PF1_02	374 (-)	0	100.00	PF1	TTTTT
Y$SUC2_01	375 (-)	0	100.00	MIG1	TTTTT
AS$PF1_01	375 (-)	0	100.00	PF1	TTTTT
AS$PF1_02	375 (-)	0	100.00	PF1	TTTTT
Y$MAL61_03	376 (-)	0	100.00	MIG1	ATTTT
AS$TBP_60	377 (+)	1	90.00	TBP	AAATATATATT
HS$MSH2_01	377 (-)	0	100.00	HNF-3alpha, HNF-3B	TATTT
CHICK$MYOGN_02	378 (+)	1	87.50		TATATTTAT
AS$TBP_41	378 (-)	0	100.00	TBP	TATAAATATT
AS$TBP_62	378 (-)	1	90.00	TBP	TAATATATATT
SEF1$CONS_01	379 (+)	1	88.89	SEF1	ATATTTAWW
MAIZE$PMS1_02	379 (-)	0	100.00		TAAATAT
CHICK$MLC2A_03	380 (+)	0	100.00	TFIID	TATTTATT
HS$MSH2_01	380 (+)	0	100.00	HNF-3alpha, HNF-3B	TATTT
SV$SV40_03	380 (-)	0	100.00	ETF	ATAAATA
HS$IL6_07	380 (-)	1	87.50		CAATAAATA
CAEEL$MEC3_08	381 (+)	1	87.50	mec-3	ATCTATTAT
HS$GRH_03	381 (+)	0	100.00	POU1F1a	TAAAT
CAEEL$MEC3_07	381 (-)	1	87.50	mec-3	ATAATGAAT
MOUSE$POU4F1_01	381 (-)	0	100.00	POU4F1(1)	ATAATAAAT
HS$CD40_01	381 (-)	1	87.50	AKNA	TTAATAAAT
Y$PDC1_02	382 (+)	0	100.00	MIG1	TTTAT
Y$HAP4_01	382 (+)	0	100.00	MIG1	TTTAT
AS$MEIS1BHOXA9_07	382 (+)	0	100.00	HOXA9, Meis-1b	TTTATTA
HS$EG_08	382 (-)	0	100.00		ATAAA
HS$GFAP_01	382 (-)	0	100.00	TFIID	ATAAA
HS$GFAP_02	382 (-)	0	100.00	TBP	ATAAA
HS$GP2B_11	386 (+)	0	100.00	GATA-1	TTATCG
Y$ARS1_05	386 (+)	0	100.00	ABF2	TTATC
AS$MEIS1AHOXA9_13	386 (+)	0	100.00	HOXA9, Meis-1a	TTATCGA
AS$MTTFA_01	386 (+)	0	100.00	mtTFA	TTATC
AS$MTTFA_02	386 (+)	0	100.00	mtTFA	TTATC
AS$MTTFA_03	386 (+)	0	100.00	mtTFA	TTATC
AS$MTTFA_04	386 (+)	0	100.00	mtTFA	TTATC
AS$MTTFA_05	386 (+)	0	100.00	mtTFA	TTATC
AS$MTTFA_06	386 (+)	0	100.00	mtTFA	TTATC
AS$MTTFA_07	386 (+)	0	100.00	mtTFA	TTATC
AS$MTTFA_08	386 (+)	0	100.00	mtTFA	TTATC
AS$MTTFA_09	386 (+)	0	100.00	mtTFA	TTATC
EBV$BHLF1_05	386 (-)	0	100.00	TBP	GATAA
Y$DAL3_01	386 (-)	0	100.00	DAL80	GATAA
DROME$HSP70_10	387 (+)	0	100.00	BEAF-32A, BEAF-32B	TATCG
DROME$HSP70_10	387 (-)	0	100.00	BEAF-32A, BEAF-32B	CGATA
DROME$HSP70_11	387 (-)	0	100.00	BEAF-32A, BEAF-32B	CGATA
AS$FTZ_18	396 (-)	1	90.00	Ftz	TAGCAATTAAG
AS$FTZ_36	396 (-)	1	90.00	Ftz	AAGCAAGTAAG
HS$TSHB_04	397 (-)	0	100.00	T3R-beta1	AAGTAA
AS$NKX3A_09	398 (-)	0	100.00	NKX3A	CAAGTA
AMV$AMV_02	400 (+)	0	100.00	C/EBPalpha	CTTGC
HIV1$HIV1_28	400 (+)	1	87.50	C/EBPbeta	CTTGCTACA
RAV0$RAV0_01	400 (-)	0	100.00	C/EBPalpha	GCAAG
RAT$EAI_07	404 (+)	0	100.00		CTACCC
TO$E4_02	404 (+)	0	100.00		CTACC
AS$STAT5A_68	405 (+)	1	87.50	STAT5A	TACCCAGAA
SV$SV40_37	407 (-)	0	100.00		CTGGG
CHICK$STN1_01	408 (+)	0	100.00	LBP-1, MyoD	CCAGC
CHICK$STN1_03	408 (+)	0	100.00	LBP-1, NF-1 (-like proteins)	CCAGC
MOUSE$M2EAK_03	409 (+)	0	100.00	NF-Y	CAGCA
RAT$A1I3_04	410 (+)	0	100.00		AGCAC
RAT$A1I3_04	410 (-)	0	100.00		GTGCT
AS$mEMBP_04	412 (+)	0	100.00	EmBP-1a	CACTG
MOUSE$AAG_01	415 (+)	0	100.00	GATA-1, GATA-2	TGATA

MOUSE$AAG_01	415 (+)	0	100.00	GATA-1, GATA-2, GATA-3	TGATA
MOUSE$AAG_06	415 (+)	0	100.00	GATA-1	TGATA
MOUSE$BMG_05	415 (-)	0	100.00	factor B2, GATA-1	TATCA
RAT$GLU_04	417 (-)	0	100.00		TATAT
HS$HH4_08	417 (-)	0	100.00	HiNF-D, HiNF-M, HiNF-P, TFIID, TMF	TATAT
MOUSE$SRF_03	417 (-)	0	100.00	SRF (504 AA)	TATAT
SV$SV40_63	420 (-)	0	100.00	T-Ag	GCCTA
EBV$IR4_05	422 (+)	0	100.00	R	GGCAC
EBV$IR4_06	422 (+)	0	100.00	R	GGCAC
RAT$MT1_06	422 (-)	1	87.50		CTGAGTGCA
XENLA$U2SN_11	422 (-)	0	100.00		GTGCC
DROME$UBX_03	424 (+)	0	100.00	Zeste	CACTCA
DROME$UBX_04	424 (+)	0	100.00	Zeste	CACTCA
AS$WZF1_01	424 (+)	0	100.00	WZF1	CACTC
DROME$WHLO_03	424 (+)	0	100.00	Zeste	CACTCA
DROME$WHLO_06	424 (+)	0	100.00	Zeste	CACTCA
DROME$WHLO_07	424 (+)	0	100.00	Zeste	CACTCA
DROME$WHLO_08	424 (+)	0	100.00	Zeste	CACTCA
DROME$WHLO_09	424 (+)	0	100.00	Zeste	CACTCA
DROME$DPP_02	424 (+)	0	100.00	Zeste	CACTCA
DROME$DPP_04	424 (+)	0	100.00	Zeste	CACTCA
WHEAT$H3_02	424 (+)	0	100.00	WZF1	CACTC
WHEAT$H3_03	424 (+)	0	100.00	WZF1	CACTC
Y$ILV1_02	424 (-)	0	100.00	GCN4	TGAGTG
DROME$WHLO_02	424 (-)	0	100.00	Zeste	TGAGTG
DROME$WHLO_05	424 (-)	0	100.00	Zeste	TGAGTG
DROME$DPP_03	424 (-)	0	100.00	Zeste	TGAGTG
AS$STAT5A_35	425 (-)	1	87.50	STAT5A	TTCCTGAGG
AS$ELK1_13	428 (-)	0	100.00	Elk-1, SAP-1a, SAP-1b, SRF	TTCCTG
DROME$E74_10	429 (+)	0	100.00	E74A	AGGAA
DROME$E74_11	429 (+)	0	100.00	E74A	AGGAA
E74A$CONS_01	429 (+)	0	90.00	E74A	MGGAA
CHICK$CTNT_02	429 (-)	0	100.00	MCBF	ATTCCT
CHICK$CTNT_03	429 (-)	0	100.00	MCBF	ATTCCT
AS$EN1_07	430 (-)	0	100.00	En-1	GTATTCC
Y$GAL3_01	431 (-)	0	100.00	MIG1	TATTC
HS$TCRBL_09	432 (+)	0	100.00		AATACAA
SOYBN$BCGA_04	435 (-)	1	87.50	SEF4	CATTTTTGT
MOUSE$GSR_04	436 (+)	0	100.00		CAACAAT
AS$SOX5_01	437 (+)	0	100.00	Sox-5	AACAAT
SOX5$CONS_01	437 (+)	0	100.00	Sox-5	AACAAT
FY$MFM1_02	437 (+)	1	87.50	mat1-Mc, ste11	AACAAAGAA
AS$SOX20_01	437 (+)	0	100.00	SOX20	AACAAT
AS$SOX15_01	437 (+)	0	100.00	Sox15	AACAATG
FY$MFM1_01	438 (+)	0	100.00	mat1-Mc	ACAATG
CAEEL$MEC3_07	438 (+)	1	87.50	mec-3	ATAATGAAT
HS$IL6_07	439 (+)	1	87.50		CAATAAATA
Y$CYC1_15	440 (-)	1	87.50	TBP	TTATACATT
RAT$PL_12	441 (+)	0	100.00	POU1F1a	ATGAATAAGA
HS$PL_05	441 (+)	0	100.00	POU1F1a	ATGAATA
Y$ADE4_02	441 (+)	1	87.50	GCN4	ATGAATAAT
GHF1$CONS	441 (-)	0	87.50	POU1F1, POU1F1a, PUF-I	WTATYCAT
GHF5$CONS	441 (-)	0	87.50	GHF-5	WTATYCAT
GHF7$CONS	441 (-)	0	87.50	GHF-7	WTATYCAT
Y$GAL3_01	443 (-)	0	100.00	MIG1	TATTC
HS$ADH2_11	445 (-)	0	100.00	GR	TCTTAT
XENLA$ACY_01	447 (+)	0	100.00	SRF	AAGAT
XENLA$ACY_01	447 (-)	0	100.00	SRF	ATCTT
MOUSE$BMG_02	448 (+)	0	100.00	GATA-1	AGATA
HS$AG_03	448 (+)	0	100.00	GATA-1	AGATAG
HS$BG_47	448 (+)	0	100.00	GATA-1	AGATAG
HS$GG_04	448 (-)	0	100.00	GATA-1	ACTATCT
HS$ZG_02	448 (-)	0	100.00	GATA-1	CTATCT
HS$BG_51	448 (-)	0	100.00	GATA-1	CTATCT
MOUSE$GSHPX1_06	448 (-)	0	100.00	GATA-1	TATCT
MOUSE$GSHPX1_07	448 (-)	0	100.00	GATA-1	TATCT
MOUSE$GSHPX1_09	448 (-)	0	100.00	GATA-1	TATCT
MOUSE$IL5_06	448 (-)	0	100.00	GATA-3	CTATCT
HS$CD8A_01	448 (-)	0	100.00	GATA-3	CTATCT
MOUSE$TCRBL_07	448 (-)	0	100.00	GATA-3	CTATCT
HS$GG_08	449 (-)	0	100.00	NF-E	CTATC
MOUSE$CRISP1_02	452 (+)	0	100.00	AR	AGTAGA
CAMV$35SR_06	453 (+)	0	100.00	MNB1b	GTAGAA
MAIZE$PEPC_01	453 (+)	0	100.00	MNB1b, MNF1	GTAGAA
HS$TNFA_04	455 (+)	0	100.00		AGAAA
I$HSF_01	455 (+)	0	90.00	HSF	AGAAN
F$HSF_01	455 (+)	0	90.00	HSF	AGAAN
NT$PR1A_03	456 (+)	0	100.00	GT-1	GAAAAA
Y$GAL4_01	456 (-)	0	100.00	MIG1	TTTTC
Y$SUC2_01	457 (-)	0	100.00	MIG1	TTTTT
AS$PF1_01	457 (-)	0	100.00	PF1	TTTTT
AS$PF1_02	457 (-)	0	100.00	PF1	TTTTT
SPOL$ATPC_01	458 (+)	1	88.89	CBF-C	AAAATTCAAT
Y$MAL61_03	458 (-)	0	100.00	MIG1	ATTTT
CHICK$VIT2_14	459 (-)	0	100.00	C/EBPalpha	AGAATTT
I$HSF_01	461 (-)	0	90.00	HSF	AGAAN
F$HSF_01	461 (-)	0	90.00	HSF	AGAAN
RAT$GLU_04	465 (+)	0	100.00		TATAT
HS$HH4_08	465 (+)	0	100.00	HiNF-D, HiNF-M, HiNF-P, TFIID, TMF	TATAT
MOUSE$SRF_03	465 (+)	0	100.00	SRF (504 AA)	TATAT
XENLA$CFOS_01	470 (+)	1	88.89	Elk-1, SAP-1a, SAP-1b, SRF	CCTTATAAGG
XENLA$CFOS_01	470 (-)	1	88.89	Elk-1, SAP-1a, SAP-1b, SRF	CCTTATAAGG
AS$MTTFA_10	473 (-)	0	100.00	mtTFA	TTATG
AS$MTTFA_11	473 (-)	0	100.00	mtTFA	TTATG
Y$GAL1_10	477 (-)	0	100.00	GAL4	AGCCT
AS$AREB6_44	485 (+)	0	100.00	AREB6	GTTTC
AS$AREB6_46	485 (+)	0	100.00	AREB6	GTTTC
AS$AREB6_48	485 (+)	0	100.00	AREB6	GTTTC
AS$AREB6_49	485 (+)	0	100.00	AREB6	GTTTC
AS$AREB6_50	485 (+)	0	100.00	AREB6	GTTTC
AS$AREB6_52	485 (+)	0	100.00	AREB6	GTTTC
AS$AREB6_53	485 (+)	0	100.00	AREB6	GTTTC
HS$IGKL_01	486 (+)	0	100.00		TTTCCA
HS$IFI616_01	486 (-)	0	100.00	ISGF-3	GGAAA
MOUSE$IL5_02	486 (-)	0	100.00	NF-ATc, NF-ATp, NF-ATx	GGAAA
HS$IL4_01	486 (-)	0	100.00		GGAAA
MOUSE$IL4_06	486 (-)	0	100.00		GGAAA
MOUSE$IL4_07	486 (-)	0	100.00		GGAAA
MOUSE$IL4_08	486 (-)	0	100.00		GGAAA
HS$IL5_02	486 (-)	0	100.00		GGAAA
HS$IL13_01	486 (-)	0	100.00		GGAAA
MOUSE$CD40L_01	486 (-)	0	100.00		GGAAA
MOUSE$CD40L_02	486 (-)	0	100.00		GGAAA
MOUSE$CD40L_03	486 (-)	0	100.00		GGAAA
MOUSE$CD40L_04	486 (-)	0	100.00		GGAAA
HS$CD40L_01	486 (-)	0	100.00		GGAAA
HS$CD40L_02	486 (-)	0	100.00		GGAAA
HS$IFNG_03	486 (-)	0	100.00		GGAAA
HS$IFNG_04	486 (-)	0	100.00		GGAAA
HS$TNFA_05	486 (-)	0	100.00		GGAAA
MOUSE$MCP3_01	486 (-)	0	100.00		GGAAA
HS$TNFSF6_01	486 (-)	0	100.00		GGAAA
AS$STAT5A_70	487 (+)	1	87.50	STAT5A	TTCCAAGTA
CHICK$OA_05	489 (+)	0	100.00		CCATG
CF1$CONS_01	489 (-)	0	91.67	YY1	ANATGG
MOUSE$CMYC_05	489 (-)	0	100.00	YY1	ACATGG
AS$MSX1_12	490 (-)	1	87.50	Msx-1	CAGTACATC
AS$FTZ_55	492 (-)	0	100.00	Ftz	AGTACA
AS$FTZ_39	500 (+)	1	90.00	Ftz	AAGCAATGAAG
AS$FTZ_45	500 (+)	1	90.00	Ftz	AAGCAATTAAC
CHICK$ACRA_06	502 (+)	0	100.00		GCAAT
RSV$RSV_04	502 (+)	0	100.00	C/EBP, C/EBPalpha, C/EBPbeta, C/EBPbeta(p20), C/EBPbeta(p34), C/EBPgamma, EFII	GCAAT
AS$PAX2_44	504 (+)	1	87.50	Pax-2.1, Pax-2.2	AATGAACAC
RAT$GRH_17	505 (+)	1	88.89	GC1, POU1F1a	ATGAATAAAT
HS$ADH3_01	506 (+)	0	100.00	CAR, RAR-alpha1, RAR-beta, RXR-alpha	TGAAC
AS$FOXJ2_02	506 (+)	1	88.89	FOXJ2 (long isoform)	TAAACAAATA
AS$FOXJ2_16	506 (+)	1	88.89	FOXJ2 (long isoform)	TAAACAAATA
RAT$CYTOPB_01	506 (-)	0	100.00	GR	TGTTCA
AS$GR_07	506 (-)	0	100.00	GR	TGTTCA
AS$RAR_05	506 (-)	0	100.00	RAR-alpha1	GTTCA
AS$RAR_06	506 (-)	0	100.00	RAR-alpha1	GTTCA
AS$TR_06	506 (-)	0	100.00	T3R-alpha	GTTCA
AS$TR_07	506 (-)	0	100.00	T3R-alpha	GTTCA
AS$TR_08	506 (-)	0	100.00	T3R-alpha	GTTCA
AS$TR_11	506 (-)	0	100.00	T3R-alpha	GTTCA
MOUSE$RARA2_01	506 (-)	0	100.00	RAR-alpha1, RAR-beta2, RAR-gamma1	GTTCA
MOUSE$CRISP1_03	506 (-)	0	100.00	AR	TGTTCA
AS$HB_09	507 (+)	1	88.89	Hb	GAACAAAAAA
AS$HB_10	507 (+)	1	88.89	Hb	GAACAAAGAA
AS$SRY_03	508 (+)	0	100.00	SRY	AACAAAT
FY$MFM1_02	508 (+)	1	87.50	mat1-Mc, ste11	AACAAAGAA
HS$EGFR_14	510 (+)	0	100.00		CAAAT
MAIZE$PMS1_01	510 (+)	0	100.00		CAAAT
AS$FOXJ2_15	510 (+)	1	88.89	FOXJ2 (long isoform)	CAAATAAATA
HS$IGH_04	510 (-)	0	100.00		ATTTG
RAT$PL_07	511 (+)	0	100.00		AAATAAA
AS$TBP_03	511 (+)	0	100.00	TBP	AAATAAA
MOUSE$HOXA5_04	511 (+)	0	100.00		AAATAA
MOUSE$HOXA5_05	511 (+)	0	100.00		AAATAA
MOUSE$HOXA5_06	511 (+)	0	100.00		AAATAA
HS$MSH2_01	511 (-)	0	100.00	HNF-3alpha, HNF-3B	TATTT
HS$EG_08	513 (+)	0	100.00		ATAAA
HS$GFAP_01	513 (+)	0	100.00	TFIID	ATAAA
HS$GFAP_02	513 (+)	0	100.00	TBP	ATAAA
Y$PDC1_02	513 (-)	0	100.00	MIG1	TTTAT
Y$HAP4_01	513 (-)	0	100.00	MIG1	TTTAT
HS$GRH_03	514 (+)	0	100.00	POU1F1a	TAAAT
MOUSE$BMG_04	516 (+)	0	100.00	GATA-1	AATCT
AS$HOXA3_05	516 (-)	1	87.50	HOXA3	GCTAAGATT
XENLA$ACY_01	517 (+)	0	100.00	SRF	ATCTT
XENLA$ACY_01	517 (-)	0	100.00	SRF	AAGAT
HS$ADH2_11	518 (+)	0	100.00	GR	TCTTAT
MOUSE$GSHPX1_10	519 (+)	0	100.00	GATA-1	CTTATCA
MOUSE$AAG_02	519 (-)	0	100.00		GATAAG
HS$GP2B_05	519 (-)	0	100.00	GATA-1	TGATAAG
RAT$RPK_01	519 (-)	1	87.50	GATA-1	TCAGATAAG
RAT$AR_01	519 (-)	0	100.00	NF-kappaB	GATAAG
HS$AG_06	520 (+)	0	100.00	GATA-1	TTATCA
MOUSE$PBGD_09	520 (+)	0	100.00	GATA-1	TTATCA
Y$ARS1_05	520 (+)	0	100.00	ABF2	TTATC
MOUSE$EKLF_02	520 (+)	0	100.00	GATA-1	TTATCA
HS$SELP_02	520 (+)	0	100.00	GATA-2	TTATCA
CAEEL$PHA4_01	520 (+)	0	100.00	elt-2	TTATCA
AS$STAT5A_68	520 (+)	1	87.50	STAT5A	TTATTAGAG
AS$MTTFA_01	520 (+)	0	100.00	mtTFA	TTATC
AS$MTTFA_02	520 (+)	0	100.00	mtTFA	TTATC
AS$MTTFA_03	520 (+)	0	100.00	mtTFA	TTATC
AS$MTTFA_04	520 (+)	0	100.00	mtTFA	TTATC
AS$MTTFA_05	520 (+)	0	100.00	mtTFA	TTATC
AS$MTTFA_06	520 (+)	0	100.00	mtTFA	TTATC
AS$MTTFA_07	520 (+)	0	100.00	mtTFA	TTATC
AS$MTTFA_08	520 (+)	0	100.00	mtTFA	TTATC
AS$MTTFA_09	520 (+)	0	100.00	mtTFA	TTATC
HS$ZG_05	520 (-)	0	100.00	GATA-1	TGATAA
CHICK$GATA1_01	520 (-)	0	100.00	GATA-1	TGATAA
EBV$BHLF1_05	520 (-)	0	100.00	TBP	GATAA
MOUSE$GATA1_01	520 (-)	1	87.50	GATA-1	ATCTGATAA
Y$DAL3_01	520 (-)	0	100.00	DAL80	GATAA
MOUSE$TCRBL_02	520 (-)	0	100.00	GATA-3	TGATAA
RAT$BNP_01	520 (-)	0	100.00	GATA-4	TGATAA
MOUSE$BMG_05	521 (+)	0	100.00	factor B2, GATA-1	TATCA
MOUSE$AAG_01	521 (-)	0	100.00	GATA-1, GATA-2, GATA-3	TGATA
MOUSE$AAG_06	521 (-)	0	100.00	GATA-1	TGATA
CHICK$VIT2_13	522 (+)	0	100.00		ATCAGA
RAT$POMC_03	524 (+)	0	100.00	GR	CAGAG
AS$WZF1_01	526 (-)	0	100.00	WZF1	CACTC
WHEAT$H3_02	526 (-)	0	100.00	WZF1	CACTC
WHEAT$H3_03	526 (-)	0	100.00	WZF1	CACTC
NEUCR$NIT3_01	528 (-)	0	100.00	NIT2	TATCAC
HS$ADH2_10	528 (-)	0	100.00	GR	TATCAC
MOUSE$AAG_01	529 (+)	0	100.00	GATA-1, GATA-2, GATA-3	TGATA
MOUSE$AAG_06	529 (+)	0	100.00	GATA-1	TGATA
HS$ZG_05	529 (+)	0	100.00	GATA-1	TGATAA
HS$GP2B_05	529 (+)	0	100.00	GATA-1	TGATAAG
CHICK$GATA1_01	529 (+)	0	100.00	GATA-1	TGATAA
MOUSE$TCRBL_02	529 (+)	0	100.00	GATA-3	TGATAA
RAT$BNP_01	529 (+)	0	100.00	GATA-4	TGATAA
MOUSE$BMG_05	529 (-)	0	100.00	factor B2, GATA-1	TATCA
HS$GG_31	529 (-)	0	100.00	GATA-1	CCTTATCA
HS$AG_06	529 (-)	0	100.00	GATA-1	TTATCA
MOUSE$PBGD_09	529 (-)	0	100.00	GATA-1	TTATCA
MOUSE$EKLF_02	529 (-)	0	100.00	GATA-1	TTATCA
MOUSE$GSHPX1_10	529 (-)	0	100.00	GATA-1	CTTATCA
HS$SELP_02	529 (-)	0	100.00	GATA-2	TTATCA
CAEEL$PHA4_01	529 (-)	0	100.00	elt-2	TTATCA
MOUSE$AAG_02	530 (+)	0	100.00		GATAAG
EBV$BHLF1_05	530 (+)	0	100.00	TBP	GATAA
Y$DAL3_01	530 (+)	0	100.00	DAL80	GATAA
AS$VMYB_01	530 (+)	1	88.89	v-Myb	GATAACGGTT
RAT$AR_01	530 (+)	0	100.00	NF-kappaB	GATAAG
Y$ARS1_05	530 (-)	0	100.00	ABF2	TTATC
AS$MTTFA_01	530 (-)	0	100.00	mtTFA	TTATC
AS$MTTFA_02	530 (-)	0	100.00	mtTFA	TTATC
AS$MTTFA_03	530 (-)	0	100.00	mtTFA	TTATC
AS$MTTFA_04	530 (-)	0	100.00	mtTFA	TTATC
AS$MTTFA_05	530 (-)	0	100.00	mtTFA	TTATC
AS$MTTFA_06	530 (-)	0	100.00	mtTFA	TTATC
AS$MTTFA_07	530 (-)	0	100.00	mtTFA	TTATC
AS$MTTFA_08	530 (-)	0	100.00	mtTFA	TTATC
AS$MTTFA_09	530 (-)	0	100.00	mtTFA	TTATC
Y$GLK1_01	542 (+)	0	100.00	GCR1	GAAGGAG
MOUSE$PCP2_01	544 (+)	0	100.00	RXR-beta, T3R-alpha1, T3R-beta1	AGGAGA
HS$GP2B_14	545 (+)	1	88.89	GATA-1	GGAGATTAGA
Y$ADH2_01	545 (-)	0	100.00	ADR1	TCTCC
DROME$KR_13	546 (-)	1	87.50	Bcd	GTTAATCTC
SP$SPEC2A_02	547 (+)	0	100.00	SpOtx	AGATTA
MOUSE$CTNC_01	547 (+)	0	100.00	GATA-4	AGATTA
BOVIN$LHB_02	547 (+)	0	100.00	Pitx1	AGATTA
MOUSE$BMG_04	547 (-)	0	100.00	GATA-1	AATCT
SP$SPEC2A_02	547 (-)	0	100.00	SpOtx	TAATCT
Y$MEL1_02	549 (+)	0	100.00	MIG1	ATTAA
CHICK$MGF_01	549 (+)	0	100.00	Gbx2	ATTAA
RAT$BCAS_07	549 (-)	1	88.89	C/EBPbeta, C/EBPdelta	CTTGTTTAAT
CHICK$MGF_02	549 (-)	0	100.00	Gbx2	TTAAT
DROME$ADH_09	550 (-)	0	100.00	TBP	TATTTAA
Y$LEU2_02	550 (-)	0	100.00	TBP, TFIIA, TOA1, TOA2	TATTTAA
Y$MAL63_01	550 (-)	0	100.00	MIG1	TTTAA
HS$GRH_03	551 (+)	0	100.00	POU1F1a	TAAAT
MOUSE$HOXA5_04	552 (+)	0	100.00		AAATAA
MOUSE$HOXA5_05	552 (+)	0	100.00		AAATAA
MOUSE$HOXA5_06	552 (+)	0	100.00		AAATAA
HS$MSH2_01	552 (-)	0	100.00	HNF-3alpha, HNF-3B	TATTT
HS$ADH2_11	554 (-)	0	100.00	GR	TCTTAT
XENLA$ACY_01	556 (+)	0	100.00	SRF	AAGAT
XENLA$ACY_01	556 (-)	0	100.00	SRF	ATCTT
CF1$CONS_01	557 (+)	0	91.67	YY1	ANATGG
MOUSE$TRP1_03	562 (+)	0	100.00	Tbx2	GTGTGA
MOUSE$TRP1_04	562 (+)	0	100.00	Tbx2	GTGTGA
RAT$POMC_04	563 (+)	0	100.00	GR	TGTGAT
NEUCR$NIT3_01	564 (-)	0	100.00	NIT2	TATCAC
HS$ADH2_10	564 (-)	0	100.00	GR	TATCAC
MOUSE$AAG_01	565 (+)	0	100.00	GATA-1, GATA-2, GATA-3	TGATA
MOUSE$AAG_06	565 (+)	0	100.00	GATA-1	TGATA
MOUSE$BMG_05	565 (-)	0	100.00	factor B2, GATA-1	TATCA
Y$GAL1_12	567 (+)	0	100.00	GAL4	ATATAA
Y$GAL1_02	567 (-)	0	100.00		TTATAT
RAT$GLU_04	567 (-)	0	100.00		TATAT
HS$HH4_08	567 (-)	0	100.00	HiNF-D, HiNF-M, HiNF-P, TFIID, TMF	TATAT
MOUSE$SRF_03	567 (-)	0	100.00	SRF (504 AA)	TATAT
CHICK$BAC_05	568 (+)	0	100.00	ETF	TATAA
AD$E3_06	568 (+)	0	100.00	TFIID	TATAA
HIV1$HIV1_12	568 (+)	0	100.00	DBF4, NC1, TBP, TFIIA, TFIIA-alpha/beta precursor (major), TFIIA-alpha/beta precursor (minor), TFIIA-gamma, TFIIB, TFIID, TMF	TATAA
HIV1$HIV1_13	568 (+)	0	100.00	UBP-1	TATAA
AD$MLP_29	568 (+)	0	100.00	TFIID	TATAAA
TBP$CONS	568 (+)	0	100.00	TBP	TATAAA
HS$ASCC_04	568 (+)	0	100.00		TATAA
Y$CUP1_07	568 (+)	0	100.00	TBP	TATAAA
MOUSE$MT1_04	568 (+)	0	100.00	TBP	TATAAA
AS$TBP_02	568 (+)	0	100.00	TBP	TATAAA
XENLA$MYODA_02	568 (+)	0	100.00	TBP	TATAAA
RAT$MRF4_03	568 (+)	0	100.00	TBP	TATAAA
AS$PAX2_57	568 (+)	1	87.50	Pax-2.1, Pax-2.2	TATAAAGTG
HS$EG_08	569 (+)	0	100.00		ATAAA
HS$GFAP_01	569 (+)	0	100.00	TFIID	ATAAA
HS$GFAP_02	569 (+)	0	100.00	TBP	ATAAA
Y$PDC1_02	569 (-)	0	100.00	MIG1	TTTAT
Y$HAP4_01	569 (-)	0	100.00	MIG1	TTTAT
ARHIZ$ROLB_01	570 (-)	0	100.00	BBF1	ACTTTA
Y$PHR1_01	571 (+)	0	100.00	PRP, RPH1	AAAGTA
Y$PHR1_01	571 (-)	0	100.00	PRP, RPH1	TACTTT
RAT$GHF1_01	573 (+)	0	100.00	PTF	AGTAT
RAT$GHF1_01	573 (-)	0	100.00	PTF	ATACT
HS$GPB_05	574 (+)	0	100.00	GATA-1	GTATCT
MOUSE$GSHPX1_06	575 (+)	0	100.00	GATA-1	TATCT
MOUSE$GSHPX1_07	575 (+)	0	100.00	GATA-1	TATCT
MOUSE$GSHPX1_09	575 (+)	0	100.00	GATA-1	TATCT
MOUSE$BMG_02	575 (-)	0	100.00	GATA-1	AGATA
MOUSE$TDT_02	577 (+)	1	87.50	LyF-1	TTTGGGAGA
MOUSE$IGLL_02	577 (+)	1	87.50	LyF-1	TCAGGGAGA
MOUSE$IGLL_06	577 (+)	1	87.50	LyF-1	TTTGGGAGA
AS$LYF1_01	577 (+)	1	87.50	LyF-1	TTTGGGAGA
AS$LYF1_04	577 (+)	1	87.50	LyF-1	TTTGGGAGA
AS$LYF1_06	577 (+)	1	87.50	LyF-1	TCTGGGAGG
AS$LYF1_07	577 (+)	1	87.50	LyF-1	CCTGGGAGA
MOUSE$IGLL_03	577 (-)	1	87.50	LyF-1	CCTCCCAGA
HS$LCK_01	577 (-)	1	87.50	LyF-1	TCTCCCAGG
SV$SV40_37	578 (+)	0	100.00		CTGGG
RAT$AMGL_02	578 (+)	0	100.00	IL-6 RE-BP, STAT1, STAT5A, STAT5B, STAT6	CTGGGA
HS$APOB_10	579 (+)	0	100.00	AP-2alphaA, AP-2alphaB	TGGGAG
AS$DL_33	579 (+)	1	90.00	Dl	TGGGAGAAACC
HS$CDH1_01	579 (+)	0	100.00	LUN-1	TGGGA
HS$TLN_01	579 (+)	0	100.00	LUN-1	TGGGA
HS$A11COL_07	579 (-)	0	100.00		TCTCCCA
HS$CDH1_01	579 (-)	0	100.00	LUN-1	TCCCA
HS$TLN_01	579 (-)	0	100.00	LUN-1	TCCCA
AS$LYF1_05	580 (+)	0	100.00	LyF-1	GGGAGA
CHICK$ITGB3_01	580 (+)	0	100.00	RXR-beta, VDR	GGGAGA
Y$ADH2_01	581 (-)	0	100.00	ADR1	TCTCC
HS$TNFA_04	583 (+)	0	100.00		AGAAA
I$HSF_01	583 (+)	0	90.00	HSF	AGAAN
F$HSF_01	583 (+)	0	90.00	HSF	AGAAN
Y$GAL4_01	584 (-)	0	100.00	MIG1	TTTTC
AS$TBF1_01	591 (+)	0	100.00	TBF1	TTAGGG
CHICK$PTH_01	594 (+)	0	100.00	RXR-alpha, VDR	GGGTGT
HS$BG_01	594 (-)	0	100.00	CACCC-binding factor	CACCC
HS$GG_13	594 (-)	0	100.00	gammaCAC1, gammaCAC2	CACCC
HS$GG_14	594 (-)	0	100.00	CACCC-binding factor, Sp1	CACCC
HS$GP2B_01	594 (-)	0	100.00	CACCC-binding factor	CACCC
RAT$TOA_02	594 (-)	0	100.00	CACCC-binding factor	CACCC
HS$EG_06	594 (-)	0	100.00	Sp1	CACCC
MOUSE$GATA1_02	594 (-)	0	100.00		CACACCC
AS$CACCC_01	594 (-)	0	100.00	CACCC-binding factor	CACCC
HS$CYP24_01	594 (-)	0	100.00	VDR	ACACCC
MOUSE$TRP1_03	596 (+)	0	100.00	Tbx2	GTGTGA
MOUSE$TRP1_04	596 (+)	0	100.00	Tbx2	GTGTGA
RAT$POMC_04	597 (+)	0	100.00	GR	TGTGAT
NEUCR$NIT3_01	598 (-)	0	100.00	NIT2	TATCAC
HS$ADH2_10	598 (-)	0	100.00	GR	TATCAC
MOUSE$AAG_01	599 (+)	0	100.00	GATA-1, GATA-2, GATA-3	TGATA
MOUSE$AAG_06	599 (+)	0	100.00	GATA-1	TGATA
MOUSE$BMG_05	599 (-)	0	100.00	factor B2, GATA-1	TATCA
RAT$GLU_09	601 (-)	0	100.00	Cdx-3	TAATAT
AMV$AMV_01	603 (+)	0	100.00	C/EBPalpha	ATTAC
FSV$FSV_01	603 (+)	0	100.00	C/EBPalpha	ATTAC
AS$STAT_03	604 (-)	1	87.50	STAT6	TTCCCGTAA
AS$STAT_13	604 (-)	1	87.50	STAT6	TTCCCGTAA
AS$STAT3_01	604 (-)	1	87.50	STAT3	TTCCCGTAA
AS$ELK1_09	606 (-)	0	100.00	Elk-1, SAP-1a, SAP-1b, SRF	TTCCGT
E74A$CONS_01	607 (+)	0	90.00	E74A	MGGAA
MOUSE$MCP3_02	607 (+)	0	100.00		CGGAAA
HS$IFI616_01	608 (+)	0	100.00	ISGF-3	GGAAA
MOUSE$IL5_02	608 (+)	0	100.00	NF-ATc, NF-ATp, NF-ATx	GGAAA
HS$IL4_01	608 (+)	0	100.00		GGAAA
MOUSE$IL4_06	608 (+)	0	100.00		GGAAA
MOUSE$IL4_07	608 (+)	0	100.00		GGAAA
MOUSE$IL4_08	608 (+)	0	100.00		GGAAA
HS$IL5_02	608 (+)	0	100.00		GGAAA
HS$IL13_01	608 (+)	0	100.00		GGAAA
MOUSE$CD40L_01	608 (+)	0	100.00		GGAAA
MOUSE$CD40L_02	608 (+)	0	100.00		GGAAA
MOUSE$CD40L_03	608 (+)	0	100.00		GGAAA
MOUSE$CD40L_04	608 (+)	0	100.00		GGAAA
HS$CD40L_01	608 (+)	0	100.00		GGAAA
HS$CD40L_02	608 (+)	0	100.00		GGAAA
HS$IFNG_03	608 (+)	0	100.00		GGAAA
HS$IFNG_04	608 (+)	0	100.00		GGAAA
HS$TNFA_05	608 (+)	0	100.00		GGAAA
MOUSE$MCP3_01	608 (+)	0	100.00		GGAAA
HS$TNFSF6_01	608 (+)	0	100.00		GGAAA
HCMV$IE1_16	608 (-)	0	100.00	NF-1	CTTTCC
Y$GAL1_10	612 (+)	0	100.00	GAL4	AGCCT
HBV$S_05	613 (-)	0	100.00		GGAAGGC
XENLA$RPL14_01	615 (+)	0	100.00	HrpF, XrpFI	CTTCC
Y$TPI_01	615 (+)	0	100.00	GCR1	CTTCC
MOUSE$RPL32_01	615 (-)	0	100.00	f(alpha)-f(epsilon), HrpF, XrpFI	GGAAG
PEA3$CONS	615 (-)	0	91.67	c-Ets-1 54, c-Ets-1 68, c-Ets-2 58-64, PEA3	AGGAAR
AS$LYF1_10	615 (-)	0	100.00	LyF-1	TTTAGGAAG
HS$TNFA_02	615 (-)	0	100.00		GGAAG
HS$TNFA_03	615 (-)	0	100.00		GGAAG
AS$STAT5A_48	616 (+)	1	87.50	STAT5A	TTCCTAGAA
DROME$E74_10	616 (-)	0	100.00	E74A	AGGAA
DROME$E74_11	616 (-)	0	100.00	E74A	AGGAA
E74A$CONS_01	616 (-)	0	90.00	E74A	MGGAA
AS$LYF1_11	616 (-)	1	87.50	LyF-1	TTTGAGGAA
AS$E2TBP_03	618 (+)	0	100.00	E2	CCTAAAA
DROME$FTZ_29	618 (-)	0	100.00	Cad	TTTAGG
RAT$AMHC_04	619 (+)	1	87.50	aMEF-2, MEF-2 (516 AA)	CTAAAAAAG
MOUSE$ADA_06	620 (+)	0	100.00	TBP	TAAAAAA
Y$MAL2R_01	620 (-)	0	100.00	MIG1	TTTTA
AS$FOXJ1_04	620 (-)	0	100.00	FOXJ1	TTTTA
Y$SUC2_01	621 (-)	0	100.00	MIG1	TTTTT
AS$PF1_01	621 (-)	0	100.00	PF1	TTTTT
AS$PF1_02	621 (-)	0	100.00	PF1	TTTTT
Y$SUC2_01	622 (-)	0	100.00	MIG1	TTTTT
AS$PF1_01	622 (-)	0	100.00	PF1	TTTTT
AS$PF1_02	622 (-)	0	100.00	PF1	TTTTT
MOUSE$COX2_02	622 (-)	1	88.89	IRF-1, IRF-2	TTTCATTTTT
Y$MAL61_03	623 (-)	0	100.00	MIG1	ATTTT
HS$GMCSF_03	624 (-)	0	100.00	YY1	CATTT
MOUSE$IL4_01	624 (-)	0	100.00	NF-CLE0a, NF-CLE0b	TCATTT
AS$MEIS1BHOXA9_09	627 (+)	0	100.00	HOXA9, Meis-1b	TGACAT
AS$MEIS1BHOXA9_11	627 (+)	0	100.00	HOXA9, Meis-1b	TGACAT
POT$PR10a_01	627 (+)	0	100.00	PBF-1, PBF-2 (p24)	TGACA
AS$TGIF_01	627 (-)	0	100.00	TGIF	TGTCA
AS$TGIF_02	627 (-)	0	100.00	TGIF	TGTCA
AS$TGIF_03	627 (-)	0	100.00	TGIF	TGTCA
AS$TGIF_04	627 (-)	0	100.00	TGIF	TGTCA
AS$TGIF_05	627 (-)	0	100.00	TGIF	TGTCA
AS$TGIF_06	627 (-)	0	100.00	TGIF	TGTCA
AS$TGIF_07	627 (-)	0	100.00	TGIF	TGTCA
AS$TGIF_08	627 (-)	0	100.00	TGIF	TGTCA
AS$TGIF_10	627 (-)	0	100.00	TGIF	TGTCA
AS$TGIF_11	627 (-)	0	100.00	TGIF	TGTCA
AS$TGIF_12	627 (-)	0	100.00	TGIF	TGTCA
AS$TGIF_13	627 (-)	0	100.00	TGIF	TGTCA
AS$TGIF_14	627 (-)	0	100.00	TGIF	TGTCA
AS$TGIF_15	627 (-)	0	100.00	TGIF	TGTCA
AS$MEIS1_14	627 (-)	0	100.00	Meis-1a, Meis-1b	ATGTCA
POT$PR10a_01	627 (-)	0	100.00	PBF-1, PBF-2 (p24)	TGTCA
HS$GMCSF_03	630 (+)	0	100.00	YY1	CATTT
Y$MAL61_03	631 (+)	0	100.00	MIG1	ATTTT
RAT$PL_15	631 (-)	0	100.00	F2F, POU1F1a	TAAAAT
Y$MAL2R_01	632 (+)	0	100.00	MIG1	TTTTA
AS$FOXJ1_04	632 (+)	0	100.00	FOXJ1	TTTTA
Y$MAL63_01	633 (+)	0	100.00	MIG1	TTTAA
AS$AREB6_10	633 (+)	1	87.50	AREB6	TTTACCTGA
DROME$EN_05	633 (-)	1	87.50	En	TCAATTAAA
AS$CMYB_02	635 (+)	0	100.00	c-Myb	TAACTG
MOUSE$RVL3_03	635 (+)	0	100.00	RXR-alpha	TAACT
NT$PR1A_02	635 (+)	0	100.00	MYB1	TAACTG
MAIZE$BZ1_02	635 (+)	0	100.00	C1 (long form), MYB2	TAACTG
AS$CMYB_01	635 (-)	0	100.00	c-Myb	CAGTTA
AS$CMYB_02	635 (-)	0	100.00	c-Myb	CAGTTA
HS$PR264_03	635 (-)	0	100.00	c-Myb	CAGTTA
HS$IFNB_03	636 (+)	0	100.00		AACTGA
DROME$MDG1_01	636 (-)	0	100.00		TCAGTT
RAT$GK_02	638 (-)	0	100.00	IPF1	CATCAG
RAT$AMY2A_01	641 (+)	0	100.00	XPF-1	ATGAGA
HS$CMYC_13	643 (+)	1	87.50		GAAAAGAAA
HS$EGFR_18	643 (-)	0	100.00		TTCTTCTC
MOUSE$GSHPX1_01	643 (-)	0	100.00	PU.1	CTTCTC
I$HSF_01	644 (+)	0	90.00	HSF	AGAAN
F$HSF_01	644 (+)	0	90.00	HSF	AGAAN
MOUSE$RAS1_02	644 (-)	0	100.00	GR	TCTTCT
AS$SRY_02	646 (+)	0	100.00	SRY	AAGAAAG
HS$TNFA_04	647 (+)	0	100.00		AGAAA
I$HSF_01	647 (+)	0	90.00	HSF	AGAAN
F$HSF_01	647 (+)	0	90.00	HSF	AGAAN
AS$ZIC3_25	650 (-)	1	87.50	Zic3	TGGGTCCTT
Y$LYS1_01	655 (+)	1	87.50	LYS14	TCCAGCGGA
V$AP4_06	655 (-)	0	90.00	AP-4	CWCAGCTGGN
CHICK$STN1_01	656 (+)	0	100.00	LBP-1, MyoD	CCAGC
CHICK$STN1_03	656 (+)	0	100.00	LBP-1, NF-1 (-like proteins)	CCAGC
RAT$EAI_08	657 (+)	0	100.00	XPF-1	CAGCTG
HS$PK_04	657 (+)	0	100.00	AP-4	CAGCTG
AS$AP4_01	657 (+)	0	100.00	AP-4	CAGCTG
MOUSE$CD4_03	657 (+)	0	100.00	E12, HEB	CAGCTG
RAT$AMY2A_02	657 (+)	0	100.00	XPF-1	CAGCTG
MOUSE$ACRG_02	657 (+)	0	100.00	MyoD	CAGCTG
AS$HEN1_01	657 (+)	0	100.00	E12, HEN1	CAGCTG
RAT$EAI_08	657 (-)	0	100.00	XPF-1	CAGCTG
HS$PK_04	657 (-)	0	100.00	AP-4	CAGCTG
AD5$E1A_27	657 (-)	1	87.50		TCTCAGGTG
AS$AP4_01	657 (-)	0	100.00	AP-4	CAGCTG
MOUSE$CD4_03	657 (-)	0	100.00	E12, HEB	CAGCTG
RAT$AMY2A_02	657 (-)	0	100.00	XPF-1	CAGCTG
MOUSE$ACRG_02	657 (-)	0	100.00	MyoD	CAGCTG
AS$HEN1_01	657 (-)	0	100.00	E12, HEN1	CAGCTG
RAT$MT1_06	660 (+)	1	87.50		CTGAGTGCA
DROME$EVE_08	662 (+)	0	100.00	GAGA factor	GAGAG
DROME$EVE_13	662 (+)	0	100.00	GAGA factor	GAGAG
DROME$FTZ_32	662 (+)	0	100.00	GAGA factor	GAGAG
AS$GAGA_03	662 (+)	0	100.00	GAGA factor	GAGAG
AS$ZF5_04	662 (+)	1	88.89	ZF5	GCGAGCAAAC
DROME$EVE_09	662 (-)	0	100.00	GAGA factor	CTCTC
DROME$FTZ_32	662 (-)	0	100.00	GAGA factor	CTCTC
MOUSE$CRISP1_04	663 (+)	0	100.00	AR	AGAGCA
MOUSE$CRISP3_02	663 (+)	0	100.00	AR	AGAGCA
HS$TF_12	663 (-)	1	87.50	EBP40, EBP45	GTTTGCTTT
RAT$VEGF_02	664 (+)	0	100.00	ER-alpha, ER-beta	GAGCA
Y$FRE1_02	664 (+)	0	100.00	MAC1	GAGCAAA
Y$CTR3_02	664 (+)	0	100.00	MAC1	GAGCAAA
Y$FRE1_01	664 (-)	0	100.00	MAC1	TTTGCTC
Y$CTR1_01	664 (-)	0	100.00	MAC1	TTTGCTC
Y$CTR1_02	664 (-)	0	100.00	MAC1	TTTGCTC
Y$CTR3_01	664 (-)	0	100.00	MAC1	TTTGCTC
MOUSE$AFEP_15	665 (-)	0	100.00	C/EBPalpha	GTTTGCT
MOUSE$AFEP_16	665 (-)	0	100.00	HNF-1A	GTTTGCT
AS$AREB6_42	667 (-)	0	100.00	AREB6	GTTTG
AS$AREB6_43	667 (-)	0	100.00	AREB6	GTTTG
AS$AREB6_45	667 (-)	0	100.00	AREB6	GTTTG
AS$AREB6_47	667 (-)	0	100.00	AREB6	GTTTG
AS$AREB6_51	667 (-)	0	100.00	AREB6	GTTTG
HS$PR264_05	668 (-)	0	100.00	c-Myb	GCGTTT
AS$FTZ_49	675 (+)	0	100.00	Ftz	AAAGCT
AS$FTZ_49	677 (-)	0	100.00	Ftz	AAAGCT
AS$SRY_02	679 (-)	0	100.00	SRY	AAGAAAG
HS$TNFA_04	680 (-)	0	100.00		AGAAA
I$HSF_01	680 (-)	0	90.00	HSF	AGAAN
F$HSF_01	680 (-)	0	90.00	HSF	AGAAN
HS$DG_03	682 (+)	1	88.89		CCTTCCTTCC
XENLA$RPL14_01	683 (+)	0	100.00	HrpF, XrpFI	CTTCC
Y$TPI_01	683 (+)	0	100.00	GCR1	CTTCC
MOUSE$RPL32_01	683 (-)	0	100.00	f(alpha)-f(epsilon), HrpF, XrpFI	GGAAG
PEA3$CONS	683 (-)	0	91.67	c-Ets-1 54, c-Ets-1 68, c-Ets-2 58-64, PEA3	AGGAAR
HS$TNFA_02	683 (-)	0	100.00		GGAAG
HS$TNFA_03	683 (-)	0	100.00		GGAAG
HS$CDC2_02	684 (+)	0	100.00	c-Ets-2	TTCCTT
DROME$E74_10	684 (-)	0	100.00	E74A	AGGAA
DROME$E74_11	684 (-)	0	100.00	E74A	AGGAA
E74A$CONS_01	684 (-)	0	90.00	E74A	MGGAA
MOUSE$M1H2KB_11	684 (-)	0	100.00		GAAGGAA
HS$CDC2_01	684 (-)	0	100.00	c-Ets-2	AAGGAA
HS$GG_28	686 (-)	0	100.00	PPUR	TGGAAGG
XENLA$RPL14_01	687 (+)	0	100.00	HrpF, XrpFI	CTTCC
Y$TPI_01	687 (+)	0	100.00	GCR1	CTTCC
MOUSE$RPL32_01	687 (-)	0	100.00	f(alpha)-f(epsilon), HrpF, XrpFI	GGAAG
MOUSE$NE_01	687 (-)	1	88.89	PU.1	AGGGAGGAAG
HS$TNFA_02	687 (-)	0	100.00		GGAAG
HS$TNFA_03	687 (-)	0	100.00		GGAAG
AS$ZIC2_15	688 (-)	0	100.00	Zic2	AGGGTGGAA
AS$ADR1_05	689 (-)	0	100.00	ADR1	AGGGTGGA
RAT$PTHRP_01	689 (-)	0	100.00	RXR-alpha, VDR	GGGTGGA
HT1$HTLV1_08	690 (+)	0	100.00	c-Ets-1 68	CCACC
HS$BG_52	690 (+)	0	100.00		CCACC
HS$BG_14	690 (+)	0	100.00		CCACC
HS$BG_44	690 (+)	0	100.00	CAC-binding protein	CCACC
HS$MIP_07	690 (+)	0	100.00	Sp1	CCACCC
MOUSE$P53_09	690 (+)	0	100.00	ETF	CCACCC
DROME$EN_08	690 (-)	0	100.00	Ttk 88K	AGGGTGG
TTK88$CONS	690 (-)	0	92.86	Ttk 88K	AGGGYGG
BPV1$BPV1_24	690 (-)	0	100.00	Sp1	GGGTGG
BPV1$BPV1_24	690 (-)	0	100.00	Sp1	GGTGG
HS$AG_12	690 (-)	0	100.00	CACCC-binding factor	GGGTGG
AS$RAR_07	690 (-)	0	100.00	RAR-alpha1, RXR-alpha	GGGTGG
HS$BG_48	690 (-)	0	100.00	CAC-binding protein	GGTGG
MOUSE$CEBPA_06	690 (-)	0	100.00	Sp1	GGTGG
HS$BG_01	691 (+)	0	100.00	CACCC-binding factor	CACCC
HS$GG_13	691 (+)	0	100.00	gammaCAC1, gammaCAC2	CACCC
HS$GG_14	691 (+)	0	100.00	CACCC-binding factor, Sp1	CACCC
HS$GP2B_01	691 (+)	0	100.00	CACCC-binding factor	CACCC
RAT$TOA_02	691 (+)	0	100.00	CACCC-binding factor	CACCC
HS$EG_06	691 (+)	0	100.00	Sp1	CACCC
AS$CACCC_01	691 (+)	0	100.00	CACCC-binding factor	CACCC
HS$MSH2_01	700 (+)	0	100.00	HNF-3alpha, HNF-3B	TATTT
HS$IGH_04	701 (+)	0	100.00		ATTTG
HS$EGFR_14	701 (-)	0	100.00		CAAAT
MAIZE$PMS1_01	701 (-)	0	100.00		CAAAT
DROME$UBX_24	702 (+)	0	100.00	LEF-1	TTTGA
AS$MEIS1AHOXA9_06	702 (-)	0	100.00	HOXA9, Meis-1a	TGTCAAA
AS$TWRKY_01	703 (+)	0	100.00	WRKY3, WRKY4	TTGAC
AS$WRKY_01	703 (+)	0	100.00	WRKY1, WRKY2	TTGAC
AS$WRKY_02	703 (+)	0	100.00	WRKY1, WRKY2	TTGAC
AS$WRKY_03	703 (+)	0	100.00	WRKY1, WRKY2	TTGAC
AS$WRKY_04	703 (+)	0	100.00	WRKY1, WRKY2	TTGAC
AT$RLK4_01	703 (+)	0	100.00	WRKY18	TTGAC
AT$RLK4_02	703 (+)	0	100.00	WRKY18	TTGAC
EBV$DSL_04	704 (+)	0	100.00	Zta	TGACACA
EBV$DSL_05	704 (+)	0	100.00	Zta	TGACACA
AS$MEIS1_02	704 (+)	0	100.00	Meis-1a, Meis-1b	TGACAC
AS$MEIS1BHOXA9_10	704 (+)	0	100.00	HOXA9, Meis-1b	TGACAC
AS$MEIS1BHOXA9_12	704 (+)	0	100.00	HOXA9, Meis-1b	TGACAC
AS$MEIS1BHOXA9_13	704 (+)	0	100.00	HOXA9, Meis-1b	TGACAC
AS$MEIS1BHOXA9_14	704 (+)	0	100.00	HOXA9, Meis-1b	TGACAC
POT$PR10a_01	704 (+)	0	100.00	PBF-1, PBF-2 (p24)	TGACA
EBV$DSL_02	704 (-)	0	100.00	Zta	TGTGTCA
CHICK$OA_02	704 (-)	0	100.00	ARP-1, COUP, COUP-TF1, PPAR-alpha, RXR-alpha	GTGTCA
SV$SV40_44	704 (-)	0	100.00	AP-1, c-Jun, v-Jun, YAP1	TGTGTCA
AS$TGIF_01	704 (-)	0	100.00	TGIF	TGTCA
AS$TGIF_02	704 (-)	0	100.00	TGIF	TGTCA
AS$TGIF_03	704 (-)	0	100.00	TGIF	TGTCA
AS$TGIF_04	704 (-)	0	100.00	TGIF	TGTCA
AS$TGIF_05	704 (-)	0	100.00	TGIF	TGTCA
AS$TGIF_06	704 (-)	0	100.00	TGIF	TGTCA
AS$TGIF_07	704 (-)	0	100.00	TGIF	TGTCA
AS$TGIF_08	704 (-)	0	100.00	TGIF	TGTCA
AS$TGIF_10	704 (-)	0	100.00	TGIF	TGTCA
AS$TGIF_11	704 (-)	0	100.00	TGIF	TGTCA
AS$TGIF_12	704 (-)	0	100.00	TGIF	TGTCA
AS$TGIF_13	704 (-)	0	100.00	TGIF	TGTCA
AS$TGIF_14	704 (-)	0	100.00	TGIF	TGTCA
AS$TGIF_15	704 (-)	0	100.00	TGIF	TGTCA
POT$PR10a_01	704 (-)	0	100.00	PBF-1, PBF-2 (p24)	TGTCA
RAT$A2UG_11	705 (+)	0	100.00	GR	GACACA
MAIZE$C1_05	705 (-)	0	100.00		GTGTC
AS$ZIC3_18	707 (-)	1	87.50	Zic3	TGCATGGTG
FY$MFM1_01	708 (+)	0	100.00	mat1-Mc	ACAATG
HS$EGFR_20	713 (-)	0	100.00		TCCTGC
MOMLV$MOMULV_09	713 (-)	0	100.00	LVc	CCTGC
MOMLV$MOMULV_10	713 (-)	0	100.00	LVc	CCTGC
RADLV$RLV_03	713 (-)	0	100.00	LVc	CCTGC
AS$ARR_01	715 (+)	0	100.00	ARR1, ARR2	AGGATT
AS$NFKAPPAB_20	715 (-)	1	88.89	NF-kappaB	GGGGAATCCT
CHICK$CTNT_02	718 (+)	0	100.00	MCBF	ATTCCT
CHICK$CTNT_03	718 (+)	0	100.00	MCBF	ATTCCT
MOUSE$SAA3_02	718 (+)	1	87.50	C/EBPalpha, DBP	ATTGCTCCA
MOUSE$KROX_01	719 (+)	0	100.00	Elk-1, SAP-1a, SAP-1b, SRF	TTCCTC
MOUSE$FCGR3A_02	719 (+)	0	100.00	PU.1	TTCCTC
DROME$E74_10	719 (-)	0	100.00	E74A	AGGAA
DROME$E74_11	719 (-)	0	100.00	E74A	AGGAA
E74A$CONS_01	719 (-)	0	90.00	E74A	MGGAA
AS$LYF1_11	719 (-)	1	87.50	LyF-1	TTTGAGGAA
HS$UPA_01	719 (-)	0	100.00	c-Ets-2, PEA3	GGAGGAA
HT1$HTLV1_08	721 (+)	0	100.00	c-Ets-1 68	CCTCC
HS$CD8A_03	721 (+)	1	87.50	LyF-1	CCTCCCAAA
AS$PAX2_68	724 (+)	1	87.50	Pax-2.1, Pax-2.2	CCAAAATCA
RAT$PFK_04	726 (-)	1	88.89	HNF-6alpha, HNF-6beta, Tf-LF2	AAATCAATTT
Y$MAL61_03	726 (-)	0	100.00	MIG1	ATTTT
HS$P53_11	727 (+)	0	100.00	GAAP-1, IPCS-BF	AAATGATTTCCAC
HS$GMCSF_03	727 (-)	0	100.00	YY1	CATTT
MOUSE$IL4_01	727 (-)	0	100.00	NF-CLE0a, NF-CLE0b	TCATTT
RAT$BCAS_10	727 (-)	1	88.89	delta factor, YY1	GAAACCATTT
MOUSE$IGH_55	729 (-)	0	100.00		ATCAT
MOUSE$BMG_01	730 (+)	0	100.00	GATA-1	TGATT
HS$CYP3A4_05	730 (-)	1	87.50		TTGAAATCA
HS$HH4_01	731 (+)	0	100.00	H4TF-1	GATTTC
HS$INS_05	732 (-)	0	100.00		GGAAAT
HS$IGKL_01	733 (+)	0	100.00		TTTCCA
MOUSE$AFEP_11	733 (+)	0	100.00	C/EBPalpha, NP-Va	TTTCCAC
HS$IFI616_01	733 (-)	0	100.00	ISGF-3	GGAAA
RAT$INS1_01	733 (-)	0	100.00		GTGGAAA
MOUSE$IL5_02	733 (-)	0	100.00	NF-ATc, NF-ATp, NF-ATx	GGAAA
HS$IL4_01	733 (-)	0	100.00		GGAAA
MOUSE$IL4_06	733 (-)	0	100.00		GGAAA
MOUSE$IL4_07	733 (-)	0	100.00		GGAAA
MOUSE$IL4_08	733 (-)	0	100.00		GGAAA
HS$IL5_02	733 (-)	0	100.00		GGAAA
HS$IL13_01	733 (-)	0	100.00		GGAAA
MOUSE$CD40L_01	733 (-)	0	100.00		GGAAA
MOUSE$CD40L_02	733 (-)	0	100.00		GGAAA
MOUSE$CD40L_03	733 (-)	0	100.00		GGAAA
MOUSE$CD40L_04	733 (-)	0	100.00		GGAAA
HS$CD40L_01	733 (-)	0	100.00		GGAAA
HS$CD40L_02	733 (-)	0	100.00		GGAAA
HS$IFNG_03	733 (-)	0	100.00		GGAAA
HS$IFNG_04	733 (-)	0	100.00		GGAAA
HS$TNFA_05	733 (-)	0	100.00		GGAAA
MOUSE$MCP3_01	733 (-)	0	100.00		GGAAA
HS$TNFSF6_01	733 (-)	0	100.00		GGAAA
AS$STAT5A_36	734 (+)	1	87.50	STAT5A	TTCCACGAA
AS$STAT5A_40	734 (-)	1	87.50	STAT5A	TTCGTGGAA
HS$APOB_27	735 (+)	0	100.00		TCCACCAA
HT1$HTLV1_08	736 (+)	0	100.00	c-Ets-1 68	CCACC
HS$BG_52	736 (+)	0	100.00		CCACC
HS$BG_14	736 (+)	0	100.00		CCACC
HS$BG_44	736 (+)	0	100.00	CAC-binding protein	CCACC
BPV1$BPV1_24	736 (-)	0	100.00	Sp1	GGTGG
HS$BG_48	736 (-)	0	100.00	CAC-binding protein	GGTGG
MOUSE$CEBPA_06	736 (-)	0	100.00	Sp1	GGTGG
HS$BAC_03	739 (+)	0	100.00	SRF	CCAAT
AMULV$AMULV_01	739 (+)	0	100.00		CCAAT
EBV$DSL_06	739 (+)	0	100.00		CCAAT
MOUSE$AAG_04	739 (+)	0	100.00	alpha-CP1, alpha-CP2a, alpha-CP2b, alpha-IRP	CCAAT
CHICK$BAG_06	739 (+)	0	100.00	TGGCA-binding protein	CCAAT
HS$GG_15	739 (+)	0	100.00	NF-E	CCAAT
HS$GG_16	739 (+)	0	100.00	CDP2, Clox, CUTL1, Cutl1	CCAAT
HS$GG_17	739 (+)	0	100.00	gammaCAAT	CCAAT
HS$GG_18	739 (+)	0	100.00	CP1	CCAAT
HS$ZG_07	739 (+)	0	100.00	CP2	CCAAT
HS$ZG_08	739 (+)	0	100.00	CP1	CCAAT
HS$HH1_03	739 (+)	0	100.00	H1TF2	CCAAT
MOUSE$M2IAB_01	739 (+)	0	100.00	PU.1	CCAAT
PIG$UPA_12	739 (+)	0	100.00	NF-1	CCAAT
RADLV$RLV_10	739 (+)	0	100.00		CCAAT
MULV$MULV_03	739 (+)	0	100.00		CCAAT
MOUSE$GLUT4_04	739 (+)	0	100.00		CCAAT
MOMLV$MOMULV_15	739 (+)	0	100.00		CCAAT
RAT$ALDB_02	739 (+)	0	100.00	CBF (2), CP2	CCAAT
HS$GG_20	739 (+)	0	100.00	CP1, NF-E3	CCAAT
RAT$TH2B_01	739 (+)	0	100.00		CCAAT
RAT$TH2B_02	739 (+)	0	100.00		CCAAT
HS$GG_21	739 (+)	0	100.00	CP1	CCAAT
MOUSE$LY6E_01	739 (+)	0	100.00		CCAAT
RAT$NEU_01	739 (+)	0	100.00		CCAAT
HS$GHA_10	739 (+)	0	100.00	alpha-CBF	CCAAT
NEUCR$CPC1_02	739 (+)	0	100.00		CCAAT
HS$EG_07	739 (+)	0	100.00	CP1	CCAAT
HS$GP2B_13	739 (+)	0	100.00		CCAAT
MOUSE$BMG_10	739 (+)	0	100.00		CCAAT
HS$CYBH_01	739 (+)	0	100.00	CDP2, Clox, CP1, CUTL1, Cutl1	CCAAT
HBV$S_04	739 (+)	0	100.00		CCAAT
HS$FN_06	739 (+)	0	100.00		CCAAT
MOUSE$E2F1_02	739 (+)	0	100.00		CCAAT
HS$A24COL_01	739 (+)	0	100.00		CCAAT
MOUSE$EKLF_01	739 (+)	0	100.00	AP-1, GATA-1	CCAAT
MOUSE$A11COL_01	739 (-)	0	100.00	CBF (2), IF2	ATTGG
MOUSE$A21COL_03	739 (-)	0	100.00	CCAAT-binding factor	ATTGG
MOUSE$A21COL_04	739 (-)	0	100.00	CBF (2), CBF-A, CBF-B	ATTGG
MOUSE$A21COL_05	739 (-)	0	100.00	EFI	ATTGG
AD2$E2L_03	739 (-)	0	100.00	CRF	ATTGG
HS$GHA_06	739 (-)	0	100.00	NF-1	ATTGG
SP$H2B1_02	739 (-)	0	100.00	CBF (1)	ATTGG
SP$H2B1_03	739 (-)	0	100.00	CDF	ATTGG
HS$HSP70_02	739 (-)	0	100.00	CTF	ATTGG
HS$HSP70_07	739 (-)	0	100.00	CBTF, CP1, CTF	ATTGG
MOUSE$M2EAK_07	739 (-)	0	100.00	NF-Y	ATTGG
MOUSE$M2EAK_08	739 (-)	0	100.00	NF-Y	ATTGG
RSV$RSV_05	739 (-)	0	100.00		ATTGG
HSV1$TK_04	739 (-)	0	100.00	C/EBPalpha	ATTGG
HSV1$TK_05	739 (-)	0	100.00	NF-Y'	ATTGG
AS$HAP23_01	739 (-)	0	100.00	HAP2, HAP3, HAP4	ATTGG
MOUSE$M1H2KB_12	739 (-)	0	100.00	EFI	ATTGG
RAT$TH2A_02	739 (-)	0	100.00		ATTGG
RAT$TH2A_04	739 (-)	0	100.00		ATTGG
HS$TK_02	739 (-)	0	100.00	CP1	ATTGG
MOUSE$A11COL_07	739 (-)	0	100.00	NF-1, Sp1	ATTGG

MOUSE$A11COL_07	739 (-)	0	100.00	NF-1, Sp1	ATTGG
HS$CDC2_07	739 (-)	0	100.00	CBAF, CBF-B, CP1A	ATTGG
XENLA$GATA2_01	739 (-)	0	100.00	CBTF	ATTGG
RAT$NAT_05	739 (-)	0	100.00		ATTGG
HS$CDC2_12	739 (-)	0	100.00	CBAF	ATTGG
Y$MAL61_04	740 (-)	0	100.00	MIG1	AATTG
I$HSF_01	742 (-)	0	90.00	HSF	AGAAN
F$HSF_01	742 (-)	0	90.00	HSF	AGAAN
PA$PY_11	744 (+)	1	87.50		ACTGCCCTC
PA$PY_14	744 (+)	1	87.50		ACTGCCCTC
PA$PY_11	744 (-)	1	87.50		GAGGGCAGT
PA$PY_14	744 (-)	1	87.50		GAGGGCAGT
PIG$UPA_09	745 (-)	0	100.00	Sp1	GGGCAG
CHICK$BAG_03	746 (+)	0	100.00		TGCCC
MOUSE$CRABP2_01	746 (+)	0	100.00	RXR-alpha, RXR-gamma	TGCCCT
HS$CABD9_01	746 (+)	0	100.00	RXR-alpha, VDR	TGCCCT
RAT$VEGF_02	746 (+)	0	100.00	ER-alpha, ER-beta	TGCCC
CHICK$CYP2H1_05	746 (+)	0	100.00	CXR, RXR-gamma	TGCCCT
CHICK$BAG_03	746 (-)	0	100.00		GGGCA
RAT$NF1_01	746 (-)	0	100.00	LF-A1	GGGCA
AS$RAR_16	746 (-)	0	100.00	RAR-alpha1, RXR-alpha	AGGGCA
HS$CATHD_01	746 (-)	0	100.00	ER-alpha, Sp1	GGGCA
RAT$VEGF_01	746 (-)	0	100.00	ER-alpha, ER-beta	GGGCA
HS$ALDA_06	746 (-)	0	100.00	TR2-11	AGGGCA
CTCF$CONS	748 (+)	0	100.00	CTCF	CCCTC
MOUSE$PBGD_01	748 (+)	0	100.00	CAC-binding protein	CCCTCAC
RAT$POMC_03	757 (-)	0	100.00	GR	CAGAG
RAT$ARGL_03	759 (-)	0	100.00	NF-1	AGCCAG
AMV$AMV_02	763 (+)	0	100.00	C/EBPalpha	CTTGC
RAV0$RAV0_01	763 (-)	0	100.00	C/EBPalpha	GCAAG
MOUSE$SAA3_01	763 (-)	1	87.50	C/EBPalpha, DBP	TTATGCAAG
CHICK$VIT2_14	768 (+)	0	100.00	C/EBPalpha	AGAATTT
I$HSF_01	768 (+)	0	90.00	HSF	AGAAN
F$HSF_01	768 (+)	0	90.00	HSF	AGAAN
RAT$CYTOPB_03	769 (+)	1	87.50	NF-kappaB	GAAATTTCC
MOUSE$MIP1A_04	770 (+)	1	87.50	C/EBPalpha, C/EBPbeta	AATTTTCCC
Y$MAL61_03	771 (+)	0	100.00	MIG1	ATTTT
MOUSE$IL4_02	771 (+)	0	100.00	c-Maf, NF-ATp	ATTTTCC
HS$IL3_10	771 (-)	0	100.00	NF-ATp	TGGAAAAT
Y$GAL4_01	772 (+)	0	100.00	MIG1	TTTTC
MOUSE$IL4_04	772 (+)	0	100.00	NF-ATc, NF-ATp, NFAT-1	TTTTCC
NT$PR1A_03	772 (+)	0	100.00	GT-1	TTTTCC
MOUSE$IL2_01	772 (-)	0	100.00	NF-AT3, NF-ATc, NF-ATp, NF-ATx, NFAT-1, Pu box binding factor	GGAAAA
AS$SPZ1_03	772 (-)	1	87.50	Spz1	GGTGGTAAA
Y$RPT2_01	772 (-)	1	87.50	RPN4	GGTGGCAAA
Y$RPT5_01	772 (-)	1	87.50	RPN4	GGTGGCAAA
Y$RPT4_01	772 (-)	1	87.50	RPN4	GGTGGCAAA
Y$RPT6_01	772 (-)	1	87.50	RPN4	GGTGGCAAA
HS$IGKL_01	773 (+)	0	100.00		TTTCCA
MOUSE$AFEP_11	773 (+)	0	100.00	C/EBPalpha, NP-Va	TTTCCAC
HS$IFI616_01	773 (-)	0	100.00	ISGF-3	GGAAA
RAT$INS1_01	773 (-)	0	100.00		GTGGAAA
HS$GPC_05	773 (-)	1	90.00		AGGGGTGGAAA
MOUSE$IL5_02	773 (-)	0	100.00	NF-ATc, NF-ATp, NF-ATx	GGAAA
HS$IL4_01	773 (-)	0	100.00		GGAAA
MOUSE$IL4_06	773 (-)	0	100.00		GGAAA
MOUSE$IL4_07	773 (-)	0	100.00		GGAAA
MOUSE$IL4_08	773 (-)	0	100.00		GGAAA
HS$IL5_02	773 (-)	0	100.00		GGAAA
HS$IL13_01	773 (-)	0	100.00		GGAAA
MOUSE$CD40L_01	773 (-)	0	100.00		GGAAA
MOUSE$CD40L_02	773 (-)	0	100.00		GGAAA
MOUSE$CD40L_03	773 (-)	0	100.00		GGAAA
MOUSE$CD40L_04	773 (-)	0	100.00		GGAAA
HS$CD40L_01	773 (-)	0	100.00		GGAAA
HS$CD40L_02	773 (-)	0	100.00		GGAAA
HS$IFNG_03	773 (-)	0	100.00		GGAAA
HS$IFNG_04	773 (-)	0	100.00		GGAAA
HS$TNFA_05	773 (-)	0	100.00		GGAAA
MOUSE$MCP3_01	773 (-)	0	100.00		GGAAA
HS$TNFSF6_01	773 (-)	0	100.00		GGAAA
AS$ZIC2_15	774 (-)	1	87.50	Zic2	AGGGTGGAA
RAT$PTHRP_01	775 (-)	0	100.00	RXR-alpha, VDR	GGGTGGA
HT1$HTLV1_08	776 (+)	0	100.00	c-Ets-1 68	CCACC
HS$BG_52	776 (+)	0	100.00		CCACC
HS$BG_14	776 (+)	0	100.00		CCACC
HS$BG_44	776 (+)	0	100.00	CAC-binding protein	CCACC
HS$MIP_07	776 (+)	0	100.00	Sp1	CCACCC
MOUSE$P53_09	776 (+)	0	100.00	ETF	CCACCC
BPV1$BPV1_24	776 (-)	0	100.00	Sp1	GGGTGG
BPV1$BPV1_24	776 (-)	0	100.00	Sp1	GGTGG
HS$AG_12	776 (-)	0	100.00	CACCC-binding factor	GGGTGG
AS$RAR_07	776 (-)	0	100.00	RAR-alpha1, RXR-alpha	GGGTGG
HS$BG_48	776 (-)	0	100.00	CAC-binding protein	GGTGG
MOUSE$CEBPA_06	776 (-)	0	100.00	Sp1	GGTGG
HS$BG_01	777 (+)	0	100.00	CACCC-binding factor	CACCC
HS$GG_13	777 (+)	0	100.00	gammaCAC1, gammaCAC2	CACCC
HS$GG_14	777 (+)	0	100.00	CACCC-binding factor, Sp1	CACCC
HS$GP2B_01	777 (+)	0	100.00	CACCC-binding factor	CACCC
RAT$TOA_02	777 (+)	0	100.00	CACCC-binding factor	CACCC
HS$EG_06	777 (+)	0	100.00	Sp1	CACCC
AS$CACCC_01	777 (+)	0	100.00	CACCC-binding factor	CACCC
HS$AG_15	777 (-)	0	100.00	CAC-binding protein	GGGGTG
RAT$ME_01	778 (-)	0	100.00	T3R-alpha, T3R-beta1, T3R-beta2	TGGGGT
BPV1$BPV1_13	779 (+)	0	100.00	AP-2alphaA, AP-2alphaB	CCCCAA
BPV1$BPV1_13	779 (-)	0	100.00	AP-2alphaA, AP-2alphaB	TTGGGG
Y$ACS1_02	779 (-)	0	100.00	ADR1	TTGGGG
AS$MSX1_06	780 (+)	1	87.50	Msx-1	CCGAAAATG
AS$HOXA3_13	780 (-)	1	87.50	HOXA3	CATGTTGGG
Y$MAL61_03	783 (-)	0	100.00	MIG1	ATTTT
HS$GMCSF_03	784 (-)	0	100.00	YY1	CATTT
AS$FOXJ2_28	787 (-)	1	88.89	FOXJ2 (long isoform)	GATACAAACA
DROME$ADH_12	789 (-)	0	100.00		TACTAA
AS$GKLF_01	790 (+)	0	100.00	GKLF	TAGTAT
RAT$GHF1_01	791 (+)	0	100.00	PTF	AGTAT
RAT$GHF1_01	791 (-)	0	100.00	PTF	ATACT
HS$GPB_05	792 (+)	0	100.00	GATA-1	GTATCT
ASPN$NIAD_01	793 (+)	0	100.00	NIT2	TATCTA
NIT2$CONS_01	793 (+)	0	91.67	NIT2	TATCTM
MOUSE$GSHPX1_06	793 (+)	0	100.00	GATA-1	TATCT
MOUSE$GSHPX1_07	793 (+)	0	100.00	GATA-1	TATCT
MOUSE$GSHPX1_09	793 (+)	0	100.00	GATA-1	TATCT
F$NIT2_01	793 (+)	0	91.67	NIT2	TATCTM
MOUSE$BMG_02	793 (-)	0	100.00	GATA-1	AGATA
NEUCR$NIT3_02	793 (-)	0	100.00	NIT2	TAGATA
MAIZE$ADH2_03	796 (+)	0	100.00		CTACGG
EBV$IR4_05	800 (+)	0	100.00	R	GGCAC
EBV$IR4_06	800 (+)	0	100.00	R	GGCAC
XENLA$U2SN_11	800 (-)	0	100.00		GTGCC
DROME$ADH_34	802 (+)	1	87.50	FTZ-F1	CACAAGGTC
HS$VEGF_02	805 (+)	0	100.00		CAGGT
AS$RAR_09	806 (+)	0	100.00	RAR-alpha1, RXR-alpha	AGGTCG
HSV1$GD_01	808 (-)	0	100.00	ICP4	CCGAC
AT$COR15A_01	808 (-)	0	100.00	ANT, CBF1, CBF2, CBF3	CCGAC
AT$RD29B_01	808 (-)	0	100.00	CBF1	CCGAC
AT$RD29A_01	808 (-)	0	100.00	CBF1, DREB1A, DREB2A	CCGAC
AT$COR78_01	808 (-)	0	100.00	ANT, CBF1, CBF2, CBF3	CCGAC
AT$COR15B_01	808 (-)	0	100.00	CBF1, CBF2, CBF3	CCGAC
AS$SPZ1_25	811 (+)	1	87.50	Spz1	GGCGGGAAT
DROME$EVE_10	812 (-)	0	100.00	GAGA factor	CTCGC
DROME$KR_18	812 (-)	1	87.50	Tll	GATTCTTGC
HS$ARR_02	815 (+)	0	100.00	Crx	AGAATCC
I$HSF_01	815 (+)	0	90.00	HSF	AGAAN
F$HSF_01	815 (+)	0	90.00	HSF	AGAAN
AS$ARR_01	817 (-)	0	100.00	ARR1, ARR2	AGGATT
MOUSE$M1H2KB_11	818 (+)	0	100.00		ATCCTGA
MOUSE$P53_03	821 (+)	0	100.00		CTGACTCT
RAT$EAI_09	821 (-)	0	100.00		GTCAG
Y$HIS3_05	822 (+)	0	100.00	GCN4	TGACTC
Y$HIS4_08	822 (+)	0	100.00	GCN4	TGACTC
Y$HIS4_12	822 (+)	0	100.00	GCN4	TGACTC
HS$GMCSF_14	822 (+)	0	100.00	AP-1, c-Fos, c-Jun	TGACTCT
HS$CYP24_02	822 (+)	0	100.00	VDR	TGACTC
HS$P53_06	822 (+)	0	100.00	AP-1, c-Fos, c-Jun	TGACTCT
Y$HIS3_02	822 (-)	0	100.00	GCN4	GAGTCA
Y$ILV1_01	822 (-)	0	100.00	GCN4	GAGTCA
MOUSE$IL2_04	822 (-)	0	100.00	AP-1, c-Jun, Fra-1	AGAGTCA
PEA$PSL_02	822 (-)	0	100.00	TGA1a	GAGTCA
NT$CHN50_01	822 (-)	0	100.00	WRKY1, WRKY3, WRKY4	AGTCA
RAT$POMC_03	825 (-)	0	100.00	GR	CAGAG
AS$CETS1P54_15	826 (+)	1	88.89	c-Ets-1 54	TCTGGACCCT
MOUSE$MT1_01	828 (+)	0	100.00	Sp1	TGCAC
HS$MT2A_16	828 (+)	0	100.00	MTF-1	TGCACCC
MOUSE$MT1_07	828 (-)	0	100.00	MTF-1	GGGTGCA
HS$BG_01	830 (+)	0	100.00	CACCC-binding factor	CACCC
HS$GG_13	830 (+)	0	100.00	gammaCAC1, gammaCAC2	CACCC
HS$GG_14	830 (+)	0	100.00	CACCC-binding factor, Sp1	CACCC
HS$GP2B_01	830 (+)	0	100.00	CACCC-binding factor	CACCC
RAT$TOA_02	830 (+)	0	100.00	CACCC-binding factor	CACCC
HS$EG_06	830 (+)	0	100.00	Sp1	CACCC
AS$CACCC_01	830 (+)	0	100.00	CACCC-binding factor	CACCC
CHICK$BAG_07	831 (-)	1	87.50		GAGGAGGGG
AS$ZIC_24	831 (-)	1	87.50	Zic1	GAGGAGGGC
CTCF$CONS	832 (+)	0	100.00	CTCF	CCCTC
H4TF1$CONS	832 (-)	1	87.50	H4TF-1	GGGGGAGGG
HS$APOB_06	832 (-)	0	100.00	C/EBPalpha	GAGGAGGG
HT1$HTLV1_08	833 (+)	0	100.00	c-Ets-1 68	CCTCC
HS$PFKM_03	833 (-)	0	100.00		GGGGAGGAGG
HS$AACS_02	833 (-)	0	100.00	ARP-1	AGGAGG
HS$ZG_06	834 (+)	1	87.50	Sp1	CCCCTCCCC
HS$MIP_03	834 (-)	1	87.50	Sp1	GGGGAGGGG
AS$ZIC_12	835 (-)	1	87.50	Zic1	TGGGGAGTA
HT1$HTLV1_08	836 (+)	0	100.00	c-Ets-1 68	CCTCC
HS$CD8A_03	836 (+)	1	87.50	LyF-1	CCTCCCAAA
MOUSE$M1H2KB_07	838 (+)	0	100.00	MBP-1 (1), NF-kappaB1, NF-kappaB2, NF-kappaB2 precursor	TCCCCA
MOUSE$M1H2KB_02	838 (-)	0	100.00	AP-2alpha, AP-2alphaA, AP-2alphaB	TGGGGA
MOUSE$M1H2KB_07	838 (-)	0	100.00	MBP-1 (1), NF-kappaB1, NF-kappaB2, NF-kappaB2 precursor	TGGGGA
RAT$TS_01	838 (-)	1	88.89	AP-1, c-Fos	GAGTTGGTGA
HS$APOE_06	839 (+)	1	87.50		CCCCACCTC
BPV1$BPV1_13	839 (+)	0	100.00	AP-2alphaA, AP-2alphaB	CCCCAA
BPV1$BPV1_13	839 (-)	0	100.00	AP-2alphaA, AP-2alphaB	TTGGGG
RAT$CYTOP_03	839 (-)	1	87.50		GAGTTGGGT
Y$ACS1_02	839 (-)	0	100.00	ADR1	TTGGGG
RAT$ANTEN_01	840 (+)	0	100.00	C/EBPalpha, C/EBPbeta	CCCAAC
Y$CYC1_09	841 (-)	0	100.00	hap2, HAP2, HAP3	GTTGG
Y$CYC1_10	841 (-)	0	100.00	CP1A, HAP2	GTTGG
Y$CYC1_11	841 (-)	0	100.00	HAP3, NF-YA	GTTGG
HS$PR264_06	842 (+)	0	100.00	c-Myb	CAACT
RAT$BCAS_08	847 (+)	0	100.00	delta factor	CCATTT
RAT$BCAS_09	847 (+)	0	100.00	delta factor, STAT5	CCATTT
CF1$CONS_01	847 (-)	0	91.67	YY1	ANATGG
HS$GMCSF_03	848 (+)	0	100.00	YY1	CATTT
HS$INS_05	849 (-)	0	100.00		GGAAAT
Y$SUC2_05	849 (-)	0	100.00	MED8	AGGAAAT
HS$IFI616_01	850 (-)	0	100.00	ISGF-3	GGAAA
MOUSE$UPA_01	850 (-)	0	100.00	PEA3	AGGAAA
PEA3$CONS	850 (-)	0	91.67	c-Ets-1 54, c-Ets-1 68, c-Ets-2 58-64, PEA3	AGGAAR
MOUSE$IL5_02	850 (-)	0	100.00	NF-ATc, NF-ATp, NF-ATx	GGAAA
HS$IL4_01	850 (-)	0	100.00		GGAAA
MOUSE$IL4_06	850 (-)	0	100.00		GGAAA
MOUSE$IL4_07	850 (-)	0	100.00		GGAAA
MOUSE$IL4_08	850 (-)	0	100.00		GGAAA
HS$IL5_02	850 (-)	0	100.00		GGAAA
HS$IL13_01	850 (-)	0	100.00		GGAAA
MOUSE$CD40L_01	850 (-)	0	100.00		GGAAA
MOUSE$CD40L_02	850 (-)	0	100.00		GGAAA
MOUSE$CD40L_03	850 (-)	0	100.00		GGAAA
MOUSE$CD40L_04	850 (-)	0	100.00		GGAAA
HS$CD40L_01	850 (-)	0	100.00		GGAAA
HS$CD40L_02	850 (-)	0	100.00		GGAAA
HS$IFNG_03	850 (-)	0	100.00		GGAAA
HS$IFNG_04	850 (-)	0	100.00		GGAAA
HS$TNFA_05	850 (-)	0	100.00		GGAAA
MOUSE$MCP3_01	850 (-)	0	100.00		GGAAA
HS$TNFSF6_01	850 (-)	0	100.00		GGAAA
HS$ADH2_09	850 (-)	0	100.00	GR	AGGAAA
HS$CDC2_02	851 (+)	0	100.00	c-Ets-2	TTCCTT
DROME$E74_10	851 (-)	0	100.00	E74A	AGGAA
DROME$E74_11	851 (-)	0	100.00	E74A	AGGAA
E74A$CONS_01	851 (-)	0	90.00	E74A	MGGAA
HS$CDC2_01	851 (-)	0	100.00	c-Ets-2	AAGGAA
MOUSE$HOXC8_05	852 (+)	0	100.00		TCCTTTG
XENLA$SIAMOIS_01	852 (-)	1	87.50	LEF-1	ATCAAAGGA
HS$CYCD1_08	854 (+)	0	100.00	TCF-4E	CTTTGCT
MOUSE$IGH_58	857 (-)	1	88.89	c-Ets-1 68	GCAGGAAGCA
TCF2ALPHA$CONS	858 (-)	0	87.50	TCF-2alpha	SAGGAAGY
AS$ETS1_02	858 (-)	1	87.50	Ets-1 deltaVII, PU.1	GGAGGAAGT
XENLA$RPL14_01	859 (+)	0	100.00	HrpF, XrpFI	CTTCC
Y$TPI_01	859 (+)	0	100.00	GCR1	CTTCC
MOUSE$RPL32_01	859 (-)	0	100.00	f(alpha)-f(epsilon), HrpF, XrpFI	GGAAG
PEA3$CONS	859 (-)	0	91.67	c-Ets-1 54, c-Ets-1 68, c-Ets-2 58-64, PEA3	AGGAAR
HS$TNFA_02	859 (-)	0	100.00		GGAAG
HS$TNFA_03	859 (-)	0	100.00		GGAAG
MOUSE$GSHPX1_02	859 (-)	0	100.00	PU.1	GAGGAAG
MOUSE$KROX_01	860 (+)	0	100.00	Elk-1, SAP-1a, SAP-1b, SRF	TTCCTC
MOUSE$FCGR3A_02	860 (+)	0	100.00	PU.1	TTCCTC
DROME$E74_10	860 (-)	0	100.00	E74A	AGGAA
DROME$E74_11	860 (-)	0	100.00	E74A	AGGAA
E74A$CONS_01	860 (-)	0	90.00	E74A	MGGAA
HS$UPA_01	860 (-)	0	100.00	c-Ets-2, PEA3	GGAGGAA
HT1$HTLV1_08	862 (+)	0	100.00	c-Ets-1 68	CCTCC
HS$EGFR_19	868 (-)	0	100.00		GCCTGC
MOMLV$MOMULV_09	868 (-)	0	100.00	LVc	CCTGC
MOMLV$MOMULV_10	868 (-)	0	100.00	LVc	CCTGC
RADLV$RLV_03	868 (-)	0	100.00	LVc	CCTGC
HS$TPI_04	870 (+)	0	100.00	Sp1	AGGCGG
HS$CDC25C_04	871 (+)	0	100.00		GGCGG
Y$CHA1_01	872 (+)	0	100.00	CHA4	GCGGA
Y$CHA1_01	872 (-)	0	100.00	CHA4	TCCGC
I$BCD_01	873 (+)	0	87.50	Bcd	SGGATTAN
HS$HIOMTA_01	874 (+)	0	100.00	Crx	GGATTAC
SP$SPEC2A_02	874 (-)	0	100.00	SpOtx	TAATCC
AS$BCD_01	874 (-)	0	100.00	Bcd, PITX2	TAATCC
AMV$AMV_01	876 (+)	0	100.00	C/EBPalpha	ATTAC
FSV$FSV_01	876 (+)	0	100.00	C/EBPalpha	ATTAC
HS$P53_09	877 (+)	0	100.00	p53	TTACTTGCCCTTA
HS$TSHB_04	877 (-)	0	100.00	T3R-beta1	AAGTAA
AS$NKX3A_09	878 (-)	0	100.00	NKX3A	CAAGTA
AMV$AMV_02	880 (+)	0	100.00	C/EBPalpha	CTTGC
RAV0$RAV0_01	880 (-)	0	100.00	C/EBPalpha	GCAAG
CHICK$BAG_03	882 (+)	0	100.00		TGCCC
MAIZE$PEPC_01	882 (+)	0	100.00	MNB1b, MNF1	TGCCCTT
MOUSE$CRABP2_01	882 (+)	0	100.00	RXR-alpha, RXR-gamma	TGCCCT
HS$CABD9_01	882 (+)	0	100.00	RXR-alpha, VDR	TGCCCT
RAT$VEGF_02	882 (+)	0	100.00	ER-alpha, ER-beta	TGCCC
CHICK$CYP2H1_05	882 (+)	0	100.00	CXR, RXR-gamma	TGCCCT
CHICK$BAG_03	882 (-)	0	100.00		GGGCA
RAT$NF1_01	882 (-)	0	100.00	LF-A1	GGGCA
AS$RAR_16	882 (-)	0	100.00	RAR-alpha1, RXR-alpha	AGGGCA
HS$CATHD_01	882 (-)	0	100.00	ER-alpha, Sp1	GGGCA
RAT$VEGF_01	882 (-)	0	100.00	ER-alpha, ER-beta	GGGCA
HS$ALDA_06	882 (-)	0	100.00	TR2-11	AGGGCA
AS$AREB6_06	886 (+)	1	87.50	AREB6	CTTACCTGT
HS$TSHB_04	887 (-)	0	100.00	T3R-beta1	AAGTAA
AS$NKX3A_09	888 (-)	0	100.00	NKX3A	CAAGTA
DROME$HSP27_04	890 (+)	0	100.00	EcR	CTTGTC
Y$RFX1_02	890 (+)	1	91.67	RFX1	GTTGTCATGGCGA
DROME$HSP27_04	890 (-)	0	100.00	EcR	GACAAG
HS$GG_19	890 (-)	0	100.00	CP1, GATA-1, NFE-6	GACAAG
HS$GG_33	890 (-)	0	100.00	CP1, NFE-6	GACAAG
AS$MEIS1_04	891 (-)	0	100.00	Meis-1a, Meis-1b	TGACAA
AS$TGIF_01	892 (+)	0	100.00	TGIF	TGTCA
AS$TGIF_02	892 (+)	0	100.00	TGIF	TGTCA
AS$TGIF_03	892 (+)	0	100.00	TGIF	TGTCA
AS$TGIF_04	892 (+)	0	100.00	TGIF	TGTCA
AS$TGIF_05	892 (+)	0	100.00	TGIF	TGTCA
AS$TGIF_06	892 (+)	0	100.00	TGIF	TGTCA
AS$TGIF_07	892 (+)	0	100.00	TGIF	TGTCA
AS$TGIF_08	892 (+)	0	100.00	TGIF	TGTCA
AS$TGIF_10	892 (+)	0	100.00	TGIF	TGTCA
AS$TGIF_11	892 (+)	0	100.00	TGIF	TGTCA
AS$TGIF_12	892 (+)	0	100.00	TGIF	TGTCA
AS$TGIF_13	892 (+)	0	100.00	TGIF	TGTCA
AS$TGIF_14	892 (+)	0	100.00	TGIF	TGTCA
AS$TGIF_15	892 (+)	0	100.00	TGIF	TGTCA
POT$PR10a_01	892 (+)	0	100.00	PBF-1, PBF-2 (p24)	TGTCA
POT$PR10a_01	892 (-)	0	100.00	PBF-1, PBF-2 (p24)	TGACA
MOUSE$P53_04	893 (+)	2	91.30	NF-1	GTTATGGCGACTA
HS$P53_07	893 (+)	0	100.00	YY1	GTCATGGCGA
MOUSE$DAX1_02	894 (+)	1	87.50	SF-1	TCATGGCCA
CHICK$OA_05	895 (-)	0	100.00		CCATG
MULV$MULV_01	896 (-)	0	100.00	UCRF-L	CGCCAT
Y$PYK1_02	896 (-)	1	87.50	repressor of CAR1 expression	AGCCGCCAT
FACT1$CONS	897 (+)	0	100.00	F-ACT1	TGGCGA
HS$P53_08	897 (+)	0	100.00	NF-1	TGGCGACTGTCC
CHICK$AAC_06	898 (+)	0	100.00	delta factor, F-ACT1, MAPF2, YY1	GGCGAC
CHICK$AAC_07	898 (+)	0	100.00	YY1	GGCGAC
HS$GG_12	903 (+)	0	100.00	NF-E	CTGTC
DROME$EVE_10	903 (+)	0	100.00	GAGA factor	CTGTC
AS$MEIS1_13	903 (+)	0	100.00	Meis-1a, Meis-1b	CTGTCC
HS$CRH_01	904 (-)	0	100.00	GR	TGGACA
CHICK$STN1_01	907 (+)	0	100.00	LBP-1, MyoD	CCAGC
CHICK$STN1_03	907 (+)	0	100.00	LBP-1, NF-1 (-like proteins)	CCAGC
HS$PAI_07	908 (-)	0	100.00		AAGCTG
AS$FTZ_49	909 (-)	0	100.00	Ftz	AAAGCT
AS$SRY_01	911 (-)	0	100.00	SRY	CACAAAG
RAT$A2UG_09	914 (+)	0	100.00	GR	TGTGCC
RAT$RPK_02	914 (+)	0	100.00	Sp1	TGTGC
RAT$A2UG_08	914 (-)	0	100.00	GR	GGCACA
XENLA$U2SN_11	915 (+)	0	100.00		GTGCC
EBV$IR4_05	915 (-)	0	100.00	R	GGCAC
EBV$IR4_06	915 (-)	0	100.00	R	GGCAC
RAT$OMP_07	915 (-)	0	100.00		TGGCAC
CAMV$35SR_06	915 (-)	0	100.00	MNB1b	TGGCAC
HS$ALBU_03	916 (-)	0	100.00	NF-1/L	TGGCA
HS$RBP_01	916 (-)	0	100.00	NF-1/L	TGGCA
RAT$A1I3_02	916 (-)	0	100.00	NF-1	TGGCA
RAT$AFEP_11	916 (-)	0	100.00	GR, NF-1	TGGCA
MMTV$MMTV_43	916 (-)	0	100.00	NF-1	TGGCA
RAT$MLC_04	921 (+)	0	100.00	MAPF2, YY1	GGAGC
HS$GMCSF_10	921 (+)	0	100.00	NF-ATp	GGAGCC
HS$APOB_11	922 (+)	0	100.00	LF-A1	GAGCCT
Y$GAL1_10	923 (+)	0	100.00	GAL4	AGCCT
Y$CTA1_01	923 (+)	1	87.50	repressor of CAR1 expression	AGCCGCGCA
Y$G3PDH_01	923 (-)	1	87.50	repressor of CAR1 expression	TGCGCGGCT
SV$SV40_01	924 (+)	0	100.00	T-Ag	GCCTC
SV$SV40_02	924 (+)	0	100.00	T-Ag	GCCTC
SV$SV40_63	924 (+)	0	100.00	T-Ag	GCCTC
PA$PY_24	924 (-)	0	100.00	T-Ag	GAGGC
PA$PY_25	924 (-)	0	100.00	T-Ag	GAGGC
SV$SV40_02	924 (-)	0	100.00	T-Ag	GAGGC
AS$AP2_01	925 (+)	1	88.89	AP-2gamma	CCCTGCGGGG
DROME$EVE_10	926 (+)	0	100.00	GAGA factor	CTCGC
MOMLV$MOMULV_09	929 (-)	0	100.00	LVc	CCTGC
MOMLV$MOMULV_10	929 (-)	0	100.00	LVc	CCTGC
RADLV$RLV_03	929 (-)	0	100.00	LVc	CCTGC
MOUSE$THY1_05	930 (-)	1	87.50		CCACCCCTG
AS$VDR_04	931 (+)	0	100.00	VDR	AGGGG
Y$GLK1_02	931 (+)	0	100.00	MSN2, MSN4	AGGGG
LPV$LPV_03	936 (-)	0	100.00	Pu box binding factor	ATCAA
MOUSE$AAMY_07	939 (+)	0	100.00	PTF1-beta	ATGGGA
AS$IK_05	940 (+)	0	100.00	Ik-1, Ik-2	TGGGATT
HS$CDH1_01	940 (+)	0	100.00	LUN-1	TGGGA
HS$TLN_01	940 (+)	0	100.00	LUN-1	TGGGA
HS$CDH1_01	940 (-)	0	100.00	LUN-1	TCCCA
HS$TLN_01	940 (-)	0	100.00	LUN-1	TCCCA
AS$NFKAPPAB65_17	942 (+)	1	93.33	RelA	GGAGTGGGGTTTT
HS$HH1_02	943 (+)	0	100.00	H1TF1, HiNF-B	GATTGG
HS$HSP70_06	943 (+)	0	100.00	CCAAT-binding factor	GATTGG
HS$HSP70_09	943 (+)	0	100.00	CP1, CTF	GATTGG
MOUSE$A11COL_01	944 (+)	0	100.00	CBF (2), IF2	ATTGG
MOUSE$A21COL_03	944 (+)	0	100.00	CCAAT-binding factor	ATTGG
MOUSE$A21COL_04	944 (+)	0	100.00	CBF (2), CBF-A, CBF-B	ATTGG
MOUSE$A21COL_05	944 (+)	0	100.00	EFI	ATTGG
AD2$E2L_03	944 (+)	0	100.00	CRF	ATTGG
HS$GHA_06	944 (+)	0	100.00	NF-1	ATTGG
SP$H2B1_02	944 (+)	0	100.00	CBF (1)	ATTGG
SP$H2B1_03	944 (+)	0	100.00	CDF	ATTGG
HS$HSP70_02	944 (+)	0	100.00	CTF	ATTGG
HS$HSP70_07	944 (+)	0	100.00	CBTF, CP1, CTF	ATTGG
MOUSE$M2EAK_07	944 (+)	0	100.00	NF-Y	ATTGG
MOUSE$M2EAK_08	944 (+)	0	100.00	NF-Y	ATTGG
RSV$RSV_05	944 (+)	0	100.00		ATTGG
HSV1$TK_04	944 (+)	0	100.00	C/EBPalpha	ATTGG
HSV1$TK_05	944 (+)	0	100.00	NF-Y'	ATTGG
AS$HAP23_01	944 (+)	0	100.00	HAP2, HAP3, HAP4	ATTGG
MOUSE$M1H2KB_12	944 (+)	0	100.00	EFI	ATTGG
RAT$TH2A_02	944 (+)	0	100.00		ATTGG
RAT$TH2A_04	944 (+)	0	100.00		ATTGG
HS$TK_02	944 (+)	0	100.00	CP1	ATTGG
MOUSE$A11COL_07	944 (+)	0	100.00	NF-1, Sp1	ATTGG
HS$CDC2_07	944 (+)	0	100.00	CBAF, CBF-B, CP1A	ATTGG
XENLA$GATA2_01	944 (+)	0	100.00	CBTF	ATTGG
RAT$NAT_05	944 (+)	0	100.00		ATTGG
HS$CDC2_12	944 (+)	0	100.00	CBAF	ATTGG
HS$BAC_03	944 (-)	0	100.00	SRF	CCAAT
AMULV$AMULV_01	944 (-)	0	100.00		CCAAT
EBV$DSL_06	944 (-)	0	100.00		CCAAT
MOUSE$AAG_04	944 (-)	0	100.00	alpha-CP1, alpha-CP2a, alpha-CP2b, alpha-IRP	CCAAT
CHICK$BAG_06	944 (-)	0	100.00	TGGCA-binding protein	CCAAT
HS$GG_15	944 (-)	0	100.00	NF-E	CCAAT
HS$GG_16	944 (-)	0	100.00	CDP2, Clox, CUTL1, Cutl1	CCAAT
HS$GG_17	944 (-)	0	100.00	gammaCAAT	CCAAT
HS$GG_18	944 (-)	0	100.00	CP1	CCAAT
HS$ZG_07	944 (-)	0	100.00	CP2	CCAAT
HS$ZG_08	944 (-)	0	100.00	CP1	CCAAT
HS$HH1_03	944 (-)	0	100.00	H1TF2	CCAAT
MOUSE$M2IAB_01	944 (-)	0	100.00	PU.1	CCAAT
PIG$UPA_12	944 (-)	0	100.00	NF-1	CCAAT
RADLV$RLV_10	944 (-)	0	100.00		CCAAT
MULV$MULV_03	944 (-)	0	100.00		CCAAT
MOUSE$GLUT4_04	944 (-)	0	100.00		CCAAT
MOMLV$MOMULV_15	944 (-)	0	100.00		CCAAT
RAT$ALDB_02	944 (-)	0	100.00	CBF (2), CP2	CCAAT
HS$GG_20	944 (-)	0	100.00	CP1, NF-E3	CCAAT
RAT$TH2B_01	944 (-)	0	100.00		CCAAT
RAT$TH2B_02	944 (-)	0	100.00		CCAAT
HS$GG_21	944 (-)	0	100.00	CP1	CCAAT
MOUSE$LY6E_01	944 (-)	0	100.00		CCAAT
RAT$NEU_01	944 (-)	0	100.00		CCAAT
HS$GHA_10	944 (-)	0	100.00	alpha-CBF	CCAAT
NEUCR$CPC1_02	944 (-)	0	100.00		CCAAT
HS$EG_07	944 (-)	0	100.00	CP1	CCAAT
HS$GP2B_13	944 (-)	0	100.00		CCAAT
MOUSE$BMG_10	944 (-)	0	100.00		CCAAT
HS$CYBH_01	944 (-)	0	100.00	CDP2, Clox, CP1, CUTL1, Cutl1	CCAAT
HBV$S_04	944 (-)	0	100.00		CCAAT
HS$FN_06	944 (-)	0	100.00		CCAAT
MOUSE$E2F1_02	944 (-)	0	100.00		CCAAT
HS$A24COL_01	944 (-)	0	100.00		CCAAT
MOUSE$EKLF_01	944 (-)	0	100.00	AP-1, GATA-1	CCAAT
BPV1$BPV1_13	945 (+)	0	100.00	AP-2alphaA, AP-2alphaB	TTGGGG
Y$ACS1_02	945 (+)	0	100.00	ADR1	TTGGGG
BPV1$BPV1_13	945 (-)	0	100.00	AP-2alphaA, AP-2alphaB	CCCCAA
RAT$ME_01	946 (+)	0	100.00	T3R-alpha, T3R-beta1, T3R-beta2	TGGGGT
HS$IL6_02	947 (+)	1	90.00	NF-kappaB(-like)	GGGATTTTCCC
HS$SELE_01	947 (+)	1	88.89	NF-kappaB	GGGGATTTCC
DORSAL$CONS_02	947 (+)	1	88.89	Dl	GGGTTTTTCC
DL$CONS_01	947 (+)	1	88.89	Dl	GGGTTTTTCC
AS$NFKAPPAB_02	947 (+)	1	88.89	NF-kappaB	GGGGATTTCC
AS$NFKAPPAB_03	947 (+)	1	88.89	NF-kappaB	GGGGTTTCCC
AS$NFKAPPAB_11	947 (+)	1	88.89	NF-kappaB	GGGGATTTCC
AS$NFKAPPAB_13	947 (+)	1	88.89	NF-kappaB	GGGATTTTCC
AS$NFKAPPAB_18	947 (+)	1	88.89	NF-kappaB	GGGGCTTTCC
AS$NFKAPPAB_35	947 (+)	1	88.89	NF-kappaB	GGGGATTTCC
AS$NFKAPPAB_38	947 (+)	1	88.89	NF-kappaB	GGGGCTTTCC
MOUSE$INOS_01	947 (+)	1	88.89		GGGATTTTCC
HS$P53_10	947 (+)	0	100.00		GGGGTTTTTCCC
DROME$ZEN_04	948 (+)	1	88.89	Dl	TGGTTTTTCC
DORSAL$CONS_02	948 (+)	1	88.89	Dl	GGGTTTTTCC
DROME$SNA_01	948 (+)	0	100.00	Dl	GGGTTTTTCC
DL$CONS_01	948 (+)	1	88.89	Dl	GGGTTTTTCC
AS$DL_08	948 (+)	1	88.89	Dl	GGGTTTTCCA
AS$NFKAPPAB_03	948 (+)	1	88.89	NF-kappaB	GGGGTTTCCC
AS$NFKAPPAB_27	948 (+)	1	88.89	NF-kappaB	GGGATTTCCC
AS$ZIC2_28	948 (+)	1	87.50	Zic2	GGGTTTGCC
HS$P53_05	948 (+)	0	100.00	NF-kappaB, RelA	GGGTTTTCC
MOUSE$AACR_04	948 (-)	1	88.89	alphaA-CRYBP1	GGGAAATCCC
DROME$TWI_03	948 (-)	1	88.89	Dl	GAGAAAACCC
AACRYBP1$CONS_01	948 (-)	1	88.89	alphaA-CRYBP1	GGGAAATCCC
DROME$TWI_08	948 (-)	1	90.00	Dl	GGGCAAAACCC
AS$NFKAPPAB_30	948 (-)	0	100.00	NF-kappaB	GGGAAAACCC
DL$CONS_02	949 (-)	0	88.89	Dl	GRGAAAANC
Y$GAL4_01	951 (+)	0	100.00	MIG1	TTTTC
MOUSE$IL4_04	951 (+)	0	100.00	NF-ATc, NF-ATp, NFAT-1	TTTTCC
NT$PR1A_03	951 (+)	0	100.00	GT-1	TTTTCC
HS$HSP70_08	951 (-)	1	88.89		GAAGGGAAAA
MOUSE$IL2_01	951 (-)	0	100.00	NF-AT3, NF-ATc, NF-ATp, NF-ATx, NFAT-1, Pu box binding factor	GGAAAA
HS$IFI616_01	952 (-)	0	100.00	ISGF-3	GGAAA
RAT$IGF1R_06	952 (-)	1	87.50	WT1 -KTS, WT1 I -KTS	GAGGGGGAA
RAT$IGF1R_10	952 (-)	1	87.50	WT1 -KTS, WT1 I -KTS	GAGGGGGAA
MOUSE$IL5_02	952 (-)	0	100.00	NF-ATc, NF-ATp, NF-ATx	GGAAA
HS$IL4_01	952 (-)	0	100.00		GGAAA
MOUSE$IL4_06	952 (-)	0	100.00		GGAAA
MOUSE$IL4_07	952 (-)	0	100.00		GGAAA
MOUSE$IL4_08	952 (-)	0	100.00		GGAAA
HS$IL5_02	952 (-)	0	100.00		GGAAA
HS$IL13_01	952 (-)	0	100.00		GGAAA
MOUSE$CD40L_01	952 (-)	0	100.00		GGAAA
MOUSE$CD40L_02	952 (-)	0	100.00		GGAAA
MOUSE$CD40L_03	952 (-)	0	100.00		GGAAA
MOUSE$CD40L_04	952 (-)	0	100.00		GGAAA
HS$CD40L_01	952 (-)	0	100.00		GGAAA
HS$CD40L_02	952 (-)	0	100.00		GGAAA
HS$IFNG_03	952 (-)	0	100.00		GGAAA
HS$IFNG_04	952 (-)	0	100.00		GGAAA
HS$TNFA_05	952 (-)	0	100.00		GGAAA
MOUSE$MCP3_01	952 (-)	0	100.00		GGAAA
HS$TNFSF6_01	952 (-)	0	100.00		GGAAA
AS$SPZ1_06	953 (-)	1	87.50	Spz1	GGAGGGAAA
MZF1$CONS_01	954 (-)	0	92.86	MZF-1	GNGGGGA
MOUSE$AQP7_01	954 (-)	0	100.00	PPAR-alpha, PPAR-gamma, RXR-alpha	AGGGGA
V$MZF1_01	954 (-)	0	87.50	MZF1	NGNGGGGA
HS$ZG_06	955 (+)	1	87.50	Sp1	CCCCTCCCC
HS$NPY_04	955 (+)	0	100.00	Sp1	CCCCTCC
MOUSE$P53_07	955 (+)	0	100.00	ETF	CCCCTCCC
HS$MIP_06	955 (+)	0	100.00	Sp1, Sp3	CCCCTCCC
RAT$CYP2D5_01	955 (+)	0	100.00	Sp1	CCCCTCCC
HS$MIP_03	955 (-)	1	87.50	Sp1	GGGGAGGGG
AS$VDR_04	955 (-)	0	100.00	VDR	AGGGG
Y$GLK1_02	955 (-)	0	100.00	MSN2, MSN4	AGGGG
CTCF$CONS	956 (+)	0	100.00	CTCF	CCCTC
MOUSE$THY1_04	956 (+)	0	100.00	R1, R2, Sp1	CCCTCCC
MOUSE$CMYC_04	956 (-)	0	100.00	MAZ	GGGAGGG
MOUSE$CD4_04	956 (-)	0	100.00	MAZ	GGGAGGG
HT1$HTLV1_08	957 (+)	0	100.00	c-Ets-1 68	CCTCC
MOUSE$P53_08	957 (+)	1	92.86		CCTCCCACGTGCT
HS$CD8A_02	957 (+)	1	87.50	LyF-1	CCTCCCAAG
HS$APOB_10	958 (-)	0	100.00	AP-2alphaA, AP-2alphaB	TGGGAG
HS$CDH1_01	959 (+)	0	100.00	LUN-1	TCCCA
HS$TLN_01	959 (+)	0	100.00	LUN-1	TCCCA
MOUSE$AAMY_07	959 (-)	0	100.00	PTF1-beta	ATGGGA
HS$CDH1_01	959 (-)	0	100.00	LUN-1	TGGGA
HS$TLN_01	959 (-)	0	100.00	LUN-1	TGGGA
CHICK$OA_05	961 (+)	0	100.00		CCATG
CF1$CONS_01	961 (-)	0	91.67	YY1	ANATGG
MOUSE$CMYC_05	961 (-)	0	100.00	YY1	ACATGG
MOUSE$IGH_10	962 (+)	0	100.00	muEBP-C2, TFE3-S	CATGTG
MOUSE$IGKL_10	962 (+)	0	100.00	muEBP-C2	CATGTG
XENLA$TFIIIA_02	962 (+)	1	87.50	USF1	CACGTGCTC
HS$P53_01	962 (+)	0	100.00		CATGTG
HS$P53_02	962 (+)	0	100.00		CATGTG
RAT$FAS_03	962 (+)	0	100.00	SREBP-1, USF-1, USF2	CATGTG
MOUSE$TBX2_01	962 (+)	0	100.00	Mitf, USF-1	CATGTG
HS$CDC25A_03	962 (+)	0	100.00	c-Myc, Max	CATGTG
HS$CDC25A_04	962 (+)	0	100.00	c-Myc, Max	CATGTG
RAT$ADH1_01	962 (-)	0	100.00	USF-1	CACATG
AD$MLP_40	962 (-)	0	100.00	USF1	CACATG
RAT$RPK_02	964 (+)	0	100.00	Sp1	TGTGC
RAT$A2UG_08	964 (-)	0	100.00	GR	AGCACA
RAT$A1I3_04	965 (+)	0	100.00		GTGCT
RAT$A1I3_04	965 (-)	0	100.00		AGCAC
RAT$VEGF_02	966 (-)	0	100.00	ER-alpha, ER-beta	GAGCA
HS$KAI1_01	967 (+)	1	90.00	p53	GCTCAAGCCTG
TDNA$NOS_01	967 (-)	0	100.00	ASF-1, OBF3.1, TGA1a, TGA1b	TGAGC
HS$BG_40	973 (+)	0	100.00	CP2	GACTGGC
CHICK$STN1_02	974 (-)	0	100.00	LBP-1, NF-1 (-like proteins)	CCAGT
HS$PAI_07	974 (-)	0	100.00		CCAGT
MOUSE$POLA1_02	974 (-)	0	100.00	E2F-1	GCGCCAGT
HS$H2A1_01	977 (-)	1	87.50	E2F-1	TTTCGCGCC
HS$E2F2_05	978 (+)	0	100.00	E2F	GCGCTAA
HS$P107_01	978 (-)	1	87.50	E2F	TTTTCGCGC
Y$MAL2R_01	982 (-)	0	100.00	MIG1	TTTTA
AS$FOXJ1_04	982 (-)	0	100.00	FOXJ1	TTTTA
MOUSE$CRISP3_01	986 (-)	0	100.00	AR	AAAACT
DROME$UBX_24	989 (+)	0	100.00	LEF-1	TTTGA
TDNA$NOS_01	991 (+)	0	100.00	ASF-1, OBF3.1, TGA1a, TGA1b	TGAGC
MOUSE$GSHPX1_01	995 (+)	0	100.00	PU.1	CTTCTC
I$HSF_01	995 (-)	0	90.00	HSF	AGAAN
F$HSF_01	995 (-)	0	90.00	HSF	AGAAN
VAV$VAVI_01	996 (+)	1	87.50	VITF	TTCTCAAAT
AS$STAT_09	996 (+)	1	87.50	STAT6	TTCTCAGAA
AS$STAT5A_37	996 (+)	1	87.50	STAT5A	TTCTGAGAA
HS$P21WAF1_10	996 (-)	1	87.50	STAT5B	TTCTGAGAA
DROME$UBX_24	999 (-)	0	100.00	LEF-1	TTTGA
DROME$KNI_05	1001 (+)	1	87.50	Tll	AAAAGTCAA
HS$PDGFB_03	1012 (-)	1	88.89		GGACGGTGGG
HT1$HTLV1_08	1013 (+)	0	100.00	c-Ets-1 68	CCACC
HS$BG_52	1013 (+)	0	100.00		CCACC
HS$BG_14	1013 (+)	0	100.00		CCACC
HS$BG_44	1013 (+)	0	100.00	CAC-binding protein	CCACC
BPV1$BPV1_24	1013 (-)	0	100.00	Sp1	GGTGG
HS$BG_48	1013 (-)	0	100.00	CAC-binding protein	GGTGG
MOUSE$CEBPA_06	1013 (-)	0	100.00	Sp1	GGTGG
HS$TERT_01	1014 (+)	0	100.00	c-Myc	CACCGT
PARS$PAL_04	1016 (+)	0	100.00		CCGTCC
HS$CS1_04	1022 (+)	0	100.00	T3R	AGGGAG
HS$P21WAF1_05	1022 (+)	0	100.00	RXR-alpha, VDR	AGGGAG
AS$VDR_08	1022 (+)	0	100.00	VDR	AGGGAG
RAT$GSTP_07	1024 (+)	1	87.50	NF-1A, SF-A	GGAGCAGGA
RAT$MLC_04	1024 (+)	0	100.00	MAPF2, YY1	GGAGC
RAT$VEGF_02	1025 (+)	0	100.00	ER-alpha, ER-beta	GAGCA
MOMLV$MOMULV_09	1027 (-)	0	100.00	LVc	CCTGC
MOMLV$MOMULV_10	1027 (-)	0	100.00	LVc	CCTGC
RADLV$RLV_03	1027 (-)	0	100.00	LVc	CCTGC
HS$VEGF_02	1028 (+)	0	100.00		CAGGT
TO$E4_02	1030 (-)	0	100.00		CTACC
DROME$ADH_06	1034 (+)	0	100.00	Adf-1	GCTGC
HS$CMYC_16	1034 (+)	0	100.00	CTCF	GCTGC
DROME$ADH_14	1034 (-)	0	100.00	Adf-1	GCAGC
RAT$ATP1A2_01	1035 (+)	1	87.50		CAGCTGGGC
MOUSE$M2EAK_03	1036 (-)	0	100.00	NF-Y	CAGCA
CHICK$BG_05	1036 (-)	0	100.00	AP-2	GCCCAGCA
CHICK$STN1_01	1037 (-)	0	100.00	LBP-1, MyoD	CCAGC
CHICK$STN1_03	1037 (-)	0	100.00	LBP-1, NF-1 (-like proteins)	CCAGC
SV$SV40_37	1038 (+)	0	100.00		CTGGG
SV$SV40_63	1039 (+)	0	100.00	T-Ag	TGGGC
HS$GMCSF_10	1041 (-)	0	100.00	NF-ATp	GGAGCC
AS$STAT5A_39	1041 (-)	1	87.50	STAT5A	CCCGGAGCA
RAT$MLC_04	1042 (-)	0	100.00	MAPF2, YY1	GGAGC
AS$STAT5A_69	1043 (-)	1	87.50	STAT5A	TACCCGGAG
MAIZE$ADH1P_02	1045 (-)	0	100.00		CCCCGG
Y$MAL2R_01	1045 (-)	0	100.00	MIG1	CCCCGG
Y$MAL63_01	1045 (-)	0	100.00	MIG1	CCCCGG
AS$CF1_17	1047 (+)	1	87.50	CF1	GGGGTCACT
EBV$IR4_04	1048 (+)	0	100.00	R	GGGAC
EBV$IR4_05	1048 (+)	0	100.00	R	GGGAC
AS$PR_01	1048 (+)	0	100.00	PR B	GGGACA
RAT$A2UG_10	1048 (-)	0	100.00	GR	TGTCCC
MAIZE$C1_05	1050 (-)	0	100.00		GTGTC
RAT$MT1_04	1057 (+)	1	87.50		TGCGCTCGG
Y$MEL1_01	1061 (+)	0	100.00	GAL4	TTCGG
Y$MEL1_01	1061 (-)	0	100.00	GAL4	CCGAA
HS$TERT_05	1063 (-)	1	87.50	Sp1	CCCAGCCCC
HS$TERT_06	1063 (-)	1	87.50	Sp1	CCCAGCCCC
HS$CYCD1_15	1063 (-)	0	100.00	Sp1, Sp2, Sp3, Sp4	GCCCG
HS$MIP_04	1065 (+)	0	100.00	Sp1	GGCTGGG
QUAIL$STN1_02	1065 (-)	0	100.00		CCAGCC
CHICK$STN1_01	1066 (-)	0	100.00	LBP-1, MyoD	CCAGC
CHICK$STN1_03	1066 (-)	0	100.00	LBP-1, NF-1 (-like proteins)	CCAGC
SV$SV40_37	1067 (+)	0	100.00		CTGGG
RAT$AMGL_02	1067 (+)	0	100.00	IL-6 RE-BP, STAT1, STAT5A, STAT5B, STAT6	CTGGGA
HS$APOB_10	1068 (+)	0	100.00	AP-2alphaA, AP-2alphaB	TGGGAG
HS$CDH1_01	1068 (+)	0	100.00	LUN-1	TGGGA
HS$TLN_01	1068 (+)	0	100.00	LUN-1	TGGGA
HS$CDH1_01	1068 (-)	0	100.00	LUN-1	TCCCA
HS$TLN_01	1068 (-)	0	100.00	LUN-1	TCCCA
DROME$EVE_11	1069 (-)	0	100.00	GAGA factor	CGCTCCC
RAT$MLC_04	1070 (+)	0	100.00	MAPF2, YY1	GGAGC
DROME$EVE_04	1071 (-)	0	100.00	GAGA factor	CGCTC
DROME$EVE_08	1071 (-)	0	100.00	GAGA factor	CGCTC
DROME$EVE_10	1071 (-)	0	100.00	GAGA factor	CGCTC
AS$AHRARNT_50	1073 (+)	0	100.00	AhR, Arnt	GCGTG
RAT$CYTOP_04	1073 (-)	0	100.00	AhR, Arnt	CACGC
RAT$A1I3_04	1075 (+)	0	100.00		GTGCT
RAT$A1I3_04	1075 (-)	0	100.00		AGCAC
AS$PAX2_63	1076 (-)	1	87.50	Pax-2.1, Pax-2.2	TGGAAAGCT
HCMV$IE1_16	1078 (+)	0	100.00	NF-1	CTTTCC
SV$SV40_25	1078 (+)	0	100.00		CTTTCCA
SV$SV40_42	1078 (-)	0	100.00		TGGAAAG
HS$IGKL_01	1079 (+)	0	100.00		TTTCCA
MOUSE$AFEP_11	1079 (+)	0	100.00	C/EBPalpha, NP-Va	TTTCCAC
HS$IFI616_01	1079 (-)	0	100.00	ISGF-3	GGAAA
RAT$INS1_01	1079 (-)	0	100.00		GTGGAAA
MOUSE$IL5_02	1079 (-)	0	100.00	NF-ATc, NF-ATp, NF-ATx	GGAAA
HS$IL4_01	1079 (-)	0	100.00		GGAAA
MOUSE$IL4_06	1079 (-)	0	100.00		GGAAA
MOUSE$IL4_07	1079 (-)	0	100.00		GGAAA
MOUSE$IL4_08	1079 (-)	0	100.00		GGAAA
HS$IL5_02	1079 (-)	0	100.00		GGAAA
HS$IL13_01	1079 (-)	0	100.00		GGAAA
MOUSE$CD40L_01	1079 (-)	0	100.00		GGAAA
MOUSE$CD40L_02	1079 (-)	0	100.00		GGAAA
MOUSE$CD40L_03	1079 (-)	0	100.00		GGAAA
MOUSE$CD40L_04	1079 (-)	0	100.00		GGAAA
HS$CD40L_01	1079 (-)	0	100.00		GGAAA
HS$CD40L_02	1079 (-)	0	100.00		GGAAA
HS$IFNG_03	1079 (-)	0	100.00		GGAAA
HS$IFNG_04	1079 (-)	0	100.00		GGAAA
HS$TNFA_05	1079 (-)	0	100.00		GGAAA
MOUSE$MCP3_01	1079 (-)	0	100.00		GGAAA
HS$TNFSF6_01	1079 (-)	0	100.00		GGAAA
AS$STAT5A_36	1080 (+)	1	87.50	STAT5A	TTCCACGAA
AS$STAT5A_70	1080 (+)	1	87.50	STAT5A	TTCCCCGAC
AS$STAT5A_40	1080 (-)	1	87.50	STAT5A	TTCGTGGAA
MAIZE$ADH1P_01	1082 (-)	0	100.00		CGTGG
MAIZE$ADH1P_03	1082 (-)	0	100.00		CGTGG
DROME$ADH_06	1085 (-)	0	100.00	Adf-1	CGTCG
HS$TERT_01	1087 (-)	0	100.00	c-Myc	CACCGT
Y$ACT1_01	1088 (-)	1	87.50	REB1	CTGTCACCG
HS$APOA2_06	1089 (-)	0	100.00		TCACC
HS$P53_03	1090 (+)	0	100.00	Pax-2, Pax-5, Pax-8	GTGAC
HS$MMP1_06	1090 (-)	0	100.00		GTCAC
PEA$IAA45_07	1090 (-)	0	100.00	ARF1	TGTCAC
AS$MEIS1_02	1091 (+)	0	100.00	Meis-1a, Meis-1b	TGACAC
AS$MEIS1BHOXA9_10	1091 (+)	0	100.00	HOXA9, Meis-1b	TGACAC
AS$MEIS1BHOXA9_12	1091 (+)	0	100.00	HOXA9, Meis-1b	TGACAC
AS$MEIS1BHOXA9_13	1091 (+)	0	100.00	HOXA9, Meis-1b	TGACAC
AS$MEIS1BHOXA9_14	1091 (+)	0	100.00	HOXA9, Meis-1b	TGACAC
POT$PR10a_01	1091 (+)	0	100.00	PBF-1, PBF-2 (p24)	TGACA
CHICK$OA_02	1091 (-)	0	100.00	ARP-1, COUP, COUP-TF1, PPAR-alpha, RXR-alpha	GTGTCA
AS$TGIF_01	1091 (-)	0	100.00	TGIF	TGTCA
AS$TGIF_02	1091 (-)	0	100.00	TGIF	TGTCA
AS$TGIF_03	1091 (-)	0	100.00	TGIF	TGTCA
AS$TGIF_04	1091 (-)	0	100.00	TGIF	TGTCA
AS$TGIF_05	1091 (-)	0	100.00	TGIF	TGTCA
AS$TGIF_06	1091 (-)	0	100.00	TGIF	TGTCA
AS$TGIF_07	1091 (-)	0	100.00	TGIF	TGTCA
AS$TGIF_08	1091 (-)	0	100.00	TGIF	TGTCA
AS$TGIF_10	1091 (-)	0	100.00	TGIF	TGTCA
AS$TGIF_11	1091 (-)	0	100.00	TGIF	TGTCA
AS$TGIF_12	1091 (-)	0	100.00	TGIF	TGTCA
AS$TGIF_13	1091 (-)	0	100.00	TGIF	TGTCA
AS$TGIF_14	1091 (-)	0	100.00	TGIF	TGTCA
AS$TGIF_15	1091 (-)	0	100.00	TGIF	TGTCA
POT$PR10a_01	1091 (-)	0	100.00	PBF-1, PBF-2 (p24)	TGTCA
MAIZE$C1_05	1092 (-)	0	100.00		GTGTC
RICE$EM_03	1092 (-)	1	88.89	TRAB1	GACGCGTGTC
RAT$CYTOP_04	1094 (+)	0	100.00	AhR, Arnt	CACGC
AS$AHRARNT_50	1094 (-)	0	100.00	AhR, Arnt	GCGTG

Number of sites found: 2098

Start a new search

RB2 P130

JASPAR RARA/RXRA MOTIF

Access EPDnew
- *H. sapiens*
- *H. sapiens non-coding*
- *M. mulatta*
- *M. musculus*
- *M. musculus non-coding*
- *R. norvegicus*
- *C. familiaris*
- *G. gallus*
- *D. melanogaster*
- *A. mellifera*
- *D. rerio*
- *C. elegans*
- *A. thaliana*
- *Z. mays*
- *S. cerevisiae*
- *S. pombe*
- *P. falciparum*
- Select / Download
- Promoter analysis tools
- FTP site

Access EPD
- Promoter elements
- Select / Download
- FTP site

Access MGA Database
- MGA-Search
- MGA Data Overview
- MGA FTP site

Documents

Other Resources

References

What is new

Contact us

in H. sapiens SEARCH

Please help us make EPD better! Take our short survey to suggest new tools or organism covered. It will take only a few minutes.

General Information

[Help]

Promoter ID:	**RBL2_1**
Promoter type:	region
Organism:	Homo sapiens (Human)
Gene Symbol:	RBL2
Description of the gene:	RB transcriptional corepressor like 2
Sequence:	gtttgcggcggcgcaggcgcggtgcgggcggcggacgggcgggcgcttcGCCGTTTGAAT
Position in the genome:	Chromosome [NC_000016.10]; Strand [+]; Position [53434491]
Ensembl:	ENSG00000103479
RefSeq:	NM_001323610
NCBI Gene:	RBL2
GeneCards:	RBL2

Promoter Image [View it at UCSC]

[Help]

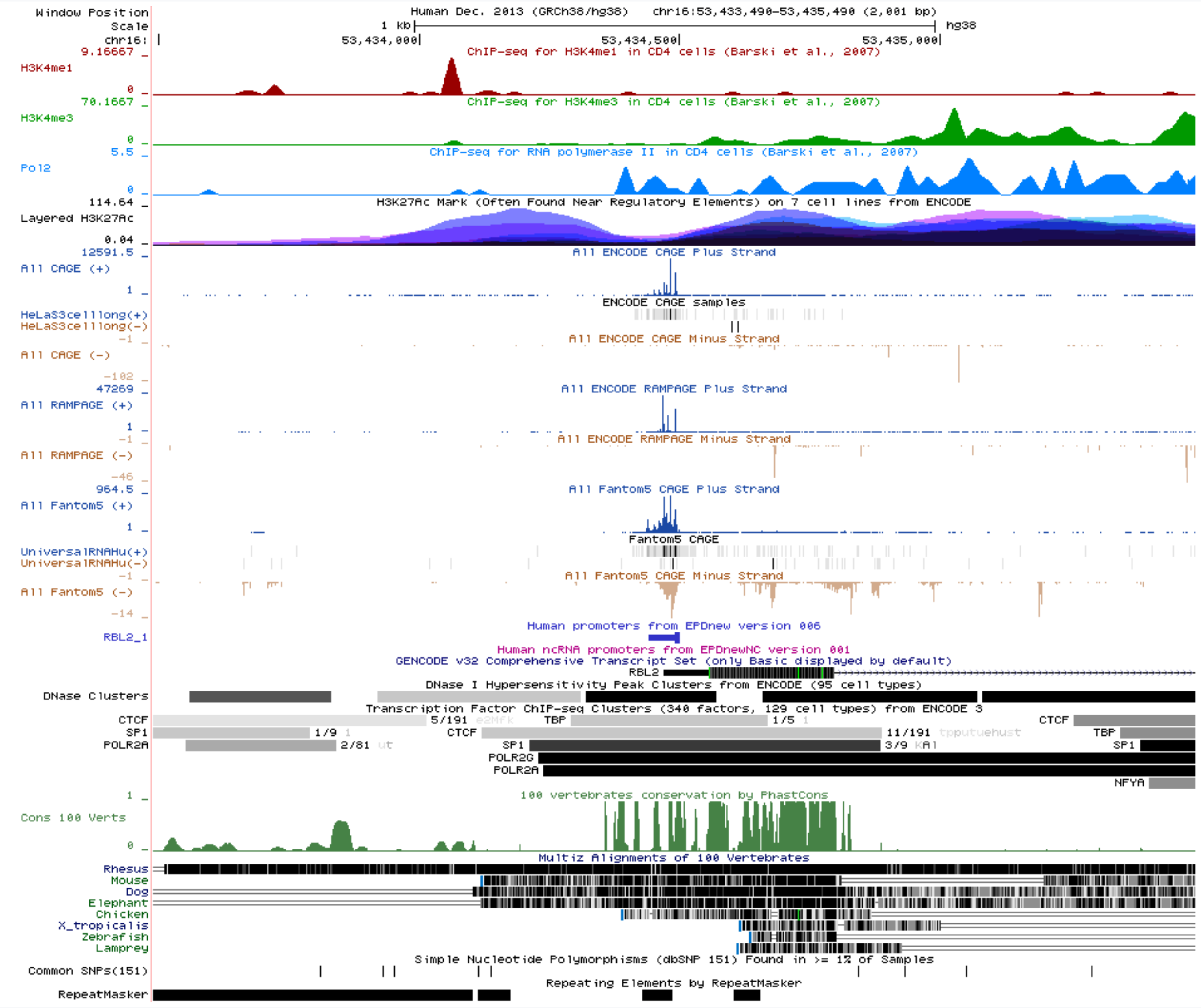

Other viewers

[Help]

EPD view at UCSC (hg38):	chr16:53432490–53436490 (European mirror)
EPD view at UCSC (hg19):	chr16:53466402–53470402 (European mirror)
SwissRegulon (hg19):	chr16:53466403–53470403
FANTOM5 (hg38):	chr16:53432491–53436491
DBTSS/KERO (hg38):	chr16:53432491–53436491
refTSS (hg38):	chr16:53432490–53436490 (European mirror)

Selective viewer showing the 3 Fantom5 tissues with the highest expression of **RBL2_1** (European mirror)

GM12878 viewer (European mirror)

Sequence Retrieval Tool

[Help]

Get sequence RBL2_1 **from** -1000 **to** 100 bp relative to TSS GET SEQUENCE

☐ lower case upstream TSS

```
>FP021856 RBL2_1 :+U EU:NC; range -1000 to 100.
ACAGGAGTTTGGTTATTCAAATCAGCCTACCAGAAAATTCGGAGACTGGGGTTTTTAAAG
AATGACTTGGCGGGTAGGGGGCCAGGGATTGGCGAATGCTAATTTGTCAGGTGGGAGGTG
AAATCACAGGGGGTTGAAGTGGGCTCTTGCTGTCTTCTGTTACTGAGTGGAATTGCAGAA
CTTGTTGAGCCAGATTATGGTCTGAGTGGCGCCAGCTAGTGCATCGGAATGCGCGGTCTG
AAAAGTATCTCCAGCACCAATCTTAGGTTTTACAATAGTGATGTTATCCCTGAGAGCAAT
TGGGGAGGTCAGGAATCTTATAGCCTCTGGCTGCAAGCCTCCTAAATCATAATTTCTAAT
CTTGTGGCTAATTTGTTAGTTCTACAAAGGCAGACTGATCCCCAGGCAAGAATGGGGTTT
GTTTTTGGAAAGGACTGTTACAATCTTTGTTTCAAAGTGAAATTAGAAATTAAATTCCTC
CTGTAGTTAGTTAGGTCTTCGCCCAGGAATGAACAAGGGCAGCTCGGAAGTGAGAAGCGT
GGAGTCATTTAGGTCAGATCCCTTGCACTGTCATAACTTTCTCACTGTTAGGATTTTTGC
AAAGGCAGTTTCGTGAACGTACAGAGACAGGCCCTTGCTATTATCCCTATTTTTTAGATA
AGGATATCCAGGCGATGAGGAAGTTTTACTTCTGGGAACAGCCTGGATACGAAACCTTCA
CACGTCAGTGTCTTTTGGACATTTTCTCGTCAGTACAGCCCTGTTGAATGTTCTCACGGT
GGGGAGGTACGTGTTTAAAATGCGGGGAAGGTGCTTTTATTTCACCCCTGGTGAAACTAG
GGGAGCTAATTTTTTTAAACATGATTTTTGGCCCCCTTGAACCGCCGGCCTGGACTACGT
TTCCCAGCAGCCCGTGCTCAAGACTACGGGTGCCTGCAGGCGGTCAGCGTCGTTTGCGGC
GGCGCAGGCGCGGTGCGGGCGGCGGACGGGCGGGCGCTTCGCCGTTTGAATGGCTGCGGG
CCCGGGCCCTCACCTCACCTGAGGTCCGGCCGCCCAGGGGTGCGCTATGCCGTCGGGAGG
TGACCAGTCGCCACCGCCCCC
```

Search Motif Tool

[Help]

Library: Transcription Factor Motifs (JASPAR Motif: RARA::RXRA

From: -1000 To: 100 bp relative to TSS and a cut-off (p-value) of: 0.001 SEARCH

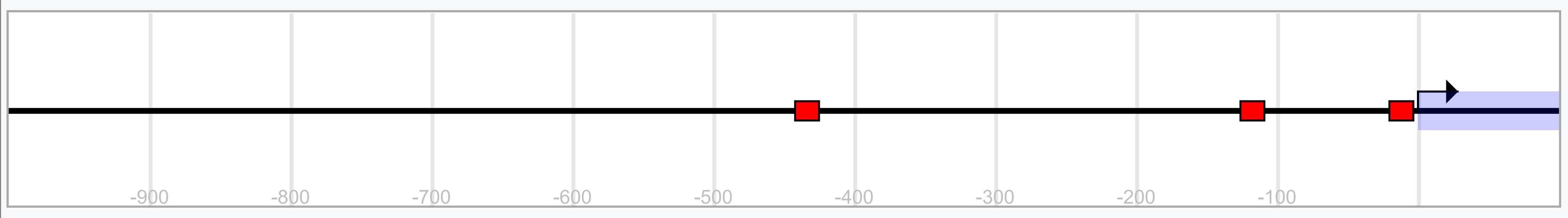

RARA::RXRA [p-value = 0.001]: -442, -126, -21

Expression Profile Tool

[Help]

RBL2_1 is expressed in **1036 samples** with an **average expression of 115.421 tags** per 10M.

The TSSs distribution around the EPDnew annotated start site (click on the histogram bars to explore samples):

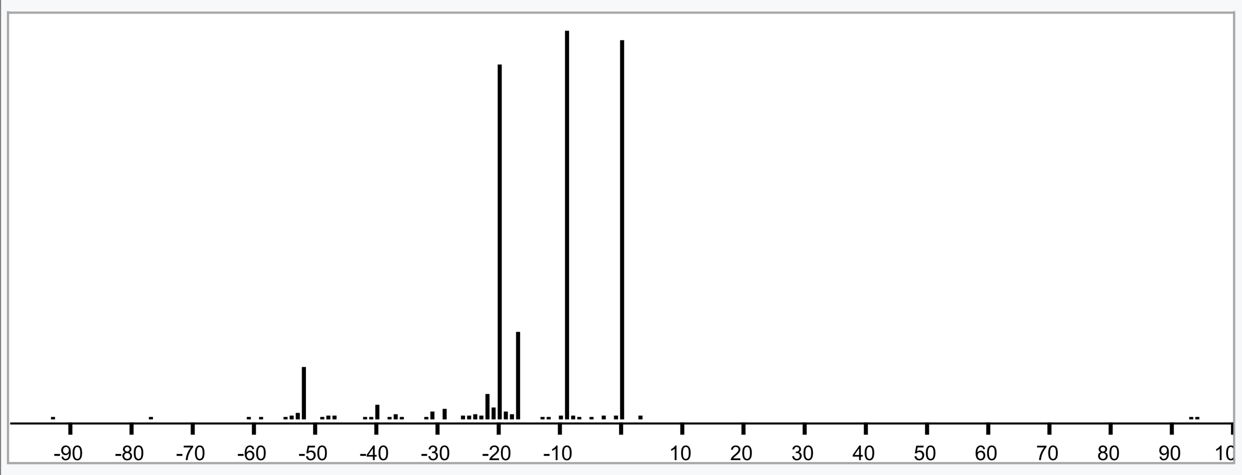

Last update October 2019

RB2 P130

JASPAR RARA MOTIF

Access EPDnew
H. sapiens
H. sapiens non-coding
M. mulatta
M. musculus
M. musculus non-coding
R. norvegicus
C. familiaris
G. gallus
D. melanogaster
A. mellifera
D. rerio
C. elegans
A. thaliana
Z. mays
S. cerevisiae
S. pombe
P. falciparum
Select / Download
Promoter analysis tools
FTP site
Access EPD
Promoter elements
Select / Download
FTP site
Access MGA Database
MGA-Search
MGA Data Overview
MGA FTP site
Documents
Other Resources
References
What is new
Contact us

in H. sapiens SEARCH

Please help us make EPD better! Take our short survey to suggest new tools or organism covered. It will take only a few minutes.

General Information

[Help]

Promoter ID:	**RBL2_1**
Promoter type:	region
Organism:	Homo sapiens (Human)
Gene Symbol:	RBL2
Description of the gene:	RB transcriptional corepressor like 2
Sequence:	gtttgcggcggcgcaggcgcggtgcggcggcggacgggcgggcgcttcGCCGTTTGAAT
Position in the genome:	Chromosome [NC_000016.10]; Strand [+]; Position [53434491]
Ensembl:	ENSG00000103479
RefSeq:	NM_001323610
NCBI Gene:	RBL2
GeneCards:	RBL2

Promoter Image [View it at UCSC]

[Help]

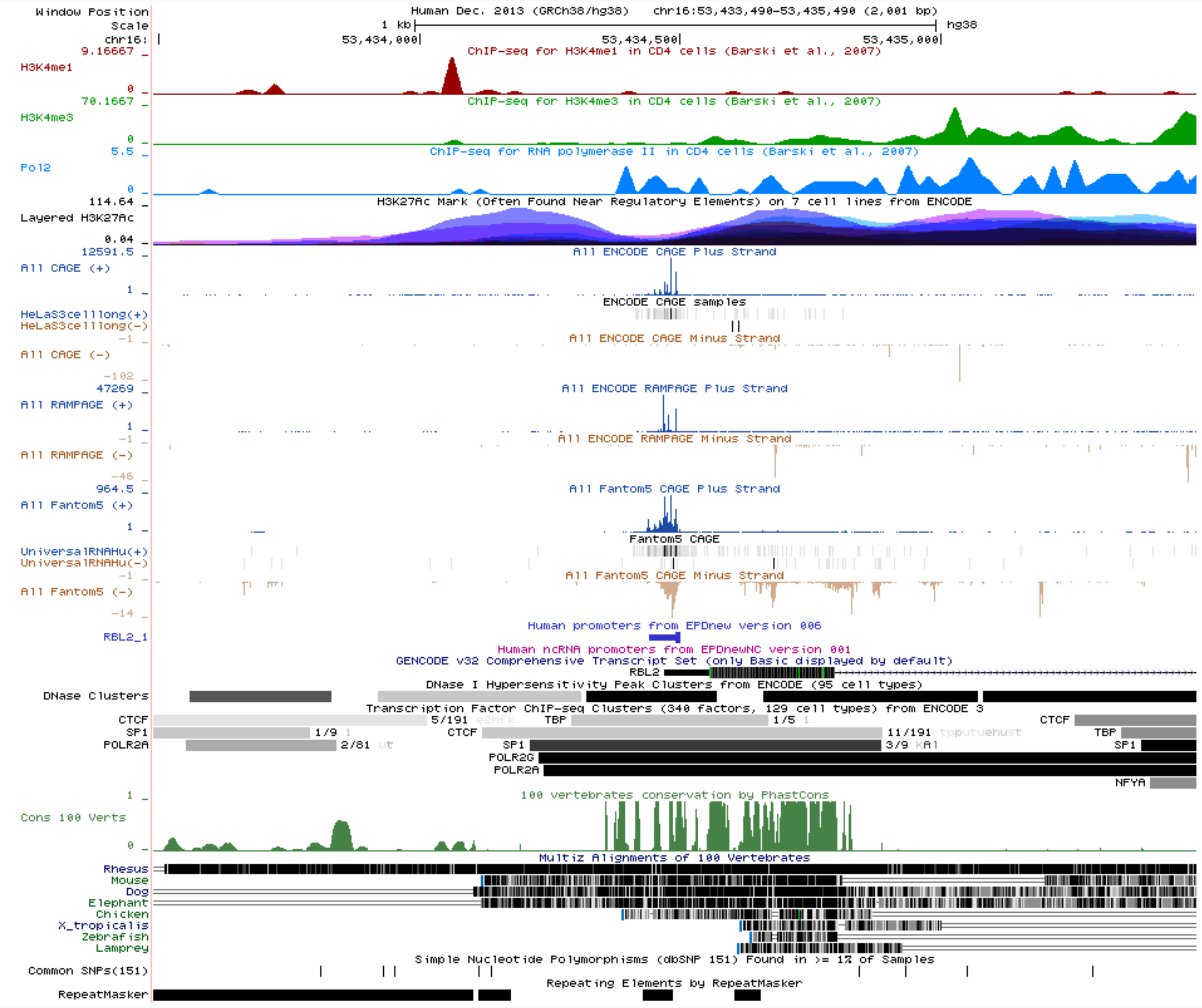

Other viewers

[Help]

EPD view at UCSC (hg38): chr16:53432490–53436490 (European mirror)
EPD view at UCSC (hg19): chr16:53466402–53470402 (European mirror)
SwissRegulon (hg19): chr16:53466403–53470403
FANTOM5 (hg38): chr16:53432491–53436491
DBTSS/KERO (hg38): chr16:53432491–53436491
refTSS (hg38): chr16:53432490–53436490 (European mirror)

Selective viewer showing the 3 Fantom5 tissues with the highest expression of **RBL2_1** (European mirror)
GM12878 viewer (European mirror)

Sequence Retrieval Tool

[Help]

Get sequence RBL2_1 **from** -1000 **to** 100 bp relative to TSS GET SEQUENCE
lower case upstream TSS

```
>FP021856 RBL2_1 :+U EU:NC; range -1000 to 100.
ACAGGAGTTTGGTTATTCAAATCAGCCTACCAGAAAATTCGGAGACTGGGGTTTTTAAAG
AATGACTTGGCGGGTAGGGGGCCAGGGATTGGCGAATGCTAATTTGTCAGGTGGGAGGTG
AAATCACAGGGGGTTGAAGTGGGCTCTTGCTGTCTTCTGTTACTGAGTGGAATTGCAGAA
CTTGTTGAGCCAGATTATGGTCTGAGTGGCGCCAGCTAGTGCATCGGAATGCGCGGTCTG
AAAAGTATCTCCAGCACCAATCTTAGGTTTTACAATAGTGATGTTATCCCTGAGAGCAAT
TGGGGAGGTCAGGAATCTTATAGCCTCTGGCTGCAAGCCTCCTAAATCATAATTTCTAAT
CTTGTGGCTAATTTGTTAGTTCTACAAAGGCAGACTGATCCCCAGGCAAGAATGGGGTTT
GTTTTTGGAAAGGACTGTTACAATCTTTGTTTCAAAGTGAAATTAGAAATTAAATTCCTC
CTGTAGTTAGTTAGGTCTTCGCCCAGGAATGAACAAGGGCAGCTCGGAAGTGAGAAGCGT
GGAGTCATTTAGGTCAGATCCCTTGCACTGTCATAACTTTCTCACTGTTAGGATTTTTGC
AAAGGCAGTTTCGTGAACGTACAGAGACAGGCCCTTGCTATTATCCCTATTTTTTAGATA
AGGATATCCAGGCGATGAGGAAGTTTTACTTCTGGGAACAGCCTGGATACGAAACCTTCA
CACGTCAGTGTCTTTTGGACATTTTCTCGTCAGTACAGCCCTGTTGAATGTTCTCACGGT
GGGGAGGTACGTGTTTAAAATGCGGGGAAGGTGCTTTTATTTCACCCCTGGTGAAACTAG
GGGAGCTAATTTTTTTAAACATGATTTTTGGCCCCCTTGAACCGCCGGCCTGGACTACGT
TTCCCAGCAGCCCGTGCTCAAGACTACGGGTGCCTGCAGGCGGTCAGCGTCGTTTGCGGC
GGCGCAGGCGCGGTGCGGGCGGCGGACGGGCGGGCGCTTCGCCGTTTGAATGGCTGCGGG
CCCGGGCCCTCACCTCACCTGAGGTCCGGCCGCCCAGGGGTGCGCTATGCCGTCGGGAGG
TGACCAGTCGCCACCGCCCCC
```

Search Motif Tool

[Help]

Library: Transcription Factor Motifs (JASPAR Motif: RARA
From: -1000 To: 100 bp relative to TSS and a cut-off (p-value) of: 0.001 SEARCH

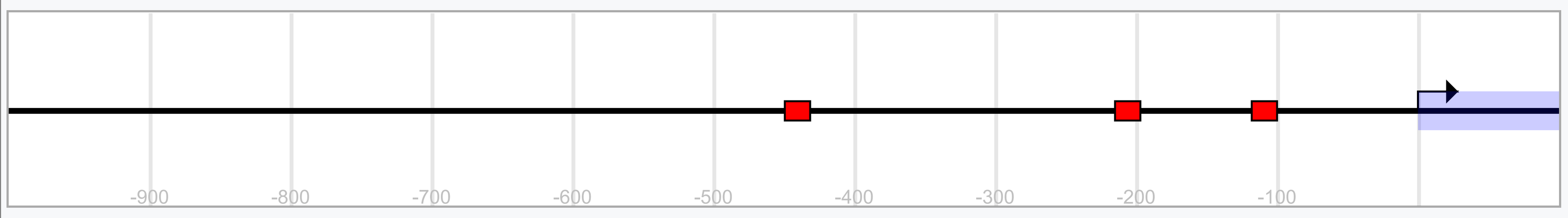

RARA [p-value = 0.001]: -450, -215, -118

Expression Profile Tool

[Help]

RBL2_1 is expressed in **1036 samples** with an **average expression of 115.421 tags** per 10M.

The TSSs distribution around the EPDnew annotated start site (click on the histogram bars to explore samples):

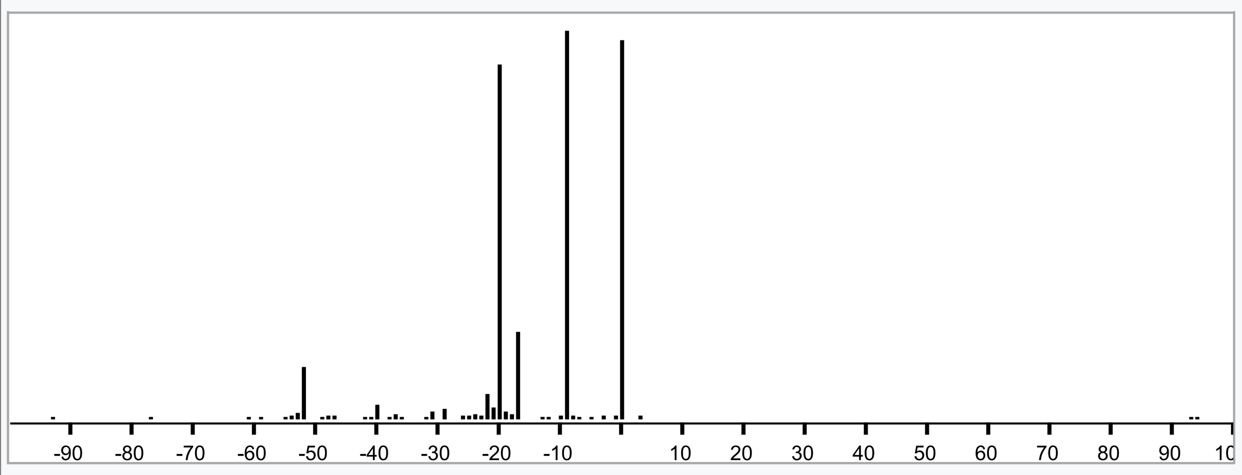

RB2 P130

PATCH RARE MOTIFS

Thank you for using Patch at Host - It's Fri, 16.4.2021 - 12:49 MEZ

Start a new search

Name of your search:	default
Name of your sequence:	default.seq
Sites searched for:	all
Maximum number of mismatches:	2
Mismatch penalty:	100
Lower score boundary:	87.5

Scanning sequence: FP021856 RBL2_1 :+U EU:NC; range -1000 to 100.
View a graphic of the following search results

Identifier	Position	Mismatches	Score	Binding Factor	Sequence (Search Pattern)
RAT$CYTOP_03	5 (+)	1	87.50		GAGTTGGGT
AS$AREB6_42	7 (+)	0	100.00	AREB6	GTTTG
AS$AREB6_43	7 (+)	0	100.00	AREB6	GTTTG
AS$AREB6_45	7 (+)	0	100.00	AREB6	GTTTG
AS$AREB6_47	7 (+)	0	100.00	AREB6	GTTTG
AS$AREB6_51	7 (+)	0	100.00	AREB6	GTTTG
CHICK$H2B_01	14 (+)	1	87.50	POU2F1	TATGCAAAT
HS$IGH_01	14 (+)	1	87.50	POU2F1	TATGCAAAT
AD4$ORI_01	14 (+)	1	87.50	POU2F1, POU2F2, POU2F2 (Oct-2.1), POU2F2 (Oct-2.3), POU2F2 (Oct-2.4), POU2F2 (Oct-2.6), POU3F1	TATGCAAAT
XENLA$U2SN_10	14 (+)	1	87.50	POU2F1	TATGCAAAT
Y$GAL3_01	14 (+)	0	100.00	MIG1	TATTC
PM$H2BA_02	14 (-)	1	87.50	POU2F1	ATTTGCATA
MOUSE$IGKL_12	14 (-)	1	87.50	POU2F1	ATTTGCATA
MOUSE$IGKL_13	14 (-)	1	87.50	NF-A3	ATTTGCATA
MOUSE$IGKL_17	15 (-)	1	88.89	POU2F1	TGATTTGCAT
MOUSE$IGKL_18	15 (-)	1	88.89	POU2F2, POU2F2 (Oct-2.1), POU2F2B	TGATTTGCAT
AS$MATA1_10	15 (-)	1	88.89	MATa1	TGATGTGAAT
MOUSE$MBP_04	16 (+)	0	100.00	TFIID	TTCAAA
HS$CYP3A4_05	16 (+)	1	87.50		TTGAAATCA
AS$PAX2_69	16 (+)	1	87.50	Pax-2.1, Pax-2.2	GTCAAATCA
HS$CDC2_10	16 (-)	0	100.00		TTGAA
HS$CYCA_06	16 (-)	0	100.00		TTGAA
HS$CDC25C_05	16 (-)	0	100.00		TTGAA
DROME$UBX_24	17 (-)	0	100.00	LEF-1	TTTGA
HS$EGFR_14	18 (+)	0	100.00		CAAAT
MAIZE$PMS1_01	18 (+)	0	100.00		CAAAT
HS$IGH_04	18 (-)	0	100.00		ATTTG
MOUSE$PLF_01	18 (-)	0	100.00	c-Fos, c-Jun, GR	TGATTTG
MOUSE$BMG_01	20 (-)	0	100.00	GATA-1	TGATT
MOUSE$MYOD_04	23 (-)	0	100.00	ARP-1, RXR-alpha, RXR-gamma, T3R-alpha	AGGCTG
Y$GAL1_10	24 (+)	0	100.00	GAL4	AGCCT
SV$SV40_63	25 (+)	0	100.00	T-Ag	GCCTA
PARS$PAL_05	26 (+)	0	100.00		CCTACCA
MOUSE$IGLL_03	26 (+)	1	87.50	LyF-1	CCTCCCAGA
PV$CHS_05	26 (+)	0	100.00		CCTACC
AS$LYF1_06	26 (-)	1	87.50	LyF-1	TCTGGGAGG
TO$E4_02	27 (+)	0	100.00		CTACC
AS$STAT5A_56	28 (+)	1	87.50	STAT5A	TACCAGGAA
AS$STAT5A_53	28 (-)	1	87.50	STAT5A	TTCCTGGTA
DROME$TWI_05	31 (+)	1	88.89	Dl	CAGAAAAATC
HS$TNFA_04	32 (+)	0	100.00		AGAAA
I$HSF_01	32 (+)	0	90.00	HSF	AGAAN
F$HSF_01	32 (+)	0	90.00	HSF	AGAAN
Y$GAL4_01	33 (-)	0	100.00	MIG1	TTTTC
Y$MAL61_03	34 (-)	0	100.00	MIG1	ATTTT
Y$MEL1_01	38 (+)	0	100.00	GAL4	TTCGG
Y$MEL1_01	38 (-)	0	100.00	GAL4	CCGAA
Y$ACS1_02	39 (-)	0	100.00	ADR1	CTCCGA
RAT$ANP_01	41 (+)	0	100.00		GGAGAC
Y$ADH2_01	41 (-)	0	100.00	ADR1	TCTCC
YL$XPR2_01	42 (-)	0	100.00		GTCTC
CHICK$STN1_02	45 (-)	0	100.00	LBP-1, NF-1 (-like proteins)	CCAGT
HS$PAI_07	45 (-)	0	100.00		CCAGT
SV$SV40_37	46 (+)	0	100.00		CTGGG
MOUSE$IGH_61	46 (+)	1	87.50	core-binding factor	CTGTGGTTT
AS$SPZ1_20	46 (+)	1	87.50	Spz1	CGGGGGTTT
Y$GAL3_01	46 (-)	0	100.00	MIG1	CCCCAG
Y$GAL4_01	46 (-)	0	100.00	MIG1	CCCCAG
Y$HAP4_01	46 (-)	0	100.00	MIG1	CCCCAG
RAT$ME_01	47 (+)	0	100.00	T3R-alpha, T3R-beta1, T3R-beta2	TGGGGT
Y$SUC2_01	52 (+)	0	100.00	MIG1	TTTTT
AS$PF1_01	52 (+)	0	100.00	PF1	TTTTT
AS$PF1_02	52 (+)	0	100.00	PF1	TTTTT
Y$MAL2R_01	53 (+)	0	100.00	MIG1	TTTTA
AS$FOXJ1_04	53 (+)	0	100.00	FOXJ1	TTTTA
MOUSE$WAP_06	54 (+)	0	100.00		TTTAAA
Y$MAL63_01	54 (+)	0	100.00	MIG1	TTTAA
AS$PAX2_59	54 (+)	1	87.50	Pax-2.1, Pax-2.2	TTGAAAGAA
MOUSE$WAP_06	54 (-)	0	100.00		TTTAAA
Y$MAL63_01	55 (-)	0	100.00	MIG1	TTTAA
ASPN$YA_02	59 (+)	0	100.00	abaA	AGAATG
I$HSF_01	59 (+)	0	90.00	HSF	AGAAN
F$HSF_01	59 (+)	0	90.00	HSF	AGAAN
ASPN$ABAA_03	59 (-)	0	100.00	abaA	CATTCT
ASPN$ABAA_04	59 (-)	0	100.00	abaA	CATTCT
ASPN$ABAA_05	59 (-)	0	100.00	abaA	CATTCT
ASPN$BRLA_03	59 (-)	0	100.00	abaA	CATTCT
ASPN$RODA_05	59 (-)	0	100.00	abaA	CATTCT
ABAA$CONS_01	59 (-)	0	91.67	abaA	CATTCY
ASPN$BRLA_05	59 (-)	0	100.00	abaA	CATTCT
AS$DSF_06	63 (+)	0	100.00	DSF	TGACTT
AS$DSF_07	63 (+)	0	100.00	DSF	TGACTT
AS$DSF_08	63 (+)	0	100.00	DSF	TGACTT
AS$DSF_09	63 (+)	0	100.00	DSF	TGACTT
Y$HIS3_03	63 (-)	0	100.00	GCN4	AAGTCA
Y$ILV1_03	63 (-)	0	100.00	GCN4	AAGTCA
RAT$CRBP1_01	63 (-)	0	100.00	COUP-TF1, RAR-beta, RXR-alpha	AAGTCA
AS$RAR_11	63 (-)	0	100.00	RAR-alpha1, RXR-alpha	AAGTCA
PV$DLEC2_01	63 (-)	1	87.50	ROM1, ROM2	GCCACGTCA
NT$CHN50_01	63 (-)	0	100.00	WRKY1, WRKY3, WRKY4	AGTCA
AS$DSF_01	63 (-)	0	100.00	DSF	AAGTCA
AS$DSF_02	63 (-)	0	100.00	DSF	AAGTCA
AS$DSF_03	63 (-)	0	100.00	DSF	AAGTCA
AS$DSF_04	63 (-)	0	100.00	DSF	AAGTCA
AS$DSF_06	63 (-)	0	100.00	DSF	AAGTCA
AS$DSF_07	63 (-)	0	100.00	DSF	AAGTCA
AS$DSF_08	63 (-)	0	100.00	DSF	AAGTCA
AS$DSF_09	63 (-)	0	100.00	DSF	AAGTCA
CHICK$OA_01	66 (+)	0	100.00	NF-1 (-like proteins)	CTTGGC
MOUSE$POLA2_02	66 (+)	1	87.50	E2F-1	TTTGGCGGG
MOUSE$BMYB_01	66 (+)	0	100.00	E2F, E2F-1, E2F-3, E2F-4	CTTGGCGGG
HS$NPY_03	66 (-)	0	100.00	AP-1, CCAAT-binding factor	CCAAG
HS$TK_03	66 (-)	1	87.50	E2F	CCCGCCAGG
MOUSE$AP2_07	67 (+)	0	100.00	NF-1	TTGGC
Y$CYB2_01	67 (-)	1	87.50	repressor of CAR1 expression	AACCGCCAA
Y$HSF1_01	67 (-)	1	87.50	repressor of CAR1 expression	AGCCGCCAA
HSV1$IE3_06	69 (+)	0	100.00	Sp1	GGCGGG
RAT$NF1_02	69 (+)	0	100.00	Sp1	GGCGGG
HS$NPY_02	69 (+)	0	100.00	Sp1	GGCGGG
HS$CDC25C_04	69 (+)	0	100.00		GGCGG
HS$MIP_02	69 (+)	0	100.00	Sp1	GGCGGG
AS$SPZ1_12	69 (+)	1	87.50	Spz1	GGAGGGTAG
HS$P21WAF1_06	69 (-)	0	100.00	SMAD-3, SMAD-4	CCCGCC
HS$P21WAF1_07	69 (-)	0	100.00		CCCGCC
Y$RAP1_01	71 (-)	1	88.89	REB1	CCGCTACCCG
HS$MIP_03	72 (+)	1	87.50	Sp1	GGGGAGGGG
RAT$EAI_07	72 (-)	0	100.00		CTACCC
HS$ZG_06	72 (-)	1	87.50	Sp1	CCCCTCCCC
HS$AAC_15	72 (-)	1	90.91	Sp1	TGCCCCCTACCC
HS$AAC_13	72 (-)	1	90.00	Sp1	GCCCCCTCCCC
TO$E4_02	73 (-)	0	100.00		CTACC
PV$CHS_05	73 (-)	0	100.00		CCTACC
AS$ADR1_15	74 (+)	0	100.00	ADR1	GTAGGGG
AS$VDR_03	76 (+)	0	100.00	VDR	AGGGGG
AS$VDR_04	76 (+)	0	100.00	VDR	AGGGG
AS$VDR_05	76 (+)	0	100.00	VDR	AGGGGG
AS$VDR_07	76 (+)	0	100.00	VDR	AGGGGG
AS$VDR_09	76 (+)	0	100.00	VDR	AGGGGG
Y$GLK1_02	76 (+)	0	100.00	MSN2, MSN4	AGGGG
MOUSE$A11COL_01	77 (+)	1	88.89	CBF (2), IF2	GGGGGCCGGG
HS$CYCD1_14	77 (-)	0	100.00	Sp1, Sp2, Sp3, Sp4	CCCCC
HS$CYCD1_15	77 (-)	0	100.00	Sp1, Sp2, Sp3, Sp4	CCCCC
PA$PY_23	78 (+)	0	100.00	T-Ag	GGGGC
SV$SV40_04	78 (+)	0	100.00	T-Ag	GGGGC
HS$GG_36	78 (+)	0	100.00	Sp1	GGGGCC
SV$SV40_63	78 (+)	0	100.00	T-Ag	GGGGC
AS$CF1_18	78 (+)	1	87.50	CF1	GGGGTCAGG
PA$PY_26	78 (-)	0	100.00	T-Ag	GCCCC
MAIZE$ADH11S_06	78 (-)	0	100.00	GCBP-1, Sp1	GCCCC
HS$GRH_01	80 (+)	0	100.00	AP-2, AP-2alphaA, AP-2alphaB, NF-1	GGCCA
RAT$TAT_15	80 (+)	0	100.00	HNF-3alpha	GGCCA
HS$GRH_01	80 (-)	0	100.00	AP-2, AP-2alphaA, AP-2alphaB, NF-1	TGGCC
MOUSE$DHFR_06	85 (+)	1	87.50		GGTATTGGC
DROME$EVE_23	85 (+)	1	87.50	Bcd	GGGATTAGC
AS$ZIC3_24	85 (+)	1	87.50	Zic3	GGGGTTGGC
MOUSE$DHFR_05	86 (+)	0	100.00		GGATTGGC
HIV1$HIV1_26	86 (+)	1	87.50	HMBP	GGAGTGGCG
HS$HH1_02	87 (+)	0	100.00	H1TF1, HiNF-B	GATTGG
HS$HSP70_06	87 (+)	0	100.00	CCAAT-binding factor	GATTGG
HS$HSP70_09	87 (+)	0	100.00	CP1, CTF	GATTGG
MOUSE$A11COL_01	88 (+)	0	100.00	CBF (2), IF2	ATTGG
MOUSE$A21COL_03	88 (+)	0	100.00	CCAAT-binding factor	ATTGG
MOUSE$A21COL_04	88 (+)	0	100.00	CBF (2), CBF-A, CBF-B	ATTGG
MOUSE$A21COL_05	88 (+)	0	100.00	EFI	ATTGG
AD2$E2L_03	88 (+)	0	100.00	CRF	ATTGG
HS$GHA_06	88 (+)	0	100.00	NF-1	ATTGG
SP$H2B1_02	88 (+)	0	100.00	CBF (1)	ATTGG
SP$H2B1_03	88 (+)	0	100.00	CDF	ATTGG
HS$HSP70_02	88 (+)	0	100.00	CTF	ATTGG
HS$HSP70_07	88 (+)	0	100.00	CBTF, CP1, CTF	ATTGG
MOUSE$M2EAK_07	88 (+)	0	100.00	NF-Y	ATTGG
MOUSE$M2EAK_08	88 (+)	0	100.00	NF-Y	ATTGG
RSV$RSV_05	88 (+)	0	100.00		ATTGG
HSV1$TK_04	88 (+)	0	100.00	C/EBPalpha	ATTGG
HSV1$TK_05	88 (+)	0	100.00	NF-Y'	ATTGG
AS$HAP23_01	88 (+)	0	100.00	HAP2, HAP3, HAP4	ATTGG
MOUSE$M1H2KB_12	88 (+)	0	100.00	EFI	ATTGG
RAT$TH2A_02	88 (+)	0	100.00		ATTGG
RAT$TH2A_04	88 (+)	0	100.00		ATTGG
HS$TK_02	88 (+)	0	100.00	CP1	ATTGG
MOUSE$A11COL_07	88 (+)	0	100.00	NF-1, Sp1	ATTGG
HS$CDC2_07	88 (+)	0	100.00	CBAF, CBF-B, CP1A	ATTGG
XENLA$GATA2_01	88 (+)	0	100.00	CBTF	ATTGG
RAT$NAT_05	88 (+)	0	100.00		ATTGG
HS$CDC2_12	88 (+)	0	100.00	CBAF	ATTGG
HS$BAC_03	88 (-)	0	100.00	SRF	CCAAT
AMULV$AMULV_01	88 (-)	0	100.00		CCAAT
EBV$DSL_06	88 (-)	0	100.00		CCAAT
MOUSE$AAG_04	88 (-)	0	100.00	alpha-CP1, alpha-CP2a, alpha-CP2b, alpha-IRP	CCAAT
MOUSE$AAG_05	88 (-)	0	100.00	alpha-CP1, CCAAT-binding factor	GCCAAT
CHICK$BAG_06	88 (-)	0	100.00	TGGCA-binding protein	CCAAT
HS$GG_15	88 (-)	0	100.00	NF-E	CCAAT
HS$GG_16	88 (-)	0	100.00	CDP2, Clox, CUTL1, Cutl1	CCAAT
HS$GG_17	88 (-)	0	100.00	gammaCAAT	CCAAT
HS$GG_18	88 (-)	0	100.00	CP1	CCAAT
HS$ZG_07	88 (-)	0	100.00	CP2	CCAAT
HS$ZG_08	88 (-)	0	100.00	CP1	CCAAT
HS$HH1_03	88 (-)	0	100.00	H1TF2	CCAAT
MOUSE$M2IAB_01	88 (-)	0	100.00	PU.1	CCAAT
AD2$ORI_01	88 (-)	0	100.00	CTF-1, DBP, NF-1	GCCAAT
PIG$UPA_12	88 (-)	0	100.00	NF-1	CCAAT
RADLV$RLV_10	88 (-)	0	100.00		CCAAT
MULV$MULV_03	88 (-)	0	100.00		CCAAT
MOUSE$GLUT4_04	88 (-)	0	100.00		CCAAT
MOMLV$MOMULV_15	88 (-)	0	100.00		CCAAT
RAT$ALDB_02	88 (-)	0	100.00	CBF (2), CP2	CCAAT
HS$GG_20	88 (-)	0	100.00	CP1, NF-E3	CCAAT
RAT$TH2B_01	88 (-)	0	100.00		CCAAT
RAT$TH2B_02	88 (-)	0	100.00		CCAAT
HS$GG_21	88 (-)	0	100.00	CP1	CCAAT
MOUSE$LY6E_01	88 (-)	0	100.00		CCAAT
RAT$NEU_01	88 (-)	0	100.00		CCAAT
HS$GHA_10	88 (-)	0	100.00	alpha-CBF	CCAAT
NEUCR$CPC1_02	88 (-)	0	100.00		CCAAT
HS$EG_07	88 (-)	0	100.00	CP1	CCAAT
HS$GP2B_13	88 (-)	0	100.00		CCAAT
MOUSE$JUND_04	88 (-)	0	100.00		GCCAAT
MOUSE$BMG_10	88 (-)	0	100.00		CCAAT
HS$CYBH_01	88 (-)	0	100.00	CDP2, Clox, CP1, CUTL1, Cutl1	CCAAT
HBV$S_04	88 (-)	0	100.00		CCAAT
HS$FN_06	88 (-)	0	100.00		CCAAT
MOUSE$E2F1_02	88 (-)	0	100.00		CCAAT
HS$A24COL_01	88 (-)	0	100.00		CCAAT
MOUSE$EKLF_01	88 (-)	0	100.00	AP-1, GATA-1	CCAAT
MOUSE$AP2_07	89 (+)	0	100.00	NF-1	TTGGC
MOUSE$BMG_08	89 (-)	0	100.00	NF-1	TCGCCAA
FACT1$CONS	90 (+)	0	100.00	F-ACT1	TGGCGA
MOUSE$IGH_35	95 (-)	1	87.50	POU2F1	ATTTGCATT
MOUSE$IGH_36	95 (-)	1	87.50	POU2F1a, POU2F1b, POU2F1c, POU2F2, POU2F2 (Oct-2.1), POU2F2 (Oct-2.3), POU2F2 (Oct-2.4), POU2F2 (Oct-2.6), POU2F2B, POU3F1, POU5F1 (Oct-5)	ATTTGCATT
OCT4$CONS	96 (+)	0	93.75	POU5F1, POU5F1A, POU5F1B	ATGCWAAT
MOUSE$E1_03	96 (-)	0	100.00	POU2F1a, POU2F1b, POU2F1c, POU5F1	ATTAGCAT
MOUSE$E1_04	96 (-)	0	100.00	POU2F1a, POU2F1b, POU2F1c, POU5F1	ATTAGCAT
AS$HOXA3_01	98 (+)	1	87.50	HOXA3, MATalpha1	GGTAATTTG
DROME$EN_06	98 (-)	1	87.50	En	CCAATTAGC
HS$GG_22	99 (+)	0	100.00		CTAAT
RAT$OPSIN_01	99 (+)	0	100.00		CTAAT
XENLA$BMP4_01	99 (+)	0	100.00	Xvent-2	CTAATT
AS$XVENT2_01	99 (+)	0	100.00	Xvent-2	CTAATT
Y$SUC2_02	100 (-)	0	100.00	MIG1	AATTA
RAT$DBH_01	100 (-)	0	100.00	ARIX, c-Fos, c-Jun, CREB, CREMtau	AATTA
DROME$YP1_03	101 (-)	1	87.50	DSXF, DSXM	TTACAAATT
HS$IGH_04	102 (+)	0	100.00		ATTTG
HS$EGFR_14	102 (-)	0	100.00		CAAAT
MAIZE$PMS1_01	102 (-)	0	100.00		CAAAT
RAT$SPI_01	102 (-)	1	87.50	C/EBPalpha	CTGAGAAAT
AS$MEIS1_04	104 (-)	0	100.00	Meis-1a, Meis-1b	TGACAA
AD5$E1A_27	105 (+)	1	87.50		TCTCAGGTG
AS$TGIF_01	105 (+)	0	100.00	TGIF	TGTCA
AS$TGIF_02	105 (+)	0	100.00	TGIF	TGTCA
AS$TGIF_03	105 (+)	0	100.00	TGIF	TGTCA
AS$TGIF_04	105 (+)	0	100.00	TGIF	TGTCA
AS$TGIF_05	105 (+)	0	100.00	TGIF	TGTCA
AS$TGIF_06	105 (+)	0	100.00	TGIF	TGTCA
AS$TGIF_07	105 (+)	0	100.00	TGIF	TGTCA
AS$TGIF_08	105 (+)	0	100.00	TGIF	TGTCA
AS$TGIF_10	105 (+)	0	100.00	TGIF	TGTCA
AS$TGIF_11	105 (+)	0	100.00	TGIF	TGTCA
AS$TGIF_12	105 (+)	0	100.00	TGIF	TGTCA
AS$TGIF_13	105 (+)	0	100.00	TGIF	TGTCA
AS$TGIF_14	105 (+)	0	100.00	TGIF	TGTCA
AS$TGIF_15	105 (+)	0	100.00	TGIF	TGTCA
AS$MEIS1_03	105 (+)	0	100.00	Meis-1a, Meis-1b	TGTCAG
AS$MEIS1_06	105 (+)	0	100.00	Meis-1a, Meis-1b	TGTCAG
POT$PR10a_01	105 (+)	0	100.00	PBF-1, PBF-2 (p24)	TGTCA
AS$ZIC2_12	105 (+)	1	87.50	Zic2	TGTCTGGTG
AS$MEIS1_10	105 (-)	0	100.00	Meis-1a, Meis-1b	CTGACA
AS$MEIS1_13	105 (-)	0	100.00	Meis-1a, Meis-1b	CTGACA
POT$PR10a_01	105 (-)	0	100.00	PBF-1, PBF-2 (p24)	TGACA
RAT$EAI_09	106 (+)	0	100.00		GTCAG
DELTAEF1$CONS_01	107 (-)	0	92.86	deltaEF1	CACCTNA
AS$AREB6_05	107 (-)	1	87.50	AREB6	CACACCTGA
AS$AREB6_14	107 (-)	0	100.00	AREB6	CCACCTGA
MOUSE$CD4_03	108 (+)	0	100.00	E12, HEB	CAGGTG
DROME$HB_12	108 (+)	0	100.00	Ac, AS-C T3, Da, Sc	CAGGTG
DROME$AC_01	108 (+)	0	100.00	Ac, AS-C T3, Da, Sc	CAGGTG
AS$SNAIL_01	108 (+)	0	100.00	Sn	CAGGTG
AS$LMO2_01	108 (+)	0	100.00	CLIM2, E12, E47, GATA-1, Lmo2, Tal-1	CAGGTG
HS$VEGF_02	108 (+)	0	100.00		CAGGT
MOUSE$ACRD_02	108 (-)	0	100.00	E12, E47, MyoD, myogenin	CACCTG
XENLA$AC_05	108 (-)	0	100.00	EMF1, MyoD	CACCTG
MOUSE$TDT_07	108 (-)	0	100.00		CCACCTG
MOUSE$IGLL_10	108 (-)	0	100.00		CCACCTG
HS$CXCR4_02	108 (-)	0	100.00	c-Myc, USF2	CACCTG
PV$PHASL_02	108 (-)	0	100.00	CAN	CACCTG
BPV1$BPV1_24	110 (+)	0	100.00	Sp1	GGTGG
HS$BG_48	110 (+)	0	100.00	CAC-binding protein	GGTGG
MOUSE$CEBPA_06	110 (+)	0	100.00	Sp1	GGTGG
HS$GPB_02	110 (+)	0	100.00	Sp1	GGTGGG
HT1$HTLV1_08	110 (-)	0	100.00	c-Ets-1 68	CCACC
HS$BG_52	110 (-)	0	100.00		CCACC
HS$LCK_02	110 (-)	1	87.50	LyF-1	CCTCCCAAC
HS$BG_14	110 (-)	0	100.00		CCACC
HS$BG_44	110 (-)	0	100.00	CAC-binding protein	CCACC
HS$APOB_10	112 (+)	0	100.00	AP-2alphaA, AP-2alphaB	TGGGAG
HS$CDH1_01	112 (+)	0	100.00	LUN-1	TGGGA
HS$TLN_01	112 (+)	0	100.00	LUN-1	TGGGA
HS$CDH1_01	112 (-)	0	100.00	LUN-1	TCCCA
HS$TLN_01	112 (-)	0	100.00	LUN-1	TCCCA
MOUSE$SCC_06	113 (+)	1	87.50	SF-1	GGGAGGTCA
HT1$HTLV1_08	114 (-)	0	100.00	c-Ets-1 68	CCTCC
HS$MHC2IN_01	114 (-)	1	88.89		TTTCACTTCC
AS$AREB6_01	114 (-)	1	87.50	AREB6	TTCACCTGC
AS$INO2_01	115 (+)	1	88.89	INO2	GATGTGAAAT
Y$INO1_01	116 (+)	1	87.50	NBF	ATGTGAAAT
HS$RARG_01	116 (+)	0	100.00	RAR-alpha1, RAR-beta, RAR-gamma, RXR-alpha	AGGTGA
HSV1$TK_12	116 (+)	0	100.00	RAR-alpha1, T3R-alpha	AGGTGA
MOUSE$IBABP_01	116 (+)	0	100.00	FXR, RXR-alpha	AGGTGA
AS$ERR1_16	116 (+)	0	100.00	ERR1	AGGTGA
RAT$ME_08	116 (+)	0	100.00	PPAR-gamma, RXR-gamma	AGGTGA
HS$CAMHC_01	116 (-)	0	100.00	Hp55, Hp65, p58, RAR-alpha1, RXR-beta, T3R-alpha, T3R-beta1, T3R-beta2	TCACCT
HS$CYP24_02	116 (-)	0	100.00	VDR	TCACCT
RAT$CYP24_01	116 (-)	0	100.00	VDR	TCACCT
HS$APOA2_06	117 (-)	0	100.00		TCACC
MOUSE$TCRBL_21	118 (+)	1	88.89	TF68	GTGACATCAC
AS$NFKAPPAB_17	118 (+)	1	88.89	NF-kappaB	GGGAAATCAC
HS$CYP3A4_05	118 (+)	1	87.50		TTGAAATCA
AS$PAX2_69	118 (+)	1	87.50	Pax-2.1, Pax-2.2	GTCAAATCA
AS$NFKAPPAB_12	118 (-)	1	88.89	NF-kappaB	GGGATTTCAC
HS$HH4_01	120 (-)	0	100.00	H4TF-1	GATTTC
MOUSE$IGH_61	121 (-)	1	87.50	core-binding factor	CTGTGGTTT
MOUSE$BMG_01	122 (-)	0	100.00	GATA-1	TGATT
RAT$POMC_04	123 (-)	0	100.00	GR	TGTGAT
AS$STRE_05	126 (+)	0	100.00		ACAGGGGG
AS$VDR_03	128 (+)	0	100.00	VDR	AGGGGG
AS$VDR_04	128 (+)	0	100.00	VDR	AGGGG
AS$VDR_05	128 (+)	0	100.00	VDR	AGGGGG
AS$VDR_07	128 (+)	0	100.00	VDR	AGGGGG
AS$VDR_09	128 (+)	0	100.00	VDR	AGGGGG
Y$GLK1_02	128 (+)	0	100.00	MSN2, MSN4	AGGGG
HS$CYCD1_14	129 (-)	0	100.00	Sp1, Sp2, Sp3, Sp4	CCCCC
HS$CYCD1_15	129 (-)	0	100.00	Sp1, Sp2, Sp3, Sp4	CCCCC
AS$CF1_37	130 (+)	1	87.50	CF1	GGGGTTGTA
HS$CDC2_10	134 (+)	0	100.00		TTGAA
HS$CYCA_06	134 (+)	0	100.00		TTGAA
HS$CDC25C_05	134 (+)	0	100.00		TTGAA
SV$SV40_63	140 (+)	0	100.00	T-Ag	TGGGC
AMV$AMV_02	146 (+)	0	100.00	C/EBPalpha	CTTGC
RAV0$RAV0_01	146 (-)	0	100.00	C/EBPalpha	GCAAG
MOUSE$THY1_06	147 (-)	0	100.00		CAGCAA
MOUSE$M2EAK_03	148 (-)	0	100.00	NF-Y	CAGCA
HS$GG_12	150 (+)	0	100.00	NF-E	CTGTC
DROME$EVE_10	150 (+)	0	100.00	GAGA factor	CTGTC
AS$MEIS1_10	150 (+)	0	100.00	Meis-1a, Meis-1b	CTGTCT
AS$TGIF_09	151 (+)	0	100.00	TGIF	TGTCT
MOUSE$RAS1_02	153 (+)	0	100.00	GR	TCTTCT
I$HSF_01	154 (-)	0	90.00	HSF	AGAAN
F$HSF_01	154 (-)	0	90.00	HSF	AGAAN
AS$MEIS1AHOXA9_07	157 (-)	0	100.00	HOXA9, Meis-1a	TAACAG
RAT$MT1_06	163 (+)	1	87.50		CTGAGTGCA
Y$ILV1_02	164 (+)	0	100.00	GCN4	TGAGTG
DROME$WHLO_02	164 (+)	0	100.00	Zeste	TGAGTG
DROME$WHLO_05	164 (+)	0	100.00	Zeste	TGAGTG
DROME$DPP_03	164 (+)	0	100.00	Zeste	TGAGTG
DROME$UBX_03	164 (-)	0	100.00	Zeste	CACTCA
DROME$UBX_04	164 (-)	0	100.00	Zeste	CACTCA
DROME$WHLO_03	164 (-)	0	100.00	Zeste	CACTCA
DROME$WHLO_06	164 (-)	0	100.00	Zeste	CACTCA
DROME$WHLO_07	164 (-)	0	100.00	Zeste	CACTCA
DROME$WHLO_08	164 (-)	0	100.00	Zeste	CACTCA
DROME$WHLO_09	164 (-)	0	100.00	Zeste	CACTCA
DROME$DPP_02	164 (-)	0	100.00	Zeste	CACTCA
DROME$DPP_04	164 (-)	0	100.00	Zeste	CACTCA
AS$WZF1_01	165 (-)	0	100.00	WZF1	CACTC
WHEAT$H3_02	165 (-)	0	100.00	WZF1	CACTC
WHEAT$H3_03	165 (-)	0	100.00	WZF1	CACTC
JCV$JCV_03	166 (+)	0	100.00	NF-1	AGTGGAA
Y$MAL61_04	171 (+)	0	100.00	MIG1	AATTG
CHICK$ACRA_06	172 (-)	0	100.00		GCAAT
RSV$RSV_04	172 (-)	0	100.00	C/EBP, C/EBPalpha, C/EBPbeta, C/EBPbeta(p20), C/EBPbeta(p34), C/EBPgamma, EFII	GCAAT
HS$RANTES_02	172 (-)	1	87.50	C/EBPbeta	TTGTGCAAT
RAT$PGS2_01	172 (-)	1	87.50	C/EBPbeta	TTATGCAAT
I$HSF_01	177 (+)	0	90.00	HSF	AGAAN
F$HSF_01	177 (+)	0	90.00	HSF	AGAAN
HS$PR264_04	178 (+)	0	100.00	c-Myb	GAACTT
TDNA$NOS_01	186 (+)	0	100.00	ASF-1, OBF3.1, TGA1a, TGA1b	TGAGC
EBV$BZLF1_04	186 (+)	0	100.00	Zta	TGAGCCA
RAT$ARGL_03	188 (+)	0	100.00	NF-1	AGCCAG
AS$PAX2_49	191 (-)	1	87.50	Pax-2.1, Pax-2.2	CATAATCCG
SP$SPEC2A_02	192 (+)	0	100.00	SpOtx	AGATTA
MOUSE$CTNC_01	192 (+)	0	100.00	GATA-4	AGATTA
BOVIN$LHB_02	192 (+)	0	100.00	Pitx1	AGATTA
MOUSE$BMG_04	192 (-)	0	100.00	GATA-1	AATCT
SP$SPEC2A_02	192 (-)	0	100.00	SpOtx	TAATCT
AS$MTTFA_10	195 (+)	0	100.00	mtTFA	TTATG
AS$MTTFA_11	195 (+)	0	100.00	mtTFA	TTATG
AS$FTZ_54	198 (-)	0	100.00	Ftz	AGACCA
Y$ILV1_02	203 (+)	0	100.00	GCN4	TGAGTG
HIV1$HIV1_26	203 (+)	1	87.50	HMBP	GGAGTGGCG
DROME$WHLO_02	203 (+)	0	100.00	Zeste	TGAGTG
DROME$WHLO_05	203 (+)	0	100.00	Zeste	TGAGTG
DROME$DPP_03	203 (+)	0	100.00	Zeste	TGAGTG
DROME$UBX_03	203 (-)	0	100.00	Zeste	CACTCA
DROME$UBX_04	203 (-)	0	100.00	Zeste	CACTCA
DROME$WHLO_03	203 (-)	0	100.00	Zeste	CACTCA
DROME$WHLO_06	203 (-)	0	100.00	Zeste	CACTCA
DROME$WHLO_07	203 (-)	0	100.00	Zeste	CACTCA
DROME$WHLO_08	203 (-)	0	100.00	Zeste	CACTCA
DROME$WHLO_09	203 (-)	0	100.00	Zeste	CACTCA
DROME$DPP_02	203 (-)	0	100.00	Zeste	CACTCA
DROME$DPP_04	203 (-)	0	100.00	Zeste	CACTCA
AS$WZF1_01	204 (-)	0	100.00	WZF1	CACTC
WHEAT$H3_02	204 (-)	0	100.00	WZF1	CACTC
WHEAT$H3_03	204 (-)	0	100.00	WZF1	CACTC
HS$CRP_03	205 (+)	1	88.89	C/EBPalpha, C/EBPbeta, C/EBPdelta	AGTGGCGCAA
MOUSE$IGH_16	211 (-)	1	87.50	E47, HEB, INSAF, MyoD, myogenin, Tal-1	CCAGCTGGC
RAT$MLC_02	211 (-)	1	87.50	HEB, MyoD, myogenin	CCAGCTGGC
CHICK$STN1_01	212 (+)	0	100.00	LBP-1, MyoD	CCAGC
CHICK$STN1_03	212 (+)	0	100.00	LBP-1, NF-1 (-like proteins)	CCAGC
DROME$HSP27_05	218 (-)	0	100.00	FXR, RXR-alpha	TGCACT
RAT$OC_05	218 (-)	0	100.00	RXR-alpha, VDR	TGCACT
MOUSE$MT1_01	219 (-)	0	100.00	Sp1	TGCAC
AS$STAT5A_43	221 (-)	1	87.50	STAT5A	TTCCGAGGC
HS$BAC_04	224 (-)	0	100.00	Elk-1, SAP-1a, SAP-1b, SRF	TTCCGA
E74A$CONS_01	225 (+)	0	90.00	E74A	MGGAA
ASPN$RODA_01	226 (+)	0	100.00	abaA	GGAATG
ASPN$RODA_02	226 (+)	0	100.00	abaA	GGAATG
ASPN$RODA_03	226 (+)	0	100.00	abaA	GGAATG
ASPN$YA_01	226 (-)	0	100.00	abaA	CATTCC
ASPN$ABAA_01	226 (-)	0	100.00	abaA	CATTCC
ASPN$ABAA_02	226 (-)	0	100.00	abaA	CATTCC
ASPN$BRLA_01	226 (-)	0	100.00	abaA	CATTCC
ASPN$BRLA_02	226 (-)	0	100.00	abaA	CATTCC
ASPN$BRLA_04	226 (-)	0	100.00	abaA	CATTCC
ASPN$WETA_01	226 (-)	0	100.00	abaA	CATTCC
ASPN$WETA_02	226 (-)	0	100.00	abaA	CATTCC
ASPN$WETA_03	226 (-)	0	100.00	abaA	CATTCC
ASPN$WETA_04	226 (-)	0	100.00	abaA	CATTCC
ASPN$RODA_04	226 (-)	0	100.00	abaA	CATTCC
ABAA$CONS_01	226 (-)	0	91.67	abaA	CATTCY
ASPN$ABAA_06	226 (-)	0	100.00	abaA	CATTCC
AS$ZIC_11	230 (+)	1	87.50	Zic1	TACGCGGTC
HS$CDC2_09	232 (+)	0	100.00		CGCGG
HS$CYCA_05	232 (+)	0	100.00		CGCGG
HS$CYCD1_14	232 (-)	0	100.00	Sp1, Sp2, Sp3, Sp4	CCGCG
Y$GAL4_01	240 (-)	0	100.00	MIG1	TTTTC
Y$PHR1_01	242 (+)	0	100.00	PRP, RPH1	AAAGTA
Y$PHR1_01	242 (-)	0	100.00	PRP, RPH1	TACTTT
RAT$GHF1_01	244 (+)	0	100.00	PTF	AGTAT
RAT$GHF1_01	244 (-)	0	100.00	PTF	ATACT
HS$GPB_05	245 (+)	0	100.00	GATA-1	GTATCT
HS$GG_03	246 (+)	0	100.00	GATA-1, POU2F1	TATCTC
ASPN$NIAD_04	246 (+)	0	100.00	NIT2	TATCTC
NIT2$CONS_01	246 (+)	0	91.67	NIT2	TATCTM
MOUSE$GSHPX1_06	246 (+)	0	100.00	GATA-1	TATCT
MOUSE$GSHPX1_07	246 (+)	0	100.00	GATA-1	TATCT
MOUSE$GSHPX1_09	246 (+)	0	100.00	GATA-1	TATCT
F$NIT2_01	246 (+)	0	91.67	NIT2	TATCTM
MOUSE$BMG_02	246 (-)	0	100.00	GATA-1	AGATA
Y$ADH2_01	248 (+)	0	100.00	ADR1	TCTCC
AS$SPZ1_24	250 (-)	1	87.50	Spz1	GGTGCTAGA
CHICK$STN1_01	251 (+)	0	100.00	LBP-1, MyoD	CCAGC
CHICK$STN1_03	251 (+)	0	100.00	LBP-1, NF-1 (-like proteins)	CCAGC
MOUSE$M2EAK_03	252 (+)	0	100.00	NF-Y	CAGCA
MESAT$PRP2_03	252 (-)	1	87.50	Alfin1	GTGGTGCTG
RAT$A1I3_04	253 (+)	0	100.00		AGCAC
RAT$A1I3_04	253 (-)	0	100.00		GTGCT
HS$BAC_03	257 (+)	0	100.00	SRF	CCAAT
AMULV$AMULV_01	257 (+)	0	100.00		CCAAT
EBV$DSL_06	257 (+)	0	100.00		CCAAT
MOUSE$AAG_04	257 (+)	0	100.00	alpha-CP1, alpha-CP2a, alpha-CP2b, alpha-IRP	CCAAT
CHICK$BAG_06	257 (+)	0	100.00	TGGCA-binding protein	CCAAT
HS$GG_15	257 (+)	0	100.00	NF-E	CCAAT
HS$GG_16	257 (+)	0	100.00	CDP2, Clox, CUTL1, Cutl1	CCAAT
HS$GG_17	257 (+)	0	100.00	gammaCAAT	CCAAT
HS$GG_18	257 (+)	0	100.00	CP1	CCAAT
HS$ZG_07	257 (+)	0	100.00	CP2	CCAAT
HS$ZG_08	257 (+)	0	100.00	CP1	CCAAT
HS$HH1_03	257 (+)	0	100.00	H1TF2	CCAAT
MOUSE$M2IAB_01	257 (+)	0	100.00	PU.1	CCAAT
PIG$UPA_12	257 (+)	0	100.00	NF-1	CCAAT
RADLV$RLV_10	257 (+)	0	100.00		CCAAT
MULV$MULV_03	257 (+)	0	100.00		CCAAT
MOUSE$GLUT4_04	257 (+)	0	100.00		CCAAT
MOMLV$MOMULV_15	257 (+)	0	100.00		CCAAT
RAT$ALDB_02	257 (+)	0	100.00	CBF (2), CP2	CCAAT
HS$GG_20	257 (+)	0	100.00	CP1, NF-E3	CCAAT
RAT$TH2B_01	257 (+)	0	100.00		CCAAT
RAT$TH2B_02	257 (+)	0	100.00		CCAAT
HS$GG_21	257 (+)	0	100.00	CP1	CCAAT
MOUSE$LY6E_01	257 (+)	0	100.00		CCAAT
RAT$NEU_01	257 (+)	0	100.00		CCAAT
HS$GHA_10	257 (+)	0	100.00	alpha-CBF	CCAAT
NEUCR$CPC1_02	257 (+)	0	100.00		CCAAT
HS$EG_07	257 (+)	0	100.00	CP1	CCAAT
HS$GP2B_13	257 (+)	0	100.00		CCAAT
MOUSE$BMG_10	257 (+)	0	100.00		CCAAT
HS$CYBH_01	257 (+)	0	100.00	CDP2, Clox, CP1, CUTL1, Cutl1	CCAAT
HBV$S_04	257 (+)	0	100.00		CCAAT
HS$FN_06	257 (+)	0	100.00		CCAAT
MOUSE$E2F1_02	257 (+)	0	100.00		CCAAT
HS$A24COL_01	257 (+)	0	100.00		CCAAT
MOUSE$EKLF_01	257 (+)	0	100.00	AP-1, GATA-1	CCAAT
MOUSE$A11COL_01	257 (-)	0	100.00	CBF (2), IF2	ATTGG
MOUSE$A21COL_03	257 (-)	0	100.00	CCAAT-binding factor	ATTGG
MOUSE$A21COL_04	257 (-)	0	100.00	CBF (2), CBF-A, CBF-B	ATTGG
MOUSE$A21COL_05	257 (-)	0	100.00	EFI	ATTGG
AD2$E2L_03	257 (-)	0	100.00	CRF	ATTGG
HS$GHA_06	257 (-)	0	100.00	NF-1	ATTGG
HS$HH1_02	257 (-)	0	100.00	H1TF1, HiNF-B	GATTGG
SP$H2B1_02	257 (-)	0	100.00	CBF (1)	ATTGG
SP$H2B1_03	257 (-)	0	100.00	CDF	ATTGG
HS$HSP70_02	257 (-)	0	100.00	CTF	ATTGG
HS$HSP70_06	257 (-)	0	100.00	CCAAT-binding factor	GATTGG
HS$HSP70_07	257 (-)	0	100.00	CBTF, CP1, CTF	ATTGG
HS$HSP70_09	257 (-)	0	100.00	CP1, CTF	GATTGG
MOUSE$M2EAK_07	257 (-)	0	100.00	NF-Y	ATTGG
MOUSE$M2EAK_08	257 (-)	0	100.00	NF-Y	ATTGG
RSV$RSV_05	257 (-)	0	100.00		ATTGG
HSV1$TK_04	257 (-)	0	100.00	C/EBPalpha	ATTGG
HSV1$TK_05	257 (-)	0	100.00	NF-Y'	ATTGG
AS$HAP23_01	257 (-)	0	100.00	HAP2, HAP3, HAP4	ATTGG
MOUSE$M1H2KB_12	257 (-)	0	100.00	EFI	ATTGG
RAT$TH2A_02	257 (-)	0	100.00		ATTGG
RAT$TH2A_04	257 (-)	0	100.00		ATTGG
HS$TK_02	257 (-)	0	100.00	CP1	ATTGG
MOUSE$A11COL_07	257 (-)	0	100.00	NF-1, Sp1	ATTGG
HS$CDC2_07	257 (-)	0	100.00	CBAF, CBF-B, CP1A	ATTGG
XENLA$GATA2_01	257 (-)	0	100.00	CBTF	ATTGG
RAT$NAT_05	257 (-)	0	100.00		ATTGG
HS$CDC2_12	257 (-)	0	100.00	CBAF	ATTGG
HS$GG_26	258 (-)	0	100.00	GATA-1	AGATTG
MOUSE$BMG_04	259 (+)	0	100.00	GATA-1	AATCT
AS$HOXA3_05	259 (-)	1	87.50	HOXA3	GCTAAGATT
XENLA$ACY_01	260 (+)	0	100.00	SRF	ATCTT
XENLA$ACY_01	260 (-)	0	100.00	SRF	AAGAT
DROME$KNI_06	263 (-)	1	87.50	Tll	AAAAGCTAA
HS$IL2_07	267 (-)	0	100.00	NF-IL-2A, POU2F1, POU2F2 (Oct-2.1)	GTAAAAC
Y$MAL2R_01	268 (+)	0	100.00	MIG1	TTTTA
AS$FOXJ1_04	268 (+)	0	100.00	FOXJ1	TTTTA
CHICK$CTNT_05	269 (+)	1	88.89	aMEF-2, MEF-2 (516 AA)	TTTAAAATAG
HS$EGFR_14	270 (+)	1	87.50		TTACAATAT
AS$FOXJ2_42	273 (-)	1	88.89	FOXJ2 (long isoform)	ATCAATATTG
AS$MSX1_04	276 (+)	1	87.50	Msx-1	TAGTGATTT
AS$MATA1_02	279 (+)	1	88.89	MATa1	TGATGTAATC
MOUSE$INOS_06	279 (+)	1	87.50	C/EBPbeta	TGATGTAAT
AS$HSO_01	280 (+)	0	100.00	MATa1, MATalpha2	GATGT
HS$BPDE_01	283 (-)	0	100.00	Crx	GGATAAC
Y$ARS1_05	284 (+)	0	100.00	ABF2	TTATC
AS$MTTFA_01	284 (+)	0	100.00	mtTFA	TTATC
AS$MTTFA_02	284 (+)	0	100.00	mtTFA	TTATC
AS$MTTFA_03	284 (+)	0	100.00	mtTFA	TTATC
AS$MTTFA_04	284 (+)	0	100.00	mtTFA	TTATC
AS$MTTFA_05	284 (+)	0	100.00	mtTFA	TTATC
AS$MTTFA_06	284 (+)	0	100.00	mtTFA	TTATC
AS$MTTFA_07	284 (+)	0	100.00	mtTFA	TTATC
AS$MTTFA_08	284 (+)	0	100.00	mtTFA	TTATC
AS$MTTFA_09	284 (+)	0	100.00	mtTFA	TTATC
EBV$BHLF1_05	284 (-)	0	100.00	TBP	GATAA
Y$DAL3_01	284 (-)	0	100.00	DAL80	GATAA
MOUSE$IGLL_02	285 (-)	1	87.50	LyF-1	TCAGGGAGA
RAT$MT1_06	290 (+)	1	87.50		CTGAGTGCA
DROME$EVE_08	292 (+)	0	100.00	GAGA factor	GAGAG
DROME$EVE_13	292 (+)	0	100.00	GAGA factor	GAGAG
DROME$FTZ_32	292 (+)	0	100.00	GAGA factor	GAGAG
AS$GAGA_03	292 (+)	0	100.00	GAGA factor	GAGAG
DROME$EVE_09	292 (-)	0	100.00	GAGA factor	CTCTC
DROME$FTZ_32	292 (-)	0	100.00	GAGA factor	CTCTC
MOUSE$CRISP1_04	293 (+)	0	100.00	AR	AGAGCA
MOUSE$CRISP3_02	293 (+)	0	100.00	AR	AGAGCA
RAT$VEGF_02	294 (+)	0	100.00	ER-alpha, ER-beta	GAGCA
CHICK$ACRA_06	296 (+)	0	100.00		GCAAT
RSV$RSV_04	296 (+)	0	100.00	C/EBP, C/EBPalpha, C/EBPbeta, C/EBPbeta(p20), C/EBPbeta(p34), C/EBPgamma, EFII	GCAAT
XENLA$AC_05	297 (+)	0	100.00	EMF1, MyoD	CAATTG
XENLA$AC_05	297 (-)	0	100.00	EMF1, MyoD	CAATTG
Y$MAL61_04	297 (-)	0	100.00	MIG1	AATTG
Y$MAL61_04	298 (+)	0	100.00	MIG1	AATTG
AS$ADR1_13	298 (+)	0	100.00	ADR1	AATTGGGG
MOUSE$A11COL_01	299 (+)	0	100.00	CBF (2), IF2	ATTGG
MOUSE$A21COL_03	299 (+)	0	100.00	CCAAT-binding factor	ATTGG
MOUSE$A21COL_04	299 (+)	0	100.00	CBF (2), CBF-A, CBF-B	ATTGG
MOUSE$A21COL_05	299 (+)	0	100.00	EFI	ATTGG
AD2$E2L_03	299 (+)	0	100.00	CRF	ATTGG
HS$GHA_06	299 (+)	0	100.00	NF-1	ATTGG
SP$H2B1_02	299 (+)	0	100.00	CBF (1)	ATTGG
SP$H2B1_03	299 (+)	0	100.00	CDF	ATTGG
HS$HSP70_02	299 (+)	0	100.00	CTF	ATTGG
HS$HSP70_07	299 (+)	0	100.00	CBTF, CP1, CTF	ATTGG
MOUSE$M2EAK_07	299 (+)	0	100.00	NF-Y	ATTGG
MOUSE$M2EAK_08	299 (+)	0	100.00	NF-Y	ATTGG
RSV$RSV_05	299 (+)	0	100.00		ATTGG
HSV1$TK_04	299 (+)	0	100.00	C/EBPalpha	ATTGG
HSV1$TK_05	299 (+)	0	100.00	NF-Y'	ATTGG
AS$HAP23_01	299 (+)	0	100.00	HAP2, HAP3, HAP4	ATTGG
MOUSE$M1H2KB_12	299 (+)	0	100.00	EFI	ATTGG
RAT$TH2A_02	299 (+)	0	100.00		ATTGG
RAT$TH2A_04	299 (+)	0	100.00		ATTGG
HS$TK_02	299 (+)	0	100.00	CP1	ATTGG
MOUSE$A11COL_07	299 (+)	0	100.00	NF-1, Sp1	ATTGG
HS$CDC2_07	299 (+)	0	100.00	CBAF, CBF-B, CP1A	ATTGG
XENLA$GATA2_01	299 (+)	0	100.00	CBTF	ATTGG
RAT$NAT_05	299 (+)	0	100.00		ATTGG
HS$CDC2_12	299 (+)	0	100.00	CBAF	ATTGG
HS$BAC_03	299 (-)	0	100.00	SRF	CCAAT
AMULV$AMULV_01	299 (-)	0	100.00		CCAAT
EBV$DSL_06	299 (-)	0	100.00		CCAAT
MOUSE$AAG_04	299 (-)	0	100.00	alpha-CP1, alpha-CP2a, alpha-CP2b, alpha-IRP	CCAAT
CHICK$BAG_06	299 (-)	0	100.00	TGGCA-binding protein	CCAAT
HS$GG_15	299 (-)	0	100.00	NF-E	CCAAT
HS$GG_16	299 (-)	0	100.00	CDP2, Clox, CUTL1, Cutl1	CCAAT
HS$GG_17	299 (-)	0	100.00	gammaCAAT	CCAAT
HS$GG_18	299 (-)	0	100.00	CP1	CCAAT
HS$ZG_07	299 (-)	0	100.00	CP2	CCAAT
HS$ZG_08	299 (-)	0	100.00	CP1	CCAAT
HS$HH1_03	299 (-)	0	100.00	H1TF2	CCAAT
MOUSE$M2IAB_01	299 (-)	0	100.00	PU.1	CCAAT
PIG$UPA_12	299 (-)	0	100.00	NF-1	CCAAT
RADLV$RLV_10	299 (-)	0	100.00		CCAAT
MULV$MULV_03	299 (-)	0	100.00		CCAAT
MOUSE$GLUT4_04	299 (-)	0	100.00		CCAAT
MOMLV$MOMULV_15	299 (-)	0	100.00		CCAAT
RAT$ALDB_02	299 (-)	0	100.00	CBF (2), CP2	CCAAT
HS$GG_20	299 (-)	0	100.00	CP1, NF-E3	CCAAT
RAT$TH2B_01	299 (-)	0	100.00		CCAAT
RAT$TH2B_02	299 (-)	0	100.00		CCAAT
HS$GG_21	299 (-)	0	100.00	CP1	CCAAT
MOUSE$LY6E_01	299 (-)	0	100.00		CCAAT
RAT$NEU_01	299 (-)	0	100.00		CCAAT
HS$GHA_10	299 (-)	0	100.00	alpha-CBF	CCAAT
NEUCR$CPC1_02	299 (-)	0	100.00		CCAAT
HS$EG_07	299 (-)	0	100.00	CP1	CCAAT
HS$GP2B_13	299 (-)	0	100.00		CCAAT
MOUSE$BMG_10	299 (-)	0	100.00		CCAAT
HS$CYBH_01	299 (-)	0	100.00	CDP2, Clox, CP1, CUTL1, Cutl1	CCAAT
HBV$S_04	299 (-)	0	100.00		CCAAT
HS$FN_06	299 (-)	0	100.00		CCAAT
MOUSE$E2F1_02	299 (-)	0	100.00		CCAAT
HS$A24COL_01	299 (-)	0	100.00		CCAAT
MOUSE$EKLF_01	299 (-)	0	100.00	AP-1, GATA-1	CCAAT
BPV1$BPV1_13	300 (+)	0	100.00	AP-2alphaA, AP-2alphaB	TTGGGG
Y$ACS1_02	300 (+)	0	100.00	ADR1	TTGGGG
BPV1$BPV1_13	300 (-)	0	100.00	AP-2alphaA, AP-2alphaB	CCCCAA
HS$CD8A_03	300 (-)	1	87.50	LyF-1	CCTCCCAAA
MOUSE$M1H2KB_02	301 (+)	0	100.00	AP-2alpha, AP-2alphaA, AP-2alphaB	TGGGGA
MOUSE$M1H2KB_07	301 (+)	0	100.00	MBP-1 (1), NF-kappaB1, NF-kappaB2, NF-kappaB2 precursor	TGGGGA
MOUSE$M1H2KB_07	301 (-)	0	100.00	MBP-1 (1), NF-kappaB1, NF-kappaB2, NF-kappaB2 precursor	TCCCCA
MOUSE$SCC_06	303 (+)	0	100.00	SF-1	GGGAGGTCA
HT1$HTLV1_08	304 (-)	0	100.00	c-Ets-1 68	CCTCC
MOUSE$M1H2LD_01	305 (+)	1	88.89	RAR-beta, RXR-beta	GAGGTCAGGG
HS$CD2_06	305 (+)	0	100.00		GAGGTCA
AS$CF1_18	305 (+)	1	87.50	CF1	GGGGTCAGG
CHICK$OA_02	306 (+)	0	100.00	ARP-1, COUP, COUP-TF1, PPAR-alpha, RXR-alpha	AGGTCA
XENLA$VITA2_02	306 (+)	0	100.00	ER-alpha, ER-beta1, NHP-1, T3R-alpha1, T3R-beta1	AGGTCA
PPAR$CONS_01	306 (+)	0	100.00	PPAR-alpha	AGGTCA
AS$GR_12	306 (+)	0	100.00	GR-alpha, GR-beta	AGGTCA
RAT$CRBP2_01	306 (+)	0	100.00	COUP-TF1, PPAR-alpha, RAR-alpha1, RAR-beta, RXR-alpha, RXR-beta, TGIF	AGGTCA
RAT$CRBP1_01	306 (+)	0	100.00	COUP-TF1, RAR-beta, RXR-alpha	AGGTCA
AS$RAR_02	306 (+)	0	100.00	RAR-beta, RXR-alpha	AGGTCA
MOUSE$CRABP2_02	306 (+)	0	100.00	RXR-alpha, RXR-gamma	AGGTCA
AS$RXR_01	306 (+)	0	100.00	COUP-TF1, PPAR-alpha, RXR-alpha	AGGTCA
AS$RXR_02	306 (+)	0	100.00	COUP-TF1, RXR-alpha	AGGTCA
AS$RAR_03	306 (+)	0	100.00	RAR-alpha1, RXR-beta, T3R-beta1	AGGTCA
AS$NGFIB_01	306 (+)	0	100.00	NGFI-B	AGGTCA
MOUSE$CRBP1_01	306 (+)	0	100.00	RAR-alpha1, RAR-beta2, RAR-gamma1	AGGTCA
AS$RAR_07	306 (+)	0	100.00	RAR-alpha1, RXR-alpha	AGGTCA
AS$RAR_08	306 (+)	0	100.00	RAR-alpha1, RXR-alpha	AGGTCA
AS$RAR_09	306 (+)	0	100.00	RAR-alpha1, RXR-alpha	AGGTCA
AS$RAR_10	306 (+)	0	100.00	RAR-alpha1, RXR-alpha	AGGTCA
AS$RAR_11	306 (+)	0	100.00	RAR-alpha1, RXR-alpha	AGGTCA
AS$RAR_12	306 (+)	0	100.00	RAR-alpha1, RXR-alpha	AGGTCA
AS$RAR_13	306 (+)	0	100.00	RAR-alpha1, RXR-alpha	AGGTCA
AS$RAR_14	306 (+)	0	100.00	RAR-alpha1, RXR-alpha	AGGTCA
AS$RAR_15	306 (+)	0	100.00	RAR-alpha1, RXR-alpha	AGGTCA
AS$RAR_16	306 (+)	0	100.00	RAR-alpha1, [illegible]	AGGTCA

AS$RAR_16	306 (+)	0	100.00	RAR-alpha1, RXR-alpha	AGGTCA
AS$RAR_17	306 (+)	0	100.00	RAR-alpha1, RXR-alpha	AGGTCA
AS$RAR_18	306 (+)	0	100.00	RAR-alpha1, RXR-alpha	AGGTCA
AS$RAR_21	306 (+)	0	100.00	RAR-alpha1, RXR-alpha	AGGTCA
AS$TR_15	306 (+)	0	100.00	RXR-alpha, T3R-beta1	AGGTCA
AS$RAR_23	306 (+)	0	100.00	RAR-alpha1, RXR-alpha	AGGTCA
AS$TR_16	306 (+)	0	100.00	T3R-beta1	AGGTCA
AS$TR_17	306 (+)	0	100.00	T3R-beta1	AGGTCA
AS$TR_18	306 (+)	0	100.00	RXR-alpha, T3R-beta1	AGGTCA
AS$RXR_03	306 (+)	0	100.00	RXR-alpha	AGGTCA
MOUSE$PCP2_01	306 (+)	0	100.00	RXR-beta, T3R-alpha1, T3R-beta1	AGGTCA
AS$RXR_04	306 (+)	0	100.00	ARP-1, RXR-alpha	AGGTCA
AS$RXR_05	306 (+)	0	100.00	ARP-1, RAR-alpha1, RXR-alpha	AGGTCA
AS$RXR_6	306 (+)	0	100.00	RXR-alpha, TAF(II)28	AGGTCA
AS$TR_20	306 (+)	0	100.00	RAR-beta, RXR-alpha, T3R-alpha1, T3R-beta1, T3R-beta2	AGGTCA
AS$TR_34	306 (+)	0	100.00	T3R-alpha1, T3R-alpha2	AGGTCA
MOUSE$MYOD_04	306 (+)	0	100.00	ARP-1, RXR-alpha, RXR-gamma, T3R-alpha	AGGTCA
AS$LXRA_01	306 (+)	0	100.00	LXR-alpha, RXR-alpha	AGGTCA
RAT$FAS_04	306 (+)	0	100.00	LXR-alpha, RXR-alpha	AGGTCA
AS$LXRB_01	306 (+)	0	100.00	LXR-beta, RXR-alpha	AGGTCA
AS$RXR_08	306 (+)	0	100.00	RXR-alpha	AGGTCA
AS$LXRB_02	306 (+)	0	100.00	LXR-beta, RXR-alpha	AGGTCA
AS$FXR_01	306 (+)	0	100.00	FXR, RXR-alpha	AGGTCA
AS$VDR_01	306 (+)	0	100.00	RXR-alpha, VDR	AGGTCA
AS$GCNF_02	306 (+)	0	100.00	GCNF	AGGTCA
AS$GCNF_03	306 (+)	0	100.00	GCNF	AGGTCA
AS$GCNF_05	306 (+)	0	100.00	GCNF	AGGTCA
AS$SXR_01	306 (+)	0	100.00	RXR-alpha, SXR	AGGTCA
HS$CYP3A4_01	306 (+)	0	100.00	PXR-1, RXR-alpha, RXR-beta, SXR	AGGTCA
AS$BXR2_01	306 (+)	0	100.00	BXR-beta	AGGTCA
AS$BXR2_02	306 (+)	0	100.00	BXR-beta	AGGTCA
AS$ERR1_03	306 (+)	0	100.00	ERR1	AGGTCA
AS$ERR1_04	306 (+)	0	100.00	ERR1	AGGTCA
AS$ERR1_05	306 (+)	0	100.00	ERR1	AGGTCA
AS$ERR1_07	306 (+)	0	100.00	ERR1	AGGTCA
AS$ERR1_08	306 (+)	0	100.00	ERR1	AGGTCA
AS$ERR1_09	306 (+)	0	100.00	ERR1	AGGTCA
AS$ERR1_11	306 (+)	0	100.00	ERR1	AGGTCA
AS$ERR1_13	306 (+)	0	100.00	ERR1	AGGTCA
AS$ERR1_15	306 (+)	0	100.00	ERR1	AGGTCA
AS$ERR1_17	306 (+)	0	100.00	ERR1	AGGTCA
AS$ERR1_20	306 (+)	0	100.00	ERR1	AGGTCA
AS$ERR1_21	306 (+)	0	100.00	ERR1	AGGTCA
AS$ERR1_22	306 (+)	0	100.00	ERR1	AGGTCA
AS$ERR1_24	306 (+)	0	100.00	ERR1	AGGTCA
AS$ERR1_25	306 (+)	0	100.00	ERR1	AGGTCA
AS$TR_35	306 (+)	0	100.00	T3R-alpha	AGGTCA
AS$REVERBA_01	306 (+)	0	100.00	REVERB-alpha	AGGTCA
AS$REVERBA_02	306 (+)	0	100.00	REVERB-alpha	AGGTCA
AS$REVERBA_03	306 (+)	0	100.00	REVERB-alpha	AGGTCA
AS$REVERBA_04	306 (+)	0	100.00	REVERB-alpha	AGGTCA
AS$REVERBA_05	306 (+)	0	100.00	REVERB-alpha	AGGTCA
MOUSE$CRBP1_02	306 (+)	0	100.00	REVERB-alpha	AGGTCA
MOUSE$PTHR_01	306 (+)	0	100.00	COUP-TF1, RAR-alpha1, RXR-alpha	AGGTCA
RAT$CYP3A23_01	306 (+)	0	100.00	PXR-1	AGGTCA
MOUSE$AQP7_01	306 (+)	0	100.00	PPAR-alpha, PPAR-gamma, RXR-alpha	AGGTCA
XENLA$VITA2_02	306 (-)	0	100.00	ER-alpha, ER-beta1, NHP-1, T3R-alpha1, T3R-beta1	TGACCT
AS$GR_12	306 (-)	0	100.00	GR-alpha, GR-beta	TGACCT
CHICK$OA_12	306 (-)	0	100.00	ER-alpha	TGACCT
CHICK$OA_13	306 (-)	0	100.00	ER-alpha	TGACCT
CHICK$OA_15	306 (-)	0	100.00	ER-alpha	TGACCT
MOUSE$CRABP2_01	306 (-)	0	100.00	RXR-alpha, RXR-gamma	TGACCT
AS$TR_16	306 (-)	0	100.00	T3R-beta1	TGACCT
AS$TR_17	306 (-)	0	100.00	T3R-beta1	TGACCT
AS$TR_18	306 (-)	0	100.00	RXR-alpha, T3R-beta1	TGACCT
RAT$AOX_01	306 (-)	0	100.00	PPAR-alpha, RXR-alpha	TGACCT
BOVIN$OT_01	306 (-)	0	100.00	SF-1	TGACCT
AS$SF1_01	306 (-)	0	100.00	SF-1	TGACCT
AS$FXR_01	306 (-)	0	100.00	FXR, RXR-alpha	TGACCT
HS$SHP1_01	306 (-)	0	100.00	FXR, RXR-alpha	TGACCT
MOUSE$CRABP1_01	306 (-)	0	100.00	TR2-11	TGACCT
CHICK$OA_03	307 (+)	0	100.00	ER-alpha	GGTCA
CHICK$OA_04	307 (+)	0	100.00	c-Fos, c-Jun, ER-alpha	GGTCA
AS$RAR_04	307 (+)	0	100.00	RAR-alpha1, T3R-alpha	GGTCA
AS$RAR_06	307 (+)	0	100.00	RAR-alpha1	GGTCA
AS$TR_08	307 (+)	0	100.00	T3R-alpha	GGTCA
AS$TR_09	307 (+)	0	100.00	RAR-alpha1, T3R-alpha	GGTCA
AS$TR_10	307 (+)	0	100.00	T3R-alpha	GGTCA
NT$CHN50_01	307 (+)	0	100.00	WRKY1, WRKY3, WRKY4	GGTCA
HS$ADH3_01	307 (-)	0	100.00	CAR, RAR-alpha1, RAR-beta, RXR-alpha	TGACC
CHICK$OA_14	307 (-)	0	100.00	ER-alpha	TGACC
RAT$EAI_09	308 (+)	0	100.00		GTCAG
AS$ELK1_13	310 (-)	0	100.00	Elk-1, SAP-1a, SAP-1b, SRF	TTCCTG
DROME$E74_10	311 (+)	0	100.00	E74A	AGGAA
DROME$E74_11	311 (+)	0	100.00	E74A	AGGAA
E74A$CONS_01	311 (+)	0	90.00	E74A	MGGAA
CHICK$CTNT_02	311 (-)	0	100.00	MCBF	ATTCCT
CHICK$CTNT_03	311 (-)	0	100.00	MCBF	ATTCCT
MOUSE$BMG_04	314 (+)	0	100.00	GATA-1	AATCT
XENLA$ACY_01	315 (+)	0	100.00	SRF	ATCTT
XENLA$ACY_01	315 (-)	0	100.00	SRF	AAGAT
HS$ADH2_11	316 (+)	0	100.00	GR	TCTTAT
MOUSE$JUND_08	316 (-)	0	100.00		TATAAGA
AS$PAX2_62	317 (+)	1	87.50	Pax-2.1, Pax-2.2	CTTAAAGCC
CHICK$BAC_05	318 (-)	0	100.00	ETF	TATAA
AD$E3_06	318 (-)	0	100.00	TFIID	TATAA
HS$GG_02	318 (-)	0	100.00	GATA-1	GGCTATAA
HIV1$HIV1_12	318 (-)	0	100.00	DBF4, NC1, TBP, TFIIA, TFIIA-alpha/beta precursor (major), TFIIA-alpha/beta precursor (minor), TFIIA-gamma, TFIIB, TFIID, TMF	TATAA
HIV1$HIV1_13	318 (-)	0	100.00	UBP-1	TATAA
HS$ASCC_04	318 (-)	0	100.00		TATAA
Y$GAL1_10	322 (+)	0	100.00	GAL4	AGCCT
SV$SV40_01	323 (+)	0	100.00	T-Ag	GCCTC
SV$SV40_02	323 (+)	0	100.00	T-Ag	GCCTC
SV$SV40_63	323 (+)	0	100.00	T-Ag	GCCTC
PA$PY_24	323 (-)	0	100.00	T-Ag	GAGGC
PA$PY_25	323 (-)	0	100.00	T-Ag	GAGGC
SV$SV40_02	323 (-)	0	100.00	T-Ag	GAGGC
PA$PY_12	324 (-)	0	100.00		AGAGG
RAT$POMC_03	325 (-)	0	100.00	GR	CAGAG
CHICK$VIT2_01	327 (+)	1	92.31	ER-alpha, NF-1 (-like proteins)	TTGGCTGCAAGCCT
CHICK$VIT2_02	327 (+)	1	92.31	NF-1 (-like proteins)	TTGGCTGCAAGCCT
RAT$ARGL_03	327 (-)	0	100.00	NF-1	AGCCAG
DROME$ADH_06	330 (+)	0	100.00	Adf-1	GCTGC
HS$CMYC_16	330 (+)	0	100.00	CTCF	GCTGC
AS$FTZ_52	330 (+)	0	100.00	Ftz	GCTGCA
DROME$ADH_14	330 (-)	0	100.00	Adf-1	GCAGC
RAV0$RAV0_01	333 (+)	0	100.00	C/EBPalpha	GCAAG
AMV$AMV_02	333 (-)	0	100.00	C/EBPalpha	CTTGC
Y$GAL1_10	336 (+)	0	100.00	GAL4	AGCCT
SV$SV40_01	337 (+)	0	100.00	T-Ag	GCCTC
SV$SV40_02	337 (+)	0	100.00	T-Ag	GCCTC
SV$SV40_63	337 (+)	0	100.00	T-Ag	GCCTC
PA$PY_24	337 (-)	0	100.00	T-Ag	GAGGC
PA$PY_25	337 (-)	0	100.00	T-Ag	GAGGC
SV$SV40_02	337 (-)	0	100.00	T-Ag	GAGGC
HT1$HTLV1_08	338 (+)	0	100.00	c-Ets-1 68	CCTCC
HS$CD8A_03	338 (+)	1	87.50	LyF-1	CCTCCCAAA
AS$LYF1_10	338 (-)	1	87.50	LyF-1	TTTAGGAAG
HS$AACS_02	338 (-)	0	100.00	ARP-1	AGGAGG
AS$PAX2_68	341 (+)	1	87.50	Pax-2.1, Pax-2.2	CCAAAATCA
DROME$FTZ_29	341 (-)	0	100.00	Cad	TTTAGG
MOUSE$ALBU_08	342 (-)	1	88.89	C/EBPalpha, C/EBPbeta, C/EBPbeta(p20), C/EBPgamma, DBP	TATGATTTTG
HS$GRH_03	343 (+)	0	100.00	POU1F1a	TAAAT
MOUSE$BMG_01	345 (-)	0	100.00	GATA-1	TGATT
MOUSE$IGH_55	346 (+)	0	100.00		ATCAT
AS$FOXJ2_46	348 (+)	1	88.89	FOXJ2 (long isoform)	CATAATGTCT
AS$MTTFA_10	348 (-)	0	100.00	mtTFA	TTATG
AS$MTTFA_11	348 (-)	0	100.00	mtTFA	TTATG
Y$SUC2_02	350 (-)	0	100.00	MIG1	AATTA
RAT$DBH_01	350 (-)	0	100.00	ARIX, c-Fos, c-Jun, CREB, CREMtau	AATTA
DROME$YP1_03	351 (-)	1	87.50	DSXF, DSXM	TTACAAATT
RAT$KINK_01	352 (-)	1	87.50	C/EBPalpha, C/EBPbeta	TTTAGAAAT
HS$TNFA_04	353 (-)	0	100.00		AGAAA
I$HSF_01	353 (-)	0	90.00	HSF	AGAAN
F$HSF_01	353 (-)	0	90.00	HSF	AGAAN
HS$GG_22	356 (+)	0	100.00		CTAAT
RAT$OPSIN_01	356 (+)	0	100.00		CTAAT
RAT$PINA_01	356 (+)	0	100.00	Crx	CTAATCT
RAT$PINA_05	356 (+)	0	100.00	Crx	CTAATCT
HS$BPDE_02	356 (+)	0	100.00	Crx	CTAATCT
HS$BG_05	356 (-)	0	100.00	GATA-1	AAGATTAG
SP$SPEC2A_02	357 (+)	0	100.00	SpOtx	TAATCT
EVI1$CONS_07	357 (-)	1	87.50	Evi-1	ACAAGATAA
SP$SPEC2A_02	357 (-)	0	100.00	SpOtx	AGATTA
MOUSE$CTNC_01	357 (-)	0	100.00	GATA-4	AGATTA
BOVIN$LHB_02	357 (-)	0	100.00	Pitx1	AGATTA
V$EVI1_06	357 (-)	1	87.50	Evi-1	ACAAGATAA
MOUSE$BMG_04	358 (+)	0	100.00	GATA-1	AATCT
XENLA$ACY_01	359 (+)	0	100.00	SRF	ATCTT
XENLA$ACY_01	359 (-)	0	100.00	SRF	AAGAT
Y$SPS4_01	360 (-)	1	87.50	NDT80	GCCACAAAA
MOUSE$HES1_01	361 (+)	0	100.00	HES-1	CTTGTG
AS$NCX_28	366 (+)	1	88.89	Ncx	GGCTAATTGG
AS$HOXA3_01	367 (+)	1	87.50	HOXA3, MATalpha1	GGTAATTTG
DROME$EN_06	367 (-)	1	87.50	En	CCAATTAGC
HS$GG_22	368 (+)	0	100.00		CTAAT
RAT$OPSIN_01	368 (+)	0	100.00		CTAAT
XENLA$BMP4_01	368 (+)	0	100.00	Xvent-2	CTAATT
AS$XVENT2_01	368 (+)	0	100.00	Xvent-2	CTAATT
Y$SUC2_02	369 (-)	0	100.00	MIG1	AATTA
RAT$DBH_01	369 (-)	0	100.00	ARIX, c-Fos, c-Jun, CREB, CREMtau	AATTA
RICE$GLUB1_02	369 (-)	1	87.50	MYB5	AACAAACTA
DROME$YP1_03	370 (-)	1	87.50	DSXF, DSXM	TTACAAATT
HS$IGH_04	371 (+)	0	100.00		ATTTG
HS$EGFR_14	371 (-)	0	100.00		CAAAT
MAIZE$PMS1_01	371 (-)	0	100.00		CAAAT
AS$SRY_03	371 (-)	0	100.00	SRY	AACAAAT
RAT$KINT1_04	374 (+)	1	88.89	HNF-3alpha, HNF-3B, HNF-3gamma	TGTTTGTTCT
HS$FIX_04	376 (-)	0	100.00	AR, HNF-4alpha	AACTAA
I$HSF_01	379 (-)	0	90.00	HSF	AGAAN
F$HSF_01	379 (-)	0	90.00	HSF	AGAAN
CAMV$35SR_06	380 (-)	0	100.00	MNB1b	GTAGAA
MAIZE$PEPC_01	380 (-)	0	100.00	MNB1b, MNF1	GTAGAA
DROME$YP1_03	382 (+)	1	87.50	DSXF, DSXM	CTACAAAGT
AS$HOXA3_08	382 (-)	1	87.50	HOXA3	CCTTTGTTG
XENLA$ENGRAILED2_01	383 (-)	0	100.00	LEF-1	CTTTGTA
MOUSE$ECADH_04	383 (-)	0	100.00	LEF-1	CTTTGTA
HS$GHA_07	396 (+)	0	100.00	GR, GR-alpha, GR-beta	TGATCC
MOUSE$TTPA_01	396 (+)	0	100.00	AP-1	TGATC
MOUSE$M1H2KB_07	399 (+)	0	100.00	MBP-1 (1), NF-kappaB1, NF-kappaB2, NF-kappaB2 precursor	TCCCCA
MOUSE$M1H2KB_02	399 (-)	0	100.00	AP-2alpha, AP-2alphaA, AP-2alphaB	TGGGGA
MOUSE$M1H2KB_07	399 (-)	0	100.00	MBP-1 (1), NF-kappaB1, NF-kappaB2, NF-kappaB2 precursor	TGGGGA
Y$GAL3_01	400 (+)	0	100.00	MIG1	CCCCAG
Y$GAL4_01	400 (+)	0	100.00	MIG1	CCCCAG
Y$HAP4_01	400 (+)	0	100.00	MIG1	CCCCAG
SV$SV40_36	400 (-)	0	100.00	AP-2alphaA, AP-2alphaB, AP-2beta	GCCTGGGG
SV$SV40_37	401 (-)	0	100.00		CTGGG
MOUSE$ACRD_01	402 (-)	0	100.00	myogenin	TGCCTGG
Y$CYC1_16	403 (+)	0	100.00	MOT3	CAGGCA
MOUSE$AFEP_14	404 (-)	0	100.00	C/EBPalpha, HNF-1, HNF-1A	TTGCCT
Y$ANB1_05	404 (-)	0	100.00	MOT3	TTGCCT
RAV0$RAV0_01	406 (+)	0	100.00	C/EBPalpha	GCAAG
AMV$AMV_02	406 (-)	0	100.00	C/EBPalpha	CTTGC
DROME$KR_18	406 (-)	1	87.50	Tll	GATTCTTGC
ASPN$YA_02	409 (+)	0	100.00	abaA	AGAATG
I$HSF_01	409 (+)	0	90.00	HSF	AGAAN
F$HSF_01	409 (+)	0	90.00	HSF	AGAAN
ASPN$ABAA_03	409 (-)	0	100.00	abaA	CATTCT
ASPN$ABAA_04	409 (-)	0	100.00	abaA	CATTCT
ASPN$ABAA_05	409 (-)	0	100.00	abaA	CATTCT
ASPN$BRLA_03	409 (-)	0	100.00	abaA	CATTCT
ASPN$RODA_05	409 (-)	0	100.00	abaA	CATTCT
ABAA$CONS_01	409 (-)	0	91.67	abaA	CATTCY
ASPN$BRLA_05	409 (-)	0	100.00	abaA	CATTCT
RAT$ME_01	413 (+)	0	100.00	T3R-alpha, T3R-beta1, T3R-beta2	TGGGGT
AS$SRY_18	413 (-)	1	88.89	SRY	ACAAACTCCA
RAT$KINK_02	416 (+)	1	88.89	HNF-3alpha, HNF-3B, HNF-3gamma	TGTTTGTTTT
AS$GAMYB_16	416 (-)	0	100.00	GAMYB	AACAAACC
MOUSE$ALBU_20	417 (+)	1	88.89	HNF-1A, HNF-1B, HNF-1C, HNF-3	GTTTGTTCTT
HS$TF_12	417 (+)	1	87.50	EBP40, EBP45	GTTTGCTTT
MOUSE$HNF3B_02	417 (+)	0	100.00	HNF-3alpha, HNF-3B	GTTTGTTTT
AS$AREB6_42	417 (+)	0	100.00	AREB6	GTTTG
AS$AREB6_43	417 (+)	0	100.00	AREB6	GTTTG
AS$AREB6_45	417 (+)	0	100.00	AREB6	GTTTG
AS$AREB6_47	417 (+)	0	100.00	AREB6	GTTTG
AS$AREB6_51	417 (+)	0	100.00	AREB6	GTTTG
RICE$GLUB1_03	417 (-)	0	100.00	MYB5	AACAAAC
AS$FOXJ1_05	418 (+)	0	100.00	FOXJ1	TTTGTTT
RAT$IGFBP1_01	418 (-)	0	100.00	HNF-3alpha, HNF-3B, HNF-3gamma	AAACAAA
AS$FOXO3_01	418 (-)	0	100.00	FOXO3a	AAAACAAA
PEA$IAA45_06	419 (-)	0	100.00		AAAACAA
MOUSE$CDX2_01	420 (+)	0	100.00	FOXM1a, FOXM1b, HNF-3alpha, HNF-3B	TGTTT
RAT$FABPI_04	420 (+)	0	100.00	FOXM1a, FOXM1b, HNF-3alpha, HNF-3B	TGTTT
HS$ALP_02	420 (+)	0	100.00	HNF-3alpha, HNF-3B	TGTTT
MOUSE$ECADH_03	420 (-)	0	100.00	FOXM1a, FOXM1b, HNF-3alpha, HNF-3B	AAACA
AS$PAX2_68	420 (-)	1	87.50	Pax-2.1, Pax-2.2	CCAAAATCA
HS$ADH2_11	420 (-)	0	100.00	GR	AAAACA
AS$PAX2_60	421 (-)	1	87.50	Pax-2.1, Pax-2.2	TCCAAAGAC
Y$SUC2_01	422 (+)	0	100.00	MIG1	TTTTT
AS$PF1_01	422 (+)	0	100.00	PF1	TTTTT
AS$PF1_02	422 (+)	0	100.00	PF1	TTTTT
HS$P21WAF1_11	422 (+)	1	87.50	STAT5B	TTCTTGGAA
AS$STAT5A_43	422 (-)	1	87.50	STAT5A	TTCCAAGAA
CHICK$LYS_19	424 (+)	1	87.50	AP-3, C/EBPalpha	TTTGGAAAT
CHICK$LYS_24	424 (+)	1	87.50	AP-3, C/EBPalpha, TGGCA-binding protein	TTTGGCAAG
SV$SV40_42	426 (+)	0	100.00		TGGAAAG
HS$IGKL_01	426 (-)	0	100.00		TTTCCA
SV$SV40_25	426 (-)	0	100.00		CTTTCCA
HS$IFI616_01	427 (+)	0	100.00	ISGF-3	GGAAA
MOUSE$IL5_02	427 (+)	0	100.00	NF-ATc, NF-ATp, NF-ATx	GGAAA
HS$IL4_01	427 (+)	0	100.00		GGAAA
MOUSE$IL4_06	427 (+)	0	100.00		GGAAA
MOUSE$IL4_07	427 (+)	0	100.00		GGAAA
MOUSE$IL4_08	427 (+)	0	100.00		GGAAA
HS$IL5_02	427 (+)	0	100.00		GGAAA
HS$IL13_01	427 (+)	0	100.00		GGAAA
MOUSE$CD40L_01	427 (+)	0	100.00		GGAAA
MOUSE$CD40L_02	427 (+)	0	100.00		GGAAA
MOUSE$CD40L_03	427 (+)	0	100.00		GGAAA
MOUSE$CD40L_04	427 (+)	0	100.00		GGAAA
HS$CD40L_01	427 (+)	0	100.00		GGAAA
HS$CD40L_02	427 (+)	0	100.00		GGAAA
HS$IFNG_03	427 (+)	0	100.00		GGAAA
HS$IFNG_04	427 (+)	0	100.00		GGAAA
HS$TNFA_05	427 (+)	0	100.00		GGAAA
MOUSE$MCP3_01	427 (+)	0	100.00		GGAAA
HS$TNFSF6_01	427 (+)	0	100.00		GGAAA
HCMV$IE1_16	427 (-)	0	100.00	NF-1	CTTTCC
RABBIT$UG_03	429 (-)	0	100.00	PR B	AGTCCTTT
CHICK$VIT2_20	431 (+)	1	87.50	Sp1	AGGAGTGTT
WHEAT$LMWG1D1_03	434 (-)	1	87.50	ESBF I	TGTAAAAGT
AS$MEIS1AHOXA9_07	435 (-)	0	100.00	HOXA9, Meis-1a	TAACAG
CHICK$LYS_21	436 (-)	1	87.50	AP-3, C/EBPalpha	ATTGCAACA
MMTV$MMTV_06	437 (+)	0	100.00	GR, PR B	GTTACA
AS$PR_03	437 (+)	0	100.00	PR B	GTTACA
HS$EGFR_14	438 (+)	1	87.50		TTACAATAT
AS$HFH2_29	441 (+)	1	90.91	FOXD3	CAATGTTTGTTT
AS$HOX1_02	441 (+)	1	87.50	HOX1, HOX2, HOX3, HOX4, HOX5, HOX6, HOX7	CAATCATTG
AS$ATHB2_02	441 (+)	1	87.50	ATHB-2, GLABRA2	CAATCATTG
HS$GG_26	441 (-)	0	100.00	GATA-1	AGATTG
AS$HDE_02	441 (-)	1	87.50	CPHB-1	CAATGATTG
AS$ATHB2_01	441 (-)	1	87.50	ATHB-2, GLABRA2	CAATGATTG
MOUSE$BMG_04	442 (+)	0	100.00	GATA-1	AATCT
XENLA$ACY_01	443 (+)	0	100.00	SRF	ATCTT
XENLA$ACY_01	443 (-)	0	100.00	SRF	AAGAT
FY$MFM1_02	443 (-)	1	87.50	mat1-Mc, ste11	AACAAAGAA
AS$SRY_21	443 (-)	1	88.89	SRY	AAACAAAGCT
HIV1$HIV1_36	444 (+)	0	100.00	LEF-1	TCTTTGT
XENLA$TWIN_02	444 (+)	0	100.00	LEF-1	TCTTTGTT
MOUSE$LY49A_01	445 (+)	0	100.00	TCF-1(P)	CTTTGTT
XENLA$ENGRAILED2_03	445 (+)	0	100.00	LEF-1	CTTTGTT
HS$CD3E_01	445 (-)	0	100.00	LEF-1, mat1-Mc, Sox-13, Sox-5, SRY, TCF-1, TCF-1A, TCF-1B, TCF-1C, TCF-1E, TCF-1F, TCF-1G	AACAAAG
MOUSE$SRY_01	445 (-)	0	100.00	SRY	AACAAAG
HS$SRY_01	445 (-)	0	100.00	SRY	AACAAAG
HS$ADA_08	445 (-)	0	100.00	LEF-1	AACAAAG
AS$TCF1_01	445 (-)	0	100.00	TCF-1B	AACAAAG
AS$TCF1_02	445 (-)	0	100.00	TCF-1B	AACAAAG
AS$SOX15_02	445 (-)	0	100.00	Sox15	AACAAAG
AS$FOXJ1_05	446 (+)	0	100.00	FOXJ1	TTTGTTT
RAT$IGFBP1_01	446 (-)	0	100.00	HNF-3alpha, HNF-3B, HNF-3gamma	AAACAAA
AS$SRY_13	446 (-)	0	100.00	SRY	TTGAAACAAA
AS$PAX2_59	447 (-)	1	87.50	Pax-2.1, Pax-2.2	TTGAAAGAA
HS$IL2_15	448 (+)	0	100.00	c-Fos, c-Jun, Fra-1, JunB	TGTTTCA
MOUSE$IL2_09	448 (+)	0	100.00	AP-1, c-Fos, c-Jun, Fra-1	TGTTTCA
MOUSE$CDX2_01	448 (+)	0	100.00	FOXM1a, FOXM1b, HNF-3alpha, HNF-3B	TGTTT
RAT$FABPI_04	448 (+)	0	100.00	FOXM1a, FOXM1b, HNF-3alpha, HNF-3B	TGTTT
HS$ALP_02	448 (+)	0	100.00	HNF-3alpha, HNF-3B	TGTTT
MOUSE$ECADH_03	448 (-)	0	100.00	FOXM1a, FOXM1b, HNF-3alpha, HNF-3B	AAACA
AS$AREB6_44	449 (+)	0	100.00	AREB6	GTTTC
AS$AREB6_46	449 (+)	0	100.00	AREB6	GTTTC
AS$AREB6_48	449 (+)	0	100.00	AREB6	GTTTC
AS$AREB6_49	449 (+)	0	100.00	AREB6	GTTTC
AS$AREB6_50	449 (+)	0	100.00	AREB6	GTTTC
AS$AREB6_52	449 (+)	0	100.00	AREB6	GTTTC
AS$AREB6_53	449 (+)	0	100.00	AREB6	GTTTC
MOUSE$MBP_04	451 (+)	0	100.00	TFIID	TTCAAA
HS$CD3E_02	451 (+)	0	100.00	SRY, TCF-1A	TTCAAAG
AS$TCF1_03	451 (+)	0	100.00	TCF-1B	TTCAAAG
HS$MMP7_02	451 (+)	0	100.00	TCF-4	TTCAAAG
HS$CDC2_10	451 (-)	0	100.00		TTGAA
HS$CYCA_06	451 (-)	0	100.00		TTGAA
HS$CDC25C_05	451 (-)	0	100.00		TTGAA
XENLA$SIAMOIS_03	451 (-)	0	100.00	TCF-3	CTTTGAA
HS$MMP7_01	451 (-)	0	100.00	TCF-4	CTTTGAA
AS$STAT5A_40	451 (-)	1	87.50	STAT5A	CACTATGAA
DROME$UBX_24	452 (-)	0	100.00	LEF-1	TTTGA
AS$STAT5A_34	453 (+)	1	87.50	STAT5A	CAAACTGAA
AS$DSF_12	454 (+)	1	90.00	DSF	AAAGTCAAATT
HS$IFNB_02	455 (+)	0	100.00	IRF-1, IRF-2	AAGTGA
HS$IFNB_09	455 (+)	0	100.00		AAGTGA
PRDIBF1$CONS	455 (+)	1	88.89	PRDI-BF1	AAGTGAAAGT
Y$INO1_01	455 (+)	1	87.50	NBF	ATGTGAAAT
Y$SUC2_02	461 (+)	0	100.00	MIG1	AATTA
RAT$DBH_01	461 (+)	0	100.00	ARIX, c-Fos, c-Jun, CREB, CREMtau	AATTA
XENLA$BMP4_01	461 (-)	0	100.00	Xvent-2	CTAATT
AS$XVENT2_01	461 (-)	0	100.00	Xvent-2	CTAATT
RAT$KINK_01	462 (+)	1	87.50	C/EBPalpha, C/EBPbeta	TTTAGAAAT
HS$GG_22	462 (-)	0	100.00		CTAAT
RAT$OPSIN_01	462 (-)	0	100.00		CTAAT
DROME$YP1_03	463 (+)	1	87.50	DSXF, DSXM	TTACAAATT
HS$TNFA_04	465 (+)	0	100.00		AGAAA
I$HSF_01	465 (+)	0	90.00	HSF	AGAAN
F$HSF_01	465 (+)	0	90.00	HSF	AGAAN
DROME$EN_01	466 (+)	1	88.89	En, Eve, Ftz, Prd, Zen-1, Zen-2	TAAATTAAAT
AS$NKX61_02	467 (-)	0	100.00	Nkx6-1	TTAATTT
Y$SUC2_02	468 (+)	0	100.00	MIG1	AATTA
RAT$DBH_01	468 (+)	0	100.00	ARIX, c-Fos, c-Jun, CREB, CREMtau	AATTA
AS$TBP_50	468 (+)	1	90.00	TBP	AGTTAAATTCC
Y$MEL1_02	469 (+)	0	100.00	MIG1	ATTAA
CHICK$MGF_01	469 (+)	0	100.00	Gbx2	ATTAA
CHICK$MGF_02	469 (-)	0	100.00	Gbx2	TTAAT
DROME$PEL_01	470 (+)	0	100.00	TBP	TTAAATT
Y$MAL63_01	470 (-)	0	100.00	MIG1	TTTAA
HS$GRH_03	471 (+)	0	100.00	POU1F1a	TAAAT
HS$GHA_07	472 (-)	0	100.00	GR, GR-alpha, GR-beta	GGAATTT
AS$ETS1_02	473 (-)	1	87.50	Ets-1 deltaVII, PU.1	GGAGGAAGT
AS$SPZ1_16	473 (-)	1	87.50	Spz1	GGAGGATTT
AS$SPZ1_21	473 (-)	1	87.50	Spz1	GGAGGGATT
CHICK$CTNT_02	474 (+)	0	100.00	MCBF	ATTCCT
CHICK$CTNT_03	474 (+)	0	100.00	MCBF	ATTCCT
MOUSE$KROX_01	475 (+)	0	100.00	Elk-1, SAP-1a, SAP-1b, SRF	TTCCTC
MOUSE$FCGR3A_02	475 (+)	0	100.00	PU.1	TTCCTC
DROME$E74_10	475 (-)	0	100.00	E74A	AGGAA
DROME$E74_11	475 (-)	0	100.00	E74A	AGGAA
E74A$CONS_01	475 (-)	0	90.00	E74A	MGGAA
HS$UPA_01	475 (-)	0	100.00	c-Ets-2, PEA3	GGAGGAA
HS$GG_25	476 (-)	0	100.00	PPUR	AGGAGGA
HT1$HTLV1_08	477 (+)	0	100.00	c-Ets-1 68	CCTCC
AS$HOXA3_07	477 (+)	1	87.50	HOXA3	CCTACTGTA
HS$AACS_02	477 (-)	0	100.00	ARP-1	AGGAGG
MAIZE$A1_02	480 (+)	1	87.50	C1 (long form), C1 (short form), C1-I, P (long form), P (short form)	CCGGTAGTT
LPV$LPV_02	482 (+)	1	87.50	Pu box binding factor (BJA-B)	TGTGGTTAG
LPV$LPV_03	482 (+)	1	87.50	Pu box binding factor	TGTGGTTAG
RICE$GLUB1_02	484 (-)	1	87.50	MYB5	AACAAACTA
MOUSE$RVL3_03	485 (-)	0	100.00	RXR-alpha	TAACT
HS$FIX_04	487 (-)	0	100.00	AR, HNF-4alpha	AACTAA
MOUSE$RVL3_03	489 (-)	0	100.00	RXR-alpha	TAACT
MOUSE$MYOGN_01	493 (+)	0	100.00	ARP-1	AGGTCT
AS$ERR1_19	493 (+)	0	100.00	ERR1	AGGTCT
RAT$3KAT_01	493 (-)	0	100.00	PPAR-alpha, RXR-alpha	AGACCT
HS$LCK_01	499 (+)	1	87.50	LyF-1	TCTCCCAGG
AS$LYF1_07	499 (-)	1	87.50	LyF-1	CCTGGGAGA
HS$CD11B_01	500 (+)	0	100.00	Sp1	CGCCC
SV$SV40_63	501 (-)	0	100.00	T-Ag	TGGGC
RAT$SPI23_02	502 (+)	1	87.50	C/EBPalpha	CCCAGAAAT
SV$SV40_37	502 (-)	0	100.00		CTGGG
AS$ELK1_13	504 (-)	0	100.00	Elk-1, SAP-1a, SAP-1b, SRF	TTCCTG
DROME$E74_10	505 (+)	0	100.00	E74A	AGGAA
DROME$E74_11	505 (+)	0	100.00	E74A	AGGAA
E74A$CONS_01	505 (+)	0	90.00	E74A	MGGAA
CHICK$CTNT_02	505 (-)	0	100.00	MCBF	ATTCCT
CHICK$CTNT_03	505 (-)	0	100.00	MCBF	ATTCCT
CHICK$AAC_08	505 (-)	0	100.00	MCBF, MF3	CATTCCT
MCBF$CONS	505 (-)	0	100.00	MCBF	CATTCCT
ASPN$RODA_01	506 (+)	0	100.00	abaA	GGAATG
ASPN$RODA_02	506 (+)	0	100.00	abaA	GGAATG
ASPN$RODA_03	506 (+)	0	100.00	abaA	GGAATG
ASPN$YA_01	506 (-)	0	100.00	abaA	CATTCC
ASPN$ABAA_01	506 (-)	0	100.00	abaA	CATTCC
ASPN$ABAA_02	506 (-)	0	100.00	abaA	CATTCC
ASPN$BRLA_01	506 (-)	0	100.00	abaA	CATTCC
ASPN$BRLA_02	506 (-)	0	100.00	abaA	CATTCC
ASPN$BRLA_04	506 (-)	0	100.00	abaA	CATTCC
ASPN$WETA_01	506 (-)	0	100.00	abaA	CATTCC
ASPN$WETA_02	506 (-)	0	100.00	abaA	CATTCC
ASPN$WETA_03	506 (-)	0	100.00	abaA	CATTCC
ASPN$WETA_04	506 (-)	0	100.00	abaA	CATTCC
ASPN$RODA_04	506 (-)	0	100.00	abaA	CATTCC
ABAA$CONS_01	506 (-)	0	91.67	abaA	CATTCY
ASPN$ABAA_06	506 (-)	0	100.00	abaA	CATTCC
AS$PAX2_44	508 (+)	1	87.50	Pax-2.1, Pax-2.2	AATGAACAC
HS$ADH3_01	510 (+)	0	100.00	CAR, RAR-alpha1, RAR-beta, RXR-alpha	TGAAC
RAT$CYTOPB_01	510 (-)	0	100.00	GR	TGTTCA
AS$GR_07	510 (-)	0	100.00	GR	TGTTCA
AS$RAR_05	510 (-)	0	100.00	RAR-alpha1	GTTCA
AS$RAR_06	510 (-)	0	100.00	RAR-alpha1	GTTCA
AS$TR_06	510 (-)	0	100.00	T3R-alpha	GTTCA
AS$TR_07	510 (-)	0	100.00	T3R-alpha	GTTCA
AS$TR_08	510 (-)	0	100.00	T3R-alpha	GTTCA
AS$TR_11	510 (-)	0	100.00	T3R-alpha	GTTCA
MOUSE$RARA2_01	510 (-)	0	100.00	RAR-alpha1, RAR-beta2, RAR-gamma1	GTTCA
MOUSE$CRISP1_03	510 (-)	0	100.00	AR	TGTTCA
RAT$LHB_06	513 (-)	1	87.50	SF-1	TGACCTTGT
MAIZE$PEPC_01	515 (-)	0	100.00	MNB1b, MNF1	TGCCCTT
AS$RAR_16	516 (+)	0	100.00	RAR-alpha1, RXR-alpha	AGGGCA
HS$ALDA_06	516 (+)	0	100.00	TR2-11	AGGGCA
MOUSE$CRABP2_01	516 (-)	0	100.00	RXR-alpha, RXR-gamma	TGCCCT
HS$CABD9_01	516 (-)	0	100.00	RXR-alpha, VDR	TGCCCT
CHICK$CYP2H1_05	516 (-)	0	100.00	CXR, RXR-gamma	TGCCCT
CHICK$BAG_03	517 (+)	0	100.00		GGGCA
RAT$NF1_01	517 (+)	0	100.00	LF-A1	GGGCA
PIG$UPA_09	517 (+)	0	100.00	Sp1	GGGCAG
HS$CATHD_01	517 (+)	0	100.00	ER-alpha, Sp1	GGGCA
RAT$VEGF_01	517 (+)	0	100.00	ER-alpha, ER-beta	GGGCA
CHICK$BAG_03	517 (-)	0	100.00		TGCCC
RAT$VEGF_02	517 (-)	0	100.00	ER-alpha, ER-beta	TGCCC
DROME$ADH_14	519 (+)	0	100.00	Adf-1	GCAGC
DROME$ADH_06	519 (-)	0	100.00	Adf-1	GCTGC
HS$CMYC_16	519 (-)	0	100.00	CTCF	GCTGC
AS$ETS1_09	523 (+)	1	88.89	c-Ets-1	CCCGGAAGTG
MOUSE$COX5_03	523 (+)	1	88.89		CCCGGAAGTG
AS$CETS1P54_06	523 (+)	1	88.89	c-Ets-1 54	CCCGGAAGTG
AS$NRF2_06	523 (+)	1	88.89	NRF-2	CCCGGAAGTG
HS$BAC_04	524 (-)	0	100.00	Elk-1, SAP-1a, SAP-1b, SRF	TTCCGA
AD5$E1A_17	525 (+)	0	100.00	PEA3	CGGAAGT
E4TF1$CONS	525 (+)	0	100.00	GABP	CGGAAGTG
EF1A$CONS	525 (+)	0	100.00	GABP	CGGAAGTG
E74A$CONS_01	525 (+)	0	90.00	E74A	MGGAA
MOUSE$RPL32_01	526 (+)	0	100.00	f(alpha)-f(epsilon), HrpF, XrpFI	GGAAG
HS$TNFA_02	526 (+)	0	100.00		GGAAG
HS$TNFA_03	526 (+)	0	100.00		GGAAG
XENLA$RPL14_01	526 (-)	0	100.00	HrpF, XrpFI	CTTCC
HS$MHC2IN_01	526 (-)	1	88.89		TTTCACTTCC
Y$TPI_01	526 (-)	0	100.00	GCR1	CTTCC
MOUSE$IL2RA_03	526 (-)	0	100.00	c-Ets-1, Elf-1	ACTTCC
HS$IFNB_02	528 (+)	0	100.00	IRF-1, IRF-2	AAGTGA
HS$IFNB_09	528 (+)	0	100.00		AAGTGA
MOUSE$GSHPX1_01	532 (-)	0	100.00	PU.1	CTTCTC
I$HSF_01	533 (+)	0	90.00	HSF	AGAAN
F$HSF_01	533 (+)	0	90.00	HSF	AGAAN
AS$AHRARNT_50	537 (+)	0	100.00	AhR, Arnt	GCGTG
RAT$CYTOP_04	537 (-)	0	100.00	AhR, Arnt	CACGC
MAIZE$ADH1P_01	538 (+)	0	100.00		CGTGG
MAIZE$ADH1P_03	538 (+)	0	100.00		CGTGG
AS$ZIC_08	538 (+)	1	87.50	Zic1	CGGGGAGTC
AS$ALFIN1_06	539 (+)	0	100.00	Alfin1	GTGGAG
CHICK$LYS_18	541 (+)	0	100.00	AP-1	GGAGTCA
HS$UPA_08	541 (+)	1	87.50	UEF-1	GGAGTCATG
HS$PAI1_02	541 (+)	0	100.00	SMAD-3, SMAD-4	GGAGTCA
AS$CF1_34	541 (+)	1	87.50	CF1	GGGGTCATT
Y$HIS3_02	542 (+)	0	100.00	GCN4	GAGTCA
Y$ILV1_01	542 (+)	0	100.00	GCN4	GAGTCA
PEA$PSL_02	542 (+)	0	100.00	TGA1a	GAGTCA
Y$HIS3_05	542 (-)	0	100.00	GCN4	TGACTC
Y$HIS4_08	542 (-)	0	100.00	GCN4	TGACTC
Y$HIS4_12	542 (-)	0	100.00	GCN4	TGACTC
HS$CYP24_02	542 (-)	0	100.00	VDR	TGACTC
NT$CHN50_01	543 (+)	0	100.00	WRKY1, WRKY3, WRKY4	AGTCA
MOUSE$IL4_01	545 (+)	0	100.00	NF-CLE0a, NF-CLE0b	TCATTT
HS$GMCSF_03	546 (+)	0	100.00	YY1	CATTT
HS$ARR_02	546 (+)	0	100.00	Crx	CATTTAG
HS$GRH_03	547 (-)	0	100.00	POU1F1a	TAAAT
DROME$FTZ_29	548 (+)	0	100.00	Cad	TTTAGG
CHICK$OA_02	551 (+)	0	100.00	ARP-1, COUP, COUP-TF1, PPAR-alpha, RXR-alpha	AGGTCA
XENLA$VITA2_02	551 (+)	0	100.00	ER-alpha, ER-beta1, NHP-1, T3R-alpha1, T3R-beta1	AGGTCA
PPAR$CONS_01	551 (+)	0	100.00	PPAR-alpha	AGGTCA
AS$GR_12	551 (+)	0	100.00	GR-alpha, GR-beta	AGGTCA
RAT$CRBP2_01	551 (+)	0	100.00	COUP-TF1, PPAR-alpha, RAR-alpha1, RAR-beta, RXR-alpha, RXR-beta, TGIF	AGGTCA
RAT$CRBP1_01	551 (+)	0	100.00	COUP-TF1, RAR-beta, RXR-alpha	AGGTCA
AS$RAR_02	551 (+)	0	100.00	RAR-beta, RXR-alpha	AGGTCA
MOUSE$CRABP2_02	551 (+)	0	100.00	RXR-alpha, RXR-gamma	AGGTCA
AS$RXR_01	551 (+)	0	100.00	COUP-TF1, PPAR-alpha, RXR-alpha	AGGTCA
AS$RXR_02	551 (+)	0	100.00	COUP-TF1, RXR-alpha	AGGTCA
AS$RAR_03	551 (+)	0	100.00	RAR-alpha1, RXR-beta, T3R-beta1	AGGTCA
AS$NGFIB_01	551 (+)	0	100.00	NGFI-B	AGGTCA
MOUSE$CRBP1_01	551 (+)	0	100.00	RAR-alpha1, RAR-beta2, RAR-gamma1	AGGTCA
AS$RAR_07	551 (+)	0	100.00	RAR-alpha1, RXR-alpha	AGGTCA
AS$RAR_08	551 (+)	0	100.00	RAR-alpha1, RXR-alpha	AGGTCA
AS$RAR_09	551 (+)	0	100.00	RAR-alpha1, RXR-alpha	AGGTCA
AS$RAR_10	551 (+)	0	100.00	RAR-alpha1, RXR-alpha	AGGTCA
AS$RAR_11	551 (+)	0	100.00	RAR-alpha1, RXR-alpha	AGGTCA
AS$RAR_12	551 (+)	0	100.00	RAR-alpha1, RXR-alpha	AGGTCA
AS$RAR_13	551 (+)	0	100.00	RAR-alpha1, RXR-alpha	AGGTCA
AS$RAR_14	551 (+)	0	100.00	RAR-alpha1, RXR-alpha	AGGTCA
AS$RAR_15	551 (+)	0	100.00	RAR-alpha1, RXR-alpha	AGGTCA
AS$RAR_16	551 (+)	0	100.00	RAR-alpha1, RXR-alpha	AGGTCA
AS$RAR_17	551 (+)	0	100.00	RAR-alpha1, RXR-alpha	AGGTCA
AS$RAR_18	551 (+)	0	100.00	RAR-alpha1, RXR-alpha	AGGTCA
AS$RAR_21	551 (+)	0	100.00	RAR-alpha1, RXR-alpha	AGGTCA
AS$TR_15	551 (+)	0	100.00	RXR-alpha, T3R-beta1	AGGTCA
AS$RAR_23	551 (+)	0	100.00	RAR-alpha1, RXR-alpha	AGGTCA
AS$TR_16	551 (+)	0	100.00	T3R-beta1	AGGTCA
AS$TR_17	551 (+)	0	100.00	T3R-beta1	AGGTCA
AS$TR_18	551 (+)	0	100.00	RXR-alpha, T3R-beta1	AGGTCA
AS$RXR_03	551 (+)	0	100.00	RXR-alpha	AGGTCA
MOUSE$PCP2_01	551 (+)	0	100.00	RXR-beta, T3R-alpha1, T3R-beta1	AGGTCA
AS$RXR_04	551 (+)	0	100.00	ARP-1, RXR-alpha	AGGTCA
AS$RXR_05	551 (+)	0	100.00	ARP-1, RAR-alpha1, RXR-alpha	AGGTCA
AS$RXR_6	551 (+)	0	100.00	RXR-alpha, TAF(II)28	AGGTCA
AS$TR_20	551 (+)	0	100.00	RAR-beta, RXR-alpha, T3R-alpha1, T3R-beta1, T3R-beta2	AGGTCA
AS$TR_34	551 (+)	0	100.00	T3R-alpha1, T3R-alpha2	AGGTCA
MOUSE$MYOD_04	551 (+)	0	100.00	ARP-1, RXR-alpha, RXR-gamma, T3R-alpha	AGGTCA
AS$LXRA_01	551 (+)	0	100.00	LXR-alpha, RXR-alpha	AGGTCA
RAT$FAS_04	551 (+)	0	100.00	LXR-alpha, RXR-alpha	AGGTCA
AS$LXRB_01	551 (+)	0	100.00	LXR-beta, RXR-alpha	AGGTCA
AS$RXR_08	551 (+)	0	100.00	RXR-alpha	AGGTCA
AS$LXRB_02	551 (+)	0	100.00	LXR-beta, RXR-alpha	AGGTCA
AS$FXR_01	551 (+)	0	100.00	FXR, RXR-alpha	AGGTCA
AS$VDR_01	551 (+)	0	100.00	RXR-alpha, VDR	AGGTCA
AS$GCNF_02	551 (+)	0	100.00	GCNF	AGGTCA
AS$GCNF_03	551 (+)	0	100.00	GCNF	AGGTCA
AS$GCNF_05	551 (+)	0	100.00	GCNF	AGGTCA
AS$SXR_01	551 (+)	0	100.00	RXR-alpha, SXR	AGGTCA
HS$CYP3A4_01	551 (+)	0	100.00	PXR-1, RXR-alpha, RXR-beta, SXR	AGGTCA
AS$BXR2_01	551 (+)	0	100.00	BXR-beta	AGGTCA
AS$BXR2_02	551 (+)	0	100.00	BXR-beta	AGGTCA
AS$ERR1_03	551 (+)	0	100.00	ERR1	AGGTCA
AS$ERR1_04	551 (+)	0	100.00	ERR1	AGGTCA
AS$ERR1_05	551 (+)	0	100.00	ERR1	AGGTCA
AS$ERR1_07	551 (+)	0	100.00	ERR1	AGGTCA
AS$ERR1_08	551 (+)	0	100.00	ERR1	AGGTCA
AS$ERR1_09	551 (+)	0	100.00	ERR1	AGGTCA
AS$ERR1_11	551 (+)	0	100.00	ERR1	AGGTCA
AS$ERR1_13	551 (+)	0	100.00	ERR1	AGGTCA
AS$ERR1_15	551 (+)	0	100.00	ERR1	AGGTCA
AS$ERR1_17	551 (+)	0	100.00	ERR1	AGGTCA
AS$ERR1_20	551 (+)	0	100.00	ERR1	AGGTCA
AS$ERR1_21	551 (+)	0	100.00	ERR1	AGGTCA
AS$ERR1_22	551 (+)	0	100.00	ERR1	AGGTCA
AS$ERR1_24	551 (+)	0	100.00	ERR1	AGGTCA
AS$ERR1_25	551 (+)	0	100.00	ERR1	AGGTCA
AS$TR_35	551 (+)	0	100.00	T3R-alpha	AGGTCA
AS$REVERBA_01	551 (+)	0	100.00	REVERB-alpha	AGGTCA
AS$REVERBA_02	551 (+)	0	100.00	REVERB-alpha	AGGTCA
AS$REVERBA_03	551 (+)	0	100.00	REVERB-alpha	AGGTCA
AS$REVERBA_04	551 (+)	0	100.00	REVERB-alpha	AGGTCA
AS$REVERBA_05	551 (+)	0	100.00	REVERB-alpha	AGGTCA
MOUSE$CRBP1_02	551 (+)	0	100.00	REVERB-alpha	AGGTCA
MOUSE$PTHR_01	551 (+)	0	100.00	COUP-TF1, RAR-alpha1, RXR-alpha	AGGTCA
RAT$CYP3A23_01	551 (+)	0	100.00	PXR-1	AGGTCA
MOUSE$AQP7_01	551 (+)	0	100.00	PPAR-alpha, PPAR-gamma, RXR-alpha	AGGTCA
XENLA$VITA2_02	551 (-)	0	100.00	ER-alpha, ER-beta1, NHP-1, T3R-alpha1, T3R-beta1	TGACCT
AS$GR_12	551 (-)	0	100.00	GR-alpha, GR-beta	TGACCT
CHICK$OA_12	551 (-)	0	100.00	ER-alpha	TGACCT
CHICK$OA_13	551 (-)	0	100.00	ER-alpha	TGACCT
CHICK$OA_15	551 (-)	0	100.00	ER-alpha	TGACCT
MOUSE$CRABP2_01	551 (-)	0	100.00	RXR-alpha, RXR-gamma	TGACCT
AS$TR_16	551 (-)	0	100.00	T3R-beta1	TGACCT
AS$TR_17	551 (-)	0	100.00	T3R-beta1	TGACCT
AS$TR_18	551 (-)	0	100.00	RXR-alpha, T3R-beta1	TGACCT
RAT$AOX_01	551 (-)	0	100.00	PPAR-alpha, RXR-alpha	TGACCT
BOVIN$OT_01	551 (-)	0	100.00	SF-1	TGACCT
AS$SF1_01	551 (-)	0	100.00	SF-1	TGACCT
AS$FXR_01	551 (-)	0	100.00	FXR, RXR-alpha	TGACCT
HS$SHP1_01	551 (-)	0	100.00	FXR, RXR-alpha	TGACCT
MOUSE$CRABP1_01	551 (-)	0	100.00	TR2-11	TGACCT
CHICK$OA_03	552 (+)	0	100.00	ER-alpha	GGTCA
CHICK$OA_04	552 (+)	0	100.00	c-Fos, c-Jun, ER-alpha	GGTCA
AS$RAR_04	552 (+)	0	100.00	RAR-alpha1, T3R-alpha	GGTCA
AS$RAR_06	552 (+)	0	100.00	RAR-alpha1	GGTCA
AS$TR_08	552 (+)	0	100.00	T3R-alpha	GGTCA
AS$TR_09	552 (+)	0	100.00	RAR-alpha1, T3R-alpha	GGTCA
AS$TR_10	552 (+)	0	100.00	T3R-alpha	GGTCA
NT$CHN50_01	552 (+)	0	100.00	WRKY1, WRKY3, WRKY4	GGTCA
HS$ADH3_01	552 (-)	0	100.00	CAR, RAR-alpha1, RAR-beta, RXR-alpha	TGACC
CHICK$OA_14	552 (-)	0	100.00	ER-alpha	TGACC
RAT$EAI_09	553 (+)	0	100.00		GTCAG
HS$APOB_11	561 (+)	0	100.00	LF-A1	CCTTGC
AMV$AMV_02	562 (+)	0	100.00	C/EBPalpha	CTTGC
RAV0$RAV0_01	562 (-)	0	100.00	C/EBPalpha	GCAAG
MOUSE$MT1_01	564 (+)	0	100.00	Sp1	TGCAC
DROME$HSP27_05	564 (+)	0	100.00	FXR, RXR-alpha	TGCACT
RAT$OC_05	564 (+)	0	100.00	RXR-alpha, VDR	TGCACT
AS$mEMBP_04	566 (+)	0	100.00	EmBP-1a	CACTG
HS$GG_12	568 (+)	0	100.00	NF-E	CTGTC
DROME$EVE_10	568 (+)	0	100.00	GAGA factor	CTGTC
HS$D1A_01	568 (+)	0	100.00	Meis-2a, Meis-2b, Meis-2c, Meis-2d, TGIF	CTGTCA
AS$MEIS1_11	568 (+)	0	100.00	Meis-1a, Meis-1b	CTGTCA
AS$MEIS1_12	568 (+)	0	100.00	Meis-1a, Meis-1b	CTGTCA
HS$UPA_06	568 (-)	0	100.00	Pbx-1a, Pbx-1b, Pbx-2, PKNOX1, PKNOX2, UEF-3	TGACAG
AS$MEIS1_01	568 (-)	0	100.00	Meis-1a, Meis-1b	TGACAG
AS$MEIS1_03	568 (-)	0	100.00	Meis-1a, Meis-1b	TGACAG
AS$MEIS1_04	568 (-)	0	100.00	Meis-1a, Meis-1b	TGACAG
AS$MEIS1_05	568 (-)	0	100.00	Meis-1a, Meis-1b	TGACAG
AS$MEIS1_06	568 (-)	0	100.00	Meis-1a, Meis-1b	TGACAG
AS$MEIS1_07	568 (-)	0	100.00	Meis-1a, Meis-1b	TGACAG
AS$MEIS1_08	568 (-)	0	100.00	Meis-1a, Meis-1b	TGACAG
AS$MEIS1_14	568 (-)	0	100.00	Meis-1a, Meis-1b	TGACAG
AS$MEIS1_19	568 (-)	0	100.00	Meis-1a, Meis-1b	TGACAG
AS$MEIS1AHOXA9_01	568 (-)	0	100.00	HOXA9, Meis-1a	TGACAG
AS$MEIS1AHOXA9_02	568 (-)	0	100.00	HOXA9, Meis-1a	TGACAG
AS$MEIS1AHOXA9_03	568 (-)	0	100.00	HOXA9, Meis-1a	TGACAG
AS$MEIS1AHOXA9_04	568 (-)	0	100.00	HOXA9, Meis-1a	TGACAG
AS$MEIS1AHOXA9_05	568 (-)	0	100.00	HOXA9, Meis-1a	TGACAG
AS$MEIS1AHOXA9_06	568 (-)	0	100.00	HOXA9, Meis-1a	TGACAG
AS$MEIS1AHOXA9_09	568 (-)	0	100.00	HOXA9, Meis-1a	TGACAG
AS$MEIS1AHOXA9_10	568 (-)	0	100.00	HOXA9, Meis-1a	TGACAG
AS$MEIS1AHOXA9_13	568 (-)	0	100.00	HOXA9, Meis-1a	TGACAG
AS$MEIS1BHOXA9_01	568 (-)	0	100.00	HOXA9, Meis-1b	TGACAG
AS$MEIS1BHOXA9_02	568 (-)	0	100.00	HOXA9, Meis-1b	TGACAG
AS$MEIS1BHOXA9_03	568 (-)	0	100.00	HOXA9, Meis-1b	TGACAG

AS$MEIS1BHOXA9_03	568 (-)	0	100.00	HOXA9, Meis-1b	TGACAG
AS$MEIS1BHOXA9_04	568 (-)	0	100.00	HOXA9, Meis-1b	TGACAG
AS$MEIS1BHOXA9_05	568 (-)	0	100.00	HOXA9, Meis-1b	TGACAG
AS$MEIS1BHOXA9_06	568 (-)	0	100.00	HOXA9, Meis-1b	TGACAG
AS$MEIS1BHOXA9_07	568 (-)	0	100.00	HOXA9, Meis-1b	TGACAG
AS$TGIF_01	569 (+)	0	100.00	TGIF	TGTCA
AS$TGIF_02	569 (+)	0	100.00	TGIF	TGTCA
AS$TGIF_03	569 (+)	0	100.00	TGIF	TGTCA
AS$TGIF_04	569 (+)	0	100.00	TGIF	TGTCA
AS$TGIF_05	569 (+)	0	100.00	TGIF	TGTCA
AS$TGIF_06	569 (+)	0	100.00	TGIF	TGTCA
AS$TGIF_07	569 (+)	0	100.00	TGIF	TGTCA
AS$TGIF_08	569 (+)	0	100.00	TGIF	TGTCA
AS$TGIF_10	569 (+)	0	100.00	TGIF	TGTCA
AS$TGIF_11	569 (+)	0	100.00	TGIF	TGTCA
AS$TGIF_12	569 (+)	0	100.00	TGIF	TGTCA
AS$TGIF_13	569 (+)	0	100.00	TGIF	TGTCA
AS$TGIF_14	569 (+)	0	100.00	TGIF	TGTCA
AS$TGIF_15	569 (+)	0	100.00	TGIF	TGTCA
POT$PR10a_01	569 (+)	0	100.00	PBF-1, PBF-2 (p24)	TGTCA
POT$PR10a_01	569 (-)	0	100.00	PBF-1, PBF-2 (p24)	TGACA
AS$EN1_06	571 (-)	0	100.00	En-1	GTTATGA
AS$MTTFA_10	572 (-)	0	100.00	mtTFA	TTATG
AS$MTTFA_11	572 (-)	0	100.00	mtTFA	TTATG
MOUSE$RVL3_03	574 (+)	0	100.00	RXR-alpha	TAACT
HS$ADH2_09	576 (-)	0	100.00	GR	GAAAGT
HS$TNFA_04	578 (-)	0	100.00		AGAAA
I$HSF_01	578 (-)	0	90.00	HSF	AGAAN
F$HSF_01	578 (-)	0	90.00	HSF	AGAAN
AS$mEMBP_04	583 (+)	0	100.00	EmBP-1a	CACTG
AS$SRY_14	584 (-)	1	88.89	SRY	TCCTATCAGT
AS$MEIS1AHOXA9_07	585 (-)	0	100.00	HOXA9, Meis-1a	TAACAG
AS$ARR_01	590 (+)	0	100.00	ARR1, ARR2	AGGATT
DROME$KR_18	592 (+)	1	87.50	Tll	GATTCTTGC
Y$MAL61_03	593 (+)	0	100.00	MIG1	ATTTT
Y$SUC2_01	594 (+)	0	100.00	MIG1	TTTTT
AS$PF1_01	594 (+)	0	100.00	PF1	TTTTT
AS$PF1_02	594 (+)	0	100.00	PF1	TTTTT
CHICK$VIT2_14	595 (-)	0	100.00	C/EBPalpha	TTGCAAAA
SV$SV40_20	596 (-)	1	87.50	POU2F1	CTTTGCATA
SV$SV40_28	596 (-)	1	87.50	POU2F1	CTTTGCATA
AS$STAT5A_58	597 (+)	1	87.50	STAT5A	TTCCAAAGG
HS$CYCD1_10	598 (+)	0	100.00	TCF-4E	TGCAAAG
ESP$CONS_02	598 (+)	0	92.86		TGYAAAG
RICE$GLUB1_04	598 (+)	0	100.00		TGCAAAG
AS$VMYB_02	604 (-)	1	88.89	v-Myb	CCAAACTGCC
AS$AREB6_44	608 (+)	0	100.00	AREB6	GTTTC
AS$AREB6_46	608 (+)	0	100.00	AREB6	GTTTC
AS$AREB6_48	608 (+)	0	100.00	AREB6	GTTTC
AS$AREB6_49	608 (+)	0	100.00	AREB6	GTTTC
AS$AREB6_50	608 (+)	0	100.00	AREB6	GTTTC
AS$AREB6_52	608 (+)	0	100.00	AREB6	GTTTC
AS$AREB6_53	608 (+)	0	100.00	AREB6	GTTTC
AS$STAT5A_66	610 (+)	1	87.50	STAT5A	TTCGTGAAA
HS$RARB_01	613 (-)	0	100.00	CAR, CF1, RAR-alpha1, RAR-beta, RAR-beta2, RAR-gamma, RXR-alpha, RXR-beta, RXR-gamma, v-ErbA	GTTCAC
HS$ADH3_01	614 (+)	0	100.00	CAR, RAR-alpha1, RAR-beta, RXR-alpha	TGAAC
AS$MEIS1AHOXA9_12	614 (+)	0	100.00	HOXA9, Meis-1a	TGAACG
AS$RAR_05	614 (-)	0	100.00	RAR-alpha1	GTTCA
AS$RAR_06	614 (-)	0	100.00	RAR-alpha1	GTTCA
AS$TR_06	614 (-)	0	100.00	T3R-alpha	GTTCA
AS$TR_07	614 (-)	0	100.00	T3R-alpha	GTTCA
AS$TR_08	614 (-)	0	100.00	T3R-alpha	GTTCA
AS$TR_11	614 (-)	0	100.00	T3R-alpha	GTTCA
MOUSE$RARA2_01	614 (-)	0	100.00	RAR-alpha1, RAR-beta2, RAR-gamma1	GTTCA
AS$NCX_31	615 (-)	1	88.89	Ncx	CCGTACGTTC
AS$STAT5A_54	620 (+)	1	87.50	STAT5A	TACAGAGAA
RAT$POMC_03	622 (+)	0	100.00	GR	CAGAG
AS$MSX1_09	622 (-)	1	87.50	Msx-1	CTGTATCTG
AS$ARF1_01	624 (+)	0	100.00	ARF1, ARF5	GAGACA
PEA$IAA45_07	624 (+)	0	100.00	ARF1	GAGACA
YL$XPR2_01	624 (-)	0	100.00		GTCTC
AS$ARF1_01	624 (-)	0	100.00	ARF1, ARF5	TGTCTC
AS$ARF1_05	624 (-)	0	100.00	ARF1, ARF4, ARF5, ARF6, ARF7, ARF8	TGTCTC
AS$TGIF_09	625 (-)	0	100.00	TGIF	TGTCT
AS$MEIS1_10	625 (-)	0	100.00	Meis-1a, Meis-1b	CTGTCT
MOUSE$MCK_11	625 (-)	1	88.89	p53	GGGCCTGCCT
HS$GG_12	626 (-)	0	100.00	NF-E	CTGTC
DROME$EVE_10	626 (-)	0	100.00	GAGA factor	CTGTC
HS$APOB_11	633 (+)	0	100.00	LF-A1	CCTTGC
AMV$AMV_02	634 (+)	0	100.00	C/EBPalpha	CTTGC
RAV0$RAV0_01	634 (-)	0	100.00	C/EBPalpha	GCAAG
AS$UBX_54	637 (-)	1	90.91	Ubx	AGGGATAATTGC
AS$HOXA3_02	637 (-)	1	87.50	HOXA3	CATAATAGC
Y$ARS1_05	641 (+)	0	100.00	ABF2	TTATC
AS$MTTFA_01	641 (+)	0	100.00	mtTFA	TTATC
AS$MTTFA_02	641 (+)	0	100.00	mtTFA	TTATC
AS$MTTFA_03	641 (+)	0	100.00	mtTFA	TTATC
AS$MTTFA_04	641 (+)	0	100.00	mtTFA	TTATC
AS$MTTFA_05	641 (+)	0	100.00	mtTFA	TTATC
AS$MTTFA_06	641 (+)	0	100.00	mtTFA	TTATC
AS$MTTFA_07	641 (+)	0	100.00	mtTFA	TTATC
AS$MTTFA_08	641 (+)	0	100.00	mtTFA	TTATC
AS$MTTFA_09	641 (+)	0	100.00	mtTFA	TTATC
EBV$BHLF1_05	641 (-)	0	100.00	TBP	GATAA
Y$DAL3_01	641 (-)	0	100.00	DAL80	GATAA
HS$MSH2_01	648 (+)	0	100.00	HNF-3alpha, HNF-3B	TATTT
HS$BG_29	648 (-)	1	88.89	GATA-1	CTAAAGAATA
Y$MAL61_03	649 (+)	0	100.00	MIG1	ATTTT
RAT$AMHC_04	649 (-)	1	87.50	aMEF-2, MEF-2 (516 AA)	CTAAAAAAG
Y$SUC2_01	650 (+)	0	100.00	MIG1	TTTTT
AS$PF1_01	650 (+)	0	100.00	PF1	TTTTT
AS$PF1_02	650 (+)	0	100.00	PF1	TTTTT
MOUSE$ADA_06	650 (-)	0	100.00	TBP	TAAAAAA
Y$SUC2_01	651 (+)	0	100.00	MIG1	TTTTT
AS$PF1_01	651 (+)	0	100.00	PF1	TTTTT
AS$PF1_02	651 (+)	0	100.00	PF1	TTTTT
Y$MAL2R_01	652 (+)	0	100.00	MIG1	TTTTA
AS$FOXJ1_04	652 (+)	0	100.00	FOXJ1	TTTTA
AS$E2TBP_02	652 (-)	1	87.50	TBP	TATATAAAA
RAT$RPK_01	654 (+)	1	87.50	GATA-1	TCAGATAAG
NEUCR$NIT3_02	655 (+)	0	100.00	NIT2	TAGATA
ASPN$NIAD_01	655 (-)	0	100.00	NIT2	TATCTA
NIT2$CONS_01	655 (-)	0	91.67	NIT2	TATCTM
F$NIT2_01	655 (-)	0	91.67	NIT2	TATCTM
MOUSE$BMG_02	656 (+)	0	100.00	GATA-1	AGATA
AS$GATA1_01	656 (+)	0	100.00	GATA-1	AGATAA
MOUSE$PBGD_06	656 (+)	0	100.00	GATA-1	AGATAA
HS$AG_09	656 (+)	0	100.00	GATA-1	AGATAA
MOUSE$MCCPA_01	656 (+)	0	100.00	GATA-1, GATA-2	AGATAA
RAT$BNP_01	656 (+)	0	100.00	GATA-4	AGATAA
RAT$BNP_02	656 (+)	0	100.00	GATA-4	AGATAA
MOUSE$PDGFRA_01	656 (+)	0	100.00	GATA-4	AGATAA
HS$ET1_01	656 (-)	0	100.00	GATA-2	TTATCT
HS$GG_29	656 (-)	0	100.00	GATA-1	TTATCT
HS$AG_02	656 (-)	0	100.00	GATA-1	TTATCT
MOUSE$EPOR_01	656 (-)	0	100.00	GATA-1	TTATCT
HS$AG_14	656 (-)	0	100.00	GATA-1	TTATCT
HS$TCRBL_10	656 (-)	0	100.00	GATA-3	TTATCT
MOUSE$GSHPX1_06	656 (-)	0	100.00	GATA-1	TATCT
MOUSE$GSHPX1_07	656 (-)	0	100.00	GATA-1	TATCT
MOUSE$GSHPX1_09	656 (-)	0	100.00	GATA-1	TATCT
HS$BG_31	656 (-)	0	100.00	GATA-1	TTATCT
MOUSE$TCRBL_03	656 (-)	0	100.00	GATA-3	TTATCT
MOUSE$AAG_02	657 (+)	0	100.00		GATAAG
EBV$BHLF1_05	657 (+)	0	100.00	TBP	GATAA
Y$DAL3_01	657 (+)	0	100.00	DAL80	GATAA
RAT$AR_01	657 (+)	0	100.00	NF-kappaB	GATAAG
Y$ARS1_05	657 (-)	0	100.00	ABF2	TTATC
AS$MTTFA_01	657 (-)	0	100.00	mtTFA	TTATC
AS$MTTFA_02	657 (-)	0	100.00	mtTFA	TTATC
AS$MTTFA_03	657 (-)	0	100.00	mtTFA	TTATC
AS$MTTFA_04	657 (-)	0	100.00	mtTFA	TTATC
AS$MTTFA_05	657 (-)	0	100.00	mtTFA	TTATC
AS$MTTFA_06	657 (-)	0	100.00	mtTFA	TTATC
AS$MTTFA_07	657 (-)	0	100.00	mtTFA	TTATC
AS$MTTFA_08	657 (-)	0	100.00	mtTFA	TTATC
AS$MTTFA_09	657 (-)	0	100.00	mtTFA	TTATC
AS$ETS1_02	676 (+)	1	87.50	Ets-1 deltaVII, PU.1	GGAGGAAGT
TCF2ALPHA$CONS	677 (+)	0	87.50	TCF-2alpha	SAGGAAGY
MOUSE$GSHPX1_02	677 (+)	0	100.00	PU.1	GAGGAAG
AS$NCX_37	677 (+)	1	88.89	Ncx	GAGGATGTTT
MOUSE$KROX_01	677 (-)	0	100.00	Elk-1, SAP-1a, SAP-1b, SRF	TTCCTC
MOUSE$FCGR3A_02	677 (-)	0	100.00	PU.1	TTCCTC
PEA3$CONS	678 (+)	0	91.67	c-Ets-1 54, c-Ets-1 68, c-Ets-2 58-64, PEA3	AGGAAR
TIN1$CONS	678 (+)	1	87.50	TIN-1	AGGAAGTTC
DROME$E74_10	678 (+)	0	100.00	E74A	AGGAA
DROME$E74_11	678 (+)	0	100.00	E74A	AGGAA
E74A$CONS_01	678 (+)	0	90.00	E74A	MGGAA
MOUSE$RPL32_01	679 (+)	0	100.00	f(alpha)-f(epsilon), HrpF, XrpFI	GGAAG
HS$TNFA_02	679 (+)	0	100.00		GGAAG
HS$TNFA_03	679 (+)	0	100.00		GGAAG
XENLA$RPL14_01	679 (-)	0	100.00	HrpF, XrpFI	CTTCC
Y$TPI_01	679 (-)	0	100.00	GCR1	CTTCC
MOUSE$IL2RA_03	679 (-)	0	100.00	c-Ets-1, Elf-1	ACTTCC
MOUSE$CRISP3_01	682 (-)	0	100.00	AR	AAAACT
MOUSE$M1H2LD_04	683 (+)	1	88.89	ICSBP	GTTTCACTTC
HS$IL2_07	683 (-)	0	100.00	NF-IL-2A, POU2F1, POU2F2 (Oct-2.1)	GTAAAAC
Y$MAL2R_01	684 (+)	0	100.00	MIG1	TTTTA
AS$FOXJ1_04	684 (+)	0	100.00	FOXJ1	TTTTA
HS$TSHB_04	686 (-)	0	100.00	T3R-beta1	AAGTAA
RAT$BCAS_09	688 (+)	1	90.00	delta factor, STAT5	ACTTCTTGGAA
RAT$SPI23_02	688 (-)	1	87.50	C/EBPalpha	CCCAGAAAT
I$HSF_01	689 (-)	0	90.00	HSF	AGAAN
F$HSF_01	689 (-)	0	90.00	HSF	AGAAN
AS$STAT_01	690 (+)	1	87.50	STAT6	TTCCGGGAA
AS$STAT_02	690 (+)	1	87.50	STAT6	TTCCGGGAA
AS$STAT_12	690 (+)	1	87.50	STAT6	TTCTCGGAA
AS$STAT5A_37	690 (+)	1	87.50	STAT5A	TTCTGAGAA
AS$STAT5A_38	690 (+)	1	87.50	STAT5A	TTCCGGGAA
AS$STAT5A_44	690 (+)	1	87.50	STAT5A	TTCCGGGAA
HS$P21WAF1_10	690 (+)	1	87.50	STAT5B	TTCTGAGAA
HS$P21WAF1_11	690 (+)	1	87.50	STAT5B	TTCTTGGAA
STAT$CONS_01	690 (-)	0	88.89	STAT1, STAT1alpha, STAT1beta, STAT2, STAT3, STAT4, STAT5, STAT5A, STAT5B, STAT6	TTCCCRKAA
AS$STAT_06	690 (-)	0	100.00	STAT6	TTCCCAGAA
AS$STAT_09	690 (-)	1	87.50	STAT6	TTCTCAGAA
AS$STAT_10	690 (-)	1	87.50	STAT6	TTCCCGGAA
AS$STAT_11	690 (-)	1	87.50	STAT6	TTCCCCGAA
AS$STAT_14	690 (-)	0	100.00	STAT6	TTCCCAGAA
AS$STAT5A_35	690 (-)	1	87.50	STAT5A	TTCCGAGAA
AS$STAT5A_39	690 (-)	1	87.50	STAT5A	TTCCCGGAA
AS$STAT5A_41	690 (-)	0	100.00	STAT5A	TTCCCAGAA
AS$STAT5A_43	690 (-)	1	87.50	STAT5A	TTCCAAGAA
AS$STAT5A_46	690 (-)	1	87.50	STAT5A	TTCGCAGAA
AS$STAT5A_47	690 (-)	1	87.50	STAT5A	TTCCCCGAA
AS$STAT5A_48	690 (-)	1	87.50	STAT5A	TTCCTAGAA
AS$STAT5A_68	690 (-)	1	87.50	STAT5A	TACCCAGAA
HS$P21WAF1_09	690 (-)	1	87.50	STAT1, STAT3	TTCCCGGAA
V$STAT_01	690 (-)	0	88.89	STATx	TTCCCRKAA
SV$SV40_37	692 (+)	0	100.00		CTGGG
HAPF1$CONS	692 (+)	0	92.86	C/EBPbeta	CTGGRAA
RAT$AMGL_02	692 (+)	0	100.00	IL-6 RE-BP, STAT1, STAT5A, STAT5B, STAT6	CTGGGA
AS$IK_06	693 (+)	0	100.00	Ik-1, Ik-2	TGGGAA
HS$CDH1_01	693 (+)	0	100.00	LUN-1	TGGGA
HS$TLN_01	693 (+)	0	100.00	LUN-1	TGGGA
RABBIT$UG_11	693 (-)	0	100.00	PR B	TGTTCCCA
HS$CDH1_01	693 (-)	0	100.00	LUN-1	TCCCA
HS$TLN_01	693 (-)	0	100.00	LUN-1	TCCCA
HS$IL4_02	695 (+)	0	100.00		GGAAC
AS$PR_03	695 (-)	0	100.00	PR B	TGTTCC
AS$TR_07	695 (-)	0	100.00	T3R-alpha	GTTCC
AS$TR_10	695 (-)	0	100.00	T3R-alpha	GTTCC
MOMLV$MOMULV_01	696 (+)	0	100.00	LVa	GAACAG
MOMLV$MOMULV_02	696 (+)	0	100.00	LVa	GAACAG
MOUSE$MYOD_04	699 (-)	0	100.00	ARP-1, RXR-alpha, RXR-gamma, T3R-alpha	AGGCTG
Y$GAL1_10	700 (+)	0	100.00	GAL4	AGCCT
AS$PAX2_46	709 (+)	1	87.50	Pax-2.1, Pax-2.2	ACGAAACAT
AS$PAX2_51	709 (+)	0	100.00	Pax-2.1, Pax-2.2	ACGAAACCT
AS$PAX2_52	709 (+)	0	100.00	Pax-2.1, Pax-2.2	ACGAAACCT
AS$AREB6_44	711 (-)	0	100.00	AREB6	GTTTC
AS$AREB6_46	711 (-)	0	100.00	AREB6	GTTTC
AS$AREB6_48	711 (-)	0	100.00	AREB6	GTTTC
AS$AREB6_49	711 (-)	0	100.00	AREB6	GTTTC
AS$AREB6_50	711 (-)	0	100.00	AREB6	GTTTC
AS$AREB6_52	711 (-)	0	100.00	AREB6	GTTTC
AS$AREB6_53	711 (-)	0	100.00	AREB6	GTTTC
MOUSE$TRP1_03	718 (-)	0	100.00	Tbx2	GTGTGA
MOUSE$TRP1_04	718 (-)	0	100.00	Tbx2	GTGTGA
PV$PHSB_02	719 (+)	0	100.00	ROM1, ROM2	CACACGTCA
AD5$E1A_03	720 (-)	0	100.00	CREB	TGACGTGT
BZIP911$CONS_02	720 (-)	0	100.00	bZIP911	TGACGTGT
AS$mEMBP_15	721 (+)	0	100.00	EmBP-1a	CACGT
BZIP910$CONS_01	721 (-)	0	100.00	bZIP910	TGACGTG
RICE$EM_01	721 (-)	0	100.00	OSBZ8, TRAB1	ACGTG
RICE$EM_02	721 (-)	0	100.00	OSBZ8	ACGTG
HS$ENO1_01	721 (-)	0	100.00	HIF-1	GACGTG
AT$GST6_02	721 (-)	0	100.00	OBF4	GACGTG
AD$E4_16	722 (+)	0	100.00	ATF, atf1, ATF3, c-Jun, CRE-BP1, CREB, deltaCREB, EivF, TREB-1	ACGTCA
RAT$TH_02	722 (+)	0	100.00	ATF	ACGTCA
TDNA$NOS_02	722 (+)	0	100.00	HBP-1, HBP-1a, HBP-1b	ACGTCA
RAT$TH2B_04	722 (+)	0	100.00		ACGTCA
RICE$NR1_01	722 (+)	0	100.00	HBP-1a, HBP-1b	ACGTCA
Y$LPD1_02	722 (+)	0	100.00	ATF	ACGTCA
HS$GHA_03	722 (-)	0	100.00	GR, GR-alpha, GR-beta	TGACGT
HS$VIP_04	722 (-)	0	100.00	ATF	TGACGT
CAMV$35SR_03	722 (-)	0	100.00	HBP-1, HBP-1a, HBP-1a(1), HBP-1a(c14), HBP-1b, HBP-1b(c1)	TGACGT
RAT$TH2A_01	722 (-)	0	100.00		TGACGT
AT$DBP_01	722 (-)	0	100.00	GBF1, HBP-1a, HBP-1b, OBF3.1, OBF3.2, OBF4, OBF5, TGA1, TGA3, TGA6	TGACGT
PEA$LEGA_01	722 (-)	0	100.00	HBP-1a, HBP-1b	TGACGT
HT1$HTLV1_20	722 (-)	0	100.00	TAF-I, TAF-II, Tax	TGACGT
BZIP910$CONS_02	722 (-)	0	100.00	bZIP910	TGACGT
TDNA$NOS_01	723 (+)	0	100.00	ASF-1, OBF3.1, TGA1a, TGA1b	CGTCAG
HS$VIP_01	723 (+)	0	100.00	c-Fos, c-Jun, CRE-BP1, CREB	CGTCA
CAMV$35SR_01	723 (-)	0	100.00	ASF-1, OBF4, OBF5, SARP, TGA1, TGA1a, TGA1b, TGA2, TGA2.1, TGA2.2, TGA3, TGA6, ZAP1	TGACG
PIG$UPA_02	723 (-)	0	100.00	CREB	TGACG
PIG$UPA_03	723 (-)	0	100.00	CREB, CREBbeta	TGACG
HS$INS_04	723 (-)	0	100.00	CREB	TGACG
HS$PL_12	723 (-)	0	100.00		TGACG
RAT$EAI_09	724 (+)	0	100.00		GTCAG
HIV1$HIV1_02	724 (-)	0	100.00	AP-1	CACTGAC
AS$mEMBP_04	726 (-)	0	100.00	EmBP-1a	CACTG
MAIZE$C1_05	728 (+)	0	100.00		GTGTC
AS$TGIF_09	729 (+)	0	100.00	TGIF	TGTCT
AS$PAI_05	730 (-)	0	100.00		CAAAAGAC
CHICK$LYS_19	734 (+)	1	87.50	AP-3, C/EBPalpha	TTTGGAAAT
HS$CRH_01	736 (+)	0	100.00	GR	TGGACA
HS$GMCSF_03	740 (+)	0	100.00	YY1	CATTT
Y$MAL61_03	741 (+)	0	100.00	MIG1	ATTTT
Y$GAL4_01	742 (+)	0	100.00	MIG1	TTTTC
HS$TNFA_04	743 (-)	0	100.00		AGAAA
I$HSF_01	743 (-)	0	90.00	HSF	AGAAN
F$HSF_01	743 (-)	0	90.00	HSF	AGAAN
AS$STAT_04	744 (+)	1	87.50	STAT6	TTCCCGTCA
AS$STAT5A_44	744 (+)	1	87.50	STAT5A	TTCTCGACA
Y$HIS3_01	747 (-)	0	100.00	GCN4	TGACGA
HT1$HTLV1_20	747 (-)	0	100.00	TAF-I, TAF-II, Tax	TGACGA
RAT$MAD7_03	747 (-)	0	100.00		TGACGA
TDNA$NOS_01	748 (+)	0	100.00	ASF-1, OBF3.1, TGA1a, TGA1b	CGTCAG
HS$VIP_01	748 (+)	0	100.00	c-Fos, c-Jun, CRE-BP1, CREB	CGTCA
CAMV$35SR_01	748 (-)	0	100.00	ASF-1, OBF4, OBF5, SARP, TGA1, TGA1a, TGA1b, TGA2, TGA2.1, TGA2.2, TGA3, TGA6, ZAP1	TGACG
PIG$UPA_02	748 (-)	0	100.00	CREB	TGACG
PIG$UPA_03	748 (-)	0	100.00	CREB, CREBbeta	TGACG
HS$INS_04	748 (-)	0	100.00	CREB	TGACG
HS$PL_12	748 (-)	0	100.00		TGACG
RAT$EAI_09	749 (+)	0	100.00		GTCAG
AS$MSX1_12	751 (+)	1	87.50	Msx-1	CAGTACATC
AS$FTZ_55	752 (+)	0	100.00	Ftz	AGTACA
MOUSE$TDT_03	760 (-)	1	87.50	LyF-1	TTCAGCAGG
AS$STAT5A_47	760 (-)	1	87.50	STAT5A	TTCGACAGG
AS$STAT5A_48	760 (-)	1	87.50	STAT5A	TTCGACAGG
SV$SV40_46	761 (-)	1	90.00	TEF-1	ACATTCCACAG
HS$CDC2_10	764 (+)	0	100.00		TTGAA
HS$CYCA_06	764 (+)	0	100.00		TTGAA
HS$CDC25C_05	764 (+)	0	100.00		TTGAA
Y$TY1_03	765 (-)	0	100.00	GCN4	ACATTCA
MMTV$MMTV_02	768 (-)	0	100.00	GR	AGAACAT
MMTV$MMTV_03	769 (+)	0	100.00	AR, GR	TGTTCT
MMTV$MMTV_05	769 (+)	0	100.00	GR	TGTTCT
MMTV$MMTV_06	769 (+)	0	100.00	GR, PR B	TGTTCT
MMTV$MMTV_08	769 (+)	0	100.00	GR, PR B	TGTTCT
MMTV$MMTV_09	769 (+)	0	100.00	GR, PR B	TGTTCT
MMTV$MMTV_10	769 (+)	0	100.00	GR, PR B	TGTTCT
MMTV$MMTV_12	769 (+)	0	100.00	PR-alpha, PR-beta	TGTTCT
MMTV$MMTV_14	769 (+)	0	100.00	PR B	TGTTCT
MMTV$MMTV_19	769 (+)	0	100.00	GR, PR B	TGTTCT
MMTV$MMTV_22	769 (+)	0	100.00	PR B, PR-alpha, PR-beta	TGTTCT
MMTV$MMTV_23	769 (+)	0	100.00	GR	TGTTCT
MMTV$MMTV_24	769 (+)	0	100.00	PR-alpha, PR-beta	TGTTCT
MOUSE$RAS1_01	769 (+)	0	100.00	GR	TGTTCT
RAT$TAT_02	769 (+)	0	100.00	GR, GR-alpha, GR-beta, PR-alpha, PR-beta	TGTTCT
RABBIT$UG_02	769 (+)	0	100.00	GR	TGTTCT
RABBIT$UG_04	769 (+)	0	100.00	GR	TGTTCT
RABBIT$UG_05	769 (+)	0	100.00	GR	TGTTCT
CHICK$PR_03	769 (+)	0	100.00	PR-alpha, PR-beta	TGTTCT
AS$GR_02	769 (+)	0	100.00	GR-alpha, GR-beta	TGTTCT
AS$GR_03	769 (+)	0	100.00	GR-alpha, GR-beta	TGTTCT
AS$GR_04	769 (+)	0	100.00	GR, GR-alpha, GR-beta	TGTTCT
AS$GR_05	769 (+)	0	100.00	GR	TGTTCT
AS$GR_07	769 (+)	0	100.00	GR	TGTTCT
AS$GR_08	769 (+)	0	100.00	GR	TGTTCT
AS$GR_09	769 (+)	0	100.00	GR	TGTTCT
AS$GR_10	769 (+)	0	100.00	GR	TGTTCT
AS$GR_11	769 (+)	0	100.00	GR-alpha, GR-beta	TGTTCT
RAT$ANF_02	769 (+)	0	100.00	GR	TGTTCT
MOUSE$CRISP1_01	769 (+)	0	100.00	AR	TGTTCT
MOUSE$CRISP1_02	769 (+)	0	100.00	AR	TGTTCT
MOUSE$CRISP3_01	769 (+)	0	100.00	AR	TGTTCT
RAT$SGK_02	769 (+)	0	100.00	GR	TGTTCT
CHICK$LYS_01	769 (-)	0	100.00	GR, GR-alpha, GR-beta, PR, PR A	AGAACA
AS$GR_02	769 (-)	0	100.00	GR-alpha, GR-beta	AGAACA
AS$GR_03	769 (-)	0	100.00	GR-alpha, GR-beta	AGAACA
AS$GR_04	769 (-)	0	100.00	GR, GR-alpha, GR-beta	AGAACA
HIV1$HIV1_22	769 (-)	0	100.00	GR	AGAACA
AS$GR_05	769 (-)	0	100.00	GR	AGAACA
AS$GR_11	769 (-)	0	100.00	GR-alpha, GR-beta	AGAACA
RAT$GSTYA2_01	769 (-)	0	100.00	GR	AGAACA
I$HSF_01	770 (-)	0	90.00	HSF	AGAAN
F$HSF_01	770 (-)	0	90.00	HSF	AGAAN
CHICK$BAG_05	775 (-)	1	88.89	CACCC-binding factor	CCCCACCCTG
HS$TERT_01	776 (-)	0	100.00	c-Myc	CACCGT
BPV1$BPV1_24	778 (+)	0	100.00	Sp1	GGTGG
RAT$NEU_03	778 (+)	1	88.89		GGTGGGGGGG
HS$BG_43	778 (+)	0	100.00	CAC-binding protein	GGTGGGG
HS$BG_48	778 (+)	0	100.00	CAC-binding protein	GGTGG
MOUSE$CEBPA_06	778 (+)	0	100.00	Sp1	GGTGG
HS$GPB_02	778 (+)	0	100.00	Sp1	GGTGGG
V$MZF1_01	778 (+)	0	87.50	MZF1	NGNGGGGA
HT1$HTLV1_08	778 (-)	0	100.00	c-Ets-1 68	CCACC
HS$BG_52	778 (-)	0	100.00		CCACC
HS$BG_14	778 (-)	0	100.00		CCACC
HS$BG_44	778 (-)	0	100.00	CAC-binding protein	CCACC
Y$MEL1_02	779 (+)	0	100.00	MIG1	GTGGGG
Y$MAL61_03	779 (+)	0	100.00	MIG1	GTGGGG
Y$MAL61_04	779 (+)	0	100.00	MIG1	GTGGGG
MZF1$CONS_01	779 (+)	0	92.86	MZF-1	GNGGGGA
HS$LCK_02	779 (-)	1	87.50	LyF-1	CCTCCCAAC
MOUSE$M1H2KB_02	780 (+)	0	100.00	AP-2alpha, AP-2alphaA, AP-2alphaB	TGGGGA
MOUSE$M1H2KB_07	780 (+)	0	100.00	MBP-1 (1), NF-kappaB1, NF-kappaB2, NF-kappaB2 precursor	TGGGGA
MOUSE$M1H2KB_07	780 (-)	0	100.00	MBP-1 (1), NF-kappaB1, NF-kappaB2, NF-kappaB2 precursor	TCCCCA
AS$ZIC_07	781 (+)	1	87.50	Zic1	GGGGGGGTA
AS$ZIC_17	781 (+)	1	87.50	Zic1	GGGGGGGTA
AS$ZIC2_21	781 (+)	1	87.50	Zic2	GGGGGGGTA
AS$ZIC2_23	781 (+)	1	87.50	Zic2	GGGGAGATA
AS$ZIC3_21	781 (+)	1	87.50	Zic3	GGGGGGGTA
HT1$HTLV1_08	783 (-)	0	100.00	c-Ets-1 68	CCTCC
AS$FTZ_53	784 (+)	0	100.00	Ftz	GAGGTA
WHEAT$EM_02	786 (-)	1	87.50	Vp1	ACACGTGCC
RICE$RAB16A_03	788 (+)	0	100.00	OSBZ8, TRAB1	TACGTG
HS$VEGF_01	788 (+)	0	100.00	HIF-1	TACGTG
RICE$EM_01	789 (+)	0	100.00	OSBZ8, TRAB1	ACGTG
RICE$EM_02	789 (+)	0	100.00	OSBZ8	ACGTG
AS$mEMBP_15	789 (-)	0	100.00	EmBP-1a	CACGT
HS$PL_02	791 (-)	0	100.00	POU1F1a	TAAACAC
MOUSE$CDX2_01	792 (+)	0	100.00	FOXM1a, FOXM1b, HNF-3alpha, HNF-3B	TGTTT
RAT$FABPI_04	792 (+)	0	100.00	FOXM1a, FOXM1b, HNF-3alpha, HNF-3B	TGTTT
HS$ALP_02	792 (+)	0	100.00	HNF-3alpha, HNF-3B	TGTTT
MOUSE$ECADH_03	792 (-)	0	100.00	FOXM1a, FOXM1b, HNF-3alpha, HNF-3B	AAACA
AS$FREAC2_01	793 (-)	0	100.00	FOXF2	TAAAC
MOUSE$WAP_06	794 (+)	0	100.00		TTTAAA
Y$MAL63_01	794 (+)	0	100.00	MIG1	TTTAA
MOUSE$WAP_06	794 (-)	0	100.00		TTTAAA
Y$HO_08	794 (-)	1	87.50	PHO2	CAATTTAAA
Y$MAL63_01	795 (-)	0	100.00	MIG1	TTTAA
RAT$PL_15	796 (+)	0	100.00	F2F, POU1F1a	TTAAAT
Y$MAL2R_01	796 (-)	0	100.00	MIG1	TTTTA
AS$FOXJ1_04	796 (-)	0	100.00	FOXJ1	TTTTA
Y$MAL61_03	797 (-)	0	100.00	MIG1	ATTTT
HS$GMCSF_03	798 (-)	0	100.00	YY1	CATTT
V$MZF1_01	801 (+)	0	87.50	MZF1	NGNGGGGA
HS$TCRA_08	801 (-)	0	100.00	c-Ets-1, LEF-1, PEBP2alphaA1	CCGCA
AS$STAT_11	801 (-)	1	87.50	STAT6	TTCCCCGAA
AS$STAT5A_47	801 (-)	1	87.50	STAT5A	TTCCCCGAA
MZF1$CONS_01	802 (+)	0	92.86	MZF-1	GNGGGGA
Y$SUC2_01	802 (-)	0	100.00	MIG1	CCCCGC
Y$PDC1_02	802 (-)	0	100.00	MIG1	CCCCGC
HS$CDC2_06	805 (+)	0	100.00	c-Ets-2	GGGAAG
MOUSE$RPL32_01	806 (+)	0	100.00	f(alpha)-f(epsilon), HrpF, XrpFI	GGAAG
HS$TNFA_02	806 (+)	0	100.00		GGAAG
HS$TNFA_03	806 (+)	0	100.00		GGAAG
MAIZE$BZ1_03	806 (+)	1	87.50	B-Peru	GGCAGGTGC
XENLA$RPL14_01	806 (-)	0	100.00	HrpF, XrpFI	CTTCC
Y$TPI_01	806 (-)	0	100.00	GCR1	CTTCC
AS$ZIC2_25	808 (+)	1	87.50	Zic2	ACGGTGCTT
AS$ERR1_10	809 (+)	0	100.00	ERR1	AGGTGC
RAT$A1I3_04	811 (+)	0	100.00		GTGCT
RAT$A1I3_04	811 (-)	0	100.00		AGCAC
AS$PAX2_67	813 (-)	1	87.50	Pax-2.1, Pax-2.2	AATAAATGC
AS$TBP_12	814 (-)	1	90.00	TBP	TGATATAAAAG
Y$MAL2R_01	815 (+)	0	100.00	MIG1	TTTTA
AS$FOXJ1_04	815 (+)	0	100.00	FOXJ1	TTTTA
Y$PDC1_02	816 (+)	0	100.00	MIG1	TTTAT
Y$HAP4_01	816 (+)	0	100.00	MIG1	TTTAT
RAT$PL_07	816 (-)	0	100.00		AAATAAA
HS$EG_08	816 (-)	0	100.00		ATAAA
HS$GFAP_01	816 (-)	0	100.00	TFIID	ATAAA
HS$GFAP_02	816 (-)	0	100.00	TBP	ATAAA
AS$TBP_03	816 (-)	0	100.00	TBP	AAATAAA
Y$GAL1_15	817 (+)	0	100.00	MIG1	TTATTTC
MOUSE$HOXA5_04	817 (-)	0	100.00		AAATAA
MOUSE$HOXA5_05	817 (-)	0	100.00		AAATAA
MOUSE$HOXA5_06	817 (-)	0	100.00		AAATAA
HS$MSH2_01	818 (+)	0	100.00	HNF-3alpha, HNF-3B	TATTT
AS$CF1_31	820 (-)	1	87.50	CF1	GGGGTGACA
AS$APOA2_06	822 (+)	0	100.00		TCACC
MOUSE$THY1_05	822 (+)	1	87.50		CCACCCCTG
MOUSE$RVL3_03	822 (+)	0	100.00	RXR-alpha	TCACCC
RAT$OC_01	822 (-)	0	100.00	RXR-alpha, VDR	GGGTGA
AS$RAR_13	822 (-)	0	100.00	RAR-alpha1, RXR-alpha	GGGTGA
HS$IBABP_01	822 (-)	0	100.00	FXR, RXR-alpha	GGGTGA
RAT$PTHRP_01	822 (-)	0	100.00	RXR-alpha, VDR	GGGTGA
HS$OC_03	822 (-)	0	100.00	VDR	GGGTGA
AS$VDR_02	822 (-)	0	100.00	RXR-alpha, VDR	GGGTGA
HS$BG_01	823 (+)	0	100.00	CACCC-binding factor	CACCC
HS$GG_13	823 (+)	0	100.00	gammaCAC1, gammaCAC2	CACCC
HS$GG_14	823 (+)	0	100.00	CACCC-binding factor, Sp1	CACCC
HS$GP2B_01	823 (+)	0	100.00	CACCC-binding factor	CACCC
RAT$TOA_02	823 (+)	0	100.00	CACCC-binding factor	CACCC
HS$EG_06	823 (+)	0	100.00	Sp1	CACCC
AS$CACCC_01	823 (+)	0	100.00	CACCC-binding factor	CACCC
HS$AG_15	823 (-)	0	100.00	CAC-binding protein	GGGGTG
AS$VDR_04	825 (-)	0	100.00	VDR	AGGGG
Y$GLK1_02	825 (-)	0	100.00	MSN2, MSN4	AGGGG
Y$GSY2_01	825 (-)	0	100.00		CCAGGGG
MOUSE$TDT_04	826 (-)	1	88.89		GTCACCAGGG
MOUSE$TDT_03	827 (-)	1	87.50	LyF-1	TTCAGCAGG
AS$AREB6_03	827 (-)	1	87.50	AREB6	TTCACCTGG
AS$APOA2_06	830 (-)	0	100.00		TCACC
HS$M1B7_02	831 (-)	1	87.50		GAGTTTCAC
HS$IFI616_01	832 (+)	0	100.00	ISGF-3	TGAAACT
AS$VMYB_19	832 (+)	1	88.89	v-Myb	TGAAACTTGG
MOUSE$IL5_03	832 (-)	0	100.00	AP-1	AGTTTCA
AS$AREB6_44	833 (-)	0	100.00	AREB6	GTTTC
AS$AREB6_46	833 (-)	0	100.00	AREB6	GTTTC
AS$AREB6_48	833 (-)	0	100.00	AREB6	GTTTC
AS$AREB6_49	833 (-)	0	100.00	AREB6	GTTTC
AS$AREB6_50	833 (-)	0	100.00	AREB6	GTTTC
AS$AREB6_52	833 (-)	0	100.00	AREB6	GTTTC
AS$AREB6_53	833 (-)	0	100.00	AREB6	GTTTC
AS$ZIC3_20	838 (+)	1	87.50	Zic3	TGGGGGAGC
AS$VDR_04	839 (+)	0	100.00	VDR	AGGGG
Y$GLK1_02	839 (+)	0	100.00	MSN2, MSN4	AGGGG
MOUSE$AQP7_01	839 (+)	0	100.00	PPAR-alpha, PPAR-gamma, RXR-alpha	AGGGGA
LP$LPS1B_01	839 (-)	1	87.50		AGCCCCCCT
AS$ZIC2_23	840 (+)	1	87.50	Zic2	GGGGAGATA
RAT$MLC_04	842 (+)	0	100.00	MAPF2, YY1	GGAGC
HS$FAC_01	843 (+)	1	88.89	p53	GAGCTATTTT
HS$GG_22	846 (+)	0	100.00		CTAAT
RAT$OPSIN_01	846 (+)	0	100.00		CTAAT
XENLA$BMP4_01	846 (+)	0	100.00	Xvent-2	CTAATT
AS$XVENT2_01	846 (+)	0	100.00	Xvent-2	CTAATT
DROME$EVE_29	847 (+)	1	88.89	Hb	TTATTTTTTT
Y$SUC2_02	847 (-)	0	100.00	MIG1	AATTA
RAT$DBH_01	847 (-)	0	100.00	ARIX, c-Fos, c-Jun, CREB, CREMtau	AATTA
Y$MAL61_03	849 (+)	0	100.00	MIG1	ATTTT
Y$SUC2_01	850 (+)	0	100.00	MIG1	TTTTT
AS$PF1_01	850 (+)	0	100.00	PF1	TTTTT
AS$PF1_02	850 (+)	0	100.00	PF1	TTTTT
Y$SUC2_01	851 (+)	0	100.00	MIG1	TTTTT
AS$PF1_01	851 (+)	0	100.00	PF1	TTTTT
AS$PF1_02	851 (+)	0	100.00	PF1	TTTTT
MOUSE$ADA_06	851 (+)	0	100.00	TBP	TAAAAAA
Y$SUC2_01	852 (+)	0	100.00	MIG1	TTTTT
AS$PF1_01	852 (+)	0	100.00	PF1	TTTTT
AS$PF1_02	852 (+)	0	100.00	PF1	TTTTT
Y$MAL2R_01	853 (+)	0	100.00	MIG1	TTTTA
AS$FOXJ1_04	853 (+)	0	100.00	FOXJ1	TTTTA
MOUSE$TTPA_02	854 (+)	1	88.89	C/EBPalpha, HNF-3alpha, HNF-3beta	TTCAAACATG
MOUSE$WAP_06	854 (+)	0	100.00		TTTAAA
Y$MAL63_01	854 (+)	0	100.00	MIG1	TTTAA
MOUSE$WAP_06	854 (-)	0	100.00		TTTAAA
Y$MAL63_01	855 (-)	0	100.00	MIG1	TTTAA
AS$FREAC2_01	856 (+)	0	100.00	FOXF2	TAAAC
MOUSE$ECADH_03	857 (+)	0	100.00	FOXM1a, FOXM1b, HNF-3alpha, HNF-3B	AAACA
MOUSE$CDX2_01	857 (-)	0	100.00	FOXM1a, FOXM1b, HNF-3alpha, HNF-3B	TGTTT
RAT$FABPI_04	857 (-)	0	100.00	FOXM1a, FOXM1b, HNF-3alpha, HNF-3B	TGTTT
HS$ALP_02	857 (-)	0	100.00	HNF-3alpha, HNF-3B	TGTTT
MOUSE$IGH_55	861 (-)	0	100.00		ATCAT
MOUSE$BMG_01	862 (+)	0	100.00	GATA-1	TGATT
AS$PAX2_45	862 (-)	1	87.50	Pax-2.1, Pax-2.2	TAAAAATCA
AS$PAX2_68	862 (-)	1	87.50	Pax-2.1, Pax-2.2	CCAAAATCA
Y$MAL61_03	864 (+)	0	100.00	MIG1	ATTTT
Y$SUC2_01	865 (+)	0	100.00	MIG1	TTTTT
AS$PF1_01	865 (+)	0	100.00	PF1	TTTTT
AS$PF1_02	865 (+)	0	100.00	PF1	TTTTT
MOUSE$AP2_07	868 (+)	0	100.00	NF-1	TTGGC
HS$GRH_01	869 (+)	0	100.00	AP-2, AP-2alphaA, AP-2alphaB, NF-1	TGGCC
HS$GRH_01	869 (-)	0	100.00	AP-2, AP-2alphaA, AP-2alphaB, NF-1	GGCCA
RAT$TAT_15	869 (-)	0	100.00	HNF-3alpha	GGCCA
HS$GG_36	870 (-)	0	100.00	Sp1	GGGGCC
AS$ZIC3_22	870 (-)	1	87.50	Zic3	AGGGGGGCC
PA$PY_26	871 (+)	0	100.00	T-Ag	GCCCC
MAIZE$ADH11S_06	871 (+)	0	100.00	GCBP-1, Sp1	GCCCC
PA$PY_23	871 (-)	0	100.00	T-Ag	GGGGC
SV$SV40_04	871 (-)	0	100.00	T-Ag	GGGGC
SV$SV40_63	871 (-)	0	100.00	T-Ag	GGGGC
HS$CYCD1_14	872 (+)	0	100.00	Sp1, Sp2, Sp3, Sp4	CCCCC
HS$CYCD1_15	872 (+)	0	100.00	Sp1, Sp2, Sp3, Sp4	CCCCC
AS$VDR_03	872 (-)	0	100.00	VDR	AGGGGG
AS$VDR_05	872 (-)	0	100.00	VDR	AGGGGG
AS$VDR_07	872 (-)	0	100.00	VDR	AGGGGG
AS$VDR_09	872 (-)	0	100.00	VDR	AGGGGG
AS$VDR_04	873 (-)	0	100.00	VDR	AGGGG
Y$GLK1_02	873 (-)	0	100.00	MSN2, MSN4	AGGGG
HS$CDC2_10	877 (+)	0	100.00		TTGAA
HS$CYCA_06	877 (+)	0	100.00		TTGAA
HS$CDC25C_05	877 (+)	0	100.00		TTGAA
HS$ADH3_01	878 (+)	0	100.00	CAR, RAR-alpha1, RAR-beta, RXR-alpha	TGAAC
RAT$3KAT_01	878 (+)	0	100.00	PPAR-alpha, RXR-alpha	TGAACC
RAT$CYP24_02	878 (+)	0	100.00	VDR	TGAACC
AS$RAR_01	878 (-)	0	100.00	RAR-gamma, RXR-alpha	GGTTCA
AS$RAR_05	878 (-)	0	100.00	RAR-alpha1	GTTCA
AS$RAR_06	878 (-)	0	100.00	RAR-alpha1	GTTCA
AS$TR_06	878 (-)	0	100.00	T3R-alpha	GTTCA
AS$TR_07	878 (-)	0	100.00	T3R-alpha	GTTCA
AS$TR_08	878 (-)	0	100.00	T3R-alpha	GTTCA
AS$TR_11	878 (-)	0	100.00	T3R-alpha	GTTCA
MOUSE$RARA2_01	878 (-)	0	100.00	RAR-alpha1, RAR-beta2, RAR-gamma1	GTTCA
MOUSE$RARB_01	878 (-)	0	100.00	RAR-beta, RXR-alpha, RXR-beta	GGTTCA
DROME$HSP27_05	878 (-)	0	100.00	FXR, RXR-alpha	GGTTCA
MOUSE$SPP1_01	878 (-)	0	100.00	RXR-alpha, VDR	GGTTCA
HS$P21WAF1_05	878 (-)	0	100.00	RXR-alpha, VDR	GGTTCA
HS$CABD9_01	878 (-)	0	100.00	RXR-alpha, VDR	GGTTCA
AS$SXR_02	878 (-)	0	100.00	RXR-alpha, SXR	GGTTCA
AS$SXR_03	878 (-)	0	100.00	RXR-alpha, SXR	GGTTCA
AS$SXR_04	878 (-)	0	100.00	RXR-alpha, SXR	GGTTCA
Y$ILV2_02	880 (+)	1	87.50	repressor of CAR1 expression	AGCCGCCGG
HS$CDC25C_04	882 (-)	0	100.00		GGCGG
AS$RAR_14	886 (-)	0	100.00	RAR-alpha1, RXR-alpha	AGGCCG
CHICK$CA2_02	891 (+)	0	100.00	RXR-alpha, VDR	TGGACT
RAT$PEPCK_17	891 (-)	0	100.00	GR	AGTCCA
AS$TR_34	891 (-)	0	100.00	T3R-alpha1, T3R-alpha2	AGTCCA
MOUSE$TS_04	898 (+)	1	87.50		AGTTTCCCA
AS$AREB6_44	899 (+)	0	100.00	AREB6	GTTTC
AS$AREB6_46	899 (+)	0	100.00	AREB6	GTTTC
AS$AREB6_48	899 (+)	0	100.00	AREB6	GTTTC
AS$AREB6_49	899 (+)	0	100.00	AREB6	GTTTC
AS$AREB6_50	899 (+)	0	100.00	AREB6	GTTTC
AS$AREB6_52	899 (+)	0	100.00	AREB6	GTTTC
AS$AREB6_53	899 (+)	0	100.00	AREB6	GTTTC
HS$IFI616_01	900 (-)	0	100.00	ISGF-3	GGAAA
MOUSE$IL5_02	900 (-)	0	100.00	NF-ATc, NF-ATp, NF-ATx	GGAAA
HS$IL4_01	900 (-)	0	100.00		GGAAA
MOUSE$IL4_06	900 (-)	0	100.00		GGAAA
MOUSE$IL4_07	900 (-)	0	100.00		GGAAA
MOUSE$IL4_08	900 (-)	0	100.00		GGAAA
HS$IL5_02	900 (-)	0	100.00		GGAAA
HS$IL13_01	900 (-)	0	100.00		GGAAA
MOUSE$CD40L_01	900 (-)	0	100.00		GGAAA
MOUSE$CD40L_02	900 (-)	0	100.00		GGAAA
MOUSE$CD40L_03	900 (-)	0	100.00		GGAAA
MOUSE$CD40L_04	900 (-)	0	100.00		GGAAA
HS$CD40L_01	900 (-)	0	100.00		GGAAA
HS$CD40L_02	900 (-)	0	100.00		GGAAA
HS$IFNG_03	900 (-)	0	100.00		GGAAA
HS$IFNG_04	900 (-)	0	100.00		GGAAA
HS$TNFA_05	900 (-)	0	100.00		GGAAA
MOUSE$MCP3_01	900 (-)	0	100.00		GGAAA
HS$TNFSF6_01	900 (-)	0	100.00		GGAAA
AS$STAT_06	901 (+)	1	87.50	STAT6	TTCCCAGAA
AS$STAT_14	901 (+)	1	87.50	STAT6	TTCCCAGAA
AS$STAT5A_41	901 (+)	1	87.50	STAT5A	TTCCCAGAA
HAPF1$CONS	901 (-)	0	92.86	C/EBPbeta	CTGGRAA
AS$IK_06	901 (-)	0	100.00	Ik-1, Ik-2	TGGGAA
HS$CDH1_01	902 (+)	0	100.00	LUN-1	TCCCA
HS$TLN_01	902 (+)	0	100.00	LUN-1	TCCCA
RAT$AMGL_02	902 (-)	0	100.00	IL-6 RE-BP, STAT1, STAT5A, STAT5B, STAT6	CTGGGA
HS$CDH1_01	902 (-)	0	100.00	LUN-1	TGGGA
HS$TLN_01	902 (-)	0	100.00	LUN-1	TGGGA
SV$SV40_37	903 (-)	0	100.00		CTGGG
CHICK$STN1_01	904 (+)	0	100.00	LBP-1, MyoD	CCAGC
CHICK$STN1_03	904 (+)	0	100.00	LBP-1, NF-1 (-like proteins)	CCAGC
MOUSE$M2EAK_03	905 (+)	0	100.00	NF-Y	CAGCA
DROME$ADH_14	907 (+)	0	100.00	Adf-1	GCAGC
DROME$ADH_06	907 (-)	0	100.00	Adf-1	GCTGC
HS$CMYC_16	907 (-)	0	100.00	CTCF	GCTGC
HS$CYCD1_15	910 (+)	0	100.00	Sp1, Sp2, Sp3, Sp4	GCCCG
XENLA$TFIIIA_02	911 (+)	1	87.50	USF1	CACGTGCTC
RAT$LPK_01	911 (+)	0	100.00	USF-1, USF1, USF2, USF2b	CCCGTG
RAT$LPK_01	911 (-)	0	100.00	USF-1, USF1, USF2, USF2b	CACGGG
RAT$A1I3_04	914 (+)	0	100.00		GTGCT
RAT$A1I3_04	914 (-)	0	100.00		AGCAC
RAT$VEGF_02	915 (-)	0	100.00	ER-alpha, ER-beta	GAGCA
TDNA$NOS_01	916 (-)	0	100.00	ASF-1, OBF3.1, TGA1a, TGA1b	TGAGC
MOUSE$SCC_05	918 (-)	1	87.50	SF-1	TAGCCTTGA
ALV$LTR_02	920 (-)	1	87.50	EFII	TGTAGTCTT
MAIZE$ADH2_03	924 (+)	0	100.00		CTACGG
SIF$CONS	925 (-)	0	91.67	SIF	CCCGTM
DAUCE$DC3_02	927 (-)	0	100.00	DPBF-1, DPBF-2	CACCCG
HS$BG_01	928 (-)	0	100.00	CACCC-binding factor	CACCC
HS$GG_13	928 (-)	0	100.00	gammaCAC1, gammaCAC2	CACCC
HS$GG_14	928 (-)	0	100.00	CACCC-binding factor, Sp1	CACCC
HS$GP2B_01	928 (-)	0	100.00	CACCC-binding factor	CACCC
RAT$TOA_02	928 (-)	0	100.00	CACCC-binding factor	CACCC
HS$EG_06	928 (-)	0	100.00	Sp1	CACCC
AS$CACCC_01	928 (-)	0	100.00	CACCC-binding factor	CACCC
XENLA$U2SN_11	930 (+)	0	100.00		GTGCC
EBV$IR4_05	930 (-)	0	100.00	R	GGCAC
EBV$IR4_06	930 (-)	0	100.00	R	GGCAC
Y$CTT1_02	931 (-)	1	87.50	repressor of CAR1 expression	CTGCAGGCT
Y$CYC1_16	931 (-)	0	100.00	MOT3	CAGGCA
HS$EGFR_19	932 (+)	0	100.00		GCCTGC
KER1$CONS	932 (+)	0	100.00	AP-2alphaA	GCCTGCAGGC
HS$EK14_01	932 (+)	0	100.00	AP-2alphaA	GCCTGCAGGC
KER1$CONS	932 (-)	0	100.00	AP-2alphaA	GCCTGCAGGC
HS$EK14_01	932 (-)	0	100.00	AP-2alphaA	GCCTGCAGGC
MOMLV$MOMULV_09	933 (+)	0	100.00	LVc	CCTGC
MOMLV$MOMULV_10	933 (+)	0	100.00	LVc	CCTGC
RADLV$RLV_03	933 (+)	0	100.00	LVc	CCTGC
Y$CTT1_02	934 (+)	1	87.50	repressor of CAR1 expression	CTGCAGGCT
HS$EGFR_19	936 (-)	0	100.00		GCCTGC
MOMLV$MOMULV_09	936 (-)	0	100.00	LVc	CCTGC
MOMLV$MOMULV_10	936 (-)	0	100.00	LVc	CCTGC
RADLV$RLV_03	936 (-)	0	100.00	LVc	CCTGC
HS$TPI_04	938 (+)	0	100.00	Sp1	AGGCGG
HS$CDC25C_04	939 (+)	0	100.00		GGCGG
AS$CF1_04	940 (+)	1	87.50	CF1	GGGGTCAGC
AS$CF1_32	940 (+)	1	87.50	CF1	GGGGTCAGC
HS$APOE_12	941 (+)	0	100.00	LXR-alpha, LXR-beta, RXR-alpha	CGGTCA
AS$ERR1_06	941 (+)	0	100.00	ERR1	CGGTCA
AS$MEIS1AHOXA9_08	941 (-)	0	100.00	HOXA9, Meis-1a	TGACCG
MOUSE$SREBP1C_01	941 (-)	0	100.00	LXR-alpha, LXR-beta, RXR-alpha	TGACCG
MOUSE$SREBP1C_03	941 (-)	0	100.00	LXR-alpha, RXR-alpha	TGACCG
CHICK$OA_03	942 (+)	0	100.00	ER-alpha	GGTCA
CHICK$OA_04	942 (+)	0	100.00	c-Fos, c-Jun, ER-alpha	GGTCA
AS$RAR_04	942 (+)	0	100.00	RAR-alpha1, T3R-alpha	GGTCA
AS$RAR_06	942 (+)	0	100.00	RAR-alpha1	GGTCA
AS$TR_08	942 (+)	0	100.00	T3R-alpha	GGTCA
AS$TR_09	942 (+)	0	100.00	RAR-alpha1, T3R-alpha	GGTCA
AS$TR_10	942 (+)	0	100.00	T3R-alpha	GGTCA
NT$CHN50_01	942 (+)	0	100.00	WRKY1, WRKY3, WRKY4	GGTCA
HS$ADH3_01	942 (-)	0	100.00	CAR, RAR-alpha1, RAR-beta, RXR-alpha	TGACC
CHICK$OA_14	942 (-)	0	100.00	ER-alpha	TGACC
RAT$EAI_09	943 (+)	0	100.00		GTCAG
DROME$ADH_06	948 (+)	0	100.00	Adf-1	CGTCG
AS$AREB6_42	952 (+)	0	100.00	AREB6	GTTTG
AS$AREB6_43	952 (+)	0	100.00	AREB6	GTTTG
AS$AREB6_45	952 (+)	0	100.00	AREB6	GTTTG
AS$AREB6_47	952 (+)	0	100.00	AREB6	GTTTG
AS$AREB6_51	952 (+)	0	100.00	AREB6	GTTTG
MOUSE$E2F1_03	953 (-)	1	87.50	E2F-1	CGCGGCAAA
AS$ZIC_16	955 (+)	1	87.50	Zic1	TGCGGGGGC
HS$TCRA_08	955 (-)	0	100.00	c-Ets-1, LEF-1, PEBP2alphaA1	CCGCA
MOUSE$HOX14_01	956 (+)	1	87.50	Krox-20, Krox-24	GCGGGGGCG
HS$HSP70_10	956 (+)	1	88.89	Sp1	GGGGCGGCGC
HSV1$TK_03	956 (+)	1	88.89	Sp1	GGGGCGGCGC
HS$IGF2_02	956 (+)	1	87.50	WT1	GCGGGGGCG
AS$NGFIC_01	956 (+)	1	87.50	NGFI-C	GCGGGGGCG
MOUSE$JUND_05	956 (+)	1	87.50	Sp1	GCGGGGGCG
RAT$IGF1R_04	956 (+)	1	87.50	WT1, WT1 -KTS, WT1 I, WT1 I -KTS	GCGGGGGCG

				WT1 I -KTS	
RAT$IGF1R_09	956 (+)	0	100.00	WT1 -KTS, WT1 I -KTS	GCGGCGGCG
CHICK$BLCR_02	956 (+)	0	100.00	NF-E4, Sp1	GCGGC
WTZFP$CONS	956 (-)	1	87.50	WT1, WT1 -KTS, WT1 I, WT1 I -KTS, WT1 I-del2, WT1-del2	CGCCCCCGC
HS$IGF2_01	956 (-)	1	87.50	WT1	CGCCCCCGC
HS$IGF2_03	956 (-)	1	87.50	WT1	CGCCCCCGC
MOUSE$CEBPA_02	957 (-)	0	100.00	CUP	GCCGCCG
HS$CDC25C_04	958 (+)	0	100.00		GGCGG
AS$GCCBOX_01	958 (-)	0	100.00	ERF2, ERF3, ERF4	GCCGCC
AS$GCCBOX_02	958 (-)	0	100.00	Pti4	GCCGCC
CHICK$BLCR_02	959 (+)	0	100.00	NF-E4, Sp1	GCGGC
MOUSE$JUND_09	962 (+)	1	87.50	Sp1	GCGCGGGCG
RAT$IGF1R_05	962 (-)	1	87.50	WT1 -KTS, WT1 I -KTS	CGCCCGCGC
HS$P53_04	962 (-)	1	87.50	Egr-1	CGCCTACGC
HS$EGFR_19	964 (-)	0	100.00		GCCTGC
MOMLV$MOMULV_09	964 (-)	0	100.00	LVc	CCTGC
MOMLV$MOMULV_10	964 (-)	0	100.00	LVc	CCTGC
RADLV$RLV_03	964 (-)	0	100.00	LVc	CCTGC
HSV1$TK_08	967 (+)	0	100.00	ZF5	GGCGCG
HS$CDC2_09	969 (+)	0	100.00		CGCGG
HS$CYCA_05	969 (+)	0	100.00		CGCGG
HS$CYCD1_14	969 (-)	0	100.00	Sp1, Sp2, Sp3, Sp4	CCGCG
MESAT$PRP2_04	970 (+)	1	87.50	Alfin1	GCGGTGCTG
HS$DHFR_08	972 (+)	1	87.50	Sp1	GGGGCGGGC
RAT$CYP24_02	972 (-)	0	100.00	VDR	CGCACC
MOUSE$JUND_09	973 (+)	1	87.50	Sp1	GCGCGGGCG
RAT$IGF1R_01	973 (+)	1	87.50	WT1, WT1 -KTS, WT1 I, WT1 I -KTS	GTGGGGGCG
HS$EGFR_09	973 (-)	1	90.00	ETF	GCCCCCCGCAC
RAT$IGF1R_05	973 (-)	1	87.50	WT1 -KTS, WT1 I -KTS	CGCCCGCGC
HS$TCRA_08	974 (-)	0	100.00	c-Ets-1, LEF-1, PEBP2alphaA1	CCGCA
CHICK$GATA1_09	975 (+)	1	88.89		GCGGGCGGGG
HS$RBG_04	976 (+)	1	90.00		CGGGCGGGGGA
MOUSE$SREBP1C_02	976 (-)	0	100.00	LXR-alpha, LXR-beta, RXR-alpha	CGCCCG
MOUSE$GSC_01	976 (-)	1	87.50	SMAD-4	GGCCGCCCG
HS$CYCD1_15	976 (-)	0	100.00	Sp1, Sp2, Sp3, Sp4	GCCCG
HS$APOE_08	977 (+)	0	100.00	Sp1	GGGCGG
HA$HMGCR_03	977 (+)	0	100.00		GGGCGG
HSV1$IE3_04	977 (+)	0	100.00	Sp1	GGGCGG
HSV1$IE3_07	977 (+)	0	100.00	Sp1	GGGCGG
HSV1$IE3_09	977 (+)	0	100.00	Sp1	GGGCGG
HSV1$IE4_01	977 (+)	0	100.00	Sp1	GGGCGG
HSV1$IE4_03	977 (+)	0	100.00	Sp1	GGGCGG
SV$SV40_06	977 (+)	0	100.00	LSF	GGGCGG
SV$SV40_07	977 (+)	0	100.00	ETF, Sp1	GGGCGG
SV$SV40_13	977 (+)	0	100.00	Sp1	GGGCGG
HS$U2SN_03	977 (+)	0	100.00	Sp1	GGGCGG
HS$U2SN_05	977 (+)	0	100.00	Sp1	GGGCGG
PIG$UPA_10	977 (+)	0	100.00	Sp1	GGGCGG
PIG$UPA_11	977 (+)	0	100.00	Sp1	GGGCGG
PIG$UPA_14	977 (+)	0	100.00	Sp1	GGGCGG
MOUSE$GLUT4_05	977 (+)	0	100.00	Sp1	GGGCGG
AD5$E1A_15	977 (+)	0	100.00	Sp1	GGGCGG
HS$NPY_01	977 (+)	0	100.00	Sp1	GGGCGG
RAT$IGF2_01	977 (+)	0	100.00	Sp1	GGGCGG
HS$TK_01	977 (+)	0	100.00	CP1	GGGCGG
HS$A24COL_03	977 (+)	0	100.00	Sp1	GGGCGG
HS$TK_07	977 (+)	0	100.00	Sp1	GGGCGG
CHICK$ACRA_07	977 (-)	0	100.00	Sp1	CCGCCC
MOUSE$APRT_01	977 (-)	0	100.00	Sp1	CCGCCC
RAT$GF_07	977 (-)	0	100.00	Sp1	CCGCCC
HA$HMGCR_03	977 (-)	0	100.00		CCGCCC
HSV1$IE3_05	977 (-)	0	100.00	Sp1	CCGCCC
HSV1$IE4_04	977 (-)	0	100.00	Sp1	CCGCCC
MOUSE$EPOR_02	977 (-)	0	100.00	Sp1	CCGCCC
RAT$CDC2_02	977 (-)	0	100.00		CCGCCC
MOUSE$E2F1_01	977 (-)	0	100.00		CCGCCC
HS$CD11B_01	977 (-)	0	100.00	Sp1	CGCCC
HS$TERT_04	977 (-)	0	100.00	Sp1	CCGCCC
HS$TERT_07	977 (-)	0	100.00	Sp1	CCGCCC
HS$CDC25C_04	978 (+)	0	100.00		GGCGG
AS$GCCBOX_01	978 (-)	0	100.00	ERF2, ERF3, ERF4	GCCGCC
AS$GCCBOX_02	978 (-)	0	100.00	Pti4	GCCGCC
CHICK$BLCR_02	979 (+)	0	100.00	NF-E4, Sp1	GCGGC
HS$CDC25C_04	981 (+)	0	100.00		GGCGG
Y$CHA1_01	982 (+)	0	100.00	CHA4	GCGGA
Y$CHA1_01	982 (-)	0	100.00	CHA4	TCCGC
PARS$PAL_04	984 (-)	0	100.00		CCGTCC
SIF$CONS	985 (-)	0	91.67	SIF	CCCGTM
HS$SREC_02	985 (-)	1	88.89	Sp1	CCCGCCCCTC
HS$DHFR_08	987 (+)	1	87.50	Sp1	GGGGCGGGC
MOUSE$SREBP1C_02	987 (-)	0	100.00	LXR-alpha, LXR-beta, RXR-alpha	CGCCCG
MOUSE$GSC_01	987 (-)	1	87.50	SMAD-4	GGCCGCCCG
HS$CYCD1_15	987 (-)	0	100.00	Sp1, Sp2, Sp3, Sp4	GCCCG
HS$APOE_08	988 (+)	0	100.00	Sp1	GGGCGG
HA$HMGCR_03	988 (+)	0	100.00		GGGCGG
HSV1$IE3_04	988 (+)	0	100.00	Sp1	GGGCGG
HSV1$IE3_07	988 (+)	0	100.00	Sp1	GGGCGG
HSV1$IE3_09	988 (+)	0	100.00	Sp1	GGGCGG
HSV1$IE4_01	988 (+)	0	100.00	Sp1	GGGCGG
HSV1$IE4_03	988 (+)	0	100.00	Sp1	GGGCGG
SV$SV40_06	988 (+)	0	100.00	LSF	GGGCGG
SV$SV40_07	988 (+)	0	100.00	ETF, Sp1	GGGCGG
SV$SV40_13	988 (+)	0	100.00	Sp1	GGGCGG
HS$U2SN_03	988 (+)	0	100.00	Sp1	GGGCGG
HS$U2SN_05	988 (+)	0	100.00	Sp1	GGGCGG
PIG$UPA_10	988 (+)	0	100.00	Sp1	GGGCGG
PIG$UPA_11	988 (+)	0	100.00	Sp1	GGGCGG
PIG$UPA_14	988 (+)	0	100.00	Sp1	GGGCGG
MOUSE$GLUT4_05	988 (+)	0	100.00	Sp1	GGGCGG
AD5$E1A_15	988 (+)	0	100.00	Sp1	GGGCGG
HS$NPY_01	988 (+)	0	100.00	Sp1	GGGCGG
RAT$IGF2_01	988 (+)	0	100.00	Sp1	GGGCGG
HS$TK_01	988 (+)	0	100.00	CP1	GGGCGG
MOUSE$JUND_09	988 (+)	1	87.50	Sp1	GCGCGGGCG
HS$A24COL_03	988 (+)	0	100.00	Sp1	GGGCGG
HS$TK_07	988 (+)	0	100.00	Sp1	GGGCGG
CHICK$ACRA_07	988 (-)	0	100.00	Sp1	CCGCCC
MOUSE$APRT_01	988 (-)	0	100.00	Sp1	CCGCCC
HS$APOE_09	988 (-)	0	100.00	Sp1	GCCCGCCC
RAT$GF_07	988 (-)	0	100.00	Sp1	CCGCCC
HA$HMGCR_03	988 (-)	0	100.00		CCGCCC
HSV1$IE3_05	988 (-)	0	100.00	Sp1	CCGCCC
HSV1$IE4_04	988 (-)	0	100.00	Sp1	CCGCCC
MOUSE$EPOR_02	988 (-)	0	100.00	Sp1	CCGCCC
RAT$CDC2_02	988 (-)	0	100.00		CCGCCC
MOUSE$E2F1_01	988 (-)	0	100.00		CCGCCC
HS$CD11B_01	988 (-)	0	100.00	Sp1	CGCCC
RAT$IGF1R_05	988 (-)	1	87.50	WT1 -KTS, WT1 I -KTS	CGCCCGCGC
HS$TERT_04	988 (-)	0	100.00	Sp1	CCGCCC
HS$TERT_07	988 (-)	0	100.00	Sp1	CCGCCC
HSV1$IE3_06	989 (+)	0	100.00	Sp1	GGCGGG
RAT$NF1_02	989 (+)	0	100.00	Sp1	GGCGGG
HS$NPY_02	989 (+)	0	100.00	Sp1	GGCGGG
HS$CDC25C_04	989 (+)	0	100.00		GGCGG
HS$MIP_02	989 (+)	0	100.00	Sp1	GGCGGG
HS$P21WAF1_06	989 (-)	0	100.00	SMAD-3, SMAD-4	CCCGCC
HS$P21WAF1_07	989 (-)	0	100.00		CCCGCC
RAT$IGF1R_07	990 (-)	1	87.50	WT1 -KTS, WT1 I -KTS	AGCCCCCGC
MOUSE$SREBP1C_02	991 (-)	0	100.00	LXR-alpha, LXR-beta, RXR-alpha	CGCCCG
HS$CYCD1_15	991 (-)	0	100.00	Sp1, Sp2, Sp3, Sp4	GCCCG
HS$APP_01	992 (+)	0	100.00		GGGCGC
AS$SPZ1_22	992 (+)	0	100.00	Spz1	GGGCGCTTC
HS$CD11B_01	992 (-)	0	100.00	Sp1	CGCCC
HS$PR264_08	1002 (-)	0	100.00	c-Myb	AAACGG
AS$AREB6_42	1004 (+)	0	100.00	AREB6	GTTTG
AS$AREB6_43	1004 (+)	0	100.00	AREB6	GTTTG
AS$AREB6_45	1004 (+)	0	100.00	AREB6	GTTTG
AS$AREB6_47	1004 (+)	0	100.00	AREB6	GTTTG
AS$AREB6_51	1004 (+)	0	100.00	AREB6	GTTTG
DROME$UBX_24	1005 (+)	0	100.00	LEF-1	TTTGA
MOUSE$MBP_04	1005 (-)	0	100.00	TFIID	TTCAAA
HS$CDC2_10	1006 (+)	0	100.00		TTGAA
HS$CYCA_06	1006 (+)	0	100.00		TTGAA
HS$CDC25C_05	1006 (+)	0	100.00		TTGAA
AS$ZIC_19	1011 (+)	1	87.50	Zic1	TGGGTGCGG
DROME$ADH_06	1013 (+)	0	100.00	Adf-1	GCTGC
HS$CMYC_16	1013 (+)	0	100.00	CTCF	GCTGC
DROME$ADH_14	1013 (-)	0	100.00	Adf-1	GCAGC
HS$MT2A_05	1013 (-)	1	87.50	AP-2, AP-2alphaA, AP-2alphaB, AP-2beta, AP-2gamma	GCCCGCGGC
HS$TCRA_08	1015 (-)	0	100.00	c-Ets-1, LEF-1, PEBP2alphaA1	CCGCA
HS$CYCD1_15	1017 (-)	0	100.00	Sp1, Sp2, Sp3, Sp4	GCCCG
HS$ASCC_01	1018 (+)	1	88.89	Sp1	GGGGCCGGGC
MOUSE$PAC1R_01	1018 (+)	0	100.00	ZAC-1a	GGGCCC
MOUSE$PAC1R_01	1018 (-)	0	100.00	ZAC-1a	GGGCCC
HS$CYCD1_15	1020 (+)	0	100.00	Sp1, Sp2, Sp3, Sp4	GCCCG
HS$ASCC_01	1020 (-)	1	88.89	Sp1	GGGGCCGGGC
HS$CYCD1_15	1023 (-)	0	100.00	Sp1, Sp2, Sp3, Sp4	GCCCG
MOUSE$PAC1R_01	1024 (+)	0	100.00	ZAC-1a	GGGCCC
MOUSE$PAC1R_01	1024 (-)	0	100.00	ZAC-1a	GGGCCC
CTCF$CONS	1027 (+)	0	100.00	CTCF	CCCTC
MOUSE$PBGD_01	1027 (+)	0	100.00	CAC-binding protein	CCCTCAC
MOUSE$M1H2KB_01	1027 (-)	1	90.00	ATF, CREB, RXR-beta	TGAGGTCAGGG
MOUSE$M1H2LD_01	1027 (-)	1	88.89	RAR-beta, RXR-beta	GAGGTCAGGG
HS$APOE_06	1028 (+)	1	87.50		CCCCACCTC
HS$PR264_01	1029 (-)	0	100.00	c-Myb	GGTGAG
HS$APOA2_06	1030 (+)	0	100.00		TCACC
HS$CAMHC_01	1030 (+)	0	100.00	Hp55, Hp65, p58, RAR-alpha1, RXR-beta, T3R-alpha, T3R-beta1, T3R-beta2	TCACCT
HS$CYP24_02	1030 (+)	0	100.00	VDR	TCACCT
RAT$CYP24_01	1030 (+)	0	100.00	VDR	TCACCT
HS$RARG_01	1030 (-)	0	100.00	RAR-alpha1, RAR-beta, RAR-gamma, RXR-alpha	AGGTGA
HSV1$TK_12	1030 (-)	0	100.00	RAR-alpha1, T3R-alpha	AGGTGA
MOUSE$IBABP_01	1030 (-)	0	100.00	FXR, RXR-alpha	AGGTGA
AS$ERR1_16	1030 (-)	0	100.00	ERR1	AGGTGA
RAT$ME_08	1030 (-)	0	100.00	PPAR-gamma, RXR-gamma	AGGTGA
DELTAEF1$CONS_01	1031 (+)	0	92.86	deltaEF1	CACCTNA
AS$ZIC_25	1033 (-)	1	87.50	Zic1	CGGGTGAGG
AS$RITA1_06	1034 (+)	1	88.89	RITA-1	CTCACGTGAG
AS$BZIPGB_06	1034 (+)	1	88.89	CPRF-2, Opaque-2, TGA1a	CTCACGTGAG
AS$AREB6_05	1034 (+)	1	87.50	AREB6	CACACCTGA
HS$PR264_01	1034 (-)	0	100.00	c-Myb	GGTGAG
AS$RITA1_06	1034 (-)	1	88.89	RITA-1	CTCACGTGAG
AS$BZIPGB_06	1034 (-)	1	88.89	CPRF-2, Opaque-2, TGA1a	CTCACGTGAG
HS$APOA2_06	1035 (+)	0	100.00		TCACC
HS$CAMHC_01	1035 (+)	0	100.00	Hp55, Hp65, p58, RAR-alpha1, RXR-beta, T3R-alpha, T3R-beta1, T3R-beta2	TCACCT
AS$AREB6_23	1035 (+)	0	100.00	AREB6	TCACCTGA
AS$AREB6_25	1035 (+)	0	100.00	AREB6	TCACCTGA
HS$CYP24_02	1035 (+)	0	100.00	VDR	TCACCT
RAT$CYP24_01	1035 (+)	0	100.00	VDR	TCACCT
HS$RARG_01	1035 (-)	0	100.00	RAR-alpha1, RAR-beta, RAR-gamma, RXR-alpha	AGGTGA
HSV1$TK_12	1035 (-)	0	100.00	RAR-alpha1, T3R-alpha	AGGTGA
MOUSE$IBABP_01	1035 (-)	0	100.00	FXR, RXR-alpha	AGGTGA
AS$ERR1_16	1035 (-)	0	100.00	ERR1	AGGTGA
RAT$ME_08	1035 (-)	0	100.00	PPAR-gamma, RXR-gamma	AGGTGA
MOUSE$ACRD_02	1036 (+)	0	100.00	E12, E47, MyoD, myogenin	CACCTG
XENLA$AC_05	1036 (+)	0	100.00	EMF1, MyoD	CACCTG
DELTAEF1$CONS_01	1036 (+)	0	92.86	deltaEF1	CACCTNA
HS$CXCR4_02	1036 (+)	0	100.00	c-Myc, USF2	CACCTG
PV$PHASL_02	1036 (+)	0	100.00	CAN	CACCTG
AD5$E1A_27	1036 (-)	1	87.50		TCTCAGGTG
MOUSE$CD4_03	1036 (-)	0	100.00	E12, HEB	CAGGTG
DROME$HB_12	1036 (-)	0	100.00	Ac, AS-C T3, Da, Sc	CAGGTG
DROME$AC_01	1036 (-)	0	100.00	Ac, AS-C T3, Da, Sc	CAGGTG
AS$SNAIL_01	1036 (-)	0	100.00	Sn	CAGGTG
AS$LMO2_01	1036 (-)	0	100.00	CLIM2, E12, E47, GATA-1, Lmo2, Tal-1	CAGGTG
HS$VEGF_02	1037 (-)	0	100.00		CAGGT
MOUSE$CRABP1_01	1042 (-)	0	100.00	TR2-11	GGACCT
HS$HH4_02	1043 (+)	0	100.00	H4TF-2	GGTCC
AS$TR_11	1043 (+)	0	100.00	T3R-alpha	GGTCC
MOUSE$GSC_01	1048 (+)	1	87.50	SMAD-4	GGCCGCCCG
HS$CAVEOLIN1_03	1048 (-)	0	100.00	Sp1	GCGGCC
Y$HOP1_01	1048 (-)	1	87.50		TGGGCGGCT
AS$GCCBOX_01	1049 (+)	0	100.00	ERF2, ERF3, ERF4	GCCGCC
AS$GCCBOX_02	1049 (+)	0	100.00	Pti4	GCCGCC
CHICK$BLCR_02	1049 (-)	0	100.00	NF-E4, Sp1	GCGGC
CHICK$ACRA_07	1050 (+)	0	100.00	Sp1	CCGCCC
MOUSE$APRT_01	1050 (+)	0	100.00	Sp1	CCGCCC
RAT$GF_07	1050 (+)	0	100.00	Sp1	CCGCCC
HA$HMGCR_03	1050 (+)	0	100.00		CCGCCC
HSV1$IE3_05	1050 (+)	0	100.00	Sp1	CCGCCC
HSV1$IE4_04	1050 (+)	0	100.00	Sp1	CCGCCC
MOUSE$EPOR_02	1050 (+)	0	100.00	Sp1	CCGCCC
RAT$CDC2_02	1050 (+)	0	100.00		CCGCCC
MOUSE$E2F1_01	1050 (+)	0	100.00		CCGCCC
HS$TERT_04	1050 (+)	0	100.00	Sp1	CCGCCC
HS$TERT_07	1050 (+)	0	100.00	Sp1	CCGCCC
HS$APOE_08	1050 (-)	0	100.00	Sp1	GGGCGG
HA$HMGCR_03	1050 (-)	0	100.00		GGGCGG
HSV1$IE3_04	1050 (-)	0	100.00	Sp1	GGGCGG
HSV1$IE3_07	1050 (-)	0	100.00	Sp1	GGGCGG
HSV1$IE3_09	1050 (-)	0	100.00	Sp1	GGGCGG
HSV1$IE4_01	1050 (-)	0	100.00	Sp1	GGGCGG
HSV1$IE4_03	1050 (-)	0	100.00	Sp1	GGGCGG
SV$SV40_06	1050 (-)	0	100.00	LSF	GGGCGG
SV$SV40_07	1050 (-)	0	100.00	ETF, Sp1	GGGCGG
SV$SV40_13	1050 (-)	0	100.00	Sp1	GGGCGG
HS$U2SN_03	1050 (-)	0	100.00	Sp1	GGGCGG
HS$U2SN_05	1050 (-)	0	100.00	Sp1	GGGCGG
PIG$UPA_10	1050 (-)	0	100.00	Sp1	GGGCGG
PIG$UPA_11	1050 (-)	0	100.00	Sp1	GGGCGG
PIG$UPA_14	1050 (-)	0	100.00	Sp1	GGGCGG
MOUSE$GLUT4_05	1050 (-)	0	100.00	Sp1	GGGCGG
AD5$E1A_15	1050 (-)	0	100.00	Sp1	GGGCGG
HS$NPY_01	1050 (-)	0	100.00	Sp1	GGGCGG
RAT$IGF2_01	1050 (-)	0	100.00	Sp1	GGGCGG
HS$TK_01	1050 (-)	0	100.00	CP1	GGGCGG
HS$CDC25C_04	1050 (-)	0	100.00		GGCGG
HS$A24COL_03	1050 (-)	0	100.00	Sp1	GGGCGG
HS$TK_07	1050 (-)	0	100.00	Sp1	GGGCGG
HS$CD11B_01	1051 (+)	0	100.00	Sp1	CGCCC
MOUSE$M1H2KB_03	1052 (+)	1	87.50	AP-2alphaA, AP-2alphaB	GCCCAGGGC
SV$SV40_63	1052 (-)	0	100.00	T-Ag	TGGGC
HS$MIP_05	1053 (+)	0	100.00	AP-2alphaA, AP-2alphaB	CCCAGGG
SV$SV40_37	1053 (-)	0	100.00		CTGGG
Y$GSY2_01	1054 (+)	0	100.00		CCAGGGG
MOUSE$THY1_05	1055 (-)	1	87.50		CCACCCCTG
AS$VDR_04	1056 (+)	0	100.00	VDR	AGGGG
Y$GLK1_02	1056 (+)	0	100.00	MSN2, MSN4	AGGGG
HS$AG_15	1057 (+)	0	100.00	CAC-binding protein	GGGGTG
AS$ZIC2_01	1057 (+)	1	87.50	Zic2	GGGGTGCTC
HS$BG_01	1058 (-)	0	100.00	CACCC-binding factor	CACCC
HS$GG_13	1058 (-)	0	100.00	gammaCAC1, gammaCAC2	CACCC
HS$GG_14	1058 (-)	0	100.00	CACCC-binding factor, Sp1	CACCC
HS$GP2B_01	1058 (-)	0	100.00	CACCC-binding factor	CACCC
RAT$TOA_02	1058 (-)	0	100.00	CACCC-binding factor	CACCC
HS$EG_06	1058 (-)	0	100.00	Sp1	CACCC
AS$CACCC_01	1058 (-)	0	100.00	CACCC-binding factor	CACCC
RAT$CYP24_02	1059 (-)	0	100.00	VDR	CGCACC
DROME$ADH_06	1071 (+)	0	100.00	Adf-1	CGTCG
HSV1$GD_01	1072 (-)	0	100.00	ICP4	CCGAC
HS$LCK_02	1072 (-)	1	87.50	LyF-1	CCTCCCAAC
AT$COR15A_01	1072 (-)	0	100.00	ANT, CBF1, CBF2, CBF3	CCGAC
AT$RD29B_01	1072 (-)	0	100.00	CBF1	CCGAC
AT$RD29A_01	1072 (-)	0	100.00	CBF1, DREB1A, DREB2A	CCGAC
AT$COR78_01	1072 (-)	0	100.00	ANT, CBF1, CBF2, CBF3	CCGAC
AT$COR15B_01	1072 (-)	0	100.00	CBF1, CBF2, CBF3	CCGAC
AS$ZIC3_14	1074 (+)	1	87.50	Zic3	CGGGAGGTT
MOUSE$SCC_06	1075 (+)	1	87.50	SF-1	GGGAGGTCA
HT1$HTLV1_08	1076 (-)	0	100.00	c-Ets-1 68	CCTCC
HS$RARG_01	1078 (+)	0	100.00	RAR-alpha1, RAR-beta, RAR-gamma, RXR-alpha	AGGTGA
HSV1$TK_12	1078 (+)	0	100.00	RAR-alpha1, T3R-alpha	AGGTGA
MOUSE$IBABP_01	1078 (+)	0	100.00	FXR, RXR-alpha	AGGTGA
AS$ERR1_16	1078 (+)	0	100.00	ERR1	AGGTGA
RAT$ME_08	1078 (+)	0	100.00	PPAR-gamma, RXR-gamma	AGGTGA
HS$CAMHC_01	1078 (-)	0	100.00	Hp55, Hp65, p58, RAR-alpha1, RXR-beta, T3R-alpha, T3R-beta1, T3R-beta2	TCACCT
HS$CYP24_02	1078 (-)	0	100.00	VDR	TCACCT
RAT$CYP24_01	1078 (-)	0	100.00	VDR	TCACCT
HS$APOA2_06	1079 (-)	0	100.00		TCACC
MOUSE$AFEP_13	1080 (+)	0	100.00	NP-III	GTGACC
HS$P53_03	1080 (+)	0	100.00	Pax-2, Pax-5, Pax-8	GTGAC
HS$MMP1_06	1080 (-)	0	100.00		GTCAC
HS$ADH3_01	1081 (+)	0	100.00	CAR, RAR-alpha1, RAR-beta, RXR-alpha	TGACC
CHICK$OA_14	1081 (+)	0	100.00	ER-alpha	TGACC
CHICK$OA_03	1081 (-)	0	100.00	ER-alpha	GGTCA
CHICK$OA_04	1081 (-)	0	100.00	c-Fos, c-Jun, ER-alpha	GGTCA
AS$RAR_04	1081 (-)	0	100.00	RAR-alpha1, T3R-alpha	GGTCA
AS$RAR_06	1081 (-)	0	100.00	RAR-alpha1	GGTCA
AS$TR_08	1081 (-)	0	100.00	T3R-alpha	GGTCA
AS$TR_09	1081 (-)	0	100.00	RAR-alpha1, T3R-alpha	GGTCA
AS$TR_10	1081 (-)	0	100.00	T3R-alpha	GGTCA
NT$CHN50_01	1081 (-)	0	100.00	WRKY1, WRKY3, WRKY4	GGTCA
RAT$CYP7A_05	1081 (-)	0	100.00	FXR, LXR-alpha, LXR-beta, RXR-alpha	TGGTCA
AS$ERR1_18	1081 (-)	0	100.00	ERR1	TGGTCA
AS$ZIC_27	1082 (-)	1	87.50	Zic1	CGACAGGTC
HS$EGFR_17	1083 (-)	0	100.00		ACTGGT
CHICK$STN1_02	1084 (+)	0	100.00	LBP-1, NF-1 (-like proteins)	CCAGT
HS$PAI_07	1084 (+)	0	100.00		CCAGT
CHICK$AAC_06	1087 (-)	0	100.00	delta factor, F-ACT1, MAPF2, YY1	GGCGAC
CHICK$AAC_07	1087 (-)	0	100.00	YY1	GGCGAC
FACT1$CONS	1088 (-)	0	100.00	F-ACT1	TGGCGA
WTZFP$CONS	1089 (+)	1	87.50	WT1, WT1 -KTS, WT1 I, WT1 I -KTS, WT1 I-del2, WT1-del2	CGCCCCCGC
HS$IGF2_01	1089 (+)	1	87.50	WT1	CGCCCCCGC
HS$IGF2_03	1089 (+)	1	87.50	WT1	CGCCCCCGC
MOUSE$HOX14_01	1089 (-)	1	87.50	Krox-20, Krox-24	GCGGGGGCG
HS$IGF2_02	1089 (-)	1	87.50	WT1	GCGGGGGCG
AS$NGFIC_01	1089 (-)	1	87.50	NGFI-C	GCGGGGGCG
MOUSE$JUND_05	1089 (-)	1	87.50	Sp1	GCGGGGGCG
RAT$IGF1R_04	1089 (-)	1	87.50	WT1, WT1 -KTS, WT1 I, WT1 I -KTS	GCGGGGGCG
RAT$IGF1R_09	1089 (-)	1	87.50	WT1 -KTS, WT1 I -KTS	GCGGCGGCG
HS$TPI_01	1090 (-)	1	90.00	Sp1	GGGGCGGCGGC
HS$RAS1_05	1091 (+)	1	88.89	Sp1	CCCCCGCCCC
HT1$HTLV1_08	1091 (+)	0	100.00	c-Ets-1 68	CCACC
HS$BG_52	1091 (+)	0	100.00		CCACC
HS$BG_14	1091 (+)	0	100.00		CCACC
HS$BG_44	1091 (+)	0	100.00	CAC-binding protein	CCACC
BPV1$BPV1_24	1091 (-)	0	100.00	Sp1	GGTGG
HS$BG_48	1091 (-)	0	100.00	CAC-binding protein	GGTGG
MOUSE$CEBPA_06	1091 (-)	0	100.00	Sp1	GGTGG
AS$ALFIN1_01	1091 (-)	1	87.50	Alfin1	GAGCGGTGG
HS$ZG_06	1092 (+)	1	87.50	Sp1	CACCACCCC
HS$MT2A_10	1092 (-)	1	87.50	Sp1	GGGGCGGGG
RAT$GSTP_09	1092 (-)	1	87.50		GGGGCGGGG
MOUSE$PFK_05	1092 (-)	1	87.50		GGGGCGGGG
HS$FN_07	1092 (-)	1	87.50	Sp1	GGGGCGGGG
AS$ZIC2_27	1092 (-)	1	87.50	Zic2	GGGCCGGTG
AS$ZIC3_17	1092 (-)	1	87.50	Zic3	GGGGGGGTG
CHICK$ACRA_07	1094 (+)	0	100.00	Sp1	CCGCCC
MOUSE$APRT_01	1094 (+)	0	100.00	Sp1	CCGCCC
RAT$GF_07	1094 (+)	0	100.00	Sp1	CCGCCC
HA$HMGCR_03	1094 (+)	0	100.00		CCGCCC
HSV1$IE3_05	1094 (+)	0	100.00	Sp1	CCGCCC
HSV1$IE4_04	1094 (+)	0	100.00	Sp1	CCGCCC
HS$TGFB1_03	1094 (+)	0	100.00	Sp1	CCGCCCCC
MOUSE$EPOR_02	1094 (+)	0	100.00	Sp1	CCGCCC
RAT$CDC2_02	1094 (+)	0	100.00		CCGCCC
MOUSE$E2F1_01	1094 (+)	0	100.00		CCGCCC
HS$TK_08	1094 (+)	0	100.00	Sp1	CCGCCCC
HS$TERT_04	1094 (+)	0	100.00	Sp1	CCGCCC
HS$TERT_07	1094 (+)	0	100.00	Sp1	CCGCCC
HS$APOE_08	1094 (-)	0	100.00	Sp1	GGGCGG
HA$HMGCR_03	1094 (-)	0	100.00		GGGCGG
HSV1$IE3_04	1094 (-)	0	100.00	Sp1	GGGCGG
HSV1$IE3_07	1094 (-)	0	100.00	Sp1	GGGCGG
HSV1$IE3_09	1094 (-)	0	100.00	Sp1	GGGCGG
HSV1$IE4_01	1094 (-)	0	100.00	Sp1	GGGCGG
HSV1$IE4_03	1094 (-)	0	100.00	Sp1	GGGCGG
RAT$MT1_01	1094 (-)	0	100.00	Sp1	GGGGGCGG
SV$SV40_06	1094 (-)	0	100.00	LSF	GGGCGG
SV$SV40_07	1094 (-)	0	100.00	ETF, Sp1	GGGCGG
SV$SV40_13	1094 (-)	0	100.00	Sp1	GGGCGG
HS$U2SN_03	1094 (-)	0	100.00	Sp1	GGGCGG
HS$U2SN_05	1094 (-)	0	100.00	Sp1	GGGCGG
PIG$UPA_10	1094 (-)	0	100.00	Sp1	GGGCGG
PIG$UPA_11	1094 (-)	0	100.00	Sp1	GGGCGG
PIG$UPA_14	1094 (-)	0	100.00	Sp1	GGGCGG
MOUSE$GLUT4_05	1094 (-)	0	100.00	Sp1	GGGCGG
AD5$E1A_15	1094 (-)	0	100.00	Sp1	GGGCGG
HS$NPY_01	1094 (-)	0	100.00	Sp1	GGGCGG
RAT$IGF2_01	1094 (-)	0	100.00	Sp1	GGGCGG
HS$TK_01	1094 (-)	0	100.00	CP1	GGGCGG
MOUSE$ECADH_02	1094 (-)	0	100.00	Sp1	GGGGGCGG
HS$CDC25C_04	1094 (-)	0	100.00		GGCGG
HS$A24COL_03	1094 (-)	0	100.00	Sp1	GGGCGG
HS$TK_05	1094 (-)	0	100.00	Sp1	GGGGGCGG
HS$TK_07	1094 (-)	0	100.00	Sp1	GGGCGG
RAT$HSC70_01	1094 (-)	0	100.00	Sp1, Sp3	GGGGCGG
YL$XPR2_03	1095 (+)	0	100.00		CGCCCCC
HS$CD11B_01	1095 (+)	0	100.00	Sp1	CGCCC
PA$PY_26	1096 (+)	0	100.00	T-Ag	GCCCC
MAIZE$ADH11S_06	1096 (+)	0	100.00	GCBP-1, Sp1	GCCCC
PA$PY_23	1096 (-)	0	100.00	T-Ag	GGGGC
SV$SV40_04	1096 (-)	0	100.00	T-Ag	GGGGC
SV$SV40_63	1096 (-)	0	100.00	T-Ag	GGGGC
HS$CYCD1_14	1097 (+)	0	100.00	Sp1, Sp2, Sp3, Sp4	CCCCC
HS$CYCD1_15	1097 (+)	0	100.00	Sp1, Sp2, Sp3, Sp4	CCCCC

Number of sites found: 2323

Start a new search
